# Correction: Semiquantitative Analysis of Clinical Heat Stress in *Clostridium difficile* Strain 630 Using a GeLC/MS Workflow with emPAI Quantitation

**DOI:** 10.1371/journal.pone.0097413

**Published:** 2014-05-07

**Authors:** The PLOS ONE Staff

## Notice of Republication

This article was republished on April 18 2014, to correct the spelling error in the title. Please download this article again to view the correct version. The originally published, uncorrected article and the republished, corrected article are provided here for reference.

## Supporting Information

File S1 Originally published, uncorrected article.
(PDF)

File S2 Republished corrected article.
(PDF)

## References

[1] Ternan NG, Jain S, Graham RLJ, McMullan G (2014) Semiquantitative Analysis of Clinical Heat Stress in Clostridium difficile Strain 630 Using a GeLC/MS Workflow with emPAI Quantitatio. PLoS ONE 9(2): e88960 doi:10.1371/journal.pone.0088960 24586458 10.1371/journal.pone.0088960 PMC3933415

