# Supplementary material for: Semiquantitative Analysis of Clinical Heat Stress in Clostridium difficile Strain 630 Using a GeLC/MS Workflow with emPAI Quantitation
Source: PLoS One. 2014 Feb 24;9(2):e88960. doi: 10.1371/journal.pone.0088960 (PMC3933415; doi:10.1371/journal.pone.0088960)
Supplement: File S1 — PROVALT output html file from 37°C single lane GeLC/MS, single injection, 177 proteins. (HTML) [file pone.0088960.s001.html]

   Complete output   Complete output    
 
   
  Protein Group 1   
      Expression Quality:  
         Score      Num Spectra      Num Peptides      High-Qual Peptides      % Coverage       2033    98    38    30    37   
   
      Peptides:   
        Query    Observed    Mr(expt)    Mr(calc)    Score    Peptide    Result File   
		    330    1051.42    2100.83    2100.04    67    ALASQALSIFGDHQDVMAAR    37Rep_8   
		    383    706.53    2116.57    2116.04    37    ALASQALSIFGDHQDVMAAR +Oxidation (M)    37RepA_1   
		    271    892.12    1782.22    1781.83    42    ATYLIDEADYIACHK    37Rep_8   
		    172    767.05    1532.08    1531.75    62    EPGSTGEPLYLDVR    37RepA_8   
		    1    403.43    804.85    804.41    38    FGDTPIR    37RepA_8   
		    42    471.39    940.76    940.50    32    FYTVNAVK    37RepA_8   
		    383    1335.93    2669.84    2669.17    32    GPSWANSLFEDNAEYGFGMYTAVK +Oxidation (M)    37RepA_8   
		    169    756.03    1510.04    1509.72    53    GTAQNPDIYFQTR    37RepA_8   
		    150    702.02    1402.03    1401.67    36    HFLDAMPSTVER    37RepA_8   
		    155    709.96    1417.90    1417.67    34    HFLDAMPSTVER +Oxidation (M)    37RepA_8   
		    170    758.56    1515.11    1514.67    81    HSLFDYYGAEDAK    37RepA_8   
		    419    779.53    2335.56    2335.01    61    IQVSPLDCTGCGNCADICPAK    37RepA_1   
		    322    1036.79    2071.57    2070.92    58    IVDAMTELVSMDICEDAK +2 Oxidation (M)    37Rep_8   
		    320    1028.79    2055.57    2054.92    66    IVDAMTELVSMDICEDAK +Oxidation (M)    37Rep_8   
		    292    938.75    1875.48    1874.99    106    IVNMNYAAVDAGINALVK    37Rep_8   
		    351    946.72    1891.43    1890.99    66    IVNMNYAAVDAGINALVK +Oxidation (M)    37RepA_1   
		    243    822.63    1643.24    1642.76    82    KHSLFDYYGAEDAK    37Rep_8   
		    193    663.98    1325.94    1325.71    34    LAEIIPEEDAVK    37RepA_1   
		    73    528.92    1055.82    1055.57    53    LGQEIGLGNR    37Rep_7   
		    193    698.50    1394.98    1394.71    52    LPFIHFFDGFR    37Rep_8   
		    408    1202.86    2403.71    2403.22    44    NGFTVGIVDDVTNTSLTPSEPIK    37Rep_8   
		    475    1310.65    3928.93    3927.84    46    NILRPMTAQEGNNLPVSTFNGIEDGTFPCGTAAYEK +Oxidation (M)    37RepA_8   
		    57    461.33    920.65    920.46    46    NPFTLDSK    37Rep_8   
		    252    841.71    1681.41    1680.84    48    NPFTLDSKEPTASFK    37Rep_8   
		    351    1095.25    2188.48    2188.96    42    QPLMEFSGACAGCGETAYIK    37Rep_8   
		    353    1103.81    2205.61    2204.95    50    QPLMEFSGACAGCGETAYIK +Oxidation (M)    37Rep_8   
		    59    529.86    1057.70    1057.53    68    SGGITMSHLR    37RepA_8   
		    265    588.14    1761.41    1760.89    72    TKEPGSTGEPLYLDVR    37Rep_7   
		    270    1055.40    2108.78    2107.99    49    TVANEAQAVACGYWHLYR    37RepA_8   
		    213    688.43    1374.85    1375.70    39    TVFDNLVSEQPK    37RepA_1   
		    77    545.39    1088.77    1088.55    43    VDVMPANTVK +Oxidation (M)    37Rep_7   
		    373    857.67    2569.99    2569.27    60    VELLENEDYASLLNFEAVQAFR    37RepA_8   
		    188    815.59    1629.16    1628.77    58    VEVPASWENAVDADK    37RepA_8   
		    300    953.67    1905.32    1904.89    41    VVELLEKPACDCTDEK    37Rep_8   
		    104    525.84    1049.66    1049.55    49    VVTQLYGDR    37RepA_1   
		    490    685.95    1369.89    1369.58    71    YAQAYFDYDSK    37Rep_1   
		    167    750.00    1497.99    1497.68    60    YAQAYFDYDSKK    37RepA_8   
		    135    663.41    1324.81    1324.70    55    YYQNIVGIVEK    37RepA_7   
   
      Matching Genes:  
               gi|115251733|emb|CAJ69568.1|  (pyruvate-flavodoxin oxidoreductase [Clostridium difficile 630]) 
           
  Protein Group 2   
      Expression Quality:  
         Score      Num Spectra      Num Peptides      High-Qual Peptides      % Coverage       1049    43    22    17    51   
   
      Peptides:   
        Query    Observed    Mr(expt)    Mr(calc)    Score    Peptide    Result File   
		    347    643.50    1927.49    1927.01    56    DKYPGLIFSQILGYGEK    37Rep_5   
		    39    462.91    923.80    923.51    51    EGVEILHK    37RepA_5   
		    193    697.98    1393.95    1393.65    28    ENPNSPLMTTYK    37Rep_5   
		    148    705.95    1409.88    1409.65    30    ENPNSPLMTTYK +Oxidation (M)    37RepA_6   
		    76    511.36    1020.70    1020.49    58    GGVSQSVMEK    37Rep_5   
		    535    937.75    2810.22    2809.41    31    GTSPANTAAGFGDHYAGLALAAGSLAALHK    37RepA_5   
		    79    549.87    1097.73    1097.57    50    IEPIEGDGIR    37Rep_7   
		    467    1279.41    2556.81    2556.15    48    IQSCEDLLDDEQAWANDFLFK    37RepA_5   
		    55    495.53    989.05    988.53    50    IVGEAMLEK    37RepA_5   
		    58    503.41    1004.80    1004.52    34    IVGEAMLEK +Oxidation (M)    37RepA_5   
		    197    739.12    1476.22    1475.80    53    LLSEADIFVTNVR    37RepA_5   
		    62    519.99    1037.97    1037.52    56    MGIAYDQIK    37RepA_5   
		    66    527.97    1053.93    1053.52    43    MGIAYDQIK +Oxidation (M)    37RepA_5   
		    94    609.99    1217.96    1217.61    41    MLGDWGAEVIK    37RepA_5   
		    119    617.97    1233.93    1233.61    30    MLGDWGAEVIK +Oxidation (M)    37Rep_5   
		    362    997.69    1993.36    1992.84    73    SPASDDENPMFELENGNK    37Rep_5   
		    340    1005.68    2009.34    2008.83    46    SPASDDENPMFELENGNK +Oxidation (M)    37RepA_5   
		    393    1061.78    2121.55    2120.93    57    SPASDDENPMFELENGNKK    37Rep_5   
		    403    1069.72    2137.42    2136.93    50    SPASDDENPMFELENGNKK +Oxidation (M)    37Rep_5   
		    359    702.86    2105.55    2105.02    66    TLDEWSALLEEADLPFEK    37RepA_5   
		    89    555.49    1108.96    1108.62    41    VGQHTVEVLK    37Rep_5   
		    190    695.56    1389.11    1388.78    57    WIQLALIQYNK    37Rep_5   
   
      Matching Genes:  
               gi|115249401|emb|CAJ67216.1|  (isocaprenoyl-CoA:2-hydroxyisocaproate CoA-transferase [Clostridium difficile 630]) 
           
  Protein Group 3   
      Expression Quality:  
         Score      Num Spectra      Num Peptides      High-Qual Peptides      % Coverage       984    42    18    14    41   
   
      Peptides:   
        Query    Observed    Mr(expt)    Mr(calc)    Score    Peptide    Result File   
		    322    680.66    2038.96    2038.93    84    AAQEQQAAQGAEQAQDNGPK    37RepA_7   
		    506    1053.76    3158.24    3157.42    62    AAQEQQAAQGAEQAQDNGPKDDNVVDADFK    37RepA_7   
		    270    927.17    1852.32    1851.80    57    DDNVVDADFKEVDEDK    37RepA_7   
		    160    681.51    1361.01    1360.65    53    DNQDATAEELKK    37Rep_7   
		    342    1065.17    2128.34    2128.03    71    EKIEAFNQAESTIYQTEK    37RepA_7   
		    449    1204.95    2407.88    2407.19    53    ELSSTMSSNINLPFITATAEGPK    37Rep_7   
		    120    643.34    1284.67    1284.68    51    FQLTDIPPAQR    37RepA_7   
		    55    484.37    966.73    966.52    47    HLNIDLSR    37Rep_7   
		    285    936.82    1871.63    1870.89    26    IEAFNQAESTIYQTEK    37RepA_4   
		    248    847.67    1693.32    1692.84    52    IINEPTAAALAYGMDK +Oxidation (M)    37RepA_7   
		    50    477.89    953.77    953.55    30    IPAVQEAVK    37Rep_7   
		    107    412.29    1233.85    1233.61    28    ISSGEKEDIEK    37Rep_7   
		    290    946.32    1890.62    1889.96    83    ITITSNTNLSEAEIEQK    37RepA_7   
		    540    978.51    2932.49    2931.41    62    SDAESYLGQTVTEAVITVPAYFTDAQR    37Rep_7   
		    475    834.30    2499.89    2499.24    77    SQIFSTAADNQTAVDIHVLQGER    37Rep_7   
		    228    796.13    1590.24    1589.87    38    SYTPQEISAIILQK    37Rep_7   
		    433    1187.86    2373.71    2373.20    57    TALQDAGLSTGDIDDVLLVGGSTR    37Rep_7   
		    38    445.34    888.66    888.46    53    TLNELGDK    37Rep_7   
   
      Matching Genes:  
               gi|115251515|emb|CAJ69348.1|  (chaperone protein [Clostridium difficile 630]) 
           
  Protein Group 4   
      Expression Quality:  
         Score      Num Spectra      Num Peptides      High-Qual Peptides      % Coverage       962    31    20    15    34   
   
      Peptides:   
        Query    Observed    Mr(expt)    Mr(calc)    Score    Peptide    Result File   
		    372    1086.32    2170.62    2170.04    53    ATYTMIFDHYEQVPASVAK    37Rep_7   
		    358    1094.17    2186.32    2186.04    42    ATYTMIFDHYEQVPASVAK +Oxidation (M)    37RepA_7   
		    157    682.00    1361.99    1361.74    59    EDSFIGIIDLLK    37RepA_7   
		    157    679.44    1356.87    1356.64    40    GGVEPQSENVWR    37Rep_7   
		    109    626.19    1250.36    1249.67    41    GNAVLLEPYFK    37RepA_7   
		    221    783.09    1564.17    1563.72    50    HSSDEEPFSALAFK    37Rep_7   
		    350    713.36    2137.06    2136.87    33    IGETHEGASQMDWMEQEK +2 Oxidation (M)    37RepA_7   
		    456    808.54    2422.59    2422.02    33    IGETHEGASQMDWMEQEKER +2 Oxidation (M)    37Rep_7   
		    447    803.26    2406.76    2406.02    30    IGETHEGASQMDWMEQEKER +Oxidation (M)    37Rep_7   
		    94    604.29    1206.57    1206.64    30    ILFYTGQTHK    37RepA_7   
		    344    685.48    2053.42    2053.05    39    INIIDTPGHVDFTVEVER    37Rep_7   
		    116    625.45    1248.88    1248.62    67    LAEEDPTFTVK    37Rep_7   
		    199    749.64    1497.27    1496.80    51    LNSNAVPMQLPIGK +Oxidation (M)    37RepA_7   
		    262    878.63    1755.24    1754.80    65    LVESVAETDEELMMK +2 Oxidation (M)    37Rep_7   
		    65    510.86    1019.71    1019.50    48    QAETYGVPR    37RepA_7   
		    476    1251.97    2501.93    2501.23    65    SGAQVINAFVPLSEMFGYSTDLR    37Rep_7   
		    87    587.88    1173.75    1173.57    42    VAPQEPGEGYK    37RepA_7   
		    51    480.86    959.70    959.52    45    VGAPQVAYR    37Rep_7   
		    115    624.56    1247.10    1246.69    60    VYAGDIAAAVGLK    37Rep_7   
		    258    895.26    1788.51    1787.89    69    VYSGTLESGSYVLNATK    37RepA_7   
   
      Matching Genes:  
               gi|115249074|emb|CAJ66885.1|  (translation elongation factor G [Clostridium difficile 630]) 
           
  Protein Group 5   
      Expression Quality:  
         Score      Num Spectra      Num Peptides      High-Qual Peptides      % Coverage       937    48    18    13    41   
   
      Peptides:   
        Query    Observed    Mr(expt)    Mr(calc)    Score    Peptide    Result File   
		    71    498.28    994.55    994.52    62    AEAHIQAGAK    37Rep_5   
		    85    505.93    1009.84    1009.65    58    AIGLVIPSLK    37Rep_4   
		    122    619.89    1237.77    1237.64    53    DKAEAHIQAGAK    37Rep_5   
		    180    718.16    1434.31    1433.74    93    FEVVAINDLTDAK    37RepA_5   
		    472    1212.34    2422.66    2423.15    25    GLMTTIHAYTNDQNTLDGPHPK    37Rep_5   
		    511    814.35    2440.03    2439.15    48    GLMTTIHAYTNDQNTLDGPHPK +Oxidation (M)    37Rep_4   
		    180    650.00    1297.99    1297.76    71    KVVISAPATGDLK    37Rep_4   
		    33    430.31    858.60    858.48    37    MLAHLFK    37Rep_4   
		    19    438.38    874.74    874.47    34    MLAHLFK +Oxidation (M)    37RepA_5   
		    452    1163.36    2324.70    2324.10    58    MMEQQDKFEVVAINDLTDAK    37Rep_5   
		    436    1179.45    2356.88    2356.09    59    MMEQQDKFEVVAINDLTDAK +2 Oxidation (M)    37RepA_5   
		    449    1171.41    2340.80    2340.10    33    MMEQQDKFEVVAINDLTDAK +Oxidation (M)    37Rep_6   
		    104    556.47    1110.93    1110.61    45    TLGYFAQLAK    37Rep_4   
		    124    589.92    1177.82    1177.63    30    VLNDKYGIEK    37Rep_4   
		    483    887.17    1772.33    1771.98    54    VPVVTGSITELVCTLGK    37Rep_2   
		    123    585.96    1169.90    1169.67    60    VVISAPATGDLK    37Rep_4   
		    308    1046.30    2090.59    2089.98    43    VVSWYDNEMSYTSQLIR    37RepA_6   
		    424    1054.34    2106.66    2105.97    74    VVSWYDNEMSYTSQLIR +Oxidation (M)    37Rep_4   
   
      Matching Genes:  
               gi|115252231|emb|CAJ70071.1|  (glyceraldehyde-3-phosphate dehydrogenase 2 [Clostridium difficile 630]) 
           
  Protein Group 6   
      Expression Quality:  
         Score      Num Spectra      Num Peptides      High-Qual Peptides      % Coverage       778    23    14    11    20   
   
      Peptides:   
        Query    Observed    Mr(expt)    Mr(calc)    Score    Peptide    Result File   
		    163    688.59    1375.17    1374.78    35    FQSLLVAIEDLK    37RepA_7   
		    318    1011.09    2020.17    2019.92    44    HTTFFEVEPDPTLECAK    37RepA_7   
		    178    714.98    1427.95    1427.69    59    IFATYSQEQVDK    37Rep_7   
		    79    560.08    1118.14    1117.65    73    IFLAASLAANK    37RepA_7   
		    337    1019.26    2036.51    2036.06    51    ILINTPSSQGGIGDLYNFK    37Rep_7   
		    85    581.34    1160.67    1160.62    82    IVGQTACTIAK    37RepA_7   
		    418    1161.82    2321.62    2321.09    63    LVEDGGFGHTSSLYIDDVNQR    37Rep_7   
		    242    818.62    1635.23    1634.74    66    NHYASEYIYNAYK    37Rep_7   
		    67    512.93    1023.84    1023.54    34    SIFEYLPR    37Rep_7   
		    66    516.32    1030.62    1030.60    71    TAVNSILVSK    37RepA_7   
		    388    710.19    2127.56    2127.07    53    VLIGEVESVEIEEAFAHEK    37RepA_1   
		    54    485.90    969.79    969.57    44    VPLAIMAQK    37RepA_7   
		    61    493.90    985.78    985.56    33    VPLAIMAQK +Oxidation (M)    37Rep_7   
		    88    570.49    1138.96    1138.64    70    YAGIASFLGLK    37Rep_7   
   
      Matching Genes:  
               gi|115252023|emb|CAJ69859.1|  (aldehyde-alcohol dehydrogenase [includes: alcohol dehydrogenase and pyruvate-formate-lyase deactivase [Clostridium difficile 630]) 
           
  Protein Group 7   
      Expression Quality:  
         Score      Num Spectra      Num Peptides      High-Qual Peptides      % Coverage       762    40    15    14    40   
   
      Peptides:   
        Query    Observed    Mr(expt)    Mr(calc)    Score    Peptide    Result File   
		    528    1270.92    2539.82    2539.30    72    AYEGGFAIGAFNISDLEQLQGVLK    37Rep_4   
		    197    530.36    1058.70    1058.56    55    DAIQAVVESK    37Rep_2   
		    89    611.50    1220.99    1220.63    56    FDILEEIQSK    37RepA_4   
		    26    409.76    817.50    817.43    41    FLAENPK    37Rep_1   
		    46    489.37    976.73    976.46    52    INMDTDLR    37RepA_4   
		    236    497.24    992.46    992.46    41    INMDTDLR +Oxidation (M)    37Rep_1   
		    69    473.87    945.72    945.53    50    KFLAENPK    37Rep_4   
		    25    423.83    845.64    845.48    47    LAMTAAIR    37Rep_4   
		    44    431.79    861.57    861.47    39    LAMTAAIR +Oxidation (M)    37RepA_1   
		    261    781.01    1560.01    1559.73    51    NSYVMIQASMSAVK +2 Oxidation (M)    37Rep_4   
		    189    773.11    1544.21    1543.74    43    NSYVMIQASMSAVK +Oxidation (M)    37RepA_4   
		    249    873.31    1744.60    1743.92    56    TGVDSLAIAIGTSHGAFK    37RepA_4   
		    152    672.99    1343.96    1343.69    43    YAGPHTLVEMVK    37RepA_4   
		    211    680.97    1359.93    1359.69    44    YAGPHTLVEMVK +Oxidation (M)    37Rep_4   
		    367    770.02    1538.02    1537.74    72    YTQPAEAVEFVER    37Rep_3   
   
      Matching Genes:  
               gi|115249409|emb|CAJ67224.1|  (putative fructose-bisphosphate aldolase [Clostridium difficile 630]) 
           
  Protein Group 8   
      Expression Quality:  
         Score      Num Spectra      Num Peptides      High-Qual Peptides      % Coverage       748    32    17    11    41   
   
      Peptides:   
        Query    Observed    Mr(expt)    Mr(calc)    Score    Peptide    Result File   
		    272    880.24    1758.47    1757.88    60    ALQSGTSHFLGQHFTK    37Rep_6   
		    125    668.95    1335.88    1335.62    50    DIENNQAMVFR    37RepA_6   
		    132    677.01    1352.01    1351.62    48    DIENNQAMVFR +Oxidation (M)    37RepA_6   
		    78    548.86    1095.70    1095.49    32    EADSMVVMAK +Oxidation (M)    37Rep_6   
		    427    1132.81    2263.61    2263.07    56    EAEHVEGFAPEVAWVTHGGNK    37Rep_6   
		    264    858.09    1714.17    1713.72    40    EDNTSIVENMDEFR +Oxidation (M)    37Rep_6   
		    285    914.16    1826.30    1825.82    36    EDNTSIVENMDEFRK    37Rep_6   
		    288    931.18    1860.35    1859.86    60    EGNLANPYHTSWGASTR    37Rep_8   
		    79    559.41    1116.80    1116.61    42    IKEETGATIR    37Rep_6   
		    309    944.75    1887.48    1886.84    37    MEDDFPQWYTDVITK    37Rep_6   
		    66    497.45    992.88    992.52    43    QFVEEITK    37Rep_6   
		    97    560.95    1119.89    1119.58    63    TDLVDYAPVK    37Rep_8   
		    209    737.41    1472.81    1472.54    40    TMWCGDAECEAK +Oxidation (M)    37Rep_6   
		    470    1235.37    3703.09    3702.73    29    TSEFLWQEGHTLHETAEEAQEETIQQLEVYK    37RepA_8   
		    194    714.91    1427.80    1427.61    36    TYTIEAMMHDGK +2 Oxidation (M)    37Rep_6   
		    149    707.01    1412.01    1411.61    40    TYTIEAMMHDGK +Oxidation (M)    37RepA_6   
		    151    709.71    1417.40    1416.91    36    VAPIQVVIVPIAAK    37RepA_6   
   
      Matching Genes:  
               gi|115249053|emb|CAJ66864.1|  (putative dual-specificity prolyl/cysteinyl-tRNA synthetase [Clostridium difficile 630]) 
           
  Protein Group 9   
      Expression Quality:  
         Score      Num Spectra      Num Peptides      High-Qual Peptides      % Coverage       733    53    13    10    61   
   
      Peptides:   
        Query    Observed    Mr(expt)    Mr(calc)    Score    Peptide    Result File   
		    171    452.32    902.62    902.53    45    AFLGLLNR    37Rep_1   
		    301    628.39    1254.77    1254.58    51    EGYPEVAEAYK    37Rep_2   
		    472    942.64    1883.27    1882.86    28    ELGLDAIHDTVHEMCK +Oxidation (M)    37Rep_3   
		    424    785.91    2354.72    2354.06    25    ELGLDAIHDTVHEMCKDEAR +Oxidation (M)    37RepA_3   
		    350    746.06    1490.11    1489.80    105    FAELLGEVVVADTK    37Rep_3   
		    219    615.34    1228.67    1228.53    42    GEMVWADEHR    37Rep_3   
		    164    415.94    1244.80    1244.52    27    GEMVWADEHR +Oxidation (M)    37RepA_2   
		    140    608.45    1214.88    1214.59    68    IAFEEAEHAAK    37RepA_2   
		    447    820.62    1639.22    1638.89    88    IGVAQGVDAEIIEGLR    37Rep_2   
		    219    563.34    1124.66    1124.50    67    VDAEYGATDGK    37Rep_2   
		    227    577.29    1152.57    1152.55    62    VGADKFEEMK    37Rep_2   
		    195    585.37    1168.72    1168.54    43    VGADKFEEMK +Oxidation (M)    37Rep_3   
		    307    690.90    1379.78    1379.67    82    VRVDAEYGATDGK    37Rep_3   
   
      Matching Genes:  
               gi|115250565|emb|CAJ68389.1|  (putative rubrerythrin [Clostridium difficile 630]) 
           
  Protein Group 10   
      Expression Quality:  
         Score      Num Spectra      Num Peptides      High-Qual Peptides      % Coverage       720    34    13    12    43   
   
      Peptides:   
        Query    Observed    Mr(expt)    Mr(calc)    Score    Peptide    Result File   
		    401    742.96    2225.85    2225.15    45    DAIDEIKPEIMLFGATHIGR    37RepA_5   
		    179    716.20    1430.38    1429.75    74    EVDAELCAILLGK    37RepA_5   
		    159    683.60    1365.19    1364.80    79    IAPVVIELLGEGR    37RepA_7   
		    21    430.86    859.71    859.44    41    ITQDDIR    37Rep_5   
		    143    666.06    1330.11    1329.70    70    LDSVDDLLEAIK    37RepA_5   
		    169    701.55    1401.08    1400.74    61    LDSVDDLLEAIKA    37RepA_5   
		    70    543.94    1085.87    1085.56    48    LEIDPEDKK    37RepA_5   
		    16    415.81    829.62    830.46    40    LGGVVGSSR    37Rep_4   
		    545    1006.14    3015.41    3015.60    53    NPAAPILEIADYGVVGDLHEIVPMLIEK    37Rep_7   
		    99    581.99    1161.96    1161.59    35    NVWIFAEQR    37Rep_5   
		    81    554.92    1107.82    1107.58    69    TGEVIALDYK    37Rep_7   
		    31    445.93    889.84    889.51    45    TTVLETVK    37RepA_8   
		    92    561.90    1121.79    1121.54    60    VGTGLTADCTK    37Rep_5   
   
      Matching Genes:  
               gi|115249407|emb|CAJ67222.1|  (electron transfer flavoprotein alpha-subunit [Clostridium difficile 630]) 
              Other Genes Matching Peptide Subset:  
               gi|115249822|emb|CAJ67639.1|  (electron transfer flavoprotein alpha-subunit [Clostridium difficile 630]) 
           
  Protein Group 11   
      Expression Quality:  
         Score      Num Spectra      Num Peptides      High-Qual Peptides      % Coverage       705    48    12    10    57   
   
      Peptides:   
        Query    Observed    Mr(expt)    Mr(calc)    Score    Peptide    Result File   
		    171    452.32    902.62    902.53    45    AFLGLLNR    37Rep_1   
		    228    621.39    1240.76    1240.56    54    EGYPEVGEAYK    37Rep_3   
		    225    699.47    1396.92    1396.66    46    EGYPEVGEAYKR    37RepA_1   
		    472    942.64    1883.27    1882.86    28    ELGLDAIHDTVHEMCK +Oxidation (M)    37Rep_3   
		    424    785.91    2354.72    2354.06    25    ELGLDAIHDTVHEMCKDEAR +Oxidation (M)    37RepA_3   
		    350    746.06    1490.11    1489.80    105    FAELLGEVVVADTK    37Rep_3   
		    127    591.41    1180.80    1180.61    66    IALEEAEHAAK    37RepA_2   
		    300    849.66    1697.30    1696.90    82    IGVAQGVDEEIIEGLR    37RepA_3   
		    219    563.34    1124.66    1124.50    67    VDAEYGATDGK    37Rep_2   
		    227    577.29    1152.57    1152.55    62    VGADKFEEMK    37Rep_2   
		    195    585.37    1168.72    1168.54    43    VGADKFEEMK +Oxidation (M)    37Rep_3   
		    307    690.90    1379.78    1379.67    82    VRVDAEYGATDGK    37Rep_3   
   
      Matching Genes:  
               gi|115250515|emb|CAJ68339.1|  (putative ruberythrin [Clostridium difficile 630]) 
           
  Protein Group 12   
      Expression Quality:  
         Score      Num Spectra      Num Peptides      High-Qual Peptides      % Coverage       680    27    12    11    28   
   
      Peptides:   
        Query    Observed    Mr(expt)    Mr(calc)    Score    Peptide    Result File   
		    322    1024.50    2046.98    2046.08    54    AAADEIGLPLFQYLGGVNAK    37RepA_4   
		    58    537.86    1073.71    1073.56    61    AGYTAVISHR    37RepA_6   
		    160    665.09    1328.17    1327.76    74    EALELIVEAITK    37Rep_6   
		    122    657.69    1313.37    1312.73    57    GIENGVANSILVK    37RepA_6   
		    81    581.38    1160.75    1160.55    50    IEEMVGEQAR    37RepA_8   
		    105    589.38    1176.74    1176.54    62    IEEMVGEQAR +Oxidation (M)    37Rep_8   
		    268    860.69    1719.36    1718.88    34    LQLVGDDLFVTNTER    37Rep_6   
		    80    559.91    1117.80    1117.56    55    MGAEVFHSLK    37Rep_6   
		    87    567.91    1133.81    1133.55    55    MGAEVFHSLK +Oxidation (M)    37Rep_6   
		    90    410.98    1229.93    1229.66    43    RAGYTAVISHR    37RepA_6   
		    381    1095.24    2188.46    2188.09    93    SGETEDSTIADLAVAVNAGQIK    37Rep_6   
		    16    418.86    835.70    835.44    42    YVLAGEGK    37Rep_6   
   
      Matching Genes:  
               gi|115252227|emb|CAJ70067.1|  (enolase [Clostridium difficile 630]) 
           
  Protein Group 13   
      Expression Quality:  
         Score      Num Spectra      Num Peptides      High-Qual Peptides      % Coverage       676    31    13    11    39   
   
      Peptides:   
        Query    Observed    Mr(expt)    Mr(calc)    Score    Peptide    Result File   
		    639    1064.31    3189.90    3189.59    78    ALEAANMTIEDIDLVEANEAFAAQSVAVIR +Oxidation (M)    37Rep_2   
		    504    1370.70    2739.39    2738.43    69    ANITPDMIDESLLGGVLTAGLGQNIAR    37RepA_5   
		    302    936.81    1871.61    1871.00    47    AQAEGKFDEEIVPVVIK    37RepA_5   
		    433    1132.30    2262.59    2262.10    51    DGTVTAGNASGINDGAAMLVVMAK    37Rep_5   
		    448    1148.34    2294.67    2294.09    61    DGTVTAGNASGINDGAAMLVVMAK +2 Oxidation (M)    37Rep_5   
		    442    1140.35    2278.69    2278.09    51    DGTVTAGNASGINDGAAMLVVMAK +Oxidation (M)    37Rep_5   
		    193    738.14    1474.27    1473.69    62    EEQDELALASQNK    37RepA_5   
		    54    458.46    914.91    914.52    33    EVVIASAAR    37Rep_5   
		    128    644.54    1287.06    1286.71    35    FDEEIVPVVIK    37RepA_5   
		    99    613.19    1224.37    1223.68    60    ILTTLLYEMK    37RepA_5   
		    115    621.10    1240.18    1239.68    40    ILTTLLYEMK +Oxidation (M)    37RepA_5   
		    94    567.41    1132.81    1132.60    49    IMGYGPVPATK    37Rep_5   
		    117    616.40    1230.79    1230.68    40    SVSAVELGVTAAK    37Rep_5   
   
      Matching Genes:  
               gi|115250080|emb|CAJ67900.1|  (acetyl-CoA acetyltransferase [Clostridium difficile 630]) 
           
  Protein Group 14   
      Expression Quality:  
         Score      Num Spectra      Num Peptides      High-Qual Peptides      % Coverage       662    22    13    11    27   
   
      Peptides:   
        Query    Observed    Mr(expt)    Mr(calc)    Score    Peptide    Result File   
		    508    1350.37    2698.72    2698.26    54    AEVTDVANAIYDGTDAIMLSGETAAGK +Oxidation (M)    37Rep_7   
		    91    573.87    1145.72    1146.55    51    DGEVVTVDASR    37Rep_7   
		    285    928.23    1854.45    1853.94    32    GDLGVEIPTEEMPIVQK    37Rep_7   
		    252    872.00    1741.98    1741.87    68    IENQEGVENLDEILK    37RepA_7   
		    50    484.02    966.03    965.59    47    INLPAITPK    37RepA_7   
		    57    486.89    971.77    971.58    52    KASDVLAIR    37Rep_7   
		    126    649.43    1296.85    1296.59    50    RTEETLDYDR    37RepA_7   
		    211    765.61    1529.21    1528.81    52    SGDSILIDDGLVGLR    37Rep_7   
		    87    594.42    1186.82    1186.62    62    SPIIATTNNEK    37RepA_8   
		    132    675.01    1348.01    1347.65    57    SSVAGNTDEVIEK    37RepA_8   
		    90    571.36    1140.72    1140.49    41    TEETLDYDR    37Rep_7   
		    275    920.10    1838.18    1837.84    60    TGNFEDPEVFLEEGQK    37Rep_7   
		    49    474.39    946.76    946.49    36    VSDGIMVAR    37Rep_7   
   
      Matching Genes:  
               gi|115252454|emb|CAJ70297.1|  (pyruvate kinase [Clostridium difficile 630]) 
           
  Protein Group 15   
      Expression Quality:  
         Score      Num Spectra      Num Peptides      High-Qual Peptides      % Coverage       647    34    12    9    45   
   
      Peptides:   
        Query    Observed    Mr(expt)    Mr(calc)    Score    Peptide    Result File   
		    37    440.89    879.76    879.45    31    AFESIWK    37Rep_6   
		    233    748.04    1494.06    1493.72    60    DVNVFEMAQSQVK    37Rep_5   
		    276    886.24    1770.47    1769.92    42    GGIIVDPSTLSQGELER    37Rep_6   
		    341    1011.80    2021.59    2021.03    68    IAVQGIGNVGSYTVLNCEK    37RepA_5   
		    139    428.37    1282.08    1281.64    28    IKEEYNVTMR    37Rep_5   
		    447    921.18    2760.50    2759.43    28    ISLEEFWASDVDIVIPAALENSITK    37RepA_2   
		    173    682.06    1362.10    1361.73    46    LGMEPAVYELLK    37Rep_6   
		    367    1130.96    2259.90    2259.22    69    LTGQSSIGVITGKPVEFGGSLGR    37RepA_6   
		    379    1138.88    2275.74    2275.04    64    LVCEAANGPTTPEADEVFAER    37RepA_6   
		    469    1285.63    2569.25    2568.11    69    SEGSYAIYNENGLDGQAMLDYMK    37RepA_5   
		    85    611.50    1220.98    1220.65    100    TAATGFGVAVTAR    37RepA_6   
		    64    492.47    982.92    982.61    42    VIEVSIPVK    37Rep_6   
   
      Matching Genes:  
               gi|115249189|emb|CAJ67001.1|  (NAD-specific glutamate dehydrogenase [Clostridium difficile 630]) 
           
  Protein Group 16   
      Expression Quality:  
         Score      Num Spectra      Num Peptides      High-Qual Peptides      % Coverage       642    19    12    11    21   
   
      Peptides:   
        Query    Observed    Mr(expt)    Mr(calc)    Score    Peptide    Result File   
		    190    734.02    1466.03    1465.69    27    AGAPFAPGANPMHGR +Oxidation (M)    37Rep_7   
		    346    1033.74    2065.47    2064.92    55    FEPITSEYLDYDEVMSK    37Rep_7   
		    347    1041.84    2081.66    2080.92    53    FEPITSEYLDYDEVMSK +Oxidation (M)    37Rep_7   
		    433    810.29    2427.86    2427.24    46    LPYEHAQDGISNTFSIVPAALGK    37RepA_1   
		    277    923.70    1845.39    1844.86    74    MAESYGFDISKPATNSK    37Rep_7   
		    290    931.74    1861.47    1860.86    55    MAESYGFDISKPATNSK +Oxidation (M)    37Rep_7   
		    370    1074.38    2146.74    2145.92    46    MVENSCEAFGYELDPEIK +Oxidation (M)    37Rep_7   
		    329    1043.08    2084.15    2084.08    42    NSYPTQSILTITSNVVYGK    37RepA_7   
		    139    660.55    1319.09    1318.69    79    SGIITGLPDAYGR    37Rep_7   
		    308    953.36    1904.71    1903.99    49    VALYGVDALIEDKNEQK    37Rep_7   
		    326    986.69    1971.36    1970.89    65    VSIDTSSVQYENDDLMR    37Rep_7   
		    307    994.70    1987.38    1986.88    51    VSIDTSSVQYENDDLMR +Oxidation (M)    37RepA_7   
   
      Matching Genes:  
               gi|115249776|emb|CAJ67593.1|  (formate acetyltransferase [Clostridium difficile 630]) 
           
  Protein Group 17   
      Expression Quality:  
         Score      Num Spectra      Num Peptides      High-Qual Peptides      % Coverage       632    37    11    10    31   
   
      Peptides:   
        Query    Observed    Mr(expt)    Mr(calc)    Score    Peptide    Result File   
		    131    652.54    1303.06    1302.71    51    ALQLHGGYGFIK    37Rep_7   
		    112    631.34    1260.66    1260.59    44    ELDTLPAEMDK    37RepA_7   
		    67    482.89    963.77    963.50    31    GLVYDAAQK    37Rep_5   
		    61    519.94    1037.87    1037.54    43    GTSAFIVESK    37RepA_5   
		    64    523.91    1045.80    1045.56    63    IAMGTLEVGR    37RepA_5   
		    83    531.89    1061.76    1061.55    70    IAMGTLEVGR +Oxidation (M)    37Rep_5   
		    300    627.51    1879.50    1879.08    80    IGVAALALGIAQGALDEAVK    37Rep_7   
		    213    727.01    1452.00    1451.75    76    IVSIYEGTSEVQK    37Rep_8   
		    53    494.42    986.82    986.59    49    VQFGKPIAK    37RepA_5   
		    151    675.46    1348.90    1348.57    54    WDGFSTGAHEDK    37RepA_5   
		    208    761.07    1520.13    1519.72    71    YYASEIANEVAYK    37Rep_7   
   
      Matching Genes:  
               gi|115249405|emb|CAJ67220.1|  (acyl-CoA dehydrogenase, short-chain specific [Clostridium difficile 630]) 
           
  Protein Group 18   
      Expression Quality:  
         Score      Num Spectra      Num Peptides      High-Qual Peptides      % Coverage       586    20    11    10    32   
   
      Peptides:   
        Query    Observed    Mr(expt)    Mr(calc)    Score    Peptide    Result File   
		    428    857.97    2570.88    2570.27    86    DILDILEDNNISVVADDLAQETR    37RepA_4   
		    86    585.51    1169.00    1168.61    49    EVVENPNAAVK    37RepA_5   
		    252    852.10    1702.19    1701.73    43    FCDPEEYDYPLVR    37RepA_5   
		    216    763.57    1525.13    1524.73    73    IHESIEVYNEHR    37RepA_5   
		    175    675.92    1349.83    1349.60    33    LNAMPEEVCSGK +Oxidation (M)    37Rep_5   
		    199    493.69    1478.05    1477.69    47    LNAMPEEVCSGKK +Oxidation (M)    37RepA_5   
		    210    722.99    1443.96    1443.74    69    MKEVVENPNAAVK +Oxidation (M)    37Rep_5   
		    269    884.67    1767.32    1766.79    49    QWSNIEGCSLAYDPK    37RepA_5   
		    119    629.92    1257.82    1257.58    41    TDVPAGDDALER    37RepA_5   
		    80    565.50    1128.98    1128.68    41    VLLTGILADSK    37RepA_5   
		    115    613.97    1225.92    1225.66    55    YISLVHPQNR    37Rep_5   
   
      Matching Genes:  
               gi|115249404|emb|CAJ67219.1|  (subunit of oxygen-sensitive 2-hydroxyisocaproyl-CoA dehydratase [Clostridium difficile 630]) 
           
  Protein Group 19   
      Expression Quality:  
         Score      Num Spectra      Num Peptides      High-Qual Peptides      % Coverage       571    26    10    9    44   
   
      Peptides:   
        Query    Observed    Mr(expt)    Mr(calc)    Score    Peptide    Result File   
		    144    665.00    1327.99    1327.73    58    ALENVLKDDLAK    37RepA_4   
		    126    596.46    1190.90    1190.62    38    EALEFVNEIK    37Rep_4   
		    293    840.11    1678.20    1677.77    67    EIDMDYVVIGHSER +Oxidation (M)    37Rep_4   
		    88    610.96    1219.91    1219.62    42    GLYGELANEVR    37RepA_4   
		    129    652.44    1302.87    1302.60    68    IGAQNMHFEEK    37RepA_4   
		    193    660.41    1318.80    1318.60    47    IGAQNMHFEEK +Oxidation (M)    37Rep_4   
		    53    513.93    1025.85    1025.60    44    KPIIAGNWK    37RepA_4   
		    272    808.58    1615.14    1614.75    64    QYFNETDETVNKK    37Rep_4   
		    281    820.05    1638.08    1637.79    70    TATAEDANDVISYIR    37Rep_4   
		    279    802.21    1602.40    1601.88    73    VVVAYEPIWAIGTGK    37RepA_3   
   
      Matching Genes:  
               gi|115252229|emb|CAJ70069.1|  (triosephosphate isomerase [Clostridium difficile 630]) 
           
  Protein Group 20   
      Expression Quality:  
         Score      Num Spectra      Num Peptides      High-Qual Peptides      % Coverage       486    24    7    6    37   
   
      Peptides:   
        Query    Observed    Mr(expt)    Mr(calc)    Score    Peptide    Result File   
		    360    969.42    1936.83    1937.95    77    AFGGADTWATSNTIAAGISK    37RepA_3   
		    332    694.54    2080.59    2080.03    74    DGVPSILNPDDANALEEALK    37RepA_4   
		    312    871.32    1740.62    1739.87    106    QAIDGDTAQVGPQIAEK    37RepA_3   
		    133    604.47    1206.92    1206.61    67    QLEDGYELIK    37RepA_3   
		    57    529.40    1056.78    1056.52    38    QVPDTNEVR    37RepA_4   
		    217    613.41    1224.81    1224.61    61    VGDYDIIFAGR    37Rep_3   
		    114    564.46    1126.91    1126.70    63    VSTPVLLTAVK    37RepA_3   
   
      Matching Genes:  
               gi|115249406|emb|CAJ67221.1|  (electron transfer flavoprotein beta-subunit [Clostridium difficile 630]) 
           
  Protein Group 21   
      Expression Quality:  
         Score      Num Spectra      Num Peptides      High-Qual Peptides      % Coverage       483    11    9    8    35   
   
      Peptides:   
        Query    Observed    Mr(expt)    Mr(calc)    Score    Peptide    Result File   
		    253    794.24    1586.46    1585.88    45    AADPIVVLFGATSIGR    37Rep_5   
		    71    545.57    1089.13    1088.55    56    ATIDAGWLDK    37RepA_5   
		    438    759.94    2276.79    2276.15    62    ENLDILYELAEIIGGEVSGSR    37Rep_5   
		    217    772.27    1542.53    1541.87    47    ENVIQTVSLELLGK    37RepA_5   
		    357    987.24    1972.46    1971.96    89    IHTGLTADCTGLAVAEDTK    37Rep_5   
		    77    513.50    1024.99    1024.66    52    LVQVVQVIK    37Rep_5   
		    53    458.37    914.72    914.49    37    NPEAPIFK    37Rep_5   
		    166    699.17    1396.32    1395.83    48    VLPELISQLSVAK    37RepA_5   
		    25    444.44    886.86    886.55    47    VSALLLGSK    37RepA_5   
   
      Matching Genes:  
               gi|115250077|emb|CAJ67897.1|  (electron transfer flavoprotein alpha-subunit [Clostridium difficile 630]) 
           
  Protein Group 22   
      Expression Quality:  
         Score      Num Spectra      Num Peptides      High-Qual Peptides      % Coverage       467    19    8    6    34   
   
      Peptides:   
        Query    Observed    Mr(expt)    Mr(calc)    Score    Peptide    Result File   
		    450    905.24    1808.47    1807.91    90    AFAGADTWATSSALAGALK    37Rep_3   
		    17    415.84    829.66    829.45    70    AGLEEAIK    37Rep_4   
		    418    1041.38    2080.75    2080.07    33    DGVPSIINPDDKAGLEEAIK    37Rep_4   
		    127    650.47    1298.93    1298.67    58    DIEVDPSNLGLK    37RepA_4   
		    62    538.93    1075.85    1075.58    51    MPCLITTLK    37RepA_4   
		    51    508.88    1015.74    1015.52    32    QVPDTTEVK    37RepA_4   
		    249    747.02    1492.03    1491.76    60    SVKPAGTIYNEDAK    37RepA_3   
		    65    459.37    916.73    916.52    73    TSAGIIIDK    37Rep_4   
   
      Matching Genes:  
               gi|115250076|emb|CAJ67896.1|  (electron transfer flavoprotein beta-subunit [Clostridium difficile 630]) 
           
  Protein Group 23   
      Expression Quality:  
         Score      Num Spectra      Num Peptides      High-Qual Peptides      % Coverage       460    15    7    7    17   
   
      Peptides:   
        Query    Observed    Mr(expt)    Mr(calc)    Score    Peptide    Result File   
		    109    641.63    1281.24    1280.66    69    ESTIEFLTSVR    37RepA_6   
		    97    592.42    1182.83    1182.63    59    GISDFLLSFGK    37Rep_6   
		    387    1151.78    2301.55    2301.01    77    HEFTTSDPGMTYSVAETSVDK    37RepA_6   
		    445    773.59    2317.73    2317.01    62    HEFTTSDPGMTYSVAETSVDK +Oxidation (M)    37Rep_6   
		    160    728.04    1454.07    1453.76    45    NVEHDFLKDPIK    37RepA_6   
		    147    648.08    1294.14    1293.66    61    TCVVSLPVEYK    37Rep_6   
		    286    990.34    1978.67    1978.03    87    VLSVLNVDYELASVDGGTK    37RepA_6   
   
      Matching Genes:  
               gi|115249724|emb|CAJ67541.1|  (putative aminoacyl-histidine dipeptidase [Clostridium difficile 630]) 
           
  Protein Group 24   
      Expression Quality:  
         Score      Num Spectra      Num Peptides      High-Qual Peptides      % Coverage       442    12    9    6    20   
   
      Peptides:   
        Query    Observed    Mr(expt)    Mr(calc)    Score    Peptide    Result File   
		    54    529.99    1057.97    1057.60    56    AVTVAVEELK    37RepA_6   
		    49    468.37    934.73    934.51    39    GTFDVVAVK    37Rep_6   
		    44    501.87    1001.73    1001.51    64    IEDALNATR    37RepA_6   
		    367    1075.41    2148.81    2148.22    48    ISNIQELLPVLEQIVQQGK    37Rep_6   
		    2    407.36    812.71    812.51    41    LAGGVAVVK    37RepA_6   
		    73    587.54    1173.07    1172.65    56    LIAEAMEIVGK    37RepA_6   
		    367    709.12    2124.34    2125.19    29    LLIIAEDVEGEALSTLVVNK    37RepA_2   
		    96    620.06    1238.11    1237.71    38    NVTAGANPILLR    37RepA_6   
		    72    508.86    1015.72    1015.55    71    VGAATEVELK    37Rep_6   
   
      Matching Genes:  
               gi|115249204|emb|CAJ67016.1|  (60 kDa chaperonin [Clostridium difficile 630]) 
           
  Protein Group 25   
      Expression Quality:  
         Score      Num Spectra      Num Peptides      High-Qual Peptides      % Coverage       434    15    8    8    41   
   
      Peptides:   
        Query    Observed    Mr(expt)    Mr(calc)    Score    Peptide    Result File   
		    136    501.34    1000.66    1000.56    48    GTQAVGIVEK    37Rep_3   
		    96    518.45    1034.89    1034.65    51    IILLGPPGAGK    37RepA_3   
		    510    1037.80    2073.59    2073.07    52    IQVYLDETKPLVDYYSK    37Rep_3   
		    6    409.31    816.60    816.43    48    NAGISLDK    37Rep_3   
		    42    429.40    856.79    856.50    47    QGIIADIK    37RepA_3   
		    452    906.57    1811.12    1810.80    83    VEGVCDVCQGELYQR    37Rep_3   
		    93    458.34    914.67    914.51    60    VVNIEVDK    37Rep_3   
		    363    511.67    1531.98    1531.78    45    YNIPHISTGDIFR    37Rep_3   
   
      Matching Genes:  
               gi|115249098|emb|CAJ66909.1|  (adenylate kinase [Clostridium difficile 630]) 
           
  Protein Group 26   
      Expression Quality:  
         Score      Num Spectra      Num Peptides      High-Qual Peptides      % Coverage       413    11    8    4    20   
   
      Peptides:   
        Query    Observed    Mr(expt)    Mr(calc)    Score    Peptide    Result File   
		    439    1220.98    2439.94    2439.29    28    DSPLVVGVGEGENFIASDIPALLK    37RepA_7   
		    62    562.42    1122.82    1122.58    59    EHGNELVAVR    37RepA_6   
		    93    583.37    1164.72    1164.59    35    EIHEQPTGVR    37Rep_7   
		    356    1086.95    2171.90    2171.01    36    ESDDVFYTWAGPEVAVASTK    37RepA_7   
		    221    749.57    1497.13    1496.67    98    GYDSAGVAVNSSNEK    37Rep_6   
		    76    547.84    1093.66    1093.49    34    MVSNMEEVR    37RepA_7   
		    189    733.52    1465.04    1464.74    63    TVVSSEHAFYLGR    37Rep_7   
		    399    753.88    2258.62    2258.03    60    WATHGEPSDVNSHPHFNQAK    37Rep_7   
   
      Matching Genes:  
               gi|115249129|emb|CAJ66940.1|  (glucosamine--fructose-6-phosphate aminotransferase [isomerizing] [Clostridium difficile 630]) 
           
  Protein Group 27   
      Expression Quality:  
         Score      Num Spectra      Num Peptides      High-Qual Peptides      % Coverage       378    15    6    3    26   
   
      Peptides:   
        Query    Observed    Mr(expt)    Mr(calc)    Score    Peptide    Result File   
		    409    558.44    1672.29    1671.87    33    IADELTQLKDEIER    37Rep_3   
		    226    839.70    1677.39    1676.84    36    IRDTDVASEMVNLSK    37RepA_4   
		    235    847.67    1693.33    1692.84    38    IRDTDVASEMVNLSK +Oxidation (M)    37RepA_4   
		    103    535.38    1068.74    1068.51    44    ISSSTEFNGK    37RepA_3   
		    491    1159.81    2317.61    2317.11    139    LESTQNNLNNTLENVTAAESR    37Rep_4   
		    453    910.15    1818.28    1817.88    88    TLSLQSANEINNTEER    37Rep_3   
   
      Matching Genes:  
               gi|115249247|emb|CAJ67060.1|  (flagellin subunit [Clostridium difficile 630]) 
           
  Protein Group 28   
      Expression Quality:  
         Score      Num Spectra      Num Peptides      High-Qual Peptides      % Coverage       377    11    6    5    30   
   
      Peptides:   
        Query    Observed    Mr(expt)    Mr(calc)    Score    Peptide    Result File   
		    237    710.96    1419.90    1419.67    74    EIMDAANNTGASVK    37RepA_2   
		    399    718.93    1435.84    1435.66    77    EIMDAANNTGASVK +Oxidation (M)    37Rep_2   
		    110    558.97    1115.93    1115.60    60    LINNLMVDGK    37RepA_2   
		    223    566.90    1131.78    1131.60    48    LINNLMVDGK +Oxidation (M)    37Rep_2   
		    385    704.46    1406.91    1406.69    29    REVLPDPMYGSK +Oxidation (M)    37Rep_2   
		    281    618.90    1235.78    1235.60    89    TGEEALEVFNK    37Rep_2   
   
      Matching Genes:  
               gi|115249073|emb|CAJ66884.1|  (30S ribosomal protein S7 [Clostridium difficile 630]) 
           
  Protein Group 29   
      Expression Quality:  
         Score      Num Spectra      Num Peptides      High-Qual Peptides      % Coverage       377    11    8    6    17   
   
      Peptides:   
        Query    Observed    Mr(expt)    Mr(calc)    Score    Peptide    Result File   
		    236    825.44    1648.87    1648.67    51    FAGMDLGMNFEEEK +2 Oxidation (M)    37RepA_7   
		    249    862.49    1722.96    1722.85    66    FIDNGIGMTEEEIKK    37RepA_7   
		    98    594.94    1187.87    1187.64    45    LVSLGEISENK    37Rep_7   
		    143    671.08    1340.14    1339.77    39    TLVINENSPIIK    37RepA_7   
		    247    826.72    1651.43    1650.90    41    VIEPLNDTNPLWLK    37Rep_7   
		    317    965.24    1928.47    1927.84    61    WISEGGTEYEISESDAR    37Rep_7   
		    186    486.18    1455.51    1454.76    32    WLYSDKDIFIR    37RepA_7   
		    268    925.77    1849.53    1848.87    42    YINQVAFSGAEDFFNK    37RepA_7   
   
      Matching Genes:  
               gi|115249282|emb|CAJ67095.1|  (chaperone protein (heat shock protein) [Clostridium difficile 630]) 
           
  Protein Group 30   
      Expression Quality:  
         Score      Num Spectra      Num Peptides      High-Qual Peptides      % Coverage       360    18    7    6    27   
   
      Peptides:   
        Query    Observed    Mr(expt)    Mr(calc)    Score    Peptide    Result File   
		    18    416.84    831.67    831.45    53    CLALLDK    37Rep_4   
		    327    1038.03    2074.04    2073.12    33    ILIPMINEAVGIYADGVASK    37RepA_4   
		    164    637.44    1272.87    1272.73    48    LVEVISGQLTSK    37Rep_4   
		    405    681.87    2042.58    2042.08    69    SINKVPVDVSESPGFVVNR    37Rep_4   
		    202    801.15    1600.29    1599.83    52    VPVDVSESPGFVVNR    37RepA_4   
		    122    643.51    1285.01    1284.66    64    VTFDTVFELSK    37RepA_4   
		    102    547.90    1093.78    1093.64    41    YRPHPLLAK    37Rep_4   
   
      Matching Genes:  
               gi|115250079|emb|CAJ67899.1|  (3-hydroxybutyryl-CoA dehydrogenase [Clostridium difficile 630]) 
           
  Protein Group 31   
      Expression Quality:  
         Score      Num Spectra      Num Peptides      High-Qual Peptides      % Coverage       349    10    6    5    24   
   
      Peptides:   
        Query    Observed    Mr(expt)    Mr(calc)    Score    Peptide    Result File   
		    497    1179.39    2356.76    2356.16    56    GLLEEDLTEMNLSSVGDIIHR +Oxidation (M)    37Rep_4   
		    344    913.24    1824.47    1823.96    82    HSIIVLAEGVGSASDLEK    37Rep_4   
		    340    908.26    1814.50    1813.94    74    TIGLLTSGGDAPGMNAAIR    37Rep_4   
		    276    916.23    1830.45    1829.93    36    TIGLLTSGGDAPGMNAAIR +Oxidation (M)    37RepA_4   
		    207    673.93    1345.84    1345.62    52    VFDKEAYEMAK +Oxidation (M)    37Rep_4   
		    48    504.86    1007.71    1007.59    49    VTVLGHVQR    37RepA_4   
   
      Matching Genes:  
               gi|115252455|emb|CAJ70298.1|  (6-phosphofructokinase [Clostridium difficile 630]) 
           
  Protein Group 32   
      Expression Quality:  
         Score      Num Spectra      Num Peptides      High-Qual Peptides      % Coverage       343    9    7    5    23   
   
      Peptides:   
        Query    Observed    Mr(expt)    Mr(calc)    Score    Peptide    Result File   
		    324    1069.51    2137.01    2136.05    56    EGVFAGGDAVTGAATVISAMGAGK    37RepA_6   
		    98    593.92    1185.83    1185.60    53    LGSESYIVYR    37Rep_6   
		    177    460.05    1377.13    1376.60    31    MELGEPDDSGRR +Oxidation (M)    37Rep_6   
		    76    591.47    1180.93    1180.60    26    TAAASIDEYLK    37RepA_6   
		    228    864.26    1726.50    1725.91    70    VAVIGSGPAGLACAGDLAK    37RepA_6   
		    185    702.09    1402.16    1401.70    63    VAVVGGGNVAMDAAR +Oxidation (M)    37Rep_6   
		    285    989.26    1976.50    1975.92    44    VCPQESQCEGVCILGIK    37RepA_6   
   
      Matching Genes:  
               gi|115250578|emb|CAJ68402.1|  (putative glutamate synthase [NADPH] small chain [Clostridium difficile 630]) 
           
  Protein Group 33   
      Expression Quality:  
         Score      Num Spectra      Num Peptides      High-Qual Peptides      % Coverage       343    12    8    6    39   
   
      Peptides:   
        Query    Observed    Mr(expt)    Mr(calc)    Score    Peptide    Result File   
		    124    580.49    1158.97    1158.68    47    ALVFENVLVR    37RepA_3   
		    21    415.83    829.66    829.47    44    EGVIVASR    37Rep_3   
		    178    565.86    1129.70    1129.56    57    ESGDIAGTPGVK    37Rep_3   
		    444    891.60    1781.18    1780.85    36    FKPLSQPGQYACEEK    37Rep_3   
		    254    428.18    1281.51    1281.58    29    HIHMSNEDATK    37Rep_3   
		    47    428.34    854.66    854.52    42    LPIALSNK    37Rep_3   
		    177    565.38    1128.74    1128.58    42    LVGPAGEVEMK    37Rep_3   
		    183    573.38    1144.75    1144.58    46    LVGPAGEVEMK +Oxidation (M)    37Rep_3   
   
      Matching Genes:  
               gi|115251734|emb|CAJ69569.1|  (putative propanediol utilization protein [Clostridium difficile 630]) 
           
  Protein Group 34   
      Expression Quality:  
         Score      Num Spectra      Num Peptides      High-Qual Peptides      % Coverage       328    10    6    6    18   
   
      Peptides:   
        Query    Observed    Mr(expt)    Mr(calc)    Score    Peptide    Result File   
		    314    962.31    1922.60    1921.92    69    AEGIEATEEEFKAELEK    37Rep_6   
		    378    1092.33    2182.65    2182.05    53    ELDIDPIDNPDLDIEEISK    37Rep_6   
		    503    1309.38    2616.74    2616.20    42    ELSALDDEFAKDTSEFDSLDELK    37Rep_6   
		    151    652.90    1303.79    1303.54    49    MASAYNMEVEK +2 Oxidation (M)    37Rep_6   
		    144    644.93    1287.84    1287.55    52    MASAYNMEVEK +Oxidation (M)    37Rep_6   
		    77    531.06    1060.10    1059.62    63    SSLVLEAITK    37Rep_6   
   
      Matching Genes:  
               gi|115252362|emb|CAJ70203.1|  (trigger factor [Clostridium difficile 630]) 
           
  Protein Group 35   
      Expression Quality:  
         Score      Num Spectra      Num Peptides      High-Qual Peptides      % Coverage       323    9    6    5    15   
   
      Peptides:   
        Query    Observed    Mr(expt)    Mr(calc)    Score    Peptide    Result File   
		    167    676.55    1351.08    1350.79    64    IVEVPVGEALIGR    37Rep_6   
		    177    501.46    1501.37    1500.82    29    MNLKPEEISSIIK    37RepA_6   
		    231    770.56    1539.11    1538.81    54    TRPVESEAPGIIDR    37Rep_6   
		    220    566.12    1695.34    1694.91    50    TRPVESEAPGIIDRR    37RepA_6   
		    470    1212.43    2422.85    2422.25    67    VELTDTGSVLTVGDGIASVYGLEK    37Rep_6   
		    112    613.97    1225.92    1225.67    59    VVNSLGQPIDGK    37Rep_6   
   
      Matching Genes:  
               gi|115252530|emb|CAJ70373.1|  (ATP synthase alpha chain [Clostridium difficile 630]) 
           
  Protein Group 36   
      Expression Quality:  
         Score      Num Spectra      Num Peptides      High-Qual Peptides      % Coverage       304    9    6    3    40   
   
      Peptides:   
        Query    Observed    Mr(expt)    Mr(calc)    Score    Peptide    Result File   
		    225    573.88    1145.74    1145.58    54    ETEGEIEVLK    37Rep_2   
		    359    1038.75    2075.49    2075.03    39    EYLPQQLSEEELEEIVK    37RepA_2   
		    71    458.42    914.83    914.59    51    KSVVTLIR    37RepA_2   
		    138    605.93    1209.85    1209.59    36    STISEVGATSMK    37RepA_2   
		    146    613.91    1225.80    1225.59    33    STISEVGATSMK +Oxidation (M)    37RepA_2   
		    429    764.98    1527.95    1527.80    91    VELDEDGIIDVIAK    37Rep_2   
   
      Matching Genes:  
               gi|115251499|emb|CAJ69332.1|  (putative tRNA binding protein [Clostridium difficile 630]) 
           
  Protein Group 37   
      Expression Quality:  
         Score      Num Spectra      Num Peptides      High-Qual Peptides      % Coverage       298    8    5    5    31   
   
      Peptides:   
        Query    Observed    Mr(expt)    Mr(calc)    Score    Peptide    Result File   
		    365    978.22    1954.43    1953.98    81    AIANSDLGLNPSNDGEVIR    37RepA_3   
		    61    435.28    868.54    868.44    42    FEFGTIR    37Rep_3   
		    164    545.88    1089.74    1089.52    48    GGELTEDELK    37Rep_3   
		    136    609.90    1217.79    1217.61    56    GGELTEDELKK    37RepA_3   
		    566    1194.36    2386.70    2386.22    71    VDYYGTPTPINQIGAISVPEPR    37Rep_3   
   
      Matching Genes:  
               gi|115251191|emb|CAJ69022.1|  (ribosome recycling factor [Clostridium difficile 630]) 
           
  Protein Group 38   
      Expression Quality:  
         Score      Num Spectra      Num Peptides      High-Qual Peptides      % Coverage       295    10    5    5    10   
   
      Peptides:   
        Query    Observed    Mr(expt)    Mr(calc)    Score    Peptide    Result File   
		    48    466.35    930.69    930.50    57    LEDAVAVSK    37Rep_6   
		    113    648.07    1294.12    1293.68    58    MVLGFLTDIGGR +Oxidation (M)    37RepA_6   
		    100    594.95    1187.89    1187.60    56    SIDNVEAEIAK    37Rep_6   
		    236    791.09    1580.16    1579.77    75    TESVNAEYALNEIK    37Rep_6   
		    187    706.97    1411.93    1411.55    49    WAGMCGESAGDQK +Oxidation (M)    37Rep_6   
   
      Matching Genes:  
               gi|115251808|emb|CAJ69643.1|  (phosphoenolpyruvate-protein phosphotransferase [Clostridium difficile 630]) 
           
  Protein Group 39   
      Expression Quality:  
         Score      Num Spectra      Num Peptides      High-Qual Peptides      % Coverage       294    9    6    5    13   
   
      Peptides:   
        Query    Observed    Mr(expt)    Mr(calc)    Score    Peptide    Result File   
		    349    1040.71    2079.41    2078.92    45    AAEETGLPYAGFDGDQADPR    37RepA_5   
		    42    468.39    934.76    934.48    48    AQFEEAIK    37RepA_5   
		    149    674.01    1346.00    1345.68    57    LLIEELEDNMK    37RepA_5   
		    180    682.05    1362.08    1361.67    50    LLIEELEDNMK +Oxidation (M)    37Rep_5   
		    404    1118.89    2235.76    2235.02    38    RAAEETGLPYAGFDGDQADPR    37RepA_5   
		    294    603.50    1807.48    1806.95    56    VVINDLLAEQYANAFK    37Rep_5   
   
      Matching Genes:  
               gi|115249403|emb|CAJ67218.1|  (subunit of oxygen-sensitive 2-hydroxyisocaproyl-CoA dehydratase [Clostridium difficile 630]) 
           
  Protein Group 40   
      Expression Quality:  
         Score      Num Spectra      Num Peptides      High-Qual Peptides      % Coverage       284    7    7    4    17   
   
      Peptides:   
        Query    Observed    Mr(expt)    Mr(calc)    Score    Peptide    Result File   
		    84    532.95    1063.89    1063.52    45    AGMMGIPYPK    37Rep_5   
		    188    733.61    1465.20    1464.73    25    DLGKPYGVEAAMAK +Oxidation (M)    37RepA_5   
		    47    482.43    962.85    962.54    27    GISAFIVEK    37RepA_5   
		    98    578.93    1155.84    1155.64    56    HLVYQAAINK    37Rep_5   
		    75    510.93    1019.84    1019.51    48    IAMSTLDGGR    37Rep_5   
		    78    518.93    1035.84    1035.50    32    IAMSTLDGGR +Oxidation (M)    37Rep_5   
		    214    763.03    1524.04    1523.75    51    ITEIYEGTSEVQR    37RepA_5   
   
      Matching Genes:  
               gi|115250075|emb|CAJ67895.1|  (butyryl-CoA dehydrogenase [Clostridium difficile 630]) 
           
  Protein Group 41   
      Expression Quality:  
         Score      Num Spectra      Num Peptides      High-Qual Peptides      % Coverage       272    9    6    4    15   
   
      Peptides:   
        Query    Observed    Mr(expt)    Mr(calc)    Score    Peptide    Result File   
		    86    565.46    1128.91    1128.62    27    EPSLGPVFGVK    37Rep_6   
		    91    581.94    1161.87    1161.59    50    FPLDTEAELK    37Rep_6   
		    468    1206.31    2410.61    2410.15    48    IYGADGVDYTPEADKEIANLEK    37Rep_6   
		    278    969.84    1937.66    1936.99    51    LGNIIVGYSYEGEPVTAR    37RepA_6   
		    261    856.72    1711.42    1711.87    70    SDIEIAQEAKPQDIR    37Rep_6   
		    22    432.87    863.72    863.48    26    VDYNLLK    37RepA_6   
   
      Matching Genes:  
               gi|115249735|emb|CAJ67552.1|  (formate--tetrahydrofolate ligase [Clostridium difficile 630]) 
           
  Protein Group 42   
      Expression Quality:  
         Score      Num Spectra      Num Peptides      High-Qual Peptides      % Coverage       270    10    4    4    8   
   
      Peptides:   
        Query    Observed    Mr(expt)    Mr(calc)    Score    Peptide    Result File   
		    217    744.08    1486.15    1485.80    77    IITNQADAEAIVTK    37Rep_6   
		    172    705.08    1408.14    1407.72    40    SFLGTSDVDIIGGK    37RepA_5   
		    217    737.94    1473.86    1473.63    84    SGGSEDTGYVVEMK +Oxidation (M)    37Rep_5   
		    232    774.02    1546.03    1545.69    69    YYNSDDKNAITDK    37Rep_6   
   
      Matching Genes:  
               gi|115251846|emb|CAJ69681.1|  (cell surface protein (S-layer precursor protein) [Clostridium difficile 630]) 
           
  Protein Group 43   
      Expression Quality:  
         Score      Num Spectra      Num Peptides      High-Qual Peptides      % Coverage       259    6    5    4    21   
   
      Peptides:   
        Query    Observed    Mr(expt)    Mr(calc)    Score    Peptide    Result File   
		    426    864.60    1727.19    1726.88    51    EGITSVGENKPQELAR    37Rep_3   
		    464    934.21    1866.41    1865.85    65    GLMTMAPFIEDEDEIR    37Rep_3   
		    471    942.13    1882.25    1881.85    60    GLMTMAPFIEDEDEIR +Oxidation (M)    37Rep_3   
		    167    552.41    1102.80    1102.57    31    IALCEEIQK    37Rep_3   
		    83    483.35    964.69    964.50    52    VGTSIFGER    37RepA_3   
   
      Matching Genes:  
               gi|115251674|emb|CAJ69509.1|  (putative alanine racemase [Clostridium difficile 630]) 
           
  Protein Group 44   
      Expression Quality:  
         Score      Num Spectra      Num Peptides      High-Qual Peptides      % Coverage       255    9    5    3    17   
   
      Peptides:   
        Query    Observed    Mr(expt)    Mr(calc)    Score    Peptide    Result File   
		    91    560.92    1119.83    1119.58    56    FEGETLPSLK    37Rep_5   
		    114    620.97    1239.92    1239.54    34    LNYSNMPEEK +Oxidation (M)    37RepA_5   
		    261    826.25    1650.48    1649.82    49    MIYVIPDFQNPTGR    37Rep_5   
		    95    567.92    1133.83    1133.55    32    MQGLQGSEIR +Oxidation (M)    37Rep_5   
		    445    1204.44    2406.86    2406.20    84    TNVNKDDILVTSGSQQGLDFAGK    37RepA_5   
   
      Matching Genes:  
               gi|115252729|emb|CAJ70573.1|  (putative amino acid aminotransferase [Clostridium difficile 630]) 
           
  Protein Group 45   
      Expression Quality:  
         Score      Num Spectra      Num Peptides      High-Qual Peptides      % Coverage       254    6    4    3    11   
   
      Peptides:   
        Query    Observed    Mr(expt)    Mr(calc)    Score    Peptide    Result File   
		    282    903.23    1804.44    1803.94    81    AGFVVSDSNIKPDNTLK    37Rep_6   
		    165    672.58    1343.15    1342.72    61    GQATSIIEVAQAR    37Rep_6   
		    41    443.84    885.66    885.47    86    VGAGNVVDR    37Rep_6   
		    287    918.70    1835.39    1834.85    26    VSEFMTPMSSIVYANK +2 Oxidation (M)    37Rep_6   
   
      Matching Genes:  
               gi|115251390|emb|CAJ69222.1|  (inosine-5'-monophosphate dehydrogenase [Clostridium difficile 630]) 
           
  Protein Group 46   
      Expression Quality:  
         Score      Num Spectra      Num Peptides      High-Qual Peptides      % Coverage       252    6    4    4    4   
   
      Peptides:   
        Query    Observed    Mr(expt)    Mr(calc)    Score    Peptide    Result File   
		    138    636.94    1271.86    1271.60    57    AQANEPLTEDGK    37Rep_8   
		    183    686.97    1371.93    1371.72    87    IGAEVDSGDILVGK    37Rep_8   
		    53    514.46    1026.90    1026.58    42    LSALGPGGLSR    37RepA_8   
		    211    723.47    1444.93    1444.70    66    VLTDEDQEIEVR    37Rep_8   
   
      Matching Genes:  
               gi|115249070|emb|CAJ66881.1|  (DNA-directed RNA polymerase beta chain [Clostridium difficile 630]) 
           
  Protein Group 47   
      Expression Quality:  
         Score      Num Spectra      Num Peptides      High-Qual Peptides      % Coverage       252    7    4    4    31   
   
      Peptides:   
        Query    Observed    Mr(expt)    Mr(calc)    Score    Peptide    Result File   
		    189    510.36    1018.71    1018.57    53    EIISSITTR    37Rep_2   
		    365    680.37    1358.73    1358.69    79    IECQGEGLVNLK    37Rep_2   
		    406    734.48    1466.95    1466.87    74    LVNDVELVNVLIK    37Rep_2   
		    198    664.98    1327.95    1327.70    46    NVPVNIWPYAR    37RepA_2   
   
      Matching Genes:  
               gi|115249997|emb|CAJ67817.1|  (hypothetical phage protein [Clostridium difficile 630]) 
              Other Genes Matching Peptide Subset:  
               gi|115251944|emb|CAJ69780.1|  (hypothetical phage protein [Clostridium difficile 630]) 
           
  Protein Group 48   
      Expression Quality:  
         Score      Num Spectra      Num Peptides      High-Qual Peptides      % Coverage       242    6    4    4    32   
   
      Peptides:   
        Query    Observed    Mr(expt)    Mr(calc)    Score    Peptide    Result File   
		    551    733.96    1465.90    1465.78    69    AGSQVSGPVPLPTEK    37Rep_1   
		    276    798.10    1594.19    1593.87    57    KAGSQVSGPVPLPTEK    37RepA_1   
		    281    541.35    1080.69    1080.62    67    LIDIANPTPK    37Rep_1   
		    30    425.82    849.62    849.46    49    LLDFSAGK    37RepA_1   
   
      Matching Genes:  
               gi|115249076|emb|CAJ66887.1|  (30S ribosomal protein S10 [Clostridium difficile 630]) 
           
  Protein Group 49   
      Expression Quality:  
         Score      Num Spectra      Num Peptides      High-Qual Peptides      % Coverage       238    7    6    3    22   
   
      Peptides:   
        Query    Observed    Mr(expt)    Mr(calc)    Score    Peptide    Result File   
		    213    612.95    1223.89    1223.63    42    AGATYVSPFVGR    37Rep_3   
		    112    480.36    958.71    958.52    46    IPMTAEGLK    37Rep_3   
		    110    558.41    1114.80    1114.61    38    MGADIATVPLK    37RepA_3   
		    179    566.40    1130.78    1130.60    34    MGADIATVPLK +Oxidation (M)    37Rep_3   
		    172    559.89    1117.77    1117.64    37    NPIHVLQAAR    37Rep_3   
		    33    423.36    844.71    844.48    41    VINQMIK    37Rep_3   
   
      Matching Genes:  
               gi|115251384|emb|CAJ69216.1|  (putative transaldolase [Clostridium difficile 630]) 
           
  Protein Group 50   
      Expression Quality:  
         Score      Num Spectra      Num Peptides      High-Qual Peptides      % Coverage       236    7    5    2    28   
   
      Peptides:   
        Query    Observed    Mr(expt)    Mr(calc)    Score    Peptide    Result File   
		    240    422.96    1265.87    1265.75    34    EGKLPVVKPGSR    37Rep_3   
		    477    962.21    1922.41    1922.00    74    FPEYAAEVLSTVVEQIK    37Rep_3   
		    237    630.05    1258.09    1257.72    65    IIIAEDVVTTGK    37Rep_3   
		    79    476.91    951.80    951.59    35    LPVVKPGSR    37RepA_3   
		    378    788.47    1574.93    1574.78    28    TNHDIGMPIYSAIK +Oxidation (M)    37Rep_3   
   
      Matching Genes:  
               gi|115249197|emb|CAJ67009.1|  (orotate phosphoribosyltransferase [Clostridium difficile 630]) 
           
  Protein Group 51   
      Expression Quality:  
         Score      Num Spectra      Num Peptides      High-Qual Peptides      % Coverage       235    5    4    4    37   
   
      Peptides:   
        Query    Observed    Mr(expt)    Mr(calc)    Score    Peptide    Result File   
		    244    738.09    1474.16    1473.79    71    IGVIGGGSITYPNAR    37RepA_1   
		    127    572.36    1142.71    1142.52    69    LSETDEFFR    37RepA_1   
		    215    478.28    954.54    954.47    46    TYSEGAISK    37Rep_1   
		    330    606.39    1816.16    1815.89    49    VFGSLDDKTYSEGAISK    37RepA_1   
   
      Matching Genes:  
               gi|115251040|emb|CAJ68871.1|  (putative decarboxylase [Clostridium difficile 630]) 
           
  Protein Group 52   
      Expression Quality:  
         Score      Num Spectra      Num Peptides      High-Qual Peptides      % Coverage       235    6    4    3    46   
   
      Peptides:   
        Query    Observed    Mr(expt)    Mr(calc)    Score    Peptide    Result File   
		    98    515.38    1028.74    1028.55    67    IANGEIPSTK    37RepA_1   
		    300    567.34    1132.66    1132.55    55    KLPNYEAGQN    37Rep_1   
		    556    738.90    1475.78    1475.67    83    VINNCGSDGGQEVK    37Rep_1   
		    401    740.55    2218.63    2218.25    30    VLAFNDLNPVAPYHILVVPK    37RepA_1   
   
      Matching Genes:  
               gi|115251501|emb|CAJ69334.1|  (histidine triad nucleotide-binding protein [Clostridium difficile 630]) 
           
  Protein Group 53   
      Expression Quality:  
         Score      Num Spectra      Num Peptides      High-Qual Peptides      % Coverage       228    6    5    3    14   
   
      Peptides:   
        Query    Observed    Mr(expt)    Mr(calc)    Score    Peptide    Result File   
		    163    669.44    1336.87    1336.61    53    DTDPQSALEYAK    37Rep_6   
		    484    832.71    2495.10    2494.24    42    ENSLHVMGLLSDGGVHSHIDHLK    37Rep_6   
		    352    1049.86    2097.71    2096.97    75    EVQASMDEIGVGEFATVSGR +Oxidation (M)    37Rep_6   
		    345    1037.81    2073.61    2073.00    30    VATYDLKPEMSAYELTDK    37Rep_6   
		    307    1045.80    2089.58    2088.99    28    VATYDLKPEMSAYELTDK +Oxidation (M)    37RepA_6   
   
      Matching Genes:  
               gi|115252228|emb|CAJ70068.1|  (2,3-bisphosphoglycerate-independent phosphoglycerate mutase [Clostridium difficile 630]) 
           
  Protein Group 54   
      Expression Quality:  
         Score      Num Spectra      Num Peptides      High-Qual Peptides      % Coverage       227    7    4    4    33   
   
      Peptides:   
        Query    Observed    Mr(expt)    Mr(calc)    Score    Peptide    Result File   
		    217    556.40    1110.79    1110.64    47    ADLDLRPALK    37Rep_2   
		    113    564.96    1127.91    1127.54    41    ANVQYYGTGR    37RepA_2   
		    332    656.47    1310.93    1310.73    96    LVAGEGNILVNGR    37Rep_2   
		    139    606.98    1211.94    1211.65    43    QPLVLTGNENK    37RepA_2   
   
      Matching Genes:  
               gi|115249113|emb|CAJ66924.1|  (30S ribosomal protein S9 [Clostridium difficile 630]) 
           
  Protein Group 55   
      Expression Quality:  
         Score      Num Spectra      Num Peptides      High-Qual Peptides      % Coverage       227    7    4    4    52   
   
      Peptides:   
        Query    Observed    Mr(expt)    Mr(calc)    Score    Peptide    Result File   
		    366    679.16    2034.45    2034.06    54    EQPQIAEVVEVGPGGIVEGK    37RepA_1   
		    354    611.35    1220.69    1220.64    58    IEGQEYTILR    37Rep_1   
		    74    454.80    907.58    907.43    46    MELTVGDK +Oxidation (M)    37RepA_1   
		    283    542.85    1083.69    1083.63    69    TASGIVLPGAAK    37Rep_1   
   
      Matching Genes:  
               gi|115249203|emb|CAJ67015.1|  (10 kDa chaperonin [Clostridium difficile 630]) 
           
  Protein Group 56   
      Expression Quality:  
         Score      Num Spectra      Num Peptides      High-Qual Peptides      % Coverage       226    8    5    2    12   
   
      Peptides:   
        Query    Observed    Mr(expt)    Mr(calc)    Score    Peptide    Result File   
		    272    638.86    1913.56    1912.98    36    AIGDNLTCIFVDHGLLR    37RepA_6   
		    43    445.87    889.73    889.50    37    FDINLIR    37Rep_6   
		    373    1084.34    2166.67    2166.08    76    GIIFTGGPNSAYLEDSPTISK    37Rep_6   
		    63    488.90    975.79    975.54    42    IIGEEFIR    37Rep_6   
		    49    511.94    1021.86    1021.49    35    MPYDVLER    37RepA_6   
   
      Matching Genes:  
               gi|115249206|emb|CAJ67019.1|  (GMP synthase [glutamine-hydrolyzing] [Clostridium difficile 630]) 
           
  Protein Group 57   
      Expression Quality:  
         Score      Num Spectra      Num Peptides      High-Qual Peptides      % Coverage       222    6    5    3    13   
   
      Peptides:   
        Query    Observed    Mr(expt)    Mr(calc)    Score    Peptide    Result File   
		    14    420.89    839.76    839.49    34    APSIPSLR    37RepA_6   
		    175    748.89    1495.76    1495.66    40    EMGDNCSVTILNK +Oxidation (M)    37RepA_6   
		    431    757.91    2270.72    2270.05    44    NSLTNKPTWSPMGSSANHEGR    37Rep_6   
		    193    793.12    1584.24    1583.77    36    TLSTGEKENLSYDK    37RepA_6   
		    203    732.10    1462.18    1461.82    68    VLEGGILFYPNLK    37Rep_6   
   
      Matching Genes:  
               gi|115250843|emb|CAJ68667.1|  (putative pyridine nucleotide-disulfide oxidoreductase [Clostridium difficile 630]) 
           
  Protein Group 58   
      Expression Quality:  
         Score      Num Spectra      Num Peptides      High-Qual Peptides      % Coverage       219    6    4    3    12   
   
      Peptides:   
        Query    Observed    Mr(expt)    Mr(calc)    Score    Peptide    Result File   
		    59    465.37    928.72    928.56    55    IGIEGSILK    37Rep_5   
		    130    646.04    1290.06    1289.70    64    ILVLNCGSSSLK    37RepA_5   
		    260    822.74    1643.47    1642.88    38    MLLIPTNEELMIAR    37Rep_5   
		    262    827.06    1652.12    1651.75    62    YGFHGTSHNYVSQR    37Rep_5   
   
      Matching Genes:  
               gi|115250207|emb|CAJ68028.1|  (acetate kinase [Clostridium difficile 630]) 
           
  Protein Group 59   
      Expression Quality:  
         Score      Num Spectra      Num Peptides      High-Qual Peptides      % Coverage       216    7    5    2    7   
   
      Peptides:   
        Query    Observed    Mr(expt)    Mr(calc)    Score    Peptide    Result File   
		    267    892.20    1782.39    1781.79    41    DVEFGELNSQDKDMR    37Rep_7   
		    453    1208.36    2414.70    2414.09    27    MINSEEFNGLDASEGFEGIIDK    37Rep_7   
		    461    1216.39    2430.76    2430.09    39    MINSEEFNGLDASEGFEGIIDK +Oxidation (M)    37Rep_7   
		    54    481.87    961.72    961.52    29    YTIHSTLK    37Rep_7   
		    213    771.99    1541.97    1541.66    80    YVDSNNENEPFSK    37Rep_7   
   
      Matching Genes:  
               gi|115251575|emb|CAJ69408.1|  (leucyl-tRNA synthetase [Clostridium difficile 630]) 
           
  Protein Group 60   
      Expression Quality:  
         Score      Num Spectra      Num Peptides      High-Qual Peptides      % Coverage       215    4    3    3    15   
   
      Peptides:   
        Query    Observed    Mr(expt)    Mr(calc)    Score    Peptide    Result File   
		    335    901.78    1801.54    1801.01    93    ILFVAGGVGSAPVYPQVK    37Rep_4   
		    77    588.45    1174.88    1175.62    46    IPLTIADYDR    37RepA_4   
		    322    883.08    1764.14    1763.78    76    NVYVSTDDGTYGFNGR    37Rep_4   
   
      Matching Genes:  
               gi|115250577|emb|CAJ68401.1|  (putative dehydrogenase, electron transfer subunit [Clostridium difficile 630]) 
           
  Protein Group 61   
      Expression Quality:  
         Score      Num Spectra      Num Peptides      High-Qual Peptides      % Coverage       213    10    4    3    28   
   
      Peptides:   
        Query    Observed    Mr(expt)    Mr(calc)    Score    Peptide    Result File   
		    370    776.05    1550.09    1549.72    30    EVSWLPSYGPEMR    37Rep_3   
		    104    538.37    1074.72    1074.57    62    FKDDVIPGGK    37RepA_3   
		    176    493.89    985.77    985.62    62    IIVNSSLIK    37Rep_2   
		    556    1155.76    2309.51    2309.11    59    TDVDVYYIPANELAAELGNDK    37Rep_3   
   
      Matching Genes:  
               gi|115249127|emb|CAJ66938.1|  (putative subunit of oxidoreductase [Clostridium difficile 630]) 
           
  Protein Group 62   
      Expression Quality:  
         Score      Num Spectra      Num Peptides      High-Qual Peptides      % Coverage       203    5    4    3    36   
   
      Peptides:   
        Query    Observed    Mr(expt)    Mr(calc)    Score    Peptide    Result File   
		    616    894.36    2680.06    2679.19    46    DMNEFGAINEVYAEYFGENKPAR +Oxidation (M)    37Rep_2   
		    407    744.88    2231.61    2231.13    70    HEVIHTNDAPAALGPYSQAIK    37RepA_1   
		    728    831.24    2490.70    2490.27    61    MKHEVIHTNDAPAALGPYSQAIK    37Rep_1   
		    733    836.32    2505.94    2506.26    26    MKHEVIHTNDAPAALGPYSQAIK +Oxidation (M)    37Rep_1   
   
      Matching Genes:  
               gi|115251566|emb|CAJ69399.1|  (putative translation inhibitor endoribonuclease [Clostridium difficile 630]) 
           
  Protein Group 63   
      Expression Quality:  
         Score      Num Spectra      Num Peptides      High-Qual Peptides      % Coverage       203    5    4    3    18   
   
      Peptides:   
        Query    Observed    Mr(expt)    Mr(calc)    Score    Peptide    Result File   
		    74    578.91    1155.80    1155.52    66    FFEGDIEGSR    37RepA_4   
		    71    573.41    1144.81    1144.59    42    HFETIANSVK    37RepA_4   
		    104    628.05    1254.08    1253.75    59    LPIILYNVPGR    37RepA_4   
		    410    824.31    2469.91    2469.18    36    VPVIAGSGSNDTMHSVNLSQEAEK    37RepA_4   
   
      Matching Genes:  
               gi|115252282|emb|CAJ70123.1|  (dihydrodipicolinate synthase [Clostridium difficile 630]) 
              Other Genes Matching Peptide Subset:  
               gi|115252280|emb|CAJ70121.1|  (dihydrodipicolinate synthase [Clostridium difficile 630]) 
           
  Protein Group 64   
      Expression Quality:  
         Score      Num Spectra      Num Peptides      High-Qual Peptides      % Coverage       202    4    4    4    24   
   
      Peptides:   
        Query    Observed    Mr(expt)    Mr(calc)    Score    Peptide    Result File   
		    264    870.95    1739.88    1738.88    65    AAALDTFETEGLFLNK    37RepA_5   
		    267    844.17    1686.33    1685.88    43    GGLINTGDLIEALESGK    37Rep_5   
		    496    1321.49    2640.96    2640.31    50    GLGANVIAFDQYPNSDLNDILTYK    37Rep_7   
		    509    1354.96    2707.90    2707.30    44    GYDGISIQQTNYIDNPYIYETLK    37Rep_7   
   
      Matching Genes:  
               gi|115249400|emb|CAJ67215.1|  ((R)-2-hydroxyisocaproate dehydrogenase [Clostridium difficile 630]) 
           
  Protein Group 65   
      Expression Quality:  
         Score      Num Spectra      Num Peptides      High-Qual Peptides      % Coverage       202    5    3    3    5   
   
      Peptides:   
        Query    Observed    Mr(expt)    Mr(calc)    Score    Peptide    Result File   
		    201    532.04    1593.11    1592.78    44    AGTNMERPGPLAAHR +Oxidation (M)    37RepA_6   
		    282    809.12    1616.23    1615.81    88    IVLEENEQSLPMSK    37RepA_3   
		    399    817.07    1632.12    1631.81    70    IVLEENEQSLPMSK +Oxidation (M)    37Rep_3   
   
      Matching Genes:  
               gi|115252300|emb|CAJ70141.1|  (proline reductase subunit proprotein [Clostridium difficile 630]) 
           
  Protein Group 66   
      Expression Quality:  
         Score      Num Spectra      Num Peptides      High-Qual Peptides      % Coverage       195    7    5    2    6   
   
      Peptides:   
        Query    Observed    Mr(expt)    Mr(calc)    Score    Peptide    Result File   
		    141    456.70    1367.09    1366.68    53    FWATELTEDKK    37RepA_8   
		    258    571.46    1711.37    1710.88    54    KGDEVYYVSKPLADK    37Rep_8   
		    179    456.64    1366.90    1366.72    32    LNAHETANKLEK    37Rep_8   
		    205    582.45    1744.33    1743.81    26    SLEEQNLNDHMTGIK +Oxidation (M)    37RepA_8   
		    140    456.68    1367.02    1366.64    30    VVHNYPHCWR    37RepA_8   
   
      Matching Genes:  
               gi|115251669|emb|CAJ69504.1|  (isoleucyl-tRNA synthetase [Clostridium difficile 630]) 
           
  Protein Group 67   
      Expression Quality:  
         Score      Num Spectra      Num Peptides      High-Qual Peptides      % Coverage       192    5    4    3    13   
   
      Peptides:   
        Query    Observed    Mr(expt)    Mr(calc)    Score    Peptide    Result File   
		    126    495.35    988.68    988.49    27    DIKPEEMK    37Rep_3   
		    101    532.92    1063.83    1063.54    53    VAAMIYGPDK    37RepA_3   
		    160    540.85    1079.69    1079.53    59    VAAMIYGPDK +Oxidation (M)    37Rep_3   
		    91    456.91    911.80    911.58    53    VIVIAGVNK    37Rep_3   
   
      Matching Genes:  
               gi|115251115|emb|CAJ68946.1|  (conserved hypothetical protein [Clostridium difficile 630]) 
           
  Protein Group 68   
      Expression Quality:  
         Score      Num Spectra      Num Peptides      High-Qual Peptides      % Coverage       183    9    4    2    16   
   
      Peptides:   
        Query    Observed    Mr(expt)    Mr(calc)    Score    Peptide    Result File   
		    163    634.53    1267.05    1266.73    38    ALVPVVVEQTGR    37RepA_3   
		    132    602.48    1202.94    1202.61    47    ETLNEILSER    37RepA_3   
		    334    723.01    1444.01    1443.79    61    IKETLNEILSER    37Rep_3   
		    59    444.37    886.72    886.42    37    SYDIFSR    37RepA_3   
   
      Matching Genes:  
               gi|115252361|emb|CAJ70202.1|  (ATP-dependent Clp protease proteolytic subunit [Clostridium difficile 630]) 
           
  Protein Group 69   
      Expression Quality:  
         Score      Num Spectra      Num Peptides      High-Qual Peptides      % Coverage       182    4    4    2    10   
   
      Peptides:   
        Query    Observed    Mr(expt)    Mr(calc)    Score    Peptide    Result File   
		    235    788.56    1575.11    1574.66    57    EFECYTQEQVDK    37Rep_6   
		    94    586.49    1170.96    1170.61    37    IAGVEIPEDTK    37Rep_6   
		    173    747.54    1493.07    1492.72    60    NIEYAGENIEVSR    37RepA_6   
		    65    494.38    986.74    986.54    28    SVGIIDIDR    37Rep_6   
   
      Matching Genes:  
               gi|115251397|emb|CAJ69229.1|  (succinate-semialdehyde dehydrogenase [NAD(P)+] [Clostridium difficile 630]) 
           
  Protein Group 70   
      Expression Quality:  
         Score      Num Spectra      Num Peptides      High-Qual Peptides      % Coverage       178    5    3    3    11   
   
      Peptides:   
        Query    Observed    Mr(expt)    Mr(calc)    Score    Peptide    Result File   
		    241    831.65    1661.28    1660.76    54    AHCSTVGAGEFLQER    37RepA_5   
		    215    839.18    1676.34    1675.81    62    EVVFAADDNVVGENAK    37RepA_6   
		    201    745.61    1489.20    1488.72    62    MENGDVVLLENTR    37RepA_5   
   
      Matching Genes:  
               gi|115252230|emb|CAJ70070.1|  (phosphoglycerate kinase [Clostridium difficile 630]) 
           
  Protein Group 71   
      Expression Quality:  
         Score      Num Spectra      Num Peptides      High-Qual Peptides      % Coverage       171    7    4    2    25   
   
      Peptides:   
        Query    Observed    Mr(expt)    Mr(calc)    Score    Peptide    Result File   
		    312    574.79    1721.35    1720.92    35    KPEEVISHAVSGMLPK    37RepA_2   
		    475    580.03    1737.07    1736.91    44    KPEEVISHAVSGMLPK +Oxidation (M)    37Rep_2   
		    137    604.99    1207.97    1207.62    36    KWYLVDAEGK    37RepA_2   
		    210    547.81    1093.60    1093.56    56    YHTGYVGGLK    37Rep_2   
   
      Matching Genes:  
               gi|115249112|emb|CAJ66923.1|  (50S ribosomal protein L13 [Clostridium difficile 630]) 
           
  Protein Group 72   
      Expression Quality:  
         Score      Num Spectra      Num Peptides      High-Qual Peptides      % Coverage       169    5    3    2    9   
   
      Peptides:   
        Query    Observed    Mr(expt)    Mr(calc)    Score    Peptide    Result File   
		    84    608.37    1214.73    1214.52    36    NYDKEEFDR    37RepA_6   
		    385    1098.87    2195.73    2195.04    71    QLATEEGYETFVIPDDVGGR    37Rep_6   
		    102    625.97    1249.93    1249.63    62    SGTTTEPALAFR    37RepA_6   
   
      Matching Genes:  
               gi|115252341|emb|CAJ70182.1|  (glucose-6-phosphate isomerase [Clostridium difficile 630]) 
           
  Protein Group 73   
      Expression Quality:  
         Score      Num Spectra      Num Peptides      High-Qual Peptides      % Coverage       167    5    4    2    37   
   
      Peptides:   
        Query    Observed    Mr(expt)    Mr(calc)    Score    Peptide    Result File   
		    633    584.37    1750.09    1749.89    34    HQKPSAMNQQGGIINK    37Rep_1   
		    636    589.65    1765.93    1765.89    44    HQKPSAMNQQGGIINK +Oxidation (M)    37Rep_1   
		    112    543.93    1085.85    1085.64    30    KGDTVVVIAGK    37RepA_1   
		    134    585.93    1169.84    1169.70    59    VLVEGVNVITK    37RepA_1   
   
      Matching Genes:  
               gi|115249088|emb|CAJ66899.1|  (50S ribosomal protein L24 [Clostridium difficile 630]) 
           
  Protein Group 74   
      Expression Quality:  
         Score      Num Spectra      Num Peptides      High-Qual Peptides      % Coverage       166    5    3    3    22   
   
      Peptides:   
        Query    Observed    Mr(expt)    Mr(calc)    Score    Peptide    Result File   
		    87    486.92    971.83    971.53    56    ILGEGNLEK    37RepA_2   
		    340    440.64    1318.91    1318.74    41    LHELKPAEGAVR    37Rep_2   
		    303    631.42    1260.83    1260.63    69    VGFEGGQMPLAR    37Rep_2   
   
      Matching Genes:  
               gi|115249096|emb|CAJ66907.1|  (50S ribosomal protein L15 [Clostridium difficile 630]) 
           
  Protein Group 75   
      Expression Quality:  
         Score      Num Spectra      Num Peptides      High-Qual Peptides      % Coverage       161    5    3    3    23   
   
      Peptides:   
        Query    Observed    Mr(expt)    Mr(calc)    Score    Peptide    Result File   
		    231    583.40    1164.79    1164.60    45    EQALVEVSYK    37Rep_2   
		    403    730.59    1459.17    1458.78    70    ILGGGLPYESAVQR    37Rep_2   
		    198    530.84    1059.67    1058.60    46    VKVETGIDAK    37Rep_2   
   
      Matching Genes:  
               gi|115249807|emb|CAJ67624.1|  (putative NUDIX-family hydrolase [Clostridium difficile 630]) 
           
  Protein Group 76   
      Expression Quality:  
         Score      Num Spectra      Num Peptides      High-Qual Peptides      % Coverage       160    5    3    2    29   
   
      Peptides:   
        Query    Observed    Mr(expt)    Mr(calc)    Score    Peptide    Result File   
		    239    730.50    1458.99    1458.76    81    AEGDTISANLDLLK    37RepA_1   
		    236    479.59    1435.74    1435.63    46    SLDNGDLDHEHGK    37RepA_1   
		    113    544.37    1086.72    1086.54    33    VCIPVEENK    37RepA_1   
   
      Matching Genes:  
               gi|115250736|emb|CAJ68560.1|  (putative dinitrogenase iron-molybdenum cofactor [Clostridium difficile 630]) 
           
  Protein Group 77   
      Expression Quality:  
         Score      Num Spectra      Num Peptides      High-Qual Peptides      % Coverage       160    6    3    2    9   
   
      Peptides:   
        Query    Observed    Mr(expt)    Mr(calc)    Score    Peptide    Result File   
		    192    528.78    1583.32    1582.75    41    FGTATHSGFGLGFER    37RepA_6   
		    92    617.48    1232.95    1232.57    37    IDYTQDFFGK    37RepA_6   
		    229    869.64    1737.26    1736.85    82    SIVVEGESDSSYPLQK    37RepA_6   
   
      Matching Genes:  
               gi|115251299|emb|CAJ69130.1|  (asparaginyl-tRNA synthetase [Clostridium difficile 630]) 
           
  Protein Group 78   
      Expression Quality:  
         Score      Num Spectra      Num Peptides      High-Qual Peptides      % Coverage       159    6    3    3    17   
   
      Peptides:   
        Query    Observed    Mr(expt)    Mr(calc)    Score    Peptide    Result File   
		    88    487.30    972.59    972.54    47    GLHDYIKK    37RepA_2   
		    98    530.45    1058.89    1058.61    70    LASGFPIGLGK    37RepA_2   
		    195    529.30    1056.58    1056.49    42    NISEEYFR    37Rep_2   
   
      Matching Genes:  
               gi|115252075|emb|CAJ69912.1|  (conserved hypothetical protein [Clostridium difficile 630]) 
           
  Protein Group 79   
      Expression Quality:  
         Score      Num Spectra      Num Peptides      High-Qual Peptides      % Coverage       159    4    3    3    22   
   
      Peptides:   
        Query    Observed    Mr(expt)    Mr(calc)    Score    Peptide    Result File   
		    400    481.98    1442.92    1442.66    48    AHNDANMLSLGER +Oxidation (M)    37Rep_2   
		    235    591.83    1181.65    1181.57    57    CAVVSDVFSAK    37Rep_2   
		    206    541.40    1080.78    1080.57    54    EIISYLESK    37Rep_2   
   
      Matching Genes:  
               gi|115252540|emb|CAJ70383.1|  (ribose-5-phosphate isomerase 2 [Clostridium difficile 630]) 
           
  Protein Group 80   
      Expression Quality:  
         Score      Num Spectra      Num Peptides      High-Qual Peptides      % Coverage       157    5    2    2    16   
   
      Peptides:   
        Query    Observed    Mr(expt)    Mr(calc)    Score    Peptide    Result File   
		    390    800.51    1599.00    1598.72    72    NLSDNHESQEAEVK    37Rep_3   
		    241    739.04    1476.06    1475.76    85    VEAGIYNEILNNK    37RepA_3   
   
      Matching Genes:  
               gi|115252635|emb|CAJ70478.1|  (putative preprotein translocase [Clostridium difficile 630]) 
           
  Protein Group 81   
      Expression Quality:  
         Score      Num Spectra      Num Peptides      High-Qual Peptides      % Coverage       154    7    4    2    30   
   
      Peptides:   
        Query    Observed    Mr(expt)    Mr(calc)    Score    Peptide    Result File   
		    242    498.27    994.53    994.46    38    GYDVIEDGK    37Rep_1   
		    107    545.46    1088.90    1088.62    43    ILLEEGFIR    37RepA_2   
		    224    698.95    1395.89    1395.64    25    TMTDPIADMLTR +2 Oxidation (M)    37RepA_1   
		    126    571.38    1140.74    1140.59    48    VYAANHEIPK    37RepA_1   
   
      Matching Genes:  
               gi|115249091|emb|CAJ66902.1|  (30S ribosomal protein S8 [Clostridium difficile 630]) 
           
  Protein Group 82   
      Expression Quality:  
         Score      Num Spectra      Num Peptides      High-Qual Peptides      % Coverage       153    6    4    1    11   
   
      Peptides:   
        Query    Observed    Mr(expt)    Mr(calc)    Score    Peptide    Result File   
		    113    615.94    1229.86    1229.60    37    HVFEGESNIAK    37Rep_6   
		    413    1121.49    2240.97    2241.13    26    IIPNEASQEAALNNGEISLMK    37Rep_6   
		    68    579.43    1156.84    1156.51    25    NEMIESAYGK +Oxidation (M)    37RepA_6   
		    219    846.59    1691.16    1690.74    65    SNSNLQTYTYSEER    37RepA_6   
   
      Matching Genes:  
               gi|115251723|emb|CAJ69558.1|  (oligopeptide ABC transporter, substrate-binding protein [Clostridium difficile 630]) 
           
  Protein Group 83   
      Expression Quality:  
         Score      Num Spectra      Num Peptides      High-Qual Peptides      % Coverage       152    4    3    3    15   
   
      Peptides:   
        Query    Observed    Mr(expt)    Mr(calc)    Score    Peptide    Result File   
		    382    793.07    1584.12    1583.83    51    GVWNFAPLDLEVPK    37Rep_3   
		    135    500.80    999.59    999.51    50    NGAQEVINR    37Rep_3   
		    82    482.88    963.74    963.50    51    YLGDLLDR    37RepA_3   
   
      Matching Genes:  
               gi|115249180|emb|CAJ66992.1|  (putative DNA-binding protein [Clostridium difficile 630]) 
           
  Protein Group 84   
      Expression Quality:  
         Score      Num Spectra      Num Peptides      High-Qual Peptides      % Coverage       152    4    3    3    11   
   
      Peptides:   
        Query    Observed    Mr(expt)    Mr(calc)    Score    Peptide    Result File   
		    135    588.91    1175.81    1175.61    51    ALLDAFHYAR    37RepA_1   
		    438    791.47    1580.93    1580.79    55    NTDIKEEYLSEIK    37Rep_2   
		    89    511.82    1021.63    1021.47    46    SDMQVVTDK    37Rep_4   
   
      Matching Genes:  
               gi|115250155|emb|CAJ67976.1|  (nitroreductase-family protein [Clostridium difficile 630]) 
           
  Protein Group 85   
      Expression Quality:  
         Score      Num Spectra      Num Peptides      High-Qual Peptides      % Coverage       151    7    3    2    14   
   
      Peptides:   
        Query    Observed    Mr(expt)    Mr(calc)    Score    Peptide    Result File   
		    54    520.43    1038.84    1038.62    41    AEAIVKPGVR    37RepA_4   
		    469    1007.48    3019.43    3018.50    30    DDISQMLVSDPTSIFYLTGVLIHPGER +Oxidation (M)    37RepA_4   
		    298    680.92    1359.83    1359.78    80    FLLNLIELGGGSK    37Rep_3   
   
      Matching Genes:  
               gi|115251664|emb|CAJ69499.1|  (probable peptidase [Clostridium difficile 630]) 
           
  Protein Group 86   
      Expression Quality:  
         Score      Num Spectra      Num Peptides      High-Qual Peptides      % Coverage       149    4    3    2    24   
   
      Peptides:   
        Query    Observed    Mr(expt)    Mr(calc)    Score    Peptide    Result File   
		    226    706.99    1411.97    1411.68    64    QNFGQVSNSYIR    37RepA_3   
		    391    1081.84    2161.67    2160.97    26    TGEGDGDDEEILVNLETMPK    37RepA_3   
		    440    887.63    1773.26    1772.86    59    VEKDEDFIFYNNLK    37Rep_3   
   
      Matching Genes:  
               gi|115250676|emb|CAJ68500.1|  (tellurium resistance protein [Clostridium difficile 630]) 
           
  Protein Group 87   
      Expression Quality:  
         Score      Num Spectra      Num Peptides      High-Qual Peptides      % Coverage       149    4    2    2    19   
   
      Peptides:   
        Query    Observed    Mr(expt)    Mr(calc)    Score    Peptide    Result File   
		    326    905.18    1808.35    1807.91    65    SIMGIMSLGLAQGEELK +2 Oxidation (M)    37RepA_1   
		    319    897.20    1792.39    1791.91    84    SIMGIMSLGLAQGEELK +Oxidation (M)    37RepA_1   
   
      Matching Genes:  
               gi|115251809|emb|CAJ69644.1|  (PTS system, phosphocarrier protein [Clostridium difficile 630]) 
           
  Protein Group 88   
      Expression Quality:  
         Score      Num Spectra      Num Peptides      High-Qual Peptides      % Coverage       146    3    3    2    7   
   
      Peptides:   
        Query    Observed    Mr(expt)    Mr(calc)    Score    Peptide    Result File   
		    422    835.28    2502.82    2502.18    36    GVAAVVGCSNMTAGGHDVNTVELTK +Oxidation (M)    37Rep_8   
		    238    810.10    1618.18    1617.80    64    LVGCTCVGQDLQLR    37Rep_8   
		    76    516.44    1030.86    1030.59    46    LVTEVLTEK    37Rep_8   
   
      Matching Genes:  
               gi|115249184|emb|CAJ66996.1|  (putative oxidoreductase, acetyl-CoA synthase subunit [Clostridium difficile 630]) 
           
  Protein Group 89   
      Expression Quality:  
         Score      Num Spectra      Num Peptides      High-Qual Peptides      % Coverage       145    4    3    1    40   
   
      Peptides:   
        Query    Observed    Mr(expt)    Mr(calc)    Score    Peptide    Result File   
		    170    452.26    902.51    902.56    36    KLAYPIAK    37Rep_1   
		    373    685.16    2052.46    2052.02    31    NYELVYVVKPNSDEEVR    37RepA_1   
		    219    694.02    1386.02    1385.78    78    VKEVVATDGEIVK    37RepA_1   
   
      Matching Genes:  
               gi|115252728|emb|CAJ70572.1|  (30S ribosomal protein S6 [Clostridium difficile 630]) 
           
  Protein Group 90   
      Expression Quality:  
         Score      Num Spectra      Num Peptides      High-Qual Peptides      % Coverage       136    4    3    2    41   
   
      Peptides:   
        Query    Observed    Mr(expt)    Mr(calc)    Score    Peptide    Result File   
		    204    675.39    1348.77    1348.59    68    GCGLCVEACPVK    37RepA_1   
		    165    628.98    1255.94    1255.71    42    IIQLDSNVINK    37RepA_1   
		    57    440.32    878.63    878.42    26    VSFNQER    37RepA_1   
   
      Matching Genes:  
               gi|115249124|emb|CAJ66935.1|  (ferredoxin [Clostridium difficile 630]) 
           
  Protein Group 91   
      Expression Quality:  
         Score      Num Spectra      Num Peptides      High-Qual Peptides      % Coverage       135    4    3    2    7   
   
      Peptides:   
        Query    Observed    Mr(expt)    Mr(calc)    Score    Peptide    Result File   
		    181    721.44    1440.87    1440.76    53    GVVDVYPNKPEPK    37RepA_7   
		    314    962.33    1922.65    1921.94    56    SLGLAGVMGGANSEITSNTK +Oxidation (M)    37Rep_7   
		    420    796.71    2387.10    2386.19    26    YVGTLGELHPDVIENYNLGQR    37RepA_7   
   
      Matching Genes:  
               gi|115249716|emb|CAJ67533.1|  (phenylalanyl-tRNA synthetase beta chain [Clostridium difficile 630]) 
           
  Protein Group 92   
      Expression Quality:  
         Score      Num Spectra      Num Peptides      High-Qual Peptides      % Coverage       129    4    3    1    20   
   
      Peptides:   
        Query    Observed    Mr(expt)    Mr(calc)    Score    Peptide    Result File   
		    148    458.30    914.58    914.45    34    AEINPDTR    37Rep_2   
		    72    465.93    929.84    929.52    33    ATANEILAK    37RepA_2   
		    56    420.83    839.65    839.49    62    IAGVDLPR    37Rep_2   
   
      Matching Genes:  
               gi|115249103|emb|CAJ66914.1|  (30S ribosomal protein S13 [Clostridium difficile 630]) 
           
  Protein Group 93   
      Expression Quality:  
         Score      Num Spectra      Num Peptides      High-Qual Peptides      % Coverage       124    2    2    2    5   
   
      Peptides:   
        Query    Observed    Mr(expt)    Mr(calc)    Score    Peptide    Result File   
		    470    1242.38    2482.74    2482.19    54    EALEELGLPYTINEGDGAFYGPK    37Rep_7   
		    77    551.92    1101.82    1101.54    70    EVADNNVSVR    37RepA_7   
   
      Matching Genes:  
               gi|115249589|emb|CAJ67406.1|  (threonyl-tRNA synthetase [Clostridium difficile 630]) 
           
  Protein Group 94   
      Expression Quality:  
         Score      Num Spectra      Num Peptides      High-Qual Peptides      % Coverage       118    4    2    2    15   
   
      Peptides:   
        Query    Observed    Mr(expt)    Mr(calc)    Score    Peptide    Result File   
		    226    706.99    1411.97    1411.68    64    QNFGQVSNSYIR    37RepA_3   
		    441    889.14    1776.26    1775.81    54    TGEGDGDDEQIVVDLSK    37Rep_3   
   
      Matching Genes:  
               gi|115250675|emb|CAJ68499.1|  (tellurium resistance protein [Clostridium difficile 630]) 
           
  Protein Group 95   
      Expression Quality:  
         Score      Num Spectra      Num Peptides      High-Qual Peptides      % Coverage       117    3    2    1    15   
   
      Peptides:   
        Query    Observed    Mr(expt)    Mr(calc)    Score    Peptide    Result File   
		    123    584.90    1167.78    1167.58    35    ESVDFPIYAK    37RepA_2   
		    165    624.41    1246.81    1246.60    82    GIEVEDVGTNSK    37RepA_2   
   
      Matching Genes:  
               gi|115251375|emb|CAJ69207.1|  (ribose-5-phosphate isomerase 1 [Clostridium difficile 630]) 
           
  Protein Group 96   
      Expression Quality:  
         Score      Num Spectra      Num Peptides      High-Qual Peptides      % Coverage       117    3    3    1    12   
   
      Peptides:   
        Query    Observed    Mr(expt)    Mr(calc)    Score    Peptide    Result File   
		    400    1003.26    2004.50    2003.92    34    DVYACCTHGVLSGPAIER    37Rep_4   
		    116    579.39    1156.77    1156.54    39    MIFSNESVSK +Oxidation (M)    37Rep_4   
		    117    580.49    1158.97    1158.64    44    TVAPLFGDAIR    37Rep_4   
   
      Matching Genes:  
               gi|115252575|emb|CAJ70418.1|  (ribose-phosphate pyrophosphokinase [Clostridium difficile 630]) 
           
  Protein Group 97   
      Expression Quality:  
         Score      Num Spectra      Num Peptides      High-Qual Peptides      % Coverage       116    4    3    2    11   
   
      Peptides:   
        Query    Observed    Mr(expt)    Mr(calc)    Score    Peptide    Result File   
		    72    574.95    1147.89    1147.56    43    NAETQGYPIR    37RepA_4   
		    76    473.38    944.75    944.48    25    NVEVEEVK    37RepA_3   
		    151    532.84    1063.66    1063.58    48    VAVDTPAHVR    37Rep_3   
   
      Matching Genes:  
               gi|115249126|emb|CAJ66937.1|  (putative subunit of oxidoreductase [Clostridium difficile 630]) 
           
  Protein Group 98   
      Expression Quality:  
         Score      Num Spectra      Num Peptides      High-Qual Peptides      % Coverage       114    2    2    2    8   
   
      Peptides:   
        Query    Observed    Mr(expt)    Mr(calc)    Score    Peptide    Result File   
		    274    855.07    1708.12    1708.83    48    NTSMLDAFAVADDVLK    37Rep_5   
		    118    642.51    1283.01    1282.67    66    VIGVGGGGNNAVNR    37RepA_4   
   
      Matching Genes:  
               gi|115251697|emb|CAJ69532.1|  (cell division protein [Clostridium difficile 630]) 
           
  Protein Group 99   
      Expression Quality:  
         Score      Num Spectra      Num Peptides      High-Qual Peptides      % Coverage       109    2    2    2    11   
   
      Peptides:   
        Query    Observed    Mr(expt)    Mr(calc)    Score    Peptide    Result File   
		    209    671.97    1341.92    1341.71    54    LENIIQNEELK    37RepA_2   
		    275    785.61    1569.21    1568.88    55    VKLENIIQNEELK    37RepA_2   
   
      Matching Genes:  
               gi|115251313|emb|CAJ69144.1|  (conserved hypothetical protein [Clostridium difficile 630]) 
           
  Protein Group 100   
      Expression Quality:  
         Score      Num Spectra      Num Peptides      High-Qual Peptides      % Coverage       106    2    2    1    5   
   
      Peptides:   
        Query    Observed    Mr(expt)    Mr(calc)    Score    Peptide    Result File   
		    218    738.00    1473.99    1473.76    78    FVIGGPQGDTGLTGR    37Rep_5   
		    17    407.27    812.53    813.50    28    LVELVNK    37Rep_1   
   
      Matching Genes:  
               gi|115249139|emb|CAJ66950.1|  (S-adenosylmethionine synthetase [Clostridium difficile 630]) 
           
  Protein Group 101   
      Expression Quality:  
         Score      Num Spectra      Num Peptides      High-Qual Peptides      % Coverage       104    3    2    2    8   
   
      Peptides:   
        Query    Observed    Mr(expt)    Mr(calc)    Score    Peptide    Result File   
		    373    682.89    2045.65    2045.05    46    VLWPFPFEAFNQIPNAR    37Rep_5   
		    122    640.52    1279.02    1278.65    58    VMTSSSSPGVALK +Oxidation (M)    37RepA_5   
   
      Matching Genes:  
               gi|115249125|emb|CAJ66936.1|  (putative oxidoreductase, thiamine diP-binding subunit [Clostridium difficile 630]) 
           
  Protein Group 102   
      Expression Quality:  
         Score      Num Spectra      Num Peptides      High-Qual Peptides      % Coverage       104    2    2    1    4   
   
      Peptides:   
        Query    Observed    Mr(expt)    Mr(calc)    Score    Peptide    Result File   
		    239    543.28    1626.82    1626.77    36    IKEHNHNVGTCYR    37Rep_8   
		    382    1122.87    2243.73    2243.14    68    SLGNGIDPLEIIEQYGADALR    37RepA_7   
   
      Matching Genes:  
               gi|115252312|emb|CAJ70153.1|  (valyl-tRNA synthetase [Clostridium difficile 630]) 
           
  Protein Group 103   
      Expression Quality:  
         Score      Num Spectra      Num Peptides      High-Qual Peptides      % Coverage       102    3    1    1    5   
   
      Peptides:   
        Query    Observed    Mr(expt)    Mr(calc)    Score    Peptide    Result File   
		    314    930.23    1858.45    1857.88    102    TIDEDESGALNPELVEK    37Rep_5   
   
      Matching Genes:  
               gi|115251648|emb|CAJ69481.1|  (low-specificity L-threonine aldolase [Clostridium difficile 630]) 
           
  Protein Group 104   
      Expression Quality:  
         Score      Num Spectra      Num Peptides      High-Qual Peptides      % Coverage       101    3    3    1    5   
   
      Peptides:   
        Query    Observed    Mr(expt)    Mr(calc)    Score    Peptide    Result File   
		    103    599.55    1197.08    1196.71    31    QLVPNLTTAIK    37Rep_6   
		    123    621.04    1240.07    1239.65    40    VDAPEILDNVR    37Rep_6   
		    32    460.38    918.74    918.45    30    YALDYFK    37RepA_6   
   
      Matching Genes:  
               gi|115249342|emb|CAJ67155.1|  (manganese-dependent inorganic pyrophosphatase [Clostridium difficile 630]) 
           
  Protein Group 105   
      Expression Quality:  
         Score      Num Spectra      Num Peptides      High-Qual Peptides      % Coverage       100    2    2    1    15   
   
      Peptides:   
        Query    Observed    Mr(expt)    Mr(calc)    Score    Peptide    Result File   
		    474    946.31    1890.60    1889.90    66    EAEQAGADFVGAEELVQK    37Rep_3   
		    467    624.47    1870.38    1869.94    34    FYDASEALTLVSDIAGAK    37Rep_3   
   
      Matching Genes:  
               gi|115249066|emb|CAJ66877.1|  (50S ribosomal protein L1 [Clostridium difficile 630]) 
           
  Protein Group 106   
      Expression Quality:  
         Score      Num Spectra      Num Peptides      High-Qual Peptides      % Coverage       99    2    2    2    6   
   
      Peptides:   
        Query    Observed    Mr(expt)    Mr(calc)    Score    Peptide    Result File   
		    336    904.17    1806.32    1805.85    59    AGATIGGANSEQMEEITK    37Rep_4   
		    342    912.14    1822.26    1821.84    40    AGATIGGANSEQMEEITK +Oxidation (M)    37Rep_4   
   
      Matching Genes:  
               gi|115250238|emb|CAJ68059.1|  (geranyltranstransferase [Clostridium difficile 630]) 
           
  Protein Group 107   
      Expression Quality:  
         Score      Num Spectra      Num Peptides      High-Qual Peptides      % Coverage       99    4    2    2    12   
   
      Peptides:   
        Query    Observed    Mr(expt)    Mr(calc)    Score    Peptide    Result File   
		    69    564.40    1126.78    1126.54    56    LQAEYANYR    37RepA_4   
		    301    976.20    1950.38    1949.87    43    TDEKEVDDENVTDINSK    37RepA_4   
   
      Matching Genes:  
               gi|115251516|emb|CAJ69349.1|  (heat shock protein [Clostridium difficile 630]) 
           
  Protein Group 108   
      Expression Quality:  
         Score      Num Spectra      Num Peptides      High-Qual Peptides      % Coverage       98    2    1    1    9   
   
      Peptides:   
        Query    Observed    Mr(expt)    Mr(calc)    Score    Peptide    Result File   
		    437    881.61    1761.20    1760.81    98    TGVGDGDDEQINVDLSK    37Rep_3   
   
      Matching Genes:  
               gi|115250845|emb|CAJ68669.1|  (tellurium resistance protein [Clostridium difficile 630]) 
           
  Protein Group 109   
      Expression Quality:  
         Score      Num Spectra      Num Peptides      High-Qual Peptides      % Coverage       98    2    2    1    11   
   
      Peptides:   
        Query    Observed    Mr(expt)    Mr(calc)    Score    Peptide    Result File   
		    411    1034.82    2067.62    2066.98    30    EALDENGLENVGIMAYSVK +Oxidation (M)    37Rep_4   
		    286    937.34    1872.66    1872.01    68    EGLLNEDVILESITSIK    37RepA_4   
   
      Matching Genes:  
               gi|115252479|emb|CAJ70322.1|  (delta-aminolevulinic acid dehydratase [Clostridium difficile 630]) 
           
  Protein Group 110   
      Expression Quality:  
         Score      Num Spectra      Num Peptides      High-Qual Peptides      % Coverage       96    2    1    1    10   
   
      Peptides:   
        Query    Observed    Mr(expt)    Mr(calc)    Score    Peptide    Result File   
		    330    935.69    1869.38    1868.91    96    LAGEGGLFFVDQEFANR    37RepA_2   
   
      Matching Genes:  
               gi|115251248|emb|CAJ69079.1|  (ferritin [Clostridium difficile 630]) 
           
  Protein Group 111   
      Expression Quality:  
         Score      Num Spectra      Num Peptides      High-Qual Peptides      % Coverage       96    3    2    1    21   
   
      Peptides:   
        Query    Observed    Mr(expt)    Mr(calc)    Score    Peptide    Result File   
		    408    734.54    1467.06    1466.75    59    LNDDAIIPNFAHK    37Rep_2   
		    528    696.75    2087.23    2087.05    37    TGICIELPTMTEAQVRPR +Oxidation (M)    37Rep_2   
   
      Matching Genes:  
               gi|115251455|emb|CAJ69288.1|  (deoxyuridine 5'-triphosphate nucleotidohydrolase [Clostridium difficile 630]) 
           
  Protein Group 112   
      Expression Quality:  
         Score      Num Spectra      Num Peptides      High-Qual Peptides      % Coverage       95    2    2    1    20   
   
      Peptides:   
        Query    Observed    Mr(expt)    Mr(calc)    Score    Peptide    Result File   
		    146    457.31    912.60    912.56    57    AVLELAGLK    37Rep_2   
		    421    827.06    2478.16    2477.28    38    GHFGAGNILIMPAVEGTGVIAGGPAR +Oxidation (M)    37RepA_2   
   
      Matching Genes:  
               gi|115249094|emb|CAJ66905.1|  (30S ribosomal protein S5 [Clostridium difficile 630]) 
           
  Protein Group 113   
      Expression Quality:  
         Score      Num Spectra      Num Peptides      High-Qual Peptides      % Coverage       95    2    2    1    8   
   
      Peptides:   
        Query    Observed    Mr(expt)    Mr(calc)    Score    Peptide    Result File   
		    107    630.01    1258.01    1257.66    60    AGINTVEELANK    37RepA_4   
		    234    748.16    1494.30    1493.92    35    ILLSSLPGVAVNAIK    37Rep_5   
   
      Matching Genes:  
               gi|115249106|emb|CAJ66917.1|  (DNA-directed RNA polymerase alpha chain [Clostridium difficile 630]) 
           
  Protein Group 114   
      Expression Quality:  
         Score      Num Spectra      Num Peptides      High-Qual Peptides      % Coverage       94    2    2    2    18   
   
      Peptides:   
        Query    Observed    Mr(expt)    Mr(calc)    Score    Peptide    Result File   
		    340    928.74    1855.47    1854.88    49    GTQFNEGGMPTYSLPLK +Oxidation (M)    37RepA_3   
		    398    716.51    1431.01    1430.76    45    VTSTGIVNGVIEDK    37Rep_2   
   
      Matching Genes:  
               gi|115252223|emb|CAJ70063.1|  (putative phosphatidylethanolamine-binding regulatory protein [Clostridium difficile 630]) 
           
  Protein Group 115   
      Expression Quality:  
         Score      Num Spectra      Num Peptides      High-Qual Peptides      % Coverage       92    3    2    2    24   
   
      Peptides:   
        Query    Observed    Mr(expt)    Mr(calc)    Score    Peptide    Result File   
		    475    671.47    1340.92    1340.73    41    LGSPVVEGASVQAK    37Rep_1   
		    353    611.28    1220.54    1220.63    51    VSEGDVLFVEK    37Rep_1   
   
      Matching Genes:  
               gi|115250193|emb|CAJ68014.1|  (50S ribosomal protein L21 [Clostridium difficile 630]) 
           
  Protein Group 116   
      Expression Quality:  
         Score      Num Spectra      Num Peptides      High-Qual Peptides      % Coverage       90    3    2    1    31   
   
      Peptides:   
        Query    Observed    Mr(expt)    Mr(calc)    Score    Peptide    Result File   
		    651    914.67    1827.32    1826.94    62    AEGDTGSPEVQIALLTAR    37Rep_1   
		    101    519.38    1036.75    1036.57    28    INELNGHLK    37RepA_1   
   
      Matching Genes:  
               gi|115250352|emb|CAJ68174.1|  (30S ribosomal protein S15 [Clostridium difficile 630]) 
           
  Protein Group 117   
      Expression Quality:  
         Score      Num Spectra      Num Peptides      High-Qual Peptides      % Coverage       89    4    2    1    16   
   
      Peptides:   
        Query    Observed    Mr(expt)    Mr(calc)    Score    Peptide    Result File   
		    296    625.44    1248.86    1248.67    56    LAVENNVSYLK    37Rep_2   
		    354    958.22    1914.43    1913.84    33    TYNNTQWDENYPLEK    37RepA_3   
   
      Matching Genes:  
               gi|115249853|emb|CAJ67670.1|  (putative acetyltransferase [Clostridium difficile 630]) 
           
  Protein Group 118   
      Expression Quality:  
         Score      Num Spectra      Num Peptides      High-Qual Peptides      % Coverage       89    3    2    2    7   
   
      Peptides:   
        Query    Observed    Mr(expt)    Mr(calc)    Score    Peptide    Result File   
		    134    610.35    1218.68    1218.55    40    ALQEAEGNMEK    37Rep_4   
		    162    693.57    1385.13    1384.73    49    EGITLQEVLNNR    37RepA_5   
   
      Matching Genes:  
               gi|115251193|emb|CAJ69024.1|  (elongation factor Ts [Clostridium difficile 630]) 
           
  Protein Group 119   
      Expression Quality:  
         Score      Num Spectra      Num Peptides      High-Qual Peptides      % Coverage       88    2    2    2    16   
   
      Peptides:   
        Query    Observed    Mr(expt)    Mr(calc)    Score    Peptide    Result File   
		    96    460.33    918.64    918.52    45    FEILEIR    37Rep_3   
		    384    696.15    2085.44    2084.99    43    VAISFGDLSENAEYDEAKK    37RepA_3   
   
      Matching Genes:  
               gi|115252616|emb|CAJ70459.1|  (transcription elongation factor [Clostridium difficile 630]) 
           
  Protein Group 120   
      Expression Quality:  
         Score      Num Spectra      Num Peptides      High-Qual Peptides      % Coverage       85    3    1    1    9   
   
      Peptides:   
        Query    Observed    Mr(expt)    Mr(calc)    Score    Peptide    Result File   
		    276    797.11    1592.21    1591.86    85    APVSNFAYLIDAIAK    37RepA_3   
   
      Matching Genes:  
               gi|115249067|emb|CAJ66878.1|  (50S ribosomal protein L10 [Clostridium difficile 630]) 
           
  Protein Group 121   
      Expression Quality:  
         Score      Num Spectra      Num Peptides      High-Qual Peptides      % Coverage       85    3    2    1    6   
   
      Peptides:   
        Query    Observed    Mr(expt)    Mr(calc)    Score    Peptide    Result File   
		    185    654.92    1307.83    1307.60    54    AATPDVNSENYK    37Rep_4   
		    39    454.36    906.70    906.48    31    EAYGQVLK    37RepA_4   
   
      Matching Genes:  
               gi|115251376|emb|CAJ69208.1|  (transketolase [Clostridium difficile 630]) 
           
  Protein Group 122   
      Expression Quality:  
         Score      Num Spectra      Num Peptides      High-Qual Peptides      % Coverage       82    1    1    1    5   
   
      Peptides:   
        Query    Observed    Mr(expt)    Mr(calc)    Score    Peptide    Result File   
		    207    607.92    1213.83    1213.67    82    AGENLLSLLER    37Rep_3   
   
      Matching Genes:  
               gi|115249105|emb|CAJ66916.1|  (30S ribosomal protein S4 [Clostridium difficile 630]) 
           
  Protein Group 123   
      Expression Quality:  
         Score      Num Spectra      Num Peptides      High-Qual Peptides      % Coverage       82    1    1    1    6   
   
      Peptides:   
        Query    Observed    Mr(expt)    Mr(calc)    Score    Peptide    Result File   
		    318    876.68    1751.35    1751.85    82    VYVNTNSLHFQSTDK    37Rep_4   
   
      Matching Genes:  
               gi|115252284|emb|CAJ70125.1|  (2,3,4,5-tetrahydropyridine-2,6-dicarboxylate N-succinyltransferase [Clostridium difficile 630]) 
           
  Protein Group 124   
      Expression Quality:  
         Score      Num Spectra      Num Peptides      High-Qual Peptides      % Coverage       80    1    1    1    6   
   
      Peptides:   
        Query    Observed    Mr(expt)    Mr(calc)    Score    Peptide    Result File   
		    302    854.18    1706.35    1705.90    80    AAINSLTQNIATQYAK    37RepA_3   
   
      Matching Genes:  
               gi|115249069|emb|CAJ66880.1|  (NADP-dependent 7-alpha-hydroxysteroid dehydrogenase [Clostridium difficile 630]) 
           
  Protein Group 125   
      Expression Quality:  
         Score      Num Spectra      Num Peptides      High-Qual Peptides      % Coverage       80    2    2    1    10   
   
      Peptides:   
        Query    Observed    Mr(expt)    Mr(calc)    Score    Peptide    Result File   
		    183    437.02    1308.03    1307.69    47    KENIAIQEAHR    37RepA_3   
		    212    458.62    1372.84    1372.66    33    QAQDAIKDEAER    37RepA_3   
   
      Matching Genes:  
               gi|115251194|emb|CAJ69025.1|  (30S ribosomal protein S2 [Clostridium difficile 630]) 
           
  Protein Group 126   
      Expression Quality:  
         Score      Num Spectra      Num Peptides      High-Qual Peptides      % Coverage       77    2    1    1    15   
   
      Peptides:   
        Query    Observed    Mr(expt)    Mr(calc)    Score    Peptide    Result File   
		    299    839.14    1676.27    1675.83    77    VETQGATGIDNELTTK    37RepA_1   
   
      Matching Genes:  
               gi|115252071|emb|CAJ69908.1|  (PTS system, IIb component [Clostridium difficile 630]) 
           
  Protein Group 127   
      Expression Quality:  
         Score      Num Spectra      Num Peptides      High-Qual Peptides      % Coverage       76    2    2    1    4   
   
      Peptides:   
        Query    Observed    Mr(expt)    Mr(calc)    Score    Peptide    Result File   
		    102    627.99    1253.96    1253.81    33    KVTGKPTVIIAK    37RepA_4   
		    68    564.00    1125.99    1125.71    43    VTGKPTVIIAK    37RepA_4   
   
      Matching Genes:  
               gi|115251377|emb|CAJ69209.1|  (transketolase [Clostridium difficile 630]) 
           
  Protein Group 128   
      Expression Quality:  
         Score      Num Spectra      Num Peptides      High-Qual Peptides      % Coverage       75    2    2    1    4   
   
      Peptides:   
        Query    Observed    Mr(expt)    Mr(calc)    Score    Peptide    Result File   
		    204    500.36    1498.07    1497.70    45    IGSGPSSSHTMGPQR    37RepA_5   
		    241    505.58    1513.73    1513.69    30    IGSGPSSSHTMGPQR +Oxidation (M)    37Rep_5   
   
      Matching Genes:  
               gi|115252279|emb|CAJ70120.1|  (L-serine dehydratase [Clostridium difficile 630]) 
           
  Protein Group 129   
      Expression Quality:  
         Score      Num Spectra      Num Peptides      High-Qual Peptides      % Coverage       70    2    2    1    8   
   
      Peptides:   
        Query    Observed    Mr(expt)    Mr(calc)    Score    Peptide    Result File   
		    228    488.27    974.52    975.49    43    GTLDTAGVDK    37Rep_1   
		    124    566.87    1131.72    1131.59    27    GTLDTAGVDKR    37RepA_1   
   
      Matching Genes:  
               gi|115249072|emb|CAJ66883.1|  (30S ribosomal protein S12 [Clostridium difficile 630]) 
           
  Protein Group 130   
      Expression Quality:  
         Score      Num Spectra      Num Peptides      High-Qual Peptides      % Coverage       70    1    1    1    6   
   
      Peptides:   
        Query    Observed    Mr(expt)    Mr(calc)    Score    Peptide    Result File   
		    310    869.17    1736.33    1735.76    70    ADIDYGFAEADTTYGK    37Rep_4   
   
      Matching Genes:  
               gi|115249083|emb|CAJ66894.1|  (30S ribosomal protein S3 [Clostridium difficile 630]) 
           
  Protein Group 131   
      Expression Quality:  
         Score      Num Spectra      Num Peptides      High-Qual Peptides      % Coverage       70    2    1    1    16   
   
      Peptides:   
        Query    Observed    Mr(expt)    Mr(calc)    Score    Peptide    Result File   
		    321    900.16    1798.31    1797.88    70    FIEEIGYYNPISEPK    37RepA_1   
   
      Matching Genes:  
               gi|115250287|emb|CAJ68109.1|  (30S ribosomal protein S16 [Clostridium difficile 630]) 
           
  Protein Group 132   
      Expression Quality:  
         Score      Num Spectra      Num Peptides      High-Qual Peptides      % Coverage       70    1    1    1    4   
   
      Peptides:   
        Query    Observed    Mr(expt)    Mr(calc)    Score    Peptide    Result File   
		    260    779.04    1556.07    1555.76    70    SIQAIDSHTAGEATR    37Rep_4   
   
      Matching Genes:  
               gi|115252294|emb|CAJ70135.1|  (putative proline racemase [Clostridium difficile 630]) 
           
  Protein Group 133   
      Expression Quality:  
         Score      Num Spectra      Num Peptides      High-Qual Peptides      % Coverage       68    2    2    0    37   
   
      Peptides:   
        Query    Observed    Mr(expt)    Mr(calc)    Score    Peptide    Result File   
		    575    505.91    1514.70    1514.77    31    EQVVVKEDNAQTR    37Rep_1   
		    102    522.87    1043.73    1043.56    37    SVIGTTPNQK    37RepA_1   
   
      Matching Genes:  
               gi|115249095|emb|CAJ66906.1|  (50S ribosomal protein L30 [Clostridium difficile 630]) 
           
  Protein Group 134   
      Expression Quality:  
         Score      Num Spectra      Num Peptides      High-Qual Peptides      % Coverage       67    2    1    1    19   
   
      Peptides:   
        Query    Observed    Mr(expt)    Mr(calc)    Score    Peptide    Result File   
		    544    724.96    1447.90    1447.74    67    FQLATGQLENTAR    37Rep_1   
   
      Matching Genes:  
               gi|115249085|emb|CAJ66896.1|  (50S ribosomal protein L29 [Clostridium difficile 630]) 
           
  Protein Group 135   
      Expression Quality:  
         Score      Num Spectra      Num Peptides      High-Qual Peptides      % Coverage       67    2    1    1    12   
   
      Peptides:   
        Query    Observed    Mr(expt)    Mr(calc)    Score    Peptide    Result File   
		    172    636.47    1270.92    1270.69    67    QNINIVDISQK    37RepA_1   
   
      Matching Genes:  
               gi|115251678|emb|CAJ69513.1|  (conserved hypothetical protein [Clostridium difficile 630]) 
           
  Protein Group 136   
      Expression Quality:  
         Score      Num Spectra      Num Peptides      High-Qual Peptides      % Coverage       66    1    1    1    12   
   
      Peptides:   
        Query    Observed    Mr(expt)    Mr(calc)    Score    Peptide    Result File   
		    311    861.62    1721.22    1720.82    66    GYDVSSISTPDLENPK    37RepA_1   
   
      Matching Genes:  
               gi|115249013|emb|CAJ66824.1|  (anti-sigma-B factor (serine-protein kinase) [Clostridium difficile 630]) 
           
  Protein Group 137   
      Expression Quality:  
         Score      Num Spectra      Num Peptides      High-Qual Peptides      % Coverage       66    3    2    0    9   
   
      Peptides:   
        Query    Observed    Mr(expt)    Mr(calc)    Score    Peptide    Result File   
		    51    473.36    944.71    944.39    31    FEEDMFK    37Rep_6   
		    556    1221.39    3661.16    3659.97    35    IEGLEYFLIPTAEVPVTNIHANEILDVAELPIK    37RepA_6   
   
      Matching Genes:  
               gi|115249017|emb|CAJ66828.1|  (seryl-tRNA synthetase [Clostridium difficile 630]) 
              Other Genes Matching Peptide Subset:  
               gi|115251893|emb|CAJ69728.1|  (seryl-tRNA synthetase [Clostridium difficile 630]) 
           
  Protein Group 138   
      Expression Quality:  
         Score      Num Spectra      Num Peptides      High-Qual Peptides      % Coverage       65    1    1    1    7   
   
      Peptides:   
        Query    Observed    Mr(expt)    Mr(calc)    Score    Peptide    Result File   
		    360    762.55    1523.08    1522.75    65    VDSVEGYTVGQEIK    37Rep_3   
   
      Matching Genes:  
               gi|115249077|emb|CAJ66888.1|  (50S ribosomal protein L3 [Clostridium difficile 630]) 
           
  Protein Group 139   
      Expression Quality:  
         Score      Num Spectra      Num Peptides      High-Qual Peptides      % Coverage       65    1    1    1    7   
   
      Peptides:   
        Query    Observed    Mr(expt)    Mr(calc)    Score    Peptide    Result File   
		    404    822.70    1643.38    1642.86    65    GFIYTLTESKPYPK    37Rep_3   
   
      Matching Genes:  
               gi|115252732|emb|CAJ70576.1|  (conserved hypothetical protein [Clostridium difficile 630]) 
           
  Protein Group 140   
      Expression Quality:  
         Score      Num Spectra      Num Peptides      High-Qual Peptides      % Coverage       64    2    2    0    10   
   
      Peptides:   
        Query    Observed    Mr(expt)    Mr(calc)    Score    Peptide    Result File   
		    223    780.09    1558.17    1557.73    37    TIGVMYNTSEVNSK +Oxidation (M)    37RepA_5   
		    378    1041.29    2080.57    2080.03    27    TLEKPGTNVSGTSDFVSVDK    37Rep_5   
   
      Matching Genes:  
               gi|115249887|emb|CAJ67706.1|  (ABC transporter, substrate-binding lipoprotein [Clostridium difficile 630]) 
           
  Protein Group 141   
      Expression Quality:  
         Score      Num Spectra      Num Peptides      High-Qual Peptides      % Coverage       64    2    2    0    42   
   
      Peptides:   
        Query    Observed    Mr(expt)    Mr(calc)    Score    Peptide    Result File   
		    352    632.02    1893.05    1892.79    28    MEATGFVSCPQCHEPK +Oxidation (M)    37RepA_1   
		    132    581.37    1160.73    1160.46    36    VCPDCGYYK    37RepA_1   
   
      Matching Genes:  
               gi|115250209|emb|CAJ68030.1|  (50S ribosomal protein L32 [Clostridium difficile 630]) 
           
  Protein Group 142   
      Expression Quality:  
         Score      Num Spectra      Num Peptides      High-Qual Peptides      % Coverage       63    1    1    1    9   
   
      Peptides:   
        Query    Observed    Mr(expt)    Mr(calc)    Score    Peptide    Result File   
		    540    745.95    2234.82    2234.16    63    NIEGVQTALVNTMNVYDILK    37Rep_3   
   
      Matching Genes:  
               gi|115249078|emb|CAJ66889.1|  (50S ribosomal protein L4 [Clostridium difficile 630]) 
           
  Protein Group 143   
      Expression Quality:  
         Score      Num Spectra      Num Peptides      High-Qual Peptides      % Coverage       63    2    2    0    14   
   
      Peptides:   
        Query    Observed    Mr(expt)    Mr(calc)    Score    Peptide    Result File   
		    100    534.41    1066.80    1066.51    31    FAPDYNVNK    37RepA_2   
		    250    736.60    1471.18    1470.80    32    IYSSLYLEDLKK    37RepA_2   
   
      Matching Genes:  
               gi|115250371|emb|CAJ68193.1|  (MarR-family transcriptional regulator [Clostridium difficile 630]) 
           
  Protein Group 144   
      Expression Quality:  
         Score      Num Spectra      Num Peptides      High-Qual Peptides      % Coverage       63    2    1    1    9   
   
      Peptides:   
        Query    Observed    Mr(expt)    Mr(calc)    Score    Peptide    Result File   
		    427    760.00    1517.98    1517.81    63    SPVDLIPFVEAGFK    37Rep_2   
   
      Matching Genes:  
               gi|115252122|emb|CAJ69960.1|  (PTS system, IIb component [Clostridium difficile 630]) 
           
  Protein Group 145   
      Expression Quality:  
         Score      Num Spectra      Num Peptides      High-Qual Peptides      % Coverage       63    2    1    1    7   
   
      Peptides:   
        Query    Observed    Mr(expt)    Mr(calc)    Score    Peptide    Result File   
		    186    652.54    1303.06    1302.59    63    SAEFCANYITK    37RepA_2   
   
      Matching Genes:  
               gi|115252727|emb|CAJ70571.1|  (single-strand binding protein [Clostridium difficile 630]) 
           
  Protein Group 146   
      Expression Quality:  
         Score      Num Spectra      Num Peptides      High-Qual Peptides      % Coverage       62    1    1    1    4   
   
      Peptides:   
        Query    Observed    Mr(expt)    Mr(calc)    Score    Peptide    Result File   
		    474    868.57    1735.13    1734.89    62    VITGLATSDDDSSITIK    37Rep_2   
   
      Matching Genes:  
               gi|115251108|emb|CAJ68939.1|  (aspartokinase [Clostridium difficile 630]) 
           
  Protein Group 147   
      Expression Quality:  
         Score      Num Spectra      Num Peptides      High-Qual Peptides      % Coverage       62    2    2    0    10   
   
      Peptides:   
        Query    Observed    Mr(expt)    Mr(calc)    Score    Peptide    Result File   
		    182    502.28    1002.55    1002.49    35    DLDVDIEGK    37Rep_2   
		    185    504.25    1006.48    1006.50    27    MLTEEQIK +Oxidation (M)    37Rep_2   
   
      Matching Genes:  
               gi|115251742|emb|CAJ69577.1|  (putative phosphoribosyltransferase [Clostridium difficile 630]) 
           
  Protein Group 148   
      Expression Quality:  
         Score      Num Spectra      Num Peptides      High-Qual Peptides      % Coverage       62    1    1    1    3   
   
      Peptides:   
        Query    Observed    Mr(expt)    Mr(calc)    Score    Peptide    Result File   
		    188    778.10    1554.19    1553.78    62    GIPVSIGTDGAPSNNR    37RepA_6   
   
      Matching Genes:  
               gi|115251756|emb|CAJ69591.1|  (putative amidohydrolas [Clostridium difficile 630]) 
           
  Protein Group 149   
      Expression Quality:  
         Score      Num Spectra      Num Peptides      High-Qual Peptides      % Coverage       62    3    1    1    8   
   
      Peptides:   
        Query    Observed    Mr(expt)    Mr(calc)    Score    Peptide    Result File   
		    503    1022.31    2042.61    2042.21    62    AIEEAGIPTIIIAALPPVVR    37Rep_3   
   
      Matching Genes:  
               gi|115252298|emb|CAJ70139.1|  (proline reductase [Clostridium difficile 630]) 
           
  Protein Group 150   
      Expression Quality:  
         Score      Num Spectra      Num Peptides      High-Qual Peptides      % Coverage       62    2    1    1    22   
   
      Peptides:   
        Query    Observed    Mr(expt)    Mr(calc)    Score    Peptide    Result File   
		    564    745.43    1488.85    1488.71    62    EAEEGCPVSAITVK    37Rep_1   
   
      Matching Genes:  
               gi|115252670|emb|CAJ70513.1|  (ferredoxin [Clostridium difficile 630]) 
           
  Protein Group 151   
      Expression Quality:  
         Score      Num Spectra      Num Peptides      High-Qual Peptides      % Coverage       59    2    1    1    5   
   
      Peptides:   
        Query    Observed    Mr(expt)    Mr(calc)    Score    Peptide    Result File   
		    139    503.80    1005.59    1005.51    59    VPGATYAEAK    37Rep_3   
   
      Matching Genes:  
               gi|115251487|emb|CAJ69320.1|  (conserved hypothetical protein [Clostridium difficile 630]) 
           
  Protein Group 152   
      Expression Quality:  
         Score      Num Spectra      Num Peptides      High-Qual Peptides      % Coverage       57    2    2    0    4   
   
      Peptides:   
        Query    Observed    Mr(expt)    Mr(calc)    Score    Peptide    Result File   
		    178    755.14    1508.27    1507.78    30    FGHLHQSVDDLIK    37RepA_6   
		    33    462.39    922.76    922.49    27    FPYEIVR    37RepA_6   
   
      Matching Genes:  
               gi|115251396|emb|CAJ69228.1|  (gamma-aminobutyrate metabolism dehydratase/isomerase [includes: 4-hydroxybutyryl-coa dehydratase; vinylacetyl-coa-delta-isomerase] [Clostridium difficile 630]) 
           
  Protein Group 153   
      Expression Quality:  
         Score      Num Spectra      Num Peptides      High-Qual Peptides      % Coverage       56    2    1    1    10   
   
      Peptides:   
        Query    Observed    Mr(expt)    Mr(calc)    Score    Peptide    Result File   
		    504    693.43    1384.85    1385.70    56    ELVPNTTDAAVEK    37Rep_1   
   
      Matching Genes:  
               gi|115249844|emb|CAJ67661.1|  (rubredoxin oxidoreductase (desulfoferrodoxin) [Clostridium difficile 630]) 
           
  Protein Group 154   
      Expression Quality:  
         Score      Num Spectra      Num Peptides      High-Qual Peptides      % Coverage       55    2    1    1    3   
   
      Peptides:   
        Query    Observed    Mr(expt)    Mr(calc)    Score    Peptide    Result File   
		    214    742.05    1482.08    1481.70    55    SHEEGISPEEVAAK    37Rep_6   
   
      Matching Genes:  
               gi|115249055|emb|CAJ66866.1|  (cysteinyl-tRNA synthetase [Clostridium difficile 630]) 
           
  Protein Group 155   
      Expression Quality:  
         Score      Num Spectra      Num Peptides      High-Qual Peptides      % Coverage       55    2    1    1    8   
   
      Peptides:   
        Query    Observed    Mr(expt)    Mr(calc)    Score    Peptide    Result File   
		    299    849.31    1696.60    1696.03    55    VALVGGGIGVAPLYLVAK    37RepA_3   
   
      Matching Genes:  
               gi|115249195|emb|CAJ67007.1|  (dihydroorotate dehydrogenase electron transfer subunit [Clostridium difficile 630]) 
           
  Protein Group 156   
      Expression Quality:  
         Score      Num Spectra      Num Peptides      High-Qual Peptides      % Coverage       54    1    1    1    7   
   
      Peptides:   
        Query    Observed    Mr(expt)    Mr(calc)    Score    Peptide    Result File   
		    121    556.95    1111.88    1111.62    54    ILVPIDGTER    37RepA_1   
   
      Matching Genes:  
               gi|115249829|emb|CAJ67646.1|  (putative universal stress protein [Clostridium difficile 630]) 
           
  Protein Group 157   
      Expression Quality:  
         Score      Num Spectra      Num Peptides      High-Qual Peptides      % Coverage       54    1    1    1    13   
   
      Peptides:   
        Query    Observed    Mr(expt)    Mr(calc)    Score    Peptide    Result File   
		    178    456.32    910.62    910.50    54    GPQAANVVR    37Rep_1   
   
      Matching Genes:  
               gi|115250391|emb|CAJ68213.1|  (putative cold shock protein [Clostridium difficile 630]) 
           
  Protein Group 158   
      Expression Quality:  
         Score      Num Spectra      Num Peptides      High-Qual Peptides      % Coverage       54    1    1    1    1   
   
      Peptides:   
        Query    Observed    Mr(expt)    Mr(calc)    Score    Peptide    Result File   
		    85    561.37    1120.72    1120.61    54    FVGSTLEQLK    37Rep_7   
   
      Matching Genes:  
               gi|115251485|emb|CAJ69318.1|  (glycyl-tRNA synthetase beta chain [Clostridium difficile 630]) 
           
  Protein Group 159   
      Expression Quality:  
         Score      Num Spectra      Num Peptides      High-Qual Peptides      % Coverage       54    2    1    1    7   
   
      Peptides:   
        Query    Observed    Mr(expt)    Mr(calc)    Score    Peptide    Result File   
		    353    748.05    1494.08    1493.85    54    AGVVLNPATPVDTIK    37Rep_3   
   
      Matching Genes:  
               gi|115251631|emb|CAJ69464.1|  (ribulose-phosphate 3-epimerase [Clostridium difficile 630]) 
           
  Protein Group 160   
      Expression Quality:  
         Score      Num Spectra      Num Peptides      High-Qual Peptides      % Coverage       54    2    1    1    2   
   
      Peptides:   
        Query    Observed    Mr(expt)    Mr(calc)    Score    Peptide    Result File   
		    273    911.26    1820.50    1819.88    54    SLYSSSEEPVTPPSNVK    37Rep_7   
   
      Matching Genes:  
               gi|115251820|emb|CAJ69655.1|  (cell surface protein [Clostridium difficile 630]) 
           
  Protein Group 161   
      Expression Quality:  
         Score      Num Spectra      Num Peptides      High-Qual Peptides      % Coverage       54    1    1    1    19   
   
      Peptides:   
        Query    Observed    Mr(expt)    Mr(calc)    Score    Peptide    Result File   
		    492    686.85    1371.68    1371.59    54    CACGNTFVAGSTK    37Rep_1   
   
      Matching Genes:  
               gi|115252547|emb|CAJ70390.1|  (50S ribosomal protein L31 [Clostridium difficile 630]) 
           
  Protein Group 162   
      Expression Quality:  
         Score      Num Spectra      Num Peptides      High-Qual Peptides      % Coverage       53    2    1    1    4   
   
      Peptides:   
        Query    Observed    Mr(expt)    Mr(calc)    Score    Peptide    Result File   
		    182    504.36    1510.05    1509.83    53    ISSKPIIATHSNSR    37RepA_4   
   
      Matching Genes:  
               gi|115252633|emb|CAJ70476.1|  (probable dipeptidase [Clostridium difficile 630]) 
           
  Protein Group 163   
      Expression Quality:  
         Score      Num Spectra      Num Peptides      High-Qual Peptides      % Coverage       52    2    1    1    7   
   
      Peptides:   
        Query    Observed    Mr(expt)    Mr(calc)    Score    Peptide    Result File   
		    271    783.05    1564.09    1563.75    52    TGAIREESFNPSEK    37RepA_3   
   
      Matching Genes:  
               gi|115250279|emb|CAJ68101.1|  (elongation factor P [Clostridium difficile 630]) 
           
  Protein Group 164   
      Expression Quality:  
         Score      Num Spectra      Num Peptides      High-Qual Peptides      % Coverage       52    2    1    1    5   
   
      Peptides:   
        Query    Observed    Mr(expt)    Mr(calc)    Score    Peptide    Result File   
		    247    638.41    1274.81    1274.60    52    NQDVSDEDILK    37Rep_3   
   
      Matching Genes:  
               gi|115251628|emb|CAJ69461.1|  (putative nitroreductase [Clostridium difficile 630]) 
           
  Protein Group 165   
      Expression Quality:  
         Score      Num Spectra      Num Peptides      High-Qual Peptides      % Coverage       51    1    1    1    7   
   
      Peptides:   
        Query    Observed    Mr(expt)    Mr(calc)    Score    Peptide    Result File   
		    223    483.29    964.56    964.50    51    VINTSEFR    37Rep_1   
   
      Matching Genes:  
               gi|115250733|emb|CAJ68557.1|  (thioredoxin [Clostridium difficile 630]) 
           
  Protein Group 166   
      Expression Quality:  
         Score      Num Spectra      Num Peptides      High-Qual Peptides      % Coverage       50    1    1    1    8   
   
      Peptides:   
        Query    Observed    Mr(expt)    Mr(calc)    Score    Peptide    Result File   
		    88    478.38    954.74    954.55    50    KYTFVVAK    37RepA_1   
   
      Matching Genes:  
               gi|115249079|emb|CAJ66890.1|  (50S ribosomal protein L23 [Clostridium difficile 630]) 
           
  Protein Group 167   
      Expression Quality:  
         Score      Num Spectra      Num Peptides      High-Qual Peptides      % Coverage       50    1    1    1    9   
   
      Peptides:   
        Query    Observed    Mr(expt)    Mr(calc)    Score    Peptide    Result File   
		    311    580.42    1158.83    1158.62    50    KAGQICDLVR    37Rep_1   
   
      Matching Genes:  
               gi|115249082|emb|CAJ66893.1|  (50S ribosomal protein L22 [Clostridium difficile 630]) 
           
  Protein Group 168   
      Expression Quality:  
         Score      Num Spectra      Num Peptides      High-Qual Peptides      % Coverage       49    1    1    1    7   
   
      Peptides:   
        Query    Observed    Mr(expt)    Mr(calc)    Score    Peptide    Result File   
		    350    941.78    1881.54    1880.88    49    TPLVIATTGYNDDEMNK    37RepA_3   
   
      Matching Genes:  
               gi|115252283|emb|CAJ70124.1|  (dihydrodipicolinate reductase [Clostridium difficile 630]) 
           
  Protein Group 169   
      Expression Quality:  
         Score      Num Spectra      Num Peptides      High-Qual Peptides      % Coverage       48    2    1    1    1   
   
      Peptides:   
        Query    Observed    Mr(expt)    Mr(calc)    Score    Peptide    Result File   
		    84    561.33    1120.65    1120.58    48    KMIEENTIK +Oxidation (M)    37Rep_7   
   
      Matching Genes:  
               gi|115250692|emb|CAJ68516.1|  (putative signaling protein [Clostridium difficile 630]) 
           
  Protein Group 170   
      Expression Quality:  
         Score      Num Spectra      Num Peptides      High-Qual Peptides      % Coverage       48    1    1    1    22   
   
      Peptides:   
        Query    Observed    Mr(expt)    Mr(calc)    Score    Peptide    Result File   
		    173    636.48    1270.94    1270.70    48    LIDEALEDLIK    37RepA_1   
   
      Matching Genes:  
               gi|110666931|ref|YP_659585.1|  (hypothetical protein CDP09 [Clostridium difficile 630]) 
           
  Protein Group 171   
      Expression Quality:  
         Score      Num Spectra      Num Peptides      High-Qual Peptides      % Coverage       47    1    1    1    3   
   
      Peptides:   
        Query    Observed    Mr(expt)    Mr(calc)    Score    Peptide    Result File   
		    417    1158.60    2315.19    2315.23    47    IATLIPIDGNDENEYLLLATK    37Rep_7   
   
      Matching Genes:  
               gi|115249009|emb|CAJ66820.1|  (DNA gyrase subunit A [Clostridium difficile 630]) 
           
  Protein Group 172   
      Expression Quality:  
         Score      Num Spectra      Num Peptides      High-Qual Peptides      % Coverage       46    2    1    1    17   
   
      Peptides:   
        Query    Observed    Mr(expt)    Mr(calc)    Score    Peptide    Result File   
		    568    1148.13    2294.25    2293.12    46    LADVAELDTLLSDKEYEAGLE    37Rep_2   
   
      Matching Genes:  
               gi|115249746|emb|CAJ67563.1|  (putative glycine cleavage system H protein [Clostridium difficile 630]) 
           
  Protein Group 173   
      Expression Quality:  
         Score      Num Spectra      Num Peptides      High-Qual Peptides      % Coverage       46    1    1    1    9   
   
      Peptides:   
        Query    Observed    Mr(expt)    Mr(calc)    Score    Peptide    Result File   
		    240    716.09    1430.16    1429.74    46    VTVDENTIGQINK    37RepA_2   
   
      Matching Genes:  
               gi|115251795|emb|CAJ69630.1|  (putative D-tyrosyl-tRNA protein [Clostridium difficile 630]) 
           
  Protein Group 174   
      Expression Quality:  
         Score      Num Spectra      Num Peptides      High-Qual Peptides      % Coverage       44    1    1    1    2   
   
      Peptides:   
        Query    Observed    Mr(expt)    Mr(calc)    Score    Peptide    Result File   
		    115    637.37    1272.72    1272.63    44    LPEPQFEGQTK    37RepA_7   
   
      Matching Genes:  
               gi|115249008|emb|CAJ66819.1|  (DNA gyrase subunit B [Clostridium difficile 630]) 
           
  Protein Group 175   
      Expression Quality:  
         Score      Num Spectra      Num Peptides      High-Qual Peptides      % Coverage       44    2    1    1    4   
   
      Peptides:   
        Query    Observed    Mr(expt)    Mr(calc)    Score    Peptide    Result File   
		    115    575.89    1149.77    1149.60    44    LISHTPEPEK    37Rep_4   
   
      Matching Genes:  
               gi|115249057|emb|CAJ66868.1|  (putative thymidylate synthase [Clostridium difficile 630]) 
           
  Protein Group 176   
      Expression Quality:  
         Score      Num Spectra      Num Peptides      High-Qual Peptides      % Coverage       43    1    1    1    3   
   
      Peptides:   
        Query    Observed    Mr(expt)    Mr(calc)    Score    Peptide    Result File   
		    17    425.89    849.76    849.47    43    VVVTGYGR    37RepA_4   
   
      Matching Genes:  
               gi|115249402|emb|CAJ67217.1|  (activator of 2-hydroxyisocaproyl-CoA dehydratase [Clostridium difficile 630]) 
           
  Protein Group 177   
      Expression Quality:  
         Score      Num Spectra      Num Peptides      High-Qual Peptides      % Coverage       43    1    1    1    2   
   
      Peptides:   
        Query    Observed    Mr(expt)    Mr(calc)    Score    Peptide    Result File   
		    226    800.66    1599.30    1598.76    43    TYVSAYHSTNLSEK    37RepA_7   
   
      Matching Genes:  
               gi|115251837|emb|CAJ69672.1|  (cell surface protein [Clostridium difficile 630]) 
             
