# Supplementary material for: Semiquantitative Analysis of Clinical Heat Stress in Clostridium difficile Strain 630 Using a GeLC/MS Workflow with emPAI Quantitation
Source: PLoS One. 2014 Feb 24;9(2):e88960. doi: 10.1371/journal.pone.0088960 (PMC3933415; doi:10.1371/journal.pone.0088960)
Supplement: File S2 — PROVALT output html file from 37°C pseudoreplicate single lane GeLC/MS, single injection, 202 proteins. (HTML) [file pone.0088960.s002.html]

   Complete output   Complete output    
 
   
  Protein Group 1   
      Expression Quality:  
         Score      Num Spectra      Num Peptides      High-Qual Peptides      % Coverage       2494    101    42    34    40   
   
      Peptides:   
        Query    Observed    Mr(expt)    Mr(calc)    Score    Peptide    Result File   
		    369    701.13    2100.35    2100.04    49    ALASQALSIFGDHQDVMAAR    37A_7   
		    382    1059.27    2116.52    2116.04    50    ALASQALSIFGDHQDVMAAR +Oxidation (M)    37_8   
		    281    891.98    1781.95    1781.83    70    ATYLIDEADYIACHK    37A_8   
		    105    528.88    1055.74    1056.54    37    ENAPMIIGGR    37_8   
		    258    767.03    1532.04    1531.75    72    EPGSTGEPLYLDVR    37_8   
		    2    403.26    804.51    804.41    32    FGDTPIR    37A_8   
		    55    471.29    940.56    940.50    33    FYTVNAVK    37A_7   
		    472    1335.72    2669.42    2669.17    60    GPSWANSLFEDNAEYGFGMYTAVK +Oxidation (M)    37A_8   
		    312    756.01    1510.01    1509.72    60    GTAQNPDIYFQTR    37_7   
		    226    702.00    1401.99    1401.67    44    HFLDAMPSTVER    37_8   
		    235    709.98    1417.95    1417.67    44    HFLDAMPSTVER +Oxidation (M)    37_8   
		    226    758.40    1514.78    1514.67    70    HSLFDYYGAEDAK    37A_8   
		    551    1168.80    2335.58    2335.01    75    IQVSPLDCTGCGNCADICPAK    37_7   
		    362    1020.77    2039.53    2038.93    64    IVDAMTELVSMDICEDAK    37_8   
		    370    1036.72    2071.42    2070.92    73    IVDAMTELVSMDICEDAK +2 Oxidation (M)    37_8   
		    367    1028.82    2055.62    2054.92    90    IVDAMTELVSMDICEDAK +Oxidation (M)    37_8   
		    301    938.55    1875.09    1874.99    94    IVNMNYAAVDAGINALVK    37A_8   
		    415    946.70    1891.39    1890.99    76    IVNMNYAAVDAGINALVK +Oxidation (M)    37_7   
		    249    822.53    1643.05    1642.76    82    KHSLFDYYGAEDAK    37A_8   
		    285    663.94    1325.88    1325.71    47    LAEIIPEEDAVK    37A_1   
		    131    528.89    1055.77    1055.57    59    LGQEIGLGNR    37_7   
		    196    698.36    1394.71    1394.71    34    LPFIHFFDGFR    37A_8   
		    566    1202.88    2403.74    2403.22    63    NGFTVGIVDDVTNTSLTPSEPIK    37_7   
		    583    1305.00    3911.98    3911.85    43    NILRPMTAQEGNNLPVSTFNGIEDGTFPCGTAAYEK    37A_8   
		    73    461.31    920.61    920.46    42    NPFTLDSK    37_8   
		    401    1095.20    2188.38    2188.96    71    QPLMEFSGACAGCGETAYIK    37_8   
		    406    736.16    2205.47    2204.95    69    QPLMEFSGACAGCGETAYIK +Oxidation (M)    37_8   
		    73    529.77    1057.53    1057.53    63    SGGITMSHLR    37A_8   
		    81    537.81    1073.61    1073.53    45    SGGITMSHLR +Oxidation (M)    37A_8   
		    373    881.65    1761.29    1760.89    115    TKEPGSTGEPLYLDVR    37_7   
		    340    1055.14    2108.26    2107.99    83    TVANEAQAVACGYWHLYR    37A_8   
		    193    688.97    1375.93    1375.70    39    TVFDNLVSEQPK    37A_8   
		    232    517.97    1550.90    1550.86    34    VAGELLPGVFHVSAR    37A_8   
		    140    545.34    1088.67    1088.55    37    VDVMPANTVK +Oxidation (M)    37_7   
		    451    1285.77    2569.52    2569.27    96    VELLENEDYASLLNFEAVQAFR    37A_8   
		    282    815.53    1629.05    1628.77    62    VEVPASWENAVDADK    37_8   
		    178    647.44    1292.87    1292.67    28    VHLYRPFSMK +Oxidation (M)    37_8   
		    307    953.67    1905.33    1904.89    55    VVELLEKPACDCTDEK    37A_8   
		    130    525.82    1049.63    1049.55    49    VVTQLYGDR    37_7   
		    191    685.84    1369.66    1369.58    69    YAQAYFDYDSK    37A_8   
		    223    749.89    1497.77    1497.68    52    YAQAYFDYDSKK    37A_8   
		    231    663.94    1325.87    1324.70    64    YYQNIVGIVEK    37_7   
   
      Matching Genes:  
               gi|115251733|emb|CAJ69568.1|  (pyruvate-flavodoxin oxidoreductase [Clostridium difficile 630]) 
           
  Protein Group 2   
      Expression Quality:  
         Score      Num Spectra      Num Peptides      High-Qual Peptides      % Coverage       1442    53    22    20    42   
   
      Peptides:   
        Query    Observed    Mr(expt)    Mr(calc)    Score    Peptide    Result File   
		    457    680.76    2039.24    2038.93    92    AAQEQQAAQGAEQAQDNGPK    37_7   
		    662    1053.85    3158.54    3157.42    39    AAQEQQAAQGAEQAQDNGPKDDNVVDADFK    37_7   
		    302    927.06    1852.11    1851.80    67    DDNVVDADFKEVDEDK    37A_7   
		    257    681.42    1360.83    1360.65    62    DNQDATAEELKK    37_7   
		    484    1065.30    2128.58    2128.03    82    EKIEAFNQAESTIYQTEK    37_7   
		    567    1204.92    2407.83    2407.19    92    ELSSTMSSNINLPFITATAEGPK    37_7   
		    575    1212.87    2423.72    2423.19    57    ELSSTMSSNINLPFITATAEGPK +Oxidation (M)    37_7   
		    100    484.34    966.66    966.52    47    HLNIDLSR    37_7   
		    410    936.67    1871.32    1870.89    95    IEAFNQAESTIYQTEK    37_7   
		    265    839.66    1677.31    1676.84    61    IINEPTAAALAYGMDK    37A_7   
		    366    847.61    1693.21    1692.84    60    IINEPTAAALAYGMDK +Oxidation (M)    37_7   
		    59    477.88    953.75    953.55    46    IPAVQEAVK    37A_7   
		    138    541.90    1081.79    1081.65    29    IPAVQEAVKK    37_7   
		    178    617.89    1233.76    1233.61    66    ISSGEKEDIEK    37_7   
		    413    946.17    1890.33    1889.96    109    ITITSNTNLSEAEIEQK    37_7   
		    639    978.40    2932.16    2931.41    63    SDAESYLGQTVTEAVITVPAYFTDAQR    37_7   
		    588    1250.81    2499.60    2499.24    106    SQIFSTAADNQTAVDIHVLQGER    37_7   
		    340    796.16    1590.31    1589.87    43    SYTPQEISAIILQK    37_7   
		    435    1187.87    2373.72    2373.20    85    TALQDAGLSTGDIDDVLLVGGSTR    37A_7   
		    70    445.29    888.56    888.46    54    TLNELGDK    37_7   
		    82    525.83    1049.65    1049.58    45    TTPSVVAFTK    37A_7   
		    119    506.79    1011.57    1011.47    42    VSQEMYQK    37_7   
   
      Matching Genes:  
               gi|115251515|emb|CAJ69348.1|  (chaperone protein [Clostridium difficile 630]) 
           
  Protein Group 3   
      Expression Quality:  
         Score      Num Spectra      Num Peptides      High-Qual Peptides      % Coverage       1347    39    25    21    44   
   
      Peptides:   
        Query    Observed    Mr(expt)    Mr(calc)    Score    Peptide    Result File   
		    492    1086.38    2170.74    2170.04    59    ATYTMIFDHYEQVPASVAK    37_7   
		    497    1094.32    2186.62    2186.04    65    ATYTMIFDHYEQVPASVAK +Oxidation (M)    37_7   
		    566    1297.60    3889.77    3888.85    28    DTTTGDTLCDPANPIILESMEFPEPVISVAIEPSSK +Oxidation (M)    37A_7   
		    259    681.98    1361.95    1361.74    60    EDSFIGIIDLLK    37_7   
		    172    679.43    1356.85    1356.64    64    GGVEPQSENVWR    37A_7   
		    155    580.35    1158.68    1158.55    53    GILADGEEAER    37_7   
		    234    783.08    1564.15    1563.72    53    HSSDEEPFSALAFK    37A_7   
		    449    808.46    2422.35    2422.02    30    IGETHEGASQMDWMEQEKER +2 Oxidation (M)    37A_7   
		    110    604.38    1206.74    1206.64    42    ILFYTGQTHK    37A_7   
		    85    542.34    1082.66    1082.57    38    ILQMHANTR    37A_7   
		    75    512.28    1022.54    1022.51    39    IMTDPFVGK +Oxidation (M)    37A_7   
		    124    625.39    1248.77    1248.62    71    LAEEDPTFTVK    37A_7   
		    208    741.49    1480.97    1480.81    50    LNSNAVPMQLPIGK    37A_7   
		    307    749.52    1497.02    1496.80    49    LNSNAVPMQLPIGK +Oxidation (M)    37_7   
		    281    878.53    1755.05    1754.80    47    LVESVAETDEELMMK +2 Oxidation (M)    37A_7   
		    74    510.82    1019.63    1019.50    42    QAETYGVPR    37A_7   
		    592    835.00    2501.98    2501.23    83    SGAQVINAFVPLSEMFGYSTDLR    37_7   
		    595    1259.92    2517.83    2517.22    54    SGAQVINAFVPLSEMFGYSTDLR +Oxidation (M)    37_7   
		    157    587.85    1173.69    1173.57    55    VAPQEPGEGYK    37_7   
		    528    1129.79    2257.56    2256.99    41    VEVVTPEDYMGDVMGDLNSR +2 Oxidation (M)    37_7   
		    521    1121.81    2241.61    2240.99    42    VEVVTPEDYMGDVMGDLNSR +Oxidation (M)    37_7   
		    60    480.87    959.72    959.52    45    VGAPQVAYR    37A_7   
		    192    624.47    1246.92    1246.69    71    VYAGDIAAAVGLK    37_7   
		    376    895.15    1788.29    1787.89    101    VYSGTLESGSYVLNATK    37_7   
		    230    776.55    1551.09    1550.77    65    YLEGEELTIDELK    37A_7   
   
      Matching Genes:  
               gi|115249074|emb|CAJ66885.1|  (translation elongation factor G [Clostridium difficile 630]) 
           
  Protein Group 4   
      Expression Quality:  
         Score      Num Spectra      Num Peptides      High-Qual Peptides      % Coverage       1177    70    19    17    46   
   
      Peptides:   
        Query    Observed    Mr(expt)    Mr(calc)    Score    Peptide    Result File   
		    79    498.28    994.55    994.52    59    AEAHIQAGAK    37_5   
		    86    505.83    1009.65    1009.65    63    AIGLVIPSLK    37A_4   
		    128    619.91    1237.81    1237.64    77    DKAEAHIQAGAK    37_5   
		    66    468.26    934.50    934.48    60    FNGEIEVK    37A_4   
		    584    808.91    2423.70    2423.15    62    GLMTTIHAYTNDQNTLDGPHPK    37_6   
		    502    814.10    2439.28    2439.15    36    GLMTTIHAYTNDQNTLDGPHPK +Oxidation (M)    37A_4   
		    255    649.98    1297.94    1297.76    67    KVVISAPATGDLK    37_4   
		    23    430.28    858.54    858.48    30    MLAHLFK    37A_5   
		    474    1163.34    2324.67    2324.10    98    MMEQQDKFEVVAINDLTDAK    37_5   
		    582    1179.31    2356.61    2356.09    73    MMEQQDKFEVVAINDLTDAK +2 Oxidation (M)    37_4   
		    476    1171.28    2340.55    2340.10    84    MMEQQDKFEVVAINDLTDAK +Oxidation (M)    37_5   
		    211    659.86    1317.70    1317.66    61    NVTVEEINAAMK    37A_4   
		    281    667.94    1333.86    1333.65    57    NVTVEEINAAMK +Oxidation (M)    37_4   
		    109    556.44    1110.87    1110.61    55    TLGYFAQLAK    37A_4   
		    177    589.88    1177.75    1177.63    64    VLNDKYGIEK    37_4   
		    381    887.18    1772.34    1771.98    62    VPVVTGSITELVCTLGK    37A_2   
		    174    585.90    1169.79    1169.67    58    VVISAPATGDLK    37_4   
		    481    1045.68    2089.34    2089.98    61    VVSWYDNEMSYTSQLIR    37_6   
		    445    1054.23    2106.44    2105.97    50    VVSWYDNEMSYTSQLIR +Oxidation (M)    37A_4   
   
      Matching Genes:  
               gi|115252231|emb|CAJ70071.1|  (glyceraldehyde-3-phosphate dehydrogenase 2 [Clostridium difficile 630]) 
           
  Protein Group 5   
      Expression Quality:  
         Score      Num Spectra      Num Peptides      High-Qual Peptides      % Coverage       1141    43    20    17    45   
   
      Peptides:   
        Query    Observed    Mr(expt)    Mr(calc)    Score    Peptide    Result File   
		    341    643.50    1927.48    1927.01    53    DKYPGLIFSQILGYGEK    37_5   
		    51    462.81    923.61    923.51    47    EGVEILHK    37A_5   
		    202    697.95    1393.89    1393.65    44    ENPNSPLMTTYK    37_5   
		    206    705.95    1409.88    1409.65    32    ENPNSPLMTTYK +Oxidation (M)    37_5   
		    83    511.31    1020.61    1020.49    66    GGVSQSVMEK    37_5   
		    92    549.86    1097.71    1097.57    29    IEPIEGDGIR    37_5   
		    519    1279.29    2556.57    2556.15    74    IQSCEDLLDDEQAWANDFLFK    37_5   
		    69    495.31    988.61    988.53    60    IVGEAMLEK    37A_5   
		    297    739.10    1476.18    1475.80    43    LLSEADIFVTNVR    37_6   
		    108    609.91    1217.80    1217.61    50    MLGDWGAEVIK    37_5   
		    112    617.93    1233.85    1233.61    50    MLGDWGAEVIK +Oxidation (M)    37A_5   
		    97    570.36    1138.71    1138.63    36    SKEGVEILHK    37_5   
		    250    779.01    1556.01    1555.75    58    SLGYDEEKINNFK    37_5   
		    348    997.65    1993.28    1992.84    77    SPASDDENPMFELENGNK    37A_5   
		    354    1005.61    2009.20    2008.83    70    SPASDDENPMFELENGNK +Oxidation (M)    37A_5   
		    387    1061.70    2121.39    2120.93    91    SPASDDENPMFELENGNKK    37A_5   
		    399    1069.71    2137.40    2136.93    66    SPASDDENPMFELENGNKK +Oxidation (M)    37A_5   
		    382    1053.73    2105.45    2105.02    73    TLDEWSALLEEADLPFEK    37A_6   
		    85    555.37    1108.72    1108.62    66    VGQHTVEVLK    37A_5   
		    182    695.47    1388.92    1388.78    56    WIQLALIQYNK    37A_5   
   
      Matching Genes:  
               gi|115249401|emb|CAJ67216.1|  (isocaprenoyl-CoA:2-hydroxyisocaproate CoA-transferase [Clostridium difficile 630]) 
           
  Protein Group 6   
      Expression Quality:  
         Score      Num Spectra      Num Peptides      High-Qual Peptides      % Coverage       965    30    17    12    25   
   
      Peptides:   
        Query    Observed    Mr(expt)    Mr(calc)    Score    Peptide    Result File   
		    168    462.26    922.50    923.42    36    EFGVEESK    37_2   
		    133    533.83    1065.65    1065.46    47    ETGMGIAEDK +Oxidation (M)    37_7   
		    209    744.49    1486.96    1486.77    39    EVDLTLATGGPGMVK    37A_7   
		    54    433.24    864.47    864.40    49    FNSSDAPK    37_7   
		    452    1011.19    2020.37    2019.92    57    HTTFFEVEPDPTLECAK    37_7   
		    568    1205.91    2409.81    2409.35    30    IAEPIGVIAAVVPTTNPTSTAIFK    37_7   
		    284    714.98    1427.94    1427.69    58    IFATYSQEQVDK    37_7   
		    359    1019.31    2036.60    2036.06    85    ILINTPSSQGGIGDLYNFK    37A_7   
		    100    581.50    1160.98    1160.62    78    IVGQTACTIAK    37A_7   
		    549    1161.83    2321.65    2321.09    91    LVEDGGFGHTSSLYIDDVNQR    37_7   
		    351    818.57    1635.13    1634.74    59    NHYASEYIYNAYK    37_7   
		    124    516.36    1030.70    1030.60    71    TAVNSILVSK    37_7   
		    271    567.98    1700.93    1700.77    39    TCGVIEKDEAFGMTK +Oxidation (M)    37A_7   
		    423    710.19    2127.54    2127.07    77    VLIGEVESVEIEEAFAHEK    37A_2   
		    101    485.84    969.66    969.57    51    VPLAIMAQK    37_7   
		    110    493.85    985.68    985.56    29    VPLAIMAQK +Oxidation (M)    37_7   
		    151    570.43    1138.85    1138.64    69    YAGIASFLGLK    37_7   
   
      Matching Genes:  
               gi|115252023|emb|CAJ69859.1|  (aldehyde-alcohol dehydrogenase [includes: alcohol dehydrogenase and pyruvate-formate-lyase deactivase [Clostridium difficile 630]) 
           
  Protein Group 7   
      Expression Quality:  
         Score      Num Spectra      Num Peptides      High-Qual Peptides      % Coverage       955    37    16    14    41   
   
      Peptides:   
        Query    Observed    Mr(expt)    Mr(calc)    Score    Peptide    Result File   
		    592    1058.89    3173.66    3173.59    99    ALEAANMTIEDIDLVEANEAFAAQSVAVIR    37A_5   
		    633    1064.39    3190.14    3189.59    55    ALEAANMTIEDIDLVEANEAFAAQSVAVIR +Oxidation (M)    37_5   
		    537    1370.81    2739.60    2738.43    86    ANITPDMIDESLLGGVLTAGLGQNIAR    37A_5   
		    539    1378.53    2755.05    2754.42    79    ANITPDMIDESLLGGVLTAGLGQNIAR +Oxidation (M)    37A_5   
		    325    936.68    1871.34    1871.00    63    AQAEGKFDEEIVPVVIK    37_5   
		    432    1132.33    2262.64    2262.10    82    DGTVTAGNASGINDGAAMLVVMAK    37A_5   
		    439    1148.11    2294.20    2294.09    71    DGTVTAGNASGINDGAAMLVVMAK +2 Oxidation (M)    37A_5   
		    461    1140.31    2278.61    2278.09    56    DGTVTAGNASGINDGAAMLVVMAK +Oxidation (M)    37_5   
		    72    481.82    961.63    961.45    35    DLNIDMNK    37_5   
		    226    738.02    1474.04    1473.69    69    EEQDELALASQNK    37_5   
		    141    644.49    1286.96    1286.71    46    FDEEIVPVVIK    37A_5   
		    113    613.01    1224.00    1223.68    43    ILTTLLYEMK    37_5   
		    120    620.90    1239.78    1239.68    38    ILTTLLYEMK +Oxidation (M)    37A_5   
		    96    575.39    1148.76    1148.59    45    IMGYGPVPATK +Oxidation (M)    37A_5   
		    218    616.37    1230.73    1230.68    45    SVSAVELGVTAAK    37A_1   
		    74    521.32    1040.62    1040.53    43    TAVGSFGGAFK    37A_5   
   
      Matching Genes:  
               gi|115250080|emb|CAJ67900.1|  (acetyl-CoA acetyltransferase [Clostridium difficile 630]) 
           
  Protein Group 8   
      Expression Quality:  
         Score      Num Spectra      Num Peptides      High-Qual Peptides      % Coverage       943    30    15    15    59   
   
      Peptides:   
        Query    Observed    Mr(expt)    Mr(calc)    Score    Peptide    Result File   
		    219    665.01    1328.01    1327.73    67    ALENVLKDDLAK    37A_4   
		    526    1056.83    2111.65    2111.09    56    ALEVGIDPILCVGETLEQR    37_4   
		    126    596.34    1190.67    1190.62    50    EALEFVNEIK    37A_4   
		    331    717.99    1433.97    1433.74    64    EALEFVNEIKDK    37_4   
		    319    832.46    1662.91    1661.77    49    EIDMDYVVIGHSER    37A_4   
		    326    840.10    1678.18    1677.77    64    EIDMDYVVIGHSER +Oxidation (M)    37A_4   
		    138    610.84    1219.67    1219.62    64    GLYGELANEVR    37A_4   
		    260    652.39    1302.77    1302.60    67    IGAQNMHFEEK    37_4   
		    271    660.41    1318.80    1318.60    53    IGAQNMHFEEK +Oxidation (M)    37_4   
		    135    513.85    1025.68    1025.60    45    KPIIAGNWK    37_4   
		    342    744.46    1486.90    1486.66    58    QYFNETDETVNK    37_4   
		    301    808.49    1614.96    1614.75    76    QYFNETDETVNKK    37A_4   
		    390    820.05    1638.08    1637.79    91    TATAEDANDVISYIR    37_4   
		    425    1002.67    2003.33    2003.08    57    VNSDKVEAVICAPFTLLK    37A_4   
		    514    802.07    1602.12    1601.88    82    VVVAYEPIWAIGTGK    37_3   
   
      Matching Genes:  
               gi|115252229|emb|CAJ70069.1|  (triosephosphate isomerase [Clostridium difficile 630]) 
           
  Protein Group 9   
      Expression Quality:  
         Score      Num Spectra      Num Peptides      High-Qual Peptides      % Coverage       893    34    17    13    37   
   
      Peptides:   
        Query    Observed    Mr(expt)    Mr(calc)    Score    Peptide    Result File   
		    119    528.34    1054.66    1054.51    32    AFDITFADR    37_6   
		    382    880.13    1758.25    1757.88    65    ALQSGTSHFLGQHFTK    37_6   
		    245    676.93    1351.85    1351.62    61    DIENNQAMVFR +Oxidation (M)    37_6   
		    90    556.35    1110.68    1111.49    47    EADSMVVMAK +2 Oxidation (M)    37A_7   
		    128    548.83    1095.64    1095.49    46    EADSMVVMAK +Oxidation (M)    37_6   
		    442    755.51    2263.49    2263.07    64    EAEHVEGFAPEVAWVTHGGNK    37A_6   
		    403    914.16    1826.30    1825.82    42    EDNTSIVENMDEFRK    37_6   
		    410    922.14    1842.26    1841.81    34    EDNTSIVENMDEFRK +Oxidation (M)    37_6   
		    419    931.13    1860.25    1859.86    76    EGNLANPYHTSWGASTR    37_6   
		    83    559.38    1116.75    1116.61    39    IKEETGATIR    37A_6   
		    85    560.82    1119.62    1119.56    80    KGNVMETVDK    37A_6   
		    330    944.57    1887.14    1886.84    45    MEDDFPQWYTDVITK    37A_6   
		    335    952.63    1903.24    1902.83    79    MEDDFPQWYTDVITK +Oxidation (M)    37A_6   
		    84    560.80    1119.58    1119.58    59    TDLVDYAPVK    37A_6   
		    292    737.38    1472.74    1472.54    38    TMWCGDAECEAK +Oxidation (M)    37_6   
		    203    714.83    1427.64    1427.61    43    TYTIEAMMHDGK +2 Oxidation (M)    37A_6   
		    199    709.57    1417.13    1416.91    43    VAPIQVVIVPIAAK    37A_6   
   
      Matching Genes:  
               gi|115249053|emb|CAJ66864.1|  (putative dual-specificity prolyl/cysteinyl-tRNA synthetase [Clostridium difficile 630]) 
           
  Protein Group 10   
      Expression Quality:  
         Score      Num Spectra      Num Peptides      High-Qual Peptides      % Coverage       841    27    15    13    31   
   
      Peptides:   
        Query    Observed    Mr(expt)    Mr(calc)    Score    Peptide    Result File   
		    614    1342.45    2682.88    2682.27    103    AEVTDVANAIYDGTDAIMLSGETAAGK    37_7   
		    98    574.32    1146.63    1146.55    56    DGEVVTVDASR    37A_7   
		    573    806.17    2415.49    2415.20    50    DISDIEFGISQGIDYIAASFVR    37_7   
		    396    928.25    1854.48    1853.94    41    GDLGVEIPTEEMPIVQK    37_7   
		    278    872.08    1742.14    1741.87    85    IENQEGVENLDEILK    37A_7   
		    65    486.88    971.74    971.58    46    KASDVLAIR    37A_7   
		    219    649.39    1296.77    1296.59    31    RTEETLDYDR    37_7   
		    222    765.51    1529.00    1528.81    54    SGDSILIDDGLVGLR    37A_7   
		    160    594.39    1186.76    1186.62    68    SPIIATTNNEK    37_7   
		    166    674.89    1347.76    1347.65    79    SSVAGNTDEVIEK    37A_7   
		    152    571.34    1140.66    1140.49    41    TEETLDYDR    37_7   
		    298    920.09    1838.17    1837.84    76    TGNFEDPEVFLEEGQK    37A_7   
		    57    474.36    946.71    946.49    40    VSDGIMVAR    37A_7   
		    62    482.28    962.54    962.49    30    VSDGIMVAR +Oxidation (M)    37A_7   
		    5    403.29    804.56    804.44    41    YPVEAVK    37_7   
   
      Matching Genes:  
               gi|115252454|emb|CAJ70297.1|  (pyruvate kinase [Clostridium difficile 630]) 
           
  Protein Group 11   
      Expression Quality:  
         Score      Num Spectra      Num Peptides      High-Qual Peptides      % Coverage       819    55    12    12    64   
   
      Peptides:   
        Query    Observed    Mr(expt)    Mr(calc)    Score    Peptide    Result File   
		    114    452.34    902.67    902.53    56    AFLGLLNR    37A_1   
		    222    628.33    1254.64    1254.58    73    EGYPEVAEAYK    37A_2   
		    428    706.42    1410.83    1410.68    41    EGYPEVAEAYKR    37_3   
		    302    746.03    1490.04    1489.80    101    FAELLGEVVVADTK    37A_3   
		    480    880.60    2638.76    2638.14    40    FVCTVCGYIHEGDAAPAQCPVCK    37A_2   
		    286    615.32    1228.64    1228.53    46    GEMVWADEHR    37_2   
		    194    608.38    1214.75    1214.59    62    IAFEEAEHAAK    37A_2   
		    541    820.57    1639.12    1638.89    121    IGVAQGVDAEIIEGLR    37_2   
		    250    563.30    1124.58    1124.50    70    VDAEYGATDGK    37_2   
		    247    577.34    1152.67    1152.55    58    VGADKFEEMK    37_3   
		    254    585.32    1168.63    1168.54    58    VGADKFEEMK +Oxidation (M)    37_3   
		    264    690.95    1379.88    1379.67    93    VRVDAEYGATDGK    37A_3   
   
      Matching Genes:  
               gi|115250565|emb|CAJ68389.1|  (putative rubrerythrin [Clostridium difficile 630]) 
           
  Protein Group 12   
      Expression Quality:  
         Score      Num Spectra      Num Peptides      High-Qual Peptides      % Coverage       808    25    13    12    38   
   
      Peptides:   
        Query    Observed    Mr(expt)    Mr(calc)    Score    Peptide    Result File   
		    522    1286.41    2570.81    2570.27    137    DILDILEDNNISVVADDLAQETR    37_5   
		    272    856.53    1711.05    1710.77    57    ELEEICGYEIEEAK    37_5   
		    100    585.38    1168.74    1168.61    55    EVVENPNAAVK    37_5   
		    259    851.96    1701.91    1701.73    51    FCDPEEYDYPLVR    37A_5   
		    99    576.89    1151.76    1151.64    53    HSNTIKPSIR    37_5   
		    243    509.29    1524.85    1524.73    59    IHESIEVYNEHR    37_5   
		    167    675.88    1349.75    1349.60    38    LNAMPEEVCSGK +Oxidation (M)    37A_5   
		    210    714.98    1427.94    1427.74    55    MKEVVENPNAAVK    37_5   
		    192    722.97    1443.93    1443.74    74    MKEVVENPNAAVK +Oxidation (M)    37A_5   
		    274    884.48    1766.94    1766.79    60    QWSNIEGCSLAYDPK    37A_5   
		    126    629.83    1257.65    1257.58    49    TDVPAGDDALER    37A_5   
		    92    565.44    1128.86    1128.68    69    VLLTGILADSK    37A_5   
		    108    613.92    1225.82    1225.66    51    YISLVHPQNR    37A_5   
   
      Matching Genes:  
               gi|115249404|emb|CAJ67219.1|  (subunit of oxygen-sensitive 2-hydroxyisocaproyl-CoA dehydratase [Clostridium difficile 630]) 
           
  Protein Group 13   
      Expression Quality:  
         Score      Num Spectra      Num Peptides      High-Qual Peptides      % Coverage       794    29    14    10    30   
   
      Peptides:   
        Query    Observed    Mr(expt)    Mr(calc)    Score    Peptide    Result File   
		    455    814.23    2439.68    2439.29    59    DSPLVVGVGEGENFIASDIPALLK    37A_7   
		    156    583.37    1164.72    1164.59    35    EIHEQPTGVR    37_7   
		    382    1086.85    2171.68    2171.01    67    ESDDVFYTWAGPEVAVASTK    37A_7   
		    370    861.17    1720.32    1719.92    75    FVNIPVITDIASEFR    37_6   
		    247    679.45    1356.89    1356.71    67    GAYVVAIAQSHNK    37_6   
		    258    700.48    1398.95    1398.67    73    GIDYSLAMEGSLK +Oxidation (M)    37_6   
		    171    614.47    1226.92    1226.69    37    GTPVIAIATQEK    37_6   
		    304    749.48    1496.96    1496.67    105    GYDSAGVAVNSSNEK    37_6   
		    64    491.34    980.67    980.57    56    HGTIALIEK    37A_6   
		    286    715.56    1429.10    1428.83    65    ILSITNVVGSSIAR    37_7   
		    228    753.87    1505.73    1505.76    50    IQEILDNEEYIK    37A_6   
		    149    563.84    1125.66    1125.48    43    MVSNMEEVR +2 Oxidation (M)    37_7   
		    80    555.39    1108.76    1109.48    26    MVSNMEEVR +Oxidation (M)    37A_6   
		    287    885.72    1769.43    1768.92    36    VYIVACGTAYNAGLLGK    37A_7   
   
      Matching Genes:  
               gi|115249129|emb|CAJ66940.1|  (glucosamine--fructose-6-phosphate aminotransferase [isomerizing] [Clostridium difficile 630]) 
           
  Protein Group 14   
      Expression Quality:  
         Score      Num Spectra      Num Peptides      High-Qual Peptides      % Coverage       783    38    14    12    40   
   
      Peptides:   
        Query    Observed    Mr(expt)    Mr(calc)    Score    Peptide    Result File   
		    613    1270.90    2539.79    2539.30    78    AYEGGFAIGAFNISDLEQLQGVLK    37_4   
		    143    530.38    1058.74    1058.56    66    DAIQAVVESK    37_4   
		    140    611.47    1220.93    1220.63    60    FDILEEIQSK    37A_4   
		    8    409.73    817.46    817.43    34    FLAENPK    37A_4   
		    115    489.27    976.53    976.46    59    INMDTDLR    37_4   
		    83    497.23    992.44    992.46    53    INMDTDLR +Oxidation (M)    37A_4   
		    72    473.78    945.54    945.53    49    KFLAENPK    37A_4   
		    40    431.80    861.58    861.47    34    LAMTAAIR +Oxidation (M)    37A_4   
		    289    780.94    1559.87    1559.73    56    NSYVMIQASMSAVK +2 Oxidation (M)    37A_4   
		    285    772.91    1543.81    1543.74    55    NSYVMIQASMSAVK +Oxidation (M)    37A_4   
		    346    873.09    1744.17    1743.92    63    TGVDSLAIAIGTSHGAFK    37A_4   
		    283    448.97    1343.90    1343.69    58    YAGPHTLVEMVK    37_4   
		    231    680.98    1359.94    1359.69    57    YAGPHTLVEMVK +Oxidation (M)    37A_4   
		    476    770.00    1537.99    1537.74    61    YTQPAEAVEFVER    37_3   
   
      Matching Genes:  
               gi|115249409|emb|CAJ67224.1|  (putative fructose-bisphosphate aldolase [Clostridium difficile 630]) 
           
  Protein Group 15   
      Expression Quality:  
         Score      Num Spectra      Num Peptides      High-Qual Peptides      % Coverage       777    27    11    11    33   
   
      Peptides:   
        Query    Observed    Mr(expt)    Mr(calc)    Score    Peptide    Result File   
		    468    683.12    2046.33    2046.08    70    AAADEIGLPLFQYLGGVNAK    37_6   
		    75    537.85    1073.68    1073.56    69    AGYTAVISHR    37A_6   
		    287    888.15    1774.29    1773.93    101    AIVPSGASTGAFEAVELR    37A_6   
		    169    664.89    1327.77    1327.76    71    EALELIVEAITK    37A_6   
		    159    657.48    1312.96    1312.73    83    GIENGVANSILVK    37A_6   
		    145    581.35    1160.69    1160.55    57    IEEMVGEQAR    37_6   
		    99    589.34    1176.66    1176.54    56    IEEMVGEQAR +Oxidation (M)    37A_6   
		    60    482.32    962.62    963.54    49    KYVLAGEGK    37A_6   
		    285    730.06    1458.11    1457.80    45    LGANAILGVSMAVAR +Oxidation (M)    37_6   
		    274    860.72    1719.42    1718.88    83    LQLVGDDLFVTNTER    37A_7   
		    516    1095.30    2188.58    2188.09    93    SGETEDSTIADLAVAVNAGQIK    37_6   
   
      Matching Genes:  
               gi|115252227|emb|CAJ70067.1|  (enolase [Clostridium difficile 630]) 
           
  Protein Group 16   
      Expression Quality:  
         Score      Num Spectra      Num Peptides      High-Qual Peptides      % Coverage       766    17    14    10    26   
   
      Peptides:   
        Query    Observed    Mr(expt)    Mr(calc)    Score    Peptide    Result File   
		    229    757.47    1512.92    1512.83    30    AVLEGMDGKPIVIR +Oxidation (M)    37A_6   
		    67    500.73    999.44    1000.56    38    GKPSVTLDGK    37A_6   
		    310    925.74    1849.46    1848.97    37    IMFPMISSLEELLQAK    37A_6   
		    364    849.65    1697.29    1696.89    62    ISYLYNQFNPAVLR    37_6   
		    51    466.31    930.61    930.50    51    LEDAVAVSK    37A_6   
		    232    765.52    1529.03    1528.83    50    LEDAVAVSKEELVK    37A_6   
		    494    884.02    2649.03    2648.36    53    MIPILLGMGLDEFSMSPISILPAR +3 Oxidation (M)    37A_6   
		    612    873.21    2616.61    2616.37    80    MIPILLGMGLDEFSMSPISILPAR +Oxidation (M)    37_6   
		    199    639.97    1277.92    1277.68    77    MVLGFLTDIGGR    37_6   
		    213    647.95    1293.88    1293.68    64    MVLGFLTDIGGR +Oxidation (M)    37_6   
		    126    547.32    1092.63    1092.52    58    NDAEGVGLYR    37_6   
		    102    594.83    1187.64    1187.60    32    SIDNVEAEIAK    37A_6   
		    328    791.03    1580.05    1579.77    71    TESVNAEYALNEIK    37_6   
		    191    698.86    1395.70    1395.55    63    WAGMCGESAGDQK    37A_6   
   
      Matching Genes:  
               gi|115251808|emb|CAJ69643.1|  (phosphoenolpyruvate-protein phosphotransferase [Clostridium difficile 630]) 
           
  Protein Group 17   
      Expression Quality:  
         Score      Num Spectra      Num Peptides      High-Qual Peptides      % Coverage       748    20    14    10    24   
   
      Peptides:   
        Query    Observed    Mr(expt)    Mr(calc)    Score    Peptide    Result File   
		    416    757.16    2268.47    2268.04    36    AGIVTEEELQEYMDHFVMK    37A_7   
		    58    476.27    950.53    950.49    34    AVMPYGGIK +Oxidation (M)    37A_7   
		    150    568.32    1134.62    1134.51    34    DQNGAAMSLGR +Oxidation (M)    37_7   
		    494    936.61    2806.80    2807.29    64    EFIQLNYSPYEGNDSFLAGATENTK    37A_7   
		    199    717.40    1432.79    1432.68    58    ERENGGTLDVDTK    37A_7   
		    363    1033.19    2064.36    2064.92    68    FEPITSEYLDYDEVMSK    37A_7   
		    467    1041.71    2081.40    2080.92    46    FEPITSEYLDYDEVMSK +Oxidation (M)    37_7   
		    451    1214.83    2427.65    2427.24    28    LPYEHAQDGISNTFSIVPAALGK    37A_7   
		    388    923.61    1845.20    1844.86    90    MAESYGFDISKPATNSK    37_7   
		    401    621.44    1861.30    1860.86    47    MAESYGFDISKPATNSK +Oxidation (M)    37_7   
		    469    1043.36    2084.71    2084.08    50    NSYPTQSILTITSNVVYGK    37_7   
		    152    660.48    1318.95    1318.69    41    SGIITGLPDAYGR    37A_7   
		    437    986.69    1971.36    1970.89    104    VSIDTSSVQYENDDLMR    37_7   
		    443    994.66    1987.30    1986.88    48    VSIDTSSVQYENDDLMR +Oxidation (M)    37_7   
   
      Matching Genes:  
               gi|115249776|emb|CAJ67593.1|  (formate acetyltransferase [Clostridium difficile 630]) 
           
  Protein Group 18   
      Expression Quality:  
         Score      Num Spectra      Num Peptides      High-Qual Peptides      % Coverage       739    26    13    10    40   
   
      Peptides:   
        Query    Observed    Mr(expt)    Mr(calc)    Score    Peptide    Result File   
		    87    556.88    1111.74    1111.57    35    ALSIWMTFK +Oxidation (M)    37A_5   
		    88    533.30    1064.58    1064.50    49    EAAYMHSIK +Oxidation (M)    37_5   
		    385    886.16    1770.30    1769.92    68    GGIIVDPSTLSQGELER    37_6   
		    53    464.82    927.62    927.47    34    GYIDGIYK    37A_5   
		    464    1011.78    2021.54    2021.03    89    IAVQGIGNVGSYTVLNCEK    37_6   
		    215    649.43    1296.85    1297.63    43    IKEEYNVTMR +Oxidation (M)    37_6   
		    175    681.99    1361.97    1361.73    46    LGMEPAVYELLK    37A_5   
		    452    1130.83    2259.65    2259.22    57    LTGQSSIGVITGKPVEFGGSLGR    37_5   
		    458    1138.77    2275.53    2275.04    68    LVCEAANGPTTPEADEVFAER    37_5   
		    496    1285.21    2568.41    2568.11    89    SEGSYAIYNENGLDGQAMLDYMK    37A_5   
		    502    1293.63    2585.24    2584.11    32    SEGSYAIYNENGLDGQAMLDYMK +Oxidation (M)    37A_5   
		    117    611.40    1220.79    1220.65    73    TAATGFGVAVTAR    37A_6   
		    103    492.36    982.70    982.61    56    VIEVSIPVK    37_6   
   
      Matching Genes:  
               gi|115249189|emb|CAJ67001.1|  (NAD-specific glutamate dehydrogenase [Clostridium difficile 630]) 
           
  Protein Group 19   
      Expression Quality:  
         Score      Num Spectra      Num Peptides      High-Qual Peptides      % Coverage       730    22    12    12    32   
   
      Peptides:   
        Query    Observed    Mr(expt)    Mr(calc)    Score    Peptide    Result File   
		    120    529.89    1057.76    1057.60    47    AVTVAVEELK    37_6   
		    176    668.91    1335.82    1335.65    44    ESTTIVDGSGDKK    37A_6   
		    53    468.32    934.64    934.51    43    GTFDVVAVK    37A_6   
		    502    717.45    2149.34    2148.22    86    ISNIQELLPVLEQIVQQGK    37_6   
		    59    478.30    954.59    954.55    42    KALEEPLR    37A_6   
		    2    407.31    812.60    812.51    41    LAGGVAVVK    37A_6   
		    150    587.48    1172.94    1172.65    59    LIAEAMEIVGK    37_6   
		    613    709.15    2124.43    2125.19    72    LLIIAEDVEGEALSTLVVNK    37_2   
		    126    619.89    1237.76    1237.71    64    NVTAGANPILLR    37A_6   
		    720    1105.56    3313.66    3312.68    48    QIAINAGLEGAVIVQNVVNSEAETGFDALNEK    37_3   
		    373    1044.81    2087.60    2087.09    108    TNDVAGDGTTTATVLAQAIIR    37A_6   
		    113    508.83    1015.64    1015.55    76    VGAATEVELK    37_6   
   
      Matching Genes:  
               gi|115249204|emb|CAJ67016.1|  (60 kDa chaperonin [Clostridium difficile 630]) 
           
  Protein Group 20   
      Expression Quality:  
         Score      Num Spectra      Num Peptides      High-Qual Peptides      % Coverage       697    49    10    10    58   
   
      Peptides:   
        Query    Observed    Mr(expt)    Mr(calc)    Score    Peptide    Result File   
		    114    452.34    902.67    902.53    56    AFLGLLNR    37A_1   
		    196    621.37    1240.72    1240.56    53    EGYPEVGEAYK    37A_3   
		    302    746.03    1490.04    1489.80    101    FAELLGEVVVADTK    37A_3   
		    480    880.60    2638.76    2638.14    40    FVCTVCGYIHEGDAAPAQCPVCK    37A_2   
		    183    591.37    1180.73    1180.61    66    IALEEAEHAAK    37A_2   
		    385    849.69    1697.36    1696.90    102    IGVAQGVDEEIIEGLR    37A_1   
		    250    563.30    1124.58    1124.50    70    VDAEYGATDGK    37_2   
		    247    577.34    1152.67    1152.55    58    VGADKFEEMK    37_3   
		    254    585.32    1168.63    1168.54    58    VGADKFEEMK +Oxidation (M)    37_3   
		    264    690.95    1379.88    1379.67    93    VRVDAEYGATDGK    37A_3   
   
      Matching Genes:  
               gi|115250515|emb|CAJ68339.1|  (putative ruberythrin [Clostridium difficile 630]) 
           
  Protein Group 21   
      Expression Quality:  
         Score      Num Spectra      Num Peptides      High-Qual Peptides      % Coverage       695    42    13    9    39   
   
      Peptides:   
        Query    Observed    Mr(expt)    Mr(calc)    Score    Peptide    Result File   
		    78    497.30    992.59    992.52    47    ELITFGADK    37_5   
		    211    715.44    1428.86    1429.75    29    EVDAELCAILLGK    37_5   
		    300    683.55    1365.08    1364.80    80    IAPVVIELLGEGR    37_4   
		    37    430.80    859.58    859.44    44    ITQDDIR    37A_4   
		    174    665.92    1329.82    1329.70    41    LDSVDDLLEAIK    37_5   
		    320    701.48    1400.95    1400.74    85    LDSVDDLLEAIKA    37_4   
		    98    543.88    1085.74    1085.56    37    LEIDPEDKK    37A_4   
		    14    416.30    830.59    830.46    47    LGGVVGSSR    37_5   
		    580    1006.38    3016.13    3015.60    60    NPAAPILEIADYGVVGDLHEIVPMLIEK    37A_4   
		    108    554.88    1107.75    1107.58    93    TGEVIALDYK    37A_4   
		    53    445.83    889.65    889.51    35    TTVLETVK    37A_4   
		    95    561.92    1121.82    1121.54    69    VGTGLTADCTK    37_5   
		    112    481.79    961.57    961.44    28    YTTDAYTK    37_4   
   
      Matching Genes:  
               gi|115249407|emb|CAJ67222.1|  (electron transfer flavoprotein alpha-subunit [Clostridium difficile 630]) 
              Other Genes Matching Peptide Subset:  
               gi|115249822|emb|CAJ67639.1|  (electron transfer flavoprotein alpha-subunit [Clostridium difficile 630]) 
           
  Protein Group 22   
      Expression Quality:  
         Score      Num Spectra      Num Peptides      High-Qual Peptides      % Coverage       660    23    12    9    42   
   
      Peptides:   
        Query    Observed    Mr(expt)    Mr(calc)    Score    Peptide    Result File   
		    448    905.14    1808.27    1807.91    93    AFAGADTWATSSALAGALK    37_4   
		    31    415.80    829.59    829.45    61    AGLEEAIK    37_4   
		    95    536.37    1070.73    1070.63    28    AGLEEAIKLK    37A_4   
		    515    1041.29    2080.56    2080.07    61    DGVPSIINPDDKAGLEEAIK    37_4   
		    199    650.34    1298.66    1298.67    56    DIEVDPSNLGLK    37A_4   
		    108    475.27    948.52    948.43    26    EALAMGADR +Oxidation (M)    37_4   
		    155    550.38    1098.75    1098.60    57    LDPNTGTLIR    37_4   
		    96    538.84    1075.66    1075.58    50    MPCLITTLK    37A_4   
		    101    546.89    1091.77    1091.57    58    MPCLITTLK +Oxidation (M)    37A_4   
		    87    508.76    1015.50    1015.52    39    QVPDTTEVK    37A_4   
		    276    747.04    1492.06    1491.76    78    SVKPAGTIYNEDAK    37A_4   
		    90    459.31    916.61    916.52    53    TSAGIIIDK    37_4   
   
      Matching Genes:  
               gi|115250076|emb|CAJ67896.1|  (electron transfer flavoprotein beta-subunit [Clostridium difficile 630]) 
           
  Protein Group 23   
      Expression Quality:  
         Score      Num Spectra      Num Peptides      High-Qual Peptides      % Coverage       645    15    9    8    22   
   
      Peptides:   
        Query    Observed    Mr(expt)    Mr(calc)    Score    Peptide    Result File   
		    415    1110.31    2218.60    2218.17    65    AFVVGGTGLADAMSIAPVASQLK +Oxidation (M)    37A_5   
		    392    1049.77    2097.53    2097.07    85    DGSTKEDQLVDALAAAPIAGR    37_5   
		    480    1178.90    2355.79    2355.22    64    ESPAPIILATDTLSSDQNVAVSK    37_5   
		    273    743.92    1485.83    1485.80    70    IITNQADAEAIVTK    37A_4   
		    380    1031.67    2061.33    2061.92    36    LGDGDYVDFSVDYNLENK    37_5   
		    163    660.94    1319.86    1319.71    62    LYNLVNTQLDK    37A_6   
		    199    737.91    1473.81    1473.63    79    SGGSEDTGYVVEMK +Oxidation (M)    37A_5   
		    330    940.17    1878.32    1877.97    106    VDVTGGSTPSAVAVSGFVTK    37_5   
		    221    773.88    1545.74    1545.69    78    YYNSDDKNAITDK    37A_5   
   
      Matching Genes:  
               gi|115251846|emb|CAJ69681.1|  (cell surface protein (S-layer precursor protein) [Clostridium difficile 630]) 
           
  Protein Group 24   
      Expression Quality:  
         Score      Num Spectra      Num Peptides      High-Qual Peptides      % Coverage       589    25    10    7    28   
   
      Peptides:   
        Query    Observed    Mr(expt)    Mr(calc)    Score    Peptide    Result File   
		    222    435.31    1302.91    1302.71    31    ALQLHGGYGFIK    37_7   
		    198    631.40    1260.79    1260.59    51    ELDTLPAEMDK    37_7   
		    143    639.42    1276.83    1276.59    25    ELDTLPAEMDK +Oxidation (M)    37_5   
		    76    531.80    1061.58    1061.55    70    IAMGTLEVGR +Oxidation (M)    37A_5   
		    322    627.38    1879.13    1879.08    100    IGVAALALGIAQGALDEAVK    37A_5   
		    292    727.02    1452.02    1451.75    94    IVSIYEGTSEVQK    37_7   
		    77    495.35    988.68    989.56    33    MVISSNVLK    37_5   
		    68    494.37    986.72    986.59    50    VQFGKPIAK    37A_5   
		    249    675.40    1348.78    1348.57    56    WDGFSTGAHEDK    37_7   
		    318    761.02    1520.02    1519.72    79    YYASEIANEVAYK    37_7   
   
      Matching Genes:  
               gi|115249405|emb|CAJ67220.1|  (acyl-CoA dehydrogenase, short-chain specific [Clostridium difficile 630]) 
           
  Protein Group 25   
      Expression Quality:  
         Score      Num Spectra      Num Peptides      High-Qual Peptides      % Coverage       589    24    8    8    43   
   
      Peptides:   
        Query    Observed    Mr(expt)    Mr(calc)    Score    Peptide    Result File   
		    485    970.22    1938.42    1937.95    81    AFGGADTWATSNTIAAGISK    37_4   
		    630    694.50    2080.47    2080.03    65    DGVPSILNPDDANALEEALK    37_3   
		    355    883.45    1764.89    1764.80    105    ECLAMGADDAILLSDR +Oxidation (M)    37A_4   
		    420    871.10    1740.18    1739.87    111    QAIDGDTAQVGPQIAEK    37_4   
		    168    604.41    1206.81    1206.61    70    QLEDGYELIK    37A_3   
		    142    529.33    1056.65    1056.52    44    QVPDTNEVR    37_4   
		    180    613.33    1224.64    1224.61    67    VGDYDIIFAGR    37A_3   
		    142    564.35    1126.69    1126.70    46    VSTPVLLTAVK    37A_3   
   
      Matching Genes:  
               gi|115249406|emb|CAJ67221.1|  (electron transfer flavoprotein beta-subunit [Clostridium difficile 630]) 
           
  Protein Group 26   
      Expression Quality:  
         Score      Num Spectra      Num Peptides      High-Qual Peptides      % Coverage       551    20    9    6    24   
   
      Peptides:   
        Query    Observed    Mr(expt)    Mr(calc)    Score    Peptide    Result File   
		    202    641.44    1280.86    1280.66    70    ESTIEFLTSVR    37_6   
		    101    592.39    1182.76    1182.63    78    GISDFLLSFGK    37A_6   
		    561    1151.77    2301.53    2301.01    96    HEFTTSDPGMTYSVAETSVDK    37_6   
		    562    1159.76    2317.50    2317.01    86    HEFTTSDPGMTYSVAETSVDK +Oxidation (M)    37_6   
		    72    515.28    1028.54    1028.45    35    ICSETYEK    37A_6   
		    614    874.75    2621.22    2622.38    42    LLAQALGANYDLIAQYPAWEFKK    37_6   
		    211    485.61    1453.82    1453.76    32    NVEHDFLKDPIK    37A_6   
		    302    748.02    1494.02    1493.71    29    SSVMSIRDEIADR +Oxidation (M)    37_6   
		    350    990.26    1978.50    1978.03    83    VLSVLNVDYELASVDGGTK    37A_6   
   
      Matching Genes:  
               gi|115249724|emb|CAJ67541.1|  (putative aminoacyl-histidine dipeptidase [Clostridium difficile 630]) 
           
  Protein Group 27   
      Expression Quality:  
         Score      Num Spectra      Num Peptides      High-Qual Peptides      % Coverage       532    13    10    6    22   
   
      Peptides:   
        Query    Observed    Mr(expt)    Mr(calc)    Score    Peptide    Result File   
		    149    657.48    1312.94    1312.80    30    EVIPEFLLLLK    37A_7   
		    253    825.46    1648.91    1648.67    71    FAGMDLGMNFEEEK +2 Oxidation (M)    37A_7   
		    276    862.56    1723.11    1722.85    25    FIDNGIGMTEEEIKK    37A_7   
		    161    594.92    1187.83    1187.64    66    LVSLGEISENK    37_7   
		    322    769.52    1537.02    1536.79    29    LYNNQVFVADNIK    37_7   
		    377    895.16    1788.31    1787.87    61    NEDTPAMVLVSEQSIR    37_7   
		    402    931.66    1861.31    1860.93    32    VFYVSDKEQQSQYIK    37_7   
		    254    826.59    1651.17    1650.90    48    VIEPLNDTNPLWLK    37A_7   
		    333    965.10    1928.19    1927.84    79    WISEGGTEYEISESDAR    37A_7   
		    301    925.61    1849.20    1848.87    91    YINQVAFSGAEDFFNK    37A_7   
   
      Matching Genes:  
               gi|115249282|emb|CAJ67095.1|  (chaperone protein (heat shock protein) [Clostridium difficile 630]) 
           
  Protein Group 28   
      Expression Quality:  
         Score      Num Spectra      Num Peptides      High-Qual Peptides      % Coverage       526    12    8    6    44   
   
      Peptides:   
        Query    Observed    Mr(expt)    Mr(calc)    Score    Peptide    Result File   
		    472    710.90    1419.78    1419.67    72    EIMDAANNTGASVK    37_2   
		    311    718.92    1435.82    1435.66    71    EIMDAANNTGASVK +Oxidation (M)    37A_2   
		    266    669.99    1337.97    1337.72    87    IVYDAFAIVAEK    37A_2   
		    249    558.82    1115.62    1115.60    68    LINNLMVDGK    37_2   
		    169    566.86    1131.70    1131.60    38    LINNLMVDGK +Oxidation (M)    37A_2   
		    57    422.28    842.55    842.51    36    RQTLGLR    37_2   
		    209    618.89    1235.77    1235.60    95    TGEEALEVFNK    37A_2   
		    566    595.38    1783.11    1782.94    59    VGGANYQVPIEVRPER    37_2   
   
      Matching Genes:  
               gi|115249073|emb|CAJ66884.1|  (30S ribosomal protein S7 [Clostridium difficile 630]) 
           
  Protein Group 29   
      Expression Quality:  
         Score      Num Spectra      Num Peptides      High-Qual Peptides      % Coverage       519    18    10    8    49   
   
      Peptides:   
        Query    Observed    Mr(expt)    Mr(calc)    Score    Peptide    Result File   
		    188    501.32    1000.62    1000.56    57    GTQAVGIVEK    37_3   
		    203    518.37    1034.73    1034.65    43    IILLGPPGAGK    37_3   
		    626    1037.80    2073.59    2073.07    67    IQVYLDETKPLVDYYSK    37_3   
		    18    409.25    816.48    816.43    44    NAGISLDK    37_3   
		    248    577.84    1153.67    1152.54    41    NGFMLDGFPR    37_3   
		    159    585.86    1169.70    1168.53    29    NGFMLDGFPR +Oxidation (M)    37A_3   
		    336    529.06    1584.16    1583.85    38    QGIIADIKGDQAIDK    37A_3   
		    576    906.61    1811.20    1810.80    84    VEGVCDVCQGELYQR    37_3   
		    138    458.31    914.60    914.51    60    VVNIEVDK    37_3   
		    314    511.64    1531.89    1531.78    56    YNIPHISTGDIFR    37A_3   
   
      Matching Genes:  
               gi|115249098|emb|CAJ66909.1|  (adenylate kinase [Clostridium difficile 630]) 
           
  Protein Group 30   
      Expression Quality:  
         Score      Num Spectra      Num Peptides      High-Qual Peptides      % Coverage       464    10    7    5    23   
   
      Peptides:   
        Query    Observed    Mr(expt)    Mr(calc)    Score    Peptide    Result File   
		    219    738.10    1474.18    1474.77    32    EEGIIFNTLTNPK    37A_6   
		    496    1069.26    2136.51    2136.05    72    EGVFAGGDAVTGAATVISAMGAGK    37_6   
		    155    593.89    1185.77    1185.60    61    LGSESYIVYR    37_6   
		    116    611.32    1220.63    1220.50    39    MELGEPDDSGR +Oxidation (M)    37A_6   
		    372    864.16    1726.30    1725.91    101    VAVIGSGPAGLACAGDLAK    37_6   
		    260    701.53    1401.04    1401.70    93    VAVVGGGNVAMDAAR +Oxidation (M)    37_6   
		    349    989.19    1976.37    1975.92    66    VCPQESQCEGVCILGIK    37A_6   
   
      Matching Genes:  
               gi|115250578|emb|CAJ68402.1|  (putative glutamate synthase [NADPH] small chain [Clostridium difficile 630]) 
           
  Protein Group 31   
      Expression Quality:  
         Score      Num Spectra      Num Peptides      High-Qual Peptides      % Coverage       459    13    7    7    14   
   
      Peptides:   
        Query    Observed    Mr(expt)    Mr(calc)    Score    Peptide    Result File   
		    247    531.96    1592.87    1592.78    40    AGTNMERPGPLAAHR +Oxidation (M)    37A_6   
		    90    574.32    1146.63    1146.55    48    EEGELGSGITR    37A_6   
		    685    1071.54    3211.61    3210.60    84    EEVVEETVEEAAPVSEAAVVPVSTGVAGETVK    37_6   
		    212    647.92    1293.82    1293.65    69    IDKVEFADETK    37_6   
		    516    809.07    1616.12    1615.81    103    IVLEENEQSLPMSK    37_3   
		    352    817.00    1631.98    1631.81    75    IVLEENEQSLPMSK +Oxidation (M)    37A_3   
		    96    479.81    957.60    957.51    40    NAIEGVEVK    37A_3   
   
      Matching Genes:  
               gi|115252300|emb|CAJ70141.1|  (proline reductase subunit proprotein [Clostridium difficile 630]) 
           
  Protein Group 32   
      Expression Quality:  
         Score      Num Spectra      Num Peptides      High-Qual Peptides      % Coverage       447    11    7    6    26   
   
      Peptides:   
        Query    Observed    Mr(expt)    Mr(calc)    Score    Peptide    Result File   
		    247    831.49    1660.97    1660.76    63    AHCSTVGAGEFLQER    37A_5   
		    430    1107.33    2212.65    2212.13    72    AQGYEVGTSLLEIDKVEYAK    37_5   
		    253    839.04    1676.07    1675.81    76    EVVFAADDNVVGENAK    37A_5   
		    406    1064.86    2127.70    2127.18    60    FLGEAVANPVRPFTAILGGAK    37_5   
		    245    766.11    1530.21    1529.88    38    LNGALPTIQYLISK    37_5   
		    305    911.10    1820.19    1819.88    80    MTHVSTGGGASLEFLEGK    37_5   
		    311    919.10    1836.18    1835.87    58    MTHVSTGGGASLEFLEGK +Oxidation (M)    37_5   
   
      Matching Genes:  
               gi|115252230|emb|CAJ70070.1|  (phosphoglycerate kinase [Clostridium difficile 630]) 
           
  Protein Group 33   
      Expression Quality:  
         Score      Num Spectra      Num Peptides      High-Qual Peptides      % Coverage       446    11    8    5    22   
   
      Peptides:   
        Query    Observed    Mr(expt)    Mr(calc)    Score    Peptide    Result File   
		    189    624.93    1247.84    1248.59    26    AAIDMVSHSFR +Oxidation (M)    37_6   
		    185    680.33    1358.64    1358.68    69    AFEGTLLAHTDGK    37A_6   
		    62    443.78    885.55    885.42    27    INFDYSK    37_6   
		    298    742.51    1483.01    1482.75    36    NMFALLGKPGYEK +Oxidation (M)    37_6   
		    113    608.30    1214.59    1214.52    44    NYDKEEFDR    37A_6   
		    411    1098.76    2195.50    2195.04    85    QLATEEGYETFVIPDDVGGR    37A_6   
		    190    625.92    1249.83    1249.63    54    SGTTTEPALAFR    37_6   
		    342    816.62    1631.23    1630.87    105    TGLGNTFLGWIDLPK    37_6   
   
      Matching Genes:  
               gi|115252341|emb|CAJ70182.1|  (glucose-6-phosphate isomerase [Clostridium difficile 630]) 
           
  Protein Group 34   
      Expression Quality:  
         Score      Num Spectra      Num Peptides      High-Qual Peptides      % Coverage       431    14    9    7    46   
   
      Peptides:   
        Query    Observed    Mr(expt)    Mr(calc)    Score    Peptide    Result File   
		    250    580.42    1158.82    1158.68    47    ALVFENVLVR    37_3   
		    485    795.14    2382.40    2382.01    25    ASDSFNLEMHVDMEEGNAAGVK +2 Oxidation (M)    37A_3   
		    665    789.75    2366.22    2366.02    63    ASDSFNLEMHVDMEEGNAAGVK +Oxidation (M)    37_3   
		    38    415.78    829.54    829.47    44    EGVIVASR    37_3   
		    144    565.84    1129.67    1129.56    52    ESGDIAGTPGVK    37A_3   
		    569    891.55    1781.09    1780.85    57    FKPLSQPGQYACEEK    37_3   
		    335    428.23    1281.67    1281.58    38    HIHMSNEDATK    37_3   
		    237    565.34    1128.67    1128.58    58    LVGPAGEVEMK    37_3   
		    148    573.36    1144.70    1144.58    47    LVGPAGEVEMK +Oxidation (M)    37A_3   
   
      Matching Genes:  
               gi|115251734|emb|CAJ69569.1|  (putative propanediol utilization protein [Clostridium difficile 630]) 
           
  Protein Group 35   
      Expression Quality:  
         Score      Num Spectra      Num Peptides      High-Qual Peptides      % Coverage       425    21    8    7    37   
   
      Peptides:   
        Query    Observed    Mr(expt)    Mr(calc)    Score    Peptide    Result File   
		    33    416.78    831.55    831.45    48    CLALLDK    37_4   
		    512    692.17    2073.47    2073.12    68    ILIPMINEAVGIYADGVASK    37_4   
		    557    928.09    2781.26    2780.33    42    LAVIGSGTMGSGIVQTFASCGHDVCLK +Oxidation (M)    37A_4   
		    182    637.44    1272.87    1272.73    56    LVEVISGQLTSK    37A_4   
		    503    1022.22    2042.43    2042.08    50    SINKVPVDVSESPGFVVNR    37_4   
		    296    801.20    1600.39    1599.83    58    VPVDVSESPGFVVNR    37A_4   
		    248    643.48    1284.94    1284.66    66    VTFDTVFELSK    37_4   
		    154    547.87    1093.73    1093.64    37    YRPHPLLAK    37_4   
   
      Matching Genes:  
               gi|115250079|emb|CAJ67899.1|  (3-hydroxybutyryl-CoA dehydrogenase [Clostridium difficile 630]) 
           
  Protein Group 36   
      Expression Quality:  
         Score      Num Spectra      Num Peptides      High-Qual Peptides      % Coverage       418    11    8    5    45   
   
      Peptides:   
        Query    Observed    Mr(expt)    Mr(calc)    Score    Peptide    Result File   
		    600    978.18    1954.35    1953.98    101    AIANSDLGLNPSNDGEVIR    37_3   
		    118    445.75    889.49    889.43    39    ANAQMLDK    37_3   
		    75    459.76    917.51    917.51    34    EIDTLLSK    37A_3   
		    44    435.25    868.48    868.44    41    FEFGTIR    37A_3   
		    131    545.83    1089.64    1089.52    41    GGELTEDELK    37A_3   
		    271    609.88    1217.74    1217.61    69    GGELTEDELKK    37_3   
		    229    557.38    1112.75    1113.60    28    LSVPALTEER    37_3   
		    487    1194.26    2386.50    2386.22    65    VDYYGTPTPINQIGAISVPEPR    37A_3   
   
      Matching Genes:  
               gi|115251191|emb|CAJ69022.1|  (ribosome recycling factor [Clostridium difficile 630]) 
           
  Protein Group 37   
      Expression Quality:  
         Score      Num Spectra      Num Peptides      High-Qual Peptides      % Coverage       412    11    8    6    22   
   
      Peptides:   
        Query    Observed    Mr(expt)    Mr(calc)    Score    Peptide    Result File   
		    31    420.82    839.62    839.49    28    APSIPSLR    37_6   
		    226    748.95    1495.88    1495.66    27    EMGDNCSVTILNK +Oxidation (M)    37A_6   
		    304    918.13    1834.25    1833.82    60    GDDISYAGCGLPYYVGK    37A_6   
		    477    813.57    2437.70    2437.15    54    LNGELDSVLLDEIEDFDSWTK    37_8   
		    553    757.87    2270.58    2270.05    50    NSLTNKPTWSPMGSSANHEGR    37_6   
		    263    704.98    1407.94    1407.72    72    SSLIVNTPESFSK    37_6   
		    329    793.02    1584.03    1583.77    73    TLSTGEKENLSYDK    37_6   
		    215    731.95    1461.89    1461.82    48    VLEGGILFYPNLK    37A_6   
   
      Matching Genes:  
               gi|115250843|emb|CAJ68667.1|  (putative pyridine nucleotide-disulfide oxidoreductase [Clostridium difficile 630]) 
           
  Protein Group 38   
      Expression Quality:  
         Score      Num Spectra      Num Peptides      High-Qual Peptides      % Coverage       412    12    8    7    45   
   
      Peptides:   
        Query    Observed    Mr(expt)    Mr(calc)    Score    Peptide    Result File   
		    178    612.89    1223.78    1223.63    46    AGATYVSPFVGR    37A_3   
		    55    425.30    848.58    848.46    40    DFIEVVK    37_3   
		    453    722.52    2164.53    2164.13    67    EISEIVDGPISAEVISLEHK    37A_3   
		    279    720.52    1439.02    1438.73    63    FFIDTANIEEIK    37A_3   
		    97    480.28    958.55    958.52    49    IPMTAEGLK    37A_3   
		    135    558.35    1114.68    1114.61    40    MGADIATVPLK    37A_3   
		    232    559.90    1117.78    1117.64    36    NPIHVLQAAR    37_3   
		    552    873.61    1745.21    1744.98    71    TNVTLIFSAGQALLAAR    37_3   
   
      Matching Genes:  
               gi|115251384|emb|CAJ69216.1|  (putative transaldolase [Clostridium difficile 630]) 
           
  Protein Group 39   
      Expression Quality:  
         Score      Num Spectra      Num Peptides      High-Qual Peptides      % Coverage       406    11    7    5    24   
   
      Peptides:   
        Query    Observed    Mr(expt)    Mr(calc)    Score    Peptide    Result File   
		    370    1040.55    2079.09    2078.92    75    AAEETGLPYAGFDGDQADPR    37A_5   
		    101    592.88    1183.74    1183.56    63    AFTNAQFETR    37A_5   
		    48    455.77    909.53    909.46    34    KFEEVMK    37A_5   
		    165    673.94    1345.87    1345.68    51    LLIEELEDNMK    37A_5   
		    444    1118.74    2235.47    2235.02    30    RAAEETGLPYAGFDGDQADPR    37_5   
		    686    1299.99    3896.96    3896.77    65    TNFGLLENGGCEALDMPAPDFLLCCNNICNQVIK    37_5   
		    294    603.43    1807.28    1806.95    88    VVINDLLAEQYANAFK    37A_5   
   
      Matching Genes:  
               gi|115249403|emb|CAJ67218.1|  (subunit of oxygen-sensitive 2-hydroxyisocaproyl-CoA dehydratase [Clostridium difficile 630]) 
           
  Protein Group 40   
      Expression Quality:  
         Score      Num Spectra      Num Peptides      High-Qual Peptides      % Coverage       402    9    7    6    50   
   
      Peptides:   
        Query    Observed    Mr(expt)    Mr(calc)    Score    Peptide    Result File   
		    184    595.37    1188.72    1188.60    42    DENIKNESIK    37A_2   
		    217    510.29    1018.57    1018.57    54    EIISSITTR    37_2   
		    427    680.38    1358.74    1358.69    80    IECQGEGLVNLK    37_2   
		    192    605.89    1209.77    1209.63    69    LSEVVEFYPK    37A_2   
		    494    734.55    1467.08    1466.87    79    LVNDVELVNVLIK    37_2   
		    394    664.86    1327.71    1327.70    48    NVPVNIWPYAR    37_2   
		    252    566.78    1131.55    1131.49    30    YECEEFKK    37_2   
   
      Matching Genes:  
               gi|115249997|emb|CAJ67817.1|  (hypothetical phage protein [Clostridium difficile 630]) 
              Other Genes Matching Peptide Subset:  
               gi|115251944|emb|CAJ69780.1|  (hypothetical phage protein [Clostridium difficile 630]) 
           
  Protein Group 41   
      Expression Quality:  
         Score      Num Spectra      Num Peptides      High-Qual Peptides      % Coverage       397    10    7    6    24   
   
      Peptides:   
        Query    Observed    Mr(expt)    Mr(calc)    Score    Peptide    Result File   
		    190    428.88    1283.62    1283.55    32    CEEFKTEEGR    37A_4   
		    490    781.26    2340.75    2340.16    52    GLLEEDLTEMNLSSVGDIIHR    37A_4   
		    584    1179.47    2356.93    2356.16    63    GLLEEDLTEMNLSSVGDIIHR +Oxidation (M)    37_4   
		    455    913.17    1824.32    1823.96    100    HSIIVLAEGVGSASDLEK    37_4   
		    452    908.16    1814.30    1813.94    53    TIGLLTSGGDAPGMNAAIR    37_4   
		    459    916.19    1830.36    1829.93    46    TIGLLTSGGDAPGMNAAIR +Oxidation (M)    37_4   
		    85    504.85    1007.68    1007.59    51    VTVLGHVQR    37A_4   
   
      Matching Genes:  
               gi|115252455|emb|CAJ70298.1|  (6-phosphofructokinase [Clostridium difficile 630]) 
           
  Protein Group 42   
      Expression Quality:  
         Score      Num Spectra      Num Peptides      High-Qual Peptides      % Coverage       393    13    6    5    17   
   
      Peptides:   
        Query    Observed    Mr(expt)    Mr(calc)    Score    Peptide    Result File   
		    89    560.44    1118.87    1119.58    42    FEGETLPSLK    37A_5   
		    14    424.25    846.48    846.44    36    ILETTDR    37A_5   
		    93    567.83    1133.65    1133.55    50    MQGLQGSEIR +Oxidation (M)    37A_5   
		    296    896.62    1791.22    1791.87    69    QGADLQASTISQMEVSK    37_5   
		    302    905.08    1808.15    1807.86    92    QGADLQASTISQMEVSK +Oxidation (M)    37_5   
		    488    1204.36    2406.70    2406.20    104    TNVNKDDILVTSGSQQGLDFAGK    37_5   
   
      Matching Genes:  
               gi|115252729|emb|CAJ70573.1|  (putative amino acid aminotransferase [Clostridium difficile 630]) 
           
  Protein Group 43   
      Expression Quality:  
         Score      Num Spectra      Num Peptides      High-Qual Peptides      % Coverage       382    10    5    5    26   
   
      Peptides:   
        Query    Observed    Mr(expt)    Mr(calc)    Score    Peptide    Result File   
		    324    837.07    1672.12    1671.87    52    IADELTQLKDEIER    37A_4   
		    401    839.54    1677.06    1676.84    77    IRDTDVASEMVNLSK    37_4   
		    160    535.31    1068.60    1068.51    43    ISSSTEFNGK    37A_1   
		    572    1159.82    2317.62    2317.11    119    LESTQNNLNNTLENVTAAESR    37_4   
		    381    910.20    1818.39    1817.88    91    TLSLQSANEINNTEER    37A_4   
   
      Matching Genes:  
               gi|115249247|emb|CAJ67060.1|  (flagellin subunit [Clostridium difficile 630]) 
           
  Protein Group 44   
      Expression Quality:  
         Score      Num Spectra      Num Peptides      High-Qual Peptides      % Coverage       377    7    7    6    28   
   
      Peptides:   
        Query    Observed    Mr(expt)    Mr(calc)    Score    Peptide    Result File   
		    415    696.98    1391.94    1391.77    61    GYLVSNKEELLK    37_3   
		    249    579.35    1156.68    1156.56    51    ICCSYTLIK    37_3   
		    39    429.82    857.63    857.52    40    KIEEIVK    37A_3   
		    270    703.20    1404.38    1403.65    36    MDQNLNWLNEK    37A_3   
		    436    710.92    1419.83    1419.65    53    MDQNLNWLNEK +Oxidation (M)    37_3   
		    162    473.28    944.54    944.47    65    TIAAPANCK    37_3   
		    275    712.42    1422.82    1422.62    71    TTGICIDCSSPGR    37A_3   
   
      Matching Genes:  
               gi|115251115|emb|CAJ68946.1|  (conserved hypothetical protein [Clostridium difficile 630]) 
           
  Protein Group 45   
      Expression Quality:  
         Score      Num Spectra      Num Peptides      High-Qual Peptides      % Coverage       356    8    6    4    15   
   
      Peptides:   
        Query    Observed    Mr(expt)    Mr(calc)    Score    Peptide    Result File   
		    338    805.58    1609.15    1608.80    87    INGVSVVSVESDNYK    37_6   
		    409    919.64    1837.27    1836.84    93    KDEFLSYINSTDYDK    37_6   
		    407    690.49    1378.97    1378.69    36    LGGISTDVPMSFR    37_3   
		    78    549.89    1097.76    1097.71    38    LILAVTDIIK    37A_6   
		    343    980.21    1958.40    1957.97    49    TLPVTYDDGTFAGIITMK +Oxidation (M)    37A_6   
		    127    620.90    1239.79    1239.65    53    VDAPEILDNVR    37A_6   
   
      Matching Genes:  
               gi|115249342|emb|CAJ67155.1|  (manganese-dependent inorganic pyrophosphatase [Clostridium difficile 630]) 
           
  Protein Group 46   
      Expression Quality:  
         Score      Num Spectra      Num Peptides      High-Qual Peptides      % Coverage       353    8    7    4    10   
   
      Peptides:   
        Query    Observed    Mr(expt)    Mr(calc)    Score    Peptide    Result File   
		    270    789.53    1577.05    1576.73    97    CGTGLASHEVAQGYK    37_8   
		    422    1179.69    2357.37    2357.08    44    ENFDQLFPADYISEGIDQTR    37A_8   
		    348    1064.05    2126.08    2125.97    29    GNTVNPMELFDQYGADALR +Oxidation (M)    37A_8   
		    188    456.62    1366.83    1366.72    34    LNAHETANKLEK    37A_8   
		    203    704.40    1406.79    1406.70    65    LTEELINEGYAR    37A_8   
		    266    865.66    1729.30    1728.90    54    RYDLPVIQPVDETGK    37A_8   
		    189    456.62    1366.84    1366.64    30    VVHNYPHCWR    37A_8   
   
      Matching Genes:  
               gi|115251669|emb|CAJ69504.1|  (isoleucyl-tRNA synthetase [Clostridium difficile 630]) 
           
  Protein Group 47   
      Expression Quality:  
         Score      Num Spectra      Num Peptides      High-Qual Peptides      % Coverage       352    10    6    4    34   
   
      Peptides:   
        Query    Observed    Mr(expt)    Mr(calc)    Score    Peptide    Result File   
		    343    870.67    1739.32    1738.88    105    AAALDTFETEGLFLNK    37A_4   
		    162    560.87    1119.73    1119.58    25    DYEEPVIKK    37_4   
		    405    844.11    1686.21    1685.88    71    GGLINTGDLIEALESGK    37_4   
		    536    1321.24    2640.46    2640.31    65    GLGANVIAFDQYPNSDLNDILTYK    37A_4   
		    616    1354.90    2707.79    2707.30    61    GYDGISIQQTNYIDNPYIYETLK    37_7   
		    565    973.74    2918.21    2917.34    25    WSEEHKDVQVDIYPENMTEENVVK    37A_5   
   
      Matching Genes:  
               gi|115249400|emb|CAJ67215.1|  ((R)-2-hydroxyisocaproate dehydrogenase [Clostridium difficile 630]) 
           
  Protein Group 48   
      Expression Quality:  
         Score      Num Spectra      Num Peptides      High-Qual Peptides      % Coverage       323    9    5    5    21   
   
      Peptides:   
        Query    Observed    Mr(expt)    Mr(calc)    Score    Peptide    Result File   
		    82    545.35    1088.69    1088.55    49    ATIDAGWLDK    37A_5   
		    459    1139.17    2276.33    2276.15    74    ENLDILYELAEIIGGEVSGSR    37_5   
		    219    771.99    1541.97    1541.87    63    ENVIQTVSLELLGK    37A_5   
		    352    987.17    1972.33    1971.96    94    IHTGLTADCTGLAVAEDTK    37_5   
		    48    444.36    886.70    886.55    43    VSALLLGSK    37_5   
   
      Matching Genes:  
               gi|115250077|emb|CAJ67897.1|  (electron transfer flavoprotein alpha-subunit [Clostridium difficile 630]) 
           
  Protein Group 49   
      Expression Quality:  
         Score      Num Spectra      Num Peptides      High-Qual Peptides      % Coverage       312    5    5    5    5   
   
      Peptides:   
        Query    Observed    Mr(expt)    Mr(calc)    Score    Peptide    Result File   
		    131    636.81    1271.61    1271.60    53    AQANEPLTEDGK    37A_8   
		    127    564.91    1127.80    1127.58    42    DIPNVGDSAIK    37_8   
		    119    550.37    1098.72    1098.64    63    LISSTVIPNR    37_8   
		    246    817.01    1632.00    1631.85    75    STGPYSLVTQQPLGGK    37A_8   
		    240    723.46    1444.90    1444.70    79    VLTDEDQEIEVR    37_8   
   
      Matching Genes:  
               gi|115249070|emb|CAJ66881.1|  (DNA-directed RNA polymerase beta chain [Clostridium difficile 630]) 
           
  Protein Group 50   
      Expression Quality:  
         Score      Num Spectra      Num Peptides      High-Qual Peptides      % Coverage       309    7    5    4    12   
   
      Peptides:   
        Query    Observed    Mr(expt)    Mr(calc)    Score    Peptide    Result File   
		    396    903.16    1804.31    1803.94    72    AGFVVSDSNIKPDNTLK    37_6   
		    55    469.26    936.50    936.42    35    FDESPTNK    37A_6   
		    238    672.49    1342.97    1342.72    79    GQATSIIEVAQAR    37_6   
		    39    443.77    885.52    885.47    77    VGAGNVVDR    37A_6   
		    305    918.61    1835.21    1834.85    46    VSEFMTPMSSIVYANK +2 Oxidation (M)    37A_6   
   
      Matching Genes:  
               gi|115251390|emb|CAJ69222.1|  (inosine-5'-monophosphate dehydrogenase [Clostridium difficile 630]) 
           
  Protein Group 51   
      Expression Quality:  
         Score      Num Spectra      Num Peptides      High-Qual Peptides      % Coverage       309    7    6    4    14   
   
      Peptides:   
        Query    Observed    Mr(expt)    Mr(calc)    Score    Peptide    Result File   
		    120    615.90    1229.78    1229.60    42    HVFEGESNIAK    37A_6   
		    371    864.15    1726.29    1725.92    71    ITLPTASESFLYGISK    37_6   
		    243    788.48    1574.94    1574.86    30    KIGIEAEIVPYESK    37A_6   
		    92    579.31    1156.61    1156.51    33    NEMIESAYGK +Oxidation (M)    37A_6   
		    91    480.31    958.60    958.51    48    QIQQDISK    37_6   
		    362    846.55    1691.08    1690.74    85    SNSNLQTYTYSEER    37_6   
   
      Matching Genes:  
               gi|115251723|emb|CAJ69558.1|  (oligopeptide ABC transporter, substrate-binding protein [Clostridium difficile 630]) 
           
  Protein Group 52   
      Expression Quality:  
         Score      Num Spectra      Num Peptides      High-Qual Peptides      % Coverage       305    8    5    5    68   
   
      Peptides:   
        Query    Observed    Mr(expt)    Mr(calc)    Score    Peptide    Result File   
		    1    402.70    803.38    803.40    42    ACVEVAR    37_1   
		    701    1185.92    3554.74    3553.83    68    AGNLLFVSGQVPLVPETMEVVEGDVQAQTAQSLK    37_2   
		    702    1191.26    3570.77    3569.83    78    AGNLLFVSGQVPLVPETMEVVEGDVQAQTAQSLK +Oxidation (M)    37_2   
		    709    1332.70    2663.38    2663.20    74    DMNEFGAINEVYAEYFGENKPAR    37_1   
		    693    836.54    2506.59    2506.26    43    MKHEVIHTNDAPAALGPYSQAIK +Oxidation (M)    37_1   
   
      Matching Genes:  
               gi|115251566|emb|CAJ69399.1|  (putative translation inhibitor endoribonuclease [Clostridium difficile 630]) 
           
  Protein Group 53   
      Expression Quality:  
         Score      Num Spectra      Num Peptides      High-Qual Peptides      % Coverage       305    13    7    4    20   
   
      Peptides:   
        Query    Observed    Mr(expt)    Mr(calc)    Score    Peptide    Result File   
		    234    669.44    1336.86    1336.61    59    DTDPQSALEYAK    37_6   
		    476    1041.81    2081.60    2080.97    25    EVQASMDEIGVGEFATVSGR    37_6   
		    517    940.17    2817.49    2816.35    28    IVELGGSAIITADHGNAEYMLDPETGK +Oxidation (M)    37A_6   
		    368    572.40    1714.17    1713.86    47    LEKPEEMTGHSLISK +Oxidation (M)    37_6   
		    472    1037.77    2073.53    2073.00    66    VATYDLKPEMSAYELTDK    37_6   
		    482    1045.73    2089.44    2088.99    39    VATYDLKPEMSAYELTDK +Oxidation (M)    37_6   
		    91    575.77    1149.52    1149.52    41    VLCEAMDNAK    37A_6   
   
      Matching Genes:  
               gi|115252228|emb|CAJ70068.1|  (2,3-bisphosphoglycerate-independent phosphoglycerate mutase [Clostridium difficile 630]) 
           
  Protein Group 54   
      Expression Quality:  
         Score      Num Spectra      Num Peptides      High-Qual Peptides      % Coverage       278    6    6    5    21   
   
      Peptides:   
        Query    Observed    Mr(expt)    Mr(calc)    Score    Peptide    Result File   
		    383    577.01    1728.02    1726.88    48    EGITSVGENKPQELAR    37A_3   
		    106    494.36    986.70    986.60    37    EITLLAVTK    37A_3   
		    586    934.13    1866.24    1865.85    60    GLMTMAPFIEDEDEIR    37_3   
		    418    950.12    1898.22    1897.84    42    GLMTMAPFIEDEDEIR +2 Oxidation (M)    37A_3   
		    592    942.14    1882.26    1881.85    47    GLMTMAPFIEDEDEIR +Oxidation (M)    37_3   
		    172    483.28    964.55    964.50    44    VGTSIFGER    37_3   
   
      Matching Genes:  
               gi|115251674|emb|CAJ69509.1|  (putative alanine racemase [Clostridium difficile 630]) 
           
  Protein Group 55   
      Expression Quality:  
         Score      Num Spectra      Num Peptides      High-Qual Peptides      % Coverage       275    9    5    4    14   
   
      Peptides:   
        Query    Observed    Mr(expt)    Mr(calc)    Score    Peptide    Result File   
		    169    613.46    1224.90    1224.65    71    DAVGQSVHTIAK    37_6   
		    327    788.06    1574.10    1574.66    53    EFECYTQEQVDK    37_6   
		    134    422.58    1264.73    1264.65    31    GHSVSIHSNTVK    37A_6   
		    98    586.35    1170.68    1170.61    43    IAGVEIPEDTK    37A_6   
		    390    890.96    1779.91    1780.95    77    IIVIEADGPGEEDIIAK    37_6   
   
      Matching Genes:  
               gi|115251397|emb|CAJ69229.1|  (succinate-semialdehyde dehydrogenase [NAD(P)+] [Clostridium difficile 630]) 
           
  Protein Group 56   
      Expression Quality:  
         Score      Num Spectra      Num Peptides      High-Qual Peptides      % Coverage       262    7    4    4    35   
   
      Peptides:   
        Query    Observed    Mr(expt)    Mr(calc)    Score    Peptide    Result File   
		    247    556.34    1110.66    1110.64    51    ADLDLRPALK    37_2   
		    355    823.55    1645.08    1644.81    60    ALENYFNYETLIR    37A_2   
		    251    564.78    1127.55    1127.54    43    ANVQYYGTGR    37_2   
		    252    656.47    1310.93    1310.73    108    LVAGEGNILVNGR    37A_2   
   
      Matching Genes:  
               gi|115249113|emb|CAJ66924.1|  (30S ribosomal protein S9 [Clostridium difficile 630]) 
           
  Protein Group 57   
      Expression Quality:  
         Score      Num Spectra      Num Peptides      High-Qual Peptides      % Coverage       262    12    5    3    28   
   
      Peptides:   
        Query    Observed    Mr(expt)    Mr(calc)    Score    Peptide    Result File   
		    481    776.00    1549.98    1549.72    39    EVSWLPSYGPEMR    37_3   
		    489    784.00    1565.99    1565.72    33    EVSWLPSYGPEMR +Oxidation (M)    37_3   
		    221    538.36    1074.70    1074.57    67    FKDDVIPGGK    37_3   
		    128    493.85    985.68    985.62    59    IIVNSSLIK    37A_2   
		    477    770.90    2309.69    2309.11    64    TDVDVYYIPANELAAELGNDK    37A_3   
   
      Matching Genes:  
               gi|115249127|emb|CAJ66938.1|  (putative subunit of oxidoreductase [Clostridium difficile 630]) 
           
  Protein Group 58   
      Expression Quality:  
         Score      Num Spectra      Num Peptides      High-Qual Peptides      % Coverage       255    7    4    4    46   
   
      Peptides:   
        Query    Observed    Mr(expt)    Mr(calc)    Score    Peptide    Result File   
		    243    515.26    1028.51    1028.55    70    IANGEIPSTK    37_1   
		    276    567.26    1132.51    1132.55    52    KLPNYEAGQN    37_1   
		    340    738.99    1475.97    1475.67    87    VINNCGSDGGQEVK    37A_1   
		    483    740.55    2218.63    2218.25    46    VLAFNDLNPVAPYHILVVPK    37A_1   
   
      Matching Genes:  
               gi|115251501|emb|CAJ69334.1|  (histidine triad nucleotide-binding protein [Clostridium difficile 630]) 
           
  Protein Group 59   
      Expression Quality:  
         Score      Num Spectra      Num Peptides      High-Qual Peptides      % Coverage       254    7    5    4    14   
   
      Peptides:   
        Query    Observed    Mr(expt)    Mr(calc)    Score    Peptide    Result File   
		    146    581.90    1161.79    1161.59    53    FPLDTEAELK    37_6   
		    581    1206.32    2410.64    2410.15    50    IYGADGVDYTPEADKEIANLEK    37_6   
		    440    969.73    1937.46    1936.99    65    LGNIIVGYSYEGEPVTAR    37_6   
		    367    857.09    1712.17    1711.87    48    SDIEIAQEAKPQDIR    37_6   
		    214    648.44    1294.86    1294.68    38    TTTTIGVADAFAK    37_6   
   
      Matching Genes:  
               gi|115249735|emb|CAJ67552.1|  (formate--tetrahydrofolate ligase [Clostridium difficile 630]) 
           
  Protein Group 60   
      Expression Quality:  
         Score      Num Spectra      Num Peptides      High-Qual Peptides      % Coverage       253    9    5    4    8   
   
      Peptides:   
        Query    Observed    Mr(expt)    Mr(calc)    Score    Peptide    Result File   
		    348    543.37    1627.10    1626.77    41    IKEHNHNVGTCYR    37_7   
		    109    490.80    979.59    979.46    59    LYGEDANAK    37_7   
		    522    1122.87    2243.73    2243.14    77    SLGNGIDPLEIIEQYGADALR    37_7   
		    457    824.53    2470.58    2470.10    33    TTLSDAEVEHEEHDGNFYHIK    37A_7   
		    237    668.52    1335.02    1334.70    43    TYLQWLENIR    37_7   
   
      Matching Genes:  
               gi|115252312|emb|CAJ70153.1|  (valyl-tRNA synthetase [Clostridium difficile 630]) 
           
  Protein Group 61   
      Expression Quality:  
         Score      Num Spectra      Num Peptides      High-Qual Peptides      % Coverage       252    8    5    4    18   
   
      Peptides:   
        Query    Observed    Mr(expt)    Mr(calc)    Score    Peptide    Result File   
		    155    661.40    1320.79    1320.66    33    AVQLHGGYGYTR    37A_5   
		    197    733.49    1464.96    1464.73    48    DLGKPYGVEAAMAK +Oxidation (M)    37A_5   
		    97    578.86    1155.71    1155.64    57    HLVYQAAINK    37A_5   
		    344    647.18    1938.53    1938.08    65    IGIAAQALGLAQGALDETVK    37_5   
		    214    763.00    1523.99    1523.75    49    ITEIYEGTSEVQR    37A_5   
   
      Matching Genes:  
               gi|115250075|emb|CAJ67895.1|  (butyryl-CoA dehydrogenase [Clostridium difficile 630]) 
           
  Protein Group 62   
      Expression Quality:  
         Score      Num Spectra      Num Peptides      High-Qual Peptides      % Coverage       242    7    5    3    51   
   
      Peptides:   
        Query    Observed    Mr(expt)    Mr(calc)    Score    Peptide    Result File   
		    476    713.94    1425.87    1426.66    67    AHNDANMLSLGER    37_2   
		    262    591.84    1181.67    1181.57    27    CAVVSDVFSAK    37_2   
		    236    541.31    1080.61    1080.57    58    EIISYLESK    37_2   
		    700    1159.91    3476.70    3475.61    58    GIECVDYGTNNATDSVDYPVYGEIVANSVINK    37_2   
		    297    702.33    1402.65    1402.67    32    IGLGCDHGGYNLK    37A_2   
   
      Matching Genes:  
               gi|115252540|emb|CAJ70383.1|  (ribose-5-phosphate isomerase 2 [Clostridium difficile 630]) 
           
  Protein Group 63   
      Expression Quality:  
         Score      Num Spectra      Num Peptides      High-Qual Peptides      % Coverage       239    5    4    2    10   
   
      Peptides:   
        Query    Observed    Mr(expt)    Mr(calc)    Score    Peptide    Result File   
		    315    774.49    1546.97    1546.66    73    AVTSSDGMTSDWYK    37_6   
		    65    445.82    889.63    889.50    38    FDINLIR    37_6   
		    507    1084.31    2166.61    2166.08    98    GIIFTGGPNSAYLEDSPTISK    37_6   
		    101    490.28    978.55    978.44    30    TVGVMGDER +Oxidation (M)    37_6   
   
      Matching Genes:  
               gi|115249206|emb|CAJ67019.1|  (GMP synthase [glutamine-hydrolyzing] [Clostridium difficile 630]) 
           
  Protein Group 64   
      Expression Quality:  
         Score      Num Spectra      Num Peptides      High-Qual Peptides      % Coverage       235    4    3    3    24   
   
      Peptides:   
        Query    Observed    Mr(expt)    Mr(calc)    Score    Peptide    Result File   
		    429    706.95    1411.88    1411.68    83    QNFGQVSNSYIR    37_3   
		    639    1081.71    2161.41    2160.97    82    TGEGDGDDEEILVNLETMPK    37_3   
		    564    887.59    1773.17    1772.86    70    VEKDEDFIFYNNLK    37_3   
   
      Matching Genes:  
               gi|115250676|emb|CAJ68500.1|  (tellurium resistance protein [Clostridium difficile 630]) 
           
  Protein Group 65   
      Expression Quality:  
         Score      Num Spectra      Num Peptides      High-Qual Peptides      % Coverage       235    5    5    3    27   
   
      Peptides:   
        Query    Observed    Mr(expt)    Mr(calc)    Score    Peptide    Result File   
		    93    437.77    873.53    873.48    28    AVIIDESK    37_3   
		    77    460.33    918.64    918.52    41    FEILEIR    37A_3   
		    79    465.79    929.57    929.48    49    ISNESPVGK    37A_3   
		    112    501.81    1001.61    1001.58    83    KAVIIDESK    37A_3   
		    632    696.39    2086.14    2084.99    34    VAISFGDLSENAEYDEAKK    37_3   
   
      Matching Genes:  
               gi|115252616|emb|CAJ70459.1|  (transcription elongation factor [Clostridium difficile 630]) 
           
  Protein Group 66   
      Expression Quality:  
         Score      Num Spectra      Num Peptides      High-Qual Peptides      % Coverage       233    6    4    4    34   
   
      Peptides:   
        Query    Observed    Mr(expt)    Mr(calc)    Score    Peptide    Result File   
		    412    664.33    1326.65    1326.62    76    HETVDVPASNMK    37_1   
		    168    545.44    1088.87    1088.62    42    ILLEEGFIR    37A_1   
		    443    694.48    1386.95    1386.81    55    VLNGLGISVISTSK    37_2   
		    186    571.43    1140.84    1140.59    60    VYAANHEIPK    37A_1   
   
      Matching Genes:  
               gi|115249091|emb|CAJ66902.1|  (30S ribosomal protein S8 [Clostridium difficile 630]) 
           
  Protein Group 67   
      Expression Quality:  
         Score      Num Spectra      Num Peptides      High-Qual Peptides      % Coverage       232    8    5    4    31   
   
      Peptides:   
        Query    Observed    Mr(expt)    Mr(calc)    Score    Peptide    Result File   
		    255    573.83    1145.65    1145.58    58    ETEGEIEVLK    37_2   
		    598    1038.65    2075.29    2075.03    48    EYLPQQLSEEELEEIVK    37_2   
		    96    458.34    914.66    914.59    29    KSVVTLIR    37A_2   
		    191    605.88    1209.74    1209.59    56    STISEVGATSMK    37A_2   
		    283    613.83    1225.65    1225.59    41    STISEVGATSMK +Oxidation (M)    37_2   
   
      Matching Genes:  
               gi|115251499|emb|CAJ69332.1|  (putative tRNA binding protein [Clostridium difficile 630]) 
           
  Protein Group 68   
      Expression Quality:  
         Score      Num Spectra      Num Peptides      High-Qual Peptides      % Coverage       230    6    4    3    47   
   
      Peptides:   
        Query    Observed    Mr(expt)    Mr(calc)    Score    Peptide    Result File   
		    383    652.87    1303.72    1303.63    75    EGASKEEADQIK    37_1   
		    115    436.23    870.44    870.44    37    EVVDNAPK    37_1   
		    475    721.26    2160.76    2160.05    55    FGVSASAPVMVAGAAAGGPAAEEK +Oxidation (M)    37A_1   
		    344    749.03    1496.05    1495.74    63    TEFDVVLTDVGSSK    37A_1   
   
      Matching Genes:  
               gi|115249068|emb|CAJ66879.1|  (50S ribosomal protein L7/L12 [Clostridium difficile 630]) 
           
  Protein Group 69   
      Expression Quality:  
         Score      Num Spectra      Num Peptides      High-Qual Peptides      % Coverage       228    5    4    3    11   
   
      Peptides:   
        Query    Observed    Mr(expt)    Mr(calc)    Score    Peptide    Result File   
		    296    899.69    1797.36    1796.95    32    AMDLLVPGVGEIVGGSQR    37A_6   
		    138    638.90    1275.78    1275.64    45    ITTMDLNDIPK +Oxidation (M)    37A_6   
		    100    489.35    976.69    976.53    55    IVNSEFIR    37_6   
		    282    869.55    1737.09    1736.85    96    SIVVEGESDSSYPLQK    37A_6   
   
      Matching Genes:  
               gi|115251299|emb|CAJ69130.1|  (asparaginyl-tRNA synthetase [Clostridium difficile 630]) 
           
  Protein Group 70   
      Expression Quality:  
         Score      Num Spectra      Num Peptides      High-Qual Peptides      % Coverage       227    5    4    4    12   
   
      Peptides:   
        Query    Observed    Mr(expt)    Mr(calc)    Score    Peptide    Result File   
		    123    618.38    1234.75    1234.64    70    GKETVISTADSK    37_5   
		    55    465.31    928.61    928.56    56    IGIEGSILK    37A_5   
		    244    822.59    1643.17    1642.88    54    MLLIPTNEELMIAR    37A_5   
		    245    827.47    1652.92    1651.75    47    YGFHGTSHNYVSQR    37A_5   
   
      Matching Genes:  
               gi|115250207|emb|CAJ68028.1|  (acetate kinase [Clostridium difficile 630]) 
           
  Protein Group 71   
      Expression Quality:  
         Score      Num Spectra      Num Peptides      High-Qual Peptides      % Coverage       225    4    3    2    15   
   
      Peptides:   
        Query    Observed    Mr(expt)    Mr(calc)    Score    Peptide    Result File   
		    372    901.63    1801.24    1801.01    92    ILFVAGGVGSAPVYPQVK    37A_4   
		    354    882.98    1763.94    1763.78    98    NVYVSTDDGTYGFNGR    37A_4   
		    134    609.37    1216.72    1216.68    35    SSQPGQFIIIK    37A_4   
   
      Matching Genes:  
               gi|115250577|emb|CAJ68401.1|  (putative dehydrogenase, electron transfer subunit [Clostridium difficile 630]) 
           
  Protein Group 72   
      Expression Quality:  
         Score      Num Spectra      Num Peptides      High-Qual Peptides      % Coverage       220    5    4    4    10   
   
      Peptides:   
        Query    Observed    Mr(expt)    Mr(calc)    Score    Peptide    Result File   
		    319    777.56    1553.11    1552.73    51    EAYPGDVFYLHSR    37_6   
		    180    676.45    1350.88    1350.79    61    IVEVPVGEALIGR    37A_6   
		    233    770.51    1539.01    1538.81    49    TRPVESEAPGIIDR    37A_6   
		    170    613.91    1225.80    1225.67    59    VVNSLGQPIDGK    37_6   
   
      Matching Genes:  
               gi|115252530|emb|CAJ70373.1|  (ATP synthase alpha chain [Clostridium difficile 630]) 
           
  Protein Group 73   
      Expression Quality:  
         Score      Num Spectra      Num Peptides      High-Qual Peptides      % Coverage       218    5    4    4    16   
   
      Peptides:   
        Query    Observed    Mr(expt)    Mr(calc)    Score    Peptide    Result File   
		    252    707.49    1412.97    1412.74    81    LEELGLGLKPSEE    37A_4   
		    71    473.78    945.54    944.52    44    SLEEVIQK    37A_4   
		    539    1085.76    2169.50    2169.16    46    TENVPIGVLPVDSIYTPVEK    37_4   
		    106    552.79    1103.57    1103.54    47    VSYHVENTR    37A_4   
   
      Matching Genes:  
               gi|115249106|emb|CAJ66917.1|  (DNA-directed RNA polymerase alpha chain [Clostridium difficile 630]) 
           
  Protein Group 74   
      Expression Quality:  
         Score      Num Spectra      Num Peptides      High-Qual Peptides      % Coverage       215    8    4    3    20   
   
      Peptides:   
        Query    Observed    Mr(expt)    Mr(calc)    Score    Peptide    Result File   
		    595    962.14    1922.26    1922.00    73    FPEYAAEVLSTVVEQIK    37_3   
		    166    476.31    950.61    951.59    44    LPVVKPGSR    37_3   
		    325    780.53    1559.04    1558.78    64    TNHDIGMPIYSAIK    37A_3   
		    495    788.52    1575.02    1574.78    34    TNHDIGMPIYSAIK +Oxidation (M)    37_3   
   
      Matching Genes:  
               gi|115249197|emb|CAJ67009.1|  (orotate phosphoribosyltransferase [Clostridium difficile 630]) 
           
  Protein Group 75   
      Expression Quality:  
         Score      Num Spectra      Num Peptides      High-Qual Peptides      % Coverage       211    4    3    3    14   
   
      Peptides:   
        Query    Observed    Mr(expt)    Mr(calc)    Score    Peptide    Result File   
		    399    937.30    1872.59    1872.01    79    EGLLNEDVILESITSIK    37A_4   
		    543    730.91    2189.72    2189.16    60    VNVEDLIYPLFVVEGENIK    37_4   
		    153    619.41    1236.81    1236.56    72    YASNYYGPFR    37A_4   
   
      Matching Genes:  
               gi|115252479|emb|CAJ70322.1|  (delta-aminolevulinic acid dehydratase [Clostridium difficile 630]) 
           
  Protein Group 76   
      Expression Quality:  
         Score      Num Spectra      Num Peptides      High-Qual Peptides      % Coverage       209    10    4    4    52   
   
      Peptides:   
        Query    Observed    Mr(expt)    Mr(calc)    Score    Peptide    Result File   
		    614    1018.15    2034.29    2034.06    56    EQPQIAEVVEVGPGGIVEGK    37_1   
		    298    611.31    1220.60    1220.64    50    IEGQEYTILR    37_1   
		    180    454.71    907.40    907.43    42    MELTVGDK +Oxidation (M)    37_1   
		    165    542.87    1083.72    1083.63    61    TASGIVLPGAAK    37A_1   
   
      Matching Genes:  
               gi|115249203|emb|CAJ67015.1|  (10 kDa chaperonin [Clostridium difficile 630]) 
           
  Protein Group 77   
      Expression Quality:  
         Score      Num Spectra      Num Peptides      High-Qual Peptides      % Coverage       208    6    3    2    28   
   
      Peptides:   
        Query    Observed    Mr(expt)    Mr(calc)    Score    Peptide    Result File   
		    412    905.24    1808.46    1807.91    91    SIMGIMSLGLAQGEELK +2 Oxidation (M)    37A_1   
		    582    896.58    1791.14    1791.91    86    SIMGIMSLGLAQGEELK +Oxidation (M)    37_1   
		    37    416.83    831.65    831.47    31    STVEVVAK    37A_1   
   
      Matching Genes:  
               gi|115251809|emb|CAJ69644.1|  (PTS system, phosphocarrier protein [Clostridium difficile 630]) 
           
  Protein Group 78   
      Expression Quality:  
         Score      Num Spectra      Num Peptides      High-Qual Peptides      % Coverage       201    7    3    3    19   
   
      Peptides:   
        Query    Observed    Mr(expt)    Mr(calc)    Score    Peptide    Result File   
		    413    936.16    1870.30    1869.94    97    FYDASEALTLVSDIAGAK    37A_3   
		    500    790.50    1578.99    1578.83    49    LTENFTALMDAIIK    37_3   
		    189    617.40    1232.79    1232.64    55    SITVASSMGPGVK    37A_3   
   
      Matching Genes:  
               gi|115249066|emb|CAJ66877.1|  (50S ribosomal protein L1 [Clostridium difficile 630]) 
           
  Protein Group 79   
      Expression Quality:  
         Score      Num Spectra      Num Peptides      High-Qual Peptides      % Coverage       198    10    4    3    23   
   
      Peptides:   
        Query    Observed    Mr(expt)    Mr(calc)    Score    Peptide    Result File   
		    321    634.88    1267.74    1266.73    43    ALVPVVVEQTGR    37_3   
		    141    563.33    1124.64    1124.52    32    DNFMSALEAK    37A_3   
		    356    657.40    1312.78    1312.66    58    EYGLIDEVFTK    37_3   
		    441    482.31    1443.92    1443.79    65    IKETLNEILSER    37_3   
   
      Matching Genes:  
               gi|115252361|emb|CAJ70202.1|  (ATP-dependent Clp protease proteolytic subunit [Clostridium difficile 630]) 
           
  Protein Group 80   
      Expression Quality:  
         Score      Num Spectra      Num Peptides      High-Qual Peptides      % Coverage       193    5    4    2    31   
   
      Peptides:   
        Query    Observed    Mr(expt)    Mr(calc)    Score    Peptide    Result File   
		    179    583.37    1164.72    1164.60    47    EQALVEVSYK    37A_2   
		    490    730.49    1458.96    1458.78    88    ILGGGLPYESAVQR    37_2   
		    457    469.92    1406.73    1406.73    33    VKDALEMLSHHK    37_2   
		    146    530.25    1058.48    1058.60    25    VKVETGIDAK    37A_2   
   
      Matching Genes:  
               gi|115249807|emb|CAJ67624.1|  (putative NUDIX-family hydrolase [Clostridium difficile 630]) 
           
  Protein Group 81   
      Expression Quality:  
         Score      Num Spectra      Num Peptides      High-Qual Peptides      % Coverage       191    6    4    3    14   
   
      Peptides:   
        Query    Observed    Mr(expt)    Mr(calc)    Score    Peptide    Result File   
		    367    1035.24    2068.47    2067.97    68    GGPGLGSIQPSQADYFMSTR    37A_5   
		    386    1043.17    2084.32    2083.96    33    GGPGLGSIQPSQADYFMSTR +Oxidation (M)    37_5   
		    208    640.41    1278.80    1278.65    41    VMTSSSSPGVALK +Oxidation (M)    37_7   
		    442    1154.31    2306.61    2306.13    49    YFFGYPITPQSELPEYLSR    37A_5   
   
      Matching Genes:  
               gi|115249125|emb|CAJ66936.1|  (putative oxidoreductase, thiamine diP-binding subunit [Clostridium difficile 630]) 
           
  Protein Group 82   
      Expression Quality:  
         Score      Num Spectra      Num Peptides      High-Qual Peptides      % Coverage       182    6    4    3    22   
   
      Peptides:   
        Query    Observed    Mr(expt)    Mr(calc)    Score    Peptide    Result File   
		    196    486.78    971.54    971.53    40    ILGEGNLEK    37_2   
		    378    440.29    1317.84    1318.74    45    LHELKPAEGAVR    37_2   
		    225    631.39    1260.76    1260.63    70    VGFEGGQMPLAR    37A_2   
		    235    639.38    1276.75    1276.62    27    VGFEGGQMPLAR +Oxidation (M)    37A_2   
   
      Matching Genes:  
               gi|115249096|emb|CAJ66907.1|  (50S ribosomal protein L15 [Clostridium difficile 630]) 
           
  Protein Group 83   
      Expression Quality:  
         Score      Num Spectra      Num Peptides      High-Qual Peptides      % Coverage       180    5    4    3    12   
   
      Peptides:   
        Query    Observed    Mr(expt)    Mr(calc)    Score    Peptide    Result File   
		    334    726.00    1449.98    1449.75    47    GFFPEEELITLR    37_4   
		    169    418.96    1253.86    1253.81    49    KVTGKPTVIIAK    37A_4   
		    149    539.81    1077.61    1077.46    49    MNVCAENPK +Oxidation (M)    37_4   
		    166    563.90    1125.78    1125.71    35    VTGKPTVIIAK    37_4   
   
      Matching Genes:  
               gi|115251377|emb|CAJ69209.1|  (transketolase [Clostridium difficile 630]) 
           
  Protein Group 84   
      Expression Quality:  
         Score      Num Spectra      Num Peptides      High-Qual Peptides      % Coverage       178    4    3    2    13   
   
      Peptides:   
        Query    Observed    Mr(expt)    Mr(calc)    Score    Peptide    Result File   
		    171    607.91    1213.81    1213.67    79    AGENLLSLLER    37A_3   
		    202    517.85    1033.69    1033.57    37    RLDNVVYR    37_3   
		    206    525.33    1048.64    1048.57    62    VSNYGLQLR    37_3   
   
      Matching Genes:  
               gi|115249105|emb|CAJ66916.1|  (30S ribosomal protein S4 [Clostridium difficile 630]) 
           
  Protein Group 85   
      Expression Quality:  
         Score      Num Spectra      Num Peptides      High-Qual Peptides      % Coverage       167    4    3    3    18   
   
      Peptides:   
        Query    Observed    Mr(expt)    Mr(calc)    Score    Peptide    Result File   
		    133    555.82    1109.62    1109.61    50    IAYLFEAVGK    37A_3   
		    116    515.31    1028.61    1028.55    52    LLENLNEGK    37A_3   
		    386    676.91    1351.80    1351.62    65    NLMAGFAGESEAR    37_3   
   
      Matching Genes:  
               gi|115249842|emb|CAJ67659.1|  (rubrerythrin [Clostridium difficile 630]) 
           
  Protein Group 86   
      Expression Quality:  
         Score      Num Spectra      Num Peptides      High-Qual Peptides      % Coverage       166    4    3    2    6   
   
      Peptides:   
        Query    Observed    Mr(expt)    Mr(calc)    Score    Peptide    Result File   
		    203    731.47    1460.93    1460.76    59    DANANIVLNNLYK    37A_7   
		    547    1158.86    2315.72    2315.23    28    IATLIPIDGNDENEYLLLATK    37_7   
		    213    644.91    1287.81    1287.67    79    TNDEVNVVSIAK    37_7   
   
      Matching Genes:  
               gi|115249009|emb|CAJ66820.1|  (DNA gyrase subunit A [Clostridium difficile 630]) 
           
  Protein Group 87   
      Expression Quality:  
         Score      Num Spectra      Num Peptides      High-Qual Peptides      % Coverage       166    3    2    2    15   
   
      Peptides:   
        Query    Observed    Mr(expt)    Mr(calc)    Score    Peptide    Result File   
		    429    706.95    1411.88    1411.68    83    QNFGQVSNSYIR    37_3   
		    566    889.08    1776.14    1775.81    83    TGEGDGDDEQIVVDLSK    37_3   
   
      Matching Genes:  
               gi|115250675|emb|CAJ68499.1|  (tellurium resistance protein [Clostridium difficile 630]) 
           
  Protein Group 88   
      Expression Quality:  
         Score      Num Spectra      Num Peptides      High-Qual Peptides      % Coverage       165    4    3    2    4   
   
      Peptides:   
        Query    Observed    Mr(expt)    Mr(calc)    Score    Peptide    Result File   
		    289    887.26    1772.51    1771.92    47    EDLPVLLPTDVEFTGK    37A_7   
		    1    402.25    802.49    802.41    31    VALEDEK    37_7   
		    324    771.95    1541.88    1541.66    87    YVDSNNENEPFSK    37_7   
   
      Matching Genes:  
               gi|115251575|emb|CAJ69408.1|  (leucyl-tRNA synthetase [Clostridium difficile 630]) 
           
  Protein Group 89   
      Expression Quality:  
         Score      Num Spectra      Num Peptides      High-Qual Peptides      % Coverage       164    5    3    3    6   
   
      Peptides:   
        Query    Observed    Mr(expt)    Mr(calc)    Score    Peptide    Result File   
		    163    602.43    1202.84    1202.63    51    AAQLVEMLGAGK +Oxidation (M)    37_7   
		    289    721.51    1441.00    1440.76    52    GVVDVYPNKPEPK    37_7   
		    421    962.18    1922.34    1921.94    61    SLGLAGVMGGANSEITSNTK +Oxidation (M)    37_7   
   
      Matching Genes:  
               gi|115249716|emb|CAJ67533.1|  (phenylalanyl-tRNA synthetase beta chain [Clostridium difficile 630]) 
           
  Protein Group 90   
      Expression Quality:  
         Score      Num Spectra      Num Peptides      High-Qual Peptides      % Coverage       158    5    3    2    19   
   
      Peptides:   
        Query    Observed    Mr(expt)    Mr(calc)    Score    Peptide    Result File   
		    227    530.34    1058.67    1058.61    56    LASGFPIGLGK    37_2   
		    226    529.28    1056.55    1056.49    34    NISEEYFR    37_2   
		    421    677.39    1352.76    1352.55    68    WDGDNFMSPER    37_2   
   
      Matching Genes:  
               gi|115252075|emb|CAJ69912.1|  (conserved hypothetical protein [Clostridium difficile 630]) 
           
  Protein Group 91   
      Expression Quality:  
         Score      Num Spectra      Num Peptides      High-Qual Peptides      % Coverage       157    3    3    2    37   
   
      Peptides:   
        Query    Observed    Mr(expt)    Mr(calc)    Score    Peptide    Result File   
		    577    589.68    1766.01    1765.89    43    HQKPSAMNQQGGIINK +Oxidation (M)    37_1   
		    166    543.91    1085.81    1085.64    36    KGDTVVVIAGK    37A_1   
		    190    585.99    1169.96    1169.70    78    VLVEGVNVITK    37A_1   
   
      Matching Genes:  
               gi|115249088|emb|CAJ66899.1|  (50S ribosomal protein L24 [Clostridium difficile 630]) 
           
  Protein Group 92   
      Expression Quality:  
         Score      Num Spectra      Num Peptides      High-Qual Peptides      % Coverage       156    4    3    2    31   
   
      Peptides:   
        Query    Observed    Mr(expt)    Mr(calc)    Score    Peptide    Result File   
		    336    734.07    1466.12    1465.78    63    AGSQVSGPVPLPTEK    37A_1   
		    164    541.43    1080.84    1080.62    67    LIDIANPTPK    37A_1   
		    57    425.79    849.57    849.46    26    LLDFSAGK    37A_1   
   
      Matching Genes:  
               gi|115249076|emb|CAJ66887.1|  (30S ribosomal protein S10 [Clostridium difficile 630]) 
           
  Protein Group 93   
      Expression Quality:  
         Score      Num Spectra      Num Peptides      High-Qual Peptides      % Coverage       154    4    3    2    15   
   
      Peptides:   
        Query    Observed    Mr(expt)    Mr(calc)    Score    Peptide    Result File   
		    172    578.84    1155.66    1155.52    66    FFEGDIEGSR    37_4   
		    116    573.40    1144.78    1144.59    39    HFETIANSVK    37A_4   
		    509    824.28    2469.81    2469.18    49    VPVIAGSGSNDTMHSVNLSQEAEK    37A_4   
   
      Matching Genes:  
               gi|115252282|emb|CAJ70123.1|  (dihydrodipicolinate synthase [Clostridium difficile 630]) 
           
  Protein Group 94   
      Expression Quality:  
         Score      Num Spectra      Num Peptides      High-Qual Peptides      % Coverage       149    5    3    2    11   
   
      Peptides:   
        Query    Observed    Mr(expt)    Mr(calc)    Score    Peptide    Result File   
		    245    574.83    1147.66    1147.56    53    NAETQGYPIR    37_3   
		    161    473.27    944.53    944.48    35    NVEVEEVK    37_3   
		    211    532.84    1063.66    1063.58    61    VAVDTPAHVR    37_3   
   
      Matching Genes:  
               gi|115249126|emb|CAJ66937.1|  (putative subunit of oxidoreductase [Clostridium difficile 630]) 
           
  Protein Group 95   
      Expression Quality:  
         Score      Num Spectra      Num Peptides      High-Qual Peptides      % Coverage       149    4    3    1    12   
   
      Peptides:   
        Query    Observed    Mr(expt)    Mr(calc)    Score    Peptide    Result File   
		    263    727.52    1453.02    1452.71    88    ALLDAVNTGDYSSK    37A_4   
		    229    630.38    1258.75    1258.57    36    HMPFVEENEK    37_4   
		    177    685.97    1369.92    1370.65    25    RISYMSEVDQK +Oxidation (M)    37A_7   
   
      Matching Genes:  
               gi|115252096|emb|CAJ69934.1|  (proline iminopeptidase [Clostridium difficile 630]) 
           
  Protein Group 96   
      Expression Quality:  
         Score      Num Spectra      Num Peptides      High-Qual Peptides      % Coverage       146    5    2    2    15   
   
      Peptides:   
        Query    Observed    Mr(expt)    Mr(calc)    Score    Peptide    Result File   
		    340    797.14    1592.26    1591.86    105    APVSNFAYLIDAIAK    37A_3   
		    137    559.80    1117.58    1117.59    41    SEVVSEIVEK    37A_3   
   
      Matching Genes:  
               gi|115249067|emb|CAJ66878.1|  (50S ribosomal protein L10 [Clostridium difficile 630]) 
           
  Protein Group 97   
      Expression Quality:  
         Score      Num Spectra      Num Peptides      High-Qual Peptides      % Coverage       146    4    3    2    6   
   
      Peptides:   
        Query    Observed    Mr(expt)    Mr(calc)    Score    Peptide    Result File   
		    585    1242.40    2482.79    2482.19    49    EALEELGLPYTINEGDGAFYGPK    37_7   
		    88    551.80    1101.59    1101.54    65    EVADNNVSVR    37A_7   
		    33    418.82    835.63    835.46    32    GPHLPSTK    37_7   
   
      Matching Genes:  
               gi|115249589|emb|CAJ67406.1|  (threonyl-tRNA synthetase [Clostridium difficile 630]) 
           
  Protein Group 98   
      Expression Quality:  
         Score      Num Spectra      Num Peptides      High-Qual Peptides      % Coverage       146    5    3    2    14   
   
      Peptides:   
        Query    Observed    Mr(expt)    Mr(calc)    Score    Peptide    Result File   
		    336    962.17    1922.33    1921.92    69    AEGIEATEEEFKAELEK    37A_6   
		    406    1092.27    2182.52    2182.05    52    ELDIDPIDNPDLDIEEISK    37A_6   
		    492    873.10    2616.28    2616.20    25    ELSALDDEFAKDTSEFDSLDELK    37A_6   
   
      Matching Genes:  
               gi|115252362|emb|CAJ70203.1|  (trigger factor [Clostridium difficile 630]) 
           
  Protein Group 99   
      Expression Quality:  
         Score      Num Spectra      Num Peptides      High-Qual Peptides      % Coverage       142    2    2    2    39   
   
      Peptides:   
        Query    Observed    Mr(expt)    Mr(calc)    Score    Peptide    Result File   
		    119    456.34    910.66    910.50    65    GPQAANVVR    37A_1   
		    577    919.06    1836.11    1835.88    77    TLEEGQSVEFEVVDGAK    37_2   
   
      Matching Genes:  
               gi|115250391|emb|CAJ68213.1|  (putative cold shock protein [Clostridium difficile 630]) 
           
  Protein Group 100   
      Expression Quality:  
         Score      Num Spectra      Num Peptides      High-Qual Peptides      % Coverage       141    4    3    1    25   
   
      Peptides:   
        Query    Observed    Mr(expt)    Mr(calc)    Score    Peptide    Result File   
		    514    1094.33    2186.65    2186.10    36    ELDNLEEGVVVTIVDNETAK    37_6   
		    355    822.59    1643.16    1642.86    73    GFIYTLTESKPYPK    37A_3   
		    594    955.67    1909.32    1909.02    32    LQVGTVTNMYTIVETLK    37_3   
   
      Matching Genes:  
               gi|115252732|emb|CAJ70576.1|  (conserved hypothetical protein [Clostridium difficile 630]) 
           
  Protein Group 101   
      Expression Quality:  
         Score      Num Spectra      Num Peptides      High-Qual Peptides      % Coverage       139    4    2    2    13   
   
      Peptides:   
        Query    Observed    Mr(expt)    Mr(calc)    Score    Peptide    Result File   
		    321    775.45    1548.88    1548.81    82    ELSDIIGFTASQIR    37A_3   
		    503    793.06    1584.10    1583.83    57    GVWNFAPLDLEVPK    37_3   
   
      Matching Genes:  
               gi|115249180|emb|CAJ66992.1|  (putative DNA-binding protein [Clostridium difficile 630]) 
           
  Protein Group 102   
      Expression Quality:  
         Score      Num Spectra      Num Peptides      High-Qual Peptides      % Coverage       138    4    3    2    15   
   
      Peptides:   
        Query    Observed    Mr(expt)    Mr(calc)    Score    Peptide    Result File   
		    149    466.29    930.56    930.48    54    ENEVATIR    37_3   
		    326    783.02    1564.02    1563.75    30    TGAIREESFNPSEK    37A_3   
		    26    413.24    824.47    824.40    54    TGEYLSR    37_3   
   
      Matching Genes:  
               gi|115250279|emb|CAJ68101.1|  (elongation factor P [Clostridium difficile 630]) 
           
  Protein Group 103   
      Expression Quality:  
         Score      Num Spectra      Num Peptides      High-Qual Peptides      % Coverage       138    6    3    1    9   
   
      Peptides:   
        Query    Observed    Mr(expt)    Mr(calc)    Score    Peptide    Result File   
		    139    520.35    1038.69    1038.62    32    AEAIVKPGVR    37_4   
		    78    517.80    1033.59    1033.53    31    ATVSGELTEK    37A_7   
		    174    680.98    1359.95    1359.78    75    FLLNLIELGGGSK    37A_5   
   
      Matching Genes:  
               gi|115251664|emb|CAJ69499.1|  (probable peptidase [Clostridium difficile 630]) 
           
  Protein Group 104   
      Expression Quality:  
         Score      Num Spectra      Num Peptides      High-Qual Peptides      % Coverage       135    3    2    2    9   
   
      Peptides:   
        Query    Observed    Mr(expt)    Mr(calc)    Score    Peptide    Result File   
		    374    869.15    1736.28    1735.90    79    VTTAFNDTTIADGIAVK    37_6   
		    427    1103.74    2205.47    2205.04    56    YSFSTIMEDKPGNFAELTR    37_5   
   
      Matching Genes:  
               gi|115251567|emb|CAJ69400.1|  (threonine dehydratase catabolic [Clostridium difficile 630]) 
           
  Protein Group 105   
      Expression Quality:  
         Score      Num Spectra      Num Peptides      High-Qual Peptides      % Coverage       135    5    2    2    12   
   
      Peptides:   
        Query    Observed    Mr(expt)    Mr(calc)    Score    Peptide    Result File   
		    304    748.00    1493.99    1493.85    63    AGVVLNPATPVDTIK    37A_3   
		    186    616.90    1231.79    1231.68    72    LAPSILSADFAK    37A_3   
   
      Matching Genes:  
               gi|115251631|emb|CAJ69464.1|  (ribulose-phosphate 3-epimerase [Clostridium difficile 630]) 
           
  Protein Group 106   
      Expression Quality:  
         Score      Num Spectra      Num Peptides      High-Qual Peptides      % Coverage       130    2    2    2    20   
   
      Peptides:   
        Query    Observed    Mr(expt)    Mr(calc)    Score    Peptide    Result File   
		    508    881.67    2641.98    2641.24    61    LPGEVESYEYGLEYGTDTLEIHK    37A_3   
		    325    633.39    1264.77    1264.67    69    LSNYDVNSLIK    37_2   
   
      Matching Genes:  
               gi|115251797|emb|CAJ69632.1|  (adenine phosphoribosyltransferase [Clostridium difficile 630]) 
           
  Protein Group 107   
      Expression Quality:  
         Score      Num Spectra      Num Peptides      High-Qual Peptides      % Coverage       128    5    2    1    21   
   
      Peptides:   
        Query    Observed    Mr(expt)    Mr(calc)    Score    Peptide    Result File   
		    579    935.60    1869.18    1868.91    95    LAGEGGLFFVDQEFANR    37_2   
		    590    669.07    2004.20    2004.03    33    VTALINNLAAVANSESDFR    37_2   
   
      Matching Genes:  
               gi|115251248|emb|CAJ69079.1|  (ferritin [Clostridium difficile 630]) 
           
  Protein Group 108   
      Expression Quality:  
         Score      Num Spectra      Num Peptides      High-Qual Peptides      % Coverage       124    3    2    2    12   
   
      Peptides:   
        Query    Observed    Mr(expt)    Mr(calc)    Score    Peptide    Result File   
		    483    778.56    1555.11    1554.83    74    DVIDAVSISLNAPNK    37_3   
		    197    508.34    1014.67    1013.58    50    INELIDVAK    37_3   
   
      Matching Genes:  
               gi|115249398|emb|CAJ67213.1|  (Radical SAM-superfamily protein [Clostridium difficile 630]) 
           
  Protein Group 109   
      Expression Quality:  
         Score      Num Spectra      Num Peptides      High-Qual Peptides      % Coverage       124    4    2    2    15   
   
      Peptides:   
        Query    Observed    Mr(expt)    Mr(calc)    Score    Peptide    Result File   
		    258    584.87    1167.73    1167.58    56    ESVDFPIYAK    37_2   
		    219    624.36    1246.71    1246.60    68    GIEVEDVGTNSK    37A_2   
   
      Matching Genes:  
               gi|115251375|emb|CAJ69207.1|  (ribose-5-phosphate isomerase 1 [Clostridium difficile 630]) 
           
  Protein Group 110   
      Expression Quality:  
         Score      Num Spectra      Num Peptides      High-Qual Peptides      % Coverage       122    4    2    1    21   
   
      Peptides:   
        Query    Observed    Mr(expt)    Mr(calc)    Score    Peptide    Result File   
		    337    738.12    1474.23    1473.79    85    IGVIGGGSITYPNAR    37A_1   
		    138    478.30    954.59    954.47    37    TYSEGAISK    37A_1   
   
      Matching Genes:  
               gi|115251040|emb|CAJ68871.1|  (putative decarboxylase [Clostridium difficile 630]) 
           
  Protein Group 111   
      Expression Quality:  
         Score      Num Spectra      Num Peptides      High-Qual Peptides      % Coverage       120    4    3    1    13   
   
      Peptides:   
        Query    Observed    Mr(expt)    Mr(calc)    Score    Peptide    Result File   
		    263    654.86    1307.71    1307.60    57    AATPDVNSENYK    37_4   
		    189    609.97    1217.92    1217.64    35    NSVAYPNLNVK    37_4   
		    368    897.10    1792.19    1791.95    28    QAILASIDYNGPVYIR    37A_4   
   
      Matching Genes:  
               gi|115251376|emb|CAJ69208.1|  (transketolase [Clostridium difficile 630]) 
           
  Protein Group 112   
      Expression Quality:  
         Score      Num Spectra      Num Peptides      High-Qual Peptides      % Coverage       120    5    2    1    16   
   
      Peptides:   
        Query    Observed    Mr(expt)    Mr(calc)    Score    Peptide    Result File   
		    344    534.00    1598.97    1598.72    31    NLSDNHESQEAEVK    37A_3   
		    292    738.94    1475.87    1475.76    89    VEAGIYNEILNNK    37A_3   
   
      Matching Genes:  
               gi|115252635|emb|CAJ70478.1|  (putative preprotein translocase [Clostridium difficile 630]) 
           
  Protein Group 113   
      Expression Quality:  
         Score      Num Spectra      Num Peptides      High-Qual Peptides      % Coverage       114    2    2    2    10   
   
      Peptides:   
        Query    Observed    Mr(expt)    Mr(calc)    Score    Peptide    Result File   
		    251    580.92    1159.82    1159.67    48    MIIFPAIDIK    37_3   
		    535    844.05    1686.08    1685.83    66    VNVYYDNPLEVAYK    37_3   
   
      Matching Genes:  
               gi|115250593|emb|CAJ68417.1|  (putative 1-(5-phosphoribosyl)-5-[(5-phosphoribosylamino)methylidene amino] imidazole-4-carboxamide isomerase [Clostridium difficile 630]) 
           
  Protein Group 114   
      Expression Quality:  
         Score      Num Spectra      Num Peptides      High-Qual Peptides      % Coverage       114    4    3    1    12   
   
      Peptides:   
        Query    Observed    Mr(expt)    Mr(calc)    Score    Peptide    Result File   
		    76    459.79    917.57    917.48    37    GENIGTTVK    37A_3   
		    205    632.35    1262.68    1262.61    46    NVDAVYDKDPK    37A_3   
		    164    475.30    948.59    948.47    31    VQTAIDMR +Oxidation (M)    37_3   
   
      Matching Genes:  
               gi|115251192|emb|CAJ69023.1|  (uridylate kinase [Clostridium difficile 630]) 
           
  Protein Group 115   
      Expression Quality:  
         Score      Num Spectra      Num Peptides      High-Qual Peptides      % Coverage       113    3    2    2    5   
   
      Peptides:   
        Query    Observed    Mr(expt)    Mr(calc)    Score    Peptide    Result File   
		    303    686.50    1370.99    1371.70    60    GEADTISAAPVSVR    37_4   
		    123    591.81    1181.61    1181.59    53    LGSISDSVYNK    37A_4   
   
      Matching Genes:  
               gi|115251246|emb|CAJ69077.1|  (cell surface protein [Clostridium difficile 630]) 
           
  Protein Group 116   
      Expression Quality:  
         Score      Num Spectra      Num Peptides      High-Qual Peptides      % Coverage       111    2    2    1    12   
   
      Peptides:   
        Query    Observed    Mr(expt)    Mr(calc)    Score    Peptide    Result File   
		    551    915.24    2742.70    2742.33    74    GIMTVEDALMAVEAGVDAIVVSNHGGR +2 Oxidation (M)    37A_4   
		    288    779.93    1557.85    1557.75    37    VLDCTPGACEVLPK    37A_4   
   
      Matching Genes:  
               gi|115250297|emb|CAJ68119.1|  (putative FMN-dependent dehydrogenase [Clostridium difficile 630]) 
           
  Protein Group 117   
      Expression Quality:  
         Score      Num Spectra      Num Peptides      High-Qual Peptides      % Coverage       110    3    2    1    21   
   
      Peptides:   
        Query    Observed    Mr(expt)    Mr(calc)    Score    Peptide    Result File   
		    425    711.51    2131.50    2131.16    81    IAQMIVKPIYDINIEEVK +Oxidation (M)    37A_2   
		    320    489.97    1466.89    1466.75    29    LNDDAIIPNFAHK    37A_2   
   
      Matching Genes:  
               gi|115251455|emb|CAJ69288.1|  (deoxyuridine 5'-triphosphate nucleotidohydrolase [Clostridium difficile 630]) 
           
  Protein Group 118   
      Expression Quality:  
         Score      Num Spectra      Num Peptides      High-Qual Peptides      % Coverage       107    2    2    1    10   
   
      Peptides:   
        Query    Observed    Mr(expt)    Mr(calc)    Score    Peptide    Result File   
		    411    854.55    1707.09    1705.90    38    AAINSLTQNIATQYAK    37_4   
		    233    634.39    1266.77    1267.64    69    SEELAHEVINK    37_4   
   
      Matching Genes:  
               gi|115249069|emb|CAJ66880.1|  (NADP-dependent 7-alpha-hydroxysteroid dehydrogenase [Clostridium difficile 630]) 
           
  Protein Group 119   
      Expression Quality:  
         Score      Num Spectra      Num Peptides      High-Qual Peptides      % Coverage       107    3    2    1    3   
   
      Peptides:   
        Query    Observed    Mr(expt)    Mr(calc)    Score    Peptide    Result File   
		    245    674.43    1346.85    1346.69    74    AESDKIDESLLK    37_7   
		    113    498.33    994.65    994.50    33    NLESYIEK    37_7   
   
      Matching Genes:  
               gi|115251485|emb|CAJ69318.1|  (glycyl-tRNA synthetase beta chain [Clostridium difficile 630]) 
           
  Protein Group 120   
      Expression Quality:  
         Score      Num Spectra      Num Peptides      High-Qual Peptides      % Coverage       103    2    2    1    9   
   
      Peptides:   
        Query    Observed    Mr(expt)    Mr(calc)    Score    Peptide    Result File   
		    296    604.05    1809.14    1808.96    37    TKPHVNIGTIGHVDHGK    37A_5   
		    417    1083.27    2164.52    2164.04    66    YQLGEAVDFANIDKAPEER    37_5   
   
      Matching Genes:  
               gi|115249061|emb|CAJ66872.1|  (elongation factor TU [Clostridium difficile 630]) 
              Other Genes Matching Peptide Subset:  
               gi|115249075|emb|CAJ66886.1|  (elongation factor TU [Clostridium difficile 630]) 
           
  Protein Group 121   
      Expression Quality:  
         Score      Num Spectra      Num Peptides      High-Qual Peptides      % Coverage       103    5    2    2    4   
   
      Peptides:   
        Query    Observed    Mr(expt)    Mr(calc)    Score    Peptide    Result File   
		    256    699.02    1396.02    1395.74    49    AGEDQQPIILANK    37_6   
		    330    777.01    1552.01    1551.69    54    HNAPTEPDNSGSAAGK    37_7   
   
      Matching Genes:  
               gi|115251837|emb|CAJ69672.1|  (cell surface protein [Clostridium difficile 630]) 
           
  Protein Group 122   
      Expression Quality:  
         Score      Num Spectra      Num Peptides      High-Qual Peptides      % Coverage       103    2    2    1    9   
   
      Peptides:   
        Query    Observed    Mr(expt)    Mr(calc)    Score    Peptide    Result File   
		    497    1003.17    2004.33    2003.92    70    DVYACCTHGVLSGPAIER    37_4   
		    119    579.26    1156.51    1156.54    33    MIFSNESVSK +Oxidation (M)    37A_4   
   
      Matching Genes:  
               gi|115252575|emb|CAJ70418.1|  (ribose-phosphate pyrophosphokinase [Clostridium difficile 630]) 
           
  Protein Group 123   
      Expression Quality:  
         Score      Num Spectra      Num Peptides      High-Qual Peptides      % Coverage       98    3    2    1    7   
   
      Peptides:   
        Query    Observed    Mr(expt)    Mr(calc)    Score    Peptide    Result File   
		    73    444.80    887.59    888.46    25    GDVTVVDGK    37A_2   
		    287    778.95    1555.89    1555.76    73    SIQAIDSHTAGEATR    37A_4   
   
      Matching Genes:  
               gi|115252294|emb|CAJ70135.1|  (putative proline racemase [Clostridium difficile 630]) 
           
  Protein Group 124   
      Expression Quality:  
         Score      Num Spectra      Num Peptides      High-Qual Peptides      % Coverage       96    2    2    1    12   
   
      Peptides:   
        Query    Observed    Mr(expt)    Mr(calc)    Score    Peptide    Result File   
		    259    588.36    1174.71    1174.66    59    TPEQLSIFLK    37_2   
		    156    551.34    1100.66    1100.58    37    VLEDDKNLR    37A_2   
   
      Matching Genes:  
               gi|115249226|emb|CAJ67039.1|  (phosphoribosylaminoimidazole carboxylase catalytic subunit [Clostridium difficile 630]) 
           
  Protein Group 125   
      Expression Quality:  
         Score      Num Spectra      Num Peptides      High-Qual Peptides      % Coverage       96    2    1    1    9   
   
      Peptides:   
        Query    Observed    Mr(expt)    Mr(calc)    Score    Peptide    Result File   
		    478    715.91    1429.81    1429.74    96    VTVDENTIGQINK    37_2   
   
      Matching Genes:  
               gi|115251795|emb|CAJ69630.1|  (putative D-tyrosyl-tRNA protein [Clostridium difficile 630]) 
           
  Protein Group 126   
      Expression Quality:  
         Score      Num Spectra      Num Peptides      High-Qual Peptides      % Coverage       93    4    2    2    13   
   
      Peptides:   
        Query    Observed    Mr(expt)    Mr(calc)    Score    Peptide    Result File   
		    495    491.57    1471.69    1470.80    45    IYSSLYLEDLKK    37_2   
		    5    409.77    817.53    817.42    48    LSDGLGEK    37A_2   
   
      Matching Genes:  
               gi|115250371|emb|CAJ68193.1|  (MarR-family transcriptional regulator [Clostridium difficile 630]) 
           
  Protein Group 127   
      Expression Quality:  
         Score      Num Spectra      Num Peptides      High-Qual Peptides      % Coverage       91    4    2    2    13   
   
      Peptides:   
        Query    Observed    Mr(expt)    Mr(calc)    Score    Peptide    Result File   
		    163    458.23    914.44    914.45    44    AEINPDTR    37_2   
		    26    420.77    839.52    839.49    47    IAGVDLPR    37A_2   
   
      Matching Genes:  
               gi|115249103|emb|CAJ66914.1|  (30S ribosomal protein S13 [Clostridium difficile 630]) 
           
  Protein Group 128   
      Expression Quality:  
         Score      Num Spectra      Num Peptides      High-Qual Peptides      % Coverage       91    3    2    1    18   
   
      Peptides:   
        Query    Observed    Mr(expt)    Mr(calc)    Score    Peptide    Result File   
		    560    574.71    1721.12    1720.92    64    KPEEVISHAVSGMLPK    37_2   
		    190    604.87    1207.72    1207.62    27    KWYLVDAEGK    37A_2   
   
      Matching Genes:  
               gi|115249112|emb|CAJ66923.1|  (50S ribosomal protein L13 [Clostridium difficile 630]) 
           
  Protein Group 129   
      Expression Quality:  
         Score      Num Spectra      Num Peptides      High-Qual Peptides      % Coverage       91    2    1    1    22   
   
      Peptides:   
        Query    Observed    Mr(expt)    Mr(calc)    Score    Peptide    Result File   
		    511    745.43    1488.85    1488.71    91    EAEEGCPVSAITVK    37_1   
   
      Matching Genes:  
               gi|115252670|emb|CAJ70513.1|  (ferredoxin [Clostridium difficile 630]) 
           
  Protein Group 130   
      Expression Quality:  
         Score      Num Spectra      Num Peptides      High-Qual Peptides      % Coverage       91    2    2    1    32   
   
      Peptides:   
        Query    Observed    Mr(expt)    Mr(calc)    Score    Peptide    Result File   
		    452    685.26    2052.74    2052.02    29    NYELVYVVKPNSDEEVR    37A_1   
		    308    694.02    1386.02    1385.78    62    VKEVVATDGEIVK    37A_1   
   
      Matching Genes:  
               gi|115252728|emb|CAJ70572.1|  (30S ribosomal protein S6 [Clostridium difficile 630]) 
           
  Protein Group 131   
      Expression Quality:  
         Score      Num Spectra      Num Peptides      High-Qual Peptides      % Coverage       89    1    1    1    7   
   
      Peptides:   
        Query    Observed    Mr(expt)    Mr(calc)    Score    Peptide    Result File   
		    311    762.46    1522.91    1522.75    89    VDSVEGYTVGQEIK    37A_3   
   
      Matching Genes:  
               gi|115249077|emb|CAJ66888.1|  (50S ribosomal protein L3 [Clostridium difficile 630]) 
           
  Protein Group 132   
      Expression Quality:  
         Score      Num Spectra      Num Peptides      High-Qual Peptides      % Coverage       88    4    2    1    4   
   
      Peptides:   
        Query    Observed    Mr(expt)    Mr(calc)    Score    Peptide    Result File   
		    83    462.33    922.64    922.49    30    FPYEIVR    37_6   
		    284    486.29    1455.84    1455.65    58    TESMHGAGSPQAQR    37_6   
   
      Matching Genes:  
               gi|115251396|emb|CAJ69228.1|  (gamma-aminobutyrate metabolism dehydratase/isomerase [includes: 4-hydroxybutyryl-coa dehydratase; vinylacetyl-coa-delta-isomerase] [Clostridium difficile 630]) 
           
  Protein Group 133   
      Expression Quality:  
         Score      Num Spectra      Num Peptides      High-Qual Peptides      % Coverage       87    2    1    1    12   
   
      Peptides:   
        Query    Observed    Mr(expt)    Mr(calc)    Score    Peptide    Result File   
		    393    863.67    1725.32    1724.79    87    IVDEYDYGYNAIYK    37A_1   
   
      Matching Genes:  
               gi|115249824|emb|CAJ67641.1|  (hypothetical protein [Clostridium difficile 630]) 
           
  Protein Group 134   
      Expression Quality:  
         Score      Num Spectra      Num Peptides      High-Qual Peptides      % Coverage       86    2    2    1    3   
   
      Peptides:   
        Query    Observed    Mr(expt)    Mr(calc)    Score    Peptide    Result File   
		    295    916.04    1830.07    1829.79    28    TLVTCDSFGSHYSNDK    37A_7   
		    50    459.37    916.72    916.52    58    VVASETAIK    37A_7   
   
      Matching Genes:  
               gi|115250664|emb|CAJ68488.1|  (putative nitric oxide reductase flavoprotein [Clostridium difficile 630]) 
           
  Protein Group 135   
      Expression Quality:  
         Score      Num Spectra      Num Peptides      High-Qual Peptides      % Coverage       85    3    2    1    25   
   
      Peptides:   
        Query    Observed    Mr(expt)    Mr(calc)    Score    Peptide    Result File   
		    237    628.98    1255.94    1255.71    59    IIQLDSNVINK    37A_1   
		    93    440.30    878.59    878.42    26    VSFNQER    37A_1   
   
      Matching Genes:  
               gi|115249124|emb|CAJ66935.1|  (ferredoxin [Clostridium difficile 630]) 
           
  Protein Group 136   
      Expression Quality:  
         Score      Num Spectra      Num Peptides      High-Qual Peptides      % Coverage       84    2    2    1    14   
   
      Peptides:   
        Query    Observed    Mr(expt)    Mr(calc)    Score    Peptide    Result File   
		    590    625.44    1873.29    1872.99    39    GVEELEMISGQKPVITK +Oxidation (M)    37_3   
		    88    435.81    869.61    869.53    45    LVSVSLPR    37_3   
   
      Matching Genes:  
               gi|115249089|emb|CAJ66900.1|  (50S ribosomal protein L5 [Clostridium difficile 630]) 
           
  Protein Group 137   
      Expression Quality:  
         Score      Num Spectra      Num Peptides      High-Qual Peptides      % Coverage       84    1    1    1    4   
   
      Peptides:   
        Query    Observed    Mr(expt)    Mr(calc)    Score    Peptide    Result File   
		    298    909.14    1816.26    1815.97    84    ILVINNPSNPTGSVYTK    37A_5   
   
      Matching Genes:  
               gi|115249115|emb|CAJ66926.1|  (aspartate aminotransferase [Clostridium difficile 630]) 
           
  Protein Group 138   
      Expression Quality:  
         Score      Num Spectra      Num Peptides      High-Qual Peptides      % Coverage       83    1    1    1    5   
   
      Peptides:   
        Query    Observed    Mr(expt)    Mr(calc)    Score    Peptide    Result File   
		    397    832.57    1663.13    1662.82    83    TEGYSEGNVPLQTLR    37_4   
   
      Matching Genes:  
               gi|115249083|emb|CAJ66894.1|  (30S ribosomal protein S3 [Clostridium difficile 630]) 
           
  Protein Group 139   
      Expression Quality:  
         Score      Num Spectra      Num Peptides      High-Qual Peptides      % Coverage       83    3    2    1    25   
   
      Peptides:   
        Query    Observed    Mr(expt)    Mr(calc)    Score    Peptide    Result File   
		    622    698.03    2091.08    2090.83    30    LVEFPSDHTCSHDGCGHH    37_1   
		    328    479.62    1435.85    1435.63    53    SLDNGDLDHEHGK    37A_1   
   
      Matching Genes:  
               gi|115250736|emb|CAJ68560.1|  (putative dinitrogenase iron-molybdenum cofactor [Clostridium difficile 630]) 
           
  Protein Group 140   
      Expression Quality:  
         Score      Num Spectra      Num Peptides      High-Qual Peptides      % Coverage       81    1    1    1    5   
   
      Peptides:   
        Query    Observed    Mr(expt)    Mr(calc)    Score    Peptide    Result File   
		    391    930.05    1858.09    1857.88    81    TIDEDESGALNPELVEK    37A_4   
   
      Matching Genes:  
               gi|115251648|emb|CAJ69481.1|  (low-specificity L-threonine aldolase [Clostridium difficile 630]) 
           
  Protein Group 141   
      Expression Quality:  
         Score      Num Spectra      Num Peptides      High-Qual Peptides      % Coverage       79    2    2    1    11   
   
      Peptides:   
        Query    Observed    Mr(expt)    Mr(calc)    Score    Peptide    Result File   
		    43    417.77    833.52    833.46    33    LSVSATTR    37_3   
		    332    788.51    1575.00    1574.76    46    TGEVDGVNYFFISK    37A_3   
   
      Matching Genes:  
               gi|115251645|emb|CAJ69478.1|  (guanylate kinase [Clostridium difficile 630]) 
           
  Protein Group 142   
      Expression Quality:  
         Score      Num Spectra      Num Peptides      High-Qual Peptides      % Coverage       78    2    1    1    4   
   
      Peptides:   
        Query    Observed    Mr(expt)    Mr(calc)    Score    Peptide    Result File   
		    180    693.01    1384.01    1383.80    78    ILITGSPIGGISEK    37A_5   
   
      Matching Genes:  
               gi|115250793|emb|CAJ68617.1|  (putative 2-hydroxyacyl-CoA dehydratase [Clostridium difficile 630]) 
           
  Protein Group 143   
      Expression Quality:  
         Score      Num Spectra      Num Peptides      High-Qual Peptides      % Coverage       78    2    2    1    7   
   
      Peptides:   
        Query    Observed    Mr(expt)    Mr(calc)    Score    Peptide    Result File   
		    320    778.02    1554.03    1553.78    32    GIPVSIGTDGAPSNNR    37_6   
		    343    817.54    1633.06    1632.75    46    SVMDTGDGLPEAWQK    37_6   
   
      Matching Genes:  
               gi|115251756|emb|CAJ69591.1|  (putative amidohydrolas [Clostridium difficile 630]) 
           
  Protein Group 144   
      Expression Quality:  
         Score      Num Spectra      Num Peptides      High-Qual Peptides      % Coverage       76    3    2    1    17   
   
      Peptides:   
        Query    Observed    Mr(expt)    Mr(calc)    Score    Peptide    Result File   
		    76    425.72    849.43    849.46    30    VFGVSVDK    37_1   
		    241    512.24    1022.46    1022.50    46    VNTLNYDGK    37_1   
   
      Matching Genes:  
               gi|115249079|emb|CAJ66890.1|  (50S ribosomal protein L23 [Clostridium difficile 630]) 
           
  Protein Group 145   
      Expression Quality:  
         Score      Num Spectra      Num Peptides      High-Qual Peptides      % Coverage       75    1    1    1    8   
   
      Peptides:   
        Query    Observed    Mr(expt)    Mr(calc)    Score    Peptide    Result File   
		    439    716.51    1431.00    1430.76    75    VTSTGIVNGVIEDK    37_3   
   
      Matching Genes:  
               gi|115252223|emb|CAJ70063.1|  (putative phosphatidylethanolamine-binding regulatory protein [Clostridium difficile 630]) 
           
  Protein Group 146   
      Expression Quality:  
         Score      Num Spectra      Num Peptides      High-Qual Peptides      % Coverage       72    1    1    1    4   
   
      Peptides:   
        Query    Observed    Mr(expt)    Mr(calc)    Score    Peptide    Result File   
		    139    611.34    1220.67    1220.64    72    AYGANLVLTDGK    37A_4   
   
      Matching Genes:  
               gi|115250635|emb|CAJ68459.1|  (putative O-acetylserine sulfhydrylase [Clostridium difficile 630]) 
           
  Protein Group 147   
      Expression Quality:  
         Score      Num Spectra      Num Peptides      High-Qual Peptides      % Coverage       71    3    2    0    18   
   
      Peptides:   
        Query    Observed    Mr(expt)    Mr(calc)    Score    Peptide    Result File   
		    188    479.73    957.44    957.51    38    AIEQEQLK    37_2   
		    284    687.44    1372.87    1372.66    33    NEVPNFGPGDTVK    37A_2   
   
      Matching Genes:  
               gi|115250291|emb|CAJ68113.1|  (50S ribosomal protein L19 [Clostridium difficile 630]) 
           
  Protein Group 148   
      Expression Quality:  
         Score      Num Spectra      Num Peptides      High-Qual Peptides      % Coverage       70    2    2    0    11   
   
      Peptides:   
        Query    Observed    Mr(expt)    Mr(calc)    Score    Peptide    Result File   
		    261    687.98    1373.94    1373.69    34    FIVGYGIDYAEK    37A_3   
		    210    504.25    1006.49    1006.50    36    MLTEEQIK +Oxidation (M)    37_2   
   
      Matching Genes:  
               gi|115251742|emb|CAJ69577.1|  (putative phosphoribosyltransferase [Clostridium difficile 630]) 
           
  Protein Group 149   
      Expression Quality:  
         Score      Num Spectra      Num Peptides      High-Qual Peptides      % Coverage       69    1    1    1    4   
   
      Peptides:   
        Query    Observed    Mr(expt)    Mr(calc)    Score    Peptide    Result File   
		    165    622.90    1243.79    1243.68    69    TGAASGAAIDVLAK    37A_4   
   
      Matching Genes:  
               gi|115249560|emb|CAJ67377.1|  (putative ornithine cyclodeaminase [Clostridium difficile 630]) 
           
  Protein Group 150   
      Expression Quality:  
         Score      Num Spectra      Num Peptides      High-Qual Peptides      % Coverage       68    1    1    1    6   
   
      Peptides:   
        Query    Observed    Mr(expt)    Mr(calc)    Score    Peptide    Result File   
		    536    1078.23    2154.46    2153.99    68    CIWGEGDGSTLTVVDTPYGK    37_4   
   
      Matching Genes:  
               gi|115251895|emb|CAJ69730.1|  (nitrilase (carbon-nitrogen hydrolase) [Clostridium difficile 630]) 
           
  Protein Group 151   
      Expression Quality:  
         Score      Num Spectra      Num Peptides      High-Qual Peptides      % Coverage       67    2    1    1    13   
   
      Peptides:   
        Query    Observed    Mr(expt)    Mr(calc)    Score    Peptide    Result File   
		    444    677.39    1352.77    1352.71    67    VQLVGFGTFETR    37_1   
   
      Matching Genes:  
               gi|115252557|emb|CAJ70400.1|  (DNA-binding protein HU [Clostridium difficile 630]) 
           
  Protein Group 152   
      Expression Quality:  
         Score      Num Spectra      Num Peptides      High-Qual Peptides      % Coverage       66    2    2    0    8   
   
      Peptides:   
        Query    Observed    Mr(expt)    Mr(calc)    Score    Peptide    Result File   
		    171    576.31    1150.61    1150.57    33    IASFSQQSQR    37_4   
		    117    576.33    1150.64    1149.60    33    LISHTPEPEK    37A_4   
   
      Matching Genes:  
               gi|115249057|emb|CAJ66868.1|  (putative thymidylate synthase [Clostridium difficile 630]) 
           
  Protein Group 153   
      Expression Quality:  
         Score      Num Spectra      Num Peptides      High-Qual Peptides      % Coverage       66    2    2    1    6   
   
      Peptides:   
        Query    Observed    Mr(expt)    Mr(calc)    Score    Peptide    Result File   
		    78    533.86    1065.70    1065.59    41    ASVVHALNQK    37A_5   
		    150    436.22    1305.64    1305.62    25    HSSEEIGKYEK    37A_5   
   
      Matching Genes:  
               gi|115249122|emb|CAJ66933.1|  (butyrate kinase [Clostridium difficile 630]) 
           
  Protein Group 154   
      Expression Quality:  
         Score      Num Spectra      Num Peptides      High-Qual Peptides      % Coverage       66    2    1    1    5   
   
      Peptides:   
        Query    Observed    Mr(expt)    Mr(calc)    Score    Peptide    Result File   
		    191    503.79    1005.56    1005.51    66    VPGATYAEAK    37_3   
   
      Matching Genes:  
               gi|115251487|emb|CAJ69320.1|  (conserved hypothetical protein [Clostridium difficile 630]) 
           
  Protein Group 155   
      Expression Quality:  
         Score      Num Spectra      Num Peptides      High-Qual Peptides      % Coverage       65    1    1    1    2   
   
      Peptides:   
        Query    Observed    Mr(expt)    Mr(calc)    Score    Peptide    Result File   
		    237    672.44    1342.86    1342.67    65    VVNINANEVDEK    37_6   
   
      Matching Genes:  
               gi|115251568|emb|CAJ69401.1|  (L-aspartate-beta-decarboxylase [Clostridium difficile 630]) 
           
  Protein Group 156   
      Expression Quality:  
         Score      Num Spectra      Num Peptides      High-Qual Peptides      % Coverage       63    1    1    1    9   
   
      Peptides:   
        Query    Observed    Mr(expt)    Mr(calc)    Score    Peptide    Result File   
		    487    781.07    1560.12    1559.86    63    FINGILGSVVDEIGK    37_3   
   
      Matching Genes:  
               gi|115250234|emb|CAJ68055.1|  (N utilization substance protein B [Clostridium difficile 630]) 
           
  Protein Group 157   
      Expression Quality:  
         Score      Num Spectra      Num Peptides      High-Qual Peptides      % Coverage       61    2    1    1    12   
   
      Peptides:   
        Query    Observed    Mr(expt)    Mr(calc)    Score    Peptide    Result File   
		    348    636.36    1270.71    1270.69    61    QNINIVDISQK    37_1   
   
      Matching Genes:  
               gi|115251678|emb|CAJ69513.1|  (conserved hypothetical protein [Clostridium difficile 630]) 
           
  Protein Group 158   
      Expression Quality:  
         Score      Num Spectra      Num Peptides      High-Qual Peptides      % Coverage       60    1    1    1    4   
   
      Peptides:   
        Query    Observed    Mr(expt)    Mr(calc)    Score    Peptide    Result File   
		    562    868.58    1735.16    1734.89    60    VITGLATSDDDSSITIK    37_2   
   
      Matching Genes:  
               gi|115251108|emb|CAJ68939.1|  (aspartokinase [Clostridium difficile 630]) 
           
  Protein Group 159   
      Expression Quality:  
         Score      Num Spectra      Num Peptides      High-Qual Peptides      % Coverage       59    1    1    1    5   
   
      Peptides:   
        Query    Observed    Mr(expt)    Mr(calc)    Score    Peptide    Result File   
		    256    712.92    1423.82    1423.68    59    MVNLNDAYEIAR +Oxidation (M)    37A_4   
   
      Matching Genes:  
               gi|115252284|emb|CAJ70125.1|  (2,3,4,5-tetrahydropyridine-2,6-dicarboxylate N-succinyltransferase [Clostridium difficile 630]) 
           
  Protein Group 160   
      Expression Quality:  
         Score      Num Spectra      Num Peptides      High-Qual Peptides      % Coverage       58    1    1    1    6   
   
      Peptides:   
        Query    Observed    Mr(expt)    Mr(calc)    Score    Peptide    Result File   
		    309    815.52    1629.03    1628.88    58    FGDGGVDILPIANLTK    37A_4   
   
      Matching Genes:  
               gi|115249811|emb|CAJ67628.1|  (NH3-dependent NAD(+) synthetase [Clostridium difficile 630]) 
           
  Protein Group 161   
      Expression Quality:  
         Score      Num Spectra      Num Peptides      High-Qual Peptides      % Coverage       58    1    1    1    10   
   
      Peptides:   
        Query    Observed    Mr(expt)    Mr(calc)    Score    Peptide    Result File   
		    412    693.95    1385.88    1385.70    58    ELVPNTTDAAVEK    37_3   
   
      Matching Genes:  
               gi|115249844|emb|CAJ67661.1|  (rubredoxin oxidoreductase (desulfoferrodoxin) [Clostridium difficile 630]) 
           
  Protein Group 162   
      Expression Quality:  
         Score      Num Spectra      Num Peptides      High-Qual Peptides      % Coverage       58    1    1    1    7   
   
      Peptides:   
        Query    Observed    Mr(expt)    Mr(calc)    Score    Peptide    Result File   
		    363    652.35    1302.68    1302.59    58    SAEFCANYITK    37_2   
   
      Matching Genes:  
               gi|115252727|emb|CAJ70571.1|  (single-strand binding protein [Clostridium difficile 630]) 
           
  Protein Group 163   
      Expression Quality:  
         Score      Num Spectra      Num Peptides      High-Qual Peptides      % Coverage       57    1    1    1    6   
   
      Peptides:   
        Query    Observed    Mr(expt)    Mr(calc)    Score    Peptide    Result File   
		    384    1041.20    2080.38    2080.03    57    TLEKPGTNVSGTSDFVSVDK    37_5   
   
      Matching Genes:  
               gi|115249887|emb|CAJ67706.1|  (ABC transporter, substrate-binding lipoprotein [Clostridium difficile 630]) 
           
  Protein Group 164   
      Expression Quality:  
         Score      Num Spectra      Num Peptides      High-Qual Peptides      % Coverage       57    1    1    1    9   
   
      Peptides:   
        Query    Observed    Mr(expt)    Mr(calc)    Score    Peptide    Result File   
		    347    801.55    1601.08    1600.82    57    LMVVNDAVANNSVQK    37A_2   
   
      Matching Genes:  
               gi|115252122|emb|CAJ69960.1|  (PTS system, IIb component [Clostridium difficile 630]) 
           
  Protein Group 165   
      Expression Quality:  
         Score      Num Spectra      Num Peptides      High-Qual Peptides      % Coverage       56    1    1    1    7   
   
      Peptides:   
        Query    Observed    Mr(expt)    Mr(calc)    Score    Peptide    Result File   
		    527    862.88    2585.61    2585.31    56    NLIQPEDFSIEEIDEILELAQK    37A_4   
   
      Matching Genes:  
               gi|115249194|emb|CAJ67006.1|  (aspartate carbamoyltransferase catalytic chain [Clostridium difficile 630]) 
           
  Protein Group 166   
      Expression Quality:  
         Score      Num Spectra      Num Peptides      High-Qual Peptides      % Coverage       56    1    1    1    3   
   
      Peptides:   
        Query    Observed    Mr(expt)    Mr(calc)    Score    Peptide    Result File   
		    559    1195.37    2388.72    2388.17    56    LAPSLTLGCGSWGGNSVSENVGVK    37_7   
   
      Matching Genes:  
               gi|115249343|emb|CAJ67156.1|  (aldehyde-alcohol dehydrogenase [includes: alcohol dehydrogenase; acetaldehyde dehydrogenase [acetylating]; pyruvate-formate-lyase deactivase [Clostridium difficile 630]) 
           
  Protein Group 167   
      Expression Quality:  
         Score      Num Spectra      Num Peptides      High-Qual Peptides      % Coverage       56    1    1    1    5   
   
      Peptides:   
        Query    Observed    Mr(expt)    Mr(calc)    Score    Peptide    Result File   
		    404    952.58    1903.15    1902.89    56    SLGPEPWQVCYVEPSR    37A_4   
   
      Matching Genes:  
               gi|115251486|emb|CAJ69319.1|  (glycyl-tRNA synthetase alpha chain [Clostridium difficile 630]) 
           
  Protein Group 168   
      Expression Quality:  
         Score      Num Spectra      Num Peptides      High-Qual Peptides      % Coverage       55    1    1    1    4   
   
      Peptides:   
        Query    Observed    Mr(expt)    Mr(calc)    Score    Peptide    Result File   
		    306    621.88    1241.75    1241.64    55    VDLPLWQESR    37_3   
   
      Matching Genes:  
               gi|115250457|emb|CAJ68280.1|  (putative ATP-binding protein [Clostridium difficile 630]) 
           
  Protein Group 169   
      Expression Quality:  
         Score      Num Spectra      Num Peptides      High-Qual Peptides      % Coverage       54    1    1    1    7   
   
      Peptides:   
        Query    Observed    Mr(expt)    Mr(calc)    Score    Peptide    Result File   
		    385    870.05    1738.08    1737.95    54    IIIENLTNLENVPEK    37A_3   
   
      Matching Genes:  
               gi|115251016|emb|CAJ68845.1|  (putative cyclase [Clostridium difficile 630]) 
           
  Protein Group 170   
      Expression Quality:  
         Score      Num Spectra      Num Peptides      High-Qual Peptides      % Coverage       54    2    1    1    4   
   
      Peptides:   
        Query    Observed    Mr(expt)    Mr(calc)    Score    Peptide    Result File   
		    353    504.32    1509.93    1509.83    54    ISSKPIIATHSNSR    37_4   
   
      Matching Genes:  
               gi|115252633|emb|CAJ70476.1|  (probable dipeptidase [Clostridium difficile 630]) 
           
  Protein Group 171   
      Expression Quality:  
         Score      Num Spectra      Num Peptides      High-Qual Peptides      % Coverage       53    2    1    1    6   
   
      Peptides:   
        Query    Observed    Mr(expt)    Mr(calc)    Score    Peptide    Result File   
		    161    455.30    908.59    908.57    53    TPPAAVLIK    37_2   
   
      Matching Genes:  
               gi|115249065|emb|CAJ66876.1|  (50S ribosomal protein L11 [Clostridium difficile 630]) 
           
  Protein Group 172   
      Expression Quality:  
         Score      Num Spectra      Num Peptides      High-Qual Peptides      % Coverage       53    1    1    1    2   
   
      Peptides:   
        Query    Observed    Mr(expt)    Mr(calc)    Score    Peptide    Result File   
		    123    533.38    1064.74    1064.55    53    IGGAEQIYSK    37_6   
   
      Matching Genes:  
               gi|115251373|emb|CAJ69205.1|  (putative phosphoglucomutase [Clostridium difficile 630]) 
           
  Protein Group 173   
      Expression Quality:  
         Score      Num Spectra      Num Peptides      High-Qual Peptides      % Coverage       53    2    1    1    5   
   
      Peptides:   
        Query    Observed    Mr(expt)    Mr(calc)    Score    Peptide    Result File   
		    215    638.38    1274.75    1274.60    53    NQDVSDEDILK    37A_3   
   
      Matching Genes:  
               gi|115251628|emb|CAJ69461.1|  (putative nitroreductase [Clostridium difficile 630]) 
           
  Protein Group 174   
      Expression Quality:  
         Score      Num Spectra      Num Peptides      High-Qual Peptides      % Coverage       52    1    1    1    6   
   
      Peptides:   
        Query    Observed    Mr(expt)    Mr(calc)    Score    Peptide    Result File   
		    242    551.30    1100.58    1100.61    52    EDIQVIGISK    37_2   
   
      Matching Genes:  
               gi|115250867|emb|CAJ68692.1|  (putative thiol peroxidase (bacterioferritin comigratory protein) [Clostridium difficile 630]) 
           
  Protein Group 175   
      Expression Quality:  
         Score      Num Spectra      Num Peptides      High-Qual Peptides      % Coverage       51    1    1    1    9   
   
      Peptides:   
        Query    Observed    Mr(expt)    Mr(calc)    Score    Peptide    Result File   
		    281    580.29    1158.58    1158.62    51    KAGQICDLVR    37_1   
   
      Matching Genes:  
               gi|115249082|emb|CAJ66893.1|  (50S ribosomal protein L22 [Clostridium difficile 630]) 
           
  Protein Group 176   
      Expression Quality:  
         Score      Num Spectra      Num Peptides      High-Qual Peptides      % Coverage       51    1    1    1    16   
   
      Peptides:   
        Query    Observed    Mr(expt)    Mr(calc)    Score    Peptide    Result File   
		    408    900.22    1798.43    1797.88    51    FIEEIGYYNPISEPK    37A_1   
   
      Matching Genes:  
               gi|115250287|emb|CAJ68109.1|  (30S ribosomal protein S16 [Clostridium difficile 630]) 
           
  Protein Group 177   
      Expression Quality:  
         Score      Num Spectra      Num Peptides      High-Qual Peptides      % Coverage       50    1    1    1    6   
   
      Peptides:   
        Query    Observed    Mr(expt)    Mr(calc)    Score    Peptide    Result File   
		    149    574.88    1147.75    1147.61    50    GLTSVATDSLGK    37A_3   
   
      Matching Genes:  
               gi|115250535|emb|CAJ68359.1|  (putative transcriptional regulator [Clostridium difficile 630]) 
           
  Protein Group 178   
      Expression Quality:  
         Score      Num Spectra      Num Peptides      High-Qual Peptides      % Coverage       50    1    1    1    5   
   
      Peptides:   
        Query    Observed    Mr(expt)    Mr(calc)    Score    Peptide    Result File   
		    351    436.97    1307.89    1307.69    50    KENIAIQEAHR    37_3   
   
      Matching Genes:  
               gi|115251194|emb|CAJ69025.1|  (30S ribosomal protein S2 [Clostridium difficile 630]) 
           
  Protein Group 179   
      Expression Quality:  
         Score      Num Spectra      Num Peptides      High-Qual Peptides      % Coverage       48    1    1    1    11   
   
      Peptides:   
        Query    Observed    Mr(expt)    Mr(calc)    Score    Peptide    Result File   
		    449    700.70    2099.09    2098.09    48    GVTGFVGPGSKPVPLSEDEVK    37A_3   
   
      Matching Genes:  
               gi|115249064|emb|CAJ66875.1|  (transcription antitermination protein [Clostridium difficile 630]) 
           
  Protein Group 180   
      Expression Quality:  
         Score      Num Spectra      Num Peptides      High-Qual Peptides      % Coverage       48    2    1    1    3   
   
      Peptides:   
        Query    Observed    Mr(expt)    Mr(calc)    Score    Peptide    Result File   
		    162    438.67    1312.98    1312.62    48    IGHMGENANLNK +Oxidation (M)    37_5   
   
      Matching Genes:  
               gi|115251586|emb|CAJ69419.1|  (putative aminotransferase [Clostridium difficile 630]) 
           
  Protein Group 181   
      Expression Quality:  
         Score      Num Spectra      Num Peptides      High-Qual Peptides      % Coverage       47    3    1    1    7   
   
      Peptides:   
        Query    Observed    Mr(expt)    Mr(calc)    Score    Peptide    Result File   
		    143    493.80    985.58    985.52    47    IQELAEGAR    37A_1   
   
      Matching Genes:  
               gi|115249093|emb|CAJ66904.1|  (50S ribosomal protein L18 [Clostridium difficile 630]) 
           
  Protein Group 182   
      Expression Quality:  
         Score      Num Spectra      Num Peptides      High-Qual Peptides      % Coverage       47    1    1    1    4   
   
      Peptides:   
        Query    Observed    Mr(expt)    Mr(calc)    Score    Peptide    Result File   
		    79    520.39    1038.77    1038.64    47    VTALINAIPK    37A_7   
   
      Matching Genes:  
               gi|115252333|emb|CAJ70174.1|  (PTS system, IIc component [Clostridium difficile 630]) 
           
  Protein Group 183   
      Expression Quality:  
         Score      Num Spectra      Num Peptides      High-Qual Peptides      % Coverage       46    1    1    1    5   
   
      Peptides:   
        Query    Observed    Mr(expt)    Mr(calc)    Score    Peptide    Result File   
		    280    756.44    1510.87    1510.81    46    VVEPDILIEEVEK    37A_4   
   
      Matching Genes:  
               gi|115250078|emb|CAJ67898.1|  (3-hydroxybutyryl-CoA dehydratase [Clostridium difficile 630]) 
           
  Protein Group 184   
      Expression Quality:  
         Score      Num Spectra      Num Peptides      High-Qual Peptides      % Coverage       46    1    1    1    4   
   
      Peptides:   
        Query    Observed    Mr(expt)    Mr(calc)    Score    Peptide    Result File   
		    165    600.33    1198.65    1199.55    46    FLGEEYNNSK    37A_3   
   
      Matching Genes:  
               gi|115252486|emb|CAJ70329.1|  (precorrin-4 C(11)-methyltransferase [Clostridium difficile 630]) 
           
  Protein Group 185   
      Expression Quality:  
         Score      Num Spectra      Num Peptides      High-Qual Peptides      % Coverage       46    1    1    1    4   
   
      Peptides:   
        Query    Observed    Mr(expt)    Mr(calc)    Score    Peptide    Result File   
		    187    617.33    1232.65    1232.63    46    GIGYAFQQPPR    37A_3   
   
      Matching Genes:  
               gi|115252673|emb|CAJ70516.1|  (ABC transporter, ATP-binding protein [Clostridium difficile 630]) 
           
  Protein Group 186   
      Expression Quality:  
         Score      Num Spectra      Num Peptides      High-Qual Peptides      % Coverage       45    4    1    1    1   
   
      Peptides:   
        Query    Observed    Mr(expt)    Mr(calc)    Score    Peptide    Result File   
		    177    450.33    1347.98    1347.77    45    GRPVTGPGNRPLK    37A_8   
   
      Matching Genes:  
               gi|115249071|emb|CAJ66882.1|  (DNA-directed RNA polymerase beta' chain [Clostridium difficile 630]) 
           
  Protein Group 187   
      Expression Quality:  
         Score      Num Spectra      Num Peptides      High-Qual Peptides      % Coverage       45    1    1    1    2   
   
      Peptides:   
        Query    Observed    Mr(expt)    Mr(calc)    Score    Peptide    Result File   
		    154    652.36    1302.71    1302.60    45    SATHDQNFLDR    37A_6   
   
      Matching Genes:  
               gi|115251601|emb|CAJ69434.1|  (conserved hypothetical protein [Clostridium difficile 630]) 
           
  Protein Group 188   
      Expression Quality:  
         Score      Num Spectra      Num Peptides      High-Qual Peptides      % Coverage       44    1    1    1    8   
   
      Peptides:   
        Query    Observed    Mr(expt)    Mr(calc)    Score    Peptide    Result File   
		    268    670.87    1339.72    1339.63    44    VMFELAGVSEDK +Oxidation (M)    37A_2   
   
      Matching Genes:  
               gi|115249084|emb|CAJ66895.1|  (50S ribosomal protein L16 [Clostridium difficile 630]) 
           
  Protein Group 189   
      Expression Quality:  
         Score      Num Spectra      Num Peptides      High-Qual Peptides      % Coverage       43    1    1    1    9   
   
      Peptides:   
        Query    Observed    Mr(expt)    Mr(calc)    Score    Peptide    Result File   
		    462    745.84    2234.50    2234.16    43    NIEGVQTALVNTMNVYDILK    37A_3   
   
      Matching Genes:  
               gi|115249078|emb|CAJ66889.1|  (50S ribosomal protein L4 [Clostridium difficile 630]) 
           
  Protein Group 190   
      Expression Quality:  
         Score      Num Spectra      Num Peptides      High-Qual Peptides      % Coverage       43    1    1    1    3   
   
      Peptides:   
        Query    Observed    Mr(expt)    Mr(calc)    Score    Peptide    Result File   
		    377    872.82    1743.62    1742.87    43    IFPTGVEDVPNEEGLK    37_6   
   
      Matching Genes:  
               gi|115252150|emb|CAJ69988.1|  (6-phospho-beta-glucosidase [Clostridium difficile 630]) 
           
  Protein Group 191   
      Expression Quality:  
         Score      Num Spectra      Num Peptides      High-Qual Peptides      % Coverage       42    2    1    1    7   
   
      Peptides:   
        Query    Observed    Mr(expt)    Mr(calc)    Score    Peptide    Result File   
		    176    556.91    1111.80    1111.62    42    ILVPIDGTER    37A_1   
   
      Matching Genes:  
               gi|115249829|emb|CAJ67646.1|  (putative universal stress protein [Clostridium difficile 630]) 
           
  Protein Group 192   
      Expression Quality:  
         Score      Num Spectra      Num Peptides      High-Qual Peptides      % Coverage       42    3    1    1    8   
   
      Peptides:   
        Query    Observed    Mr(expt)    Mr(calc)    Score    Peptide    Result File   
		    438    682.07    2043.19    2042.21    42    AIEEAGIPTIIIAALPPVVR    37A_3   
   
      Matching Genes:  
               gi|115252298|emb|CAJ70139.1|  (proline reductase [Clostridium difficile 630]) 
           
  Protein Group 193   
      Expression Quality:  
         Score      Num Spectra      Num Peptides      High-Qual Peptides      % Coverage       41    1    1    1    2   
   
      Peptides:   
        Query    Observed    Mr(expt)    Mr(calc)    Score    Peptide    Result File   
		    66    494.80    987.58    987.46    41    QEDLEAGAR    37A_6   
   
      Matching Genes:  
               gi|115249055|emb|CAJ66866.1|  (cysteinyl-tRNA synthetase [Clostridium difficile 630]) 
           
  Protein Group 194   
      Expression Quality:  
         Score      Num Spectra      Num Peptides      High-Qual Peptides      % Coverage       41    1    1    1    1   
   
      Peptides:   
        Query    Observed    Mr(expt)    Mr(calc)    Score    Peptide    Result File   
		    98    516.32    1030.63    1030.59    41    LVTEVLTEK    37_8   
   
      Matching Genes:  
               gi|115249184|emb|CAJ66996.1|  (putative oxidoreductase, acetyl-CoA synthase subunit [Clostridium difficile 630]) 
           
  Protein Group 195   
      Expression Quality:  
         Score      Num Spectra      Num Peptides      High-Qual Peptides      % Coverage       41    1    1    1    7   
   
      Peptides:   
        Query    Observed    Mr(expt)    Mr(calc)    Score    Peptide    Result File   
		    221    625.41    1248.81    1248.67    41    LAVENNVSYLK    37A_2   
   
      Matching Genes:  
               gi|115249853|emb|CAJ67670.1|  (putative acetyltransferase [Clostridium difficile 630]) 
           
  Protein Group 196   
      Expression Quality:  
         Score      Num Spectra      Num Peptides      High-Qual Peptides      % Coverage       41    4    1    1    1   
   
      Peptides:   
        Query    Observed    Mr(expt)    Mr(calc)    Score    Peptide    Result File   
		    147    561.36    1120.70    1120.58    41    KMIEENTIK +Oxidation (M)    37_7   
   
      Matching Genes:  
               gi|115250692|emb|CAJ68516.1|  (putative signaling protein [Clostridium difficile 630]) 
           
  Protein Group 197   
      Expression Quality:  
         Score      Num Spectra      Num Peptides      High-Qual Peptides      % Coverage       40    1    1    1    2   
   
      Peptides:   
        Query    Observed    Mr(expt)    Mr(calc)    Score    Peptide    Result File   
		    55    425.30    848.58    848.43    40    DMIEVVK +Oxidation (M)    37_3   
   
      Matching Genes:  
               gi|115249743|emb|CAJ67560.1|  (putative carbon monoxide dehydrogenase/acetyl-CoA synthase complex, alpha subunit [Clostridium difficile 630]) 
           
  Protein Group 198   
      Expression Quality:  
         Score      Num Spectra      Num Peptides      High-Qual Peptides      % Coverage       40    1    1    1    8   
   
      Peptides:   
        Query    Observed    Mr(expt)    Mr(calc)    Score    Peptide    Result File   
		    522    845.33    2532.96    2532.12    40    GISFMENQAGWHGTAPSDEELEK    37A_4   
   
      Matching Genes:  
               gi|115252520|emb|CAJ70363.1|  (transketolase, thiamine disphosphate-binding subunit [Clostridium difficile 630]) 
           
  Protein Group 199   
      Expression Quality:  
         Score      Num Spectra      Num Peptides      High-Qual Peptides      % Coverage       40    1    1    1    2   
   
      Peptides:   
        Query    Observed    Mr(expt)    Mr(calc)    Score    Peptide    Result File   
		    201    709.93    1417.84    1417.63    40    LIDNNSNKEEEN    37A_6   
   
      Matching Genes:  
               gi|115252615|emb|CAJ70458.1|  (lysyl-tRNA synthetase [Clostridium difficile 630]) 
           
  Protein Group 200   
      Expression Quality:  
         Score      Num Spectra      Num Peptides      High-Qual Peptides      % Coverage       39    2    1    0    6   
   
      Peptides:   
        Query    Observed    Mr(expt)    Mr(calc)    Score    Peptide    Result File   
		    20    410.25    818.48    818.45    39    IETTVTR    37A_1   
   
      Matching Genes:  
               gi|115249107|emb|CAJ66918.1|  (50S ribosomal protein L17 [Clostridium difficile 630]) 
           
  Protein Group 201   
      Expression Quality:  
         Score      Num Spectra      Num Peptides      High-Qual Peptides      % Coverage       39    1    1    0    5   
   
      Peptides:   
        Query    Observed    Mr(expt)    Mr(calc)    Score    Peptide    Result File   
		    166    600.88    1199.75    1199.55    39    YNLQEDFSGK    37A_3   
   
      Matching Genes:  
               gi|115250846|emb|CAJ68670.1|  (tellurium resistance protein [Clostridium difficile 630]) 
           
  Protein Group 202   
      Expression Quality:  
         Score      Num Spectra      Num Peptides      High-Qual Peptides      % Coverage       39    1    1    0    4   
   
      Peptides:   
        Query    Observed    Mr(expt)    Mr(calc)    Score    Peptide    Result File   
		    187    499.31    996.61    997.56    39    IPVTQDGLR    37_3   
   
      Matching Genes:  
               gi|115251164|emb|CAJ68995.1|  (fructose-6-phosphate aldolase 2 [Clostridium difficile 630]) 
             
