# Supplementary material for: Semiquantitative Analysis of Clinical Heat Stress in Clostridium difficile Strain 630 Using a GeLC/MS Workflow with emPAI Quantitation
Source: PLoS One. 2014 Feb 24;9(2):e88960. doi: 10.1371/journal.pone.0088960 (PMC3933415; doi:10.1371/journal.pone.0088960)
Supplement: File S3 — PROVALT output html file from 37°C biological replicate, single lane GeLC/MS, 3 injections, 163 proteins. (HTML) [file pone.0088960.s003.html]

   Complete output   Complete output    
 
   
  Protein Group 1   
      Expression Quality:  
         Score      Num Spectra      Num Peptides      High-Qual Peptides      % Coverage       1305    74    29    16    30   
   
      Peptides:   
        Query    Observed    Mr(expt)    Mr(calc)    Score    Peptide    Result File   
		    484    1051.45    2100.88    2100.04    56    ALASQALSIFGDHQDVMAAR    biorepCdiff_7   
		    264    595.14    1782.40    1781.83    27    ATYLIDEADYIACHK    biorepCdiffA_7   
		    36    481.35    960.69    960.54    28    DFILAQVR    biorepCdiffA_8   
		    43    528.85    1055.68    1056.54    32    ENAPMIIGGR    biorepCdiffA_8   
		    252    767.07    1532.12    1531.75    44    EPGSTGEPLYLDVR    biorepCdiff_8   
		    65    471.38    940.74    940.50    31    FYTVNAVK    biorepCdiff_7   
		    187    756.04    1510.06    1509.72    42    GTAQNPDIYFQTR    biorepCdiffA_7   
		    202    702.00    1401.99    1401.67    42    HFLDAMPSTVER    biorepCdiff_8   
		    160    505.91    1514.72    1514.67    46    HSLFDYYGAEDAK    biorepCdiffA_8   
		    431    1168.81    2335.61    2335.01    52    IQVSPLDCTGCGNCADICPAK    biorepCdiffA_7   
		    347    938.77    1875.53    1874.99    97    IVNMNYAAVDAGINALVK    biorepCdiff_8   
		    277    822.56    1643.11    1642.76    87    KHSLFDYYGAEDAK    biorepCdiff_8   
		    57    528.97    1055.93    1055.57    32    LGQEIGLGNR    biorepCdiffA_7   
		    197    466.03    1395.08    1394.71    39    LPFIHFFDGFR    biorepCdiff_8   
		    27    461.27    920.52    920.46    41    NPFTLDSK    biorepCdiffA_8   
		    202    676.51    1351.01    1350.65    34    QFPEEADGLFAK    biorepCdiff_7   
		    440    1095.83    2189.64    2188.96    29    QPLMEFSGACAGCGETAYIK    biorepCdiff_8   
		    257    881.68    1761.34    1760.89    50    TKEPGSTGEPLYLDVR    biorepCdiffA_7   
		    416    1055.32    2108.63    2107.99    63    TVANEAQAVACGYWHLYR    biorepCdiff_8   
		    138    689.16    1376.30    1375.70    35    TVFDNLVSEQPK    biorepCdiffA_7   
		    261    518.27    1551.79    1550.86    38    VAGELLPGVFHVSAR    biorepCdiff_8   
		    112    537.46    1072.90    1072.56    28    VDVMPANTVK    biorepCdiff_7   
		    89    545.22    1088.43    1088.55    26    VDVMPANTVK +Oxidation (M)    biorepCdiff_8   
		    482    857.68    2570.01    2569.27    66    VELLENEDYASLLNFEAVQAFR    biorepCdiffA_1   
		    179    815.43    1628.85    1628.77    59    VEVPASWENAVDADK    biorepCdiffA_8   
		    354    953.68    1905.35    1904.89    28    VVELLEKPACDCTDEK    biorepCdiff_8   
		    107    525.97    1049.93    1049.55    42    VVTQLYGDR    biorepCdiff_7   
		    135    685.98    1369.94    1369.58    64    YAQAYFDYDSK    biorepCdiffA_7   
		    148    663.50    1324.99    1324.70    47    YYQNIVGIVEK    biorepCdiff_8   
   
      Matching Genes:  
               gi|115251733|emb|CAJ69568.1|  (pyruvate-flavodoxin oxidoreductase [Clostridium difficile 630]) 
           
  Protein Group 2   
      Expression Quality:  
         Score      Num Spectra      Num Peptides      High-Qual Peptides      % Coverage       796    36    14    11    49   
   
      Peptides:   
        Query    Observed    Mr(expt)    Mr(calc)    Score    Peptide    Result File   
		    92    498.32    994.62    994.52    67    AEAHIQAGAK    biorepCdiff_5   
		    97    505.98    1009.94    1009.65    44    AIGLVIPSLK    biorepCdiff_7   
		    108    619.26    1236.51    1237.64    71    DKAEAHIQAGAK    biorepCdiffA_5   
		    294    718.08    1434.15    1433.74    53    FEVVAINDLTDAK    biorepCdiff_5   
		    45    468.51    935.00    934.48    38    FNGEIEVK    biorepCdiffA_5   
		    631    1212.98    2423.95    2423.15    33    GLMTTIHAYTNDQNTLDGPHPK    biorepCdiff_5   
		    177    649.99    1297.97    1297.76    65    KVVISAPATGDLK    biorepCdiffA_3   
		    512    775.95    2324.84    2324.10    28    MMEQQDKFEVVAINDLTDAK    biorepCdiffA_5   
		    508    927.52    2779.55    2778.36    75    NPADLPWAELGVDIVLECTGFFTSK    biorepCdiffA_1   
		    348    660.06    1318.10    1317.66    54    NVTVEEINAAMK    biorepCdiff_2   
		    110    556.50    1110.98    1110.61    51    TLGYFAQLAK    biorepCdiffA_1   
		    579    887.35    1772.68    1771.98    98    VPVVTGSITELVCTLGK    biorepCdiff_2   
		    230    586.08    1170.14    1169.67    54    VVISAPATGDLK    biorepCdiff_3   
		    635    1046.29    2090.56    2089.98    65    VVSWYDNEMSYTSQLIR    biorepCdiff_2   
   
      Matching Genes:  
               gi|115252231|emb|CAJ70071.1|  (glyceraldehyde-3-phosphate dehydrogenase 2 [Clostridium difficile 630]) 
           
  Protein Group 3   
      Expression Quality:  
         Score      Num Spectra      Num Peptides      High-Qual Peptides      % Coverage       771    41    18    11    26   
   
      Peptides:   
        Query    Observed    Mr(expt)    Mr(calc)    Score    Peptide    Result File   
		    130    681.60    1361.19    1360.69    34    AVTDLLDEMNIK    biorepCdiffA_7   
		    106    641.46    1280.92    1280.55    40    DMYLNSYYGR    biorepCdiffA_7   
		    45    497.84    993.67    994.47    34    ENMLWFR    biorepCdiffA_7   
		    137    688.54    1375.06    1374.78    48    FQSLLVAIEDLK    biorepCdiffA_7   
		    255    1011.07    2020.13    2019.92    56    HTTFFEVEPDPTLECAK    biorepCdiffA_8   
		    171    714.98    1427.94    1427.69    25    IFATYSQEQVDK    biorepCdiffA_7   
		    119    560.02    1118.02    1117.65    58    IFLAASLAANK    biorepCdiff_7   
		    259    1019.21    2036.41    2036.06    28    ILINTPSSQGGIGDLYNFK    biorepCdiffA_8   
		    84    588.52    1175.02    1174.62    36    LAGFEVPVDTK    biorepCdiffA_7   
		    99    511.54    1021.07    1020.57    30    LSPVLAMYK    biorepCdiff_7   
		    444    774.90    2321.68    2321.09    44    LVEDGGFGHTSSLYIDDVNQR    biorepCdiffA_1   
		    276    818.59    1635.17    1634.74    50    NHYASEYIYNAYK    biorepCdiff_8   
		    158    621.64    1241.27    1240.71    49    NIILVNGGLNSK    biorepCdiff_7   
		    102    516.51    1031.01    1030.60    48    TAVNSILVSK    biorepCdiff_7   
		    235    562.91    1685.72    1684.78    27    TCGVIEKDEAFGMTK    biorepCdiffA_7   
		    503    1064.84    2127.67    2127.07    58    VLIGEVESVEIEEAFAHEK    biorepCdiff_7   
		    39    485.95    969.88    969.57    47    VPLAIMAQK    biorepCdiffA_7   
		    125    570.46    1138.90    1138.64    59    YAGIASFLGLK    biorepCdiff_7   
   
      Matching Genes:  
               gi|115252023|emb|CAJ69859.1|  (aldehyde-alcohol dehydrogenase [includes: alcohol dehydrogenase and pyruvate-formate-lyase deactivase [Clostridium difficile 630]) 
           
  Protein Group 4   
      Expression Quality:  
         Score      Num Spectra      Num Peptides      High-Qual Peptides      % Coverage       674    33    12    10    31   
   
      Peptides:   
        Query    Observed    Mr(expt)    Mr(calc)    Score    Peptide    Result File   
		    59    530.02    1058.02    1057.60    50    AVTVAVEELK    biorepCdiffA_6   
		    94    496.36    990.71    990.48    67    EADLSMLGR    biorepCdiff_6   
		    489    883.93    2648.76    2648.36    57    EMLQDIAILTGAQVISEELGYDLK    biorepCdiffA_1   
		    269    767.05    1532.09    1531.82    73    FGSPLITNDGVTIAK    biorepCdiffA_2   
		    77    468.40    934.78    934.51    57    GTFDVVAVK    biorepCdiff_6   
		    380    717.30    2148.87    2148.22    57    ISNIQELLPVLEQIVQQGK    biorepCdiffA_6   
		    588    760.02    2277.03    2276.31    33    KISNIQELLPVLEQIVQQGK    biorepCdiff_6   
		    139    587.52    1173.02    1172.65    52    LIAEAMEIVGK    biorepCdiff_6   
		    163    619.96    1237.91    1237.71    33    NVTAGANPILLR    biorepCdiff_6   
		    520    1105.38    3313.11    3312.68    51    QIAINAGLEGAVIVQNVVNSEAETGFDALNEK    biorepCdiffA_2   
		    360    1044.88    2087.75    2087.09    85    TNDVAGDGTTTATVLAQAIIR    biorepCdiffA_6   
		    53    508.88    1015.74    1015.55    59    VGAATEVELK    biorepCdiffA_6   
   
      Matching Genes:  
               gi|115249204|emb|CAJ67016.1|  (60 kDa chaperonin [Clostridium difficile 630]) 
           
  Protein Group 5   
      Expression Quality:  
         Score      Num Spectra      Num Peptides      High-Qual Peptides      % Coverage       538    23    12    6    49   
   
      Peptides:   
        Query    Observed    Mr(expt)    Mr(calc)    Score    Peptide    Result File   
		    637    847.80    2540.39    2539.30    78    AYEGGFAIGAFNISDLEQLQGVLK    biorepCdiff_4   
		    53    530.36    1058.71    1058.56    44    DAIQAVVESK    biorepCdiffA_4   
		    89    611.51    1221.01    1220.63    40    FDILEEIQSK    biorepCdiffA_4   
		    87    609.58    1217.14    1216.59    31    IDDVLGSANSIN    biorepCdiffA_4   
		    130    489.51    977.00    976.46    52    INMDTDLR    biorepCdiff_4   
		    29    423.97    845.93    845.48    32    LAMTAAIR    biorepCdiff_4   
		    371    781.14    1560.27    1559.73    38    NSYVMIQASMSAVK +2 Oxidation (M)    biorepCdiff_4   
		    654    911.80    2732.39    2732.18    30    TCIDAGFSSVMIDGSHFDFEENVR    biorepCdiff_4   
		    275    873.23    1744.44    1743.92    54    TGVDSLAIAIGTSHGAFK    biorepCdiffA_4   
		    162    672.98    1343.94    1343.69    39    YAGPHTLVEMVK    biorepCdiffA_4   
		    171    680.96    1359.90    1359.69    38    YAGPHTLVEMVK +Oxidation (M)    biorepCdiffA_4   
		    481    770.11    1538.20    1537.74    62    YTQPAEAVEFVER    biorepCdiff_3   
   
      Matching Genes:  
               gi|115249409|emb|CAJ67224.1|  (putative fructose-bisphosphate aldolase [Clostridium difficile 630]) 
           
  Protein Group 6   
      Expression Quality:  
         Score      Num Spectra      Num Peptides      High-Qual Peptides      % Coverage       528    14    10    8    24   
   
      Peptides:   
        Query    Observed    Mr(expt)    Mr(calc)    Score    Peptide    Result File   
		    387    1086.79    2171.57    2171.01    38    ESDDVFYTWAGPEVAVASTK    biorepCdiffA_6   
		    417    611.49    1831.46    1830.90    49    FESQTDTEVIAHLVDK    biorepCdiff_6   
		    372    861.28    1720.55    1719.92    32    FVNIPVITDIASEFR    biorepCdiff_6   
		    150    679.52    1357.03    1356.71    49    GAYVVAIAQSHNK    biorepCdiffA_6   
		    105    614.52    1227.03    1226.69    45    GTPVIAIATQEK    biorepCdiffA_6   
		    295    749.53    1497.04    1496.67    91    GYDSAGVAVNSSNEK    biorepCdiff_6   
		    298    754.11    1506.20    1505.76    48    IQEILDNEEYIK    biorepCdiff_6   
		    112    622.47    1242.93    1242.72    81    KAAEVIVEGLSK    biorepCdiffA_6   
		    279    733.56    1465.10    1464.74    48    TVVSSEHAFYLGR    biorepCdiff_6   
		    580    754.05    2259.14    2258.03    47    WATHGEPSDVNSHPHFNQAK    biorepCdiff_6   
   
      Matching Genes:  
               gi|115249129|emb|CAJ66940.1|  (glucosamine--fructose-6-phosphate aminotransferase [isomerizing] [Clostridium difficile 630]) 
           
  Protein Group 7   
      Expression Quality:  
         Score      Num Spectra      Num Peptides      High-Qual Peptides      % Coverage       518    21    13    7    38   
   
      Peptides:   
        Query    Observed    Mr(expt)    Mr(calc)    Score    Peptide    Result File   
		    376    864.89    1727.76    1727.89    44    ALCEELLAMPVVAGQK    biorepCdiff_6   
		    387    880.10    1758.18    1757.88    46    ALQSGTSHFLGQHFTK    biorepCdiff_6   
		    139    669.02    1336.03    1335.62    51    DIENNQAMVFR    biorepCdiffA_6   
		    583    1132.83    2263.64    2263.07    60    EAEHVEGFAPEVAWVTHGGNK    biorepCdiff_6   
		    413    914.16    1826.31    1825.82    42    EDNTSIVENMDEFRK    biorepCdiff_6   
		    426    931.12    1860.22    1859.86    32    EGNLANPYHTSWGASTR    biorepCdiff_6   
		    448    963.18    1924.34    1923.90    53    GVAVEVDDRDNYTTGWK    biorepCdiff_6   
		    120    559.34    1116.67    1116.61    32    IKEETGATIR    biorepCdiff_6   
		    485    1022.34    2042.67    2042.00    26    LCVRPTSETIICTMYAK    biorepCdiff_6   
		    439    944.71    1887.40    1886.84    33    MEDDFPQWYTDVITK    biorepCdiff_6   
		    95    497.35    992.69    992.52    40    QFVEEITK    biorepCdiff_6   
		    66    560.91    1119.80    1119.58    34    TDLVDYAPVK    biorepCdiffA_6   
		    171    709.70    1417.38    1416.91    25    VAPIQVVIVPIAAK    biorepCdiffA_6   
   
      Matching Genes:  
               gi|115249053|emb|CAJ66864.1|  (putative dual-specificity prolyl/cysteinyl-tRNA synthetase [Clostridium difficile 630]) 
           
  Protein Group 8   
      Expression Quality:  
         Score      Num Spectra      Num Peptides      High-Qual Peptides      % Coverage       479    18    7    6    39   
   
      Peptides:   
        Query    Observed    Mr(expt)    Mr(calc)    Score    Peptide    Result File   
		    602    946.20    1890.39    1889.90    85    EAEQAGADFVGAEELVQK    biorepCdiff_3   
		    234    594.95    1187.89    1187.58    27    FDETVEAHIK    biorepCdiff_3   
		    594    936.27    1870.53    1869.94    124    FYDASEALTLVSDIAGAK    biorepCdiff_3   
		    492    790.63    1579.24    1578.83    73    LTENFTALMDAIIK    biorepCdiff_3   
		    88    512.89    1023.76    1023.52    56    SGTVTFDVAK    biorepCdiffA_3   
		    260    617.57    1233.13    1232.64    67    SITVASSMGPGVK    biorepCdiff_3   
		    151    539.48    1076.94    1076.63    47    TNIIHVPVGK    biorepCdiff_4   
   
      Matching Genes:  
               gi|115249066|emb|CAJ66877.1|  (50S ribosomal protein L1 [Clostridium difficile 630]) 
           
  Protein Group 9   
      Expression Quality:  
         Score      Num Spectra      Num Peptides      High-Qual Peptides      % Coverage       454    21    8    7    29   
   
      Peptides:   
        Query    Observed    Mr(expt)    Mr(calc)    Score    Peptide    Result File   
		    423    683.38    2047.12    2046.08    41    AAADEIGLPLFQYLGGVNAK    biorepCdiffA_5   
		    641    1237.05    2472.08    2471.17    28    AGYKPGEDVMLGLDVAATEMYNK    biorepCdiff_6   
		    394    888.20    1774.39    1773.93    81    AIVPSGASTGAFEAVELR    biorepCdiff_6   
		    210    665.03    1328.06    1327.76    65    EALELIVEAITK    biorepCdiff_6   
		    196    657.54    1313.07    1312.73    57    GIENGVANSILVK    biorepCdiff_6   
		    84    560.03    1118.04    1117.56    65    MGAEVFHSLK    biorepCdiffA_5   
		    394    1095.40    2188.79    2188.09    69    SGETEDSTIADLAVAVNAGQIK    biorepCdiffA_6   
		    57    525.50    1048.98    1048.59    48    SVIELVYAR    biorepCdiffA_6   
   
      Matching Genes:  
               gi|115252227|emb|CAJ70067.1|  (enolase [Clostridium difficile 630]) 
           
  Protein Group 10   
      Expression Quality:  
         Score      Num Spectra      Num Peptides      High-Qual Peptides      % Coverage       445    15    11    5    18   
   
      Peptides:   
        Query    Observed    Mr(expt)    Mr(calc)    Score    Peptide    Result File   
		    271    484.38    1450.13    1449.69    37    AGAPFAPGANPMHGR    biorepCdiff_7   
		    334    1033.44    2064.87    2064.92    43    FEPITSEYLDYDEVMSK    biorepCdiffA_7   
		    141    460.73    1379.17    1378.73    53    KLWDEAMVLFK    biorepCdiffA_7   
		    102    626.61    1251.20    1250.64    31    LWDEAMVLFK    biorepCdiffA_7   
		    273    923.66    1845.32    1844.86    30    MAESYGFDISKPATNSK    biorepCdiffA_7   
		    43    491.35    980.69    980.45    31    MNAWQGFK    biorepCdiffA_7   
		    368    1066.36    2130.71    2129.93    34    MVENSCEAFGYELDPEIK    biorepCdiffA_7   
		    132    660.47    1318.93    1318.69    53    SGIITGLPDAYGR    biorepCdiffA_6   
		    67    549.47    1096.92    1096.59    33    VSGYAVNFIK    biorepCdiffA_7   
		    308    986.71    1971.41    1970.89    60    VSIDTSSVQYENDDLMR    biorepCdiffA_7   
		    209    533.48    1597.43    1596.72    40    YSYEALEMALHDR    biorepCdiffA_7   
   
      Matching Genes:  
               gi|115249776|emb|CAJ67593.1|  (formate acetyltransferase [Clostridium difficile 630]) 
           
  Protein Group 11   
      Expression Quality:  
         Score      Num Spectra      Num Peptides      High-Qual Peptides      % Coverage       441    34    7    6    52   
   
      Peptides:   
        Query    Observed    Mr(expt)    Mr(calc)    Score    Peptide    Result File   
		    125    452.50    902.98    902.53    50    AFLGLLNR    biorepCdiff_2   
		    492    746.18    1490.34    1489.80    91    FAELLGEVVVADTK    biorepCdiff_2   
		    751    1320.42    2638.82    2638.14    28    FVCTVCGYIHEGDAAPAQCPVCK    biorepCdiff_3   
		    117    591.43    1180.85    1180.61    65    IALEEAEHAAK    biorepCdiffA_2   
		    309    849.63    1697.25    1696.90    86    IGVAQGVDEEIIEGLR    biorepCdiffA_1   
		    335    577.44    1152.87    1152.55    56    VGADKFEEMK    biorepCdiff_1   
		    196    691.02    1380.03    1379.67    65    VRVDAEYGATDGK    biorepCdiffA_1   
   
      Matching Genes:  
               gi|115250515|emb|CAJ68339.1|  (putative ruberythrin [Clostridium difficile 630]) 
           
  Protein Group 12   
      Expression Quality:  
         Score      Num Spectra      Num Peptides      High-Qual Peptides      % Coverage       431    35    7    6    52   
   
      Peptides:   
        Query    Observed    Mr(expt)    Mr(calc)    Score    Peptide    Result File   
		    125    452.50    902.98    902.53    50    AFLGLLNR    biorepCdiff_2   
		    492    746.18    1490.34    1489.80    91    FAELLGEVVVADTK    biorepCdiff_2   
		    751    1320.42    2638.82    2638.14    28    FVCTVCGYIHEGDAAPAQCPVCK    biorepCdiff_3   
		    267    608.44    1214.86    1214.59    63    IAFEEAEHAAK    biorepCdiff_2   
		    290    820.64    1639.27    1638.89    78    IGVAQGVDAEIIEGLR    biorepCdiffA_1   
		    335    577.44    1152.87    1152.55    56    VGADKFEEMK    biorepCdiff_1   
		    196    691.02    1380.03    1379.67    65    VRVDAEYGATDGK    biorepCdiffA_1   
   
      Matching Genes:  
               gi|115250565|emb|CAJ68389.1|  (putative rubrerythrin [Clostridium difficile 630]) 
           
  Protein Group 13   
      Expression Quality:  
         Score      Num Spectra      Num Peptides      High-Qual Peptides      % Coverage       429    17    9    6    25   
   
      Peptides:   
        Query    Observed    Mr(expt)    Mr(calc)    Score    Peptide    Result File   
		    527    680.71    2039.10    2038.93    71    AAQEQQAAQGAEQAQDNGPK    biorepCdiff_4   
		    532    1053.66    3157.97    3157.42    28    AAQEQQAAQGAEQAQDNGPKDDNVVDADFK    biorepCdiffA_7   
		    366    1065.35    2128.68    2128.03    45    EKIEAFNQAESTIYQTEK    biorepCdiffA_7   
		    589    803.60    2407.78    2407.19    29    ELSSTMSSNINLPFITATAEGPK    biorepCdiff_7   
		    72    477.91    953.80    953.55    44    IPAVQEAVK    biorepCdiff_7   
		    351    946.26    1890.51    1889.96    77    ITITSNTNLSEAEIEQK    biorepCdiff_8   
		    609    834.30    2499.88    2499.24    57    SQIFSTAADNQTAVDIHVLQGER    biorepCdiff_7   
		    319    796.12    1590.22    1589.87    25    SYTPQEISAIILQK    biorepCdiff_7   
		    436    792.33    2373.96    2373.20    53    TALQDAGLSTGDIDDVLLVGGSTR    biorepCdiffA_7   
   
      Matching Genes:  
               gi|115251515|emb|CAJ69348.1|  (chaperone protein [Clostridium difficile 630]) 
           
  Protein Group 14   
      Expression Quality:  
         Score      Num Spectra      Num Peptides      High-Qual Peptides      % Coverage       427    19    7    5    48   
   
      Peptides:   
        Query    Observed    Mr(expt)    Mr(calc)    Score    Peptide    Result File   
		    420    711.07    1420.12    1419.67    80    EIMDAANNTGASVK    biorepCdiff_3   
		    180    670.06    1338.11    1337.72    74    IVYDAFAIVAEK    biorepCdiffA_2   
		    235    558.91    1115.81    1115.60    57    LINNLMVDGK    biorepCdiff_2   
		    430    696.54    1391.07    1390.69    54    REVLPDPMYGSK    biorepCdiff_2   
		    378    647.48    1939.43    1939.04    35    RVGGANYQVPIEVRPER    biorepCdiffA_2   
		    289    618.92    1235.83    1235.60    94    TGEEALEVFNK    biorepCdiff_2   
		    559    892.74    1783.47    1782.94    33    VGGANYQVPIEVRPER    biorepCdiff_3   
   
      Matching Genes:  
               gi|115249073|emb|CAJ66884.1|  (30S ribosomal protein S7 [Clostridium difficile 630]) 
           
  Protein Group 15   
      Expression Quality:  
         Score      Num Spectra      Num Peptides      High-Qual Peptides      % Coverage       421    12    8    6    33   
   
      Peptides:   
        Query    Observed    Mr(expt)    Mr(calc)    Score    Peptide    Result File   
		    368    794.28    1586.54    1585.88    27    AADPIVVLFGATSIGR    biorepCdiff_5   
		    76    545.45    1088.89    1088.55    48    ATIDAGWLDK    biorepCdiffA_5   
		    599    760.02    2277.04    2276.15    60    ENLDILYELAEIIGGEVSGSR    biorepCdiff_5   
		    489    987.28    1972.55    1971.96    74    IHTGLTADCTGLAVAEDTK    biorepCdiff_5   
		    118    629.63    1257.25    1256.69    69    MGNVLVVIEQR    biorepCdiffA_5   
		    268    699.21    1396.40    1395.83    54    VLPELISQLSVAK    biorepCdiff_5   
		    28    444.36    886.71    886.55    39    VSALLLGSK    biorepCdiffA_5   
		    183    637.10    1272.18    1271.65    50    YADVGIVGDVHK    biorepCdiff_5   
   
      Matching Genes:  
               gi|115250077|emb|CAJ67897.1|  (electron transfer flavoprotein alpha-subunit [Clostridium difficile 630]) 
           
  Protein Group 16   
      Expression Quality:  
         Score      Num Spectra      Num Peptides      High-Qual Peptides      % Coverage       413    15    11    2    21   
   
      Peptides:   
        Query    Observed    Mr(expt)    Mr(calc)    Score    Peptide    Result File   
		    521    1086.44    2170.86    2170.04    29    ATYTMIFDHYEQVPASVAK    biorepCdiff_7   
		    129    681.54    1361.07    1361.74    39    EDSFIGIIDLLK    biorepCdiffA_7   
		    210    679.57    1357.13    1356.64    38    GGVEPQSENVWR    biorepCdiff_7   
		    79    580.43    1158.84    1158.55    27    GILADGEEAER    biorepCdiffA_7   
		    314    782.82    1563.63    1563.72    29    HSSDEEPFSALAFK    biorepCdiff_7   
		    509    713.51    2137.51    2136.87    39    IGETHEGASQMDWMEQEK +2 Oxidation (M)    biorepCdiff_7   
		    161    625.49    1248.97    1248.62    37    LAEEDPTFTVK    biorepCdiff_7   
		    285    741.64    1481.27    1480.81    50    LNSNAVPMQLPIGK    biorepCdiff_7   
		    37    480.86    959.70    959.52    35    VGAPQVAYR    biorepCdiffA_7   
		    266    895.26    1788.50    1787.89    56    VYSGTLESGSYVLNATK    biorepCdiffA_7   
		    200    776.77    1551.52    1550.77    34    YLEGEELTIDELK    biorepCdiffA_7   
   
      Matching Genes:  
               gi|115249074|emb|CAJ66885.1|  (translation elongation factor G [Clostridium difficile 630]) 
           
  Protein Group 17   
      Expression Quality:  
         Score      Num Spectra      Num Peptides      High-Qual Peptides      % Coverage       413    16    10    6    29   
   
      Peptides:   
        Query    Observed    Mr(expt)    Mr(calc)    Score    Peptide    Result File   
		    380    964.80    1927.59    1927.01    43    DKYPGLIFSQILGYGEK    biorepCdiffA_5   
		    275    706.04    1410.07    1409.65    37    ENPNSPLMTTYK +Oxidation (M)    biorepCdiff_5   
		    47    495.37    988.72    988.53    44    IVGEAMLEK    biorepCdiffA_6   
		    322    739.20    1476.38    1475.80    40    LLSEADIFVTNVR    biorepCdiff_5   
		    97    519.94    1037.86    1037.52    34    MGIAYDQIK    biorepCdiff_5   
		    147    609.91    1217.81    1217.61    42    MLGDWGAEVIK    biorepCdiff_5   
		    160    617.98    1233.95    1233.61    36    MLGDWGAEVIK +Oxidation (M)    biorepCdiff_5   
		    452    1061.76    2121.51    2120.93    33    SPASDDENPMFELENGNKK    biorepCdiffA_5   
		    512    1053.28    2104.55    2105.02    60    TLDEWSALLEEADLPFEK    biorepCdiff_6   
		    196    695.72    1389.42    1388.78    44    WIQLALIQYNK    biorepCdiffA_5   
   
      Matching Genes:  
               gi|115249401|emb|CAJ67216.1|  (isocaprenoyl-CoA:2-hydroxyisocaproate CoA-transferase [Clostridium difficile 630]) 
           
  Protein Group 18   
      Expression Quality:  
         Score      Num Spectra      Num Peptides      High-Qual Peptides      % Coverage       398    18    7    7    50   
   
      Peptides:   
        Query    Observed    Mr(expt)    Mr(calc)    Score    Peptide    Result File   
		    51    457.46    912.90    912.56    52    AVLELAGLK    biorepCdiffA_3   
		    607    637.43    1909.27    1909.01    41    FAALVVVGDENGHVGIGAGK    biorepCdiff_3   
		    738    1231.92    2461.82    2461.29    68    GHFGAGNILIMPAVEGTGVIAGGPAR    biorepCdiff_3   
		    739    1240.02    2478.03    2477.28    56    GHFGAGNILIMPAVEGTGVIAGGPAR +Oxidation (M)    biorepCdiff_3   
		    435    728.08    1454.15    1453.78    72    KPIDAGQLDLQEK    biorepCdiff_3   
		    584    929.39    1856.77    1856.09    57    NLIVVPIVGTTIPHEVR    biorepCdiff_3   
		    538    537.79    1610.35    1609.88    52    RKPIDAGQLDLQEK    biorepCdiff_2   
   
      Matching Genes:  
               gi|115249094|emb|CAJ66905.1|  (30S ribosomal protein S5 [Clostridium difficile 630]) 
           
  Protein Group 19   
      Expression Quality:  
         Score      Num Spectra      Num Peptides      High-Qual Peptides      % Coverage       353    12    7    3    41   
   
      Peptides:   
        Query    Observed    Mr(expt)    Mr(calc)    Score    Peptide    Result File   
		    304    797.08    1592.15    1591.86    65    APVSNFAYLIDAIAK    biorepCdiffA_3   
		    209    687.46    1372.91    1372.76    56    GLTVEEVTELRK    biorepCdiffA_3   
		    113    565.50    1128.99    1128.60    30    IVEMANIPSR    biorepCdiffA_3   
		    221    573.39    1144.77    1144.59    39    IVEMANIPSR +Oxidation (M)    biorepCdiff_3   
		    432    723.50    1444.99    1444.65    37    MGIVEGAFYDESK    biorepCdiff_3   
		    110    559.93    1117.85    1117.59    38    SEVVSEIVEK    biorepCdiffA_3   
		    191    520.04    1038.08    1037.54    88    SSAAVVVDYK    biorepCdiff_3   
   
      Matching Genes:  
               gi|115249067|emb|CAJ66878.1|  (50S ribosomal protein L10 [Clostridium difficile 630]) 
           
  Protein Group 20   
      Expression Quality:  
         Score      Num Spectra      Num Peptides      High-Qual Peptides      % Coverage       347    26    6    5    34   
   
      Peptides:   
        Query    Observed    Mr(expt)    Mr(calc)    Score    Peptide    Result File   
		    495    970.46    1938.91    1937.95    82    AFGGADTWATSNTIAAGISK    biorepCdiff_4   
		    453    694.54    2080.61    2080.03    39    DGVPSILNPDDANALEEALK    biorepCdiffA_3   
		    256    871.15    1740.29    1739.87    68    QAIDGDTAQVGPQIAEK    biorepCdiffA_6   
		    84    604.44    1206.87    1206.61    58    QLEDGYELIK    biorepCdiffA_4   
		    255    613.55    1225.08    1224.61    55    VGDYDIIFAGR    biorepCdiff_3   
		    65    564.57    1127.12    1126.70    45    VSTPVLLTAVK    biorepCdiffA_4   
   
      Matching Genes:  
               gi|115249406|emb|CAJ67221.1|  (electron transfer flavoprotein beta-subunit [Clostridium difficile 630]) 
           
  Protein Group 21   
      Expression Quality:  
         Score      Num Spectra      Num Peptides      High-Qual Peptides      % Coverage       336    11    7    5    31   
   
      Peptides:   
        Query    Observed    Mr(expt)    Mr(calc)    Score    Peptide    Result File   
		    77    494.49    986.97    986.51    32    EVAPALMEK    biorepCdiffA_3   
		    235    598.51    1195.01    1194.63    45    GMDIIFVTTAK    biorepCdiff_3   
		    381    929.78    1857.55    1856.99    60    GVEELEMISGQKPVITK    biorepCdiffA_3   
		    596    937.80    1873.58    1872.99    50    GVEELEMISGQKPVITK +Oxidation (M)    biorepCdiff_3   
		    120    579.95    1157.89    1157.62    63    IVINMGIGDAR    biorepCdiffA_3   
		    124    587.98    1173.94    1173.62    39    IVINMGIGDAR +Oxidation (M)    biorepCdiffA_3   
		    31    435.92    869.82    869.53    47    LVSVSLPR    biorepCdiffA_3   
   
      Matching Genes:  
               gi|115249089|emb|CAJ66900.1|  (50S ribosomal protein L5 [Clostridium difficile 630]) 
           
  Protein Group 22   
      Expression Quality:  
         Score      Num Spectra      Num Peptides      High-Qual Peptides      % Coverage       330    15    7    4    38   
   
      Peptides:   
        Query    Observed    Mr(expt)    Mr(calc)    Score    Peptide    Result File   
		    436    670.23    2007.66    2007.14    43    GVVIPVTAVEAGPMVVTQIK    biorepCdiffA_3   
		    642    1012.89    2023.77    2023.14    33    GVVIPVTAVEAGPMVVTQIK +Oxidation (M)    biorepCdiff_3   
		    10    416.84    831.66    831.47    32    IDVTGISK    biorepCdiffA_3   
		    630    992.75    1983.49    1982.96    66    TVDKDGYNAIQIGFEDAK    biorepCdiff_3   
		    283    762.50    1522.98    1522.75    61    VDSVEGYTVGQEIK    biorepCdiffA_3   
		    116    570.39    1138.77    1138.57    68    VGMTQIFTDK    biorepCdiffA_3   
		    163    565.09    1128.16    1127.65    27    VTVQNLEVVK    biorepCdiff_4   
   
      Matching Genes:  
               gi|115249077|emb|CAJ66888.1|  (50S ribosomal protein L3 [Clostridium difficile 630]) 
           
  Protein Group 23   
      Expression Quality:  
         Score      Num Spectra      Num Peptides      High-Qual Peptides      % Coverage       328    11    5    5    28   
   
      Peptides:   
        Query    Observed    Mr(expt)    Mr(calc)    Score    Peptide    Result File   
		    438    699.59    1397.17    1396.76    63    AEIGLTYIYGIGK    biorepCdiff_2   
		    168    659.40    1316.78    1316.62    69    DLSEDQVNELR    biorepCdiffA_2   
		    238    723.50    1444.99    1444.72    45    DLSEDQVNELRK    biorepCdiffA_2   
		    25    420.86    839.70    839.49    62    IAGVDLPR    biorepCdiffA_2   
		    526    780.05    1558.08    1557.80    89    IKDLSEDQVNELR    biorepCdiff_2   
   
      Matching Genes:  
               gi|115249103|emb|CAJ66914.1|  (30S ribosomal protein S13 [Clostridium difficile 630]) 
           
  Protein Group 24   
      Expression Quality:  
         Score      Num Spectra      Num Peptides      High-Qual Peptides      % Coverage       328    11    6    6    26   
   
      Peptides:   
        Query    Observed    Mr(expt)    Mr(calc)    Score    Peptide    Result File   
		    245    607.92    1213.83    1213.67    75    AGENLLSLLER    biorepCdiff_3   
		    489    783.04    1564.07    1564.81    72    QLVTHGHFTLNGNK    biorepCdiff_3   
		    74    492.40    982.79    982.61    40    VDIPSLIVK    biorepCdiffA_3   
		    92    525.37    1048.72    1048.57    40    VSNYGLQLR    biorepCdiffA_3   
		    364    675.01    1348.01    1347.65    58    WLEANVEGMTAK    biorepCdiff_3   
		    374    683.00    1363.98    1363.64    43    WLEANVEGMTAK +Oxidation (M)    biorepCdiff_3   
   
      Matching Genes:  
               gi|115249105|emb|CAJ66916.1|  (30S ribosomal protein S4 [Clostridium difficile 630]) 
           
  Protein Group 25   
      Expression Quality:  
         Score      Num Spectra      Num Peptides      High-Qual Peptides      % Coverage       318    12    5    4    27   
   
      Peptides:   
        Query    Observed    Mr(expt)    Mr(calc)    Score    Peptide    Result File   
		    375    616.54    1231.06    1230.61    44    AADDAAGLAISEK    biorepCdiff_1   
		    260    558.60    1672.78    1671.87    30    IADELTQLKDEIER    biorepCdiffA_4   
		    389    839.66    1677.30    1676.84    48    IRDTDVASEMVNLSK    biorepCdiff_5   
		    452    1159.82    2317.62    2317.11    124    LESTQNNLNNTLENVTAAESR    biorepCdiffA_4   
		    297    910.24    1818.47    1817.88    72    TLSLQSANEINNTEER    biorepCdiffA_4   
   
      Matching Genes:  
               gi|115249247|emb|CAJ67060.1|  (flagellin subunit [Clostridium difficile 630]) 
           
  Protein Group 26   
      Expression Quality:  
         Score      Num Spectra      Num Peptides      High-Qual Peptides      % Coverage       318    11    8    4    20   
   
      Peptides:   
        Query    Observed    Mr(expt)    Mr(calc)    Score    Peptide    Result File   
		    184    641.51    1281.00    1280.66    40    ESTIEFLTSVR    biorepCdiff_6   
		    88    592.39    1182.77    1182.63    52    GISDFLLSFGK    biorepCdiffA_6   
		    301    759.13    1516.24    1515.83    30    GNVLEGLKPESVFK    biorepCdiff_6   
		    596    1151.74    2301.46    2301.01    52    HEFTTSDPGMTYSVAETSVDK    biorepCdiff_6   
		    600    1159.81    2317.61    2317.01    32    HEFTTSDPGMTYSVAETSVDK +Oxidation (M)    biorepCdiff_6   
		    277    485.74    1454.20    1453.76    28    NVEHDFLKDPIK    biorepCdiff_6   
		    191    648.02    1294.02    1293.66    35    TCVVSLPVEYK    biorepCdiff_6   
		    469    990.29    1978.56    1978.03    49    VLSVLNVDYELASVDGGTK    biorepCdiff_6   
   
      Matching Genes:  
               gi|115249724|emb|CAJ67541.1|  (putative aminoacyl-histidine dipeptidase [Clostridium difficile 630]) 
           
  Protein Group 27   
      Expression Quality:  
         Score      Num Spectra      Num Peptides      High-Qual Peptides      % Coverage       318    18    7    4    37   
   
      Peptides:   
        Query    Observed    Mr(expt)    Mr(calc)    Score    Peptide    Result File   
		    17    416.89    831.76    831.45    37    CLALLDK    biorepCdiff_4   
		    541    692.32    2073.94    2073.12    50    ILIPMINEAVGIYADGVASK    biorepCdiff_4   
		    657    922.74    2765.19    2764.33    26    LAVIGSGTMGSGIVQTFASCGHDVCLK    biorepCdiff_4   
		    29    445.75    889.49    889.46    33    LLDELCK    biorepCdiffA_4   
		    701    1052.23    3153.68    3152.60    46    LLDELCKEDTILATNTSSLSITEIASSTK    biorepCdiff_4   
		    121    637.50    1272.99    1272.73    53    LVEVISGQLTSK    biorepCdiffA_4   
		    130    643.53    1285.05    1284.66    73    VTFDTVFELSK    biorepCdiffA_4   
   
      Matching Genes:  
               gi|115250079|emb|CAJ67899.1|  (3-hydroxybutyryl-CoA dehydrogenase [Clostridium difficile 630]) 
           
  Protein Group 28   
      Expression Quality:  
         Score      Num Spectra      Num Peptides      High-Qual Peptides      % Coverage       316    13    7    4    35   
   
      Peptides:   
        Query    Observed    Mr(expt)    Mr(calc)    Score    Peptide    Result File   
		    156    443.72    1328.12    1327.73    31    ALENVLKDDLAK    biorepCdiffA_4   
		    80    596.48    1190.95    1190.62    32    EALEFVNEIK    biorepCdiffA_4   
		    187    611.03    1220.05    1219.62    54    GLYGELANEVR    biorepCdiff_4   
		    142    652.44    1302.87    1302.60    52    IGAQNMHFEEK    biorepCdiffA_4   
		    235    808.53    1615.04    1614.75    58    QYFNETDETVNKK    biorepCdiffA_4   
		    249    820.12    1638.22    1637.79    60    TATAEDANDVISYIR    biorepCdiffA_4   
		    386    802.17    1602.33    1601.88    29    VVVAYEPIWAIGTGK    biorepCdiff_4   
   
      Matching Genes:  
               gi|115252229|emb|CAJ70069.1|  (triosephosphate isomerase [Clostridium difficile 630]) 
           
  Protein Group 29   
      Expression Quality:  
         Score      Num Spectra      Num Peptides      High-Qual Peptides      % Coverage       314    12    7    3    29   
   
      Peptides:   
        Query    Observed    Mr(expt)    Mr(calc)    Score    Peptide    Result File   
		    489    743.02    2226.04    2225.15    28    DAIDEIKPEIMLFGATHIGR    biorepCdiffA_5   
		    132    497.41    992.80    992.52    36    ELITFGADK    biorepCdiff_4   
		    235    683.53    1365.05    1364.80    68    IAPVVIELLGEGR    biorepCdiff_6   
		    169    701.52    1401.03    1400.74    48    LDSVDDLLEAIKA    biorepCdiffA_6   
		    538    1006.59    3016.76    3015.60    38    NPAAPILEIADYGVVGDLHEIVPMLIEK    biorepCdiffA_4   
		    133    581.96    1161.90    1161.59    27    NVWIFAEQR    biorepCdiff_5   
		    79    554.90    1107.78    1107.58    69    TGEVIALDYK    biorepCdiffA_5   
   
      Matching Genes:  
               gi|115249407|emb|CAJ67222.1|  (electron transfer flavoprotein alpha-subunit [Clostridium difficile 630]) 
           
  Protein Group 30   
      Expression Quality:  
         Score      Num Spectra      Num Peptides      High-Qual Peptides      % Coverage       310    15    7    5    40   
   
      Peptides:   
        Query    Observed    Mr(expt)    Mr(calc)    Score    Peptide    Result File   
		    483    733.18    1464.34    1463.75    41    FENGTEITAELLK    biorepCdiff_2   
		    193    486.85    971.69    971.53    46    ILGEGNLEK    biorepCdiff_2   
		    171    440.63    1318.87    1318.74    25    LHELKPAEGAVR    biorepCdiffA_2   
		    536    532.38    1594.11    1593.87    30    MKLHELKPAEGAVR +Oxidation (M)    biorepCdiff_2   
		    305    631.43    1260.85    1260.63    61    VGFEGGQMPLAR    biorepCdiff_2   
		    320    639.47    1276.93    1276.62    51    VGFEGGQMPLAR +Oxidation (M)    biorepCdiff_2   
		    234    718.06    1434.11    1433.75    56    VYTEVNVEVLNR    biorepCdiffA_2   
   
      Matching Genes:  
               gi|115249096|emb|CAJ66907.1|  (50S ribosomal protein L15 [Clostridium difficile 630]) 
           
  Protein Group 31   
      Expression Quality:  
         Score      Num Spectra      Num Peptides      High-Qual Peptides      % Coverage       310    11    7    2    20   
   
      Peptides:   
        Query    Observed    Mr(expt)    Mr(calc)    Score    Peptide    Result File   
		    207    652.59    1303.16    1302.71    36    ALQLHGGYGFIK    biorepCdiff_5   
		    176    631.42    1260.83    1260.59    32    ELDTLPAEMDK    biorepCdiff_5   
		    55    523.93    1045.84    1045.56    71    IAMGTLEVGR    biorepCdiffA_7   
		    104    532.02    1062.03    1061.55    29    IAMGTLEVGR +Oxidation (M)    biorepCdiff_5   
		    410    627.57    1879.67    1879.08    74    IGVAALALGIAQGALDEAVK    biorepCdiff_7   
		    224    485.13    1452.37    1451.75    34    IVSIYEGTSEVQK    biorepCdiffA_5   
		    89    495.93    989.84    989.56    34    MVISSNVLK    biorepCdiff_5   
   
      Matching Genes:  
               gi|115249405|emb|CAJ67220.1|  (acyl-CoA dehydrogenase, short-chain specific [Clostridium difficile 630]) 
           
  Protein Group 32   
      Expression Quality:  
         Score      Num Spectra      Num Peptides      High-Qual Peptides      % Coverage       305    15    7    4    25   
   
      Peptides:   
        Query    Observed    Mr(expt)    Mr(calc)    Score    Peptide    Result File   
		    747    1058.90    3173.69    3173.59    52    ALEAANMTIEDIDLVEANEAFAAQSVAVIR    biorepCdiff_5   
		    189    738.08    1474.14    1473.69    50    EEQDELALASQNK    biorepCdiffA_6   
		    141    644.50    1286.98    1286.71    47    FDEEIVPVVIK    biorepCdiffA_5   
		    152    612.97    1223.93    1223.68    34    ILTTLLYEMK    biorepCdiff_6   
		    121    567.42    1132.83    1132.60    39    IMGYGPVPATK    biorepCdiff_5   
		    157    616.45    1230.89    1230.68    38    SVSAVELGVTAAK    biorepCdiff_6   
		    66    521.46    1040.90    1040.53    45    TAVGSFGGAFK    biorepCdiffA_5   
   
      Matching Genes:  
               gi|115250080|emb|CAJ67900.1|  (acetyl-CoA acetyltransferase [Clostridium difficile 630]) 
           
  Protein Group 33   
      Expression Quality:  
         Score      Num Spectra      Num Peptides      High-Qual Peptides      % Coverage       290    10    8    2    29   
   
      Peptides:   
        Query    Observed    Mr(expt)    Mr(calc)    Score    Peptide    Result File   
		    337    748.28    1494.54    1493.72    30    DVNVFEMAQSQVK    biorepCdiff_5   
		    42    464.70    927.38    927.47    27    GYIDGIYK    biorepCdiffA_5   
		    244    682.11    1362.20    1361.73    46    LGMEPAVYELLK    biorepCdiff_5   
		    594    1130.95    2259.89    2259.22    43    LTGQSSIGVITGKPVEFGGSLGR    biorepCdiff_5   
		    587    1138.86    2275.70    2275.04    39    LVCEAANGPTTPEADEVFAER    biorepCdiff_6   
		    650    1285.59    2569.16    2568.11    39    SEGSYAIYNENGLDGQAMLDYMK    biorepCdiff_5   
		    149    611.41    1220.80    1220.65    27    TAATGFGVAVTAR    biorepCdiff_5   
		    45    492.40    982.78    982.61    39    VIEVSIPVK    biorepCdiffA_6   
   
      Matching Genes:  
               gi|115249189|emb|CAJ67001.1|  (NAD-specific glutamate dehydrogenase [Clostridium difficile 630]) 
           
  Protein Group 34   
      Expression Quality:  
         Score      Num Spectra      Num Peptides      High-Qual Peptides      % Coverage       284    13    6    6    28   
   
      Peptides:   
        Query    Observed    Mr(expt)    Mr(calc)    Score    Peptide    Result File   
		    469    756.62    1511.23    1510.87    51    ALVVIADKNDNVIK    biorepCdiff_3   
		    243    723.47    1444.92    1444.77    43    EFAQILNNINAAK    biorepCdiffA_3   
		    700    1118.32    2234.62    2234.16    57    NIEGVQTALVNTMNVYDILK    biorepCdiff_3   
		    505    1126.40    2250.79    2250.16    49    NIEGVQTALVNTMNVYDILK +Oxidation (M)    biorepCdiffA_3   
		    328    838.19    1674.37    1673.91    44    TKEFAQILNNINAAK    biorepCdiffA_3   
		    208    687.05    1372.08    1371.69    40    YDSFIITTDAVK    biorepCdiffA_3   
   
      Matching Genes:  
               gi|115249078|emb|CAJ66889.1|  (50S ribosomal protein L4 [Clostridium difficile 630]) 
           
  Protein Group 35   
      Expression Quality:  
         Score      Num Spectra      Num Peptides      High-Qual Peptides      % Coverage       274    9    5    4    45   
   
      Peptides:   
        Query    Observed    Mr(expt)    Mr(calc)    Score    Peptide    Result File   
		    482    664.52    1327.02    1326.62    74    HETVDVPASNMK    biorepCdiff_1   
		    105    545.46    1088.91    1088.62    46    ILLEEGFIR    biorepCdiffA_1   
		    274    777.10    1552.19    1551.74    29    KENVGGEVICYVW    biorepCdiffA_2   
		    191    683.02    1364.02    1363.65    53    TMTDPIADMLTR    biorepCdiffA_1   
		    200    694.55    1387.09    1386.81    72    VLNGLGISVISTSK    biorepCdiffA_1   
   
      Matching Genes:  
               gi|115249091|emb|CAJ66902.1|  (30S ribosomal protein S8 [Clostridium difficile 630]) 
           
  Protein Group 36   
      Expression Quality:  
         Score      Num Spectra      Num Peptides      High-Qual Peptides      % Coverage       274    9    4    4    9   
   
      Peptides:   
        Query    Observed    Mr(expt)    Mr(calc)    Score    Peptide    Result File   
		    257    872.16    1742.30    1741.87    78    IENQEGVENLDEILK    biorepCdiffA_6   
		    38    483.92    965.82    965.59    47    INLPAITPK    biorepCdiffA_7   
		    308    765.60    1529.19    1528.81    51    SGDSILIDDGLVGLR    biorepCdiff_6   
		    225    674.97    1347.92    1347.65    98    SSVAGNTDEVIEK    biorepCdiff_6   
   
      Matching Genes:  
               gi|115252454|emb|CAJ70297.1|  (pyruvate kinase [Clostridium difficile 630]) 
           
  Protein Group 37   
      Expression Quality:  
         Score      Num Spectra      Num Peptides      High-Qual Peptides      % Coverage       269    10    6    4    20   
   
      Peptides:   
        Query    Observed    Mr(expt)    Mr(calc)    Score    Peptide    Result File   
		    304    856.66    1711.31    1710.77    43    ELEEICGYEIEEAK    biorepCdiffA_5   
		    301    852.38    1702.75    1701.73    31    FCDPEEYDYPLVR    biorepCdiffA_5   
		    254    763.52    1525.02    1524.73    56    IHESIEVYNEHR    biorepCdiffA_5   
		    426    884.72    1767.42    1766.79    58    QWSNIEGCSLAYDPK    biorepCdiff_5   
		    119    630.04    1258.07    1257.58    39    TDVPAGDDALER    biorepCdiffA_5   
		    87    565.80    1129.59    1128.68    42    VLLTGILADSK    biorepCdiffA_5   
   
      Matching Genes:  
               gi|115249404|emb|CAJ67219.1|  (subunit of oxygen-sensitive 2-hydroxyisocaproyl-CoA dehydratase [Clostridium difficile 630]) 
           
  Protein Group 38   
      Expression Quality:  
         Score      Num Spectra      Num Peptides      High-Qual Peptides      % Coverage       261    7    7    3    15   
   
      Peptides:   
        Query    Observed    Mr(expt)    Mr(calc)    Score    Peptide    Result File   
		    108    646.96    1291.91    1291.61    35    ELISNGCDAVSK    biorepCdiffA_7   
		    114    657.58    1313.14    1312.80    27    EVIPEFLLLLK    biorepCdiffA_7   
		    302    769.63    1537.25    1536.79    33    LYNNQVFVADNIK    biorepCdiff_7   
		    384    895.28    1788.54    1787.87    52    NEDTPAMVLVSEQSIR    biorepCdiff_7   
		    122    671.00    1339.98    1339.77    40    TLVINENSPIIK    biorepCdiffA_7   
		    279    931.59    1861.17    1860.93    29    VFYVSDKEQQSQYIK    biorepCdiffA_7   
		    400    925.72    1849.42    1848.87    45    YINQVAFSGAEDFFNK    biorepCdiff_7   
   
      Matching Genes:  
               gi|115249282|emb|CAJ67095.1|  (chaperone protein (heat shock protein) [Clostridium difficile 630]) 
           
  Protein Group 39   
      Expression Quality:  
         Score      Num Spectra      Num Peptides      High-Qual Peptides      % Coverage       256    14    5    5    26   
   
      Peptides:   
        Query    Observed    Mr(expt)    Mr(calc)    Score    Peptide    Result File   
		    294    905.19    1808.36    1807.91    77    AFAGADTWATSSALAGALK    biorepCdiffA_4   
		    661    1041.33    2080.65    2080.07    41    DGVPSIINPDDKAGLEEAIK    biorepCdiff_3   
		    139    650.53    1299.05    1298.67    46    DIEVDPSNLGLK    biorepCdiffA_4   
		    104    550.49    1098.96    1098.60    51    LDPNTGTLIR    biorepCdiffA_3   
		    99    538.91    1075.81    1075.58    41    MPCLITTLK    biorepCdiffA_3   
   
      Matching Genes:  
               gi|115250076|emb|CAJ67896.1|  (electron transfer flavoprotein beta-subunit [Clostridium difficile 630]) 
           
  Protein Group 40   
      Expression Quality:  
         Score      Num Spectra      Num Peptides      High-Qual Peptides      % Coverage       254    8    6    4    21   
   
      Peptides:   
        Query    Observed    Mr(expt)    Mr(calc)    Score    Peptide    Result File   
		    405    903.17    1804.33    1803.94    41    AGFVVSDSNIKPDNTLK    biorepCdiff_6   
		    340    815.67    1629.32    1628.81    50    EGFLYLAEAGADFVK    biorepCdiff_6   
		    142    672.48    1342.94    1342.72    60    GQATSIIEVAQAR    biorepCdiffA_6   
		    320    981.21    1960.40    1959.95    30    LSFEEGVDSYVPYAGSLK    biorepCdiffA_6   
		    503    1354.49    2706.97    2706.28    33    VPALVEAGADVLCIDSSEGFSEWQK    biorepCdiffA_6   
		    286    918.74    1835.47    1834.85    40    VSEFMTPMSSIVYANK +2 Oxidation (M)    biorepCdiffA_6   
   
      Matching Genes:  
               gi|115251390|emb|CAJ69222.1|  (inosine-5'-monophosphate dehydrogenase [Clostridium difficile 630]) 
           
  Protein Group 41   
      Expression Quality:  
         Score      Num Spectra      Num Peptides      High-Qual Peptides      % Coverage       241    8    5    4    39   
   
      Peptides:   
        Query    Observed    Mr(expt)    Mr(calc)    Score    Peptide    Result File   
		    586    734.19    1466.36    1465.78    46    AGSQVSGPVPLPTEK    biorepCdiff_1   
		    274    798.09    1594.17    1593.87    72    KAGSQVSGPVPLPTEK    biorepCdiffA_1   
		    99    541.49    1080.97    1080.62    49    LIDIANPTPK    biorepCdiffA_1   
		    25    425.91    849.80    849.46    41    LLDFSAGK    biorepCdiffA_1   
		    11    415.17    828.33    827.52    33    QVVTILR    biorepCdiffA_1   
   
      Matching Genes:  
               gi|115249076|emb|CAJ66887.1|  (30S ribosomal protein S10 [Clostridium difficile 630]) 
           
  Protein Group 42   
      Expression Quality:  
         Score      Num Spectra      Num Peptides      High-Qual Peptides      % Coverage       231    9    5    3    34   
   
      Peptides:   
        Query    Observed    Mr(expt)    Mr(calc)    Score    Peptide    Result File   
		    640    703.25    2106.72    2105.99    80    MPDLNAASVEAAMSMIAGTAR    biorepCdiff_2   
		    652    713.98    2138.92    2137.98    48    MPDLNAASVEAAMSMIAGTAR +2 Oxidation (M)    biorepCdiff_2   
		    634    1045.88    2089.74    2089.09    43    TADQAGMIIPVVITVYQDR    biorepCdiff_2   
		    637    1053.88    2105.74    2105.08    33    TADQAGMIIPVVITVYQDR +Oxidation (M)    biorepCdiff_2   
		    65    455.35    908.69    908.57    27    TPPAAVLIK    biorepCdiffA_2   
   
      Matching Genes:  
               gi|115249065|emb|CAJ66876.1|  (50S ribosomal protein L11 [Clostridium difficile 630]) 
           
  Protein Group 43   
      Expression Quality:  
         Score      Num Spectra      Num Peptides      High-Qual Peptides      % Coverage       221    8    5    3    29   
   
      Peptides:   
        Query    Observed    Mr(expt)    Mr(calc)    Score    Peptide    Result File   
		    139    612.99    1223.96    1223.63    46    AGATYVSPFVGR    biorepCdiffA_3   
		    680    1083.30    2164.58    2164.13    49    EISEIVDGPISAEVISLEHK    biorepCdiff_3   
		    237    720.58    1439.14    1438.73    64    FFIDTANIEEIK    biorepCdiffA_3   
		    146    480.45    958.89    958.52    36    IPMTAEGLK    biorepCdiff_3   
		    213    559.97    1117.93    1117.64    26    NPIHVLQAAR    biorepCdiff_3   
   
      Matching Genes:  
               gi|115251384|emb|CAJ69216.1|  (putative transaldolase [Clostridium difficile 630]) 
           
  Protein Group 44   
      Expression Quality:  
         Score      Num Spectra      Num Peptides      High-Qual Peptides      % Coverage       214    4    4    3    4   
   
      Peptides:   
        Query    Observed    Mr(expt)    Mr(calc)    Score    Peptide    Result File   
		    181    687.13    1372.24    1371.72    77    IGAEVDSGDILVGK    biorepCdiff_8   
		    278    824.06    1646.11    1645.81    60    SNSGTCINQTPIINK    biorepCdiff_8   
		    180    817.02    1632.03    1631.85    46    STGPYSLVTQQPLGGK    biorepCdiffA_8   
		    71    501.45    1000.89    1000.58    31    VTADEIIIK    biorepCdiff_8   
   
      Matching Genes:  
               gi|115249070|emb|CAJ66881.1|  (DNA-directed RNA polymerase beta chain [Clostridium difficile 630]) 
           
  Protein Group 45   
      Expression Quality:  
         Score      Num Spectra      Num Peptides      High-Qual Peptides      % Coverage       214    8    5    2    40   
   
      Peptides:   
        Query    Observed    Mr(expt)    Mr(calc)    Score    Peptide    Result File   
		    107    556.41    1110.80    1110.64    33    ADLDLRPALK    biorepCdiffA_2   
		    293    823.70    1645.38    1644.81    39    ALENYFNYETLIR    biorepCdiffA_1   
		    166    656.48    1310.95    1310.73    56    LVAGEGNILVNGR    biorepCdiffA_2   
		    123    606.93    1211.84    1211.65    37    QPLVLTGNENK    biorepCdiffA_2   
		    376    965.73    1929.45    1929.06    49    QPLVLTGNENKYDVIVK    biorepCdiffA_2   
   
      Matching Genes:  
               gi|115249113|emb|CAJ66924.1|  (30S ribosomal protein S9 [Clostridium difficile 630]) 
           
  Protein Group 46   
      Expression Quality:  
         Score      Num Spectra      Num Peptides      High-Qual Peptides      % Coverage       209    6    5    3    23   
   
      Peptides:   
        Query    Observed    Mr(expt)    Mr(calc)    Score    Peptide    Result File   
		    162    634.47    1266.93    1266.73    50    ALVPVVVEQTGR    biorepCdiffA_3   
		    111    563.42    1124.83    1124.52    34    DNFMSALEAK    biorepCdiffA_3   
		    239    602.43    1202.85    1202.61    45    ETLNEILSER    biorepCdiff_3   
		    186    657.50    1312.98    1312.66    45    EYGLIDEVFTK    biorepCdiffA_3   
		    241    723.05    1444.08    1443.79    35    IKETLNEILSER    biorepCdiffA_3   
   
      Matching Genes:  
               gi|115252361|emb|CAJ70202.1|  (ATP-dependent Clp protease proteolytic subunit [Clostridium difficile 630]) 
           
  Protein Group 47   
      Expression Quality:  
         Score      Num Spectra      Num Peptides      High-Qual Peptides      % Coverage       207    7    5    3    17   
   
      Peptides:   
        Query    Observed    Mr(expt)    Mr(calc)    Score    Peptide    Result File   
		    86    561.01    1120.01    1119.58    45    FEGETLPSLK    biorepCdiffA_5   
		    228    731.61    1461.20    1460.73    46    LGWTCASPEILSK    biorepCdiffA_5   
		    152    613.01    1224.01    1223.55    34    LNYSNMPEEK    biorepCdiff_5   
		    289    826.12    1650.22    1649.82    33    MIYVIPDFQNPTGR    biorepCdiffA_5   
		    627    1204.46    2406.90    2406.20    49    TNVNKDDILVTSGSQQGLDFAGK    biorepCdiff_5   
   
      Matching Genes:  
               gi|115252729|emb|CAJ70573.1|  (putative amino acid aminotransferase [Clostridium difficile 630]) 
           
  Protein Group 48   
      Expression Quality:  
         Score      Num Spectra      Num Peptides      High-Qual Peptides      % Coverage       205    8    4    3    19   
   
      Peptides:   
        Query    Observed    Mr(expt)    Mr(calc)    Score    Peptide    Result File   
		    292    903.77    1805.53    1804.98    34    ATIGQVGNIEHGNVVIGK    biorepCdiffA_4   
		    146    531.98    1061.95    1061.55    51    SAGVSAQLMAK    biorepCdiff_4   
		    252    825.13    1648.24    1647.86    70    TANIALLNYADGEKR    biorepCdiffA_4   
		    176    589.42    1176.82    1176.58    50    VATIEYDPNR    biorepCdiff_4   
   
      Matching Genes:  
               gi|115249080|emb|CAJ66891.1|  (50S ribosomal protein L2 [Clostridium difficile 630]) 
           
  Protein Group 49   
      Expression Quality:  
         Score      Num Spectra      Num Peptides      High-Qual Peptides      % Coverage       199    6    5    1    13   
   
      Peptides:   
        Query    Observed    Mr(expt)    Mr(calc)    Score    Peptide    Result File   
		    307    638.89    1913.64    1912.98    35    AIGDNLTCIFVDHGLLR    biorepCdiffA_6   
		    206    774.54    1547.06    1546.66    34    AVTSSDGMTSDWYK    biorepCdiffA_6   
		    50    445.91    889.80    889.50    33    FDINLIR    biorepCdiff_6   
		    538    1084.23    2166.45    2166.08    67    GIIFTGGPNSAYLEDSPTISK    biorepCdiff_6   
		    88    488.87    975.73    975.54    30    IIGEEFIR    biorepCdiff_6   
   
      Matching Genes:  
               gi|115249206|emb|CAJ67019.1|  (GMP synthase [glutamine-hydrolyzing] [Clostridium difficile 630]) 
           
  Protein Group 50   
      Expression Quality:  
         Score      Num Spectra      Num Peptides      High-Qual Peptides      % Coverage       187    6    4    3    5   
   
      Peptides:   
        Query    Observed    Mr(expt)    Mr(calc)    Score    Peptide    Result File   
		    316    526.76    1577.26    1576.73    44    CGTGLASHEVAQGYK    biorepCdiff_7   
		    360    571.43    1711.26    1710.88    49    KGDEVYYVSKPLADK    biorepCdiff_7   
		    157    704.51    1407.01    1406.70    63    LTEELINEGYAR    biorepCdiffA_7   
		    371    873.16    1744.31    1743.81    31    SLEEQNLNDHMTGIK +Oxidation (M)    biorepCdiff_7   
   
      Matching Genes:  
               gi|115251669|emb|CAJ69504.1|  (isoleucyl-tRNA synthetase [Clostridium difficile 630]) 
           
  Protein Group 51   
      Expression Quality:  
         Score      Num Spectra      Num Peptides      High-Qual Peptides      % Coverage       183    6    3    2    11   
   
      Peptides:   
        Query    Observed    Mr(expt)    Mr(calc)    Score    Peptide    Result File   
		    383    831.67    1661.32    1660.76    38    AHCSTVGAGEFLQER    biorepCdiff_5   
		    358    839.17    1676.32    1675.81    99    EVVFAADDNVVGENAK    biorepCdiff_6   
		    332    745.55    1489.08    1488.72    46    MENGDVVLLENTR    biorepCdiff_5   
   
      Matching Genes:  
               gi|115252230|emb|CAJ70070.1|  (phosphoglycerate kinase [Clostridium difficile 630]) 
           
  Protein Group 52   
      Expression Quality:  
         Score      Num Spectra      Num Peptides      High-Qual Peptides      % Coverage       180    7    4    3    15   
   
      Peptides:   
        Query    Observed    Mr(expt)    Mr(calc)    Score    Peptide    Result File   
		    41    479.51    957.00    956.55    46    AVELLLDGK    biorepCdiffA_4   
		    602    1171.60    2341.19    2340.16    50    GLLEEDLTEMNLSSVGDIIHR    biorepCdiff_4   
		    605    786.65    2356.93    2356.16    32    GLLEEDLTEMNLSSVGDIIHR +Oxidation (M)    biorepCdiff_4   
		    447    908.31    1814.61    1813.94    52    TIGLLTSGGDAPGMNAAIR    biorepCdiff_4   
   
      Matching Genes:  
               gi|115252455|emb|CAJ70298.1|  (6-phosphofructokinase [Clostridium difficile 630]) 
           
  Protein Group 53   
      Expression Quality:  
         Score      Num Spectra      Num Peptides      High-Qual Peptides      % Coverage       171    6    4    2    25   
   
      Peptides:   
        Query    Observed    Mr(expt)    Mr(calc)    Score    Peptide    Result File   
		    189    518.32    1034.64    1034.65    34    IILLGPPGAGK    biorepCdiff_3   
		    658    1037.90    2073.78    2073.07    47    IQVYLDETKPLVDYYSK    biorepCdiff_3   
		    571    906.64    1811.27    1810.80    63    VEGVCDVCQGELYQR    biorepCdiff_3   
		    287    511.97    1532.90    1531.78    27    YNIPHISTGDIFR    biorepCdiffA_3   
   
      Matching Genes:  
               gi|115249098|emb|CAJ66909.1|  (adenylate kinase [Clostridium difficile 630]) 
           
  Protein Group 54   
      Expression Quality:  
         Score      Num Spectra      Num Peptides      High-Qual Peptides      % Coverage       171    5    4    2    12   
   
      Peptides:   
        Query    Observed    Mr(expt)    Mr(calc)    Score    Peptide    Result File   
		    133    581.92    1161.82    1161.59    30    FPLDTEAELK    biorepCdiff_6   
		    365    1047.36    2092.70    2092.14    48    HVENITQVYGLPAVVAINR    biorepCdiffA_6   
		    629    1206.40    2410.78    2410.15    28    IYGADGVDYTPEADKEIANLEK    biorepCdiff_6   
		    246    857.14    1712.27    1711.87    65    SDIEIAQEAKPQDIR    biorepCdiffA_6   
   
      Matching Genes:  
               gi|115249735|emb|CAJ67552.1|  (formate--tetrahydrofolate ligase [Clostridium difficile 630]) 
           
  Protein Group 55   
      Expression Quality:  
         Score      Num Spectra      Num Peptides      High-Qual Peptides      % Coverage       170    5    3    3    44   
   
      Peptides:   
        Query    Observed    Mr(expt)    Mr(calc)    Score    Peptide    Result File   
		    388    1018.26    2034.51    2034.06    44    EQPQIAEVVEVGPGGIVEGK    biorepCdiffA_1   
		    130    610.94    1219.87    1220.64    65    IEGQEYTILR    biorepCdiffA_2   
		    102    543.04    1084.07    1083.63    61    TASGIVLPGAAK    biorepCdiffA_1   
   
      Matching Genes:  
               gi|115249203|emb|CAJ67015.1|  (10 kDa chaperonin [Clostridium difficile 630]) 
           
  Protein Group 56   
      Expression Quality:  
         Score      Num Spectra      Num Peptides      High-Qual Peptides      % Coverage       168    6    3    2    27   
   
      Peptides:   
        Query    Observed    Mr(expt)    Mr(calc)    Score    Peptide    Result File   
		    340    858.26    1714.51    1713.88    69    APAFEGNNMVVIIDPK    biorepCdiffA_3   
		    463    749.57    1497.12    1496.79    39    LRPGIEQNDLNTK    biorepCdiff_3   
		    390    940.33    1878.64    1877.97    60    NLDLVQISPNANPPVCK    biorepCdiffA_3   
   
      Matching Genes:  
               gi|115249701|emb|CAJ67518.1|  (translation initiation factor IF-3 [Clostridium difficile 630]) 
           
  Protein Group 57   
      Expression Quality:  
         Score      Num Spectra      Num Peptides      High-Qual Peptides      % Coverage       167    5    3    2    26   
   
      Peptides:   
        Query    Observed    Mr(expt)    Mr(calc)    Score    Peptide    Result File   
		    626    978.29    1954.56    1953.98    68    AIANSDLGLNPSNDGEVIR    biorepCdiff_3   
		    29    435.43    868.85    868.44    39    FEFGTIR    biorepCdiffA_3   
		    732    1194.47    2386.94    2386.22    60    VDYYGTPTPINQIGAISVPEPR    biorepCdiff_3   
   
      Matching Genes:  
               gi|115251191|emb|CAJ69022.1|  (ribosome recycling factor [Clostridium difficile 630]) 
           
  Protein Group 58   
      Expression Quality:  
         Score      Num Spectra      Num Peptides      High-Qual Peptides      % Coverage       163    4    4    2    14   
   
      Peptides:   
        Query    Observed    Mr(expt)    Mr(calc)    Score    Peptide    Result File   
		    523    1040.78    2079.54    2078.92    53    AAEETGLPYAGFDGDQADPR    biorepCdiff_5   
		    150    652.02    1302.03    1301.66    40    IQGLVEVMEER    biorepCdiffA_5   
		    171    674.03    1346.05    1345.68    36    LLIEELEDNMK    biorepCdiffA_5   
		    333    603.54    1807.61    1806.95    34    VVINDLLAEQYANAFK    biorepCdiffA_5   
   
      Matching Genes:  
               gi|115249403|emb|CAJ67218.1|  (subunit of oxygen-sensitive 2-hydroxyisocaproyl-CoA dehydratase [Clostridium difficile 630]) 
           
  Protein Group 59   
      Expression Quality:  
         Score      Num Spectra      Num Peptides      High-Qual Peptides      % Coverage       163    8    3    3    42   
   
      Peptides:   
        Query    Observed    Mr(expt)    Mr(calc)    Score    Peptide    Result File   
		    382    1002.33    2002.64    2002.00    72    LEANAGDVVTLNEVLACSK    biorepCdiffA_1   
		    183    671.61    1341.20    1340.73    50    LGSPVVEGASVQAK    biorepCdiffA_1   
		    273    611.46    1220.91    1220.63    41    VSEGDVLFVEK    biorepCdiff_2   
   
      Matching Genes:  
               gi|115250193|emb|CAJ68014.1|  (50S ribosomal protein L21 [Clostridium difficile 630]) 
           
  Protein Group 60   
      Expression Quality:  
         Score      Num Spectra      Num Peptides      High-Qual Peptides      % Coverage       157    6    4    1    23   
   
      Peptides:   
        Query    Observed    Mr(expt)    Mr(calc)    Score    Peptide    Result File   
		    121    580.53    1159.05    1158.68    33    ALVFENVLVR    biorepCdiffA_3   
		    555    891.69    1781.37    1780.85    58    FKPLSQPGQYACEEK    biorepCdiff_3   
		    21    428.38    854.74    854.52    39    LPIALSNK    biorepCdiffA_3   
		    117    573.41    1144.81    1144.58    27    LVGPAGEVEMK +Oxidation (M)    biorepCdiffA_3   
   
      Matching Genes:  
               gi|115251734|emb|CAJ69569.1|  (putative propanediol utilization protein [Clostridium difficile 630]) 
           
  Protein Group 61   
      Expression Quality:  
         Score      Num Spectra      Num Peptides      High-Qual Peptides      % Coverage       156    5    3    2    26   
   
      Peptides:   
        Query    Observed    Mr(expt)    Mr(calc)    Score    Peptide    Result File   
		    339    580.48    1158.96    1158.62    50    KAGQICDLVR    biorepCdiff_1   
		    103    543.50    1084.98    1084.61    69    NVDEALAILK    biorepCdiffA_1   
		    285    506.38    1010.74    1010.58    37    TSHIEVVVK    biorepCdiff_1   
   
      Matching Genes:  
               gi|115249082|emb|CAJ66893.1|  (50S ribosomal protein L22 [Clostridium difficile 630]) 
           
  Protein Group 62   
      Expression Quality:  
         Score      Num Spectra      Num Peptides      High-Qual Peptides      % Coverage       156    6    3    2    33   
   
      Peptides:   
        Query    Observed    Mr(expt)    Mr(calc)    Score    Peptide    Result File   
		    465    798.62    2392.85    2392.22    34    HKPTFTPHVDGGDFVVVVNAEK    biorepCdiffA_2   
		    344    861.69    1721.37    1720.92    82    KPEEVISHAVSGMLPK    biorepCdiffA_3   
		    229    543.98    1085.95    1085.64    40    LATEIATVLR    biorepCdiff_2   
   
      Matching Genes:  
               gi|115249112|emb|CAJ66923.1|  (50S ribosomal protein L13 [Clostridium difficile 630]) 
           
  Protein Group 63   
      Expression Quality:  
         Score      Num Spectra      Num Peptides      High-Qual Peptides      % Coverage       155    5    3    3    20   
   
      Peptides:   
        Query    Observed    Mr(expt)    Mr(calc)    Score    Peptide    Result File   
		    413    845.12    1688.22    1687.71    47    ALDACEDKEDTMYK    biorepCdiff_4   
		    199    619.55    1237.08    1236.59    48    FGVEEIEAESK    biorepCdiff_4   
		    500    976.18    1950.34    1949.87    60    TDEKEVDDENVTDINSK    biorepCdiff_4   
   
      Matching Genes:  
               gi|115251516|emb|CAJ69349.1|  (heat shock protein [Clostridium difficile 630]) 
           
  Protein Group 64   
      Expression Quality:  
         Score      Num Spectra      Num Peptides      High-Qual Peptides      % Coverage       152    5    3    2    38   
   
      Peptides:   
        Query    Observed    Mr(expt)    Mr(calc)    Score    Peptide    Result File   
		    412    1073.31    2144.60    2144.06    39    FGVSASAPVMVAGAAAGGPAAEEK    biorepCdiffA_1   
		    602    749.20    1496.39    1495.74    67    TEFDVVLTDVGSSK    biorepCdiff_1   
		    92    528.89    1055.76    1055.62    46    VLELNELVK    biorepCdiffA_1   
   
      Matching Genes:  
               gi|115249068|emb|CAJ66879.1|  (50S ribosomal protein L7/L12 [Clostridium difficile 630]) 
           
  Protein Group 65   
      Expression Quality:  
         Score      Num Spectra      Num Peptides      High-Qual Peptides      % Coverage       152    6    3    2    22   
   
      Peptides:   
        Query    Observed    Mr(expt)    Mr(calc)    Score    Peptide    Result File   
		    369    670.54    1339.06    1338.71    31    LYRPANTFVMK    biorepCdiff_2   
		    309    834.06    1666.12    1665.77    57    MLSEMAIQDPEGFAK    biorepCdiffA_2   
		    318    842.12    1682.24    1681.77    64    MLSEMAIQDPEGFAK +Oxidation (M)    biorepCdiffA_2   
   
      Matching Genes:  
               gi|115249703|emb|CAJ67520.1|  (50S ribosomal protein L20 [Clostridium difficile 630]) 
           
  Protein Group 66   
      Expression Quality:  
         Score      Num Spectra      Num Peptides      High-Qual Peptides      % Coverage       152    3    3    2    14   
   
      Peptides:   
        Query    Observed    Mr(expt)    Mr(calc)    Score    Peptide    Result File   
		    75    588.95    1175.90    1175.62    48    IPLTIADYDR    biorepCdiffA_4   
		    282    883.15    1764.29    1763.78    73    NVYVSTDDGTYGFNGR    biorepCdiffA_4   
		    436    593.90    1778.67    1777.89    31    QLTDSIYLMEIEAPR    biorepCdiff_4   
   
      Matching Genes:  
               gi|115250577|emb|CAJ68401.1|  (putative dehydrogenase, electron transfer subunit [Clostridium difficile 630]) 
           
  Protein Group 67   
      Expression Quality:  
         Score      Num Spectra      Num Peptides      High-Qual Peptides      % Coverage       148    4    4    1    14   
   
      Peptides:   
        Query    Observed    Mr(expt)    Mr(calc)    Score    Peptide    Result File   
		    548    1092.34    2182.67    2182.05    51    ELDIDPIDNPDLDIEEISK    biorepCdiff_6   
		    676    1312.88    2623.74    2624.31    35    GENYNLVIGSNTFIPGFEEQLVGK    biorepCdiff_6   
		    56    525.48    1048.94    1049.58    30    IVIETQYGK    biorepCdiffA_6   
		    14    433.93    865.85    865.44    32    YNIPGFR    biorepCdiffA_6   
   
      Matching Genes:  
               gi|115252362|emb|CAJ70203.1|  (trigger factor [Clostridium difficile 630]) 
           
  Protein Group 68   
      Expression Quality:  
         Score      Num Spectra      Num Peptides      High-Qual Peptides      % Coverage       146    4    3    3    20   
   
      Peptides:   
        Query    Observed    Mr(expt)    Mr(calc)    Score    Peptide    Result File   
		    651    904.08    2709.23    2708.25    42    KTEGLQDTQTHYCPGCTHGIIHR    biorepCdiff_4   
		    679    976.91    2927.70    2926.57    40    LVGEVLEELGVLGDAVGVVPVGCSVLGYK    biorepCdiff_4   
		    497    861.42    2581.23    2580.16    64    TEGLQDTQTHYCPGCTHGIIHR    biorepCdiffA_4   
   
      Matching Genes:  
               gi|115249126|emb|CAJ66937.1|  (putative subunit of oxidoreductase [Clostridium difficile 630]) 
           
  Protein Group 69   
      Expression Quality:  
         Score      Num Spectra      Num Peptides      High-Qual Peptides      % Coverage       146    3    3    2    17   
   
      Peptides:   
        Query    Observed    Mr(expt)    Mr(calc)    Score    Peptide    Result File   
		    418    870.72    1739.43    1738.88    61    AAALDTFETEGLFLNK    biorepCdiff_5   
		    394    844.22    1686.43    1685.88    56    GGLINTGDLIEALESGK    biorepCdiff_5   
		    641    1321.67    2641.33    2640.31    29    GLGANVIAFDQYPNSDLNDILTYK    biorepCdiff_7   
   
      Matching Genes:  
               gi|115249400|emb|CAJ67215.1|  ((R)-2-hydroxyisocaproate dehydrogenase [Clostridium difficile 630]) 
           
  Protein Group 70   
      Expression Quality:  
         Score      Num Spectra      Num Peptides      High-Qual Peptides      % Coverage       144    3    3    3    6   
   
      Peptides:   
        Query    Observed    Mr(expt)    Mr(calc)    Score    Peptide    Result File   
		    106    513.89    1025.77    1025.59    41    GIGASPGVALGK    biorepCdiff_6   
		    92    594.93    1187.84    1187.60    51    SIDNVEAEIAK    biorepCdiffA_6   
		    216    791.02    1580.03    1579.77    52    TESVNAEYALNEIK    biorepCdiffA_6   
   
      Matching Genes:  
               gi|115251808|emb|CAJ69643.1|  (phosphoenolpyruvate-protein phosphotransferase [Clostridium difficile 630]) 
           
  Protein Group 71   
      Expression Quality:  
         Score      Num Spectra      Num Peptides      High-Qual Peptides      % Coverage       142    6    3    2    36   
   
      Peptides:   
        Query    Observed    Mr(expt)    Mr(calc)    Score    Peptide    Result File   
		    801    1332.98    2663.95    2663.20    64    DMNEFGAINEVYAEYFGENKPAR    biorepCdiff_1   
		    764    1116.90    2231.78    2231.13    46    HEVIHTNDAPAALGPYSQAIK    biorepCdiff_1   
		    467    1246.38    2490.75    2490.27    32    MKHEVIHTNDAPAALGPYSQAIK    biorepCdiffA_1   
   
      Matching Genes:  
               gi|115251566|emb|CAJ69399.1|  (putative translation inhibitor endoribonuclease [Clostridium difficile 630]) 
           
  Protein Group 72   
      Expression Quality:  
         Score      Num Spectra      Num Peptides      High-Qual Peptides      % Coverage       138    5    3    2    38   
   
      Peptides:   
        Query    Observed    Mr(expt)    Mr(calc)    Score    Peptide    Result File   
		    389    1024.75    2047.49    2046.82    46    DECDNSYIYTFYTNDK    biorepCdiffA_1   
		    676    907.39    1812.76    1812.85    25    EEFEELKNNTYIER    biorepCdiff_1   
		    319    863.58    1725.15    1724.79    67    IVDEYDYGYNAIYK    biorepCdiffA_1   
   
      Matching Genes:  
               gi|115249824|emb|CAJ67641.1|  (hypothetical protein [Clostridium difficile 630]) 
           
  Protein Group 73   
      Expression Quality:  
         Score      Num Spectra      Num Peptides      High-Qual Peptides      % Coverage       136    5    3    1    44   
   
      Peptides:   
        Query    Observed    Mr(expt)    Mr(calc)    Score    Peptide    Result File   
		    120    590.43    1178.84    1178.55    37    DLTSEELMNK    biorepCdiffA_1   
		    231    724.99    1447.96    1447.74    67    FQLATGQLENTAR    biorepCdiffA_1   
		    26    426.41    850.80    850.45    32    SELFSLR    biorepCdiffA_1   
   
      Matching Genes:  
               gi|115249085|emb|CAJ66896.1|  (50S ribosomal protein L29 [Clostridium difficile 630]) 
           
  Protein Group 74   
      Expression Quality:  
         Score      Num Spectra      Num Peptides      High-Qual Peptides      % Coverage       131    3    2    2    15   
   
      Peptides:   
        Query    Observed    Mr(expt)    Mr(calc)    Score    Peptide    Result File   
		    225    706.97    1411.93    1411.68    65    QNFGQVSNSYIR    biorepCdiffA_3   
		    355    889.08    1776.15    1775.81    66    TGEGDGDDEQIVVDLSK    biorepCdiffA_3   
   
      Matching Genes:  
               gi|115250675|emb|CAJ68499.1|  (tellurium resistance protein [Clostridium difficile 630]) 
           
  Protein Group 75   
      Expression Quality:  
         Score      Num Spectra      Num Peptides      High-Qual Peptides      % Coverage       128    5    3    3    17   
   
      Peptides:   
        Query    Observed    Mr(expt)    Mr(calc)    Score    Peptide    Result File   
		    291    775.62    1549.23    1548.81    46    ELSDIIGFTASQIR    biorepCdiffA_3   
		    493    793.13    1584.25    1583.83    42    GVWNFAPLDLEVPK    biorepCdiff_3   
		    151    482.86    963.70    963.50    40    YLGDLLDR    biorepCdiff_3   
   
      Matching Genes:  
               gi|115249180|emb|CAJ66992.1|  (putative DNA-binding protein [Clostridium difficile 630]) 
           
  Protein Group 76   
      Expression Quality:  
         Score      Num Spectra      Num Peptides      High-Qual Peptides      % Coverage       126    4    3    2    35   
   
      Peptides:   
        Query    Observed    Mr(expt)    Mr(calc)    Score    Peptide    Result File   
		    228    541.44    1080.87    1080.57    41    EIISYLESK    biorepCdiff_2   
		    771    1159.86    3476.55    3475.61    60    GIECVDYGTNNATDSVDYPVYGEIVANSVINK    biorepCdiff_2   
		    222    702.51    1403.00    1402.67    25    IGLGCDHGGYNLK    biorepCdiffA_2   
   
      Matching Genes:  
               gi|115252540|emb|CAJ70383.1|  (ribose-5-phosphate isomerase 2 [Clostridium difficile 630]) 
           
  Protein Group 77   
      Expression Quality:  
         Score      Num Spectra      Num Peptides      High-Qual Peptides      % Coverage       125    4    2    2    21   
   
      Peptides:   
        Query    Observed    Mr(expt)    Mr(calc)    Score    Peptide    Result File   
		    590    738.18    1474.35    1473.79    73    IGVIGGGSITYPNAR    biorepCdiff_1   
		    114    572.40    1142.79    1142.52    52    LSETDEFFR    biorepCdiffA_1   
   
      Matching Genes:  
               gi|115251040|emb|CAJ68871.1|  (putative decarboxylase [Clostridium difficile 630]) 
           
  Protein Group 78   
      Expression Quality:  
         Score      Num Spectra      Num Peptides      High-Qual Peptides      % Coverage       124    3    3    1    26   
   
      Peptides:   
        Query    Observed    Mr(expt)    Mr(calc)    Score    Peptide    Result File   
		    809    1179.86    3536.55    3535.76    39    LVMNLGFSHPVEMEDPEGITVEAPNQTELIVK    biorepCdiff_3   
		    85    508.38    1014.74    1014.57    50    QLSAELNIK    biorepCdiffA_3   
		    40    447.38    892.74    892.47    35    YVDEVIR    biorepCdiffA_3   
   
      Matching Genes:  
               gi|115249092|emb|CAJ66903.1|  (50S ribosomal protein L6 [Clostridium difficile 630]) 
           
  Protein Group 79   
      Expression Quality:  
         Score      Num Spectra      Num Peptides      High-Qual Peptides      % Coverage       124    3    3    1    5   
   
      Peptides:   
        Query    Observed    Mr(expt)    Mr(calc)    Score    Peptide    Result File   
		    511    1072.96    2143.91    2143.00    35    ADLVTGMVGEFDELQGFMGK    biorepCdiff_7   
		    30    430.95    859.89    859.48    50    FAGDILPK    biorepCdiff_7   
		    117    558.50    1114.98    1114.58    39    LADALFFYR    biorepCdiff_7   
   
      Matching Genes:  
               gi|115251485|emb|CAJ69318.1|  (glycyl-tRNA synthetase beta chain [Clostridium difficile 630]) 
           
  Protein Group 80   
      Expression Quality:  
         Score      Num Spectra      Num Peptides      High-Qual Peptides      % Coverage       121    4    2    2    13   
   
      Peptides:   
        Query    Observed    Mr(expt)    Mr(calc)    Score    Peptide    Result File   
		    225    706.97    1411.93    1411.68    65    QNFGQVSNSYIR    biorepCdiffA_3   
		    552    887.74    1773.47    1772.86    56    VEKDEDFIFYNNLK    biorepCdiff_3   
   
      Matching Genes:  
               gi|115250676|emb|CAJ68500.1|  (tellurium resistance protein [Clostridium difficile 630]) 
           
  Protein Group 81   
      Expression Quality:  
         Score      Num Spectra      Num Peptides      High-Qual Peptides      % Coverage       121    2    2    1    12   
   
      Peptides:   
        Query    Observed    Mr(expt)    Mr(calc)    Score    Peptide    Result File   
		    84    507.92    1013.82    1013.49    30    FWEEYLK    biorepCdiffA_3   
		    448    739.10    1476.19    1475.76    91    VEAGIYNEILNNK    biorepCdiff_3   
   
      Matching Genes:  
               gi|115252635|emb|CAJ70478.1|  (putative preprotein translocase [Clostridium difficile 630]) 
           
  Protein Group 82   
      Expression Quality:  
         Score      Num Spectra      Num Peptides      High-Qual Peptides      % Coverage       118    3    3    1    11   
   
      Peptides:   
        Query    Observed    Mr(expt)    Mr(calc)    Score    Peptide    Result File   
		    375    1069.32    2136.62    2136.05    36    EGVFAGGDAVTGAATVISAMGAGK    biorepCdiffA_6   
		    246    691.53    1381.05    1380.74    53    QAVVQPEIDNIR    biorepCdiff_6   
		    327    989.31    1976.60    1975.92    29    VCPQESQCEGVCILGIK    biorepCdiffA_6   
   
      Matching Genes:  
               gi|115250578|emb|CAJ68402.1|  (putative glutamate synthase [NADPH] small chain [Clostridium difficile 630]) 
           
  Protein Group 83   
      Expression Quality:  
         Score      Num Spectra      Num Peptides      High-Qual Peptides      % Coverage       115    3    3    1    4   
   
      Peptides:   
        Query    Observed    Mr(expt)    Mr(calc)    Score    Peptide    Result File   
		    45    497.84    993.67    994.47    34    ENMLWFR    biorepCdiffA_7   
		    621    1195.43    2388.84    2388.17    51    LAPSLTLGCGSWGGNSVSENVGVK    biorepCdiff_6   
		    99    511.54    1021.07    1020.57    30    LSPVLAMYK    biorepCdiff_7   
   
      Matching Genes:  
               gi|115249343|emb|CAJ67156.1|  (aldehyde-alcohol dehydrogenase [includes: alcohol dehydrogenase; acetaldehyde dehydrogenase [acetylating]; pyruvate-formate-lyase deactivase [Clostridium difficile 630]) 
           
  Protein Group 84   
      Expression Quality:  
         Score      Num Spectra      Num Peptides      High-Qual Peptides      % Coverage       112    6    3    1    8   
   
      Peptides:   
        Query    Observed    Mr(expt)    Mr(calc)    Score    Peptide    Result File   
		    217    669.43    1336.85    1336.61    52    DTDPQSALEYAK    biorepCdiff_6   
		    501    1037.84    2073.67    2073.00    30    VATYDLKPEMSAYELTDK    biorepCdiff_6   
		    81    583.46    1164.91    1164.60    30    VELAYNAMVR    biorepCdiffA_6   
   
      Matching Genes:  
               gi|115252228|emb|CAJ70068.1|  (2,3-bisphosphoglycerate-independent phosphoglycerate mutase [Clostridium difficile 630]) 
           
  Protein Group 85   
      Expression Quality:  
         Score      Num Spectra      Num Peptides      High-Qual Peptides      % Coverage       111    2    2    2    13   
   
      Peptides:   
        Query    Observed    Mr(expt)    Mr(calc)    Score    Peptide    Result File   
		    373    1003.39    2004.77    2003.92    54    DVYACCTHGVLSGPAIER    biorepCdiffA_4   
		    611    796.05    2385.12    2384.26    57    NVILLDDMIDTAGTIVNAANALK    biorepCdiff_4   
   
      Matching Genes:  
               gi|115252575|emb|CAJ70418.1|  (ribose-phosphate pyrophosphokinase [Clostridium difficile 630]) 
           
  Protein Group 86   
      Expression Quality:  
         Score      Num Spectra      Num Peptides      High-Qual Peptides      % Coverage       109    3    2    2    10   
   
      Peptides:   
        Query    Observed    Mr(expt)    Mr(calc)    Score    Peptide    Result File   
		    202    687.98    1373.95    1373.76    61    ALQATGLEVTMIK    biorepCdiffA_2   
		    427    696.06    1390.11    1389.75    48    ALQATGLEVTMIK +Oxidation (M)    biorepCdiff_2   
   
      Matching Genes:  
               gi|115249104|emb|CAJ66915.1|  (30S ribosomal protein S11 [Clostridium difficile 630]) 
           
  Protein Group 87   
      Expression Quality:  
         Score      Num Spectra      Num Peptides      High-Qual Peptides      % Coverage       107    3    2    1    11   
   
      Peptides:   
        Query    Observed    Mr(expt)    Mr(calc)    Score    Peptide    Result File   
		    426    869.15    1736.28    1735.76    69    ADIDYGFAEADTTYGK    biorepCdiff_4   
		    256    832.62    1663.23    1662.82    38    TEGYSEGNVPLQTLR    biorepCdiffA_4   
   
      Matching Genes:  
               gi|115249083|emb|CAJ66894.1|  (30S ribosomal protein S3 [Clostridium difficile 630]) 
           
  Protein Group 88   
      Expression Quality:  
         Score      Num Spectra      Num Peptides      High-Qual Peptides      % Coverage       107    2    2    2    8   
   
      Peptides:   
        Query    Observed    Mr(expt)    Mr(calc)    Score    Peptide    Result File   
		    214    628.04    1254.07    1253.75    46    LPIILYNVPGR    biorepCdiff_4   
		    366    761.21    1520.41    1519.78    61    TAMNILGFNVGDLR    biorepCdiff_4   
   
      Matching Genes:  
               gi|115252280|emb|CAJ70121.1|  (dihydrodipicolinate synthase [Clostridium difficile 630]) 
           
  Protein Group 89   
      Expression Quality:  
         Score      Num Spectra      Num Peptides      High-Qual Peptides      % Coverage       107    2    2    2    8   
   
      Peptides:   
        Query    Observed    Mr(expt)    Mr(calc)    Score    Peptide    Result File   
		    214    628.04    1254.07    1253.75    46    LPIILYNVPGR    biorepCdiff_4   
		    366    761.21    1520.41    1519.78    61    TAMNLLGFNVGDLR    biorepCdiff_4   
   
      Matching Genes:  
               gi|115252282|emb|CAJ70123.1|  (dihydrodipicolinate synthase [Clostridium difficile 630]) 
           
  Protein Group 90   
      Expression Quality:  
         Score      Num Spectra      Num Peptides      High-Qual Peptides      % Coverage       106    3    3    0    41   
   
      Peptides:   
        Query    Observed    Mr(expt)    Mr(calc)    Score    Peptide    Result File   
		    73    478.40    954.79    954.55    39    KYTFVVAK    biorepCdiffA_1   
		    500    908.01    2721.00    2720.31    31    MTNPHDVIIRPVVTEHSMAEMGEK    biorepCdiffA_1   
		    75    426.01    850.00    849.46    36    VFGVSVDK    biorepCdiff_1   
   
      Matching Genes:  
               gi|115249079|emb|CAJ66890.1|  (50S ribosomal protein L23 [Clostridium difficile 630]) 
           
  Protein Group 91   
      Expression Quality:  
         Score      Num Spectra      Num Peptides      High-Qual Peptides      % Coverage       106    3    3    1    5   
   
      Peptides:   
        Query    Observed    Mr(expt)    Mr(calc)    Score    Peptide    Result File   
		    168    620.98    1239.95    1239.65    44    VDAPEILDNVR    biorepCdiff_6   
		    30    460.36    918.70    918.45    30    YALDYFK    biorepCdiffA_6   
		    24    453.36    904.70    904.38    32    YAMDMFK    biorepCdiffA_6   
   
      Matching Genes:  
               gi|115249342|emb|CAJ67155.1|  (manganese-dependent inorganic pyrophosphatase [Clostridium difficile 630]) 
           
  Protein Group 92   
      Expression Quality:  
         Score      Num Spectra      Num Peptides      High-Qual Peptides      % Coverage       106    4    2    2    14   
   
      Peptides:   
        Query    Observed    Mr(expt)    Mr(calc)    Score    Peptide    Result File   
		    122    605.98    1209.95    1209.63    59    LSEVVEFYPK    biorepCdiffA_2   
		    175    665.00    1327.98    1327.70    47    NVPVNIWPYAR    biorepCdiffA_2   
   
      Matching Genes:  
               gi|115249997|emb|CAJ67817.1|  (hypothetical phage protein [Clostridium difficile 630]) 
              Other Genes Matching Peptide Subset:  
               gi|115251944|emb|CAJ69780.1|  (hypothetical phage protein [Clostridium difficile 630]) 
           
  Protein Group 93   
      Expression Quality:  
         Score      Num Spectra      Num Peptides      High-Qual Peptides      % Coverage       105    2    2    2    4   
   
      Peptides:   
        Query    Observed    Mr(expt)    Mr(calc)    Score    Peptide    Result File   
		    108    518.37    1034.73    1034.55    46    QALSYALNR    biorepCdiff_6   
		    240    846.56    1691.11    1690.74    59    SNSNLQTYTYSEER    biorepCdiffA_6   
   
      Matching Genes:  
               gi|115251723|emb|CAJ69558.1|  (oligopeptide ABC transporter, substrate-binding protein [Clostridium difficile 630]) 
           
  Protein Group 94   
      Expression Quality:  
         Score      Num Spectra      Num Peptides      High-Qual Peptides      % Coverage       102    3    2    1    9   
   
      Peptides:   
        Query    Observed    Mr(expt)    Mr(calc)    Score    Peptide    Result File   
		    429    1035.28    2068.55    2067.97    39    GGPGLGSIQPSQADYFMSTR    biorepCdiffA_5   
		    124    632.50    1262.98    1262.65    63    VMTSSSSPGVALK    biorepCdiffA_5   
   
      Matching Genes:  
               gi|115249125|emb|CAJ66936.1|  (putative oxidoreductase, thiamine diP-binding subunit [Clostridium difficile 630]) 
           
  Protein Group 95   
      Expression Quality:  
         Score      Num Spectra      Num Peptides      High-Qual Peptides      % Coverage       102    5    1    1    10   
   
      Peptides:   
        Query    Observed    Mr(expt)    Mr(calc)    Score    Peptide    Result File   
		    361    935.32    1868.62    1868.91    102    LAGEGGLFFVDQEFANR    biorepCdiffA_1   
   
      Matching Genes:  
               gi|115251248|emb|CAJ69079.1|  (ferritin [Clostridium difficile 630]) 
           
  Protein Group 96   
      Expression Quality:  
         Score      Num Spectra      Num Peptides      High-Qual Peptides      % Coverage       102    4    3    1    6   
   
      Peptides:   
        Query    Observed    Mr(expt)    Mr(calc)    Score    Peptide    Result File   
		    478    1042.75    2083.49    2082.92    40    GTYGDTYGIHGNNNESSIGK    biorepCdiff_7   
		    241    700.53    1399.05    1399.73    33    IAEELGNREEIK    biorepCdiff_7   
		    60    537.38    1072.75    1072.57    29    INLNQTGWK    biorepCdiffA_7   
   
      Matching Genes:  
               gi|115251764|emb|CAJ69599.1|  (cell surface protein [Clostridium difficile 630]) 
           
  Protein Group 97   
      Expression Quality:  
         Score      Num Spectra      Num Peptides      High-Qual Peptides      % Coverage       102    3    2    1    32   
   
      Peptides:   
        Query    Observed    Mr(expt)    Mr(calc)    Score    Peptide    Result File   
		    735    1027.39    2052.77    2052.02    38    NYELVYVVKPNSDEEVR    biorepCdiff_1   
		    198    694.02    1386.03    1385.78    64    VKEVVATDGEIVK    biorepCdiffA_1   
   
      Matching Genes:  
               gi|115252728|emb|CAJ70572.1|  (30S ribosomal protein S6 [Clostridium difficile 630]) 
           
  Protein Group 98   
      Expression Quality:  
         Score      Num Spectra      Num Peptides      High-Qual Peptides      % Coverage       98    3    3    0    11   
   
      Peptides:   
        Query    Observed    Mr(expt)    Mr(calc)    Score    Peptide    Result File   
		    105    535.50    1068.99    1068.59    26    FTAEVYVLK    biorepCdiff_5   
		    353    590.32    1767.94    1767.78    34    HYAHVDCPGHADYVK    biorepCdiffA_3   
		    468    1083.35    2164.68    2164.04    38    YQLGEAVDFANIDKAPEER    biorepCdiffA_5   
   
      Matching Genes:  
               gi|115249061|emb|CAJ66872.1|  (elongation factor TU [Clostridium difficile 630]) 
              Other Genes Matching Peptide Subset:  
               gi|115249075|emb|CAJ66886.1|  (elongation factor TU [Clostridium difficile 630]) 
           
  Protein Group 99   
      Expression Quality:  
         Score      Num Spectra      Num Peptides      High-Qual Peptides      % Coverage       98    2    2    1    25   
   
      Peptides:   
        Query    Observed    Mr(expt)    Mr(calc)    Score    Peptide    Result File   
		    510    1031.34    3091.01    3091.55    62    KGEFFCSEAVLQTINDALGQPLSPEITK    biorepCdiffA_2   
		    98    530.46    1058.91    1058.61    36    LASGFPIGLGK    biorepCdiffA_2   
   
      Matching Genes:  
               gi|115252075|emb|CAJ69912.1|  (conserved hypothetical protein [Clostridium difficile 630]) 
           
  Protein Group 100   
      Expression Quality:  
         Score      Num Spectra      Num Peptides      High-Qual Peptides      % Coverage       97    3    2    2    5   
   
      Peptides:   
        Query    Observed    Mr(expt)    Mr(calc)    Score    Peptide    Result File   
		    43    465.38    928.74    928.56    44    IGIEGSILK    biorepCdiffA_5   
		    199    646.11    1290.21    1289.70    53    ILVLNCGSSSLK    biorepCdiff_5   
   
      Matching Genes:  
               gi|115250207|emb|CAJ68028.1|  (acetate kinase [Clostridium difficile 630]) 
           
  Protein Group 101   
      Expression Quality:  
         Score      Num Spectra      Num Peptides      High-Qual Peptides      % Coverage       97    4    2    2    19   
   
      Peptides:   
        Query    Observed    Mr(expt)    Mr(calc)    Score    Peptide    Result File   
		    242    566.42    1130.83    1130.67    50    IQVFEGVVLK    biorepCdiff_2   
		    402    687.44    1372.86    1372.66    47    NEVPNFGPGDTVK    biorepCdiff_2   
   
      Matching Genes:  
               gi|115250291|emb|CAJ68113.1|  (50S ribosomal protein L19 [Clostridium difficile 630]) 
           
  Protein Group 102   
      Expression Quality:  
         Score      Num Spectra      Num Peptides      High-Qual Peptides      % Coverage       97    3    3    0    5   
   
      Peptides:   
        Query    Observed    Mr(expt)    Mr(calc)    Score    Peptide    Result File   
		    127    572.97    1143.93    1143.57    32    APESLIEEEK    biorepCdiff_7   
		    198    668.58    1335.15    1334.70    33    TYLQWLENIR    biorepCdiff_7   
		    491    1338.56    2675.11    2675.09    32    YNEEDNMAEEEDMMNLLMEGIR    biorepCdiffA_7   
   
      Matching Genes:  
               gi|115252312|emb|CAJ70153.1|  (valyl-tRNA synthetase [Clostridium difficile 630]) 
           
  Protein Group 103   
      Expression Quality:  
         Score      Num Spectra      Num Peptides      High-Qual Peptides      % Coverage       94    6    2    1    19   
   
      Peptides:   
        Query    Observed    Mr(expt)    Mr(calc)    Score    Peptide    Result File   
		    215    518.44    1034.86    1034.54    34    GITEVVFDR    biorepCdiff_2   
		    570    574.82    1721.42    1720.87    60    SANNIYAQIIDDTKR    biorepCdiff_2   
   
      Matching Genes:  
               gi|115249093|emb|CAJ66904.1|  (50S ribosomal protein L18 [Clostridium difficile 630]) 
           
  Protein Group 104   
      Expression Quality:  
         Score      Num Spectra      Num Peptides      High-Qual Peptides      % Coverage       94    3    1    1    25   
   
      Peptides:   
        Query    Observed    Mr(expt)    Mr(calc)    Score    Peptide    Result File   
		    351    919.23    1836.44    1835.88    94    TLEEGQSVEFEVVDGAK    biorepCdiffA_1   
   
      Matching Genes:  
               gi|115250391|emb|CAJ68213.1|  (putative cold shock protein [Clostridium difficile 630]) 
           
  Protein Group 105   
      Expression Quality:  
         Score      Num Spectra      Num Peptides      High-Qual Peptides      % Coverage       93    3    2    1    22   
   
      Peptides:   
        Query    Observed    Mr(expt)    Mr(calc)    Score    Peptide    Result File   
		    139    651.97    1301.93    1301.68    32    EVEASVGGGAVTVK    biorepCdiff_8   
		    186    675.39    1348.77    1348.63    61    NIDDIQASQMSK    biorepCdiffA_1   
   
      Matching Genes:  
               gi|115249020|emb|CAJ66831.1|  (conserved hypothetical protein [Clostridium difficile 630]) 
           
  Protein Group 106   
      Expression Quality:  
         Score      Num Spectra      Num Peptides      High-Qual Peptides      % Coverage       92    5    2    1    16   
   
      Peptides:   
        Query    Observed    Mr(expt)    Mr(calc)    Score    Peptide    Result File   
		    114    583.48    1164.94    1164.60    34    EQALVEVSYK    biorepCdiffA_2   
		    480    730.57    1459.12    1458.78    58    ILGGGLPYESAVQR    biorepCdiff_2   
   
      Matching Genes:  
               gi|115249807|emb|CAJ67624.1|  (putative NUDIX-family hydrolase [Clostridium difficile 630]) 
           
  Protein Group 107   
      Expression Quality:  
         Score      Num Spectra      Num Peptides      High-Qual Peptides      % Coverage       88    2    2    1    5   
   
      Peptides:   
        Query    Observed    Mr(expt)    Mr(calc)    Score    Peptide    Result File   
		    227    676.56    1351.11    1350.79    54    IVEVPVGEALIGR    biorepCdiff_6   
		    205    770.52    1539.02    1538.81    34    TRPVESEAPGIIDR    biorepCdiffA_6   
   
      Matching Genes:  
               gi|115252530|emb|CAJ70373.1|  (ATP synthase alpha chain [Clostridium difficile 630]) 
           
  Protein Group 108   
      Expression Quality:  
         Score      Num Spectra      Num Peptides      High-Qual Peptides      % Coverage       87    3    2    2    7   
   
      Peptides:   
        Query    Observed    Mr(expt)    Mr(calc)    Score    Peptide    Result File   
		    50    522.93    1043.85    1043.55    43    ALEAAEELAK    biorepCdiffA_4   
		    231    638.52    1275.02    1274.65    44    HYGLTTEDIVK    biorepCdiff_4   
   
      Matching Genes:  
               gi|115251376|emb|CAJ69208.1|  (transketolase [Clostridium difficile 630]) 
           
  Protein Group 109   
      Expression Quality:  
         Score      Num Spectra      Num Peptides      High-Qual Peptides      % Coverage       87    2    2    1    13   
   
      Peptides:   
        Query    Observed    Mr(expt)    Mr(calc)    Score    Peptide    Result File   
		    66    484.89    967.76    967.57    39    ILLDEPIR    biorepCdiffA_3   
		    220    566.65    1131.29    1131.60    48    MAVQADSVAIK    biorepCdiff_3   
   
      Matching Genes:  
               gi|115252722|emb|CAJ70566.1|  (50S ribosomal protein L9 [Clostridium difficile 630]) 
           
  Protein Group 110   
      Expression Quality:  
         Score      Num Spectra      Num Peptides      High-Qual Peptides      % Coverage       82    3    2    1    26   
   
      Peptides:   
        Query    Observed    Mr(expt)    Mr(calc)    Score    Peptide    Result File   
		    322    584.39    1750.16    1749.89    35    HQKPSAMNQQGGIINK    biorepCdiffA_1   
		    119    586.00    1169.98    1169.70    47    VLVEGVNVITK    biorepCdiffA_1   
   
      Matching Genes:  
               gi|115249088|emb|CAJ66899.1|  (50S ribosomal protein L24 [Clostridium difficile 630]) 
           
  Protein Group 111   
      Expression Quality:  
         Score      Num Spectra      Num Peptides      High-Qual Peptides      % Coverage       81    2    2    2    4   
   
      Peptides:   
        Query    Observed    Mr(expt)    Mr(calc)    Score    Peptide    Result File   
		    335    807.63    1613.24    1612.76    40    FFNEEEMNAILTR    biorepCdiff_6   
		    142    593.72    1185.42    1184.64    41    VGQEGVTSPIAK    biorepCdiff_6   
   
      Matching Genes:  
               gi|115251791|emb|CAJ69626.1|  (putative aspartyl-tRNA synthetase [Clostridium difficile 630]) 
           
  Protein Group 112   
      Expression Quality:  
         Score      Num Spectra      Num Peptides      High-Qual Peptides      % Coverage       81    2    2    1    4   
   
      Peptides:   
        Query    Observed    Mr(expt)    Mr(calc)    Score    Peptide    Result File   
		    111    621.48    1240.95    1240.61    28    AFDYVTQEIR    biorepCdiffA_6   
		    314    809.11    1616.21    1615.81    53    IVLEENEQSLPMSK    biorepCdiffA_3   
   
      Matching Genes:  
               gi|115252300|emb|CAJ70141.1|  (proline reductase subunit proprotein [Clostridium difficile 630]) 
           
  Protein Group 113   
      Expression Quality:  
         Score      Num Spectra      Num Peptides      High-Qual Peptides      % Coverage       79    2    2    1    6   
   
      Peptides:   
        Query    Observed    Mr(expt)    Mr(calc)    Score    Peptide    Result File   
		    59    501.97    1001.93    1001.55    51    FVIEPLER    biorepCdiffA_5   
		    167    669.64    1337.27    1336.65    28    VDIVELSEDYR    biorepCdiffA_5   
   
      Matching Genes:  
               gi|115249106|emb|CAJ66917.1|  (DNA-directed RNA polymerase alpha chain [Clostridium difficile 630]) 
           
  Protein Group 114   
      Expression Quality:  
         Score      Num Spectra      Num Peptides      High-Qual Peptides      % Coverage       78    3    2    1    16   
   
      Peptides:   
        Query    Observed    Mr(expt)    Mr(calc)    Score    Peptide    Result File   
		    53    460.47    918.92    918.52    40    FEILEIR    biorepCdiffA_3   
		    456    696.24    2085.69    2084.99    38    VAISFGDLSENAEYDEAKK    biorepCdiffA_3   
   
      Matching Genes:  
               gi|115252616|emb|CAJ70459.1|  (transcription elongation factor [Clostridium difficile 630]) 
           
  Protein Group 115   
      Expression Quality:  
         Score      Num Spectra      Num Peptides      High-Qual Peptides      % Coverage       77    2    2    1    4   
   
      Peptides:   
        Query    Observed    Mr(expt)    Mr(calc)    Score    Peptide    Result File   
		    90    592.94    1183.86    1183.61    29    LDIYQEYIK    biorepCdiffA_6   
		    159    687.55    1373.08    1372.72    48    NLSKEEVEANIK    biorepCdiffA_6   
   
      Matching Genes:  
               gi|115249054|emb|CAJ66865.1|  (glutamyl-tRNA synthetase [Clostridium difficile 630]) 
           
  Protein Group 116   
      Expression Quality:  
         Score      Num Spectra      Num Peptides      High-Qual Peptides      % Coverage       76    2    1    1    3   
   
      Peptides:   
        Query    Observed    Mr(expt)    Mr(calc)    Score    Peptide    Result File   
		    379    869.60    1737.19    1736.85    76    SIVVEGESDSSYPLQK    biorepCdiff_6   
   
      Matching Genes:  
               gi|115251299|emb|CAJ69130.1|  (asparaginyl-tRNA synthetase [Clostridium difficile 630]) 
           
  Protein Group 117   
      Expression Quality:  
         Score      Num Spectra      Num Peptides      High-Qual Peptides      % Coverage       76    2    1    1    3   
   
      Peptides:   
        Query    Observed    Mr(expt)    Mr(calc)    Score    Peptide    Result File   
		    248    694.43    1386.84    1386.70    76    KLEEGADVDLGNK    biorepCdiff_6   
   
      Matching Genes:  
               gi|115251396|emb|CAJ69228.1|  (gamma-aminobutyrate metabolism dehydratase/isomerase [includes: 4-hydroxybutyryl-coa dehydratase; vinylacetyl-coa-delta-isomerase] [Clostridium difficile 630]) 
           
  Protein Group 118   
      Expression Quality:  
         Score      Num Spectra      Num Peptides      High-Qual Peptides      % Coverage       76    4    2    1    13   
   
      Peptides:   
        Query    Observed    Mr(expt)    Mr(calc)    Score    Peptide    Result File   
		    348    864.64    1727.26    1726.88    50    EGITSVGENKPQELAR    biorepCdiffA_3   
		    590    934.30    1866.58    1865.85    26    GLMTMAPFIEDEDEIR    biorepCdiff_3   
   
      Matching Genes:  
               gi|115251674|emb|CAJ69509.1|  (putative alanine racemase [Clostridium difficile 630]) 
           
  Protein Group 119   
      Expression Quality:  
         Score      Num Spectra      Num Peptides      High-Qual Peptides      % Coverage       73    3    2    1    29   
   
      Peptides:   
        Query    Observed    Mr(expt)    Mr(calc)    Score    Peptide    Result File   
		    347    914.46    1826.90    1826.94    41    AEGDTGSPEVQIALLTAR    biorepCdiffA_1   
		    17    417.93    833.84    833.50    32    NLLAYLK    biorepCdiffA_1   
   
      Matching Genes:  
               gi|115250352|emb|CAJ68174.1|  (30S ribosomal protein S15 [Clostridium difficile 630]) 
           
  Protein Group 120   
      Expression Quality:  
         Score      Num Spectra      Num Peptides      High-Qual Peptides      % Coverage       70    2    1    1    9   
   
      Peptides:   
        Query    Observed    Mr(expt)    Mr(calc)    Score    Peptide    Result File   
		    351    881.72    1761.43    1760.81    70    TGVGDGDDEQINVDLSK    biorepCdiffA_3   
   
      Matching Genes:  
               gi|115250845|emb|CAJ68669.1|  (tellurium resistance protein [Clostridium difficile 630]) 
           
  Protein Group 121   
      Expression Quality:  
         Score      Num Spectra      Num Peptides      High-Qual Peptides      % Coverage       70    2    1    1    5   
   
      Peptides:   
        Query    Observed    Mr(expt)    Mr(calc)    Score    Peptide    Result File   
		    329    696.69    1391.37    1390.68    70    MEEEGVFNVLPK    biorepCdiff_4   
   
      Matching Genes:  
               gi|115251194|emb|CAJ69025.1|  (30S ribosomal protein S2 [Clostridium difficile 630]) 
           
  Protein Group 122   
      Expression Quality:  
         Score      Num Spectra      Num Peptides      High-Qual Peptides      % Coverage       69    3    2    1    7   
   
      Peptides:   
        Query    Observed    Mr(expt)    Mr(calc)    Score    Peptide    Result File   
		    161    661.49    1320.97    1320.66    29    AVQLHGGYGYTR    biorepCdiffA_5   
		    355    763.10    1524.18    1523.75    40    ITEIYEGTSEVQR    biorepCdiff_5   
   
      Matching Genes:  
               gi|115250075|emb|CAJ67895.1|  (butyryl-CoA dehydrogenase [Clostridium difficile 630]) 
           
  Protein Group 123   
      Expression Quality:  
         Score      Num Spectra      Num Peptides      High-Qual Peptides      % Coverage       68    1    1    1    18   
   
      Peptides:   
        Query    Observed    Mr(expt)    Mr(calc)    Score    Peptide    Result File   
		    630    688.96    2063.86    2063.24    68    IATAGVVLGAVTGAVSGVLLAPK    biorepCdiff_2   
   
      Matching Genes:  
               gi|115250942|emb|CAJ68770.1|  (putative membrane protein [Clostridium difficile 630]) 
           
  Protein Group 124   
      Expression Quality:  
         Score      Num Spectra      Num Peptides      High-Qual Peptides      % Coverage       68    1    1    1    6   
   
      Peptides:   
        Query    Observed    Mr(expt)    Mr(calc)    Score    Peptide    Result File   
		    422    712.53    1423.05    1422.62    68    TTGICIDCSSPGR    biorepCdiff_3   
   
      Matching Genes:  
               gi|115251115|emb|CAJ68946.1|  (conserved hypothetical protein [Clostridium difficile 630]) 
           
  Protein Group 125   
      Expression Quality:  
         Score      Num Spectra      Num Peptides      High-Qual Peptides      % Coverage       68    2    1    1    2   
   
      Peptides:   
        Query    Observed    Mr(expt)    Mr(calc)    Score    Peptide    Result File   
		    303    772.00    1541.99    1541.66    68    YVDSNNENEPFSK    biorepCdiff_7   
   
      Matching Genes:  
               gi|115251575|emb|CAJ69408.1|  (leucyl-tRNA synthetase [Clostridium difficile 630]) 
           
  Protein Group 126   
      Expression Quality:  
         Score      Num Spectra      Num Peptides      High-Qual Peptides      % Coverage       66    2    2    0    5   
   
      Peptides:   
        Query    Observed    Mr(expt)    Mr(calc)    Score    Peptide    Result File   
		    212    534.76    1601.26    1600.80    30    DLEEYLHLLEEAK    biorepCdiffA_7   
		    544    1125.33    2248.64    2248.13    36    ELGLFMIPEEGPGFPMFLPK    biorepCdiff_7   
   
      Matching Genes:  
               gi|115249589|emb|CAJ67406.1|  (threonyl-tRNA synthetase [Clostridium difficile 630]) 
           
  Protein Group 127   
      Expression Quality:  
         Score      Num Spectra      Num Peptides      High-Qual Peptides      % Coverage       65    2    2    0    15   
   
      Peptides:   
        Query    Observed    Mr(expt)    Mr(calc)    Score    Peptide    Result File   
		    288    813.19    1624.36    1623.81    31    HVPVYITEDMVGHK    biorepCdiffA_1   
		    291    821.10    1640.19    1639.80    34    HVPVYITEDMVGHK +Oxidation (M)    biorepCdiffA_1   
   
      Matching Genes:  
               gi|115249081|emb|CAJ66892.1|  (30S ribosomal protein S19 [Clostridium difficile 630]) 
           
  Protein Group 128   
      Expression Quality:  
         Score      Num Spectra      Num Peptides      High-Qual Peptides      % Coverage       64    2    2    0    15   
   
      Peptides:   
        Query    Observed    Mr(expt)    Mr(calc)    Score    Peptide    Result File   
		    243    736.62    1471.23    1470.80    38    IYSSLYLEDLKK    biorepCdiffA_2   
		    159    648.45    1294.89    1294.63    26    YLIDITEEGDK    biorepCdiffA_2   
   
      Matching Genes:  
               gi|115250371|emb|CAJ68193.1|  (MarR-family transcriptional regulator [Clostridium difficile 630]) 
           
  Protein Group 129   
      Expression Quality:  
         Score      Num Spectra      Num Peptides      High-Qual Peptides      % Coverage       63    2    2    0    3   
   
      Peptides:   
        Query    Observed    Mr(expt)    Mr(calc)    Score    Peptide    Result File   
		    407    625.84    1874.51    1873.98    29    DGSLVGAILINDLSCTVK    biorepCdiff_7   
		    140    607.46    1212.90    1212.65    34    VNLFDVIEHK    biorepCdiff_7   
   
      Matching Genes:  
               gi|115250664|emb|CAJ68488.1|  (putative nitric oxide reductase flavoprotein [Clostridium difficile 630]) 
           
  Protein Group 130   
      Expression Quality:  
         Score      Num Spectra      Num Peptides      High-Qual Peptides      % Coverage       63    1    1    1    2   
   
      Peptides:   
        Query    Observed    Mr(expt)    Mr(calc)    Score    Peptide    Result File   
		    183    749.05    1496.09    1495.69    63    VYGEGADSQGVSATR    biorepCdiffA_7   
   
      Matching Genes:  
               gi|115251074|emb|CAJ68905.1|  (chaperone [Clostridium difficile 630]) 
           
  Protein Group 131   
      Expression Quality:  
         Score      Num Spectra      Num Peptides      High-Qual Peptides      % Coverage       63    1    1    1    9   
   
      Peptides:   
        Query    Observed    Mr(expt)    Mr(calc)    Score    Peptide    Result File   
		    230    715.99    1429.96    1429.74    63    VTVDENTIGQINK    biorepCdiffA_2   
   
      Matching Genes:  
               gi|115251795|emb|CAJ69630.1|  (putative D-tyrosyl-tRNA protein [Clostridium difficile 630]) 
           
  Protein Group 132   
      Expression Quality:  
         Score      Num Spectra      Num Peptides      High-Qual Peptides      % Coverage       62    4    2    0    18   
   
      Peptides:   
        Query    Observed    Mr(expt)    Mr(calc)    Score    Peptide    Result File   
		    538    697.60    1393.20    1392.70    31    KGDAAEMAFIELV    biorepCdiff_1   
		    275    494.94    987.87    987.55    31    NLVTCLLR    biorepCdiff_1   
   
      Matching Genes:  
               gi|115249107|emb|CAJ66918.1|  (50S ribosomal protein L17 [Clostridium difficile 630]) 
           
  Protein Group 133   
      Expression Quality:  
         Score      Num Spectra      Num Peptides      High-Qual Peptides      % Coverage       62    1    1    1    15   
   
      Peptides:   
        Query    Observed    Mr(expt)    Mr(calc)    Score    Peptide    Result File   
		    304    838.83    1675.64    1675.83    62    VETQGATGIDNELTTK    biorepCdiffA_1   
   
      Matching Genes:  
               gi|115252071|emb|CAJ69908.1|  (PTS system, IIb component [Clostridium difficile 630]) 
           
  Protein Group 134   
      Expression Quality:  
         Score      Num Spectra      Num Peptides      High-Qual Peptides      % Coverage       61    1    1    1    12   
   
      Peptides:   
        Query    Observed    Mr(expt)    Mr(calc)    Score    Peptide    Result File   
		    318    861.73    1721.45    1720.82    61    GYDVSSISTPDLENPK    biorepCdiffA_1   
   
      Matching Genes:  
               gi|115249013|emb|CAJ66824.1|  (anti-sigma-B factor (serine-protein kinase) [Clostridium difficile 630]) 
           
  Protein Group 135   
      Expression Quality:  
         Score      Num Spectra      Num Peptides      High-Qual Peptides      % Coverage       60    1    1    1    6   
   
      Peptides:   
        Query    Observed    Mr(expt)    Mr(calc)    Score    Peptide    Result File   
		    689    1029.57    3085.68    3085.44    60    EFYPDASEFYLSDGYDLVNALTGSTIAK    biorepCdiff_4   
   
      Matching Genes:  
               gi|115251246|emb|CAJ69077.1|  (cell surface protein [Clostridium difficile 630]) 
           
  Protein Group 136   
      Expression Quality:  
         Score      Num Spectra      Num Peptides      High-Qual Peptides      % Coverage       60    1    1    1    13   
   
      Peptides:   
        Query    Observed    Mr(expt)    Mr(calc)    Score    Peptide    Result File   
		    753    881.73    2642.16    2641.24    60    LPGEVESYEYGLEYGTDTLEIHK    biorepCdiff_3   
   
      Matching Genes:  
               gi|115251797|emb|CAJ69632.1|  (adenine phosphoribosyltransferase [Clostridium difficile 630]) 
           
  Protein Group 137   
      Expression Quality:  
         Score      Num Spectra      Num Peptides      High-Qual Peptides      % Coverage       58    1    1    1    4   
   
      Peptides:   
        Query    Observed    Mr(expt)    Mr(calc)    Score    Peptide    Result File   
		    611    1182.90    2363.78    2364.29    58    EILGDIPISLSNEIGNIGILER    biorepCdiff_6   
   
      Matching Genes:  
               gi|115250762|emb|CAJ68586.1|  (putative hydantoinase [Clostridium difficile 630]) 
           
  Protein Group 138   
      Expression Quality:  
         Score      Num Spectra      Num Peptides      High-Qual Peptides      % Coverage       58    2    1    1    3   
   
      Peptides:   
        Query    Observed    Mr(expt)    Mr(calc)    Score    Peptide    Result File   
		    195    644.44    1286.87    1286.63    58    ADAYGHGAVEVAK    biorepCdiff_5   
   
      Matching Genes:  
               gi|115252523|emb|CAJ70366.1|  (alanine racemase [Clostridium difficile 630]) 
           
  Protein Group 139   
      Expression Quality:  
         Score      Num Spectra      Num Peptides      High-Qual Peptides      % Coverage       57    2    1    1    16   
   
      Peptides:   
        Query    Observed    Mr(expt)    Mr(calc)    Score    Peptide    Result File   
		    671    900.41    1798.80    1797.88    57    FIEEIGYYNPISEPK    biorepCdiff_1   
   
      Matching Genes:  
               gi|115250287|emb|CAJ68109.1|  (30S ribosomal protein S16 [Clostridium difficile 630]) 
           
  Protein Group 140   
      Expression Quality:  
         Score      Num Spectra      Num Peptides      High-Qual Peptides      % Coverage       56    1    1    1    13   
   
      Peptides:   
        Query    Observed    Mr(expt)    Mr(calc)    Score    Peptide    Result File   
		    145    631.68    1261.34    1260.71    56    TIVVAVEEFVR    biorepCdiffA_1   
   
      Matching Genes:  
               gi|115249086|emb|CAJ66897.1|  (30S ribosomal protein S17 [Clostridium difficile 630]) 
           
  Protein Group 141   
      Expression Quality:  
         Score      Num Spectra      Num Peptides      High-Qual Peptides      % Coverage       56    1    1    1    0   
   
      Peptides:   
        Query    Observed    Mr(expt)    Mr(calc)    Score    Peptide    Result File   
		    256    773.51    1545.01    1544.67    56    NIAYNYTDESNNK    biorepCdiff_8   
   
      Matching Genes:  
               gi|115249677|emb|CAJ67494.1|  (toxin A [Clostridium difficile 630]) 
           
  Protein Group 142   
      Expression Quality:  
         Score      Num Spectra      Num Peptides      High-Qual Peptides      % Coverage       56    1    1    1    6   
   
      Peptides:   
        Query    Observed    Mr(expt)    Mr(calc)    Score    Peptide    Result File   
		    400    815.78    1629.55    1628.88    56    FGDGGVDILPIANLTK    biorepCdiff_4   
   
      Matching Genes:  
               gi|115249811|emb|CAJ67628.1|  (NH3-dependent NAD(+) synthetase [Clostridium difficile 630]) 
           
  Protein Group 143   
      Expression Quality:  
         Score      Num Spectra      Num Peptides      High-Qual Peptides      % Coverage       56    1    1    1    6   
   
      Peptides:   
        Query    Observed    Mr(expt)    Mr(calc)    Score    Peptide    Result File   
		    176    683.55    1365.09    1364.70    56    IEELQFNPFTK    biorepCdiffA_4   
   
      Matching Genes:  
               gi|115251448|emb|CAJ69281.1|  (conserved hypothetical protein [Clostridium difficile 630]) 
           
  Protein Group 144   
      Expression Quality:  
         Score      Num Spectra      Num Peptides      High-Qual Peptides      % Coverage       55    1    1    1    8   
   
      Peptides:   
        Query    Observed    Mr(expt)    Mr(calc)    Score    Peptide    Result File   
		    339    663.01    1324.00    1323.64    55    VMFELAGVSEDK    biorepCdiff_3   
   
      Matching Genes:  
               gi|115249084|emb|CAJ66895.1|  (50S ribosomal protein L16 [Clostridium difficile 630]) 
           
  Protein Group 145   
      Expression Quality:  
         Score      Num Spectra      Num Peptides      High-Qual Peptides      % Coverage       54    2    2    0    6   
   
      Peptides:   
        Query    Observed    Mr(expt)    Mr(calc)    Score    Peptide    Result File   
		    153    454.04    1359.10    1358.68    29    AFEGTLLAHTDGK    biorepCdiffA_6   
		    378    816.74    1631.46    1630.87    25    TGLGNTFLGWIDLPK    biorepCdiff_5   
   
      Matching Genes:  
               gi|115252341|emb|CAJ70182.1|  (glucose-6-phosphate isomerase [Clostridium difficile 630]) 
           
  Protein Group 146   
      Expression Quality:  
         Score      Num Spectra      Num Peptides      High-Qual Peptides      % Coverage       54    1    1    1    22   
   
      Peptides:   
        Query    Observed    Mr(expt)    Mr(calc)    Score    Peptide    Result File   
		    147    636.60    1271.18    1270.70    54    LIDEALEDLIK    biorepCdiffA_1   
   
      Matching Genes:  
               gi|110666931|ref|YP_659585.1|  (hypothetical protein CDP09 [Clostridium difficile 630]) 
           
  Protein Group 147   
      Expression Quality:  
         Score      Num Spectra      Num Peptides      High-Qual Peptides      % Coverage       53    1    1    1    4   
   
      Peptides:   
        Query    Observed    Mr(expt)    Mr(calc)    Score    Peptide    Result File   
		    435    763.28    2286.82    2286.04    53    NSLTNKPTWSPMGSSANHEGR +Oxidation (M)    biorepCdiffA_6   
   
      Matching Genes:  
               gi|115250843|emb|CAJ68667.1|  (putative pyridine nucleotide-disulfide oxidoreductase [Clostridium difficile 630]) 
           
  Protein Group 148   
      Expression Quality:  
         Score      Num Spectra      Num Peptides      High-Qual Peptides      % Coverage       51    2    1    1    2   
   
      Peptides:   
        Query    Observed    Mr(expt)    Mr(calc)    Score    Peptide    Result File   
		    57    453.03    904.04    903.51    51    YDLVPGLK    biorepCdiff_5   
   
      Matching Genes:  
               gi|115249115|emb|CAJ66926.1|  (aspartate aminotransferase [Clostridium difficile 630]) 
           
  Protein Group 149   
      Expression Quality:  
         Score      Num Spectra      Num Peptides      High-Qual Peptides      % Coverage       48    1    1    1    6   
   
      Peptides:   
        Query    Observed    Mr(expt)    Mr(calc)    Score    Peptide    Result File   
		    522    844.23    1686.44    1685.83    48    VNVYYDNPLEVAYK    biorepCdiff_3   
   
      Matching Genes:  
               gi|115250593|emb|CAJ68417.1|  (putative 1-(5-phosphoribosyl)-5-[(5-phosphoribosylamino)methylidene amino] imidazole-4-carboxamide isomerase [Clostridium difficile 630]) 
           
  Protein Group 150   
      Expression Quality:  
         Score      Num Spectra      Num Peptides      High-Qual Peptides      % Coverage       48    1    1    1    10   
   
      Peptides:   
        Query    Observed    Mr(expt)    Mr(calc)    Score    Peptide    Result File   
		    419    635.57    1269.13    1268.60    48    IGIDEDHSVER    biorepCdiff_1   
   
      Matching Genes:  
               gi|115252725|emb|CAJ70569.1|  (conserved hypothetical protein [Clostridium difficile 630]) 
           
  Protein Group 151   
      Expression Quality:  
         Score      Num Spectra      Num Peptides      High-Qual Peptides      % Coverage       47    2    1    1    6   
   
      Peptides:   
        Query    Observed    Mr(expt)    Mr(calc)    Score    Peptide    Result File   
		    39    497.77    993.53    993.55    47    YPFLLVDK    biorepCdiffA_8   
   
      Matching Genes:  
               gi|115249137|emb|CAJ66948.1|  ((3R)-hydroxymyristoyl-[acyl carrier protein] dehydratase [Clostridium difficile 630]) 
           
  Protein Group 152   
      Expression Quality:  
         Score      Num Spectra      Num Peptides      High-Qual Peptides      % Coverage       47    2    1    1    2   
   
      Peptides:   
        Query    Observed    Mr(expt)    Mr(calc)    Score    Peptide    Result File   
		    330    800.57    1599.13    1598.76    47    TYVSAYHSTNLSEK    biorepCdiff_6   
   
      Matching Genes:  
               gi|115251837|emb|CAJ69672.1|  (cell surface protein [Clostridium difficile 630]) 
           
  Protein Group 153   
      Expression Quality:  
         Score      Num Spectra      Num Peptides      High-Qual Peptides      % Coverage       46    2    1    1    5   
   
      Peptides:   
        Query    Observed    Mr(expt)    Mr(calc)    Score    Peptide    Result File   
		    76    493.90    985.79    985.62    46    IIVNSSLIK    biorepCdiffA_3   
   
      Matching Genes:  
               gi|115249127|emb|CAJ66938.1|  (putative subunit of oxidoreductase [Clostridium difficile 630]) 
           
  Protein Group 154   
      Expression Quality:  
         Score      Num Spectra      Num Peptides      High-Qual Peptides      % Coverage       45    1    1    1    7   
   
      Peptides:   
        Query    Observed    Mr(expt)    Mr(calc)    Score    Peptide    Result File   
		    107    552.04    1102.06    1101.57    45    IPTWIDTEK    biorepCdiffA_1   
   
      Matching Genes:  
               gi|115250980|emb|CAJ68809.1|  (conserved hypothetical protein [Clostridium difficile 630]) 
           
  Protein Group 155   
      Expression Quality:  
         Score      Num Spectra      Num Peptides      High-Qual Peptides      % Coverage       45    1    1    1    23   
   
      Peptides:   
        Query    Observed    Mr(expt)    Mr(calc)    Score    Peptide    Result File   
		    784    784.73    2351.17    2350.11    45    EAEAALNAFMSSVQDALVNNEK    biorepCdiff_1   
   
      Matching Genes:  
               gi|115252557|emb|CAJ70400.1|  (DNA-binding protein HU [Clostridium difficile 630]) 
           
  Protein Group 156   
      Expression Quality:  
         Score      Num Spectra      Num Peptides      High-Qual Peptides      % Coverage       44    2    1    1    7   
   
      Peptides:   
        Query    Observed    Mr(expt)    Mr(calc)    Score    Peptide    Result File   
		    460    748.10    1494.18    1493.85    44    AGVVLNPATPVDTIK    biorepCdiff_3   
   
      Matching Genes:  
               gi|115251631|emb|CAJ69464.1|  (ribulose-phosphate 3-epimerase [Clostridium difficile 630]) 
           
  Protein Group 157   
      Expression Quality:  
         Score      Num Spectra      Num Peptides      High-Qual Peptides      % Coverage       43    2    1    1    4   
   
      Peptides:   
        Query    Observed    Mr(expt)    Mr(calc)    Score    Peptide    Result File   
		    350    726.24    1450.46    1449.75    43    GFFPEEELITLR    biorepCdiff_4   
   
      Matching Genes:  
               gi|115251377|emb|CAJ69209.1|  (transketolase [Clostridium difficile 630]) 
           
  Protein Group 158   
      Expression Quality:  
         Score      Num Spectra      Num Peptides      High-Qual Peptides      % Coverage       42    2    1    1    3   
   
      Peptides:   
        Query    Observed    Mr(expt)    Mr(calc)    Score    Peptide    Result File   
		    160    689.97    1377.93    1377.68    42    EDLFNSLEVGQK    biorepCdiffA_6   
   
      Matching Genes:  
               gi|115250007|emb|CAJ67827.1|  (putative 30S ribosomal protein S1 [Clostridium difficile 630]) 
           
  Protein Group 159   
      Expression Quality:  
         Score      Num Spectra      Num Peptides      High-Qual Peptides      % Coverage       41    1    1    1    19   
   
      Peptides:   
        Query    Observed    Mr(expt)    Mr(calc)    Score    Peptide    Result File   
		    179    670.49    1338.97    1338.70    41    VSHLLEVTEIAE    biorepCdiffA_1   
   
      Matching Genes:  
               gi|115249095|emb|CAJ66906.1|  (50S ribosomal protein L30 [Clostridium difficile 630]) 
           
  Protein Group 160   
      Expression Quality:  
         Score      Num Spectra      Num Peptides      High-Qual Peptides      % Coverage       41    1    1    1    4   
   
      Peptides:   
        Query    Observed    Mr(expt)    Mr(calc)    Score    Peptide    Result File   
		    215    628.58    1255.14    1254.72    41    IANAILVGDIEK    biorepCdiff_4   
   
      Matching Genes:  
               gi|115249121|emb|CAJ66932.1|  (phosphate butyryltransferase [Clostridium difficile 630]) 
           
  Protein Group 161   
      Expression Quality:  
         Score      Num Spectra      Num Peptides      High-Qual Peptides      % Coverage       41    4    1    1    17   
   
      Peptides:   
        Query    Observed    Mr(expt)    Mr(calc)    Score    Peptide    Result File   
		    453    1147.87    2293.74    2293.12    41    LADVAELDTLLSDKEYEAGLE    biorepCdiffA_2   
   
      Matching Genes:  
               gi|115249746|emb|CAJ67563.1|  (putative glycine cleavage system H protein [Clostridium difficile 630]) 
           
  Protein Group 162   
      Expression Quality:  
         Score      Num Spectra      Num Peptides      High-Qual Peptides      % Coverage       41    1    1    1    3   
   
      Peptides:   
        Query    Observed    Mr(expt)    Mr(calc)    Score    Peptide    Result File   
		    319    736.59    1471.17    1470.69    41    ENMQNHVETLTR    biorepCdiff_5   
   
      Matching Genes:  
               gi|115250313|emb|CAJ68135.1|  (cysteine desulfurase [Clostridium difficile 630]) 
           
  Protein Group 163   
      Expression Quality:  
         Score      Num Spectra      Num Peptides      High-Qual Peptides      % Coverage       41    1    1    1    4   
   
      Peptides:   
        Query    Observed    Mr(expt)    Mr(calc)    Score    Peptide    Result File   
		    259    693.17    1384.32    1383.80    41    ILITGSPIGGISEK    biorepCdiff_5   
   
      Matching Genes:  
               gi|115250793|emb|CAJ68617.1|  (putative 2-hydroxyacyl-CoA dehydratase [Clostridium difficile 630]) 
             
