# Supplementary material for: Semiquantitative Analysis of Clinical Heat Stress in Clostridium difficile Strain 630 Using a GeLC/MS Workflow with emPAI Quantitation
Source: PLoS One. 2014 Feb 24;9(2):e88960. doi: 10.1371/journal.pone.0088960 (PMC3933415; doi:10.1371/journal.pone.0088960)
Supplement: File S4 — PROVALT output html file – overall 37°C proteome, 224 proteins. (HTML) [file pone.0088960.s004.html]

   Complete output   Complete output    
 
   
  Protein Group 1   
      Expression Quality:  
         Score      Num Spectra      Num Peptides      High-Qual Peptides      % Coverage       2608    175    45    34    41   
   
      Peptides:   
        Query    Observed    Mr(expt)    Mr(calc)    Score    Peptide    Result File   
		    484    1051.45    2100.88    2100.04    56    ALASQALSIFGDHQDVMAAR    biorepCdiff_7   
		    382    1059.27    2116.52    2116.04    50    ALASQALSIFGDHQDVMAAR +Oxidation (M)    37_8   
		    281    891.98    1781.95    1781.83    70    ATYLIDEADYIACHK    37A_8   
		    36    481.35    960.69    960.54    28    DFILAQVR    biorepCdiffA_8   
		    105    528.88    1055.74    1056.54    37    ENAPMIIGGR    37_8   
		    258    767.03    1532.04    1531.75    72    EPGSTGEPLYLDVR    37_8   
		    2    403.26    804.51    804.41    32    FGDTPIR    37A_8   
		    55    471.29    940.56    940.50    33    FYTVNAVK    37A_7   
		    472    1335.72    2669.42    2669.17    60    GPSWANSLFEDNAEYGFGMYTAVK +Oxidation (M)    37A_8   
		    312    756.01    1510.01    1509.72    60    GTAQNPDIYFQTR    37_7   
		    226    702.00    1401.99    1401.67    44    HFLDAMPSTVER    37_8   
		    235    709.98    1417.95    1417.67    44    HFLDAMPSTVER +Oxidation (M)    37_8   
		    226    758.40    1514.78    1514.67    70    HSLFDYYGAEDAK    37A_8   
		    551    1168.80    2335.58    2335.01    75    IQVSPLDCTGCGNCADICPAK    37_7   
		    362    1020.77    2039.53    2038.93    64    IVDAMTELVSMDICEDAK    37_8   
		    370    1036.72    2071.42    2070.92    73    IVDAMTELVSMDICEDAK +2 Oxidation (M)    37_8   
		    367    1028.82    2055.62    2054.92    90    IVDAMTELVSMDICEDAK +Oxidation (M)    37_8   
		    347    938.77    1875.53    1874.99    97    IVNMNYAAVDAGINALVK    biorepCdiff_8   
		    415    946.70    1891.39    1890.99    76    IVNMNYAAVDAGINALVK +Oxidation (M)    37_7   
		    277    822.56    1643.11    1642.76    87    KHSLFDYYGAEDAK    biorepCdiff_8   
		    285    663.94    1325.88    1325.71    47    LAEIIPEEDAVK    37A_1   
		    131    528.89    1055.77    1055.57    59    LGQEIGLGNR    37_7   
		    197    466.03    1395.08    1394.71    39    LPFIHFFDGFR    biorepCdiff_8   
		    566    1202.88    2403.74    2403.22    63    NGFTVGIVDDVTNTSLTPSEPIK    37_7   
		    583    1305.00    3911.98    3911.85    43    NILRPMTAQEGNNLPVSTFNGIEDGTFPCGTAAYEK    37A_8   
		    73    461.31    920.61    920.46    42    NPFTLDSK    37_8   
		    202    676.51    1351.01    1350.65    34    QFPEEADGLFAK    biorepCdiff_7   
		    401    1095.20    2188.38    2188.96    71    QPLMEFSGACAGCGETAYIK    37_8   
		    406    736.16    2205.47    2204.95    69    QPLMEFSGACAGCGETAYIK +Oxidation (M)    37_8   
		    73    529.77    1057.53    1057.53    63    SGGITMSHLR    37A_8   
		    81    537.81    1073.61    1073.53    45    SGGITMSHLR +Oxidation (M)    37A_8   
		    373    881.65    1761.29    1760.89    115    TKEPGSTGEPLYLDVR    37_7   
		    340    1055.14    2108.26    2107.99    83    TVANEAQAVACGYWHLYR    37A_8   
		    193    688.97    1375.93    1375.70    39    TVFDNLVSEQPK    37A_8   
		    261    518.27    1551.79    1550.86    38    VAGELLPGVFHVSAR    biorepCdiff_8   
		    112    537.46    1072.90    1072.56    28    VDVMPANTVK    biorepCdiff_7   
		    140    545.34    1088.67    1088.55    37    VDVMPANTVK +Oxidation (M)    37_7   
		    451    1285.77    2569.52    2569.27    96    VELLENEDYASLLNFEAVQAFR    37A_8   
		    282    815.53    1629.05    1628.77    62    VEVPASWENAVDADK    37_8   
		    178    647.44    1292.87    1292.67    28    VHLYRPFSMK +Oxidation (M)    37_8   
		    307    953.67    1905.33    1904.89    55    VVELLEKPACDCTDEK    37A_8   
		    130    525.82    1049.63    1049.55    49    VVTQLYGDR    37_7   
		    191    685.84    1369.66    1369.58    69    YAQAYFDYDSK    37A_8   
		    223    749.89    1497.77    1497.68    52    YAQAYFDYDSKK    37A_8   
		    231    663.94    1325.87    1324.70    64    YYQNIVGIVEK    37_7   
   
      Matching Genes:  
               gi|115251733|emb|CAJ69568.1|  (pyruvate-flavodoxin oxidoreductase [Clostridium difficile 630]) 
           
  Protein Group 2   
      Expression Quality:  
         Score      Num Spectra      Num Peptides      High-Qual Peptides      % Coverage       1442    70    22    20    42   
   
      Peptides:   
        Query    Observed    Mr(expt)    Mr(calc)    Score    Peptide    Result File   
		    457    680.76    2039.24    2038.93    92    AAQEQQAAQGAEQAQDNGPK    37_7   
		    662    1053.85    3158.54    3157.42    39    AAQEQQAAQGAEQAQDNGPKDDNVVDADFK    37_7   
		    302    927.06    1852.11    1851.80    67    DDNVVDADFKEVDEDK    37A_7   
		    257    681.42    1360.83    1360.65    62    DNQDATAEELKK    37_7   
		    484    1065.30    2128.58    2128.03    82    EKIEAFNQAESTIYQTEK    37_7   
		    567    1204.92    2407.83    2407.19    92    ELSSTMSSNINLPFITATAEGPK    37_7   
		    575    1212.87    2423.72    2423.19    57    ELSSTMSSNINLPFITATAEGPK +Oxidation (M)    37_7   
		    100    484.34    966.66    966.52    47    HLNIDLSR    37_7   
		    410    936.67    1871.32    1870.89    95    IEAFNQAESTIYQTEK    37_7   
		    265    839.66    1677.31    1676.84    61    IINEPTAAALAYGMDK    37A_7   
		    366    847.61    1693.21    1692.84    60    IINEPTAAALAYGMDK +Oxidation (M)    37_7   
		    59    477.88    953.75    953.55    46    IPAVQEAVK    37A_7   
		    138    541.90    1081.79    1081.65    29    IPAVQEAVKK    37_7   
		    178    617.89    1233.76    1233.61    66    ISSGEKEDIEK    37_7   
		    413    946.17    1890.33    1889.96    109    ITITSNTNLSEAEIEQK    37_7   
		    639    978.40    2932.16    2931.41    63    SDAESYLGQTVTEAVITVPAYFTDAQR    37_7   
		    588    1250.81    2499.60    2499.24    106    SQIFSTAADNQTAVDIHVLQGER    37_7   
		    340    796.16    1590.31    1589.87    43    SYTPQEISAIILQK    37_7   
		    435    1187.87    2373.72    2373.20    85    TALQDAGLSTGDIDDVLLVGGSTR    37A_7   
		    70    445.29    888.56    888.46    54    TLNELGDK    37_7   
		    82    525.83    1049.65    1049.58    45    TTPSVVAFTK    37A_7   
		    119    506.79    1011.57    1011.47    42    VSQEMYQK    37_7   
   
      Matching Genes:  
               gi|115251515|emb|CAJ69348.1|  (chaperone protein [Clostridium difficile 630]) 
           
  Protein Group 3   
      Expression Quality:  
         Score      Num Spectra      Num Peptides      High-Qual Peptides      % Coverage       1386    54    26    21    44   
   
      Peptides:   
        Query    Observed    Mr(expt)    Mr(calc)    Score    Peptide    Result File   
		    492    1086.38    2170.74    2170.04    59    ATYTMIFDHYEQVPASVAK    37_7   
		    497    1094.32    2186.62    2186.04    65    ATYTMIFDHYEQVPASVAK +Oxidation (M)    37_7   
		    566    1297.60    3889.77    3888.85    28    DTTTGDTLCDPANPIILESMEFPEPVISVAIEPSSK +Oxidation (M)    37A_7   
		    259    681.98    1361.95    1361.74    60    EDSFIGIIDLLK    37_7   
		    172    679.43    1356.85    1356.64    64    GGVEPQSENVWR    37A_7   
		    155    580.35    1158.68    1158.55    53    GILADGEEAER    37_7   
		    234    783.08    1564.15    1563.72    53    HSSDEEPFSALAFK    37A_7   
		    509    713.51    2137.51    2136.87    39    IGETHEGASQMDWMEQEK +2 Oxidation (M)    biorepCdiff_7   
		    449    808.46    2422.35    2422.02    30    IGETHEGASQMDWMEQEKER +2 Oxidation (M)    37A_7   
		    110    604.38    1206.74    1206.64    42    ILFYTGQTHK    37A_7   
		    85    542.34    1082.66    1082.57    38    ILQMHANTR    37A_7   
		    75    512.28    1022.54    1022.51    39    IMTDPFVGK +Oxidation (M)    37A_7   
		    124    625.39    1248.77    1248.62    71    LAEEDPTFTVK    37A_7   
		    208    741.49    1480.97    1480.81    50    LNSNAVPMQLPIGK    37A_7   
		    307    749.52    1497.02    1496.80    49    LNSNAVPMQLPIGK +Oxidation (M)    37_7   
		    281    878.53    1755.05    1754.80    47    LVESVAETDEELMMK +2 Oxidation (M)    37A_7   
		    74    510.82    1019.63    1019.50    42    QAETYGVPR    37A_7   
		    592    835.00    2501.98    2501.23    83    SGAQVINAFVPLSEMFGYSTDLR    37_7   
		    595    1259.92    2517.83    2517.22    54    SGAQVINAFVPLSEMFGYSTDLR +Oxidation (M)    37_7   
		    157    587.85    1173.69    1173.57    55    VAPQEPGEGYK    37_7   
		    528    1129.79    2257.56    2256.99    41    VEVVTPEDYMGDVMGDLNSR +2 Oxidation (M)    37_7   
		    521    1121.81    2241.61    2240.99    42    VEVVTPEDYMGDVMGDLNSR +Oxidation (M)    37_7   
		    60    480.87    959.72    959.52    45    VGAPQVAYR    37A_7   
		    192    624.47    1246.92    1246.69    71    VYAGDIAAAVGLK    37_7   
		    376    895.15    1788.29    1787.89    101    VYSGTLESGSYVLNATK    37_7   
		    230    776.55    1551.09    1550.77    65    YLEGEELTIDELK    37A_7   
   
      Matching Genes:  
               gi|115249074|emb|CAJ66885.1|  (translation elongation factor G [Clostridium difficile 630]) 
           
  Protein Group 4   
      Expression Quality:  
         Score      Num Spectra      Num Peptides      High-Qual Peptides      % Coverage       1353    106    21    19    54   
   
      Peptides:   
        Query    Observed    Mr(expt)    Mr(calc)    Score    Peptide    Result File   
		    92    498.32    994.62    994.52    67    AEAHIQAGAK    biorepCdiff_5   
		    86    505.83    1009.65    1009.65    63    AIGLVIPSLK    37A_4   
		    128    619.91    1237.81    1237.64    77    DKAEAHIQAGAK    37_5   
		    294    718.08    1434.15    1433.74    53    FEVVAINDLTDAK    biorepCdiff_5   
		    66    468.26    934.50    934.48    60    FNGEIEVK    37A_4   
		    584    808.91    2423.70    2423.15    62    GLMTTIHAYTNDQNTLDGPHPK    37_6   
		    502    814.10    2439.28    2439.15    36    GLMTTIHAYTNDQNTLDGPHPK +Oxidation (M)    37A_4   
		    255    649.98    1297.94    1297.76    67    KVVISAPATGDLK    37_4   
		    23    430.28    858.54    858.48    30    MLAHLFK    37A_5   
		    474    1163.34    2324.67    2324.10    98    MMEQQDKFEVVAINDLTDAK    37_5   
		    582    1179.31    2356.61    2356.09    73    MMEQQDKFEVVAINDLTDAK +2 Oxidation (M)    37_4   
		    476    1171.28    2340.55    2340.10    84    MMEQQDKFEVVAINDLTDAK +Oxidation (M)    37_5   
		    508    927.52    2779.55    2778.36    75    NPADLPWAELGVDIVLECTGFFTSK    biorepCdiffA_1   
		    211    659.86    1317.70    1317.66    61    NVTVEEINAAMK    37A_4   
		    281    667.94    1333.86    1333.65    57    NVTVEEINAAMK +Oxidation (M)    37_4   
		    109    556.44    1110.87    1110.61    55    TLGYFAQLAK    37A_4   
		    177    589.88    1177.75    1177.63    64    VLNDKYGIEK    37_4   
		    579    887.35    1772.68    1771.98    98    VPVVTGSITELVCTLGK    biorepCdiff_2   
		    174    585.90    1169.79    1169.67    58    VVISAPATGDLK    37_4   
		    635    1046.29    2090.56    2089.98    65    VVSWYDNEMSYTSQLIR    biorepCdiff_2   
		    445    1054.23    2106.44    2105.97    50    VVSWYDNEMSYTSQLIR +Oxidation (M)    37A_4   
   
      Matching Genes:  
               gi|115252231|emb|CAJ70071.1|  (glyceraldehyde-3-phosphate dehydrogenase 2 [Clostridium difficile 630]) 
           
  Protein Group 5   
      Expression Quality:  
         Score      Num Spectra      Num Peptides      High-Qual Peptides      % Coverage       1321    71    26    16    35   
   
      Peptides:   
        Query    Observed    Mr(expt)    Mr(calc)    Score    Peptide    Result File   
		    130    681.60    1361.19    1360.69    34    AVTDLLDEMNIK    biorepCdiffA_7   
		    106    641.46    1280.92    1280.55    40    DMYLNSYYGR    biorepCdiffA_7   
		    168    462.26    922.50    923.42    36    EFGVEESK    37_2   
		    45    497.84    993.67    994.47    34    ENMLWFR    biorepCdiffA_7   
		    133    533.83    1065.65    1065.46    47    ETGMGIAEDK +Oxidation (M)    37_7   
		    209    744.49    1486.96    1486.77    39    EVDLTLATGGPGMVK    37A_7   
		    54    433.24    864.47    864.40    49    FNSSDAPK    37_7   
		    137    688.54    1375.06    1374.78    48    FQSLLVAIEDLK    biorepCdiffA_7   
		    452    1011.19    2020.37    2019.92    57    HTTFFEVEPDPTLECAK    37_7   
		    568    1205.91    2409.81    2409.35    30    IAEPIGVIAAVVPTTNPTSTAIFK    37_7   
		    284    714.98    1427.94    1427.69    58    IFATYSQEQVDK    37_7   
		    119    560.02    1118.02    1117.65    58    IFLAASLAANK    biorepCdiff_7   
		    359    1019.31    2036.60    2036.06    85    ILINTPSSQGGIGDLYNFK    37A_7   
		    100    581.50    1160.98    1160.62    78    IVGQTACTIAK    37A_7   
		    84    588.52    1175.02    1174.62    36    LAGFEVPVDTK    biorepCdiffA_7   
		    99    511.54    1021.07    1020.57    30    LSPVLAMYK    biorepCdiff_7   
		    549    1161.83    2321.65    2321.09    91    LVEDGGFGHTSSLYIDDVNQR    37_7   
		    351    818.57    1635.13    1634.74    59    NHYASEYIYNAYK    37_7   
		    158    621.64    1241.27    1240.71    49    NIILVNGGLNSK    biorepCdiff_7   
		    124    516.36    1030.70    1030.60    71    TAVNSILVSK    37_7   
		    235    562.91    1685.72    1684.78    27    TCGVIEKDEAFGMTK    biorepCdiffA_7   
		    271    567.98    1700.93    1700.77    39    TCGVIEKDEAFGMTK +Oxidation (M)    37A_7   
		    423    710.19    2127.54    2127.07    77    VLIGEVESVEIEEAFAHEK    37A_2   
		    101    485.84    969.66    969.57    51    VPLAIMAQK    37_7   
		    110    493.85    985.68    985.56    29    VPLAIMAQK +Oxidation (M)    37_7   
		    151    570.43    1138.85    1138.64    69    YAGIASFLGLK    37_7   
   
      Matching Genes:  
               gi|115252023|emb|CAJ69859.1|  (aldehyde-alcohol dehydrogenase [includes: alcohol dehydrogenase and pyruvate-formate-lyase deactivase [Clostridium difficile 630]) 
           
  Protein Group 6   
      Expression Quality:  
         Score      Num Spectra      Num Peptides      High-Qual Peptides      % Coverage       1180    59    21    17    48   
   
      Peptides:   
        Query    Observed    Mr(expt)    Mr(calc)    Score    Peptide    Result File   
		    341    643.50    1927.48    1927.01    53    DKYPGLIFSQILGYGEK    37_5   
		    51    462.81    923.61    923.51    47    EGVEILHK    37A_5   
		    202    697.95    1393.89    1393.65    44    ENPNSPLMTTYK    37_5   
		    275    706.04    1410.07    1409.65    37    ENPNSPLMTTYK +Oxidation (M)    biorepCdiff_5   
		    83    511.31    1020.61    1020.49    66    GGVSQSVMEK    37_5   
		    92    549.86    1097.71    1097.57    29    IEPIEGDGIR    37_5   
		    519    1279.29    2556.57    2556.15    74    IQSCEDLLDDEQAWANDFLFK    37_5   
		    69    495.31    988.61    988.53    60    IVGEAMLEK    37A_5   
		    297    739.10    1476.18    1475.80    43    LLSEADIFVTNVR    37_6   
		    97    519.94    1037.86    1037.52    34    MGIAYDQIK    biorepCdiff_5   
		    108    609.91    1217.80    1217.61    50    MLGDWGAEVIK    37_5   
		    112    617.93    1233.85    1233.61    50    MLGDWGAEVIK +Oxidation (M)    37A_5   
		    97    570.36    1138.71    1138.63    36    SKEGVEILHK    37_5   
		    250    779.01    1556.01    1555.75    58    SLGYDEEKINNFK    37_5   
		    348    997.65    1993.28    1992.84    77    SPASDDENPMFELENGNK    37A_5   
		    354    1005.61    2009.20    2008.83    70    SPASDDENPMFELENGNK +Oxidation (M)    37A_5   
		    387    1061.70    2121.39    2120.93    91    SPASDDENPMFELENGNKK    37A_5   
		    399    1069.71    2137.40    2136.93    66    SPASDDENPMFELENGNKK +Oxidation (M)    37A_5   
		    382    1053.73    2105.45    2105.02    73    TLDEWSALLEEADLPFEK    37A_6   
		    85    555.37    1108.72    1108.62    66    VGQHTVEVLK    37A_5   
		    182    695.47    1388.92    1388.78    56    WIQLALIQYNK    37A_5   
   
      Matching Genes:  
               gi|115249401|emb|CAJ67216.1|  (isocaprenoyl-CoA:2-hydroxyisocaproate CoA-transferase [Clostridium difficile 630]) 
           
  Protein Group 7   
      Expression Quality:  
         Score      Num Spectra      Num Peptides      High-Qual Peptides      % Coverage       1107    55    22    17    49   
   
      Peptides:   
        Query    Observed    Mr(expt)    Mr(calc)    Score    Peptide    Result File   
		    119    528.34    1054.66    1054.51    32    AFDITFADR    37_6   
		    376    864.89    1727.76    1727.89    44    ALCEELLAMPVVAGQK    biorepCdiff_6   
		    382    880.13    1758.25    1757.88    65    ALQSGTSHFLGQHFTK    37_6   
		    139    669.02    1336.03    1335.62    51    DIENNQAMVFR    biorepCdiffA_6   
		    245    676.93    1351.85    1351.62    61    DIENNQAMVFR +Oxidation (M)    37_6   
		    90    556.35    1110.68    1111.49    47    EADSMVVMAK +2 Oxidation (M)    37A_7   
		    128    548.83    1095.64    1095.49    46    EADSMVVMAK +Oxidation (M)    37_6   
		    442    755.51    2263.49    2263.07    64    EAEHVEGFAPEVAWVTHGGNK    37A_6   
		    403    914.16    1826.30    1825.82    42    EDNTSIVENMDEFRK    37_6   
		    410    922.14    1842.26    1841.81    34    EDNTSIVENMDEFRK +Oxidation (M)    37_6   
		    419    931.13    1860.25    1859.86    76    EGNLANPYHTSWGASTR    37_6   
		    448    963.18    1924.34    1923.90    53    GVAVEVDDRDNYTTGWK    biorepCdiff_6   
		    83    559.38    1116.75    1116.61    39    IKEETGATIR    37A_6   
		    85    560.82    1119.62    1119.56    80    KGNVMETVDK    37A_6   
		    485    1022.34    2042.67    2042.00    26    LCVRPTSETIICTMYAK    biorepCdiff_6   
		    330    944.57    1887.14    1886.84    45    MEDDFPQWYTDVITK    37A_6   
		    335    952.63    1903.24    1902.83    79    MEDDFPQWYTDVITK +Oxidation (M)    37A_6   
		    95    497.35    992.69    992.52    40    QFVEEITK    biorepCdiff_6   
		    84    560.80    1119.58    1119.58    59    TDLVDYAPVK    37A_6   
		    292    737.38    1472.74    1472.54    38    TMWCGDAECEAK +Oxidation (M)    37_6   
		    203    714.83    1427.64    1427.61    43    TYTIEAMMHDGK +2 Oxidation (M)    37A_6   
		    199    709.57    1417.13    1416.91    43    VAPIQVVIVPIAAK    37A_6   
   
      Matching Genes:  
               gi|115249053|emb|CAJ66864.1|  (putative dual-specificity prolyl/cysteinyl-tRNA synthetase [Clostridium difficile 630]) 
           
  Protein Group 8   
      Expression Quality:  
         Score      Num Spectra      Num Peptides      High-Qual Peptides      % Coverage       1027    43    18    15    39   
   
      Peptides:   
        Query    Observed    Mr(expt)    Mr(calc)    Score    Peptide    Result File   
		    455    814.23    2439.68    2439.29    59    DSPLVVGVGEGENFIASDIPALLK    37A_7   
		    156    583.37    1164.72    1164.59    35    EIHEQPTGVR    37_7   
		    382    1086.85    2171.68    2171.01    67    ESDDVFYTWAGPEVAVASTK    37A_7   
		    417    611.49    1831.46    1830.90    49    FESQTDTEVIAHLVDK    biorepCdiff_6   
		    370    861.17    1720.32    1719.92    75    FVNIPVITDIASEFR    37_6   
		    247    679.45    1356.89    1356.71    67    GAYVVAIAQSHNK    37_6   
		    258    700.48    1398.95    1398.67    73    GIDYSLAMEGSLK +Oxidation (M)    37_6   
		    105    614.52    1227.03    1226.69    45    GTPVIAIATQEK    biorepCdiffA_6   
		    304    749.48    1496.96    1496.67    105    GYDSAGVAVNSSNEK    37_6   
		    64    491.34    980.67    980.57    56    HGTIALIEK    37A_6   
		    286    715.56    1429.10    1428.83    65    ILSITNVVGSSIAR    37_7   
		    228    753.87    1505.73    1505.76    50    IQEILDNEEYIK    37A_6   
		    112    622.47    1242.93    1242.72    81    KAAEVIVEGLSK    biorepCdiffA_6   
		    149    563.84    1125.66    1125.48    43    MVSNMEEVR +2 Oxidation (M)    37_7   
		    80    555.39    1108.76    1109.48    26    MVSNMEEVR +Oxidation (M)    37A_6   
		    279    733.56    1465.10    1464.74    48    TVVSSEHAFYLGR    biorepCdiff_6   
		    287    885.72    1769.43    1768.92    36    VYIVACGTAYNAGLLGK    37A_7   
		    580    754.05    2259.14    2258.03    47    WATHGEPSDVNSHPHFNQAK    biorepCdiff_6   
   
      Matching Genes:  
               gi|115249129|emb|CAJ66940.1|  (glucosamine--fructose-6-phosphate aminotransferase [isomerizing] [Clostridium difficile 630]) 
           
  Protein Group 9   
      Expression Quality:  
         Score      Num Spectra      Num Peptides      High-Qual Peptides      % Coverage       1019    35    21    12    34   
   
      Peptides:   
        Query    Observed    Mr(expt)    Mr(calc)    Score    Peptide    Result File   
		    271    484.38    1450.13    1449.69    37    AGAPFAPGANPMHGR    biorepCdiff_7   
		    416    757.16    2268.47    2268.04    36    AGIVTEEELQEYMDHFVMK    37A_7   
		    58    476.27    950.53    950.49    34    AVMPYGGIK +Oxidation (M)    37A_7   
		    150    568.32    1134.62    1134.51    34    DQNGAAMSLGR +Oxidation (M)    37_7   
		    494    936.61    2806.80    2807.29    64    EFIQLNYSPYEGNDSFLAGATENTK    37A_7   
		    199    717.40    1432.79    1432.68    58    ERENGGTLDVDTK    37A_7   
		    363    1033.19    2064.36    2064.92    68    FEPITSEYLDYDEVMSK    37A_7   
		    467    1041.71    2081.40    2080.92    46    FEPITSEYLDYDEVMSK +Oxidation (M)    37_7   
		    141    460.73    1379.17    1378.73    53    KLWDEAMVLFK    biorepCdiffA_7   
		    451    1214.83    2427.65    2427.24    28    LPYEHAQDGISNTFSIVPAALGK    37A_7   
		    102    626.61    1251.20    1250.64    31    LWDEAMVLFK    biorepCdiffA_7   
		    388    923.61    1845.20    1844.86    90    MAESYGFDISKPATNSK    37_7   
		    401    621.44    1861.30    1860.86    47    MAESYGFDISKPATNSK +Oxidation (M)    37_7   
		    43    491.35    980.69    980.45    31    MNAWQGFK    biorepCdiffA_7   
		    368    1066.36    2130.71    2129.93    34    MVENSCEAFGYELDPEIK    biorepCdiffA_7   
		    469    1043.36    2084.71    2084.08    50    NSYPTQSILTITSNVVYGK    37_7   
		    132    660.47    1318.93    1318.69    53    SGIITGLPDAYGR    biorepCdiffA_6   
		    67    549.47    1096.92    1096.59    33    VSGYAVNFIK    biorepCdiffA_7   
		    437    986.69    1971.36    1970.89    104    VSIDTSSVQYENDDLMR    37_7   
		    443    994.66    1987.30    1986.88    48    VSIDTSSVQYENDDLMR +Oxidation (M)    37_7   
		    209    533.48    1597.43    1596.72    40    YSYEALEMALHDR    biorepCdiffA_7   
   
      Matching Genes:  
               gi|115249776|emb|CAJ67593.1|  (formate acetyltransferase [Clostridium difficile 630]) 
           
  Protein Group 10   
      Expression Quality:  
         Score      Num Spectra      Num Peptides      High-Qual Peptides      % Coverage       997    52    17    14    41   
   
      Peptides:   
        Query    Observed    Mr(expt)    Mr(calc)    Score    Peptide    Result File   
		    592    1058.89    3173.66    3173.59    99    ALEAANMTIEDIDLVEANEAFAAQSVAVIR    37A_5   
		    633    1064.39    3190.14    3189.59    55    ALEAANMTIEDIDLVEANEAFAAQSVAVIR +Oxidation (M)    37_5   
		    537    1370.81    2739.60    2738.43    86    ANITPDMIDESLLGGVLTAGLGQNIAR    37A_5   
		    539    1378.53    2755.05    2754.42    79    ANITPDMIDESLLGGVLTAGLGQNIAR +Oxidation (M)    37A_5   
		    325    936.68    1871.34    1871.00    63    AQAEGKFDEEIVPVVIK    37_5   
		    432    1132.33    2262.64    2262.10    82    DGTVTAGNASGINDGAAMLVVMAK    37A_5   
		    439    1148.11    2294.20    2294.09    71    DGTVTAGNASGINDGAAMLVVMAK +2 Oxidation (M)    37A_5   
		    461    1140.31    2278.61    2278.09    56    DGTVTAGNASGINDGAAMLVVMAK +Oxidation (M)    37_5   
		    72    481.82    961.63    961.45    35    DLNIDMNK    37_5   
		    226    738.02    1474.04    1473.69    69    EEQDELALASQNK    37_5   
		    141    644.50    1286.98    1286.71    47    FDEEIVPVVIK    biorepCdiffA_5   
		    113    613.01    1224.00    1223.68    43    ILTTLLYEMK    37_5   
		    120    620.90    1239.78    1239.68    38    ILTTLLYEMK +Oxidation (M)    37A_5   
		    121    567.42    1132.83    1132.60    39    IMGYGPVPATK    biorepCdiff_5   
		    96    575.39    1148.76    1148.59    45    IMGYGPVPATK +Oxidation (M)    37A_5   
		    218    616.37    1230.73    1230.68    45    SVSAVELGVTAAK    37A_1   
		    66    521.46    1040.90    1040.53    45    TAVGSFGGAFK    biorepCdiffA_5   
   
      Matching Genes:  
               gi|115250080|emb|CAJ67900.1|  (acetyl-CoA acetyltransferase [Clostridium difficile 630]) 
           
  Protein Group 11   
      Expression Quality:  
         Score      Num Spectra      Num Peptides      High-Qual Peptides      % Coverage       980    55    16    15    41   
   
      Peptides:   
        Query    Observed    Mr(expt)    Mr(calc)    Score    Peptide    Result File   
		    59    530.02    1058.02    1057.60    50    AVTVAVEELK    biorepCdiffA_6   
		    94    496.36    990.71    990.48    67    EADLSMLGR    biorepCdiff_6   
		    489    883.93    2648.76    2648.36    57    EMLQDIAILTGAQVISEELGYDLK    biorepCdiffA_1   
		    176    668.91    1335.82    1335.65    44    ESTTIVDGSGDKK    37A_6   
		    269    767.05    1532.09    1531.82    73    FGSPLITNDGVTIAK    biorepCdiffA_2   
		    77    468.40    934.78    934.51    57    GTFDVVAVK    biorepCdiff_6   
		    502    717.45    2149.34    2148.22    86    ISNIQELLPVLEQIVQQGK    37_6   
		    59    478.30    954.59    954.55    42    KALEEPLR    37A_6   
		    588    760.02    2277.03    2276.31    33    KISNIQELLPVLEQIVQQGK    biorepCdiff_6   
		    2    407.31    812.60    812.51    41    LAGGVAVVK    37A_6   
		    150    587.48    1172.94    1172.65    59    LIAEAMEIVGK    37_6   
		    613    709.15    2124.43    2125.19    72    LLIIAEDVEGEALSTLVVNK    37_2   
		    126    619.89    1237.76    1237.71    64    NVTAGANPILLR    37A_6   
		    520    1105.38    3313.11    3312.68    51    QIAINAGLEGAVIVQNVVNSEAETGFDALNEK    biorepCdiffA_2   
		    373    1044.81    2087.60    2087.09    108    TNDVAGDGTTTATVLAQAIIR    37A_6   
		    113    508.83    1015.64    1015.55    76    VGAATEVELK    37_6   
   
      Matching Genes:  
               gi|115249204|emb|CAJ67016.1|  (60 kDa chaperonin [Clostridium difficile 630]) 
           
  Protein Group 12   
      Expression Quality:  
         Score      Num Spectra      Num Peptides      High-Qual Peptides      % Coverage       943    43    15    15    59   
   
      Peptides:   
        Query    Observed    Mr(expt)    Mr(calc)    Score    Peptide    Result File   
		    219    665.01    1328.01    1327.73    67    ALENVLKDDLAK    37A_4   
		    526    1056.83    2111.65    2111.09    56    ALEVGIDPILCVGETLEQR    37_4   
		    126    596.34    1190.67    1190.62    50    EALEFVNEIK    37A_4   
		    331    717.99    1433.97    1433.74    64    EALEFVNEIKDK    37_4   
		    319    832.46    1662.91    1661.77    49    EIDMDYVVIGHSER    37A_4   
		    326    840.10    1678.18    1677.77    64    EIDMDYVVIGHSER +Oxidation (M)    37A_4   
		    138    610.84    1219.67    1219.62    64    GLYGELANEVR    37A_4   
		    260    652.39    1302.77    1302.60    67    IGAQNMHFEEK    37_4   
		    271    660.41    1318.80    1318.60    53    IGAQNMHFEEK +Oxidation (M)    37_4   
		    135    513.85    1025.68    1025.60    45    KPIIAGNWK    37_4   
		    342    744.46    1486.90    1486.66    58    QYFNETDETVNK    37_4   
		    301    808.49    1614.96    1614.75    76    QYFNETDETVNKK    37A_4   
		    390    820.05    1638.08    1637.79    91    TATAEDANDVISYIR    37_4   
		    425    1002.67    2003.33    2003.08    57    VNSDKVEAVICAPFTLLK    37A_4   
		    514    802.07    1602.12    1601.88    82    VVVAYEPIWAIGTGK    37_3   
   
      Matching Genes:  
               gi|115252229|emb|CAJ70069.1|  (triosephosphate isomerase [Clostridium difficile 630]) 
           
  Protein Group 13   
      Expression Quality:  
         Score      Num Spectra      Num Peptides      High-Qual Peptides      % Coverage       918    48    14    13    43   
   
      Peptides:   
        Query    Observed    Mr(expt)    Mr(calc)    Score    Peptide    Result File   
		    468    683.12    2046.33    2046.08    70    AAADEIGLPLFQYLGGVNAK    37_6   
		    641    1237.05    2472.08    2471.17    28    AGYKPGEDVMLGLDVAATEMYNK    biorepCdiff_6   
		    75    537.85    1073.68    1073.56    69    AGYTAVISHR    37A_6   
		    287    888.15    1774.29    1773.93    101    AIVPSGASTGAFEAVELR    37A_6   
		    169    664.89    1327.77    1327.76    71    EALELIVEAITK    37A_6   
		    159    657.48    1312.96    1312.73    83    GIENGVANSILVK    37A_6   
		    145    581.35    1160.69    1160.55    57    IEEMVGEQAR    37_6   
		    99    589.34    1176.66    1176.54    56    IEEMVGEQAR +Oxidation (M)    37A_6   
		    60    482.32    962.62    963.54    49    KYVLAGEGK    37A_6   
		    285    730.06    1458.11    1457.80    45    LGANAILGVSMAVAR +Oxidation (M)    37_6   
		    274    860.72    1719.42    1718.88    83    LQLVGDDLFVTNTER    37A_7   
		    84    560.03    1118.04    1117.56    65    MGAEVFHSLK    biorepCdiffA_5   
		    516    1095.30    2188.58    2188.09    93    SGETEDSTIADLAVAVNAGQIK    37_6   
		    57    525.50    1048.98    1048.59    48    SVIELVYAR    biorepCdiffA_6   
   
      Matching Genes:  
               gi|115252227|emb|CAJ70067.1|  (enolase [Clostridium difficile 630]) 
           
  Protein Group 14   
      Expression Quality:  
         Score      Num Spectra      Num Peptides      High-Qual Peptides      % Coverage       907    36    16    14    32   
   
      Peptides:   
        Query    Observed    Mr(expt)    Mr(calc)    Score    Peptide    Result File   
		    614    1342.45    2682.88    2682.27    103    AEVTDVANAIYDGTDAIMLSGETAAGK    37_7   
		    98    574.32    1146.63    1146.55    56    DGEVVTVDASR    37A_7   
		    573    806.17    2415.49    2415.20    50    DISDIEFGISQGIDYIAASFVR    37_7   
		    396    928.25    1854.48    1853.94    41    GDLGVEIPTEEMPIVQK    37_7   
		    278    872.08    1742.14    1741.87    85    IENQEGVENLDEILK    37A_7   
		    38    483.92    965.82    965.59    47    INLPAITPK    biorepCdiffA_7   
		    65    486.88    971.74    971.58    46    KASDVLAIR    37A_7   
		    219    649.39    1296.77    1296.59    31    RTEETLDYDR    37_7   
		    222    765.51    1529.00    1528.81    54    SGDSILIDDGLVGLR    37A_7   
		    160    594.39    1186.76    1186.62    68    SPIIATTNNEK    37_7   
		    225    674.97    1347.92    1347.65    98    SSVAGNTDEVIEK    biorepCdiff_6   
		    152    571.34    1140.66    1140.49    41    TEETLDYDR    37_7   
		    298    920.09    1838.17    1837.84    76    TGNFEDPEVFLEEGQK    37A_7   
		    57    474.36    946.71    946.49    40    VSDGIMVAR    37A_7   
		    62    482.28    962.54    962.49    30    VSDGIMVAR +Oxidation (M)    37A_7   
		    5    403.29    804.56    804.44    41    YPVEAVK    37_7   
   
      Matching Genes:  
               gi|115252454|emb|CAJ70297.1|  (pyruvate kinase [Clostridium difficile 630]) 
           
  Protein Group 15   
      Expression Quality:  
         Score      Num Spectra      Num Peptides      High-Qual Peptides      % Coverage       877    61    17    12    51   
   
      Peptides:   
        Query    Observed    Mr(expt)    Mr(calc)    Score    Peptide    Result File   
		    613    1270.90    2539.79    2539.30    78    AYEGGFAIGAFNISDLEQLQGVLK    37_4   
		    143    530.38    1058.74    1058.56    66    DAIQAVVESK    37_4   
		    140    611.47    1220.93    1220.63    60    FDILEEIQSK    37A_4   
		    8    409.73    817.46    817.43    34    FLAENPK    37A_4   
		    87    609.58    1217.14    1216.59    31    IDDVLGSANSIN    biorepCdiffA_4   
		    115    489.27    976.53    976.46    59    INMDTDLR    37_4   
		    83    497.23    992.44    992.46    53    INMDTDLR +Oxidation (M)    37A_4   
		    72    473.78    945.54    945.53    49    KFLAENPK    37A_4   
		    29    423.97    845.93    845.48    32    LAMTAAIR    biorepCdiff_4   
		    40    431.80    861.58    861.47    34    LAMTAAIR +Oxidation (M)    37A_4   
		    289    780.94    1559.87    1559.73    56    NSYVMIQASMSAVK +2 Oxidation (M)    37A_4   
		    285    772.91    1543.81    1543.74    55    NSYVMIQASMSAVK +Oxidation (M)    37A_4   
		    654    911.80    2732.39    2732.18    30    TCIDAGFSSVMIDGSHFDFEENVR    biorepCdiff_4   
		    346    873.09    1744.17    1743.92    63    TGVDSLAIAIGTSHGAFK    37A_4   
		    283    448.97    1343.90    1343.69    58    YAGPHTLVEMVK    37_4   
		    231    680.98    1359.94    1359.69    57    YAGPHTLVEMVK +Oxidation (M)    37A_4   
		    481    770.11    1538.20    1537.74    62    YTQPAEAVEFVER    biorepCdiff_3   
   
      Matching Genes:  
               gi|115249409|emb|CAJ67224.1|  (putative fructose-bisphosphate aldolase [Clostridium difficile 630]) 
           
  Protein Group 16   
      Expression Quality:  
         Score      Num Spectra      Num Peptides      High-Qual Peptides      % Coverage       826    20    15    12    28   
   
      Peptides:   
        Query    Observed    Mr(expt)    Mr(calc)    Score    Peptide    Result File   
		    229    757.47    1512.92    1512.83    30    AVLEGMDGKPIVIR +Oxidation (M)    37A_6   
		    106    513.89    1025.77    1025.59    41    GIGASPGVALGK    biorepCdiff_6   
		    67    500.73    999.44    1000.56    38    GKPSVTLDGK    37A_6   
		    310    925.74    1849.46    1848.97    37    IMFPMISSLEELLQAK    37A_6   
		    364    849.65    1697.29    1696.89    62    ISYLYNQFNPAVLR    37_6   
		    51    466.31    930.61    930.50    51    LEDAVAVSK    37A_6   
		    232    765.52    1529.03    1528.83    50    LEDAVAVSKEELVK    37A_6   
		    494    884.02    2649.03    2648.36    53    MIPILLGMGLDEFSMSPISILPAR +3 Oxidation (M)    37A_6   
		    612    873.21    2616.61    2616.37    80    MIPILLGMGLDEFSMSPISILPAR +Oxidation (M)    37_6   
		    199    639.97    1277.92    1277.68    77    MVLGFLTDIGGR    37_6   
		    213    647.95    1293.88    1293.68    64    MVLGFLTDIGGR +Oxidation (M)    37_6   
		    126    547.32    1092.63    1092.52    58    NDAEGVGLYR    37_6   
		    92    594.93    1187.84    1187.60    51    SIDNVEAEIAK    biorepCdiffA_6   
		    328    791.03    1580.05    1579.77    71    TESVNAEYALNEIK    37_6   
		    191    698.86    1395.70    1395.55    63    WAGMCGESAGDQK    37A_6   
   
      Matching Genes:  
               gi|115251808|emb|CAJ69643.1|  (phosphoenolpyruvate-protein phosphotransferase [Clostridium difficile 630]) 
           
  Protein Group 17   
      Expression Quality:  
         Score      Num Spectra      Num Peptides      High-Qual Peptides      % Coverage       820    90    12    12    64   
   
      Peptides:   
        Query    Observed    Mr(expt)    Mr(calc)    Score    Peptide    Result File   
		    114    452.34    902.67    902.53    56    AFLGLLNR    37A_1   
		    222    628.33    1254.64    1254.58    73    EGYPEVAEAYK    37A_2   
		    428    706.42    1410.83    1410.68    41    EGYPEVAEAYKR    37_3   
		    302    746.03    1490.04    1489.80    101    FAELLGEVVVADTK    37A_3   
		    480    880.60    2638.76    2638.14    40    FVCTVCGYIHEGDAAPAQCPVCK    37A_2   
		    286    615.32    1228.64    1228.53    46    GEMVWADEHR    37_2   
		    267    608.44    1214.86    1214.59    63    IAFEEAEHAAK    biorepCdiff_2   
		    541    820.57    1639.12    1638.89    121    IGVAQGVDAEIIEGLR    37_2   
		    250    563.30    1124.58    1124.50    70    VDAEYGATDGK    37_2   
		    247    577.34    1152.67    1152.55    58    VGADKFEEMK    37_3   
		    254    585.32    1168.63    1168.54    58    VGADKFEEMK +Oxidation (M)    37_3   
		    264    690.95    1379.88    1379.67    93    VRVDAEYGATDGK    37A_3   
   
      Matching Genes:  
               gi|115250565|emb|CAJ68389.1|  (putative rubrerythrin [Clostridium difficile 630]) 
           
  Protein Group 18   
      Expression Quality:  
         Score      Num Spectra      Num Peptides      High-Qual Peptides      % Coverage       808    35    13    12    38   
   
      Peptides:   
        Query    Observed    Mr(expt)    Mr(calc)    Score    Peptide    Result File   
		    522    1286.41    2570.81    2570.27    137    DILDILEDNNISVVADDLAQETR    37_5   
		    272    856.53    1711.05    1710.77    57    ELEEICGYEIEEAK    37_5   
		    100    585.38    1168.74    1168.61    55    EVVENPNAAVK    37_5   
		    259    851.96    1701.91    1701.73    51    FCDPEEYDYPLVR    37A_5   
		    99    576.89    1151.76    1151.64    53    HSNTIKPSIR    37_5   
		    243    509.29    1524.85    1524.73    59    IHESIEVYNEHR    37_5   
		    167    675.88    1349.75    1349.60    38    LNAMPEEVCSGK +Oxidation (M)    37A_5   
		    210    714.98    1427.94    1427.74    55    MKEVVENPNAAVK    37_5   
		    192    722.97    1443.93    1443.74    74    MKEVVENPNAAVK +Oxidation (M)    37A_5   
		    274    884.48    1766.94    1766.79    60    QWSNIEGCSLAYDPK    37A_5   
		    126    629.83    1257.65    1257.58    49    TDVPAGDDALER    37A_5   
		    92    565.44    1128.86    1128.68    69    VLLTGILADSK    37A_5   
		    108    613.92    1225.82    1225.66    51    YISLVHPQNR    37A_5   
   
      Matching Genes:  
               gi|115249404|emb|CAJ67219.1|  (subunit of oxygen-sensitive 2-hydroxyisocaproyl-CoA dehydratase [Clostridium difficile 630]) 
           
  Protein Group 19   
      Expression Quality:  
         Score      Num Spectra      Num Peptides      High-Qual Peptides      % Coverage       769    36    14    10    43   
   
      Peptides:   
        Query    Observed    Mr(expt)    Mr(calc)    Score    Peptide    Result File   
		    87    556.88    1111.74    1111.57    35    ALSIWMTFK +Oxidation (M)    37A_5   
		    337    748.28    1494.54    1493.72    30    DVNVFEMAQSQVK    biorepCdiff_5   
		    88    533.30    1064.58    1064.50    49    EAAYMHSIK +Oxidation (M)    37_5   
		    385    886.16    1770.30    1769.92    68    GGIIVDPSTLSQGELER    37_6   
		    53    464.82    927.62    927.47    34    GYIDGIYK    37A_5   
		    464    1011.78    2021.54    2021.03    89    IAVQGIGNVGSYTVLNCEK    37_6   
		    215    649.43    1296.85    1297.63    43    IKEEYNVTMR +Oxidation (M)    37_6   
		    175    681.99    1361.97    1361.73    46    LGMEPAVYELLK    37A_5   
		    452    1130.83    2259.65    2259.22    57    LTGQSSIGVITGKPVEFGGSLGR    37_5   
		    458    1138.77    2275.53    2275.04    68    LVCEAANGPTTPEADEVFAER    37_5   
		    496    1285.21    2568.41    2568.11    89    SEGSYAIYNENGLDGQAMLDYMK    37A_5   
		    502    1293.63    2585.24    2584.11    32    SEGSYAIYNENGLDGQAMLDYMK +Oxidation (M)    37A_5   
		    117    611.40    1220.79    1220.65    73    TAATGFGVAVTAR    37A_6   
		    103    492.36    982.70    982.61    56    VIEVSIPVK    37_6   
   
      Matching Genes:  
               gi|115249189|emb|CAJ67001.1|  (NAD-specific glutamate dehydrogenase [Clostridium difficile 630]) 
           
  Protein Group 20   
      Expression Quality:  
         Score      Num Spectra      Num Peptides      High-Qual Peptides      % Coverage       750    54    15    9    48   
   
      Peptides:   
        Query    Observed    Mr(expt)    Mr(calc)    Score    Peptide    Result File   
		    489    743.02    2226.04    2225.15    28    DAIDEIKPEIMLFGATHIGR    biorepCdiffA_5   
		    78    497.30    992.59    992.52    47    ELITFGADK    37_5   
		    211    715.44    1428.86    1429.75    29    EVDAELCAILLGK    37_5   
		    300    683.55    1365.08    1364.80    80    IAPVVIELLGEGR    37_4   
		    37    430.80    859.58    859.44    44    ITQDDIR    37A_4   
		    174    665.92    1329.82    1329.70    41    LDSVDDLLEAIK    37_5   
		    320    701.48    1400.95    1400.74    85    LDSVDDLLEAIKA    37_4   
		    98    543.88    1085.74    1085.56    37    LEIDPEDKK    37A_4   
		    14    416.30    830.59    830.46    47    LGGVVGSSR    37_5   
		    580    1006.38    3016.13    3015.60    60    NPAAPILEIADYGVVGDLHEIVPMLIEK    37A_4   
		    133    581.96    1161.90    1161.59    27    NVWIFAEQR    biorepCdiff_5   
		    108    554.88    1107.75    1107.58    93    TGEVIALDYK    37A_4   
		    53    445.83    889.65    889.51    35    TTVLETVK    37A_4   
		    95    561.92    1121.82    1121.54    69    VGTGLTADCTK    37_5   
		    112    481.79    961.57    961.44    28    YTTDAYTK    37_4   
   
      Matching Genes:  
               gi|115249407|emb|CAJ67222.1|  (electron transfer flavoprotein alpha-subunit [Clostridium difficile 630]) 
              Other Genes Matching Peptide Subset:  
               gi|115249822|emb|CAJ67639.1|  (electron transfer flavoprotein alpha-subunit [Clostridium difficile 630]) 
           
  Protein Group 21   
      Expression Quality:  
         Score      Num Spectra      Num Peptides      High-Qual Peptides      % Coverage       697    83    10    10    58   
   
      Peptides:   
        Query    Observed    Mr(expt)    Mr(calc)    Score    Peptide    Result File   
		    114    452.34    902.67    902.53    56    AFLGLLNR    37A_1   
		    196    621.37    1240.72    1240.56    53    EGYPEVGEAYK    37A_3   
		    302    746.03    1490.04    1489.80    101    FAELLGEVVVADTK    37A_3   
		    480    880.60    2638.76    2638.14    40    FVCTVCGYIHEGDAAPAQCPVCK    37A_2   
		    183    591.37    1180.73    1180.61    66    IALEEAEHAAK    37A_2   
		    385    849.69    1697.36    1696.90    102    IGVAQGVDEEIIEGLR    37A_1   
		    250    563.30    1124.58    1124.50    70    VDAEYGATDGK    37_2   
		    247    577.34    1152.67    1152.55    58    VGADKFEEMK    37_3   
		    254    585.32    1168.63    1168.54    58    VGADKFEEMK +Oxidation (M)    37_3   
		    264    690.95    1379.88    1379.67    93    VRVDAEYGATDGK    37A_3   
   
      Matching Genes:  
               gi|115250515|emb|CAJ68339.1|  (putative ruberythrin [Clostridium difficile 630]) 
           
  Protein Group 22   
      Expression Quality:  
         Score      Num Spectra      Num Peptides      High-Qual Peptides      % Coverage       666    36    11    8    28   
   
      Peptides:   
        Query    Observed    Mr(expt)    Mr(calc)    Score    Peptide    Result File   
		    207    652.59    1303.16    1302.71    36    ALQLHGGYGFIK    biorepCdiff_5   
		    198    631.40    1260.79    1260.59    51    ELDTLPAEMDK    37_7   
		    143    639.42    1276.83    1276.59    25    ELDTLPAEMDK +Oxidation (M)    37_5   
		    55    523.93    1045.84    1045.56    71    IAMGTLEVGR    biorepCdiffA_7   
		    76    531.80    1061.58    1061.55    70    IAMGTLEVGR +Oxidation (M)    37A_5   
		    322    627.38    1879.13    1879.08    100    IGVAALALGIAQGALDEAVK    37A_5   
		    292    727.02    1452.02    1451.75    94    IVSIYEGTSEVQK    37_7   
		    89    495.93    989.84    989.56    34    MVISSNVLK    biorepCdiff_5   
		    68    494.37    986.72    986.59    50    VQFGKPIAK    37A_5   
		    249    675.40    1348.78    1348.57    56    WDGFSTGAHEDK    37_7   
		    318    761.02    1520.02    1519.72    79    YYASEIANEVAYK    37_7   
   
      Matching Genes:  
               gi|115249405|emb|CAJ67220.1|  (acyl-CoA dehydrogenase, short-chain specific [Clostridium difficile 630]) 
           
  Protein Group 23   
      Expression Quality:  
         Score      Num Spectra      Num Peptides      High-Qual Peptides      % Coverage       660    37    12    9    42   
   
      Peptides:   
        Query    Observed    Mr(expt)    Mr(calc)    Score    Peptide    Result File   
		    448    905.14    1808.27    1807.91    93    AFAGADTWATSSALAGALK    37_4   
		    31    415.80    829.59    829.45    61    AGLEEAIK    37_4   
		    95    536.37    1070.73    1070.63    28    AGLEEAIKLK    37A_4   
		    515    1041.29    2080.56    2080.07    61    DGVPSIINPDDKAGLEEAIK    37_4   
		    199    650.34    1298.66    1298.67    56    DIEVDPSNLGLK    37A_4   
		    108    475.27    948.52    948.43    26    EALAMGADR +Oxidation (M)    37_4   
		    155    550.38    1098.75    1098.60    57    LDPNTGTLIR    37_4   
		    96    538.84    1075.66    1075.58    50    MPCLITTLK    37A_4   
		    101    546.89    1091.77    1091.57    58    MPCLITTLK +Oxidation (M)    37A_4   
		    87    508.76    1015.50    1015.52    39    QVPDTTEVK    37A_4   
		    276    747.04    1492.06    1491.76    78    SVKPAGTIYNEDAK    37A_4   
		    90    459.31    916.61    916.52    53    TSAGIIIDK    37_4   
   
      Matching Genes:  
               gi|115250076|emb|CAJ67896.1|  (electron transfer flavoprotein beta-subunit [Clostridium difficile 630]) 
           
  Protein Group 24   
      Expression Quality:  
         Score      Num Spectra      Num Peptides      High-Qual Peptides      % Coverage       645    15    9    8    22   
   
      Peptides:   
        Query    Observed    Mr(expt)    Mr(calc)    Score    Peptide    Result File   
		    415    1110.31    2218.60    2218.17    65    AFVVGGTGLADAMSIAPVASQLK +Oxidation (M)    37A_5   
		    392    1049.77    2097.53    2097.07    85    DGSTKEDQLVDALAAAPIAGR    37_5   
		    480    1178.90    2355.79    2355.22    64    ESPAPIILATDTLSSDQNVAVSK    37_5   
		    273    743.92    1485.83    1485.80    70    IITNQADAEAIVTK    37A_4   
		    380    1031.67    2061.33    2061.92    36    LGDGDYVDFSVDYNLENK    37_5   
		    163    660.94    1319.86    1319.71    62    LYNLVNTQLDK    37A_6   
		    199    737.91    1473.81    1473.63    79    SGGSEDTGYVVEMK +Oxidation (M)    37A_5   
		    330    940.17    1878.32    1877.97    106    VDVTGGSTPSAVAVSGFVTK    37_5   
		    221    773.88    1545.74    1545.69    78    YYNSDDKNAITDK    37A_5   
   
      Matching Genes:  
               gi|115251846|emb|CAJ69681.1|  (cell surface protein (S-layer precursor protein) [Clostridium difficile 630]) 
           
  Protein Group 25   
      Expression Quality:  
         Score      Num Spectra      Num Peptides      High-Qual Peptides      % Coverage       623    31    10    7    52   
   
      Peptides:   
        Query    Observed    Mr(expt)    Mr(calc)    Score    Peptide    Result File   
		    420    711.07    1420.12    1419.67    80    EIMDAANNTGASVK    biorepCdiff_3   
		    311    718.92    1435.82    1435.66    71    EIMDAANNTGASVK +Oxidation (M)    37A_2   
		    266    669.99    1337.97    1337.72    87    IVYDAFAIVAEK    37A_2   
		    249    558.82    1115.62    1115.60    68    LINNLMVDGK    37_2   
		    169    566.86    1131.70    1131.60    38    LINNLMVDGK +Oxidation (M)    37A_2   
		    430    696.54    1391.07    1390.69    54    REVLPDPMYGSK    biorepCdiff_2   
		    57    422.28    842.55    842.51    36    RQTLGLR    37_2   
		    378    647.48    1939.43    1939.04    35    RVGGANYQVPIEVRPER    biorepCdiffA_2   
		    209    618.89    1235.77    1235.60    95    TGEEALEVFNK    37A_2   
		    566    595.38    1783.11    1782.94    59    VGGANYQVPIEVRPER    37_2   
   
      Matching Genes:  
               gi|115249073|emb|CAJ66884.1|  (30S ribosomal protein S7 [Clostridium difficile 630]) 
           
  Protein Group 26   
      Expression Quality:  
         Score      Num Spectra      Num Peptides      High-Qual Peptides      % Coverage       616    31    11    6    29   
   
      Peptides:   
        Query    Observed    Mr(expt)    Mr(calc)    Score    Peptide    Result File   
		    202    641.44    1280.86    1280.66    70    ESTIEFLTSVR    37_6   
		    101    592.39    1182.76    1182.63    78    GISDFLLSFGK    37A_6   
		    301    759.13    1516.24    1515.83    30    GNVLEGLKPESVFK    biorepCdiff_6   
		    561    1151.77    2301.53    2301.01    96    HEFTTSDPGMTYSVAETSVDK    37_6   
		    562    1159.76    2317.50    2317.01    86    HEFTTSDPGMTYSVAETSVDK +Oxidation (M)    37_6   
		    72    515.28    1028.54    1028.45    35    ICSETYEK    37A_6   
		    614    874.75    2621.22    2622.38    42    LLAQALGANYDLIAQYPAWEFKK    37_6   
		    211    485.61    1453.82    1453.76    32    NVEHDFLKDPIK    37A_6   
		    302    748.02    1494.02    1493.71    29    SSVMSIRDEIADR +Oxidation (M)    37_6   
		    191    648.02    1294.02    1293.66    35    TCVVSLPVEYK    biorepCdiff_6   
		    350    990.26    1978.50    1978.03    83    VLSVLNVDYELASVDGGTK    37A_6   
   
      Matching Genes:  
               gi|115249724|emb|CAJ67541.1|  (putative aminoacyl-histidine dipeptidase [Clostridium difficile 630]) 
           
  Protein Group 27   
      Expression Quality:  
         Score      Num Spectra      Num Peptides      High-Qual Peptides      % Coverage       611    20    12    7    25   
   
      Peptides:   
        Query    Observed    Mr(expt)    Mr(calc)    Score    Peptide    Result File   
		    108    646.96    1291.91    1291.61    35    ELISNGCDAVSK    biorepCdiffA_7   
		    149    657.48    1312.94    1312.80    30    EVIPEFLLLLK    37A_7   
		    253    825.46    1648.91    1648.67    71    FAGMDLGMNFEEEK +2 Oxidation (M)    37A_7   
		    276    862.56    1723.11    1722.85    25    FIDNGIGMTEEEIKK    37A_7   
		    161    594.92    1187.83    1187.64    66    LVSLGEISENK    37_7   
		    302    769.63    1537.25    1536.79    33    LYNNQVFVADNIK    biorepCdiff_7   
		    377    895.16    1788.31    1787.87    61    NEDTPAMVLVSEQSIR    37_7   
		    122    671.00    1339.98    1339.77    40    TLVINENSPIIK    biorepCdiffA_7   
		    402    931.66    1861.31    1860.93    32    VFYVSDKEQQSQYIK    37_7   
		    254    826.59    1651.17    1650.90    48    VIEPLNDTNPLWLK    37A_7   
		    333    965.10    1928.19    1927.84    79    WISEGGTEYEISESDAR    37A_7   
		    301    925.61    1849.20    1848.87    91    YINQVAFSGAEDFFNK    37A_7   
   
      Matching Genes:  
               gi|115249282|emb|CAJ67095.1|  (chaperone protein (heat shock protein) [Clostridium difficile 630]) 
           
  Protein Group 28   
      Expression Quality:  
         Score      Num Spectra      Num Peptides      High-Qual Peptides      % Coverage       590    50    8    8    43   
   
      Peptides:   
        Query    Observed    Mr(expt)    Mr(calc)    Score    Peptide    Result File   
		    495    970.46    1938.91    1937.95    82    AFGGADTWATSNTIAAGISK    biorepCdiff_4   
		    630    694.50    2080.47    2080.03    65    DGVPSILNPDDANALEEALK    37_3   
		    355    883.45    1764.89    1764.80    105    ECLAMGADDAILLSDR +Oxidation (M)    37A_4   
		    420    871.10    1740.18    1739.87    111    QAIDGDTAQVGPQIAEK    37_4   
		    168    604.41    1206.81    1206.61    70    QLEDGYELIK    37A_3   
		    142    529.33    1056.65    1056.52    44    QVPDTNEVR    37_4   
		    180    613.33    1224.64    1224.61    67    VGDYDIIFAGR    37A_3   
		    142    564.35    1126.69    1126.70    46    VSTPVLLTAVK    37A_3   
   
      Matching Genes:  
               gi|115249406|emb|CAJ67221.1|  (electron transfer flavoprotein beta-subunit [Clostridium difficile 630]) 
           
  Protein Group 29   
      Expression Quality:  
         Score      Num Spectra      Num Peptides      High-Qual Peptides      % Coverage       537    39    11    8    47   
   
      Peptides:   
        Query    Observed    Mr(expt)    Mr(calc)    Score    Peptide    Result File   
		    33    416.78    831.55    831.45    48    CLALLDK    37_4   
		    512    692.17    2073.47    2073.12    68    ILIPMINEAVGIYADGVASK    37_4   
		    657    922.74    2765.19    2764.33    26    LAVIGSGTMGSGIVQTFASCGHDVCLK    biorepCdiff_4   
		    557    928.09    2781.26    2780.33    42    LAVIGSGTMGSGIVQTFASCGHDVCLK +Oxidation (M)    37A_4   
		    29    445.75    889.49    889.46    33    LLDELCK    biorepCdiffA_4   
		    701    1052.23    3153.68    3152.60    46    LLDELCKEDTILATNTSSLSITEIASSTK    biorepCdiff_4   
		    182    637.44    1272.87    1272.73    56    LVEVISGQLTSK    37A_4   
		    503    1022.22    2042.43    2042.08    50    SINKVPVDVSESPGFVVNR    37_4   
		    296    801.20    1600.39    1599.83    58    VPVDVSESPGFVVNR    37A_4   
		    130    643.53    1285.05    1284.66    73    VTFDTVFELSK    biorepCdiffA_4   
		    154    547.87    1093.73    1093.64    37    YRPHPLLAK    37_4   
   
      Matching Genes:  
               gi|115250079|emb|CAJ67899.1|  (3-hydroxybutyryl-CoA dehydrogenase [Clostridium difficile 630]) 
           
  Protein Group 30   
      Expression Quality:  
         Score      Num Spectra      Num Peptides      High-Qual Peptides      % Coverage       523    21    9    8    37   
   
      Peptides:   
        Query    Observed    Mr(expt)    Mr(calc)    Score    Peptide    Result File   
		    368    794.28    1586.54    1585.88    27    AADPIVVLFGATSIGR    biorepCdiff_5   
		    82    545.35    1088.69    1088.55    49    ATIDAGWLDK    37A_5   
		    459    1139.17    2276.33    2276.15    74    ENLDILYELAEIIGGEVSGSR    37_5   
		    219    771.99    1541.97    1541.87    63    ENVIQTVSLELLGK    37A_5   
		    352    987.17    1972.33    1971.96    94    IHTGLTADCTGLAVAEDTK    37_5   
		    118    629.63    1257.25    1256.69    69    MGNVLVVIEQR    biorepCdiffA_5   
		    268    699.21    1396.40    1395.83    54    VLPELISQLSVAK    biorepCdiff_5   
		    48    444.36    886.70    886.55    43    VSALLLGSK    37_5   
		    183    637.10    1272.18    1271.65    50    YADVGIVGDVHK    biorepCdiff_5   
   
      Matching Genes:  
               gi|115250077|emb|CAJ67897.1|  (electron transfer flavoprotein alpha-subunit [Clostridium difficile 630]) 
           
  Protein Group 31   
      Expression Quality:  
         Score      Num Spectra      Num Peptides      High-Qual Peptides      % Coverage       519    24    10    8    49   
   
      Peptides:   
        Query    Observed    Mr(expt)    Mr(calc)    Score    Peptide    Result File   
		    188    501.32    1000.62    1000.56    57    GTQAVGIVEK    37_3   
		    203    518.37    1034.73    1034.65    43    IILLGPPGAGK    37_3   
		    626    1037.80    2073.59    2073.07    67    IQVYLDETKPLVDYYSK    37_3   
		    18    409.25    816.48    816.43    44    NAGISLDK    37_3   
		    248    577.84    1153.67    1152.54    41    NGFMLDGFPR    37_3   
		    159    585.86    1169.70    1168.53    29    NGFMLDGFPR +Oxidation (M)    37A_3   
		    336    529.06    1584.16    1583.85    38    QGIIADIKGDQAIDK    37A_3   
		    576    906.61    1811.20    1810.80    84    VEGVCDVCQGELYQR    37_3   
		    138    458.31    914.60    914.51    60    VVNIEVDK    37_3   
		    314    511.64    1531.89    1531.78    56    YNIPHISTGDIFR    37A_3   
   
      Matching Genes:  
               gi|115249098|emb|CAJ66909.1|  (adenylate kinase [Clostridium difficile 630]) 
           
  Protein Group 32   
      Expression Quality:  
         Score      Num Spectra      Num Peptides      High-Qual Peptides      % Coverage       517    13    8    6    26   
   
      Peptides:   
        Query    Observed    Mr(expt)    Mr(calc)    Score    Peptide    Result File   
		    219    738.10    1474.18    1474.77    32    EEGIIFNTLTNPK    37A_6   
		    496    1069.26    2136.51    2136.05    72    EGVFAGGDAVTGAATVISAMGAGK    37_6   
		    155    593.89    1185.77    1185.60    61    LGSESYIVYR    37_6   
		    116    611.32    1220.63    1220.50    39    MELGEPDDSGR +Oxidation (M)    37A_6   
		    246    691.53    1381.05    1380.74    53    QAVVQPEIDNIR    biorepCdiff_6   
		    372    864.16    1726.30    1725.91    101    VAVIGSGPAGLACAGDLAK    37_6   
		    260    701.53    1401.04    1401.70    93    VAVVGGGNVAMDAAR +Oxidation (M)    37_6   
		    349    989.19    1976.37    1975.92    66    VCPQESQCEGVCILGIK    37A_6   
   
      Matching Genes:  
               gi|115250578|emb|CAJ68402.1|  (putative glutamate synthase [NADPH] small chain [Clostridium difficile 630]) 
           
  Protein Group 33   
      Expression Quality:  
         Score      Num Spectra      Num Peptides      High-Qual Peptides      % Coverage       516    17    8    7    29   
   
      Peptides:   
        Query    Observed    Mr(expt)    Mr(calc)    Score    Peptide    Result File   
		    247    831.49    1660.97    1660.76    63    AHCSTVGAGEFLQER    37A_5   
		    430    1107.33    2212.65    2212.13    72    AQGYEVGTSLLEIDKVEYAK    37_5   
		    358    839.17    1676.32    1675.81    99    EVVFAADDNVVGENAK    biorepCdiff_6   
		    406    1064.86    2127.70    2127.18    60    FLGEAVANPVRPFTAILGGAK    37_5   
		    245    766.11    1530.21    1529.88    38    LNGALPTIQYLISK    37_5   
		    332    745.55    1489.08    1488.72    46    MENGDVVLLENTR    biorepCdiff_5   
		    305    911.10    1820.19    1819.88    80    MTHVSTGGGASLEFLEGK    37_5   
		    311    919.10    1836.18    1835.87    58    MTHVSTGGGASLEFLEGK +Oxidation (M)    37_5   
   
      Matching Genes:  
               gi|115252230|emb|CAJ70070.1|  (phosphoglycerate kinase [Clostridium difficile 630]) 
           
  Protein Group 34   
      Expression Quality:  
         Score      Num Spectra      Num Peptides      High-Qual Peptides      % Coverage       509    20    9    6    26   
   
      Peptides:   
        Query    Observed    Mr(expt)    Mr(calc)    Score    Peptide    Result File   
		    86    561.01    1120.01    1119.58    45    FEGETLPSLK    biorepCdiffA_5   
		    14    424.25    846.48    846.44    36    ILETTDR    37A_5   
		    228    731.61    1461.20    1460.73    46    LGWTCASPEILSK    biorepCdiffA_5   
		    152    613.01    1224.01    1223.55    34    LNYSNMPEEK    biorepCdiff_5   
		    289    826.12    1650.22    1649.82    33    MIYVIPDFQNPTGR    biorepCdiffA_5   
		    93    567.83    1133.65    1133.55    50    MQGLQGSEIR +Oxidation (M)    37A_5   
		    296    896.62    1791.22    1791.87    69    QGADLQASTISQMEVSK    37_5   
		    302    905.08    1808.15    1807.86    92    QGADLQASTISQMEVSK +Oxidation (M)    37_5   
		    488    1204.36    2406.70    2406.20    104    TNVNKDDILVTSGSQQGLDFAGK    37_5   
   
      Matching Genes:  
               gi|115252729|emb|CAJ70573.1|  (putative amino acid aminotransferase [Clostridium difficile 630]) 
           
  Protein Group 35   
      Expression Quality:  
         Score      Num Spectra      Num Peptides      High-Qual Peptides      % Coverage       487    15    8    7    16   
   
      Peptides:   
        Query    Observed    Mr(expt)    Mr(calc)    Score    Peptide    Result File   
		    111    621.48    1240.95    1240.61    28    AFDYVTQEIR    biorepCdiffA_6   
		    247    531.96    1592.87    1592.78    40    AGTNMERPGPLAAHR +Oxidation (M)    37A_6   
		    90    574.32    1146.63    1146.55    48    EEGELGSGITR    37A_6   
		    685    1071.54    3211.61    3210.60    84    EEVVEETVEEAAPVSEAAVVPVSTGVAGETVK    37_6   
		    212    647.92    1293.82    1293.65    69    IDKVEFADETK    37_6   
		    516    809.07    1616.12    1615.81    103    IVLEENEQSLPMSK    37_3   
		    352    817.00    1631.98    1631.81    75    IVLEENEQSLPMSK +Oxidation (M)    37A_3   
		    96    479.81    957.60    957.51    40    NAIEGVEVK    37A_3   
   
      Matching Genes:  
               gi|115252300|emb|CAJ70141.1|  (proline reductase subunit proprotein [Clostridium difficile 630]) 
           
  Protein Group 36   
      Expression Quality:  
         Score      Num Spectra      Num Peptides      High-Qual Peptides      % Coverage       480    9    8    7    8   
   
      Peptides:   
        Query    Observed    Mr(expt)    Mr(calc)    Score    Peptide    Result File   
		    131    636.81    1271.61    1271.60    53    AQANEPLTEDGK    37A_8   
		    127    564.91    1127.80    1127.58    42    DIPNVGDSAIK    37_8   
		    181    687.13    1372.24    1371.72    77    IGAEVDSGDILVGK    biorepCdiff_8   
		    119    550.37    1098.72    1098.64    63    LISSTVIPNR    37_8   
		    278    824.06    1646.11    1645.81    60    SNSGTCINQTPIINK    biorepCdiff_8   
		    246    817.01    1632.00    1631.85    75    STGPYSLVTQQPLGGK    37A_8   
		    240    723.46    1444.90    1444.70    79    VLTDEDQEIEVR    37_8   
		    71    501.45    1000.89    1000.58    31    VTADEIIIK    biorepCdiff_8   
   
      Matching Genes:  
               gi|115249070|emb|CAJ66881.1|  (DNA-directed RNA polymerase beta chain [Clostridium difficile 630]) 
           
  Protein Group 37   
      Expression Quality:  
         Score      Num Spectra      Num Peptides      High-Qual Peptides      % Coverage       479    25    7    6    39   
   
      Peptides:   
        Query    Observed    Mr(expt)    Mr(calc)    Score    Peptide    Result File   
		    602    946.20    1890.39    1889.90    85    EAEQAGADFVGAEELVQK    biorepCdiff_3   
		    234    594.95    1187.89    1187.58    27    FDETVEAHIK    biorepCdiff_3   
		    594    936.27    1870.53    1869.94    124    FYDASEALTLVSDIAGAK    biorepCdiff_3   
		    492    790.63    1579.24    1578.83    73    LTENFTALMDAIIK    biorepCdiff_3   
		    88    512.89    1023.76    1023.52    56    SGTVTFDVAK    biorepCdiffA_3   
		    260    617.57    1233.13    1232.64    67    SITVASSMGPGVK    biorepCdiff_3   
		    151    539.48    1076.94    1076.63    47    TNIIHVPVGK    biorepCdiff_4   
   
      Matching Genes:  
               gi|115249066|emb|CAJ66877.1|  (50S ribosomal protein L1 [Clostridium difficile 630]) 
           
  Protein Group 38   
      Expression Quality:  
         Score      Num Spectra      Num Peptides      High-Qual Peptides      % Coverage       471    20    10    7    51   
   
      Peptides:   
        Query    Observed    Mr(expt)    Mr(calc)    Score    Peptide    Result File   
		    250    580.42    1158.82    1158.68    47    ALVFENVLVR    37_3   
		    485    795.14    2382.40    2382.01    25    ASDSFNLEMHVDMEEGNAAGVK +2 Oxidation (M)    37A_3   
		    665    789.75    2366.22    2366.02    63    ASDSFNLEMHVDMEEGNAAGVK +Oxidation (M)    37_3   
		    38    415.78    829.54    829.47    44    EGVIVASR    37_3   
		    144    565.84    1129.67    1129.56    52    ESGDIAGTPGVK    37A_3   
		    555    891.69    1781.37    1780.85    58    FKPLSQPGQYACEEK    biorepCdiff_3   
		    335    428.23    1281.67    1281.58    38    HIHMSNEDATK    37_3   
		    21    428.38    854.74    854.52    39    LPIALSNK    biorepCdiffA_3   
		    237    565.34    1128.67    1128.58    58    LVGPAGEVEMK    37_3   
		    148    573.36    1144.70    1144.58    47    LVGPAGEVEMK +Oxidation (M)    37A_3   
   
      Matching Genes:  
               gi|115251734|emb|CAJ69569.1|  (putative propanediol utilization protein [Clostridium difficile 630]) 
           
  Protein Group 39   
      Expression Quality:  
         Score      Num Spectra      Num Peptides      High-Qual Peptides      % Coverage       465    12    9    7    22   
   
      Peptides:   
        Query    Observed    Mr(expt)    Mr(calc)    Score    Peptide    Result File   
		    31    420.82    839.62    839.49    28    APSIPSLR    37_6   
		    226    748.95    1495.88    1495.66    27    EMGDNCSVTILNK +Oxidation (M)    37A_6   
		    304    918.13    1834.25    1833.82    60    GDDISYAGCGLPYYVGK    37A_6   
		    477    813.57    2437.70    2437.15    54    LNGELDSVLLDEIEDFDSWTK    37_8   
		    553    757.87    2270.58    2270.05    50    NSLTNKPTWSPMGSSANHEGR    37_6   
		    435    763.28    2286.82    2286.04    53    NSLTNKPTWSPMGSSANHEGR +Oxidation (M)    biorepCdiffA_6   
		    263    704.98    1407.94    1407.72    72    SSLIVNTPESFSK    37_6   
		    329    793.02    1584.03    1583.77    73    TLSTGEKENLSYDK    37_6   
		    215    731.95    1461.89    1461.82    48    VLEGGILFYPNLK    37A_6   
   
      Matching Genes:  
               gi|115250843|emb|CAJ68667.1|  (putative pyridine nucleotide-disulfide oxidoreductase [Clostridium difficile 630]) 
           
  Protein Group 40   
      Expression Quality:  
         Score      Num Spectra      Num Peptides      High-Qual Peptides      % Coverage       446    15    8    6    26   
   
      Peptides:   
        Query    Observed    Mr(expt)    Mr(calc)    Score    Peptide    Result File   
		    370    1040.55    2079.09    2078.92    75    AAEETGLPYAGFDGDQADPR    37A_5   
		    101    592.88    1183.74    1183.56    63    AFTNAQFETR    37A_5   
		    150    652.02    1302.03    1301.66    40    IQGLVEVMEER    biorepCdiffA_5   
		    48    455.77    909.53    909.46    34    KFEEVMK    37A_5   
		    165    673.94    1345.87    1345.68    51    LLIEELEDNMK    37A_5   
		    444    1118.74    2235.47    2235.02    30    RAAEETGLPYAGFDGDQADPR    37_5   
		    686    1299.99    3896.96    3896.77    65    TNFGLLENGGCEALDMPAPDFLLCCNNICNQVIK    37_5   
		    294    603.43    1807.28    1806.95    88    VVINDLLAEQYANAFK    37A_5   
   
      Matching Genes:  
               gi|115249403|emb|CAJ67218.1|  (subunit of oxygen-sensitive 2-hydroxyisocaproyl-CoA dehydratase [Clostridium difficile 630]) 
           
  Protein Group 41   
      Expression Quality:  
         Score      Num Spectra      Num Peptides      High-Qual Peptides      % Coverage       446    13    8    5    22   
   
      Peptides:   
        Query    Observed    Mr(expt)    Mr(calc)    Score    Peptide    Result File   
		    189    624.93    1247.84    1248.59    26    AAIDMVSHSFR +Oxidation (M)    37_6   
		    185    680.33    1358.64    1358.68    69    AFEGTLLAHTDGK    37A_6   
		    62    443.78    885.55    885.42    27    INFDYSK    37_6   
		    298    742.51    1483.01    1482.75    36    NMFALLGKPGYEK +Oxidation (M)    37_6   
		    113    608.30    1214.59    1214.52    44    NYDKEEFDR    37A_6   
		    411    1098.76    2195.50    2195.04    85    QLATEEGYETFVIPDDVGGR    37A_6   
		    190    625.92    1249.83    1249.63    54    SGTTTEPALAFR    37_6   
		    342    816.62    1631.23    1630.87    105    TGLGNTFLGWIDLPK    37_6   
   
      Matching Genes:  
               gi|115252341|emb|CAJ70182.1|  (glucose-6-phosphate isomerase [Clostridium difficile 630]) 
           
  Protein Group 42   
      Expression Quality:  
         Score      Num Spectra      Num Peptides      High-Qual Peptides      % Coverage       443    17    8    7    27   
   
      Peptides:   
        Query    Observed    Mr(expt)    Mr(calc)    Score    Peptide    Result File   
		    41    479.51    957.00    956.55    46    AVELLLDGK    biorepCdiffA_4   
		    190    428.88    1283.62    1283.55    32    CEEFKTEEGR    37A_4   
		    490    781.26    2340.75    2340.16    52    GLLEEDLTEMNLSSVGDIIHR    37A_4   
		    584    1179.47    2356.93    2356.16    63    GLLEEDLTEMNLSSVGDIIHR +Oxidation (M)    37_4   
		    455    913.17    1824.32    1823.96    100    HSIIVLAEGVGSASDLEK    37_4   
		    452    908.16    1814.30    1813.94    53    TIGLLTSGGDAPGMNAAIR    37_4   
		    459    916.19    1830.36    1829.93    46    TIGLLTSGGDAPGMNAAIR +Oxidation (M)    37_4   
		    85    504.85    1007.68    1007.59    51    VTVLGHVQR    37A_4   
   
      Matching Genes:  
               gi|115252455|emb|CAJ70298.1|  (6-phosphofructokinase [Clostridium difficile 630]) 
           
  Protein Group 43   
      Expression Quality:  
         Score      Num Spectra      Num Peptides      High-Qual Peptides      % Coverage       433    14    9    5    13   
   
      Peptides:   
        Query    Observed    Mr(expt)    Mr(calc)    Score    Peptide    Result File   
		    270    789.53    1577.05    1576.73    97    CGTGLASHEVAQGYK    37_8   
		    422    1179.69    2357.37    2357.08    44    ENFDQLFPADYISEGIDQTR    37A_8   
		    348    1064.05    2126.08    2125.97    29    GNTVNPMELFDQYGADALR +Oxidation (M)    37A_8   
		    360    571.43    1711.26    1710.88    49    KGDEVYYVSKPLADK    biorepCdiff_7   
		    188    456.62    1366.83    1366.72    34    LNAHETANKLEK    37A_8   
		    203    704.40    1406.79    1406.70    65    LTEELINEGYAR    37A_8   
		    266    865.66    1729.30    1728.90    54    RYDLPVIQPVDETGK    37A_8   
		    371    873.16    1744.31    1743.81    31    SLEEQNLNDHMTGIK +Oxidation (M)    biorepCdiff_7   
		    189    456.62    1366.84    1366.64    30    VVHNYPHCWR    37A_8   
   
      Matching Genes:  
               gi|115251669|emb|CAJ69504.1|  (isoleucyl-tRNA synthetase [Clostridium difficile 630]) 
           
  Protein Group 44   
      Expression Quality:  
         Score      Num Spectra      Num Peptides      High-Qual Peptides      % Coverage       431    22    6    6    30   
   
      Peptides:   
        Query    Observed    Mr(expt)    Mr(calc)    Score    Peptide    Result File   
		    375    616.54    1231.06    1230.61    44    AADDAAGLAISEK    biorepCdiff_1   
		    324    837.07    1672.12    1671.87    52    IADELTQLKDEIER    37A_4   
		    401    839.54    1677.06    1676.84    77    IRDTDVASEMVNLSK    37_4   
		    160    535.31    1068.60    1068.51    43    ISSSTEFNGK    37A_1   
		    452    1159.82    2317.62    2317.11    124    LESTQNNLNNTLENVTAAESR    biorepCdiffA_4   
		    381    910.20    1818.39    1817.88    91    TLSLQSANEINNTEER    37A_4   
   
      Matching Genes:  
               gi|115249247|emb|CAJ67060.1|  (flagellin subunit [Clostridium difficile 630]) 
           
  Protein Group 45   
      Expression Quality:  
         Score      Num Spectra      Num Peptides      High-Qual Peptides      % Coverage       422    15    8    5    24   
   
      Peptides:   
        Query    Observed    Mr(expt)    Mr(calc)    Score    Peptide    Result File   
		    396    903.16    1804.31    1803.94    72    AGFVVSDSNIKPDNTLK    37_6   
		    340    815.67    1629.32    1628.81    50    EGFLYLAEAGADFVK    biorepCdiff_6   
		    55    469.26    936.50    936.42    35    FDESPTNK    37A_6   
		    238    672.49    1342.97    1342.72    79    GQATSIIEVAQAR    37_6   
		    320    981.21    1960.40    1959.95    30    LSFEEGVDSYVPYAGSLK    biorepCdiffA_6   
		    39    443.77    885.52    885.47    77    VGAGNVVDR    37A_6   
		    503    1354.49    2706.97    2706.28    33    VPALVEAGADVLCIDSSEGFSEWQK    biorepCdiffA_6   
		    305    918.61    1835.21    1834.85    46    VSEFMTPMSSIVYANK +2 Oxidation (M)    37A_6   
   
      Matching Genes:  
               gi|115251390|emb|CAJ69222.1|  (inosine-5'-monophosphate dehydrogenase [Clostridium difficile 630]) 
           
  Protein Group 46   
      Expression Quality:  
         Score      Num Spectra      Num Peptides      High-Qual Peptides      % Coverage       418    11    8    4    18   
   
      Peptides:   
        Query    Observed    Mr(expt)    Mr(calc)    Score    Peptide    Result File   
		    338    805.58    1609.15    1608.80    87    INGVSVVSVESDNYK    37_6   
		    409    919.64    1837.27    1836.84    93    KDEFLSYINSTDYDK    37_6   
		    407    690.49    1378.97    1378.69    36    LGGISTDVPMSFR    37_3   
		    78    549.89    1097.76    1097.71    38    LILAVTDIIK    37A_6   
		    343    980.21    1958.40    1957.97    49    TLPVTYDDGTFAGIITMK +Oxidation (M)    37A_6   
		    127    620.90    1239.79    1239.65    53    VDAPEILDNVR    37A_6   
		    30    460.36    918.70    918.45    30    YALDYFK    biorepCdiffA_6   
		    24    453.36    904.70    904.38    32    YAMDMFK    biorepCdiffA_6   
   
      Matching Genes:  
               gi|115249342|emb|CAJ67155.1|  (manganese-dependent inorganic pyrophosphatase [Clostridium difficile 630]) 
           
  Protein Group 47   
      Expression Quality:  
         Score      Num Spectra      Num Peptides      High-Qual Peptides      % Coverage       418    16    8    5    45   
   
      Peptides:   
        Query    Observed    Mr(expt)    Mr(calc)    Score    Peptide    Result File   
		    600    978.18    1954.35    1953.98    101    AIANSDLGLNPSNDGEVIR    37_3   
		    118    445.75    889.49    889.43    39    ANAQMLDK    37_3   
		    75    459.76    917.51    917.51    34    EIDTLLSK    37A_3   
		    44    435.25    868.48    868.44    41    FEFGTIR    37A_3   
		    131    545.83    1089.64    1089.52    41    GGELTEDELK    37A_3   
		    271    609.88    1217.74    1217.61    69    GGELTEDELKK    37_3   
		    229    557.38    1112.75    1113.60    28    LSVPALTEER    37_3   
		    487    1194.26    2386.50    2386.22    65    VDYYGTPTPINQIGAISVPEPR    37A_3   
   
      Matching Genes:  
               gi|115251191|emb|CAJ69022.1|  (ribosome recycling factor [Clostridium difficile 630]) 
           
  Protein Group 48   
      Expression Quality:  
         Score      Num Spectra      Num Peptides      High-Qual Peptides      % Coverage       413    20    8    7    45   
   
      Peptides:   
        Query    Observed    Mr(expt)    Mr(calc)    Score    Peptide    Result File   
		    178    612.89    1223.78    1223.63    46    AGATYVSPFVGR    37A_3   
		    55    425.30    848.58    848.46    40    DFIEVVK    37_3   
		    453    722.52    2164.53    2164.13    67    EISEIVDGPISAEVISLEHK    37A_3   
		    237    720.58    1439.14    1438.73    64    FFIDTANIEEIK    biorepCdiffA_3   
		    97    480.28    958.55    958.52    49    IPMTAEGLK    37A_3   
		    135    558.35    1114.68    1114.61    40    MGADIATVPLK    37A_3   
		    232    559.90    1117.78    1117.64    36    NPIHVLQAAR    37_3   
		    552    873.61    1745.21    1744.98    71    TNVTLIFSAGQALLAAR    37_3   
   
      Matching Genes:  
               gi|115251384|emb|CAJ69216.1|  (putative transaldolase [Clostridium difficile 630]) 
           
  Protein Group 49   
      Expression Quality:  
         Score      Num Spectra      Num Peptides      High-Qual Peptides      % Coverage       402    13    7    6    50   
   
      Peptides:   
        Query    Observed    Mr(expt)    Mr(calc)    Score    Peptide    Result File   
		    184    595.37    1188.72    1188.60    42    DENIKNESIK    37A_2   
		    217    510.29    1018.57    1018.57    54    EIISSITTR    37_2   
		    427    680.38    1358.74    1358.69    80    IECQGEGLVNLK    37_2   
		    192    605.89    1209.77    1209.63    69    LSEVVEFYPK    37A_2   
		    494    734.55    1467.08    1466.87    79    LVNDVELVNVLIK    37_2   
		    394    664.86    1327.71    1327.70    48    NVPVNIWPYAR    37_2   
		    252    566.78    1131.55    1131.49    30    YECEEFKK    37_2   
   
      Matching Genes:  
               gi|115249997|emb|CAJ67817.1|  (hypothetical phage protein [Clostridium difficile 630]) 
              Other Genes Matching Peptide Subset:  
               gi|115251944|emb|CAJ69780.1|  (hypothetical phage protein [Clostridium difficile 630]) 
           
  Protein Group 50   
      Expression Quality:  
         Score      Num Spectra      Num Peptides      High-Qual Peptides      % Coverage       398    18    7    7    50   
   
      Peptides:   
        Query    Observed    Mr(expt)    Mr(calc)    Score    Peptide    Result File   
		    51    457.46    912.90    912.56    52    AVLELAGLK    biorepCdiffA_3   
		    607    637.43    1909.27    1909.01    41    FAALVVVGDENGHVGIGAGK    biorepCdiff_3   
		    738    1231.92    2461.82    2461.29    68    GHFGAGNILIMPAVEGTGVIAGGPAR    biorepCdiff_3   
		    739    1240.02    2478.03    2477.28    56    GHFGAGNILIMPAVEGTGVIAGGPAR +Oxidation (M)    biorepCdiff_3   
		    435    728.08    1454.15    1453.78    72    KPIDAGQLDLQEK    biorepCdiff_3   
		    584    929.39    1856.77    1856.09    57    NLIVVPIVGTTIPHEVR    biorepCdiff_3   
		    538    537.79    1610.35    1609.88    52    RKPIDAGQLDLQEK    biorepCdiff_2   
   
      Matching Genes:  
               gi|115249094|emb|CAJ66905.1|  (30S ribosomal protein S5 [Clostridium difficile 630]) 
           
  Protein Group 51   
      Expression Quality:  
         Score      Num Spectra      Num Peptides      High-Qual Peptides      % Coverage       396    17    7    4    41   
   
      Peptides:   
        Query    Observed    Mr(expt)    Mr(calc)    Score    Peptide    Result File   
		    340    797.14    1592.26    1591.86    105    APVSNFAYLIDAIAK    37A_3   
		    209    687.46    1372.91    1372.76    56    GLTVEEVTELRK    biorepCdiffA_3   
		    113    565.50    1128.99    1128.60    30    IVEMANIPSR    biorepCdiffA_3   
		    221    573.39    1144.77    1144.59    39    IVEMANIPSR +Oxidation (M)    biorepCdiff_3   
		    432    723.50    1444.99    1444.65    37    MGIVEGAFYDESK    biorepCdiff_3   
		    137    559.80    1117.58    1117.59    41    SEVVSEIVEK    37A_3   
		    191    520.04    1038.08    1037.54    88    SSAAVVVDYK    biorepCdiff_3   
   
      Matching Genes:  
               gi|115249067|emb|CAJ66878.1|  (50S ribosomal protein L10 [Clostridium difficile 630]) 
           
  Protein Group 52   
      Expression Quality:  
         Score      Num Spectra      Num Peptides      High-Qual Peptides      % Coverage       391    15    7    6    30   
   
      Peptides:   
        Query    Observed    Mr(expt)    Mr(calc)    Score    Peptide    Result File   
		    171    607.91    1213.81    1213.67    79    AGENLLSLLER    37A_3   
		    489    783.04    1564.07    1564.81    72    QLVTHGHFTLNGNK    biorepCdiff_3   
		    202    517.85    1033.69    1033.57    37    RLDNVVYR    37_3   
		    74    492.40    982.79    982.61    40    VDIPSLIVK    biorepCdiffA_3   
		    206    525.33    1048.64    1048.57    62    VSNYGLQLR    37_3   
		    364    675.01    1348.01    1347.65    58    WLEANVEGMTAK    biorepCdiff_3   
		    374    683.00    1363.98    1363.64    43    WLEANVEGMTAK +Oxidation (M)    biorepCdiff_3   
   
      Matching Genes:  
               gi|115249105|emb|CAJ66916.1|  (30S ribosomal protein S4 [Clostridium difficile 630]) 
           
  Protein Group 53   
      Expression Quality:  
         Score      Num Spectra      Num Peptides      High-Qual Peptides      % Coverage       383    14    7    6    68   
   
      Peptides:   
        Query    Observed    Mr(expt)    Mr(calc)    Score    Peptide    Result File   
		    1    402.70    803.38    803.40    42    ACVEVAR    37_1   
		    701    1185.92    3554.74    3553.83    68    AGNLLFVSGQVPLVPETMEVVEGDVQAQTAQSLK    37_2   
		    702    1191.26    3570.77    3569.83    78    AGNLLFVSGQVPLVPETMEVVEGDVQAQTAQSLK +Oxidation (M)    37_2   
		    709    1332.70    2663.38    2663.20    74    DMNEFGAINEVYAEYFGENKPAR    37_1   
		    764    1116.90    2231.78    2231.13    46    HEVIHTNDAPAALGPYSQAIK    biorepCdiff_1   
		    467    1246.38    2490.75    2490.27    32    MKHEVIHTNDAPAALGPYSQAIK    biorepCdiffA_1   
		    693    836.54    2506.59    2506.26    43    MKHEVIHTNDAPAALGPYSQAIK +Oxidation (M)    37_1   
   
      Matching Genes:  
               gi|115251566|emb|CAJ69399.1|  (putative translation inhibitor endoribonuclease [Clostridium difficile 630]) 
           
  Protein Group 54   
      Expression Quality:  
         Score      Num Spectra      Num Peptides      High-Qual Peptides      % Coverage       377    8    7    6    28   
   
      Peptides:   
        Query    Observed    Mr(expt)    Mr(calc)    Score    Peptide    Result File   
		    415    696.98    1391.94    1391.77    61    GYLVSNKEELLK    37_3   
		    249    579.35    1156.68    1156.56    51    ICCSYTLIK    37_3   
		    39    429.82    857.63    857.52    40    KIEEIVK    37A_3   
		    270    703.20    1404.38    1403.65    36    MDQNLNWLNEK    37A_3   
		    436    710.92    1419.83    1419.65    53    MDQNLNWLNEK +Oxidation (M)    37_3   
		    162    473.28    944.54    944.47    65    TIAAPANCK    37_3   
		    275    712.42    1422.82    1422.62    71    TTGICIDCSSPGR    37A_3   
   
      Matching Genes:  
               gi|115251115|emb|CAJ68946.1|  (conserved hypothetical protein [Clostridium difficile 630]) 
           
  Protein Group 55   
      Expression Quality:  
         Score      Num Spectra      Num Peptides      High-Qual Peptides      % Coverage       372    15    6    6    34   
   
      Peptides:   
        Query    Observed    Mr(expt)    Mr(calc)    Score    Peptide    Result File   
		    438    699.59    1397.17    1396.76    63    AEIGLTYIYGIGK    biorepCdiff_2   
		    163    458.23    914.44    914.45    44    AEINPDTR    37_2   
		    168    659.40    1316.78    1316.62    69    DLSEDQVNELR    biorepCdiffA_2   
		    238    723.50    1444.99    1444.72    45    DLSEDQVNELRK    biorepCdiffA_2   
		    25    420.86    839.70    839.49    62    IAGVDLPR    biorepCdiffA_2   
		    526    780.05    1558.08    1557.80    89    IKDLSEDQVNELR    biorepCdiff_2   
   
      Matching Genes:  
               gi|115249103|emb|CAJ66914.1|  (30S ribosomal protein S13 [Clostridium difficile 630]) 
           
  Protein Group 56   
      Expression Quality:  
         Score      Num Spectra      Num Peptides      High-Qual Peptides      % Coverage       358    16    7    4    38   
   
      Peptides:   
        Query    Observed    Mr(expt)    Mr(calc)    Score    Peptide    Result File   
		    436    670.23    2007.66    2007.14    43    GVVIPVTAVEAGPMVVTQIK    biorepCdiffA_3   
		    642    1012.89    2023.77    2023.14    33    GVVIPVTAVEAGPMVVTQIK +Oxidation (M)    biorepCdiff_3   
		    10    416.84    831.66    831.47    32    IDVTGISK    biorepCdiffA_3   
		    630    992.75    1983.49    1982.96    66    TVDKDGYNAIQIGFEDAK    biorepCdiff_3   
		    311    762.46    1522.91    1522.75    89    VDSVEGYTVGQEIK    37A_3   
		    116    570.39    1138.77    1138.57    68    VGMTQIFTDK    biorepCdiffA_3   
		    163    565.09    1128.16    1127.65    27    VTVQNLEVVK    biorepCdiff_4   
   
      Matching Genes:  
               gi|115249077|emb|CAJ66888.1|  (50S ribosomal protein L3 [Clostridium difficile 630]) 
           
  Protein Group 57   
      Expression Quality:  
         Score      Num Spectra      Num Peptides      High-Qual Peptides      % Coverage       355    9    7    5    16   
   
      Peptides:   
        Query    Observed    Mr(expt)    Mr(calc)    Score    Peptide    Result File   
		    120    615.90    1229.78    1229.60    42    HVFEGESNIAK    37A_6   
		    371    864.15    1726.29    1725.92    71    ITLPTASESFLYGISK    37_6   
		    243    788.48    1574.94    1574.86    30    KIGIEAEIVPYESK    37A_6   
		    92    579.31    1156.61    1156.51    33    NEMIESAYGK +Oxidation (M)    37A_6   
		    108    518.37    1034.73    1034.55    46    QALSYALNR    biorepCdiff_6   
		    91    480.31    958.60    958.51    48    QIQQDISK    37_6   
		    362    846.55    1691.08    1690.74    85    SNSNLQTYTYSEER    37_6   
   
      Matching Genes:  
               gi|115251723|emb|CAJ69558.1|  (oligopeptide ABC transporter, substrate-binding protein [Clostridium difficile 630]) 
           
  Protein Group 58   
      Expression Quality:  
         Score      Num Spectra      Num Peptides      High-Qual Peptides      % Coverage       352    13    6    4    34   
   
      Peptides:   
        Query    Observed    Mr(expt)    Mr(calc)    Score    Peptide    Result File   
		    343    870.67    1739.32    1738.88    105    AAALDTFETEGLFLNK    37A_4   
		    162    560.87    1119.73    1119.58    25    DYEEPVIKK    37_4   
		    405    844.11    1686.21    1685.88    71    GGLINTGDLIEALESGK    37_4   
		    536    1321.24    2640.46    2640.31    65    GLGANVIAFDQYPNSDLNDILTYK    37A_4   
		    616    1354.90    2707.79    2707.30    61    GYDGISIQQTNYIDNPYIYETLK    37_7   
		    565    973.74    2918.21    2917.34    25    WSEEHKDVQVDIYPENMTEENVVK    37A_5   
   
      Matching Genes:  
               gi|115249400|emb|CAJ67215.1|  ((R)-2-hydroxyisocaproate dehydrogenase [Clostridium difficile 630]) 
           
  Protein Group 59   
      Expression Quality:  
         Score      Num Spectra      Num Peptides      High-Qual Peptides      % Coverage       348    15    6    5    48   
   
      Peptides:   
        Query    Observed    Mr(expt)    Mr(calc)    Score    Peptide    Result File   
		    247    556.34    1110.66    1110.64    51    ADLDLRPALK    37_2   
		    355    823.55    1645.08    1644.81    60    ALENYFNYETLIR    37A_2   
		    251    564.78    1127.55    1127.54    43    ANVQYYGTGR    37_2   
		    252    656.47    1310.93    1310.73    108    LVAGEGNILVNGR    37A_2   
		    123    606.93    1211.84    1211.65    37    QPLVLTGNENK    biorepCdiffA_2   
		    376    965.73    1929.45    1929.06    49    QPLVLTGNENKYDVIVK    biorepCdiffA_2   
   
      Matching Genes:  
               gi|115249113|emb|CAJ66924.1|  (30S ribosomal protein S9 [Clostridium difficile 630]) 
           
  Protein Group 60   
      Expression Quality:  
         Score      Num Spectra      Num Peptides      High-Qual Peptides      % Coverage       339    21    7    6    40   
   
      Peptides:   
        Query    Observed    Mr(expt)    Mr(calc)    Score    Peptide    Result File   
		    483    733.18    1464.34    1463.75    41    FENGTEITAELLK    biorepCdiff_2   
		    193    486.85    971.69    971.53    46    ILGEGNLEK    biorepCdiff_2   
		    378    440.29    1317.84    1318.74    45    LHELKPAEGAVR    37_2   
		    536    532.38    1594.11    1593.87    30    MKLHELKPAEGAVR +Oxidation (M)    biorepCdiff_2   
		    225    631.39    1260.76    1260.63    70    VGFEGGQMPLAR    37A_2   
		    320    639.47    1276.93    1276.62    51    VGFEGGQMPLAR +Oxidation (M)    biorepCdiff_2   
		    234    718.06    1434.11    1433.75    56    VYTEVNVEVLNR    biorepCdiffA_2   
   
      Matching Genes:  
               gi|115249096|emb|CAJ66907.1|  (50S ribosomal protein L15 [Clostridium difficile 630]) 
           
  Protein Group 61   
      Expression Quality:  
         Score      Num Spectra      Num Peptides      High-Qual Peptides      % Coverage       336    13    7    5    31   
   
      Peptides:   
        Query    Observed    Mr(expt)    Mr(calc)    Score    Peptide    Result File   
		    77    494.49    986.97    986.51    32    EVAPALMEK    biorepCdiffA_3   
		    235    598.51    1195.01    1194.63    45    GMDIIFVTTAK    biorepCdiff_3   
		    381    929.78    1857.55    1856.99    60    GVEELEMISGQKPVITK    biorepCdiffA_3   
		    596    937.80    1873.58    1872.99    50    GVEELEMISGQKPVITK +Oxidation (M)    biorepCdiff_3   
		    120    579.95    1157.89    1157.62    63    IVINMGIGDAR    biorepCdiffA_3   
		    124    587.98    1173.94    1173.62    39    IVINMGIGDAR +Oxidation (M)    biorepCdiffA_3   
		    31    435.92    869.82    869.53    47    LVSVSLPR    biorepCdiffA_3   
   
      Matching Genes:  
               gi|115249089|emb|CAJ66900.1|  (50S ribosomal protein L5 [Clostridium difficile 630]) 
           
  Protein Group 62   
      Expression Quality:  
         Score      Num Spectra      Num Peptides      High-Qual Peptides      % Coverage       336    15    6    5    52   
   
      Peptides:   
        Query    Observed    Mr(expt)    Mr(calc)    Score    Peptide    Result File   
		    412    664.33    1326.65    1326.62    76    HETVDVPASNMK    37_1   
		    105    545.46    1088.91    1088.62    46    ILLEEGFIR    biorepCdiffA_1   
		    274    777.10    1552.19    1551.74    29    KENVGGEVICYVW    biorepCdiffA_2   
		    191    683.02    1364.02    1363.65    53    TMTDPIADMLTR    biorepCdiffA_1   
		    200    694.55    1387.09    1386.81    72    VLNGLGISVISTSK    biorepCdiffA_1   
		    186    571.43    1140.84    1140.59    60    VYAANHEIPK    37A_1   
   
      Matching Genes:  
               gi|115249091|emb|CAJ66902.1|  (30S ribosomal protein S8 [Clostridium difficile 630]) 
           
  Protein Group 63   
      Expression Quality:  
         Score      Num Spectra      Num Peptides      High-Qual Peptides      % Coverage       335    19    8    4    22   
   
      Peptides:   
        Query    Observed    Mr(expt)    Mr(calc)    Score    Peptide    Result File   
		    234    669.44    1336.86    1336.61    59    DTDPQSALEYAK    37_6   
		    476    1041.81    2081.60    2080.97    25    EVQASMDEIGVGEFATVSGR    37_6   
		    517    940.17    2817.49    2816.35    28    IVELGGSAIITADHGNAEYMLDPETGK +Oxidation (M)    37A_6   
		    368    572.40    1714.17    1713.86    47    LEKPEEMTGHSLISK +Oxidation (M)    37_6   
		    472    1037.77    2073.53    2073.00    66    VATYDLKPEMSAYELTDK    37_6   
		    482    1045.73    2089.44    2088.99    39    VATYDLKPEMSAYELTDK +Oxidation (M)    37_6   
		    81    583.46    1164.91    1164.60    30    VELAYNAMVR    biorepCdiffA_6   
		    91    575.77    1149.52    1149.52    41    VLCEAMDNAK    37A_6   
   
      Matching Genes:  
               gi|115252228|emb|CAJ70068.1|  (2,3-bisphosphoglycerate-independent phosphoglycerate mutase [Clostridium difficile 630]) 
           
  Protein Group 64   
      Expression Quality:  
         Score      Num Spectra      Num Peptides      High-Qual Peptides      % Coverage       319    11    6    4    54   
   
      Peptides:   
        Query    Observed    Mr(expt)    Mr(calc)    Score    Peptide    Result File   
		    383    652.87    1303.72    1303.63    75    EGASKEEADQIK    37_1   
		    115    436.23    870.44    870.44    37    EVVDNAPK    37_1   
		    412    1073.31    2144.60    2144.06    39    FGVSASAPVMVAGAAAGGPAAEEK    biorepCdiffA_1   
		    475    721.26    2160.76    2160.05    55    FGVSASAPVMVAGAAAGGPAAEEK +Oxidation (M)    37A_1   
		    602    749.20    1496.39    1495.74    67    TEFDVVLTDVGSSK    biorepCdiff_1   
		    92    528.89    1055.76    1055.62    46    VLELNELVK    biorepCdiffA_1   
   
      Matching Genes:  
               gi|115249068|emb|CAJ66879.1|  (50S ribosomal protein L7/L12 [Clostridium difficile 630]) 
           
  Protein Group 65   
      Expression Quality:  
         Score      Num Spectra      Num Peptides      High-Qual Peptides      % Coverage       319    12    6    5    17   
   
      Peptides:   
        Query    Observed    Mr(expt)    Mr(calc)    Score    Peptide    Result File   
		    146    581.90    1161.79    1161.59    53    FPLDTEAELK    37_6   
		    365    1047.36    2092.70    2092.14    48    HVENITQVYGLPAVVAINR    biorepCdiffA_6   
		    581    1206.32    2410.64    2410.15    50    IYGADGVDYTPEADKEIANLEK    37_6   
		    440    969.73    1937.46    1936.99    65    LGNIIVGYSYEGEPVTAR    37_6   
		    246    857.14    1712.27    1711.87    65    SDIEIAQEAKPQDIR    biorepCdiffA_6   
		    214    648.44    1294.86    1294.68    38    TTTTIGVADAFAK    37_6   
   
      Matching Genes:  
               gi|115249735|emb|CAJ67552.1|  (formate--tetrahydrofolate ligase [Clostridium difficile 630]) 
           
  Protein Group 66   
      Expression Quality:  
         Score      Num Spectra      Num Peptides      High-Qual Peptides      % Coverage       317    12    7    4    12   
   
      Peptides:   
        Query    Observed    Mr(expt)    Mr(calc)    Score    Peptide    Result File   
		    127    572.97    1143.93    1143.57    32    APESLIEEEK    biorepCdiff_7   
		    348    543.37    1627.10    1626.77    41    IKEHNHNVGTCYR    37_7   
		    109    490.80    979.59    979.46    59    LYGEDANAK    37_7   
		    522    1122.87    2243.73    2243.14    77    SLGNGIDPLEIIEQYGADALR    37_7   
		    457    824.53    2470.58    2470.10    33    TTLSDAEVEHEEHDGNFYHIK    37A_7   
		    237    668.52    1335.02    1334.70    43    TYLQWLENIR    37_7   
		    491    1338.56    2675.11    2675.09    32    YNEEDNMAEEEDMMNLLMEGIR    biorepCdiffA_7   
   
      Matching Genes:  
               gi|115252312|emb|CAJ70153.1|  (valyl-tRNA synthetase [Clostridium difficile 630]) 
           
  Protein Group 67   
      Expression Quality:  
         Score      Num Spectra      Num Peptides      High-Qual Peptides      % Coverage       304    11    6    2    15   
   
      Peptides:   
        Query    Observed    Mr(expt)    Mr(calc)    Score    Peptide    Result File   
		    307    638.89    1913.64    1912.98    35    AIGDNLTCIFVDHGLLR    biorepCdiffA_6   
		    315    774.49    1546.97    1546.66    73    AVTSSDGMTSDWYK    37_6   
		    65    445.82    889.63    889.50    38    FDINLIR    37_6   
		    507    1084.31    2166.61    2166.08    98    GIIFTGGPNSAYLEDSPTISK    37_6   
		    88    488.87    975.73    975.54    30    IIGEEFIR    biorepCdiff_6   
		    101    490.28    978.55    978.44    30    TVGVMGDER +Oxidation (M)    37_6   
   
      Matching Genes:  
               gi|115249206|emb|CAJ67019.1|  (GMP synthase [glutamine-hydrolyzing] [Clostridium difficile 630]) 
           
  Protein Group 68   
      Expression Quality:  
         Score      Num Spectra      Num Peptides      High-Qual Peptides      % Coverage       304    7    5    3    23   
   
      Peptides:   
        Query    Observed    Mr(expt)    Mr(calc)    Score    Peptide    Result File   
		    372    901.63    1801.24    1801.01    92    ILFVAGGVGSAPVYPQVK    37A_4   
		    75    588.95    1175.90    1175.62    48    IPLTIADYDR    biorepCdiffA_4   
		    354    882.98    1763.94    1763.78    98    NVYVSTDDGTYGFNGR    37A_4   
		    436    593.90    1778.67    1777.89    31    QLTDSIYLMEIEAPR    biorepCdiff_4   
		    134    609.37    1216.72    1216.68    35    SSQPGQFIIIK    37A_4   
   
      Matching Genes:  
               gi|115250577|emb|CAJ68401.1|  (putative dehydrogenase, electron transfer subunit [Clostridium difficile 630]) 
           
  Protein Group 69   
      Expression Quality:  
         Score      Num Spectra      Num Peptides      High-Qual Peptides      % Coverage       297    7    6    5    22   
   
      Peptides:   
        Query    Observed    Mr(expt)    Mr(calc)    Score    Peptide    Result File   
		    59    501.97    1001.93    1001.55    51    FVIEPLER    biorepCdiffA_5   
		    252    707.49    1412.97    1412.74    81    LEELGLGLKPSEE    37A_4   
		    71    473.78    945.54    944.52    44    SLEEVIQK    37A_4   
		    539    1085.76    2169.50    2169.16    46    TENVPIGVLPVDSIYTPVEK    37_4   
		    167    669.64    1337.27    1336.65    28    VDIVELSEDYR    biorepCdiffA_5   
		    106    552.79    1103.57    1103.54    47    VSYHVENTR    37A_4   
   
      Matching Genes:  
               gi|115249106|emb|CAJ66917.1|  (DNA-directed RNA polymerase alpha chain [Clostridium difficile 630]) 
           
  Protein Group 70   
      Expression Quality:  
         Score      Num Spectra      Num Peptides      High-Qual Peptides      % Coverage       295    9    6    5    31   
   
      Peptides:   
        Query    Observed    Mr(expt)    Mr(calc)    Score    Peptide    Result File   
		    651    904.08    2709.23    2708.25    42    KTEGLQDTQTHYCPGCTHGIIHR    biorepCdiff_4   
		    679    976.91    2927.70    2926.57    40    LVGEVLEELGVLGDAVGVVPVGCSVLGYK    biorepCdiff_4   
		    245    574.83    1147.66    1147.56    53    NAETQGYPIR    37_3   
		    161    473.27    944.53    944.48    35    NVEVEEVK    37_3   
		    497    861.42    2581.23    2580.16    64    TEGLQDTQTHYCPGCTHGIIHR    biorepCdiffA_4   
		    211    532.84    1063.66    1063.58    61    VAVDTPAHVR    37_3   
   
      Matching Genes:  
               gi|115249126|emb|CAJ66937.1|  (putative subunit of oxidoreductase [Clostridium difficile 630]) 
           
  Protein Group 71   
      Expression Quality:  
         Score      Num Spectra      Num Peptides      High-Qual Peptides      % Coverage       284    14    6    6    28   
   
      Peptides:   
        Query    Observed    Mr(expt)    Mr(calc)    Score    Peptide    Result File   
		    469    756.62    1511.23    1510.87    51    ALVVIADKNDNVIK    biorepCdiff_3   
		    243    723.47    1444.92    1444.77    43    EFAQILNNINAAK    biorepCdiffA_3   
		    700    1118.32    2234.62    2234.16    57    NIEGVQTALVNTMNVYDILK    biorepCdiff_3   
		    505    1126.40    2250.79    2250.16    49    NIEGVQTALVNTMNVYDILK +Oxidation (M)    biorepCdiffA_3   
		    328    838.19    1674.37    1673.91    44    TKEFAQILNNINAAK    biorepCdiffA_3   
		    208    687.05    1372.08    1371.69    40    YDSFIITTDAVK    biorepCdiffA_3   
   
      Matching Genes:  
               gi|115249078|emb|CAJ66889.1|  (50S ribosomal protein L4 [Clostridium difficile 630]) 
           
  Protein Group 72   
      Expression Quality:  
         Score      Num Spectra      Num Peptides      High-Qual Peptides      % Coverage       280    8    5    5    15   
   
      Peptides:   
        Query    Observed    Mr(expt)    Mr(calc)    Score    Peptide    Result File   
		    123    618.38    1234.75    1234.64    70    GKETVISTADSK    37_5   
		    55    465.31    928.61    928.56    56    IGIEGSILK    37A_5   
		    199    646.11    1290.21    1289.70    53    ILVLNCGSSSLK    biorepCdiff_5   
		    244    822.59    1643.17    1642.88    54    MLLIPTNEELMIAR    37A_5   
		    245    827.47    1652.92    1651.75    47    YGFHGTSHNYVSQR    37A_5   
   
      Matching Genes:  
               gi|115250207|emb|CAJ68028.1|  (acetate kinase [Clostridium difficile 630]) 
           
  Protein Group 73   
      Expression Quality:  
         Score      Num Spectra      Num Peptides      High-Qual Peptides      % Coverage       280    10    6    5    21   
   
      Peptides:   
        Query    Observed    Mr(expt)    Mr(calc)    Score    Peptide    Result File   
		    348    864.64    1727.26    1726.88    50    EGITSVGENKPQELAR    biorepCdiffA_3   
		    106    494.36    986.70    986.60    37    EITLLAVTK    37A_3   
		    586    934.13    1866.24    1865.85    60    GLMTMAPFIEDEDEIR    37_3   
		    418    950.12    1898.22    1897.84    42    GLMTMAPFIEDEDEIR +2 Oxidation (M)    37A_3   
		    592    942.14    1882.26    1881.85    47    GLMTMAPFIEDEDEIR +Oxidation (M)    37_3   
		    172    483.28    964.55    964.50    44    VGTSIFGER    37_3   
   
      Matching Genes:  
               gi|115251674|emb|CAJ69509.1|  (putative alanine racemase [Clostridium difficile 630]) 
           
  Protein Group 74   
      Expression Quality:  
         Score      Num Spectra      Num Peptides      High-Qual Peptides      % Coverage       276    12    5    4    39   
   
      Peptides:   
        Query    Observed    Mr(expt)    Mr(calc)    Score    Peptide    Result File   
		    336    734.07    1466.12    1465.78    63    AGSQVSGPVPLPTEK    37A_1   
		    274    798.09    1594.17    1593.87    72    KAGSQVSGPVPLPTEK    biorepCdiffA_1   
		    164    541.43    1080.84    1080.62    67    LIDIANPTPK    37A_1   
		    25    425.91    849.80    849.46    41    LLDFSAGK    biorepCdiffA_1   
		    11    415.17    828.33    827.52    33    QVVTILR    biorepCdiffA_1   
   
      Matching Genes:  
               gi|115249076|emb|CAJ66887.1|  (30S ribosomal protein S10 [Clostridium difficile 630]) 
           
  Protein Group 75   
      Expression Quality:  
         Score      Num Spectra      Num Peptides      High-Qual Peptides      % Coverage       275    10    5    4    14   
   
      Peptides:   
        Query    Observed    Mr(expt)    Mr(calc)    Score    Peptide    Result File   
		    169    613.46    1224.90    1224.65    71    DAVGQSVHTIAK    37_6   
		    327    788.06    1574.10    1574.66    53    EFECYTQEQVDK    37_6   
		    134    422.58    1264.73    1264.65    31    GHSVSIHSNTVK    37A_6   
		    98    586.35    1170.68    1170.61    43    IAGVEIPEDTK    37A_6   
		    390    890.96    1779.91    1780.95    77    IIVIEADGPGEEDIIAK    37_6   
   
      Matching Genes:  
               gi|115251397|emb|CAJ69229.1|  (succinate-semialdehyde dehydrogenase [NAD(P)+] [Clostridium difficile 630]) 
           
  Protein Group 76   
      Expression Quality:  
         Score      Num Spectra      Num Peptides      High-Qual Peptides      % Coverage       262    14    5    3    28   
   
      Peptides:   
        Query    Observed    Mr(expt)    Mr(calc)    Score    Peptide    Result File   
		    481    776.00    1549.98    1549.72    39    EVSWLPSYGPEMR    37_3   
		    489    784.00    1565.99    1565.72    33    EVSWLPSYGPEMR +Oxidation (M)    37_3   
		    221    538.36    1074.70    1074.57    67    FKDDVIPGGK    37_3   
		    128    493.85    985.68    985.62    59    IIVNSSLIK    37A_2   
		    477    770.90    2309.69    2309.11    64    TDVDVYYIPANELAAELGNDK    37A_3   
   
      Matching Genes:  
               gi|115249127|emb|CAJ66938.1|  (putative subunit of oxidoreductase [Clostridium difficile 630]) 
           
  Protein Group 77   
      Expression Quality:  
         Score      Num Spectra      Num Peptides      High-Qual Peptides      % Coverage       261    6    5    4    23   
   
      Peptides:   
        Query    Observed    Mr(expt)    Mr(calc)    Score    Peptide    Result File   
		    172    578.84    1155.66    1155.52    66    FFEGDIEGSR    37_4   
		    116    573.40    1144.78    1144.59    39    HFETIANSVK    37A_4   
		    214    628.04    1254.07    1253.75    46    LPIILYNVPGR    biorepCdiff_4   
		    366    761.21    1520.41    1519.78    61    TAMNLLGFNVGDLR    biorepCdiff_4   
		    509    824.28    2469.81    2469.18    49    VPVIAGSGSNDTMHSVNLSQEAEK    37A_4   
   
      Matching Genes:  
               gi|115252282|emb|CAJ70123.1|  (dihydrodipicolinate synthase [Clostridium difficile 630]) 
           
  Protein Group 78   
      Expression Quality:  
         Score      Num Spectra      Num Peptides      High-Qual Peptides      % Coverage       257    11    5    4    34   
   
      Peptides:   
        Query    Observed    Mr(expt)    Mr(calc)    Score    Peptide    Result File   
		    640    703.25    2106.72    2105.99    80    MPDLNAASVEAAMSMIAGTAR    biorepCdiff_2   
		    652    713.98    2138.92    2137.98    48    MPDLNAASVEAAMSMIAGTAR +2 Oxidation (M)    biorepCdiff_2   
		    634    1045.88    2089.74    2089.09    43    TADQAGMIIPVVITVYQDR    biorepCdiff_2   
		    637    1053.88    2105.74    2105.08    33    TADQAGMIIPVVITVYQDR +Oxidation (M)    biorepCdiff_2   
		    161    455.30    908.59    908.57    53    TPPAAVLIK    37_2   
   
      Matching Genes:  
               gi|115249065|emb|CAJ66876.1|  (50S ribosomal protein L11 [Clostridium difficile 630]) 
           
  Protein Group 79   
      Expression Quality:  
         Score      Num Spectra      Num Peptides      High-Qual Peptides      % Coverage       255    8    4    4    46   
   
      Peptides:   
        Query    Observed    Mr(expt)    Mr(calc)    Score    Peptide    Result File   
		    243    515.26    1028.51    1028.55    70    IANGEIPSTK    37_1   
		    276    567.26    1132.51    1132.55    52    KLPNYEAGQN    37_1   
		    340    738.99    1475.97    1475.67    87    VINNCGSDGGQEVK    37A_1   
		    483    740.55    2218.63    2218.25    46    VLAFNDLNPVAPYHILVVPK    37A_1   
   
      Matching Genes:  
               gi|115251501|emb|CAJ69334.1|  (histidine triad nucleotide-binding protein [Clostridium difficile 630]) 
           
  Protein Group 80   
      Expression Quality:  
         Score      Num Spectra      Num Peptides      High-Qual Peptides      % Coverage       254    9    5    4    14   
   
      Peptides:   
        Query    Observed    Mr(expt)    Mr(calc)    Score    Peptide    Result File   
		    367    1035.24    2068.47    2067.97    68    GGPGLGSIQPSQADYFMSTR    37A_5   
		    386    1043.17    2084.32    2083.96    33    GGPGLGSIQPSQADYFMSTR +Oxidation (M)    37_5   
		    124    632.50    1262.98    1262.65    63    VMTSSSSPGVALK    biorepCdiffA_5   
		    208    640.41    1278.80    1278.65    41    VMTSSSSPGVALK +Oxidation (M)    37_7   
		    442    1154.31    2306.61    2306.13    49    YFFGYPITPQSELPEYLSR    37A_5   
   
      Matching Genes:  
               gi|115249125|emb|CAJ66936.1|  (putative oxidoreductase, thiamine diP-binding subunit [Clostridium difficile 630]) 
           
  Protein Group 81   
      Expression Quality:  
         Score      Num Spectra      Num Peptides      High-Qual Peptides      % Coverage       252    11    5    4    18   
   
      Peptides:   
        Query    Observed    Mr(expt)    Mr(calc)    Score    Peptide    Result File   
		    155    661.40    1320.79    1320.66    33    AVQLHGGYGYTR    37A_5   
		    197    733.49    1464.96    1464.73    48    DLGKPYGVEAAMAK +Oxidation (M)    37A_5   
		    97    578.86    1155.71    1155.64    57    HLVYQAAINK    37A_5   
		    344    647.18    1938.53    1938.08    65    IGIAAQALGLAQGALDETVK    37_5   
		    214    763.00    1523.99    1523.75    49    ITEIYEGTSEVQR    37A_5   
   
      Matching Genes:  
               gi|115250075|emb|CAJ67895.1|  (butyryl-CoA dehydrogenase [Clostridium difficile 630]) 
           
  Protein Group 82   
      Expression Quality:  
         Score      Num Spectra      Num Peptides      High-Qual Peptides      % Coverage       252    16    5    4    23   
   
      Peptides:   
        Query    Observed    Mr(expt)    Mr(calc)    Score    Peptide    Result File   
		    162    634.47    1266.93    1266.73    50    ALVPVVVEQTGR    biorepCdiffA_3   
		    111    563.42    1124.83    1124.52    34    DNFMSALEAK    biorepCdiffA_3   
		    239    602.43    1202.85    1202.61    45    ETLNEILSER    biorepCdiff_3   
		    356    657.40    1312.78    1312.66    58    EYGLIDEVFTK    37_3   
		    441    482.31    1443.92    1443.79    65    IKETLNEILSER    37_3   
   
      Matching Genes:  
               gi|115252361|emb|CAJ70202.1|  (ATP-dependent Clp protease proteolytic subunit [Clostridium difficile 630]) 
           
  Protein Group 83   
      Expression Quality:  
         Score      Num Spectra      Num Peptides      High-Qual Peptides      % Coverage       244    11    5    3    51   
   
      Peptides:   
        Query    Observed    Mr(expt)    Mr(calc)    Score    Peptide    Result File   
		    476    713.94    1425.87    1426.66    67    AHNDANMLSLGER    37_2   
		    262    591.84    1181.67    1181.57    27    CAVVSDVFSAK    37_2   
		    236    541.31    1080.61    1080.57    58    EIISYLESK    37_2   
		    771    1159.86    3476.55    3475.61    60    GIECVDYGTNNATDSVDYPVYGEIVANSVINK    biorepCdiff_2   
		    297    702.33    1402.65    1402.67    32    IGLGCDHGGYNLK    37A_2   
   
      Matching Genes:  
               gi|115252540|emb|CAJ70383.1|  (ribose-5-phosphate isomerase 2 [Clostridium difficile 630]) 
           
  Protein Group 84   
      Expression Quality:  
         Score      Num Spectra      Num Peptides      High-Qual Peptides      % Coverage       243    9    6    2    23   
   
      Peptides:   
        Query    Observed    Mr(expt)    Mr(calc)    Score    Peptide    Result File   
		    336    962.17    1922.33    1921.92    69    AEGIEATEEEFKAELEK    37A_6   
		    406    1092.27    2182.52    2182.05    52    ELDIDPIDNPDLDIEEISK    37A_6   
		    492    873.10    2616.28    2616.20    25    ELSALDDEFAKDTSEFDSLDELK    37A_6   
		    676    1312.88    2623.74    2624.31    35    GENYNLVIGSNTFIPGFEEQLVGK    biorepCdiff_6   
		    56    525.48    1048.94    1049.58    30    IVIETQYGK    biorepCdiffA_6   
		    14    433.93    865.85    865.44    32    YNIPGFR    biorepCdiffA_6   
   
      Matching Genes:  
               gi|115252362|emb|CAJ70203.1|  (trigger factor [Clostridium difficile 630]) 
           
  Protein Group 85   
      Expression Quality:  
         Score      Num Spectra      Num Peptides      High-Qual Peptides      % Coverage       239    8    5    3    27   
   
      Peptides:   
        Query    Observed    Mr(expt)    Mr(calc)    Score    Peptide    Result File   
		    93    437.77    873.53    873.48    28    AVIIDESK    37_3   
		    77    460.33    918.64    918.52    41    FEILEIR    37A_3   
		    79    465.79    929.57    929.48    49    ISNESPVGK    37A_3   
		    112    501.81    1001.61    1001.58    83    KAVIIDESK    37A_3   
		    456    696.24    2085.69    2084.99    38    VAISFGDLSENAEYDEAKK    biorepCdiffA_3   
   
      Matching Genes:  
               gi|115252616|emb|CAJ70459.1|  (transcription elongation factor [Clostridium difficile 630]) 
           
  Protein Group 86   
      Expression Quality:  
         Score      Num Spectra      Num Peptides      High-Qual Peptides      % Coverage       235    8    3    3    24   
   
      Peptides:   
        Query    Observed    Mr(expt)    Mr(calc)    Score    Peptide    Result File   
		    429    706.95    1411.88    1411.68    83    QNFGQVSNSYIR    37_3   
		    639    1081.71    2161.41    2160.97    82    TGEGDGDDEEILVNLETMPK    37_3   
		    564    887.59    1773.17    1772.86    70    VEKDEDFIFYNNLK    37_3   
   
      Matching Genes:  
               gi|115250676|emb|CAJ68500.1|  (tellurium resistance protein [Clostridium difficile 630]) 
           
  Protein Group 87   
      Expression Quality:  
         Score      Num Spectra      Num Peptides      High-Qual Peptides      % Coverage       232    9    5    4    31   
   
      Peptides:   
        Query    Observed    Mr(expt)    Mr(calc)    Score    Peptide    Result File   
		    255    573.83    1145.65    1145.58    58    ETEGEIEVLK    37_2   
		    598    1038.65    2075.29    2075.03    48    EYLPQQLSEEELEEIVK    37_2   
		    96    458.34    914.66    914.59    29    KSVVTLIR    37A_2   
		    191    605.88    1209.74    1209.59    56    STISEVGATSMK    37A_2   
		    283    613.83    1225.65    1225.59    41    STISEVGATSMK +Oxidation (M)    37_2   
   
      Matching Genes:  
               gi|115251499|emb|CAJ69332.1|  (putative tRNA binding protein [Clostridium difficile 630]) 
           
  Protein Group 88   
      Expression Quality:  
         Score      Num Spectra      Num Peptides      High-Qual Peptides      % Coverage       231    6    5    2    8   
   
      Peptides:   
        Query    Observed    Mr(expt)    Mr(calc)    Score    Peptide    Result File   
		    511    1072.96    2143.91    2143.00    35    ADLVTGMVGEFDELQGFMGK    biorepCdiff_7   
		    245    674.43    1346.85    1346.69    74    AESDKIDESLLK    37_7   
		    30    430.95    859.89    859.48    50    FAGDILPK    biorepCdiff_7   
		    117    558.50    1114.98    1114.58    39    LADALFFYR    biorepCdiff_7   
		    113    498.33    994.65    994.50    33    NLESYIEK    37_7   
   
      Matching Genes:  
               gi|115251485|emb|CAJ69318.1|  (glycyl-tRNA synthetase beta chain [Clostridium difficile 630]) 
           
  Protein Group 89   
      Expression Quality:  
         Score      Num Spectra      Num Peptides      High-Qual Peptides      % Coverage       228    7    4    3    11   
   
      Peptides:   
        Query    Observed    Mr(expt)    Mr(calc)    Score    Peptide    Result File   
		    296    899.69    1797.36    1796.95    32    AMDLLVPGVGEIVGGSQR    37A_6   
		    138    638.90    1275.78    1275.64    45    ITTMDLNDIPK +Oxidation (M)    37A_6   
		    100    489.35    976.69    976.53    55    IVNSEFIR    37_6   
		    282    869.55    1737.09    1736.85    96    SIVVEGESDSSYPLQK    37A_6   
   
      Matching Genes:  
               gi|115251299|emb|CAJ69130.1|  (asparaginyl-tRNA synthetase [Clostridium difficile 630]) 
           
  Protein Group 90   
      Expression Quality:  
         Score      Num Spectra      Num Peptides      High-Qual Peptides      % Coverage       224    15    4    4    52   
   
      Peptides:   
        Query    Observed    Mr(expt)    Mr(calc)    Score    Peptide    Result File   
		    614    1018.15    2034.29    2034.06    56    EQPQIAEVVEVGPGGIVEGK    37_1   
		    130    610.94    1219.87    1220.64    65    IEGQEYTILR    biorepCdiffA_2   
		    180    454.71    907.40    907.43    42    MELTVGDK +Oxidation (M)    37_1   
		    165    542.87    1083.72    1083.63    61    TASGIVLPGAAK    37A_1   
   
      Matching Genes:  
               gi|115249203|emb|CAJ67015.1|  (10 kDa chaperonin [Clostridium difficile 630]) 
           
  Protein Group 91   
      Expression Quality:  
         Score      Num Spectra      Num Peptides      High-Qual Peptides      % Coverage       220    7    4    3    37   
   
      Peptides:   
        Query    Observed    Mr(expt)    Mr(calc)    Score    Peptide    Result File   
		    510    1031.34    3091.01    3091.55    62    KGEFFCSEAVLQTINDALGQPLSPEITK    biorepCdiffA_2   
		    227    530.34    1058.67    1058.61    56    LASGFPIGLGK    37_2   
		    226    529.28    1056.55    1056.49    34    NISEEYFR    37_2   
		    421    677.39    1352.76    1352.55    68    WDGDNFMSPER    37_2   
   
      Matching Genes:  
               gi|115252075|emb|CAJ69912.1|  (conserved hypothetical protein [Clostridium difficile 630]) 
           
  Protein Group 92   
      Expression Quality:  
         Score      Num Spectra      Num Peptides      High-Qual Peptides      % Coverage       220    7    4    4    10   
   
      Peptides:   
        Query    Observed    Mr(expt)    Mr(calc)    Score    Peptide    Result File   
		    319    777.56    1553.11    1552.73    51    EAYPGDVFYLHSR    37_6   
		    180    676.45    1350.88    1350.79    61    IVEVPVGEALIGR    37A_6   
		    233    770.51    1539.01    1538.81    49    TRPVESEAPGIIDR    37A_6   
		    170    613.91    1225.80    1225.67    59    VVNSLGQPIDGK    37_6   
   
      Matching Genes:  
               gi|115252530|emb|CAJ70373.1|  (ATP synthase alpha chain [Clostridium difficile 630]) 
           
  Protein Group 93   
      Expression Quality:  
         Score      Num Spectra      Num Peptides      High-Qual Peptides      % Coverage       215    9    4    3    20   
   
      Peptides:   
        Query    Observed    Mr(expt)    Mr(calc)    Score    Peptide    Result File   
		    595    962.14    1922.26    1922.00    73    FPEYAAEVLSTVVEQIK    37_3   
		    166    476.31    950.61    951.59    44    LPVVKPGSR    37_3   
		    325    780.53    1559.04    1558.78    64    TNHDIGMPIYSAIK    37A_3   
		    495    788.52    1575.02    1574.78    34    TNHDIGMPIYSAIK +Oxidation (M)    37_3   
   
      Matching Genes:  
               gi|115249197|emb|CAJ67009.1|  (orotate phosphoribosyltransferase [Clostridium difficile 630]) 
           
  Protein Group 94   
      Expression Quality:  
         Score      Num Spectra      Num Peptides      High-Qual Peptides      % Coverage       212    6    5    2    11   
   
      Peptides:   
        Query    Observed    Mr(expt)    Mr(calc)    Score    Peptide    Result File   
		    212    534.76    1601.26    1600.80    30    DLEEYLHLLEEAK    biorepCdiffA_7   
		    585    1242.40    2482.79    2482.19    49    EALEELGLPYTINEGDGAFYGPK    37_7   
		    544    1125.33    2248.64    2248.13    36    ELGLFMIPEEGPGFPMFLPK    biorepCdiff_7   
		    88    551.80    1101.59    1101.54    65    EVADNNVSVR    37A_7   
		    33    418.82    835.63    835.46    32    GPHLPSTK    37_7   
   
      Matching Genes:  
               gi|115249589|emb|CAJ67406.1|  (threonyl-tRNA synthetase [Clostridium difficile 630]) 
           
  Protein Group 95   
      Expression Quality:  
         Score      Num Spectra      Num Peptides      High-Qual Peptides      % Coverage       211    4    3    3    14   
   
      Peptides:   
        Query    Observed    Mr(expt)    Mr(calc)    Score    Peptide    Result File   
		    399    937.30    1872.59    1872.01    79    EGLLNEDVILESITSIK    37A_4   
		    543    730.91    2189.72    2189.16    60    VNVEDLIYPLFVVEGENIK    37_4   
		    153    619.41    1236.81    1236.56    72    YASNYYGPFR    37A_4   
   
      Matching Genes:  
               gi|115252479|emb|CAJ70322.1|  (delta-aminolevulinic acid dehydratase [Clostridium difficile 630]) 
           
  Protein Group 96   
      Expression Quality:  
         Score      Num Spectra      Num Peptides      High-Qual Peptides      % Coverage       208    6    3    2    28   
   
      Peptides:   
        Query    Observed    Mr(expt)    Mr(calc)    Score    Peptide    Result File   
		    412    905.24    1808.46    1807.91    91    SIMGIMSLGLAQGEELK +2 Oxidation (M)    37A_1   
		    582    896.58    1791.14    1791.91    86    SIMGIMSLGLAQGEELK +Oxidation (M)    37_1   
		    37    416.83    831.65    831.47    31    STVEVVAK    37A_1   
   
      Matching Genes:  
               gi|115251809|emb|CAJ69644.1|  (PTS system, phosphocarrier protein [Clostridium difficile 630]) 
           
  Protein Group 97   
      Expression Quality:  
         Score      Num Spectra      Num Peptides      High-Qual Peptides      % Coverage       207    7    5    3    19   
   
      Peptides:   
        Query    Observed    Mr(expt)    Mr(calc)    Score    Peptide    Result File   
		    263    654.86    1307.71    1307.60    57    AATPDVNSENYK    37_4   
		    50    522.93    1043.85    1043.55    43    ALEAAEELAK    biorepCdiffA_4   
		    231    638.52    1275.02    1274.65    44    HYGLTTEDIVK    biorepCdiff_4   
		    189    609.97    1217.92    1217.64    35    NSVAYPNLNVK    37_4   
		    368    897.10    1792.19    1791.95    28    QAILASIDYNGPVYIR    37A_4   
   
      Matching Genes:  
               gi|115251376|emb|CAJ69208.1|  (transketolase [Clostridium difficile 630]) 
           
  Protein Group 98   
      Expression Quality:  
         Score      Num Spectra      Num Peptides      High-Qual Peptides      % Coverage       205    8    4    3    19   
   
      Peptides:   
        Query    Observed    Mr(expt)    Mr(calc)    Score    Peptide    Result File   
		    292    903.77    1805.53    1804.98    34    ATIGQVGNIEHGNVVIGK    biorepCdiffA_4   
		    146    531.98    1061.95    1061.55    51    SAGVSAQLMAK    biorepCdiff_4   
		    252    825.13    1648.24    1647.86    70    TANIALLNYADGEKR    biorepCdiffA_4   
		    176    589.42    1176.82    1176.58    50    VATIEYDPNR    biorepCdiff_4   
   
      Matching Genes:  
               gi|115249080|emb|CAJ66891.1|  (50S ribosomal protein L2 [Clostridium difficile 630]) 
           
  Protein Group 99   
      Expression Quality:  
         Score      Num Spectra      Num Peptides      High-Qual Peptides      % Coverage       193    10    4    2    31   
   
      Peptides:   
        Query    Observed    Mr(expt)    Mr(calc)    Score    Peptide    Result File   
		    179    583.37    1164.72    1164.60    47    EQALVEVSYK    37A_2   
		    490    730.49    1458.96    1458.78    88    ILGGGLPYESAVQR    37_2   
		    457    469.92    1406.73    1406.73    33    VKDALEMLSHHK    37_2   
		    146    530.25    1058.48    1058.60    25    VKVETGIDAK    37A_2   
   
      Matching Genes:  
               gi|115249807|emb|CAJ67624.1|  (putative NUDIX-family hydrolase [Clostridium difficile 630]) 
           
  Protein Group 100   
      Expression Quality:  
         Score      Num Spectra      Num Peptides      High-Qual Peptides      % Coverage       192    6    4    2    37   
   
      Peptides:   
        Query    Observed    Mr(expt)    Mr(calc)    Score    Peptide    Result File   
		    322    584.39    1750.16    1749.89    35    HQKPSAMNQQGGIINK    biorepCdiffA_1   
		    577    589.68    1766.01    1765.89    43    HQKPSAMNQQGGIINK +Oxidation (M)    37_1   
		    166    543.91    1085.81    1085.64    36    KGDTVVVIAGK    37A_1   
		    190    585.99    1169.96    1169.70    78    VLVEGVNVITK    37A_1   
   
      Matching Genes:  
               gi|115249088|emb|CAJ66899.1|  (50S ribosomal protein L24 [Clostridium difficile 630]) 
           
  Protein Group 101   
      Expression Quality:  
         Score      Num Spectra      Num Peptides      High-Qual Peptides      % Coverage       183    9    4    2    40   
   
      Peptides:   
        Query    Observed    Mr(expt)    Mr(calc)    Score    Peptide    Result File   
		    465    798.62    2392.85    2392.22    34    HKPTFTPHVDGGDFVVVVNAEK    biorepCdiffA_2   
		    344    861.69    1721.37    1720.92    82    KPEEVISHAVSGMLPK    biorepCdiffA_3   
		    190    604.87    1207.72    1207.62    27    KWYLVDAEGK    37A_2   
		    229    543.98    1085.95    1085.64    40    LATEIATVLR    biorepCdiff_2   
   
      Matching Genes:  
               gi|115249112|emb|CAJ66923.1|  (50S ribosomal protein L13 [Clostridium difficile 630]) 
           
  Protein Group 102   
      Expression Quality:  
         Score      Num Spectra      Num Peptides      High-Qual Peptides      % Coverage       181    6    4    3    24   
   
      Peptides:   
        Query    Observed    Mr(expt)    Mr(calc)    Score    Peptide    Result File   
		    413    845.12    1688.22    1687.71    47    ALDACEDKEDTMYK    biorepCdiff_4   
		    199    619.55    1237.08    1236.59    48    FGVEEIEAESK    biorepCdiff_4   
		    500    976.18    1950.34    1949.87    60    TDEKEVDDENVTDINSK    biorepCdiff_4   
		    59    458.27    914.53    914.54    26    VVRPSMVK    37A_4   
   
      Matching Genes:  
               gi|115251516|emb|CAJ69349.1|  (heat shock protein [Clostridium difficile 630]) 
           
  Protein Group 103   
      Expression Quality:  
         Score      Num Spectra      Num Peptides      High-Qual Peptides      % Coverage       180    7    4    3    12   
   
      Peptides:   
        Query    Observed    Mr(expt)    Mr(calc)    Score    Peptide    Result File   
		    334    726.00    1449.98    1449.75    47    GFFPEEELITLR    37_4   
		    169    418.96    1253.86    1253.81    49    KVTGKPTVIIAK    37A_4   
		    149    539.81    1077.61    1077.46    49    MNVCAENPK +Oxidation (M)    37_4   
		    166    563.90    1125.78    1125.71    35    VTGKPTVIIAK    37_4   
   
      Matching Genes:  
               gi|115251377|emb|CAJ69209.1|  (transketolase [Clostridium difficile 630]) 
           
  Protein Group 104   
      Expression Quality:  
         Score      Num Spectra      Num Peptides      High-Qual Peptides      % Coverage       179    9    3    3    17   
   
      Peptides:   
        Query    Observed    Mr(expt)    Mr(calc)    Score    Peptide    Result File   
		    321    775.45    1548.88    1548.81    82    ELSDIIGFTASQIR    37A_3   
		    503    793.06    1584.10    1583.83    57    GVWNFAPLDLEVPK    37_3   
		    151    482.86    963.70    963.50    40    YLGDLLDR    biorepCdiff_3   
   
      Matching Genes:  
               gi|115249180|emb|CAJ66992.1|  (putative DNA-binding protein [Clostridium difficile 630]) 
           
  Protein Group 105   
      Expression Quality:  
         Score      Num Spectra      Num Peptides      High-Qual Peptides      % Coverage       174    8    3    2    29   
   
      Peptides:   
        Query    Observed    Mr(expt)    Mr(calc)    Score    Peptide    Result File   
		    337    738.12    1474.23    1473.79    85    IGVIGGGSITYPNAR    37A_1   
		    114    572.40    1142.79    1142.52    52    LSETDEFFR    biorepCdiffA_1   
		    138    478.30    954.59    954.47    37    TYSEGAISK    37A_1   
   
      Matching Genes:  
               gi|115251040|emb|CAJ68871.1|  (putative decarboxylase [Clostridium difficile 630]) 
           
  Protein Group 106   
      Expression Quality:  
         Score      Num Spectra      Num Peptides      High-Qual Peptides      % Coverage       174    5    4    1    30   
   
      Peptides:   
        Query    Observed    Mr(expt)    Mr(calc)    Score    Peptide    Result File   
		    514    1094.33    2186.65    2186.10    36    ELDNLEEGVVVTIVDNETAK    37_6   
		    355    822.59    1643.16    1642.86    73    GFIYTLTESKPYPK    37A_3   
		    594    955.67    1909.32    1909.02    32    LQVGTVTNMYTIVETLK    37_3   
		    187    517.47    1032.92    1032.49    33    MGLGNDELGK    biorepCdiff_3   
   
      Matching Genes:  
               gi|115252732|emb|CAJ70576.1|  (conserved hypothetical protein [Clostridium difficile 630]) 
           
  Protein Group 107   
      Expression Quality:  
         Score      Num Spectra      Num Peptides      High-Qual Peptides      % Coverage       173    4    3    3    11   
   
      Peptides:   
        Query    Observed    Mr(expt)    Mr(calc)    Score    Peptide    Result File   
		    689    1029.57    3085.68    3085.44    60    EFYPDASEFYLSDGYDLVNALTGSTIAK    biorepCdiff_4   
		    303    686.50    1370.99    1371.70    60    GEADTISAAPVSVR    37_4   
		    123    591.81    1181.61    1181.59    53    LGSISDSVYNK    37A_4   
   
      Matching Genes:  
               gi|115251246|emb|CAJ69077.1|  (cell surface protein [Clostridium difficile 630]) 
           
  Protein Group 108   
      Expression Quality:  
         Score      Num Spectra      Num Peptides      High-Qual Peptides      % Coverage       172    5    4    2    27   
   
      Peptides:   
        Query    Observed    Mr(expt)    Mr(calc)    Score    Peptide    Result File   
		    672    1065.99    2129.97    2129.14    34    AATVETGAVVQVPLFINTGDK    biorepCdiff_3   
		    149    466.29    930.56    930.48    54    ENEVATIR    37_3   
		    326    783.02    1564.02    1563.75    30    TGAIREESFNPSEK    37A_3   
		    26    413.24    824.47    824.40    54    TGEYLSR    37_3   
   
      Matching Genes:  
               gi|115250279|emb|CAJ68101.1|  (elongation factor P [Clostridium difficile 630]) 
           
  Protein Group 109   
      Expression Quality:  
         Score      Num Spectra      Num Peptides      High-Qual Peptides      % Coverage       168    6    3    2    27   
   
      Peptides:   
        Query    Observed    Mr(expt)    Mr(calc)    Score    Peptide    Result File   
		    340    858.26    1714.51    1713.88    69    APAFEGNNMVVIIDPK    biorepCdiffA_3   
		    463    749.57    1497.12    1496.79    39    LRPGIEQNDLNTK    biorepCdiff_3   
		    390    940.33    1878.64    1877.97    60    NLDLVQISPNANPPVCK    biorepCdiffA_3   
   
      Matching Genes:  
               gi|115249701|emb|CAJ67518.1|  (translation initiation factor IF-3 [Clostridium difficile 630]) 
           
  Protein Group 110   
      Expression Quality:  
         Score      Num Spectra      Num Peptides      High-Qual Peptides      % Coverage       167    4    3    3    18   
   
      Peptides:   
        Query    Observed    Mr(expt)    Mr(calc)    Score    Peptide    Result File   
		    133    555.82    1109.62    1109.61    50    IAYLFEAVGK    37A_3   
		    116    515.31    1028.61    1028.55    52    LLENLNEGK    37A_3   
		    386    676.91    1351.80    1351.62    65    NLMAGFAGESEAR    37_3   
   
      Matching Genes:  
               gi|115249842|emb|CAJ67659.1|  (rubrerythrin [Clostridium difficile 630]) 
           
  Protein Group 111   
      Expression Quality:  
         Score      Num Spectra      Num Peptides      High-Qual Peptides      % Coverage       166    4    3    2    6   
   
      Peptides:   
        Query    Observed    Mr(expt)    Mr(calc)    Score    Peptide    Result File   
		    203    731.47    1460.93    1460.76    59    DANANIVLNNLYK    37A_7   
		    547    1158.86    2315.72    2315.23    28    IATLIPIDGNDENEYLLLATK    37_7   
		    213    644.91    1287.81    1287.67    79    TNDEVNVVSIAK    37_7   
   
      Matching Genes:  
               gi|115249009|emb|CAJ66820.1|  (DNA gyrase subunit A [Clostridium difficile 630]) 
           
  Protein Group 112   
      Expression Quality:  
         Score      Num Spectra      Num Peptides      High-Qual Peptides      % Coverage       166    6    2    2    15   
   
      Peptides:   
        Query    Observed    Mr(expt)    Mr(calc)    Score    Peptide    Result File   
		    429    706.95    1411.88    1411.68    83    QNFGQVSNSYIR    37_3   
		    566    889.08    1776.14    1775.81    83    TGEGDGDDEQIVVDLSK    37_3   
   
      Matching Genes:  
               gi|115250675|emb|CAJ68499.1|  (tellurium resistance protein [Clostridium difficile 630]) 
           
  Protein Group 113   
      Expression Quality:  
         Score      Num Spectra      Num Peptides      High-Qual Peptides      % Coverage       165    6    3    2    4   
   
      Peptides:   
        Query    Observed    Mr(expt)    Mr(calc)    Score    Peptide    Result File   
		    289    887.26    1772.51    1771.92    47    EDLPVLLPTDVEFTGK    37A_7   
		    1    402.25    802.49    802.41    31    VALEDEK    37_7   
		    324    771.95    1541.88    1541.66    87    YVDSNNENEPFSK    37_7   
   
      Matching Genes:  
               gi|115251575|emb|CAJ69408.1|  (leucyl-tRNA synthetase [Clostridium difficile 630]) 
           
  Protein Group 114   
      Expression Quality:  
         Score      Num Spectra      Num Peptides      High-Qual Peptides      % Coverage       164    6    3    3    6   
   
      Peptides:   
        Query    Observed    Mr(expt)    Mr(calc)    Score    Peptide    Result File   
		    163    602.43    1202.84    1202.63    51    AAQLVEMLGAGK +Oxidation (M)    37_7   
		    289    721.51    1441.00    1440.76    52    GVVDVYPNKPEPK    37_7   
		    421    962.18    1922.34    1921.94    61    SLGLAGVMGGANSEITSNTK +Oxidation (M)    37_7   
   
      Matching Genes:  
               gi|115249716|emb|CAJ67533.1|  (phenylalanyl-tRNA synthetase beta chain [Clostridium difficile 630]) 
           
  Protein Group 115   
      Expression Quality:  
         Score      Num Spectra      Num Peptides      High-Qual Peptides      % Coverage       164    6    3    2    7   
   
      Peptides:   
        Query    Observed    Mr(expt)    Mr(calc)    Score    Peptide    Result File   
		    83    462.33    922.64    922.49    30    FPYEIVR    37_6   
		    248    694.43    1386.84    1386.70    76    KLEEGADVDLGNK    biorepCdiff_6   
		    284    486.29    1455.84    1455.65    58    TESMHGAGSPQAQR    37_6   
   
      Matching Genes:  
               gi|115251396|emb|CAJ69228.1|  (gamma-aminobutyrate metabolism dehydratase/isomerase [includes: 4-hydroxybutyryl-coa dehydratase; vinylacetyl-coa-delta-isomerase] [Clostridium difficile 630]) 
           
  Protein Group 116   
      Expression Quality:  
         Score      Num Spectra      Num Peptides      High-Qual Peptides      % Coverage       163    5    4    1    15   
   
      Peptides:   
        Query    Observed    Mr(expt)    Mr(calc)    Score    Peptide    Result File   
		    105    535.50    1068.99    1068.59    26    FTAEVYVLK    biorepCdiff_5   
		    353    590.32    1767.94    1767.78    34    HYAHVDCPGHADYVK    biorepCdiffA_3   
		    296    604.05    1809.14    1808.96    37    TKPHVNIGTIGHVDHGK    37A_5   
		    417    1083.27    2164.52    2164.04    66    YQLGEAVDFANIDKAPEER    37_5   
   
      Matching Genes:  
               gi|115249061|emb|CAJ66872.1|  (elongation factor TU [Clostridium difficile 630]) 
              Other Genes Matching Peptide Subset:  
               gi|115249075|emb|CAJ66886.1|  (elongation factor TU [Clostridium difficile 630]) 
           
  Protein Group 117   
      Expression Quality:  
         Score      Num Spectra      Num Peptides      High-Qual Peptides      % Coverage       163    8    3    3    42   
   
      Peptides:   
        Query    Observed    Mr(expt)    Mr(calc)    Score    Peptide    Result File   
		    382    1002.33    2002.64    2002.00    72    LEANAGDVVTLNEVLACSK    biorepCdiffA_1   
		    183    671.61    1341.20    1340.73    50    LGSPVVEGASVQAK    biorepCdiffA_1   
		    273    611.46    1220.91    1220.63    41    VSEGDVLFVEK    biorepCdiff_2   
   
      Matching Genes:  
               gi|115250193|emb|CAJ68014.1|  (50S ribosomal protein L21 [Clostridium difficile 630]) 
           
  Protein Group 118   
      Expression Quality:  
         Score      Num Spectra      Num Peptides      High-Qual Peptides      % Coverage       160    4    3    2    16   
   
      Peptides:   
        Query    Observed    Mr(expt)    Mr(calc)    Score    Peptide    Result File   
		    497    1003.17    2004.33    2003.92    70    DVYACCTHGVLSGPAIER    37_4   
		    119    579.26    1156.51    1156.54    33    MIFSNESVSK +Oxidation (M)    37A_4   
		    611    796.05    2385.12    2384.26    57    NVILLDDMIDTAGTIVNAANALK    biorepCdiff_4   
   
      Matching Genes:  
               gi|115252575|emb|CAJ70418.1|  (ribose-phosphate pyrophosphokinase [Clostridium difficile 630]) 
           
  Protein Group 119   
      Expression Quality:  
         Score      Num Spectra      Num Peptides      High-Qual Peptides      % Coverage       159    5    2    2    39   
   
      Peptides:   
        Query    Observed    Mr(expt)    Mr(calc)    Score    Peptide    Result File   
		    119    456.34    910.66    910.50    65    GPQAANVVR    37A_1   
		    351    919.23    1836.44    1835.88    94    TLEEGQSVEFEVVDGAK    biorepCdiffA_1   
   
      Matching Genes:  
               gi|115250391|emb|CAJ68213.1|  (putative cold shock protein [Clostridium difficile 630]) 
           
  Protein Group 120   
      Expression Quality:  
         Score      Num Spectra      Num Peptides      High-Qual Peptides      % Coverage       158    7    3    2    38   
   
      Peptides:   
        Query    Observed    Mr(expt)    Mr(calc)    Score    Peptide    Result File   
		    389    1024.75    2047.49    2046.82    46    DECDNSYIYTFYTNDK    biorepCdiffA_1   
		    676    907.39    1812.76    1812.85    25    EEFEELKNNTYIER    biorepCdiff_1   
		    393    863.67    1725.32    1724.79    87    IVDEYDYGYNAIYK    37A_1   
   
      Matching Genes:  
               gi|115249824|emb|CAJ67641.1|  (hypothetical protein [Clostridium difficile 630]) 
           
  Protein Group 121   
      Expression Quality:  
         Score      Num Spectra      Num Peptides      High-Qual Peptides      % Coverage       157    6    3    2    26   
   
      Peptides:   
        Query    Observed    Mr(expt)    Mr(calc)    Score    Peptide    Result File   
		    281    580.29    1158.58    1158.62    51    KAGQICDLVR    37_1   
		    103    543.50    1084.98    1084.61    69    NVDEALAILK    biorepCdiffA_1   
		    285    506.38    1010.74    1010.58    37    TSHIEVVVK    biorepCdiff_1   
   
      Matching Genes:  
               gi|115249082|emb|CAJ66893.1|  (50S ribosomal protein L22 [Clostridium difficile 630]) 
           
  Protein Group 122   
      Expression Quality:  
         Score      Num Spectra      Num Peptides      High-Qual Peptides      % Coverage       152    6    4    1    50   
   
      Peptides:   
        Query    Observed    Mr(expt)    Mr(calc)    Score    Peptide    Result File   
		    73    478.40    954.79    954.55    39    KYTFVVAK    biorepCdiffA_1   
		    500    908.01    2721.00    2720.31    31    MTNPHDVIIRPVVTEHSMAEMGEK    biorepCdiffA_1   
		    75    426.01    850.00    849.46    36    VFGVSVDK    biorepCdiff_1   
		    241    512.24    1022.46    1022.50    46    VNTLNYDGK    37_1   
   
      Matching Genes:  
               gi|115249079|emb|CAJ66890.1|  (50S ribosomal protein L23 [Clostridium difficile 630]) 
           
  Protein Group 123   
      Expression Quality:  
         Score      Num Spectra      Num Peptides      High-Qual Peptides      % Coverage       152    4    2    2    11   
   
      Peptides:   
        Query    Observed    Mr(expt)    Mr(calc)    Score    Peptide    Result File   
		    426    869.15    1736.28    1735.76    69    ADIDYGFAEADTTYGK    biorepCdiff_4   
		    397    832.57    1663.13    1662.82    83    TEGYSEGNVPLQTLR    37_4   
   
      Matching Genes:  
               gi|115249083|emb|CAJ66894.1|  (30S ribosomal protein S3 [Clostridium difficile 630]) 
           
  Protein Group 124   
      Expression Quality:  
         Score      Num Spectra      Num Peptides      High-Qual Peptides      % Coverage       152    6    3    2    22   
   
      Peptides:   
        Query    Observed    Mr(expt)    Mr(calc)    Score    Peptide    Result File   
		    369    670.54    1339.06    1338.71    31    LYRPANTFVMK    biorepCdiff_2   
		    309    834.06    1666.12    1665.77    57    MLSEMAIQDPEGFAK    biorepCdiffA_2   
		    318    842.12    1682.24    1681.77    64    MLSEMAIQDPEGFAK +Oxidation (M)    biorepCdiffA_2   
   
      Matching Genes:  
               gi|115249703|emb|CAJ67520.1|  (50S ribosomal protein L20 [Clostridium difficile 630]) 
           
  Protein Group 125   
      Expression Quality:  
         Score      Num Spectra      Num Peptides      High-Qual Peptides      % Coverage       152    7    3    1    20   
   
      Peptides:   
        Query    Observed    Mr(expt)    Mr(calc)    Score    Peptide    Result File   
		    84    507.92    1013.82    1013.49    30    FWEEYLK    biorepCdiffA_3   
		    344    534.00    1598.97    1598.72    31    NLSDNHESQEAEVK    37A_3   
		    448    739.10    1476.19    1475.76    91    VEAGIYNEILNNK    biorepCdiff_3   
   
      Matching Genes:  
               gi|115252635|emb|CAJ70478.1|  (putative preprotein translocase [Clostridium difficile 630]) 
           
  Protein Group 126   
      Expression Quality:  
         Score      Num Spectra      Num Peptides      High-Qual Peptides      % Coverage       150    7    3    3    6   
   
      Peptides:   
        Query    Observed    Mr(expt)    Mr(calc)    Score    Peptide    Result File   
		    256    699.02    1396.02    1395.74    49    AGEDQQPIILANK    37_6   
		    330    777.01    1552.01    1551.69    54    HNAPTEPDNSGSAAGK    37_7   
		    330    800.57    1599.13    1598.76    47    TYVSAYHSTNLSEK    biorepCdiff_6   
   
      Matching Genes:  
               gi|115251837|emb|CAJ69672.1|  (cell surface protein [Clostridium difficile 630]) 
           
  Protein Group 127   
      Expression Quality:  
         Score      Num Spectra      Num Peptides      High-Qual Peptides      % Coverage       149    4    4    1    6   
   
      Peptides:   
        Query    Observed    Mr(expt)    Mr(calc)    Score    Peptide    Result File   
		    407    625.84    1874.51    1873.98    29    DGSLVGAILINDLSCTVK    biorepCdiff_7   
		    295    916.04    1830.07    1829.79    28    TLVTCDSFGSHYSNDK    37A_7   
		    140    607.46    1212.90    1212.65    34    VNLFDVIEHK    biorepCdiff_7   
		    50    459.37    916.72    916.52    58    VVASETAIK    37A_7   
   
      Matching Genes:  
               gi|115250664|emb|CAJ68488.1|  (putative nitric oxide reductase flavoprotein [Clostridium difficile 630]) 
           
  Protein Group 128   
      Expression Quality:  
         Score      Num Spectra      Num Peptides      High-Qual Peptides      % Coverage       149    4    3    1    12   
   
      Peptides:   
        Query    Observed    Mr(expt)    Mr(calc)    Score    Peptide    Result File   
		    263    727.52    1453.02    1452.71    88    ALLDAVNTGDYSSK    37A_4   
		    229    630.38    1258.75    1258.57    36    HMPFVEENEK    37_4   
		    177    685.97    1369.92    1370.65    25    RISYMSEVDQK +Oxidation (M)    37A_7   
   
      Matching Genes:  
               gi|115252096|emb|CAJ69934.1|  (proline iminopeptidase [Clostridium difficile 630]) 
           
  Protein Group 129   
      Expression Quality:  
         Score      Num Spectra      Num Peptides      High-Qual Peptides      % Coverage       147    7    3    2    9   
   
      Peptides:   
        Query    Observed    Mr(expt)    Mr(calc)    Score    Peptide    Result File   
		    139    520.35    1038.69    1038.62    32    AEAIVKPGVR    37_4   
		    64    517.89    1033.76    1033.53    40    ATVSGELTEK    biorepCdiffA_5   
		    174    680.98    1359.95    1359.78    75    FLLNLIELGGGSK    37A_5   
   
      Matching Genes:  
               gi|115251664|emb|CAJ69499.1|  (probable peptidase [Clostridium difficile 630]) 
           
  Protein Group 130   
      Expression Quality:  
         Score      Num Spectra      Num Peptides      High-Qual Peptides      % Coverage       141    9    3    2    27   
   
      Peptides:   
        Query    Observed    Mr(expt)    Mr(calc)    Score    Peptide    Result File   
		    215    518.44    1034.86    1034.54    34    GITEVVFDR    biorepCdiff_2   
		    143    493.80    985.58    985.52    47    IQELAEGAR    37A_1   
		    570    574.82    1721.42    1720.87    60    SANNIYAQIIDDTKR    biorepCdiff_2   
   
      Matching Genes:  
               gi|115249093|emb|CAJ66904.1|  (50S ribosomal protein L18 [Clostridium difficile 630]) 
           
  Protein Group 131   
      Expression Quality:  
         Score      Num Spectra      Num Peptides      High-Qual Peptides      % Coverage       136    6    3    1    44   
   
      Peptides:   
        Query    Observed    Mr(expt)    Mr(calc)    Score    Peptide    Result File   
		    120    590.43    1178.84    1178.55    37    DLTSEELMNK    biorepCdiffA_1   
		    231    724.99    1447.96    1447.74    67    FQLATGQLENTAR    biorepCdiffA_1   
		    26    426.41    850.80    850.45    32    SELFSLR    biorepCdiffA_1   
   
      Matching Genes:  
               gi|115249085|emb|CAJ66896.1|  (50S ribosomal protein L29 [Clostridium difficile 630]) 
           
  Protein Group 132   
      Expression Quality:  
         Score      Num Spectra      Num Peptides      High-Qual Peptides      % Coverage       135    3    2    2    6   
   
      Peptides:   
        Query    Observed    Mr(expt)    Mr(calc)    Score    Peptide    Result File   
		    298    909.14    1816.26    1815.97    84    ILVINNPSNPTGSVYTK    37A_5   
		    57    453.03    904.04    903.51    51    YDLVPGLK    biorepCdiff_5   
   
      Matching Genes:  
               gi|115249115|emb|CAJ66926.1|  (aspartate aminotransferase [Clostridium difficile 630]) 
           
  Protein Group 133   
      Expression Quality:  
         Score      Num Spectra      Num Peptides      High-Qual Peptides      % Coverage       135    7    3    2    26   
   
      Peptides:   
        Query    Observed    Mr(expt)    Mr(calc)    Score    Peptide    Result File   
		    188    479.73    957.44    957.51    38    AIEQEQLK    37_2   
		    242    566.42    1130.83    1130.67    50    IQVFEGVVLK    biorepCdiff_2   
		    402    687.44    1372.86    1372.66    47    NEVPNFGPGDTVK    biorepCdiff_2   
   
      Matching Genes:  
               gi|115250291|emb|CAJ68113.1|  (50S ribosomal protein L19 [Clostridium difficile 630]) 
           
  Protein Group 134   
      Expression Quality:  
         Score      Num Spectra      Num Peptides      High-Qual Peptides      % Coverage       135    10    2    1    21   
   
      Peptides:   
        Query    Observed    Mr(expt)    Mr(calc)    Score    Peptide    Result File   
		    361    935.32    1868.62    1868.91    102    LAGEGGLFFVDQEFANR    biorepCdiffA_1   
		    590    669.07    2004.20    2004.03    33    VTALINNLAAVANSESDFR    37_2   
   
      Matching Genes:  
               gi|115251248|emb|CAJ69079.1|  (ferritin [Clostridium difficile 630]) 
           
  Protein Group 135   
      Expression Quality:  
         Score      Num Spectra      Num Peptides      High-Qual Peptides      % Coverage       135    3    2    2    9   
   
      Peptides:   
        Query    Observed    Mr(expt)    Mr(calc)    Score    Peptide    Result File   
		    374    869.15    1736.28    1735.90    79    VTTAFNDTTIADGIAVK    37_6   
		    427    1103.74    2205.47    2205.04    56    YSFSTIMEDKPGNFAELTR    37_5   
   
      Matching Genes:  
               gi|115251567|emb|CAJ69400.1|  (threonine dehydratase catabolic [Clostridium difficile 630]) 
           
  Protein Group 136   
      Expression Quality:  
         Score      Num Spectra      Num Peptides      High-Qual Peptides      % Coverage       135    7    2    2    12   
   
      Peptides:   
        Query    Observed    Mr(expt)    Mr(calc)    Score    Peptide    Result File   
		    304    748.00    1493.99    1493.85    63    AGVVLNPATPVDTIK    37A_3   
		    186    616.90    1231.79    1231.68    72    LAPSILSADFAK    37A_3   
   
      Matching Genes:  
               gi|115251631|emb|CAJ69464.1|  (ribulose-phosphate 3-epimerase [Clostridium difficile 630]) 
           
  Protein Group 137   
      Expression Quality:  
         Score      Num Spectra      Num Peptides      High-Qual Peptides      % Coverage       130    3    2    2    20   
   
      Peptides:   
        Query    Observed    Mr(expt)    Mr(calc)    Score    Peptide    Result File   
		    508    881.67    2641.98    2641.24    61    LPGEVESYEYGLEYGTDTLEIHK    37A_3   
		    325    633.39    1264.77    1264.67    69    LSNYDVNSLIK    37_2   
   
      Matching Genes:  
               gi|115251797|emb|CAJ69632.1|  (adenine phosphoribosyltransferase [Clostridium difficile 630]) 
           
  Protein Group 138   
      Expression Quality:  
         Score      Num Spectra      Num Peptides      High-Qual Peptides      % Coverage       124    3    3    1    26   
   
      Peptides:   
        Query    Observed    Mr(expt)    Mr(calc)    Score    Peptide    Result File   
		    809    1179.86    3536.55    3535.76    39    LVMNLGFSHPVEMEDPEGITVEAPNQTELIVK    biorepCdiff_3   
		    85    508.38    1014.74    1014.57    50    QLSAELNIK    biorepCdiffA_3   
		    40    447.38    892.74    892.47    35    YVDEVIR    biorepCdiffA_3   
   
      Matching Genes:  
               gi|115249092|emb|CAJ66903.1|  (50S ribosomal protein L6 [Clostridium difficile 630]) 
           
  Protein Group 139   
      Expression Quality:  
         Score      Num Spectra      Num Peptides      High-Qual Peptides      % Coverage       124    3    2    2    12   
   
      Peptides:   
        Query    Observed    Mr(expt)    Mr(calc)    Score    Peptide    Result File   
		    483    778.56    1555.11    1554.83    74    DVIDAVSISLNAPNK    37_3   
		    197    508.34    1014.67    1013.58    50    INELIDVAK    37_3   
   
      Matching Genes:  
               gi|115249398|emb|CAJ67213.1|  (Radical SAM-superfamily protein [Clostridium difficile 630]) 
           
  Protein Group 140   
      Expression Quality:  
         Score      Num Spectra      Num Peptides      High-Qual Peptides      % Coverage       124    4    2    2    15   
   
      Peptides:   
        Query    Observed    Mr(expt)    Mr(calc)    Score    Peptide    Result File   
		    258    584.87    1167.73    1167.58    56    ESVDFPIYAK    37_2   
		    219    624.36    1246.71    1246.60    68    GIEVEDVGTNSK    37A_2   
   
      Matching Genes:  
               gi|115251375|emb|CAJ69207.1|  (ribose-5-phosphate isomerase 1 [Clostridium difficile 630]) 
           
  Protein Group 141   
      Expression Quality:  
         Score      Num Spectra      Num Peptides      High-Qual Peptides      % Coverage       120    4    3    1    4   
   
      Peptides:   
        Query    Observed    Mr(expt)    Mr(calc)    Score    Peptide    Result File   
		    45    497.84    993.67    994.47    34    ENMLWFR    biorepCdiffA_7   
		    559    1195.37    2388.72    2388.17    56    LAPSLTLGCGSWGGNSVSENVGVK    37_7   
		    99    511.54    1021.07    1020.57    30    LSPVLAMYK    biorepCdiff_7   
   
      Matching Genes:  
               gi|115249343|emb|CAJ67156.1|  (aldehyde-alcohol dehydrogenase [includes: alcohol dehydrogenase; acetaldehyde dehydrogenase [acetylating]; pyruvate-formate-lyase deactivase [Clostridium difficile 630]) 
           
  Protein Group 142   
      Expression Quality:  
         Score      Num Spectra      Num Peptides      High-Qual Peptides      % Coverage       120    3    2    2    10   
   
      Peptides:   
        Query    Observed    Mr(expt)    Mr(calc)    Score    Peptide    Result File   
		    351    436.97    1307.89    1307.69    50    KENIAIQEAHR    37_3   
		    329    696.69    1391.37    1390.68    70    MEEEGVFNVLPK    biorepCdiff_4   
   
      Matching Genes:  
               gi|115251194|emb|CAJ69025.1|  (30S ribosomal protein S2 [Clostridium difficile 630]) 
           
  Protein Group 143   
      Expression Quality:  
         Score      Num Spectra      Num Peptides      High-Qual Peptides      % Coverage       119    6    3    2    20   
   
      Peptides:   
        Query    Observed    Mr(expt)    Mr(calc)    Score    Peptide    Result File   
		    495    491.57    1471.69    1470.80    45    IYSSLYLEDLKK    37_2   
		    5    409.77    817.53    817.42    48    LSDGLGEK    37A_2   
		    159    648.45    1294.89    1294.63    26    YLIDITEEGDK    biorepCdiffA_2   
   
      Matching Genes:  
               gi|115250371|emb|CAJ68193.1|  (MarR-family transcriptional regulator [Clostridium difficile 630]) 
           
  Protein Group 144   
      Expression Quality:  
         Score      Num Spectra      Num Peptides      High-Qual Peptides      % Coverage       114    3    2    2    10   
   
      Peptides:   
        Query    Observed    Mr(expt)    Mr(calc)    Score    Peptide    Result File   
		    251    580.92    1159.82    1159.67    48    MIIFPAIDIK    37_3   
		    535    844.05    1686.08    1685.83    66    VNVYYDNPLEVAYK    37_3   
   
      Matching Genes:  
               gi|115250593|emb|CAJ68417.1|  (putative 1-(5-phosphoribosyl)-5-[(5-phosphoribosylamino)methylidene amino] imidazole-4-carboxamide isomerase [Clostridium difficile 630]) 
           
  Protein Group 145   
      Expression Quality:  
         Score      Num Spectra      Num Peptides      High-Qual Peptides      % Coverage       114    4    3    1    12   
   
      Peptides:   
        Query    Observed    Mr(expt)    Mr(calc)    Score    Peptide    Result File   
		    76    459.79    917.57    917.48    37    GENIGTTVK    37A_3   
		    205    632.35    1262.68    1262.61    46    NVDAVYDKDPK    37A_3   
		    164    475.30    948.59    948.47    31    VQTAIDMR +Oxidation (M)    37_3   
   
      Matching Genes:  
               gi|115251192|emb|CAJ69023.1|  (uridylate kinase [Clostridium difficile 630]) 
           
  Protein Group 146   
      Expression Quality:  
         Score      Num Spectra      Num Peptides      High-Qual Peptides      % Coverage       112    3    2    2    36   
   
      Peptides:   
        Query    Observed    Mr(expt)    Mr(calc)    Score    Peptide    Result File   
		    784    784.73    2351.17    2350.11    45    EAEAALNAFMSSVQDALVNNEK    biorepCdiff_1   
		    444    677.39    1352.77    1352.71    67    VQLVGFGTFETR    37_1   
   
      Matching Genes:  
               gi|115252557|emb|CAJ70400.1|  (DNA-binding protein HU [Clostridium difficile 630]) 
           
  Protein Group 147   
      Expression Quality:  
         Score      Num Spectra      Num Peptides      High-Qual Peptides      % Coverage       111    2    2    1    12   
   
      Peptides:   
        Query    Observed    Mr(expt)    Mr(calc)    Score    Peptide    Result File   
		    551    915.24    2742.70    2742.33    74    GIMTVEDALMAVEAGVDAIVVSNHGGR +2 Oxidation (M)    37A_4   
		    288    779.93    1557.85    1557.75    37    VLDCTPGACEVLPK    37A_4   
   
      Matching Genes:  
               gi|115250297|emb|CAJ68119.1|  (putative FMN-dependent dehydrogenase [Clostridium difficile 630]) 
           
  Protein Group 148   
      Expression Quality:  
         Score      Num Spectra      Num Peptides      High-Qual Peptides      % Coverage       110    3    2    1    21   
   
      Peptides:   
        Query    Observed    Mr(expt)    Mr(calc)    Score    Peptide    Result File   
		    425    711.51    2131.50    2131.16    81    IAQMIVKPIYDINIEEVK +Oxidation (M)    37A_2   
		    320    489.97    1466.89    1466.75    29    LNDDAIIPNFAHK    37A_2   
   
      Matching Genes:  
               gi|115251455|emb|CAJ69288.1|  (deoxyuridine 5'-triphosphate nucleotidohydrolase [Clostridium difficile 630]) 
           
  Protein Group 149   
      Expression Quality:  
         Score      Num Spectra      Num Peptides      High-Qual Peptides      % Coverage       109    3    2    2    10   
   
      Peptides:   
        Query    Observed    Mr(expt)    Mr(calc)    Score    Peptide    Result File   
		    202    687.98    1373.95    1373.76    61    ALQATGLEVTMIK    biorepCdiffA_2   
		    427    696.06    1390.11    1389.75    48    ALQATGLEVTMIK +Oxidation (M)    biorepCdiff_2   
   
      Matching Genes:  
               gi|115249104|emb|CAJ66915.1|  (30S ribosomal protein S11 [Clostridium difficile 630]) 
           
  Protein Group 150   
      Expression Quality:  
         Score      Num Spectra      Num Peptides      High-Qual Peptides      % Coverage       107    2    2    1    10   
   
      Peptides:   
        Query    Observed    Mr(expt)    Mr(calc)    Score    Peptide    Result File   
		    411    854.55    1707.09    1705.90    38    AAINSLTQNIATQYAK    37_4   
		    233    634.39    1266.77    1267.64    69    SEELAHEVINK    37_4   
   
      Matching Genes:  
               gi|115249069|emb|CAJ66880.1|  (NADP-dependent 7-alpha-hydroxysteroid dehydrogenase [Clostridium difficile 630]) 
           
  Protein Group 151   
      Expression Quality:  
         Score      Num Spectra      Num Peptides      High-Qual Peptides      % Coverage       107    2    2    2    8   
   
      Peptides:   
        Query    Observed    Mr(expt)    Mr(calc)    Score    Peptide    Result File   
		    214    628.04    1254.07    1253.75    46    LPIILYNVPGR    biorepCdiff_4   
		    366    761.21    1520.41    1519.78    61    TAMNILGFNVGDLR    biorepCdiff_4   
   
      Matching Genes:  
               gi|115252280|emb|CAJ70121.1|  (dihydrodipicolinate synthase [Clostridium difficile 630]) 
           
  Protein Group 152   
      Expression Quality:  
         Score      Num Spectra      Num Peptides      High-Qual Peptides      % Coverage       102    4    3    1    6   
   
      Peptides:   
        Query    Observed    Mr(expt)    Mr(calc)    Score    Peptide    Result File   
		    478    1042.75    2083.49    2082.92    40    GTYGDTYGIHGNNNESSIGK    biorepCdiff_7   
		    241    700.53    1399.05    1399.73    33    IAEELGNREEIK    biorepCdiff_7   
		    60    537.38    1072.75    1072.57    29    INLNQTGWK    biorepCdiffA_7   
   
      Matching Genes:  
               gi|115251764|emb|CAJ69599.1|  (cell surface protein [Clostridium difficile 630]) 
           
  Protein Group 153   
      Expression Quality:  
         Score      Num Spectra      Num Peptides      High-Qual Peptides      % Coverage       102    5    2    1    32   
   
      Peptides:   
        Query    Observed    Mr(expt)    Mr(calc)    Score    Peptide    Result File   
		    735    1027.39    2052.77    2052.02    38    NYELVYVVKPNSDEEVR    biorepCdiff_1   
		    198    694.02    1386.03    1385.78    64    VKEVVATDGEIVK    biorepCdiffA_1   
   
      Matching Genes:  
               gi|115252728|emb|CAJ70572.1|  (30S ribosomal protein S6 [Clostridium difficile 630]) 
           
  Protein Group 154   
      Expression Quality:  
         Score      Num Spectra      Num Peptides      High-Qual Peptides      % Coverage       101    6    3    0    24   
   
      Peptides:   
        Query    Observed    Mr(expt)    Mr(calc)    Score    Peptide    Result File   
		    20    410.25    818.48    818.45    39    IETTVTR    37A_1   
		    538    697.60    1393.20    1392.70    31    KGDAAEMAFIELV    biorepCdiff_1   
		    275    494.94    987.87    987.55    31    NLVTCLLR    biorepCdiff_1   
   
      Matching Genes:  
               gi|115249107|emb|CAJ66918.1|  (50S ribosomal protein L17 [Clostridium difficile 630]) 
           
  Protein Group 155   
      Expression Quality:  
         Score      Num Spectra      Num Peptides      High-Qual Peptides      % Coverage       99    2    2    2    8   
   
      Peptides:   
        Query    Observed    Mr(expt)    Mr(calc)    Score    Peptide    Result File   
		    339    663.01    1324.00    1323.64    55    VMFELAGVSEDK    biorepCdiff_3   
		    268    670.87    1339.72    1339.63    44    VMFELAGVSEDK +Oxidation (M)    37A_2   
   
      Matching Genes:  
               gi|115249084|emb|CAJ66895.1|  (50S ribosomal protein L16 [Clostridium difficile 630]) 
           
  Protein Group 156   
      Expression Quality:  
         Score      Num Spectra      Num Peptides      High-Qual Peptides      % Coverage       97    3    3    0    26   
   
      Peptides:   
        Query    Observed    Mr(expt)    Mr(calc)    Score    Peptide    Result File   
		    288    813.19    1624.36    1623.81    31    HVPVYITEDMVGHK    biorepCdiffA_1   
		    291    821.10    1640.19    1639.80    34    HVPVYITEDMVGHK +Oxidation (M)    biorepCdiffA_1   
		    282    581.82    1161.63    1161.58    32    KIEAMNASGNK    37_1   
   
      Matching Genes:  
               gi|115249081|emb|CAJ66892.1|  (30S ribosomal protein S19 [Clostridium difficile 630]) 
           
  Protein Group 157   
      Expression Quality:  
         Score      Num Spectra      Num Peptides      High-Qual Peptides      % Coverage       97    3    3    0    17   
   
      Peptides:   
        Query    Observed    Mr(expt)    Mr(calc)    Score    Peptide    Result File   
		    261    687.98    1373.94    1373.69    34    FIVGYGIDYAEK    37A_3   
		    122    582.48    1162.95    1162.63    27    GASVFVSDLIR    biorepCdiffA_3   
		    210    504.25    1006.49    1006.50    36    MLTEEQIK +Oxidation (M)    37_2   
   
      Matching Genes:  
               gi|115251742|emb|CAJ69577.1|  (putative phosphoribosyltransferase [Clostridium difficile 630]) 
           
  Protein Group 158   
      Expression Quality:  
         Score      Num Spectra      Num Peptides      High-Qual Peptides      % Coverage       97    2    2    1    8   
   
      Peptides:   
        Query    Observed    Mr(expt)    Mr(calc)    Score    Peptide    Result File   
		    48    434.97    867.93    867.52    38    IEPGAIIR    biorepCdiff_4   
		    256    712.92    1423.82    1423.68    59    MVNLNDAYEIAR +Oxidation (M)    37A_4   
   
      Matching Genes:  
               gi|115252284|emb|CAJ70125.1|  (2,3,4,5-tetrahydropyridine-2,6-dicarboxylate N-succinyltransferase [Clostridium difficile 630]) 
           
  Protein Group 159   
      Expression Quality:  
         Score      Num Spectra      Num Peptides      High-Qual Peptides      % Coverage       96    2    2    1    12   
   
      Peptides:   
        Query    Observed    Mr(expt)    Mr(calc)    Score    Peptide    Result File   
		    259    588.36    1174.71    1174.66    59    TPEQLSIFLK    37_2   
		    156    551.34    1100.66    1100.58    37    VLEDDKNLR    37A_2   
   
      Matching Genes:  
               gi|115249226|emb|CAJ67039.1|  (phosphoribosylaminoimidazole carboxylase catalytic subunit [Clostridium difficile 630]) 
           
  Protein Group 160   
      Expression Quality:  
         Score      Num Spectra      Num Peptides      High-Qual Peptides      % Coverage       96    3    1    1    9   
   
      Peptides:   
        Query    Observed    Mr(expt)    Mr(calc)    Score    Peptide    Result File   
		    478    715.91    1429.81    1429.74    96    VTVDENTIGQINK    37_2   
   
      Matching Genes:  
               gi|115251795|emb|CAJ69630.1|  (putative D-tyrosyl-tRNA protein [Clostridium difficile 630]) 
           
  Protein Group 161   
      Expression Quality:  
         Score      Num Spectra      Num Peptides      High-Qual Peptides      % Coverage       96    2    2    1    13   
   
      Peptides:   
        Query    Observed    Mr(expt)    Mr(calc)    Score    Peptide    Result File   
		    536    1078.23    2154.46    2153.99    68    CIWGEGDGSTLTVVDTPYGK    37_4   
		    618    1211.39    2420.77    2420.07    28    ELDAEPEIMCPGGSCIVDPFGK    biorepCdiff_4   
   
      Matching Genes:  
               gi|115251895|emb|CAJ69730.1|  (nitrilase (carbon-nitrogen hydrolase) [Clostridium difficile 630]) 
           
  Protein Group 162   
      Expression Quality:  
         Score      Num Spectra      Num Peptides      High-Qual Peptides      % Coverage       93    3    2    1    22   
   
      Peptides:   
        Query    Observed    Mr(expt)    Mr(calc)    Score    Peptide    Result File   
		    139    651.97    1301.93    1301.68    32    EVEASVGGGAVTVK    biorepCdiff_8   
		    186    675.39    1348.77    1348.63    61    NIDDIQASQMSK    biorepCdiffA_1   
   
      Matching Genes:  
               gi|115249020|emb|CAJ66831.1|  (conserved hypothetical protein [Clostridium difficile 630]) 
           
  Protein Group 163   
      Expression Quality:  
         Score      Num Spectra      Num Peptides      High-Qual Peptides      % Coverage       91    2    1    1    22   
   
      Peptides:   
        Query    Observed    Mr(expt)    Mr(calc)    Score    Peptide    Result File   
		    511    745.43    1488.85    1488.71    91    EAEEGCPVSAITVK    37_1   
   
      Matching Genes:  
               gi|115252670|emb|CAJ70513.1|  (ferredoxin [Clostridium difficile 630]) 
           
  Protein Group 164   
      Expression Quality:  
         Score      Num Spectra      Num Peptides      High-Qual Peptides      % Coverage       87    2    2    1    13   
   
      Peptides:   
        Query    Observed    Mr(expt)    Mr(calc)    Score    Peptide    Result File   
		    66    484.89    967.76    967.57    39    ILLDEPIR    biorepCdiffA_3   
		    220    566.65    1131.29    1131.60    48    MAVQADSVAIK    biorepCdiff_3   
   
      Matching Genes:  
               gi|115252722|emb|CAJ70566.1|  (50S ribosomal protein L9 [Clostridium difficile 630]) 
           
  Protein Group 165   
      Expression Quality:  
         Score      Num Spectra      Num Peptides      High-Qual Peptides      % Coverage       85    3    2    1    25   
   
      Peptides:   
        Query    Observed    Mr(expt)    Mr(calc)    Score    Peptide    Result File   
		    237    628.98    1255.94    1255.71    59    IIQLDSNVINK    37A_1   
		    93    440.30    878.59    878.42    26    VSFNQER    37A_1   
   
      Matching Genes:  
               gi|115249124|emb|CAJ66935.1|  (ferredoxin [Clostridium difficile 630]) 
           
  Protein Group 166   
      Expression Quality:  
         Score      Num Spectra      Num Peptides      High-Qual Peptides      % Coverage       83    3    2    1    25   
   
      Peptides:   
        Query    Observed    Mr(expt)    Mr(calc)    Score    Peptide    Result File   
		    622    698.03    2091.08    2090.83    30    LVEFPSDHTCSHDGCGHH    37_1   
		    328    479.62    1435.85    1435.63    53    SLDNGDLDHEHGK    37A_1   
   
      Matching Genes:  
               gi|115250736|emb|CAJ68560.1|  (putative dinitrogenase iron-molybdenum cofactor [Clostridium difficile 630]) 
           
  Protein Group 167   
      Expression Quality:  
         Score      Num Spectra      Num Peptides      High-Qual Peptides      % Coverage       81    1    1    1    5   
   
      Peptides:   
        Query    Observed    Mr(expt)    Mr(calc)    Score    Peptide    Result File   
		    391    930.05    1858.09    1857.88    81    TIDEDESGALNPELVEK    37A_4   
   
      Matching Genes:  
               gi|115251648|emb|CAJ69481.1|  (low-specificity L-threonine aldolase [Clostridium difficile 630]) 
           
  Protein Group 168   
      Expression Quality:  
         Score      Num Spectra      Num Peptides      High-Qual Peptides      % Coverage       81    2    2    2    4   
   
      Peptides:   
        Query    Observed    Mr(expt)    Mr(calc)    Score    Peptide    Result File   
		    335    807.63    1613.24    1612.76    40    FFNEEEMNAILTR    biorepCdiff_6   
		    142    593.72    1185.42    1184.64    41    VGQEGVTSPIAK    biorepCdiff_6   
   
      Matching Genes:  
               gi|115251791|emb|CAJ69626.1|  (putative aspartyl-tRNA synthetase [Clostridium difficile 630]) 
           
  Protein Group 169   
      Expression Quality:  
         Score      Num Spectra      Num Peptides      High-Qual Peptides      % Coverage       79    2    2    1    11   
   
      Peptides:   
        Query    Observed    Mr(expt)    Mr(calc)    Score    Peptide    Result File   
		    43    417.77    833.52    833.46    33    LSVSATTR    37_3   
		    332    788.51    1575.00    1574.76    46    TGEVDGVNYFFISK    37A_3   
   
      Matching Genes:  
               gi|115251645|emb|CAJ69478.1|  (guanylate kinase [Clostridium difficile 630]) 
           
  Protein Group 170   
      Expression Quality:  
         Score      Num Spectra      Num Peptides      High-Qual Peptides      % Coverage       78    3    1    1    4   
   
      Peptides:   
        Query    Observed    Mr(expt)    Mr(calc)    Score    Peptide    Result File   
		    180    693.01    1384.01    1383.80    78    ILITGSPIGGISEK    37A_5   
   
      Matching Genes:  
               gi|115250793|emb|CAJ68617.1|  (putative 2-hydroxyacyl-CoA dehydratase [Clostridium difficile 630]) 
           
  Protein Group 171   
      Expression Quality:  
         Score      Num Spectra      Num Peptides      High-Qual Peptides      % Coverage       78    2    2    1    7   
   
      Peptides:   
        Query    Observed    Mr(expt)    Mr(calc)    Score    Peptide    Result File   
		    320    778.02    1554.03    1553.78    32    GIPVSIGTDGAPSNNR    37_6   
		    343    817.54    1633.06    1632.75    46    SVMDTGDGLPEAWQK    37_6   
   
      Matching Genes:  
               gi|115251756|emb|CAJ69591.1|  (putative amidohydrolas [Clostridium difficile 630]) 
           
  Protein Group 172   
      Expression Quality:  
         Score      Num Spectra      Num Peptides      High-Qual Peptides      % Coverage       77    2    2    1    4   
   
      Peptides:   
        Query    Observed    Mr(expt)    Mr(calc)    Score    Peptide    Result File   
		    90    592.94    1183.86    1183.61    29    LDIYQEYIK    biorepCdiffA_6   
		    159    687.55    1373.08    1372.72    48    NLSKEEVEANIK    biorepCdiffA_6   
   
      Matching Genes:  
               gi|115249054|emb|CAJ66865.1|  (glutamyl-tRNA synthetase [Clostridium difficile 630]) 
           
  Protein Group 173   
      Expression Quality:  
         Score      Num Spectra      Num Peptides      High-Qual Peptides      % Coverage       75    2    2    1    5   
   
      Peptides:   
        Query    Observed    Mr(expt)    Mr(calc)    Score    Peptide    Result File   
		    66    494.80    987.58    987.46    41    QEDLEAGAR    37A_6   
		    288    742.02    1482.02    1481.70    34    SHEEGISPEEVAAK    biorepCdiff_6   
   
      Matching Genes:  
               gi|115249055|emb|CAJ66866.1|  (cysteinyl-tRNA synthetase [Clostridium difficile 630]) 
           
  Protein Group 174   
      Expression Quality:  
         Score      Num Spectra      Num Peptides      High-Qual Peptides      % Coverage       75    1    1    1    8   
   
      Peptides:   
        Query    Observed    Mr(expt)    Mr(calc)    Score    Peptide    Result File   
		    439    716.51    1431.00    1430.76    75    VTSTGIVNGVIEDK    37_3   
   
      Matching Genes:  
               gi|115252223|emb|CAJ70063.1|  (putative phosphatidylethanolamine-binding regulatory protein [Clostridium difficile 630]) 
           
  Protein Group 175   
      Expression Quality:  
         Score      Num Spectra      Num Peptides      High-Qual Peptides      % Coverage       73    2    1    1    4   
   
      Peptides:   
        Query    Observed    Mr(expt)    Mr(calc)    Score    Peptide    Result File   
		    287    778.95    1555.89    1555.76    73    SIQAIDSHTAGEATR    37A_4   
   
      Matching Genes:  
               gi|115252294|emb|CAJ70135.1|  (putative proline racemase [Clostridium difficile 630]) 
           
  Protein Group 176   
      Expression Quality:  
         Score      Num Spectra      Num Peptides      High-Qual Peptides      % Coverage       73    3    2    1    29   
   
      Peptides:   
        Query    Observed    Mr(expt)    Mr(calc)    Score    Peptide    Result File   
		    347    914.46    1826.90    1826.94    41    AEGDTGSPEVQIALLTAR    biorepCdiffA_1   
		    17    417.93    833.84    833.50    32    NLLAYLK    biorepCdiffA_1   
   
      Matching Genes:  
               gi|115250352|emb|CAJ68174.1|  (30S ribosomal protein S15 [Clostridium difficile 630]) 
           
  Protein Group 177   
      Expression Quality:  
         Score      Num Spectra      Num Peptides      High-Qual Peptides      % Coverage       72    1    1    1    4   
   
      Peptides:   
        Query    Observed    Mr(expt)    Mr(calc)    Score    Peptide    Result File   
		    139    611.34    1220.67    1220.64    72    AYGANLVLTDGK    37A_4   
   
      Matching Genes:  
               gi|115250635|emb|CAJ68459.1|  (putative O-acetylserine sulfhydrylase [Clostridium difficile 630]) 
           
  Protein Group 178   
      Expression Quality:  
         Score      Num Spectra      Num Peptides      High-Qual Peptides      % Coverage       72    2    2    1    4   
   
      Peptides:   
        Query    Observed    Mr(expt)    Mr(calc)    Score    Peptide    Result File   
		    209    664.39    1326.76    1326.58    27    ATEEFNCTIDK    biorepCdiff_6   
		    154    652.36    1302.71    1302.60    45    SATHDQNFLDR    37A_6   
   
      Matching Genes:  
               gi|115251601|emb|CAJ69434.1|  (conserved hypothetical protein [Clostridium difficile 630]) 
           
  Protein Group 179   
      Expression Quality:  
         Score      Num Spectra      Num Peptides      High-Qual Peptides      % Coverage       70    2    1    1    9   
   
      Peptides:   
        Query    Observed    Mr(expt)    Mr(calc)    Score    Peptide    Result File   
		    351    881.72    1761.43    1760.81    70    TGVGDGDDEQINVDLSK    biorepCdiffA_3   
   
      Matching Genes:  
               gi|115250845|emb|CAJ68669.1|  (tellurium resistance protein [Clostridium difficile 630]) 
           
  Protein Group 180   
      Expression Quality:  
         Score      Num Spectra      Num Peptides      High-Qual Peptides      % Coverage       69    1    1    1    4   
   
      Peptides:   
        Query    Observed    Mr(expt)    Mr(calc)    Score    Peptide    Result File   
		    165    622.90    1243.79    1243.68    69    TGAASGAAIDVLAK    37A_4   
   
      Matching Genes:  
               gi|115249560|emb|CAJ67377.1|  (putative ornithine cyclodeaminase [Clostridium difficile 630]) 
           
  Protein Group 181   
      Expression Quality:  
         Score      Num Spectra      Num Peptides      High-Qual Peptides      % Coverage       68    1    1    1    18   
   
      Peptides:   
        Query    Observed    Mr(expt)    Mr(calc)    Score    Peptide    Result File   
		    630    688.96    2063.86    2063.24    68    IATAGVVLGAVTGAVSGVLLAPK    biorepCdiff_2   
   
      Matching Genes:  
               gi|115250942|emb|CAJ68770.1|  (putative membrane protein [Clostridium difficile 630]) 
           
  Protein Group 182   
      Expression Quality:  
         Score      Num Spectra      Num Peptides      High-Qual Peptides      % Coverage       66    2    2    0    8   
   
      Peptides:   
        Query    Observed    Mr(expt)    Mr(calc)    Score    Peptide    Result File   
		    171    576.31    1150.61    1150.57    33    IASFSQQSQR    37_4   
		    117    576.33    1150.64    1149.60    33    LISHTPEPEK    37A_4   
   
      Matching Genes:  
               gi|115249057|emb|CAJ66868.1|  (putative thymidylate synthase [Clostridium difficile 630]) 
           
  Protein Group 183   
      Expression Quality:  
         Score      Num Spectra      Num Peptides      High-Qual Peptides      % Coverage       66    2    1    1    5   
   
      Peptides:   
        Query    Observed    Mr(expt)    Mr(calc)    Score    Peptide    Result File   
		    191    503.79    1005.56    1005.51    66    VPGATYAEAK    37_3   
   
      Matching Genes:  
               gi|115251487|emb|CAJ69320.1|  (conserved hypothetical protein [Clostridium difficile 630]) 
           
  Protein Group 184   
      Expression Quality:  
         Score      Num Spectra      Num Peptides      High-Qual Peptides      % Coverage       65    2    2    0    12   
   
      Peptides:   
        Query    Observed    Mr(expt)    Mr(calc)    Score    Peptide    Result File   
		    36    429.28    856.54    856.50    33    AALLAPSSK    37A_3   
		    275    503.75    1508.23    1507.76    32    TWEFIVVDDKEK    biorepCdiffA_3   
   
      Matching Genes:  
               gi|115249854|emb|CAJ67671.1|  (putative reductase [Clostridium difficile 630]) 
           
  Protein Group 185   
      Expression Quality:  
         Score      Num Spectra      Num Peptides      High-Qual Peptides      % Coverage       65    1    1    1    2   
   
      Peptides:   
        Query    Observed    Mr(expt)    Mr(calc)    Score    Peptide    Result File   
		    237    672.44    1342.86    1342.67    65    VVNINANEVDEK    37_6   
   
      Matching Genes:  
               gi|115251568|emb|CAJ69401.1|  (L-aspartate-beta-decarboxylase [Clostridium difficile 630]) 
           
  Protein Group 186   
      Expression Quality:  
         Score      Num Spectra      Num Peptides      High-Qual Peptides      % Coverage       63    3    2    0    8   
   
      Peptides:   
        Query    Observed    Mr(expt)    Mr(calc)    Score    Peptide    Result File   
		    288    603.28    1204.54    1204.54    33    DGHTNLYEEK    37_1   
		    282    791.57    1581.13    1580.79    30    NTDIKEEYLSEIK    biorepCdiffA_2   
   
      Matching Genes:  
               gi|115250155|emb|CAJ67976.1|  (nitroreductase-family protein [Clostridium difficile 630]) 
           
  Protein Group 187   
      Expression Quality:  
         Score      Num Spectra      Num Peptides      High-Qual Peptides      % Coverage       63    1    1    1    9   
   
      Peptides:   
        Query    Observed    Mr(expt)    Mr(calc)    Score    Peptide    Result File   
		    487    781.07    1560.12    1559.86    63    FINGILGSVVDEIGK    37_3   
   
      Matching Genes:  
               gi|115250234|emb|CAJ68055.1|  (N utilization substance protein B [Clostridium difficile 630]) 
           
  Protein Group 188   
      Expression Quality:  
         Score      Num Spectra      Num Peptides      High-Qual Peptides      % Coverage       63    1    1    1    2   
   
      Peptides:   
        Query    Observed    Mr(expt)    Mr(calc)    Score    Peptide    Result File   
		    183    749.05    1496.09    1495.69    63    VYGEGADSQGVSATR    biorepCdiffA_7   
   
      Matching Genes:  
               gi|115251074|emb|CAJ68905.1|  (chaperone [Clostridium difficile 630]) 
           
  Protein Group 189   
      Expression Quality:  
         Score      Num Spectra      Num Peptides      High-Qual Peptides      % Coverage       62    1    1    1    15   
   
      Peptides:   
        Query    Observed    Mr(expt)    Mr(calc)    Score    Peptide    Result File   
		    304    838.83    1675.64    1675.83    62    VETQGATGIDNELTTK    biorepCdiffA_1   
   
      Matching Genes:  
               gi|115252071|emb|CAJ69908.1|  (PTS system, IIb component [Clostridium difficile 630]) 
           
  Protein Group 190   
      Expression Quality:  
         Score      Num Spectra      Num Peptides      High-Qual Peptides      % Coverage       61    2    1    1    12   
   
      Peptides:   
        Query    Observed    Mr(expt)    Mr(calc)    Score    Peptide    Result File   
		    318    861.73    1721.45    1720.82    61    GYDVSSISTPDLENPK    biorepCdiffA_1   
   
      Matching Genes:  
               gi|115249013|emb|CAJ66824.1|  (anti-sigma-B factor (serine-protein kinase) [Clostridium difficile 630]) 
           
  Protein Group 191   
      Expression Quality:  
         Score      Num Spectra      Num Peptides      High-Qual Peptides      % Coverage       61    2    1    1    12   
   
      Peptides:   
        Query    Observed    Mr(expt)    Mr(calc)    Score    Peptide    Result File   
		    348    636.36    1270.71    1270.69    61    QNINIVDISQK    37_1   
   
      Matching Genes:  
               gi|115251678|emb|CAJ69513.1|  (conserved hypothetical protein [Clostridium difficile 630]) 
           
  Protein Group 192   
      Expression Quality:  
         Score      Num Spectra      Num Peptides      High-Qual Peptides      % Coverage       60    1    1    1    4   
   
      Peptides:   
        Query    Observed    Mr(expt)    Mr(calc)    Score    Peptide    Result File   
		    562    868.58    1735.16    1734.89    60    VITGLATSDDDSSITIK    37_2   
   
      Matching Genes:  
               gi|115251108|emb|CAJ68939.1|  (aspartokinase [Clostridium difficile 630]) 
           
  Protein Group 193   
      Expression Quality:  
         Score      Num Spectra      Num Peptides      High-Qual Peptides      % Coverage       58    2    1    1    6   
   
      Peptides:   
        Query    Observed    Mr(expt)    Mr(calc)    Score    Peptide    Result File   
		    309    815.52    1629.03    1628.88    58    FGDGGVDILPIANLTK    37A_4   
   
      Matching Genes:  
               gi|115249811|emb|CAJ67628.1|  (NH3-dependent NAD(+) synthetase [Clostridium difficile 630]) 
           
  Protein Group 194   
      Expression Quality:  
         Score      Num Spectra      Num Peptides      High-Qual Peptides      % Coverage       58    1    1    1    10   
   
      Peptides:   
        Query    Observed    Mr(expt)    Mr(calc)    Score    Peptide    Result File   
		    412    693.95    1385.88    1385.70    58    ELVPNTTDAAVEK    37_3   
   
      Matching Genes:  
               gi|115249844|emb|CAJ67661.1|  (rubredoxin oxidoreductase (desulfoferrodoxin) [Clostridium difficile 630]) 
           
  Protein Group 195   
      Expression Quality:  
         Score      Num Spectra      Num Peptides      High-Qual Peptides      % Coverage       58    1    1    1    4   
   
      Peptides:   
        Query    Observed    Mr(expt)    Mr(calc)    Score    Peptide    Result File   
		    611    1182.90    2363.78    2364.29    58    EILGDIPISLSNEIGNIGILER    biorepCdiff_6   
   
      Matching Genes:  
               gi|115250762|emb|CAJ68586.1|  (putative hydantoinase [Clostridium difficile 630]) 
           
  Protein Group 196   
      Expression Quality:  
         Score      Num Spectra      Num Peptides      High-Qual Peptides      % Coverage       58    2    1    1    3   
   
      Peptides:   
        Query    Observed    Mr(expt)    Mr(calc)    Score    Peptide    Result File   
		    195    644.44    1286.87    1286.63    58    ADAYGHGAVEVAK    biorepCdiff_5   
   
      Matching Genes:  
               gi|115252523|emb|CAJ70366.1|  (alanine racemase [Clostridium difficile 630]) 
           
  Protein Group 197   
      Expression Quality:  
         Score      Num Spectra      Num Peptides      High-Qual Peptides      % Coverage       58    1    1    1    7   
   
      Peptides:   
        Query    Observed    Mr(expt)    Mr(calc)    Score    Peptide    Result File   
		    363    652.35    1302.68    1302.59    58    SAEFCANYITK    37_2   
   
      Matching Genes:  
               gi|115252727|emb|CAJ70571.1|  (single-strand binding protein [Clostridium difficile 630]) 
           
  Protein Group 198   
      Expression Quality:  
         Score      Num Spectra      Num Peptides      High-Qual Peptides      % Coverage       57    2    1    1    6   
   
      Peptides:   
        Query    Observed    Mr(expt)    Mr(calc)    Score    Peptide    Result File   
		    384    1041.20    2080.38    2080.03    57    TLEKPGTNVSGTSDFVSVDK    37_5   
   
      Matching Genes:  
               gi|115249887|emb|CAJ67706.1|  (ABC transporter, substrate-binding lipoprotein [Clostridium difficile 630]) 
           
  Protein Group 199   
      Expression Quality:  
         Score      Num Spectra      Num Peptides      High-Qual Peptides      % Coverage       57    3    1    1    16   
   
      Peptides:   
        Query    Observed    Mr(expt)    Mr(calc)    Score    Peptide    Result File   
		    671    900.41    1798.80    1797.88    57    FIEEIGYYNPISEPK    biorepCdiff_1   
   
      Matching Genes:  
               gi|115250287|emb|CAJ68109.1|  (30S ribosomal protein S16 [Clostridium difficile 630]) 
           
  Protein Group 200   
      Expression Quality:  
         Score      Num Spectra      Num Peptides      High-Qual Peptides      % Coverage       57    1    1    1    9   
   
      Peptides:   
        Query    Observed    Mr(expt)    Mr(calc)    Score    Peptide    Result File   
		    347    801.55    1601.08    1600.82    57    LMVVNDAVANNSVQK    37A_2   
   
      Matching Genes:  
               gi|115252122|emb|CAJ69960.1|  (PTS system, IIb component [Clostridium difficile 630]) 
           
  Protein Group 201   
      Expression Quality:  
         Score      Num Spectra      Num Peptides      High-Qual Peptides      % Coverage       56    1    1    1    13   
   
      Peptides:   
        Query    Observed    Mr(expt)    Mr(calc)    Score    Peptide    Result File   
		    145    631.68    1261.34    1260.71    56    TIVVAVEEFVR    biorepCdiffA_1   
   
      Matching Genes:  
               gi|115249086|emb|CAJ66897.1|  (30S ribosomal protein S17 [Clostridium difficile 630]) 
           
  Protein Group 202   
      Expression Quality:  
         Score      Num Spectra      Num Peptides      High-Qual Peptides      % Coverage       56    1    1    1    7   
   
      Peptides:   
        Query    Observed    Mr(expt)    Mr(calc)    Score    Peptide    Result File   
		    527    862.88    2585.61    2585.31    56    NLIQPEDFSIEEIDEILELAQK    37A_4   
   
      Matching Genes:  
               gi|115249194|emb|CAJ67006.1|  (aspartate carbamoyltransferase catalytic chain [Clostridium difficile 630]) 
           
  Protein Group 203   
      Expression Quality:  
         Score      Num Spectra      Num Peptides      High-Qual Peptides      % Coverage       56    1    1    1    0   
   
      Peptides:   
        Query    Observed    Mr(expt)    Mr(calc)    Score    Peptide    Result File   
		    256    773.51    1545.01    1544.67    56    NIAYNYTDESNNK    biorepCdiff_8   
   
      Matching Genes:  
               gi|115249677|emb|CAJ67494.1|  (toxin A [Clostridium difficile 630]) 
           
  Protein Group 204   
      Expression Quality:  
         Score      Num Spectra      Num Peptides      High-Qual Peptides      % Coverage       56    1    1    1    6   
   
      Peptides:   
        Query    Observed    Mr(expt)    Mr(calc)    Score    Peptide    Result File   
		    176    683.55    1365.09    1364.70    56    IEELQFNPFTK    biorepCdiffA_4   
   
      Matching Genes:  
               gi|115251448|emb|CAJ69281.1|  (conserved hypothetical protein [Clostridium difficile 630]) 
           
  Protein Group 205   
      Expression Quality:  
         Score      Num Spectra      Num Peptides      High-Qual Peptides      % Coverage       56    1    1    1    5   
   
      Peptides:   
        Query    Observed    Mr(expt)    Mr(calc)    Score    Peptide    Result File   
		    404    952.58    1903.15    1902.89    56    SLGPEPWQVCYVEPSR    37A_4   
   
      Matching Genes:  
               gi|115251486|emb|CAJ69319.1|  (glycyl-tRNA synthetase alpha chain [Clostridium difficile 630]) 
           
  Protein Group 206   
      Expression Quality:  
         Score      Num Spectra      Num Peptides      High-Qual Peptides      % Coverage       55    1    1    1    4   
   
      Peptides:   
        Query    Observed    Mr(expt)    Mr(calc)    Score    Peptide    Result File   
		    306    621.88    1241.75    1241.64    55    VDLPLWQESR    37_3   
   
      Matching Genes:  
               gi|115250457|emb|CAJ68280.1|  (putative ATP-binding protein [Clostridium difficile 630]) 
           
  Protein Group 207   
      Expression Quality:  
         Score      Num Spectra      Num Peptides      High-Qual Peptides      % Coverage       54    1    1    1    7   
   
      Peptides:   
        Query    Observed    Mr(expt)    Mr(calc)    Score    Peptide    Result File   
		    385    870.05    1738.08    1737.95    54    IIIENLTNLENVPEK    37A_3   
   
      Matching Genes:  
               gi|115251016|emb|CAJ68845.1|  (putative cyclase [Clostridium difficile 630]) 
           
  Protein Group 208   
      Expression Quality:  
         Score      Num Spectra      Num Peptides      High-Qual Peptides      % Coverage       54    2    1    1    4   
   
      Peptides:   
        Query    Observed    Mr(expt)    Mr(calc)    Score    Peptide    Result File   
		    353    504.32    1509.93    1509.83    54    ISSKPIIATHSNSR    37_4   
   
      Matching Genes:  
               gi|115252633|emb|CAJ70476.1|  (probable dipeptidase [Clostridium difficile 630]) 
           
  Protein Group 209   
      Expression Quality:  
         Score      Num Spectra      Num Peptides      High-Qual Peptides      % Coverage       54    1    1    1    22   
   
      Peptides:   
        Query    Observed    Mr(expt)    Mr(calc)    Score    Peptide    Result File   
		    147    636.60    1271.18    1270.70    54    LIDEALEDLIK    biorepCdiffA_1   
   
      Matching Genes:  
               gi|110666931|ref|YP_659585.1|  (hypothetical protein CDP09 [Clostridium difficile 630]) 
           
  Protein Group 210   
      Expression Quality:  
         Score      Num Spectra      Num Peptides      High-Qual Peptides      % Coverage       53    1    1    1    2   
   
      Peptides:   
        Query    Observed    Mr(expt)    Mr(calc)    Score    Peptide    Result File   
		    123    533.38    1064.74    1064.55    53    IGGAEQIYSK    37_6   
   
      Matching Genes:  
               gi|115251373|emb|CAJ69205.1|  (putative phosphoglucomutase [Clostridium difficile 630]) 
           
  Protein Group 211   
      Expression Quality:  
         Score      Num Spectra      Num Peptides      High-Qual Peptides      % Coverage       53    3    1    1    5   
   
      Peptides:   
        Query    Observed    Mr(expt)    Mr(calc)    Score    Peptide    Result File   
		    215    638.38    1274.75    1274.60    53    NQDVSDEDILK    37A_3   
   
      Matching Genes:  
               gi|115251628|emb|CAJ69461.1|  (putative nitroreductase [Clostridium difficile 630]) 
           
  Protein Group 212   
      Expression Quality:  
         Score      Num Spectra      Num Peptides      High-Qual Peptides      % Coverage       52    1    1    1    6   
   
      Peptides:   
        Query    Observed    Mr(expt)    Mr(calc)    Score    Peptide    Result File   
		    242    551.30    1100.58    1100.61    52    EDIQVIGISK    37_2   
   
      Matching Genes:  
               gi|115250867|emb|CAJ68692.1|  (putative thiol peroxidase (bacterioferritin comigratory protein) [Clostridium difficile 630]) 
           
  Protein Group 213   
      Expression Quality:  
         Score      Num Spectra      Num Peptides      High-Qual Peptides      % Coverage       50    1    1    1    6   
   
      Peptides:   
        Query    Observed    Mr(expt)    Mr(calc)    Score    Peptide    Result File   
		    149    574.88    1147.75    1147.61    50    GLTSVATDSLGK    37A_3   
   
      Matching Genes:  
               gi|115250535|emb|CAJ68359.1|  (putative transcriptional regulator [Clostridium difficile 630]) 
           
  Protein Group 214   
      Expression Quality:  
         Score      Num Spectra      Num Peptides      High-Qual Peptides      % Coverage       48    2    1    1    11   
   
      Peptides:   
        Query    Observed    Mr(expt)    Mr(calc)    Score    Peptide    Result File   
		    449    700.70    2099.09    2098.09    48    GVTGFVGPGSKPVPLSEDEVK    37A_3   
   
      Matching Genes:  
               gi|115249064|emb|CAJ66875.1|  (transcription antitermination protein [Clostridium difficile 630]) 
           
  Protein Group 215   
      Expression Quality:  
         Score      Num Spectra      Num Peptides      High-Qual Peptides      % Coverage       48    2    1    1    3   
   
      Peptides:   
        Query    Observed    Mr(expt)    Mr(calc)    Score    Peptide    Result File   
		    162    438.67    1312.98    1312.62    48    IGHMGENANLNK +Oxidation (M)    37_5   
   
      Matching Genes:  
               gi|115251586|emb|CAJ69419.1|  (putative aminotransferase [Clostridium difficile 630]) 
           
  Protein Group 216   
      Expression Quality:  
         Score      Num Spectra      Num Peptides      High-Qual Peptides      % Coverage       48    1    1    1    10   
   
      Peptides:   
        Query    Observed    Mr(expt)    Mr(calc)    Score    Peptide    Result File   
		    419    635.57    1269.13    1268.60    48    IGIDEDHSVER    biorepCdiff_1   
   
      Matching Genes:  
               gi|115252725|emb|CAJ70569.1|  (conserved hypothetical protein [Clostridium difficile 630]) 
           
  Protein Group 217   
      Expression Quality:  
         Score      Num Spectra      Num Peptides      High-Qual Peptides      % Coverage       47    2    1    1    6   
   
      Peptides:   
        Query    Observed    Mr(expt)    Mr(calc)    Score    Peptide    Result File   
		    39    497.77    993.53    993.55    47    YPFLLVDK    biorepCdiffA_8   
   
      Matching Genes:  
               gi|115249137|emb|CAJ66948.1|  ((3R)-hydroxymyristoyl-[acyl carrier protein] dehydratase [Clostridium difficile 630]) 
           
  Protein Group 218   
      Expression Quality:  
         Score      Num Spectra      Num Peptides      High-Qual Peptides      % Coverage       47    1    1    1    4   
   
      Peptides:   
        Query    Observed    Mr(expt)    Mr(calc)    Score    Peptide    Result File   
		    79    520.39    1038.77    1038.64    47    VTALINAIPK    37A_7   
   
      Matching Genes:  
               gi|115252333|emb|CAJ70174.1|  (PTS system, IIc component [Clostridium difficile 630]) 
           
  Protein Group 219   
      Expression Quality:  
         Score      Num Spectra      Num Peptides      High-Qual Peptides      % Coverage       46    1    1    1    5   
   
      Peptides:   
        Query    Observed    Mr(expt)    Mr(calc)    Score    Peptide    Result File   
		    280    756.44    1510.87    1510.81    46    VVEPDILIEEVEK    37A_4   
   
      Matching Genes:  
               gi|115250078|emb|CAJ67898.1|  (3-hydroxybutyryl-CoA dehydratase [Clostridium difficile 630]) 
           
  Protein Group 220   
      Expression Quality:  
         Score      Num Spectra      Num Peptides      High-Qual Peptides      % Coverage       46    1    1    1    4   
   
      Peptides:   
        Query    Observed    Mr(expt)    Mr(calc)    Score    Peptide    Result File   
		    165    600.33    1198.65    1199.55    46    FLGEEYNNSK    37A_3   
   
      Matching Genes:  
               gi|115252486|emb|CAJ70329.1|  (precorrin-4 C(11)-methyltransferase [Clostridium difficile 630]) 
           
  Protein Group 221   
      Expression Quality:  
         Score      Num Spectra      Num Peptides      High-Qual Peptides      % Coverage       46    1    1    1    4   
   
      Peptides:   
        Query    Observed    Mr(expt)    Mr(calc)    Score    Peptide    Result File   
		    187    617.33    1232.65    1232.63    46    GIGYAFQQPPR    37A_3   
   
      Matching Genes:  
               gi|115252673|emb|CAJ70516.1|  (ABC transporter, ATP-binding protein [Clostridium difficile 630]) 
           
  Protein Group 222   
      Expression Quality:  
         Score      Num Spectra      Num Peptides      High-Qual Peptides      % Coverage       45    4    1    1    1   
   
      Peptides:   
        Query    Observed    Mr(expt)    Mr(calc)    Score    Peptide    Result File   
		    177    450.33    1347.98    1347.77    45    GRPVTGPGNRPLK    37A_8   
   
      Matching Genes:  
               gi|115249071|emb|CAJ66882.1|  (DNA-directed RNA polymerase beta' chain [Clostridium difficile 630]) 
           
  Protein Group 223   
      Expression Quality:  
         Score      Num Spectra      Num Peptides      High-Qual Peptides      % Coverage       45    2    1    1    7   
   
      Peptides:   
        Query    Observed    Mr(expt)    Mr(calc)    Score    Peptide    Result File   
		    107    552.04    1102.06    1101.57    45    IPTWIDTEK    biorepCdiffA_1   
   
      Matching Genes:  
               gi|115250980|emb|CAJ68809.1|  (conserved hypothetical protein [Clostridium difficile 630]) 
           
  Protein Group 224   
      Expression Quality:  
         Score      Num Spectra      Num Peptides      High-Qual Peptides      % Coverage       43    1    1    1    3   
   
      Peptides:   
        Query    Observed    Mr(expt)    Mr(calc)    Score    Peptide    Result File   
		    377    872.82    1743.62    1742.87    43    IFPTGVEDVPNEEGLK    37_6   
   
      Matching Genes:  
               gi|115252150|emb|CAJ69988.1|  (6-phospho-beta-glucosidase [Clostridium difficile 630]) 
             
