# Supplementary material for: Semiquantitative Analysis of Clinical Heat Stress in Clostridium difficile Strain 630 Using a GeLC/MS Workflow with emPAI Quantitation
Source: PLoS One. 2014 Feb 24;9(2):e88960. doi: 10.1371/journal.pone.0088960 (PMC3933415; doi:10.1371/journal.pone.0088960)
Supplement: File S5 — PROVALT output html file – overall 41°C proteome, 202 proteins. (HTML) [file pone.0088960.s005.html]

   Complete output   Complete output    
 
   
  Protein Group 1   
      Expression Quality:  
         Score      Num Spectra      Num Peptides      High-Qual Peptides      % Coverage       2383    138    41    32    37   
   
      Peptides:   
        Query    Observed    Mr(expt)    Mr(calc)    Score    Peptide    Result File   
		    423    1051.35    2100.69    2100.04    79    ALASQALSIFGDHQDVMAAR    41_8   
		    428    1059.26    2116.51    2116.04    49    ALASQALSIFGDHQDVMAAR +Oxidation (M)    41_8   
		    440    939.68    2816.02    2815.47    30    AVLEAEKHDGPSLIIAYAPCINHGLK    41A_8   
		    79    537.35    1072.69    1072.53    37    ENAPMIIGGR +Oxidation (M)    41A_7   
		    304    767.02    1532.03    1531.75    62    EPGSTGEPLYLDVR    41_8   
		    1    403.27    804.53    804.41    44    FGDTPIR    41_7   
		    96    471.32    940.62    940.50    34    FYTVNAVK    41_8   
		    530    1335.97    2669.94    2669.17    90    GPSWANSLFEDNAEYGFGMYTAVK +Oxidation (M)    41_8   
		    245    756.02    1510.03    1509.72    70    GTAQNPDIYFQTR    41_7   
		    176    701.98    1401.95    1401.67    63    HFLDAMPSTVER    41A_8   
		    218    709.98    1417.96    1417.67    38    HFLDAMPSTVER +Oxidation (M)    41_7   
		    203    758.51    1515.01    1514.67    83    HSLFDYYGAEDAK    41A_8   
		    462    779.49    2335.44    2335.01    98    IQVSPLDCTGCGNCADICPAK    41_7   
		    494    1020.73    2039.45    2038.93    89    IVDAMTELVSMDICEDAK    41_1   
		    416    1036.68    2071.35    2070.92    54    IVDAMTELVSMDICEDAK +2 Oxidation (M)    41_8   
		    415    1028.72    2055.43    2054.92    64    IVDAMTELVSMDICEDAK +Oxidation (M)    41_8   
		    277    938.68    1875.34    1874.99    103    IVNMNYAAVDAGINALVK    41A_1   
		    478    946.71    1891.41    1890.99    83    IVNMNYAAVDAGINALVK +Oxidation (M)    41_1   
		    223    822.56    1643.10    1642.76    80    KHSLFDYYGAEDAK    41A_8   
		    181    663.94    1325.86    1325.71    46    LAEIIPEEDAVK    41_7   
		    102    528.88    1055.75    1055.57    63    LGQEIGLGNR    41_7   
		    173    466.05    1395.12    1394.71    41    LPFIHFFDGFR    41A_7   
		    494    1202.91    2403.80    2403.22    48    NGFTVGIVDDVTNTSLTPSEPIK    41_8   
		    87    461.32    920.62    920.46    41    NPFTLDSK    41_8   
		    440    1095.86    2189.71    2188.96    76    QPLMEFSGACAGCGETAYIK    41_8   
		    331    1103.71    2205.41    2204.95    55    QPLMEFSGACAGCGETAYIK +Oxidation (M)    41A_8   
		    69    529.85    1057.69    1057.53    44    SGGITMSHLR    41A_8   
		    139    537.85    1073.68    1073.53    57    SGGITMSHLR +Oxidation (M)    41_8   
		    243    881.65    1761.29    1760.89    70    TKEPGSTGEPLYLDVR    41A_8   
		    556    703.80    2108.37    2107.99    25    TVANEAQAVACGYWHLYR    biorep41Cdiff_4   
		    170    689.04    1376.06    1375.70    42    TVFDNLVSEQPK    41A_7   
		    208    518.03    1551.06    1550.86    35    VAGELLPGVFHVSAR    41A_8   
		    72    536.85    1071.68    1072.56    36    VDVMPANTVK    41A_8   
		    78    545.37    1088.73    1088.55    36    VDVMPANTVK +Oxidation (M)    41A_8   
		    590    1285.79    2569.57    2569.27    75    VELLENEDYASLLNFEAVQAFR    41_1   
		    319    815.59    1629.17    1628.77    68    VEVPASWENAVDADK    41_8   
		    276    953.71    1905.41    1904.89    55    VVELLEKPACDCTDEK    41A_8   
		    66    525.86    1049.71    1049.55    48    VVTQLYGDR    41A_8   
		    165    685.94    1369.87    1369.58    69    YAQAYFDYDSK    41A_8   
		    198    749.94    1497.87    1497.68    69    YAQAYFDYDSKK    41A_8   
		    144    663.96    1325.90    1324.70    34    YYQNIVGIVEK    41A_8   
   
      Matching Genes:  
               gi|115251733|emb|CAJ69568.1|  (pyruvate-flavodoxin oxidoreductase [Clostridium difficile 630]) 
           
  Protein Group 2   
      Expression Quality:  
         Score      Num Spectra      Num Peptides      High-Qual Peptides      % Coverage       1261    47    22    19    38   
   
      Peptides:   
        Query    Observed    Mr(expt)    Mr(calc)    Score    Peptide    Result File   
		    366    1086.31    2170.61    2170.04    55    ATYTMIFDHYEQVPASVAK    41A_7   
		    168    682.00    1361.99    1361.74    53    EDSFIGIIDLLK    41A_7   
		    197    679.45    1356.88    1356.64    58    GGVEPQSENVWR    41_7   
		    131    580.47    1158.93    1158.55    52    GILADGEEAER    biorep41Cdiff_7   
		    122    625.96    1249.91    1249.67    60    GNAVLLEPYFK    41A_7   
		    261    783.05    1564.08    1563.72    83    HSSDEEPFSALAFK    41_7   
		    363    713.48    2137.41    2136.87    53    IGETHEGASQMDWMEQEK +2 Oxidation (M)    41A_7   
		    105    604.41    1206.81    1206.64    44    ILFYTGQTHK    41A_7   
		    107    542.33    1082.64    1082.57    55    ILQMHANTR    41_7   
		    95    512.30    1022.59    1022.51    37    IMTDPFVGK +Oxidation (M)    41_7   
		    148    625.43    1248.84    1248.62    58    LAEEDPTFTVK    41_7   
		    189    741.54    1481.06    1480.81    26    LNSNAVPMQLPIGK    biorep41CdiffA_7   
		    241    749.53    1497.05    1496.80    51    LNSNAVPMQLPIGK +Oxidation (M)    41_7   
		    259    878.68    1755.34    1754.80    74    LVESVAETDEELMMK +2 Oxidation (M)    41A_7   
		    93    510.29    1018.56    1019.50    39    QAETYGVPR    41_7   
		    442    1251.88    2501.75    2501.23    58    SGAQVINAFVPLSEMFGYSTDLR    41A_7   
		    497    1259.87    2517.72    2517.22    64    SGAQVINAFVPLSEMFGYSTDLR +Oxidation (M)    41_7   
		    99    587.87    1173.73    1173.57    59    VAPQEPGEGYK    41A_7   
		    442    1129.78    2257.54    2256.99    56    VEVVTPEDYMGDVMGDLNSR +2 Oxidation (M)    41_7   
		    77    480.80    959.59    959.52    58    VGAPQVAYR    41_7   
		    306    895.16    1788.31    1787.89    95    VYSGTLESGSYVLNATK    41_7   
		    217    776.07    1550.13    1550.77    73    YLEGEELTIDELK    41A_7   
   
      Matching Genes:  
               gi|115249074|emb|CAJ66885.1|  (translation elongation factor G [Clostridium difficile 630]) 
           
  Protein Group 3   
      Expression Quality:  
         Score      Num Spectra      Num Peptides      High-Qual Peptides      % Coverage       1259    52    21    20    44   
   
      Peptides:   
        Query    Observed    Mr(expt)    Mr(calc)    Score    Peptide    Result File   
		    342    680.82    2039.44    2038.93    76    AAQEQQAAQGAEQAQDNGPK    41A_7   
		    323    927.10    1852.19    1851.80    61    DDNVVDADFKEVDEDK    41_7   
		    165    681.48    1360.95    1360.65    55    DNQDATAEELKK    41A_7   
		    402    1065.26    2128.51    2128.03    60    EKIEAFNQAESTIYQTEK    41_7   
		    478    1204.93    2407.85    2407.19    58    ELSSTMSSNINLPFITATAEGPK    41_7   
		    479    1212.93    2423.85    2423.19    84    ELSSTMSSNINLPFITATAEGPK +Oxidation (M)    41_7   
		    133    643.48    1284.95    1284.68    44    FQLTDIPPAQR    41A_7   
		    80    484.34    966.66    966.52    38    HLNIDLSR    41_7   
		    340    936.96    1871.90    1870.89    57    IEAFNQAESTIYQTEK    41_7   
		    286    839.59    1677.17    1676.84    57    IINEPTAAALAYGMDK    41_7   
		    250    847.68    1693.34    1692.84    54    IINEPTAAALAYGMDK +Oxidation (M)    41A_7   
		    76    477.87    953.72    953.55    43    IPAVQEAVK    41_7   
		    134    617.91    1233.80    1233.61    57    ISSGEKEDIEK    41_7   
		    348    946.23    1890.45    1889.96    123    ITITSNTNLSEAEIEQK    41_7   
		    500    978.50    2932.49    2931.41    54    SDAESYLGQTVTEAVITVPAYFTDAQR    41A_7   
		    487    834.21    2499.60    2499.24    55    SQIFSTAADNQTAVDIHVLQGER    41_7   
		    225    796.03    1590.05    1589.87    52    SYTPQEISAIILQK    41A_7   
		    467    1187.86    2373.71    2373.20    80    TALQDAGLSTGDIDDVLLVGGSTR    41_7   
		    41    445.35    888.69    888.46    46    TLNELGDK    41A_7   
		    101    525.86    1049.70    1049.58    61    TTPSVVAFTK    41_7   
		    67    506.83    1011.65    1011.47    44    VSQEMYQK    41A_7   
   
      Matching Genes:  
               gi|115251515|emb|CAJ69348.1|  (chaperone protein [Clostridium difficile 630]) 
           
  Protein Group 4   
      Expression Quality:  
         Score      Num Spectra      Num Peptides      High-Qual Peptides      % Coverage       1136    52    20    16    29   
   
      Peptides:   
        Query    Observed    Mr(expt)    Mr(calc)    Score    Peptide    Result File   
		    509    998.39    2992.15    2992.50    30    ANFVAIPTSAGTGSEVTPFAVITDQDTGVK    41A_7   
		    162    681.50    1360.99    1360.69    56    AVTDLLDEMNIK    41A_8   
		    395    989.75    1977.50    1976.95    51    EFNPDVIISIGGGSAMDAGK    41_8   
		    399    997.73    1993.45    1992.95    41    EFNPDVIISIGGGSAMDAGK +Oxidation (M)    41_8   
		    76    498.43    994.85    994.47    37    ENMLWFR    biorep41Cdiff_7   
		    134    533.82    1065.62    1065.46    47    ETGMGIAEDK +Oxidation (M)    41_8   
		    38    433.27    864.52    864.40    49    FNSSDAPK    41_7   
		    253    688.52    1375.03    1374.78    48    FQSLLVAIEDLK    41_8   
		    408    1011.33    2020.65    2019.92    68    HTTFFEVEPDPTLECAK    41_8   
		    184    714.92    1427.82    1427.69    79    IFATYSQEQVDK    41A_8   
		    385    1019.29    2036.57    2036.06    103    ILINTPSSQGGIGDLYNFK    41_7   
		    157    581.43    1160.84    1160.62    86    IVGQTACTIAK    41_8   
		    160    588.41    1174.81    1174.62    73    LAGFEVPVDTK    41_8   
		    374    1161.76    2321.51    2321.09    40    LVEDGGFGHTSSLYIDDVNQR    41A_8   
		    276    818.52    1635.02    1634.74    63    NHYASEYIYNAYK    41_7   
		    124    516.37    1030.72    1030.60    86    TAVNSILVSK    41_8   
		    696    710.33    2127.96    2127.07    25    VLIGEVESVEIEEAFAHEK    biorep41Cdiff_2   
		    104    485.84    969.66    969.57    58    VPLAIMAQK    41_8   
		    62    493.82    985.64    985.56    29    VPLAIMAQK +Oxidation (M)    41A_7   
		    90    570.37    1138.73    1138.64    67    YAGIASFLGLK    41A_8   
   
      Matching Genes:  
               gi|115252023|emb|CAJ69859.1|  (aldehyde-alcohol dehydrogenase [includes: alcohol dehydrogenase and pyruvate-formate-lyase deactivase [Clostridium difficile 630]) 
              Other Genes Matching Peptide Subset:  
               gi|115249343|emb|CAJ67156.1|  (aldehyde-alcohol dehydrogenase [includes: alcohol dehydrogenase; acetaldehyde dehydrogenase [acetylating]; pyruvate-formate-lyase deactivase [Clostridium difficile 630]) 
           
  Protein Group 5   
      Expression Quality:  
         Score      Num Spectra      Num Peptides      High-Qual Peptides      % Coverage       1100    56    18    15    44   
   
      Peptides:   
        Query    Observed    Mr(expt)    Mr(calc)    Score    Peptide    Result File   
		    47    498.25    994.48    994.52    61    AEAHIQAGAK    41A_5   
		    69    505.93    1009.85    1009.65    58    AIGLVIPSLK    41_5   
		    122    619.94    1237.87    1237.64    89    DKAEAHIQAGAK    41_5   
		    41    468.33    934.65    934.48    39    FNGEIEVK    41A_5   
		    520    808.95    2423.84    2423.15    55    GLMTTIHAYTNDQNTLDGPHPK    41_6   
		    443    814.23    2439.67    2439.15    60    GLMTTIHAYTNDQNTLDGPHPK +Oxidation (M)    41A_4   
		    128    650.18    1298.34    1297.76    66    KVVISAPATGDLK    biorep41CdiffA_5   
		    30    430.52    859.03    858.48    32    MLAHLFK    biorep41Cdiff_4   
		    35    438.35    874.68    874.47    40    MLAHLFK +Oxidation (M)    41_5   
		    434    1163.43    2324.85    2324.10    81    MMEQQDKFEVVAINDLTDAK    41_5   
		    448    1179.39    2356.76    2356.09    69    MMEQQDKFEVVAINDLTDAK +2 Oxidation (M)    41_5   
		    443    1171.42    2340.83    2340.10    103    MMEQQDKFEVVAINDLTDAK +Oxidation (M)    41_5   
		    335    659.90    1317.79    1317.66    63    NVTVEEINAAMK    41_2   
		    131    556.54    1111.07    1110.61    38    TLGYFAQLAK    biorep41Cdiff_5   
		    226    591.79    1772.35    1771.98    66    VPVVTGSITELVCTLGK    41A_5   
		    190    585.91    1169.81    1169.67    54    VVISAPATGDLK    41_3   
		    363    1046.23    2090.45    2089.98    61    VVSWYDNEMSYTSQLIR    41A_6   
		    389    1054.22    2106.42    2105.97    65    VVSWYDNEMSYTSQLIR +Oxidation (M)    41A_4   
   
      Matching Genes:  
               gi|115252231|emb|CAJ70071.1|  (glyceraldehyde-3-phosphate dehydrogenase 2 [Clostridium difficile 630]) 
           
  Protein Group 6   
      Expression Quality:  
         Score      Num Spectra      Num Peptides      High-Qual Peptides      % Coverage       1078    48    22    17    50   
   
      Peptides:   
        Query    Observed    Mr(expt)    Mr(calc)    Score    Peptide    Result File   
		    281    964.75    1927.49    1927.01    62    DKYPGLIFSQILGYGEK    41A_5   
		    39    462.82    923.62    923.51    36    EGVEILHK    41A_5   
		    180    697.98    1393.95    1393.65    36    ENPNSPLMTTYK    41_5   
		    183    705.92    1409.83    1409.65    29    ENPNSPLMTTYK +Oxidation (M)    41A_6   
		    50    511.28    1020.54    1020.49    60    GGVSQSVMEK    41A_5   
		    111    549.89    1097.76    1097.57    43    IEPIEGDGIR    41_6   
		    489    1278.55    2555.09    2556.15    73    IQSCEDLLDDEQAWANDFLFK    41_5   
		    65    495.38    988.75    988.53    40    IVGEAMLEK    41_5   
		    205    739.03    1476.05    1475.80    58    LLSEADIFVTNVR    41A_6   
		    55    519.98    1037.95    1037.52    28    MGIAYDQIK    biorep41CdiffA_5   
		    87    609.91    1217.81    1217.61    61    MLGDWGAEVIK    41A_5   
		    97    618.03    1234.04    1233.61    48    MLGDWGAEVIK +Oxidation (M)    41A_5   
		    88    570.44    1138.87    1138.63    55    SKEGVEILHK    41_5   
		    42    470.81    939.60    939.42    30    SLGYDEEK    41A_5   
		    321    997.73    1993.45    1992.84    55    SPASDDENPMFELENGNK    41_5   
		    304    1005.63    2009.24    2008.83    55    SPASDDENPMFELENGNK +Oxidation (M)    41A_5   
		    365    1061.79    2121.57    2120.93    47    SPASDDENPMFELENGNKK    41_5   
		    345    1069.68    2137.34    2136.93    58    SPASDDENPMFELENGNKK +Oxidation (M)    41A_5   
		    407    703.01    2106.01    2105.02    56    TLDEWSALLEEADLPFEK    biorep41CdiffA_5   
		    89    555.41    1108.80    1108.62    48    VGQHTVEVLK    41A_6   
		    202    826.62    1651.22    1650.81    52    VVELSSFIAAPCCAK    41A_5   
		    274    695.69    1389.37    1388.78    48    WIQLALIQYNK    biorep41Cdiff_5   
   
      Matching Genes:  
               gi|115249401|emb|CAJ67216.1|  (isocaprenoyl-CoA:2-hydroxyisocaproate CoA-transferase [Clostridium difficile 630]) 
           
  Protein Group 7   
      Expression Quality:  
         Score      Num Spectra      Num Peptides      High-Qual Peptides      % Coverage       1022    56    19    14    48   
   
      Peptides:   
        Query    Observed    Mr(expt)    Mr(calc)    Score    Peptide    Result File   
		    35    440.98    879.94    879.45    32    AFESIWK    biorep41Cdiff_5   
		    90    556.94    1111.86    1111.57    38    ALSIWMTFK +Oxidation (M)    41A_6   
		    86    598.76    1195.51    1194.57    34    CSVTGIPYGGGK    biorep41CdiffA_5   
		    336    748.11    1494.21    1493.72    47    DVNVFEMAQSQVK    biorep41Cdiff_5   
		    215    756.01    1510.01    1509.71    66    DVNVFEMAQSQVK +Oxidation (M)    41A_6   
		    77    525.32    1048.62    1048.50    51    EAAYMHSIK    41A_6   
		    106    533.34    1064.66    1064.50    39    EAAYMHSIK +Oxidation (M)    41_6   
		    288    886.15    1770.29    1769.92    60    GGIIVDPSTLSQGELER    41A_6   
		    28    464.72    927.42    927.47    26    GYIDGIYK    biorep41CdiffA_5   
		    396    1011.83    2021.64    2021.03    82    IAVQGIGNVGSYTVLNCEK    41_6   
		    191    641.94    1281.87    1281.64    47    IKEEYNVTMR    41_6   
		    199    649.95    1297.89    1297.63    41    IKEEYNVTMR +Oxidation (M)    41_6   
		    232    682.01    1362.00    1361.73    44    LGMEPAVYELLK    41_6   
		    238    690.12    1378.23    1377.72    61    LGMEPAVYELLK +Oxidation (M)    41_6   
		    411    1130.86    2259.71    2259.22    53    LTGQSSIGVITGKPVEFGGSLGR    41_5   
		    481    1138.88    2275.75    2275.04    76    LVCEAANGPTTPEADEVFAER    41_6   
		    492    1285.47    2568.93    2568.11    87    SEGSYAIYNENGLDGQAMLDYMK    41_5   
		    150    611.45    1220.89    1220.65    83    TAATGFGVAVTAR    41_6   
		    61    492.43    982.85    982.61    55    VIEVSIPVK    41A_6   
   
      Matching Genes:  
               gi|115249189|emb|CAJ67001.1|  (NAD-specific glutamate dehydrogenase [Clostridium difficile 630]) 
           
  Protein Group 8   
      Expression Quality:  
         Score      Num Spectra      Num Peptides      High-Qual Peptides      % Coverage       960    48    16    15    40   
   
      Peptides:   
        Query    Observed    Mr(expt)    Mr(calc)    Score    Peptide    Result File   
		    661    847.84    2540.48    2539.30    89    AYEGGFAIGAFNISDLEQLQGVLK    41_4   
		    77    530.39    1058.77    1058.56    62    DAIQAVVESK    41A_4   
		    222    611.42    1220.83    1220.63    56    FDILEEIQSK    41_4   
		    12    409.75    817.49    817.43    41    FLAENPK    41_4   
		    60    489.32    976.63    976.46    55    INMDTDLR    41A_4   
		    65    497.31    992.60    992.46    46    INMDTDLR +Oxidation (M)    41A_4   
		    670    890.19    2667.56    2667.39    91    KAYEGGFAIGAFNISDLEQLQGVLK    41_4   
		    58    473.82    945.63    945.53    47    KFLAENPK    41A_4   
		    21    423.87    845.72    845.48    34    LAMTAAIR    biorep41Cdiff_4   
		    65    431.77    861.53    861.47    43    LAMTAAIR +Oxidation (M)    41_4   
		    419    781.00    1559.98    1559.73    65    NSYVMIQASMSAVK +2 Oxidation (M)    41_4   
		    415    772.94    1543.86    1543.74    61    NSYVMIQASMSAVK +Oxidation (M)    41_4   
		    478    873.11    1744.20    1743.92    73    TGVDSLAIAIGTSHGAFK    41_4   
		    329    672.94    1343.86    1343.69    77    YAGPHTLVEMVK    41_4   
		    341    680.93    1359.84    1359.69    55    YAGPHTLVEMVK +Oxidation (M)    41_4   
		    400    770.00    1537.98    1537.74    65    YTQPAEAVEFVER    41_3   
   
      Matching Genes:  
               gi|115249409|emb|CAJ67224.1|  (putative fructose-bisphosphate aldolase [Clostridium difficile 630]) 
           
  Protein Group 9   
      Expression Quality:  
         Score      Num Spectra      Num Peptides      High-Qual Peptides      % Coverage       891    33    15    15    39   
   
      Peptides:   
        Query    Observed    Mr(expt)    Mr(calc)    Score    Peptide    Result File   
		    646    1059.03    3174.08    3173.59    97    ALEAANMTIEDIDLVEANEAFAAQSVAVIR    41_2   
		    609    1064.39    3190.15    3189.59    73    ALEAANMTIEDIDLVEANEAFAAQSVAVIR +Oxidation (M)    41_5   
		    531    1370.61    2739.20    2738.43    67    ANITPDMIDESLLGGVLTAGLGQNIAR    41_5   
		    537    1378.46    2754.91    2754.42    71    ANITPDMIDESLLGGVLTAGLGQNIAR +Oxidation (M)    41_5   
		    260    936.79    1871.56    1871.00    64    AQAEGKFDEEIVPVVIK    41A_5   
		    428    1148.43    2294.85    2294.09    44    DGTVTAGNASGINDGAAMLVVMAK +2 Oxidation (M)    41_5   
		    423    1140.39    2278.76    2278.09    48    DGTVTAGNASGINDGAAMLVVMAK +Oxidation (M)    41_5   
		    174    737.97    1473.92    1473.69    67    EEQDELALASQNK    41A_5   
		    117    644.46    1286.91    1286.71    55    FDEEIVPVVIK    41A_5   
		    91    613.06    1224.11    1223.68    44    ILTTLLYEMK    41A_5   
		    106    620.99    1239.98    1239.68    47    ILTTLLYEMK +Oxidation (M)    41A_5   
		    71    567.40    1132.79    1132.60    61    IMGYGPVPATK    41A_5   
		    90    575.45    1148.88    1148.59    54    IMGYGPVPATK +Oxidation (M)    41_5   
		    96    616.49    1230.97    1230.68    48    SVSAVELGVTAAK    41A_5   
		    51    521.43    1040.84    1040.53    51    TAVGSFGGAFK    41A_5   
   
      Matching Genes:  
               gi|115250080|emb|CAJ67900.1|  (acetyl-CoA acetyltransferase [Clostridium difficile 630]) 
           
  Protein Group 10   
      Expression Quality:  
         Score      Num Spectra      Num Peptides      High-Qual Peptides      % Coverage       857    26    15    11    33   
   
      Peptides:   
        Query    Observed    Mr(expt)    Mr(calc)    Score    Peptide    Result File   
		    79    529.86    1057.71    1057.60    39    AVTVAVEELK    41A_6   
		    550    883.97    2648.90    2648.36    45    EMLQDIAILTGAQVISEELGYDLK    41_6   
		    166    668.96    1335.90    1335.65    47    ESTTIVDGSGDKK    41A_6   
		    283    767.05    1532.08    1531.82    59    FGSPLITNDGVTIAK    41_6   
		    194    724.52    1447.03    1446.67    55    GFVSAYMVTDVDK +Oxidation (M)    41A_6   
		    53    468.32    934.62    934.51    38    GTFDVVAVK    41A_6   
		    65    501.84    1001.67    1001.51    60    IEDALNATR    41A_6   
		    604    717.21    2148.62    2148.22    70    ISNIQELLPVLEQIVQQGK    41_4   
		    79    478.38    954.75    954.55    39    KALEEPLR    41_6   
		    7    407.35    812.69    812.51    52    LAGGVAVVK    41_6   
		    132    587.42    1172.82    1172.65    65    LIAEAMEIVGK    41_6   
		    111    595.40    1188.78    1188.64    38    LIAEAMEIVGK +Oxidation (M)    41A_6   
		    166    619.93    1237.84    1237.71    53    NVTAGANPILLR    41_6   
		    412    1044.78    2087.54    2087.09    118    TNDVAGDGTTTATVLAQAIIR    41_6   
		    72    508.85    1015.68    1015.55    79    VGAATEVELK    41A_6   
   
      Matching Genes:  
               gi|115249204|emb|CAJ67016.1|  (60 kDa chaperonin [Clostridium difficile 630]) 
           
  Protein Group 11   
      Expression Quality:  
         Score      Num Spectra      Num Peptides      High-Qual Peptides      % Coverage       857    34    12    11    34   
   
      Peptides:   
        Query    Observed    Mr(expt)    Mr(calc)    Score    Peptide    Result File   
		    405    683.22    2046.64    2046.08    63    AAADEIGLPLFQYLGGVNAK    41_6   
		    108    537.90    1073.78    1073.56    69    AGYTAVISHR    41_6   
		    245    888.08    1774.15    1773.93    89    AIVPSGASTGAFEAVELR    41A_8   
		    212    664.95    1327.89    1327.76    75    EALELIVEAITK    41_6   
		    207    657.60    1313.18    1312.73    60    GIENGVANSILVK    41_6   
		    127    581.42    1160.82    1160.55    52    IEEMVGEQAR    41_6   
		    133    589.39    1176.77    1176.54    72    IEEMVGEQAR +Oxidation (M)    41_6   
		    60    482.81    963.60    963.54    56    KYVLAGEGK    41A_6   
		    279    860.62    1719.22    1718.88    101    LQLVGDDLFVTNTER    41A_6   
		    116    559.84    1117.67    1117.56    64    MGAEVFHSLK    41_6   
		    439    1095.42    2188.82    2188.09    118    SGETEDSTIADLAVAVNAGQIK    41_6   
		    104    525.43    1048.85    1048.59    38    SVIELVYAR    41_6   
   
      Matching Genes:  
               gi|115252227|emb|CAJ70067.1|  (enolase [Clostridium difficile 630]) 
           
  Protein Group 12   
      Expression Quality:  
         Score      Num Spectra      Num Peptides      High-Qual Peptides      % Coverage       855    26    16    13    31   
   
      Peptides:   
        Query    Observed    Mr(expt)    Mr(calc)    Score    Peptide    Result File   
		    509    1342.39    2682.77    2682.27    69    AEVTDVANAIYDGTDAIMLSGETAAGK    41_7   
		    95    574.41    1146.81    1146.55    50    DGEVVTVDASR    41A_7   
		    297    581.01    1740.01    1739.78    42    FNFSHGSHEEHKER    41_7   
		    328    928.22    1854.42    1853.94    42    GDLGVEIPTEEMPIVQK    41_7   
		    300    936.29    1870.57    1869.94    26    GDLGVEIPTEEMPIVQK +Oxidation (M)    41A_7   
		    299    872.13    1742.25    1741.87    70    IENQEGVENLDEILK    41_7   
		    56    483.91    965.80    965.59    42    INLPAITPK    41A_7   
		    59    486.85    971.68    971.58    41    KASDVLAIR    41A_7   
		    138    649.40    1296.78    1296.59    43    RTEETLDYDR    41A_7   
		    209    765.47    1528.92    1528.81    71    SGDSILIDDGLVGLR    41A_7   
		    125    594.39    1186.77    1186.62    64    SPIIATTNNEK    41_7   
		    194    674.96    1347.91    1347.65    102    SSVAGNTDEVIEK    41_7   
		    94    571.33    1140.65    1140.49    28    TEETLDYDR    41A_7   
		    281    920.16    1838.30    1837.84    80    TGNFEDPEVFLEEGQK    41A_7   
		    74    474.33    946.66    946.49    59    VSDGIMVAR    41_7   
		    1    403.32    804.63    804.44    26    YPVEAVK    41A_7   
   
      Matching Genes:  
               gi|115252454|emb|CAJ70297.1|  (pyruvate kinase [Clostridium difficile 630]) 
           
  Protein Group 13   
      Expression Quality:  
         Score      Num Spectra      Num Peptides      High-Qual Peptides      % Coverage       809    34    15    11    45   
   
      Peptides:   
        Query    Observed    Mr(expt)    Mr(calc)    Score    Peptide    Result File   
		    321    904.61    1807.20    1807.91    124    AFAGADTWATSSALAGALK    41A_4   
		    34    415.78    829.54    829.45    61    AGLEEAIK    41_4   
		    80    536.37    1070.72    1070.63    30    AGLEEAIKLK    41A_4   
		    258    635.42    1268.83    1268.62    42    DGVPSIINPDDK    41_4   
		    382    1041.31    2080.61    2080.07    53    DGVPSIINPDDKAGLEEAIK    41A_4   
		    154    650.44    1298.87    1298.67    56    DIEVDPSNLGLK    41A_4   
		    118    467.23    932.45    932.44    39    EALAMGADR    41_4   
		    126    475.26    948.50    948.43    42    EALAMGADR +Oxidation (M)    41_4   
		    184    550.37    1098.73    1098.60    48    LDPNTGTLIR    41_4   
		    175    538.85    1075.68    1075.58    57    MPCLITTLK    41_4   
		    84    546.88    1091.74    1091.57    57    MPCLITTLK +Oxidation (M)    41A_4   
		    68    508.78    1015.55    1015.52    35    QVPDTTEVK    41A_4   
		    399    746.98    1491.95    1491.76    73    SVKPAGTIYNEDAK    41_4   
		    159    519.34    1036.66    1036.54    39    TEGEYVLVK    41_4   
		    110    459.33    916.65    916.52    53    TSAGIIIDK    41_4   
   
      Matching Genes:  
               gi|115250076|emb|CAJ67896.1|  (electron transfer flavoprotein beta-subunit [Clostridium difficile 630]) 
           
  Protein Group 14   
      Expression Quality:  
         Score      Num Spectra      Num Peptides      High-Qual Peptides      % Coverage       785    37    14    12    40   
   
      Peptides:   
        Query    Observed    Mr(expt)    Mr(calc)    Score    Peptide    Result File   
		    448    1286.36    2570.71    2570.27    87    DILDILEDNNISVVADDLAQETR    41A_5   
		    243    856.60    1711.19    1710.77    57    ELEEICGYEIEEAK    41_5   
		    94    585.49    1168.97    1168.61    51    EVVENPNAAVK    41_5   
		    210    852.09    1702.17    1701.73    56    FCDPEEYDYPLVR    41A_5   
		    35    480.30    958.59    958.53    27    GSLIVDEVK    biorep41CdiffA_5   
		    91    576.93    1151.84    1151.64    53    HSNTIKPSIR    41_5   
		    187    763.48    1524.94    1524.73    71    IHESIEVYNEHR    41A_5   
		    145    675.89    1349.76    1349.60    43    LNAMPEEVCSGK +Oxidation (M)    41A_5   
		    188    715.05    1428.08    1427.74    51    MKEVVENPNAAVK    41_5   
		    169    723.02    1444.03    1443.74    80    MKEVVENPNAAVK +Oxidation (M)    41A_5   
		    256    884.65    1767.28    1766.79    69    QWSNIEGCSLAYDPK    41_5   
		    129    629.93    1257.85    1257.58    37    TDVPAGDDALER    41_5   
		    69    565.33    1128.65    1128.68    52    VLLTGILADSK    biorep41CdiffA_5   
		    94    613.91    1225.81    1225.66    51    YISLVHPQNR    41A_5   
   
      Matching Genes:  
               gi|115249404|emb|CAJ67219.1|  (subunit of oxygen-sensitive 2-hydroxyisocaproyl-CoA dehydratase [Clostridium difficile 630]) 
           
  Protein Group 15   
      Expression Quality:  
         Score      Num Spectra      Num Peptides      High-Qual Peptides      % Coverage       778    78    12    9    61   
   
      Peptides:   
        Query    Observed    Mr(expt)    Mr(calc)    Score    Peptide    Result File   
		    86    452.31    902.60    902.53    53    AFLGLLNR    41A_2   
		    210    628.30    1254.59    1254.58    67    EGYPEVAEAYK    41A_2   
		    581    780.45    2338.34    2338.07    34    ELGLDAIHDTVHEMCKDEAR    41_3   
		    219    746.06    1490.10    1489.80    101    FAELLGEVVVADTK    41A_3   
		    303    615.49    1228.96    1228.53    33    GEMVWADEHR    biorep41Cdiff_3   
		    204    416.19    1245.55    1244.52    29    GEMVWADEHR +Oxidation (M)    41A_2   
		    241    608.41    1214.80    1214.59    60    IAFEEAEHAAK    41_2   
		    422    820.60    1639.19    1638.89    107    IGVAQGVDAEIIEGLR    41_1   
		    177    563.31    1124.60    1124.50    72    VDAEYGATDGK    41_3   
		    158    577.26    1152.51    1152.55    72    VGADKFEEMK    41A_2   
		    230    585.31    1168.61    1168.54    50    VGADKFEEMK +Oxidation (M)    41_1   
		    376    690.98    1379.95    1379.67    100    VRVDAEYGATDGK    41_2   
   
      Matching Genes:  
               gi|115250565|emb|CAJ68389.1|  (putative rubrerythrin [Clostridium difficile 630]) 
           
  Protein Group 16   
      Expression Quality:  
         Score      Num Spectra      Num Peptides      High-Qual Peptides      % Coverage       714    48    14    11    44   
   
      Peptides:   
        Query    Observed    Mr(expt)    Mr(calc)    Score    Peptide    Result File   
		    598    743.00    2225.99    2225.15    49    DAIDEIKPEIMLFGATHIGR    biorep41Cdiff_5   
		    75    497.09    992.17    992.52    26    ELITFGADK    biorep41Cdiff_7   
		    201    683.52    1365.03    1364.80    80    IAPVVIELLGEGR    41_7   
		    26    430.75    859.49    859.44    50    ITQDDIR    41A_4   
		    137    666.00    1329.99    1329.70    61    LDSVDDLLEAIK    41A_5   
		    281    701.64    1401.26    1400.74    50    LDSVDDLLEAIKA    biorep41Cdiff_5   
		    180    543.85    1085.68    1085.56    41    LEIDPEDKK    41_4   
		    15    416.35    830.68    830.46    53    LGGVVGSSR    41A_7   
		    494    1011.86    3032.56    3031.59    26    NPAAPILEIADYGVVGDLHEIVPMLIEK +Oxidation (M)    41A_4   
		    93    581.99    1161.96    1161.59    43    NVWIFAEQR    41_5   
		    77    554.92    1107.83    1107.58    92    TGEVIALDYK    41_5   
		    94    445.77    889.53    889.51    42    TTVLETVK    41_4   
		    83    561.84    1121.66    1121.54    72    VGTGLTADCTK    41_5   
		    130    481.78    961.55    961.44    29    YTTDAYTK    41_4   
   
      Matching Genes:  
               gi|115249407|emb|CAJ67222.1|  (electron transfer flavoprotein alpha-subunit [Clostridium difficile 630]) 
              Other Genes Matching Peptide Subset:  
               gi|115249822|emb|CAJ67639.1|  (electron transfer flavoprotein alpha-subunit [Clostridium difficile 630]) 
           
  Protein Group 17   
      Expression Quality:  
         Score      Num Spectra      Num Peptides      High-Qual Peptides      % Coverage       703    25    14    11    32   
   
      Peptides:   
        Query    Observed    Mr(expt)    Mr(calc)    Score    Peptide    Result File   
		    78    528.36    1054.71    1054.51    39    AFDITFADR    41A_6   
		    326    880.16    1758.30    1757.88    60    ALQSGTSHFLGQHFTK    41_6   
		    170    676.91    1351.80    1351.62    49    DIENNQAMVFR +Oxidation (M)    41A_6   
		    86    548.83    1095.66    1095.49    59    EADSMVVMAK +Oxidation (M)    41A_6   
		    418    1132.75    2263.48    2263.07    61    EAEHVEGFAPEVAWVTHGGNK    41A_6   
		    272    850.04    1698.06    1697.72    41    EDNTSIVENMDEFR    41A_6   
		    275    858.02    1714.03    1713.72    49    EDNTSIVENMDEFR +Oxidation (M)    41A_6   
		    354    931.18    1860.35    1859.86    61    EGNLANPYHTSWGASTR    41_6   
		    115    559.38    1116.75    1116.61    47    IKEETGATIR    41_6   
		    118    560.90    1119.78    1119.56    51    KGNVMETVDK    41_6   
		    326    944.53    1887.05    1886.84    37    MEDDFPQWYTDVITK    41A_6   
		    367    952.61    1903.21    1902.83    59    MEDDFPQWYTDVITK +Oxidation (M)    41_6   
		    117    560.86    1119.70    1119.58    59    TDLVDYAPVK    41_6   
		    184    709.56    1417.11    1416.91    31    VAPIQVVIVPIAAK    41A_6   
   
      Matching Genes:  
               gi|115249053|emb|CAJ66864.1|  (putative dual-specificity prolyl/cysteinyl-tRNA synthetase [Clostridium difficile 630]) 
           
  Protein Group 18   
      Expression Quality:  
         Score      Num Spectra      Num Peptides      High-Qual Peptides      % Coverage       688    65    10    8    56   
   
      Peptides:   
        Query    Observed    Mr(expt)    Mr(calc)    Score    Peptide    Result File   
		    86    452.31    902.60    902.53    53    AFLGLLNR    41A_2   
		    134    621.39    1240.76    1240.56    38    EGYPEVGEAYK    41A_3   
		    581    780.45    2338.34    2338.07    34    ELGLDAIHDTVHEMCKDEAR    41_3   
		    219    746.06    1490.10    1489.80    101    FAELLGEVVVADTK    41A_3   
		    192    591.38    1180.75    1180.61    55    IALEEAEHAAK    41_3   
		    434    849.63    1697.25    1696.90    113    IGVAQGVDEEIIEGLR    41_1   
		    177    563.31    1124.60    1124.50    72    VDAEYGATDGK    41_3   
		    158    577.26    1152.51    1152.55    72    VGADKFEEMK    41A_2   
		    230    585.31    1168.61    1168.54    50    VGADKFEEMK +Oxidation (M)    41_1   
		    376    690.98    1379.95    1379.67    100    VRVDAEYGATDGK    41_2   
   
      Matching Genes:  
               gi|115250515|emb|CAJ68339.1|  (putative ruberythrin [Clostridium difficile 630]) 
           
  Protein Group 19   
      Expression Quality:  
         Score      Num Spectra      Num Peptides      High-Qual Peptides      % Coverage       686    20    13    9    17   
   
      Peptides:   
        Query    Observed    Mr(expt)    Mr(calc)    Score    Peptide    Result File   
		    179    484.16    1449.46    1449.69    43    AGAPFAPGANPMHGR    biorep41CdiffA_7   
		    273    489.76    1466.26    1465.69    38    AGAPFAPGANPMHGR +Oxidation (M)    biorep41Cdiff_7   
		    112    560.33    1118.65    1118.51    32    DQNGAAMSLGR    41_7   
		    57    557.84    1113.67    1113.58    38    EQQLDVINR    biorep41CdiffA_7   
		    226    717.44    1432.87    1432.68    40    ERENGGTLDVDTK    41_7   
		    389    1033.31    2064.61    2064.92    62    FEPITSEYLDYDEVMSK    41_7   
		    394    1041.71    2081.41    2080.92    59    FEPITSEYLDYDEVMSK +Oxidation (M)    41_7   
		    320    616.06    1845.16    1844.86    45    MAESYGFDISKPATNSK    41_7   
		    335    621.41    1861.19    1860.86    53    MAESYGFDISKPATNSK +Oxidation (M)    41_7   
		    395    1043.37    2084.73    2084.08    71    NSYPTQSILTITSNVVYGK    41_7   
		    145    660.43    1318.85    1318.69    34    SGIITGLPDAYGR    41A_7   
		    369    986.69    1971.37    1970.89    99    VSIDTSSVQYENDDLMR    41_7   
		    373    994.67    1987.33    1986.88    72    VSIDTSSVQYENDDLMR +Oxidation (M)    41_7   
   
      Matching Genes:  
               gi|115249776|emb|CAJ67593.1|  (formate acetyltransferase [Clostridium difficile 630]) 
           
  Protein Group 20   
      Expression Quality:  
         Score      Num Spectra      Num Peptides      High-Qual Peptides      % Coverage       662    35    11    7    31   
   
      Peptides:   
        Query    Observed    Mr(expt)    Mr(calc)    Score    Peptide    Result File   
		    147    652.57    1303.12    1302.71    53    ALQLHGGYGFIK    41_5   
		    191    631.55    1261.08    1260.59    38    ELDTLPAEMDK    biorep41Cdiff_5   
		    45    482.83    963.65    963.50    36    GLVYDAAQK    41A_5   
		    92    524.06    1046.11    1045.56    59    IAMGTLEVGR    biorep41Cdiff_7   
		    53    531.88    1061.74    1061.55    92    IAMGTLEVGR +Oxidation (M)    41A_5   
		    344    627.54    1879.61    1879.08    91    IGVAALALGIAQGALDEAVK    41_7   
		    189    727.05    1452.09    1451.75    94    IVSIYEGTSEVQK    41A_7   
		    46    495.52    989.02    989.56    28    MVISSNVLK    biorep41CdiffA_5   
		    46    494.37    986.73    986.59    48    VQFGKPIAK    41A_5   
		    243    675.59    1349.17    1348.57    39    WDGFSTGAHEDK    biorep41Cdiff_5   
		    248    760.99    1519.96    1519.72    84    YYASEIANEVAYK    41_7   
   
      Matching Genes:  
               gi|115249405|emb|CAJ67220.1|  (acyl-CoA dehydrogenase, short-chain specific [Clostridium difficile 630]) 
           
  Protein Group 21   
      Expression Quality:  
         Score      Num Spectra      Num Peptides      High-Qual Peptides      % Coverage       658    15    12    8    23   
   
      Peptides:   
        Query    Observed    Mr(expt)    Mr(calc)    Score    Peptide    Result File   
		    176    657.50    1312.99    1312.80    31    EVIPEFLLLLK    41_7   
		    241    825.53    1649.04    1648.67    48    FAGMDLGMNFEEEK +2 Oxidation (M)    41A_7   
		    295    575.39    1723.14    1722.85    52    FIDNGIGMTEEEIKK    41_7   
		    111    545.00    1087.98    1087.58    37    IDADLSDVLK    biorep41Cdiff_7   
		    126    594.91    1187.81    1187.64    54    LVSLGEISENK    41_7   
		    253    769.53    1537.04    1536.79    68    LYNNQVFVADNIK    41_7   
		    305    895.12    1788.23    1787.87    83    NEDTPAMVLVSEQSIR    41_7   
		    271    902.81    1803.60    1803.87    30    NEDTPAMVLVSEQSIR +Oxidation (M)    41A_7   
		    174    654.92    1307.82    1307.65    38    SFLQNDRDVSK    41_7   
		    278    826.62    1651.23    1650.90    68    VIEPLNDTNPLWLK    41_7   
		    354    965.15    1928.28    1927.84    92    WISEGGTEYEISESDAR    41_7   
		    321    925.66    1849.30    1848.87    57    YINQVAFSGAEDFFNK    41_7   
   
      Matching Genes:  
               gi|115249282|emb|CAJ67095.1|  (chaperone protein (heat shock protein) [Clostridium difficile 630]) 
           
  Protein Group 22   
      Expression Quality:  
         Score      Num Spectra      Num Peptides      High-Qual Peptides      % Coverage       635    27    10    9    47   
   
      Peptides:   
        Query    Observed    Mr(expt)    Mr(calc)    Score    Peptide    Result File   
		    313    664.94    1327.88    1327.73    75    ALENVLKDDLAK    41_4   
		    391    1056.87    2111.73    2111.09    32    ALEVGIDPILCVGETLEQR    41A_4   
		    285    840.03    1678.05    1677.77    65    EIDMDYVVIGHSER +Oxidation (M)    41A_4   
		    112    610.91    1219.81    1219.62    58    GLYGELANEVR    41A_4   
		    288    652.74    1303.46    1302.60    69    IGAQNMHFEEK    41_4   
		    305    660.38    1318.75    1318.60    69    IGAQNMHFEEK +Oxidation (M)    41_4   
		    155    513.84    1025.67    1025.60    49    KPIIAGNWK    41_4   
		    436    808.52    1615.03    1614.75    67    QYFNETDETVNKK    41_4   
		    447    820.02    1638.04    1637.79    85    TATAEDANDVISYIR    41_4   
		    259    802.10    1602.19    1601.88    66    VVVAYEPIWAIGTGK    41A_4   
   
      Matching Genes:  
               gi|115252229|emb|CAJ70069.1|  (triosephosphate isomerase [Clostridium difficile 630]) 
           
  Protein Group 23   
      Expression Quality:  
         Score      Num Spectra      Num Peptides      High-Qual Peptides      % Coverage       629    17    11    6    22   
   
      Peptides:   
        Query    Observed    Mr(expt)    Mr(calc)    Score    Peptide    Result File   
		    16    420.81    839.61    839.49    36    APSIPSLR    41A_6   
		    121    567.86    1133.71    1134.53    35    CGVEVTSIDR    41_6   
		    206    740.98    1479.94    1479.67    69    EMGDNCSVTILNK    41A_6   
		    214    748.92    1495.83    1495.66    79    EMGDNCSVTILNK +Oxidation (M)    41A_6   
		    13    415.74    829.47    829.40    30    FGYTNTK    41A_6   
		    341    918.20    1834.38    1833.82    90    GDDISYAGCGLPYYVGK    41_6   
		    63    495.80    989.58    989.50    38    ICAQNISGK    41A_6   
		    222    764.55    1527.08    1526.85    30    KLLGVQVMGPGAVDK +Oxidation (M)    41A_6   
		    247    705.04    1408.07    1407.72    65    SSLIVNTPESFSK    41_6   
		    293    793.05    1584.08    1583.77    71    TLSTGEKENLSYDK    41_6   
		    262    732.05    1462.08    1461.82    86    VLEGGILFYPNLK    41_6   
   
      Matching Genes:  
               gi|115250843|emb|CAJ68667.1|  (putative pyridine nucleotide-disulfide oxidoreductase [Clostridium difficile 630]) 
           
  Protein Group 24   
      Expression Quality:  
         Score      Num Spectra      Num Peptides      High-Qual Peptides      % Coverage       579    18    12    7    33   
   
      Peptides:   
        Query    Observed    Mr(expt)    Mr(calc)    Score    Peptide    Result File   
		    82    560.95    1119.88    1119.58    43    FEGETLPSLK    41_5   
		    34    440.95    879.88    879.45    37    FMEIVNK    biorep41Cdiff_5   
		    21    424.30    846.59    846.44    33    ILETTDR    41_5   
		    201    731.36    1460.70    1460.73    60    LGWTCASPEILSK    biorep41CdiffA_5   
		    168    613.02    1224.02    1223.55    31    LNYSNMPEEK    biorep41Cdiff_5   
		    125    620.89    1239.76    1239.54    27    LNYSNMPEEK +Oxidation (M)    41_5   
		    230    826.16    1650.31    1649.82    45    MIYVIPDFQNPTGR    41_5   
		    137    560.00    1117.98    1117.55    37    MQGLQGSEIR    biorep41Cdiff_5   
		    266    897.22    1792.42    1791.87    73    QGADLQASTISQMEVSK    41_5   
		    238    905.11    1808.20    1807.86    68    QGADLQASTISQMEVSK +Oxidation (M)    41A_5   
		    457    1204.44    2406.88    2406.20    72    TNVNKDDILVTSGSQQGLDFAGK    41_5   
		    458    1329.42    2656.83    2656.31    53    VFIDEGDVILCESPSYIGAINAFK    41A_5   
   
      Matching Genes:  
               gi|115252729|emb|CAJ70573.1|  (putative amino acid aminotransferase [Clostridium difficile 630]) 
           
  Protein Group 25   
      Expression Quality:  
         Score      Num Spectra      Num Peptides      High-Qual Peptides      % Coverage       571    13    9    8    27   
   
      Peptides:   
        Query    Observed    Mr(expt)    Mr(calc)    Score    Peptide    Result File   
		    223    765.98    1529.95    1529.68    25    ATNFDEVCLGYNK    41A_6   
		    335    895.74    1789.46    1788.89    68    CVGGCPVGIDIPGFITK    41_6   
		    427    1069.25    2136.48    2136.05    55    EGVFAGGDAVTGAATVISAMGAGK    41_6   
		    147    611.32    1220.63    1220.50    42    MELGEPDDSGR +Oxidation (M)    41_6   
		    135    591.38    1180.74    1180.60    79    TAAASIDEYLK    41_6   
		    280    863.72    1725.43    1725.91    90    VAVIGSGPAGLACAGDLAK    41A_6   
		    239    693.99    1385.97    1385.71    92    VAVVGGGNVAMDAAR    41_6   
		    246    701.96    1401.91    1401.70    67    VAVVGGGNVAMDAAR +Oxidation (M)    41_6   
		    345    989.29    1976.56    1975.92    53    VCPQESQCEGVCILGIK    41A_6   
   
      Matching Genes:  
               gi|115250578|emb|CAJ68402.1|  (putative glutamate synthase [NADPH] small chain [Clostridium difficile 630]) 
           
  Protein Group 26   
      Expression Quality:  
         Score      Num Spectra      Num Peptides      High-Qual Peptides      % Coverage       556    26    10    9    37   
   
      Peptides:   
        Query    Observed    Mr(expt)    Mr(calc)    Score    Peptide    Result File   
		    11    416.86    831.71    831.45    60    CLALLDK    41A_4   
		    625    1141.70    2281.39    2281.15    55    EDTILATNTSSLSITEIASSTK    41_4   
		    582    1037.80    2073.58    2073.12    66    ILIPMINEAVGIYADGVASK    41_4   
		    586    1045.79    2089.57    2089.11    56    ILIPMINEAVGIYADGVASK +Oxidation (M)    41_4   
		    58    445.43    888.84    889.46    31    LLDELCK    biorep41Cdiff_4   
		    139    637.50    1272.98    1272.73    62    LVEVISGQLTSK    41A_4   
		    573    1022.24    2042.46    2042.08    61    SINKVPVDVSESPGFVVNR    41_4   
		    430    801.08    1600.15    1599.83    51    VPVDVSESPGFVVNR    41_4   
		    151    643.43    1284.85    1284.66    68    VTFDTVFELSK    41A_4   
		    183    547.86    1093.70    1093.64    46    YRPHPLLAK    41_4   
   
      Matching Genes:  
               gi|115250079|emb|CAJ67899.1|  (3-hydroxybutyryl-CoA dehydrogenase [Clostridium difficile 630]) 
           
  Protein Group 27   
      Expression Quality:  
         Score      Num Spectra      Num Peptides      High-Qual Peptides      % Coverage       542    15    9    8    21   
   
      Peptides:   
        Query    Observed    Mr(expt)    Mr(calc)    Score    Peptide    Result File   
		    98    583.43    1164.84    1164.59    32    EIHEQPTGVR    41A_7   
		    412    1086.86    2171.71    2171.01    59    ESDDVFYTWAGPEVAVASTK    41_7   
		    254    861.26    1720.51    1719.92    82    FVNIPVITDIASEFR    41A_7   
		    108    614.47    1226.92    1226.69    40    GTPVIAIATQEK    41A_7   
		    240    749.45    1496.89    1496.67    92    GYDSAGVAVNSSNEK    41_7   
		    182    715.51    1429.00    1428.83    73    ILSITNVVGSSIAR    41A_7   
		    204    754.04    1506.07    1505.76    45    IQEILDNEEYIK    41A_7   
		    232    733.49    1464.97    1464.74    78    TVVSSEHAFYLGR    41_7   
		    389    753.80    2258.37    2258.03    41    WATHGEPSDVNSHPHFNQAK    41A_7   
   
      Matching Genes:  
               gi|115249129|emb|CAJ66940.1|  (glucosamine--fructose-6-phosphate aminotransferase [isomerizing] [Clostridium difficile 630]) 
           
  Protein Group 28   
      Expression Quality:  
         Score      Num Spectra      Num Peptides      High-Qual Peptides      % Coverage       541    12    10    7    26   
   
      Peptides:   
        Query    Observed    Mr(expt)    Mr(calc)    Score    Peptide    Result File   
		    144    641.50    1280.99    1280.66    54    ESTIEFLTSVR    41A_6   
		    136    592.45    1182.89    1182.63    78    GISDFLLSFGK    41_6   
		    216    759.07    1516.13    1515.83    37    GNVLEGLKPESVFK    41A_6   
		    630    768.18    2301.51    2301.01    65    HEFTTSDPGMTYSVAETSVDK    41_4   
		    437    1159.79    2317.57    2317.01    51    HEFTTSDPGMTYSVAETSVDK +Oxidation (M)    41A_6   
		    99    515.32    1028.63    1028.45    34    ICSETYEK    41_6   
		    537    1248.45    2494.89    2494.29    53    LLAQALGANYDLIAQYPAWEFK    41_6   
		    195    728.01    1454.00    1453.76    52    NVEHDFLKDPIK    41A_6   
		    151    647.97    1293.93    1293.66    34    TCVVSLPVEYK    41A_6   
		    346    990.29    1978.56    1978.03    83    VLSVLNVDYELASVDGGTK    41A_6   
   
      Matching Genes:  
               gi|115249724|emb|CAJ67541.1|  (putative aminoacyl-histidine dipeptidase [Clostridium difficile 630]) 
           
  Protein Group 29   
      Expression Quality:  
         Score      Num Spectra      Num Peptides      High-Qual Peptides      % Coverage       534    11    9    7    17   
   
      Peptides:   
        Query    Observed    Mr(expt)    Mr(calc)    Score    Peptide    Result File   
		    188    639.89    1277.77    1277.60    58    EIEESIDSATGK    41_6   
		    447    1179.02    2356.03    2355.22    34    ESPAPIILATDTLSSDQNVAVSK    41_5   
		    61    545.40    1088.78    1089.59    43    IETAIELSSK    41A_5   
		    208    744.04    1486.06    1485.80    65    IITNQADAEAIVTK    41A_6   
		    161    704.99    1407.97    1407.72    54    SFLGTSDVDIIGGK    41A_5   
		    173    737.91    1473.80    1473.63    79    SGGSEDTGYVVEMK +Oxidation (M)    41A_5   
		    78    472.45    942.89    942.57    33    TAPLLLTSK    biorep41Cdiff_5   
		    300    952.23    1902.44    1901.88    93    TYNNTYSNVVTVAGEDR    41_5   
		    188    773.98    1545.95    1545.69    75    YYNSDDKNAITDK    41A_5   
   
      Matching Genes:  
               gi|115251846|emb|CAJ69681.1|  (cell surface protein (S-layer precursor protein) [Clostridium difficile 630]) 
           
  Protein Group 30   
      Expression Quality:  
         Score      Num Spectra      Num Peptides      High-Qual Peptides      % Coverage       521    24    8    7    43   
   
      Peptides:   
        Query    Observed    Mr(expt)    Mr(calc)    Score    Peptide    Result File   
		    545    970.21    1938.41    1937.95    97    AFGGADTWATSNTIAAGISK    41_4   
		    583    694.43    2080.26    2080.03    49    DGVPSILNPDDANALEEALK    41_4   
		    485    883.49    1764.98    1764.80    85    ECLAMGADDAILLSDR +Oxidation (M)    41_4   
		    278    871.12    1740.23    1739.87    105    QAIDGDTAQVGPQIAEK    41A_3   
		    211    604.39    1206.77    1206.61    57    QLEDGYELIK    41_4   
		    164    529.32    1056.62    1056.52    43    QVPDTNEVR    41_4   
		    122    613.44    1224.87    1224.61    57    VGDYDIIFAGR    41A_3   
		    194    564.41    1126.81    1126.70    28    VSTPVLLTAVK    41_4   
   
      Matching Genes:  
               gi|115249406|emb|CAJ67221.1|  (electron transfer flavoprotein beta-subunit [Clostridium difficile 630]) 
           
  Protein Group 31   
      Expression Quality:  
         Score      Num Spectra      Num Peptides      High-Qual Peptides      % Coverage       488    20    7    6    40   
   
      Peptides:   
        Query    Observed    Mr(expt)    Mr(calc)    Score    Peptide    Result File   
		    452    711.10    1420.19    1419.67    73    EIMDAANNTGASVK    biorep41Cdiff_2   
		    410    718.90    1435.78    1435.66    80    EIMDAANNTGASVK +Oxidation (M)    41_2   
		    351    670.02    1338.03    1337.72    83    IVYDAFAIVAEK    41_2   
		    214    558.39    1114.76    1115.60    68    LINNLMVDGK    41_2   
		    220    566.92    1131.82    1131.60    48    LINNLMVDGK +Oxidation (M)    41_2   
		    189    618.77    1235.54    1235.60    101    TGEEALEVFNK    41A_2   
		    344    595.57    1783.69    1782.94    35    VGGANYQVPIEVRPER    biorep41CdiffA_3   
   
      Matching Genes:  
               gi|115249073|emb|CAJ66884.1|  (30S ribosomal protein S7 [Clostridium difficile 630]) 
           
  Protein Group 32   
      Expression Quality:  
         Score      Num Spectra      Num Peptides      High-Qual Peptides      % Coverage       422    14    8    5    46   
   
      Peptides:   
        Query    Observed    Mr(expt)    Mr(calc)    Score    Peptide    Result File   
		    121    612.95    1223.88    1223.63    51    AGATYVSPFVGR    41A_3   
		    23    425.28    848.54    848.46    36    DFIEVVK    41A_3   
		    353    722.55    2164.62    2164.13    61    EISEIVDGPISAEVISLEHK    41A_3   
		    352    720.49    1438.96    1438.73    76    FFIDTANIEEIK    41_3   
		    485    1165.09    3492.25    3491.79    57    FFIDTANIEEIKEANDLGVICGVTTNPSLIAK    41A_3   
		    58    480.60    959.18    958.52    33    IPMTAEGLK    biorep41CdiffA_3   
		    174    558.39    1114.76    1114.61    69    MGADIATVPLK    41_3   
		    175    559.89    1117.77    1117.64    39    NPIHVLQAAR    41_3   
   
      Matching Genes:  
               gi|115251384|emb|CAJ69216.1|  (putative transaldolase [Clostridium difficile 630]) 
           
  Protein Group 33   
      Expression Quality:  
         Score      Num Spectra      Num Peptides      High-Qual Peptides      % Coverage       416    12    7    6    27   
   
      Peptides:   
        Query    Observed    Mr(expt)    Mr(calc)    Score    Peptide    Result File   
		    147    428.93    1283.77    1283.55    31    CEEFKTEEGR    41A_4   
		    431    781.25    2340.71    2340.16    63    GLLEEDLTEMNLSSVGDIIHR    41A_4   
		    507    913.20    1824.38    1823.96    96    HSIIVLAEGVGSASDLEK    41_4   
		    510    916.13    1830.24    1829.93    69    TIGLLTSGGDAPGMNAAIR +Oxidation (M)    41_4   
		    319    665.87    1329.73    1329.63    62    VFDKEAYEMAK    41_4   
		    182    673.91    1345.80    1345.62    48    VFDKEAYEMAK +Oxidation (M)    41A_4   
		    67    504.84    1007.68    1007.59    47    VTVLGHVQR    41A_4   
   
      Matching Genes:  
               gi|115252455|emb|CAJ70298.1|  (6-phosphofructokinase [Clostridium difficile 630]) 
           
  Protein Group 34   
      Expression Quality:  
         Score      Num Spectra      Num Peptides      High-Qual Peptides      % Coverage       410    12    8    5    32   
   
      Peptides:   
        Query    Observed    Mr(expt)    Mr(calc)    Score    Peptide    Result File   
		    193    794.17    1586.32    1585.88    33    AADPIVVLFGATSIGR    41A_5   
		    74    545.43    1088.84    1088.55    56    ATIDAGWLDK    41_5   
		    421    1139.30    2276.59    2276.15    92    ENLDILYELAEIIGGEVSGSR    41_5   
		    318    987.32    1972.62    1971.96    85    IHTGLTADCTGLAVAEDTK    41_5   
		    55    458.39    914.77    914.49    25    NPEAPIFK    41_5   
		    181    699.15    1396.29    1395.83    52    VLPELISQLSVAK    41_5   
		    29    444.38    886.75    886.55    42    VSALLLGSK    41A_5   
		    229    425.22    1272.63    1271.65    25    YADVGIVGDVHK    biorep41Cdiff_4   
   
      Matching Genes:  
               gi|115250077|emb|CAJ67897.1|  (electron transfer flavoprotein alpha-subunit [Clostridium difficile 630]) 
           
  Protein Group 35   
      Expression Quality:  
         Score      Num Spectra      Num Peptides      High-Qual Peptides      % Coverage       378    16    6    6    48   
   
      Peptides:   
        Query    Observed    Mr(expt)    Mr(calc)    Score    Peptide    Result File   
		    146    556.32    1110.62    1110.64    52    ADLDLRPALK    41A_2   
		    342    823.48    1644.95    1644.81    68    ALENYFNYETLIR    41A_2   
		    151    564.84    1127.66    1127.54    52    ANVQYYGTGR    41A_2   
		    242    656.46    1310.90    1310.73    107    LVAGEGNILVNGR    41A_2   
		    239    606.95    1211.88    1211.65    50    QPLVLTGNENK    41_2   
		    525    644.20    1929.57    1929.06    49    QPLVLTGNENKYDVIVK    41_2   
   
      Matching Genes:  
               gi|115249113|emb|CAJ66924.1|  (30S ribosomal protein S9 [Clostridium difficile 630]) 
           
  Protein Group 36   
      Expression Quality:  
         Score      Num Spectra      Num Peptides      High-Qual Peptides      % Coverage       378    8    7    6    16   
   
      Peptides:   
        Query    Observed    Mr(expt)    Mr(calc)    Score    Peptide    Result File   
		    302    805.59    1609.16    1608.80    75    INGVSVVSVESDNYK    41_6   
		    307    919.60    1837.18    1836.84    77    KDEFLSYINSTDYDK    41A_6   
		    112    549.91    1097.80    1097.71    55    LILAVTDIIK    41_6   
		    139    599.51    1197.00    1196.71    48    QLVPNLTTAIK    41_6   
		    334    972.28    1942.54    1941.98    46    TLPVTYDDGTFAGIITMK    41A_6   
		    378    980.18    1958.35    1957.97    45    TLPVTYDDGTFAGIITMK +Oxidation (M)    41_6   
		    463    1118.94    2235.87    2235.13    32    VVPLTPTSSILEAYNLMDEK +Oxidation (M)    41_6   
   
      Matching Genes:  
               gi|115249342|emb|CAJ67155.1|  (manganese-dependent inorganic pyrophosphatase [Clostridium difficile 630]) 
           
  Protein Group 37   
      Expression Quality:  
         Score      Num Spectra      Num Peptides      High-Qual Peptides      % Coverage       376    11    8    5    19   
   
      Peptides:   
        Query    Observed    Mr(expt)    Mr(calc)    Score    Peptide    Result File   
		    318    1040.66    2079.31    2078.92    65    AAEETGLPYAGFDGDQADPR    41A_5   
		    160    593.10    1184.18    1183.56    49    AFTNAQFETR    biorep41Cdiff_5   
		    210    652.13    1302.25    1301.66    49    IQGLVEVMEER    biorep41Cdiff_5   
		    165    674.09    1346.18    1345.68    67    LLIEELEDNMK    41_5   
		    148    681.99    1361.97    1361.67    35    LLIEELEDNMK +Oxidation (M)    41A_5   
		    404    1118.83    2235.65    2235.02    28    RAAEETGLPYAGFDGDQADPR    41_5   
		    237    603.44    1807.29    1806.95    51    VVINDLLAEQYANAFK    41A_5   
		    79    583.63    1165.24    1164.61    32    YRVDSLVEGK    biorep41CdiffA_5   
   
      Matching Genes:  
               gi|115249403|emb|CAJ67218.1|  (subunit of oxygen-sensitive 2-hydroxyisocaproyl-CoA dehydratase [Clostridium difficile 630]) 
           
  Protein Group 38   
      Expression Quality:  
         Score      Num Spectra      Num Peptides      High-Qual Peptides      % Coverage       364    14    8    4    49   
   
      Peptides:   
        Query    Observed    Mr(expt)    Mr(calc)    Score    Peptide    Result File   
		    197    498.48    994.94    994.46    38    GYDVIEDGK    biorep41Cdiff_2   
		    376    664.46    1326.90    1326.62    77    HETVDVPASNMK    biorep41Cdiff_2   
		    158    672.40    1342.78    1342.62    32    HETVDVPASNMK +Oxidation (M)    41A_1   
		    94    545.41    1088.81    1088.62    35    ILLEEGFIR    41A_1   
		    405    683.12    1364.22    1363.65    44    TMTDPIADMLTR    biorep41Cdiff_2   
		    355    698.94    1395.86    1395.64    36    TMTDPIADMLTR +2 Oxidation (M)    41_1   
		    351    694.55    1387.08    1386.81    41    VLNGLGISVISTSK    41_1   
		    226    571.38    1140.75    1140.59    61    VYAANHEIPK    41_1   
   
      Matching Genes:  
               gi|115249091|emb|CAJ66902.1|  (30S ribosomal protein S8 [Clostridium difficile 630]) 
           
  Protein Group 39   
      Expression Quality:  
         Score      Num Spectra      Num Peptides      High-Qual Peptides      % Coverage       358    9    7    4    14   
   
      Peptides:   
        Query    Observed    Mr(expt)    Mr(calc)    Score    Peptide    Result File   
		    246    532.03    1593.05    1592.78    46    AGTNMERPGPLAAHR +Oxidation (M)    41A_6   
		    536    1071.45    3211.33    3210.60    35    EEVVEETVEEAAPVSEAAVVPVSTGVAGETVK    41A_6   
		    150    647.91    1293.81    1293.65    46    IDKVEFADETK    41A_6   
		    423    809.06    1616.10    1615.81    93    IVLEENEQSLPMSK    41_3   
		    253    817.04    1632.07    1631.81    73    IVLEENEQSLPMSK +Oxidation (M)    41A_3   
		    69    479.85    957.68    957.51    38    NAIEGVEVK    41A_3   
		    229    418.28    1251.81    1251.54    27    YMTHMVDNNK    41_3   
   
      Matching Genes:  
               gi|115252300|emb|CAJ70141.1|  (proline reductase subunit proprotein [Clostridium difficile 630]) 
           
  Protein Group 40   
      Expression Quality:  
         Score      Num Spectra      Num Peptides      High-Qual Peptides      % Coverage       348    8    6    5    16   
   
      Peptides:   
        Query    Observed    Mr(expt)    Mr(calc)    Score    Peptide    Result File   
		    296    903.17    1804.32    1803.94    47    AGFVVSDSNIKPDNTLK    41A_6   
		    76    469.28    936.54    936.42    36    FDESPTNK    41_6   
		    222    672.46    1342.90    1342.72    74    GQATSIIEVAQAR    41_6   
		    379    981.24    1960.47    1959.95    57    LSFEEGVDSYVPYAGSLK    41_6   
		    39    443.80    885.58    885.47    91    VGAGNVVDR    41A_6   
		    342    918.72    1835.43    1834.85    43    VSEFMTPMSSIVYANK +2 Oxidation (M)    41_6   
   
      Matching Genes:  
               gi|115251390|emb|CAJ69222.1|  (inosine-5'-monophosphate dehydrogenase [Clostridium difficile 630]) 
           
  Protein Group 41   
      Expression Quality:  
         Score      Num Spectra      Num Peptides      High-Qual Peptides      % Coverage       342    15    6    5    59   
   
      Peptides:   
        Query    Observed    Mr(expt)    Mr(calc)    Score    Peptide    Result File   
		    493    1018.30    2034.59    2034.06    88    EQPQIAEVVEVGPGGIVEGK    41_1   
		    266    611.38    1220.75    1220.64    74    IEGQEYTILR    biorep41Cdiff_1   
		    38    420.79    839.56    839.50    26    IRPLADR    41_1   
		    41    447.01    892.00    891.44    41    MELTVGDK    biorep41CdiffA_1   
		    123    454.77    907.52    907.43    46    MELTVGDK +Oxidation (M)    41_1   
		    226    542.88    1083.75    1083.63    67    TASGIVLPGAAK    biorep41Cdiff_1   
   
      Matching Genes:  
               gi|115249203|emb|CAJ67015.1|  (10 kDa chaperonin [Clostridium difficile 630]) 
           
  Protein Group 42   
      Expression Quality:  
         Score      Num Spectra      Num Peptides      High-Qual Peptides      % Coverage       310    13    7    5    40   
   
      Peptides:   
        Query    Observed    Mr(expt)    Mr(calc)    Score    Peptide    Result File   
		    145    501.32    1000.62    1000.56    43    GTQAVGIVEK    41_3   
		    86    518.46    1034.90    1034.65    41    IILLGPPGAGK    41A_3   
		    526    692.17    2073.48    2073.07    31    IQVYLDETKPLVDYYSK    41_3   
		    2    409.28    816.55    816.43    42    NAGISLDK    41A_3   
		    367    495.29    1482.86    1482.78    29    NVAQGEHLDIFLK    41_3   
		    294    906.65    1811.29    1810.80    81    VEGVCDVCQGELYQR    41A_3   
		    229    511.67    1531.98    1531.78    43    YNIPHISTGDIFR    41A_3   
   
      Matching Genes:  
               gi|115249098|emb|CAJ66909.1|  (adenylate kinase [Clostridium difficile 630]) 
           
  Protein Group 43   
      Expression Quality:  
         Score      Num Spectra      Num Peptides      High-Qual Peptides      % Coverage       309    11    5    5    34   
   
      Peptides:   
        Query    Observed    Mr(expt)    Mr(calc)    Score    Peptide    Result File   
		    496    961.63    1921.24    1922.00    79    FPEYAAEVLSTVVEQIK    41_3   
		    234    630.00    1257.99    1257.72    68    IIIAEDVVTTGK    41_3   
		    485    935.08    1868.14    1867.83    50    LDIQVYESDECPLCK    41_3   
		    127    476.82    951.63    951.59    49    LPVVKPGSR    41_3   
		    404    780.51    1559.00    1558.78    63    TNHDIGMPIYSAIK    41_3   
   
      Matching Genes:  
               gi|115249197|emb|CAJ67009.1|  (orotate phosphoribosyltransferase [Clostridium difficile 630]) 
           
  Protein Group 44   
      Expression Quality:  
         Score      Num Spectra      Num Peptides      High-Qual Peptides      % Coverage       296    14    6    3    26   
   
      Peptides:   
        Query    Observed    Mr(expt)    Mr(calc)    Score    Peptide    Result File   
		    244    634.49    1266.96    1266.73    40    ALVPVVVEQTGR    41_3   
		    176    563.28    1124.56    1124.52    36    DNFMSALEAK    41_3   
		    182    571.32    1140.63    1140.51    36    DNFMSALEAK +Oxidation (M)    41_3   
		    276    657.42    1312.83    1312.66    70    EYGLIDEVFTK    41_3   
		    202    723.05    1444.08    1443.79    76    IKETLNEILSER    41A_3   
		    44    444.27    886.52    886.42    38    SYDIFSR    41A_3   
   
      Matching Genes:  
               gi|115252361|emb|CAJ70202.1|  (ATP-dependent Clp protease proteolytic subunit [Clostridium difficile 630]) 
           
  Protein Group 45   
      Expression Quality:  
         Score      Num Spectra      Num Peptides      High-Qual Peptides      % Coverage       291    10    6    4    14   
   
      Peptides:   
        Query    Observed    Mr(expt)    Mr(calc)    Score    Peptide    Result File   
		    105    531.38    1060.75    1060.52    69    EAFQNPDLK    41_6   
		    38    443.77    885.52    885.42    47    INFDYSK    41A_6   
		    85    485.35    968.69    968.48    38    IYATTDASK    41_6   
		    115    608.39    1214.77    1214.52    42    NYDKEEFDR    41A_6   
		    441    1098.88    2195.75    2195.04    67    QLATEEGYETFVIPDDVGGR    41_6   
		    172    625.97    1249.92    1249.63    28    SGTTTEPALAFR    41_6   
   
      Matching Genes:  
               gi|115252341|emb|CAJ70182.1|  (glucose-6-phosphate isomerase [Clostridium difficile 630]) 
           
  Protein Group 46   
      Expression Quality:  
         Score      Num Spectra      Num Peptides      High-Qual Peptides      % Coverage       290    9    5    5    30   
   
      Peptides:   
        Query    Observed    Mr(expt)    Mr(calc)    Score    Peptide    Result File   
		    94    458.27    914.53    914.45    41    AEINPDTR    41A_2   
		    98    465.76    929.50    929.52    57    ATANEILAK    41A_2   
		    334    659.45    1316.89    1316.62    81    DLSEDQVNELR    41_2   
		    26    420.75    839.48    839.49    53    IAGVDLPR    41A_2   
		    558    780.15    1558.28    1557.80    58    IKDLSEDQVNELR    biorep41Cdiff_2   
   
      Matching Genes:  
               gi|115249103|emb|CAJ66914.1|  (30S ribosomal protein S13 [Clostridium difficile 630]) 
           
  Protein Group 47   
      Expression Quality:  
         Score      Num Spectra      Num Peptides      High-Qual Peptides      % Coverage       290    16    5    5    23   
   
      Peptides:   
        Query    Observed    Mr(expt)    Mr(calc)    Score    Peptide    Result File   
		    117    607.95    1213.89    1213.67    83    AGENLLSLLER    41A_3   
		    85    517.92    1033.83    1033.57    48    RLDNVVYR    41A_3   
		    136    492.32    982.63    982.61    43    VDIPSLIVK    41_3   
		    160    525.38    1048.74    1048.57    60    VSNYGLQLR    41_3   
		    401    675.10    1348.18    1347.65    56    WLEANVEGMTAK    biorep41Cdiff_3   
   
      Matching Genes:  
               gi|115249105|emb|CAJ66916.1|  (30S ribosomal protein S4 [Clostridium difficile 630]) 
           
  Protein Group 48   
      Expression Quality:  
         Score      Num Spectra      Num Peptides      High-Qual Peptides      % Coverage       278    13    5    4    28   
   
      Peptides:   
        Query    Observed    Mr(expt)    Mr(calc)    Score    Peptide    Result File   
		    403    776.03    1550.04    1549.72    52    EVSWLPSYGPEMR    41_3   
		    407    784.00    1565.99    1565.72    27    EVSWLPSYGPEMR +Oxidation (M)    41_3   
		    167    538.36    1074.70    1074.57    72    FKDDVIPGGK    41_3   
		    138    493.87    985.72    985.62    56    IIVNSSLIK    41_3   
		    576    770.81    2309.41    2309.11    71    TDVDVYYIPANELAAELGNDK    41_3   
   
      Matching Genes:  
               gi|115249127|emb|CAJ66938.1|  (putative subunit of oxidoreductase [Clostridium difficile 630]) 
           
  Protein Group 49   
      Expression Quality:  
         Score      Num Spectra      Num Peptides      High-Qual Peptides      % Coverage       277    5    5    4    23   
   
      Peptides:   
        Query    Observed    Mr(expt)    Mr(calc)    Score    Peptide    Result File   
		    102    578.84    1155.66    1155.52    66    FFEGDIEGSR    41A_4   
		    100    573.37    1144.73    1144.59    46    HFETIANSVK    41A_4   
		    250    627.98    1253.95    1253.75    69    LPIILYNVPGR    41_4   
		    408    761.01    1520.01    1519.78    64    TAMNLLGFNVGDLR    41_4   
		    651    829.78    2486.31    2485.18    32    VPVIAGSGSNDTMHSVNLSQEAEK +Oxidation (M)    41_4   
   
      Matching Genes:  
               gi|115252282|emb|CAJ70123.1|  (dihydrodipicolinate synthase [Clostridium difficile 630]) 
           
  Protein Group 50   
      Expression Quality:  
         Score      Num Spectra      Num Peptides      High-Qual Peptides      % Coverage       275    7    5    4    25   
   
      Peptides:   
        Query    Observed    Mr(expt)    Mr(calc)    Score    Peptide    Result File   
		    139    495.27    988.52    988.49    41    DIKPEEMK    41_3   
		    327    696.99    1391.96    1391.77    63    GYLVSNKEELLK    41_3   
		    343    710.96    1419.91    1419.65    79    MDQNLNWLNEK +Oxidation (M)    41_3   
		    63    473.30    944.58    944.47    32    TIAAPANCK    41A_3   
		    448    712.66    1423.30    1422.62    60    TTGICIDCSSPGR    biorep41Cdiff_3   
   
      Matching Genes:  
               gi|115251115|emb|CAJ68946.1|  (conserved hypothetical protein [Clostridium difficile 630]) 
           
  Protein Group 51   
      Expression Quality:  
         Score      Num Spectra      Num Peptides      High-Qual Peptides      % Coverage       270    9    6    4    36   
   
      Peptides:   
        Query    Observed    Mr(expt)    Mr(calc)    Score    Peptide    Result File   
		    119    451.28    900.56    900.50    30    AGREDLIK    41_2   
		    222    573.85    1145.68    1145.58    58    ETEGEIEVLK    41_2   
		    544    1038.72    2075.43    2075.03    62    EYLPQQLSEEELEEIVK    41_2   
		    135    458.33    914.64    914.59    29    KSVVTLIR    41_2   
		    175    605.87    1209.72    1209.59    49    STISEVGATSMK    41A_2   
		    181    613.91    1225.80    1225.59    42    STISEVGATSMK +Oxidation (M)    41A_2   
   
      Matching Genes:  
               gi|115251499|emb|CAJ69332.1|  (putative tRNA binding protein [Clostridium difficile 630]) 
           
  Protein Group 52   
      Expression Quality:  
         Score      Num Spectra      Num Peptides      High-Qual Peptides      % Coverage       268    5    4    3    23   
   
      Peptides:   
        Query    Observed    Mr(expt)    Mr(calc)    Score    Peptide    Result File   
		    283    595.04    1188.07    1187.58    38    FDETVEAHIK    biorep41Cdiff_3   
		    363    936.36    1870.70    1869.94    111    FYDASEALTLVSDIAGAK    biorep41CdiffA_3   
		    284    790.53    1579.05    1578.83    65    LTENFTALMDAIIK    biorep41CdiffA_3   
		    136    617.80    1233.59    1232.64    54    SITVASSMGPGVK    biorep41CdiffA_3   
   
      Matching Genes:  
               gi|115249066|emb|CAJ66877.1|  (50S ribosomal protein L1 [Clostridium difficile 630]) 
           
  Protein Group 53   
      Expression Quality:  
         Score      Num Spectra      Num Peptides      High-Qual Peptides      % Coverage       262    6    4    3    68   
   
      Peptides:   
        Query    Observed    Mr(expt)    Mr(calc)    Score    Peptide    Result File   
		    1    402.31    802.60    803.40    44    ACVEVAR    41_1   
		    666    1185.97    3554.88    3553.83    106    AGNLLFVSGQVPLVPETMEVVEGDVQAQTAQSLK    41_2   
		    397    894.33    2679.97    2679.19    29    DMNEFGAINEVYAEYFGENKPAR +Oxidation (M)    41A_1   
		    370    831.29    2490.86    2490.27    83    MKHEVIHTNDAPAALGPYSQAIK    41A_1   
   
      Matching Genes:  
               gi|115251566|emb|CAJ69399.1|  (putative translation inhibitor endoribonuclease [Clostridium difficile 630]) 
           
  Protein Group 54   
      Expression Quality:  
         Score      Num Spectra      Num Peptides      High-Qual Peptides      % Coverage       261    6    5    5    19   
   
      Peptides:   
        Query    Observed    Mr(expt)    Mr(calc)    Score    Peptide    Result File   
		    344    1035.36    2068.70    2067.97    64    GGPGLGSIQPSQADYFMSTR    41_5   
		    307    1023.83    2045.65    2045.05    45    VLWPFPFEAFNQIPNAR    41A_5   
		    155    632.41    1262.81    1262.65    41    VMTSSSSPGVALK    41_7   
		    114    640.45    1278.88    1278.65    64    VMTSSSSPGVALK +Oxidation (M)    41A_5   
		    432    1154.38    2306.74    2306.13    47    YFFGYPITPQSELPEYLSR    41_5   
   
      Matching Genes:  
               gi|115249125|emb|CAJ66936.1|  (putative oxidoreductase, thiamine diP-binding subunit [Clostridium difficile 630]) 
           
  Protein Group 55   
      Expression Quality:  
         Score      Num Spectra      Num Peptides      High-Qual Peptides      % Coverage       258    14    5    4    17   
   
      Peptides:   
        Query    Observed    Mr(expt)    Mr(calc)    Score    Peptide    Result File   
		    57    533.10    1064.18    1063.52    47    AGMMGIPYPK    biorep41CdiffA_5   
		    143    661.51    1321.00    1320.66    38    AVQLHGGYGYTR    biorep41CdiffA_5   
		    75    578.92    1155.82    1155.64    68    HLVYQAAINK    41A_5   
		    357    647.42    1939.24    1938.08    55    IGIAAQALGLAQGALDETVK    biorep41CdiffA_5   
		    361    763.16    1524.31    1523.75    50    ITEIYEGTSEVQR    biorep41Cdiff_5   
   
      Matching Genes:  
               gi|115250075|emb|CAJ67895.1|  (butyryl-CoA dehydrogenase [Clostridium difficile 630]) 
           
  Protein Group 56   
      Expression Quality:  
         Score      Num Spectra      Num Peptides      High-Qual Peptides      % Coverage       256    9    6    2    36   
   
      Peptides:   
        Query    Observed    Mr(expt)    Mr(calc)    Score    Peptide    Result File   
		    322    978.23    1954.44    1953.98    85    AIANSDLGLNPSNDGEVIR    41A_3   
		    58    459.84    917.67    917.51    30    EIDTLLSK    41A_3   
		    62    435.27    868.53    868.44    36    FEFGTIR    41_3   
		    168    545.41    1088.80    1089.52    42    GGELTEDELK    41_3   
		    120    609.85    1217.69    1217.61    38    GGELTEDELKK    41A_3   
		    714    1194.43    2386.85    2386.22    25    VDYYGTPTPINQIGAISVPEPR    biorep41Cdiff_3   
   
      Matching Genes:  
               gi|115251191|emb|CAJ69022.1|  (ribosome recycling factor [Clostridium difficile 630]) 
           
  Protein Group 57   
      Expression Quality:  
         Score      Num Spectra      Num Peptides      High-Qual Peptides      % Coverage       245    13    5    3    39   
   
      Peptides:   
        Query    Observed    Mr(expt)    Mr(calc)    Score    Peptide    Result File   
		    194    734.00    1465.99    1465.78    66    AGSQVSGPVPLPTEK    41A_1   
		    249    532.58    1594.72    1593.87    34    KAGSQVSGPVPLPTEK    biorep41CdiffA_1   
		    91    541.41    1080.80    1080.62    67    LIDIANPTPK    41A_1   
		    14    425.79    849.56    849.46    44    LLDFSAGK    biorep41CdiffA_1   
		    8    415.04    828.07    827.52    34    QVVTILR    biorep41CdiffA_1   
   
      Matching Genes:  
               gi|115249076|emb|CAJ66887.1|  (30S ribosomal protein S10 [Clostridium difficile 630]) 
           
  Protein Group 58   
      Expression Quality:  
         Score      Num Spectra      Num Peptides      High-Qual Peptides      % Coverage       244    12    5    4    29   
   
      Peptides:   
        Query    Observed    Mr(expt)    Mr(calc)    Score    Peptide    Result File   
		    112    580.46    1158.90    1158.68    49    ALVFENVLVR    41A_3   
		    107    565.88    1129.74    1129.56    51    ESGDIAGTPGVK    41A_3   
		    463    891.58    1781.14    1780.85    64    FKPLSQPGQYACEEK    41_3   
		    17    428.47    854.93    854.52    38    LPIALSNK    biorep41CdiffA_3   
		    180    565.38    1128.75    1128.58    42    LVGPAGEVEMK    41_3   
   
      Matching Genes:  
               gi|115251734|emb|CAJ69569.1|  (putative propanediol utilization protein [Clostridium difficile 630]) 
           
  Protein Group 59   
      Expression Quality:  
         Score      Num Spectra      Num Peptides      High-Qual Peptides      % Coverage       240    8    4    4    30   
   
      Peptides:   
        Query    Observed    Mr(expt)    Mr(calc)    Score    Peptide    Result File   
		    182    510.33    1018.65    1018.57    40    EIISSITTR    41_2   
		    362    680.42    1358.82    1358.69    65    IECQGEGLVNLK    41_2   
		    174    605.83    1209.65    1209.63    57    LSEVVEFYPK    41A_2   
		    312    734.43    1466.84    1466.87    78    LVNDVELVNVLIK    41A_2   
   
      Matching Genes:  
               gi|115249997|emb|CAJ67817.1|  (hypothetical phage protein [Clostridium difficile 630]) 
              Other Genes Matching Peptide Subset:  
               gi|115251944|emb|CAJ69780.1|  (hypothetical phage protein [Clostridium difficile 630]) 
           
  Protein Group 60   
      Expression Quality:  
         Score      Num Spectra      Num Peptides      High-Qual Peptides      % Coverage       237    9    4    3    46   
   
      Peptides:   
        Query    Observed    Mr(expt)    Mr(calc)    Score    Peptide    Result File   
		    186    515.31    1028.61    1028.55    49    IANGEIPSTK    41_1   
		    105    567.39    1132.76    1132.55    64    KLPNYEAGQN    41A_1   
		    195    738.99    1475.97    1475.67    85    VINNCGSDGGQEVK    41A_1   
		    611    740.82    2219.45    2218.25    39    VLAFNDLNPVAPYHILVVPK    biorep41Cdiff_1   
   
      Matching Genes:  
               gi|115251501|emb|CAJ69334.1|  (histidine triad nucleotide-binding protein [Clostridium difficile 630]) 
           
  Protein Group 61   
      Expression Quality:  
         Score      Num Spectra      Num Peptides      High-Qual Peptides      % Coverage       232    5    4    4    11   
   
      Peptides:   
        Query    Observed    Mr(expt)    Mr(calc)    Score    Peptide    Result File   
		    236    777.49    1552.96    1552.73    40    EAYPGDVFYLHSR    41A_6   
		    360    937.09    1872.16    1871.84    75    ELAAFSQFGSDLDEDTK    41_6   
		    169    676.56    1351.10    1350.79    75    IVEVPVGEALIGR    41A_6   
		    228    770.56    1539.10    1538.81    42    TRPVESEAPGIIDR    41A_6   
   
      Matching Genes:  
               gi|115252530|emb|CAJ70373.1|  (ATP synthase alpha chain [Clostridium difficile 630]) 
           
  Protein Group 62   
      Expression Quality:  
         Score      Num Spectra      Num Peptides      High-Qual Peptides      % Coverage       222    5    4    3    10   
   
      Peptides:   
        Query    Observed    Mr(expt)    Mr(calc)    Score    Peptide    Result File   
		    129    581.90    1161.79    1161.59    50    FPLDTEAELK    41_6   
		    332    969.68    1937.34    1936.99    64    LGNIIVGYSYEGEPVTAR    41A_6   
		    317    857.21    1712.40    1711.87    83    SDIEIAQEAKPQDIR    41_6   
		    197    648.43    1294.85    1294.68    25    TTTTIGVADAFAK    41_6   
   
      Matching Genes:  
               gi|115249735|emb|CAJ67552.1|  (formate--tetrahydrofolate ligase [Clostridium difficile 630]) 
           
  Protein Group 63   
      Expression Quality:  
         Score      Num Spectra      Num Peptides      High-Qual Peptides      % Coverage       218    8    5    3    13   
   
      Peptides:   
        Query    Observed    Mr(expt)    Mr(calc)    Score    Peptide    Result File   
		    219    669.44    1336.87    1336.61    56    DTDPQSALEYAK    41_6   
		    318    572.41    1714.20    1713.86    27    LEKPEEMTGHSLISK +Oxidation (M)    41_6   
		    408    1037.81    2073.61    2073.00    58    VATYDLKPEMSAYELTDK    41_6   
		    105    583.37    1164.73    1165.51    29    VLCEAMDNAK +Oxidation (M)    41A_6   
		    104    582.87    1163.73    1163.57    48    VYVHAFTDGR    41A_6   
   
      Matching Genes:  
               gi|115252228|emb|CAJ70068.1|  (2,3-bisphosphoglycerate-independent phosphoglycerate mutase [Clostridium difficile 630]) 
           
  Protein Group 64   
      Expression Quality:  
         Score      Num Spectra      Num Peptides      High-Qual Peptides      % Coverage       213    5    3    2    18   
   
      Peptides:   
        Query    Observed    Mr(expt)    Mr(calc)    Score    Peptide    Result File   
		    444    814.89    2441.65    2441.04    26    FACVDGPEFDGHLVDFDQAMR +Oxidation (M)    41A_4   
		    497    901.65    1801.28    1801.01    90    ILFVAGGVGSAPVYPQVK    41_4   
		    306    883.08    1764.14    1763.78    97    NVYVSTDDGTYGFNGR    41A_4   
   
      Matching Genes:  
               gi|115250577|emb|CAJ68401.1|  (putative dehydrogenase, electron transfer subunit [Clostridium difficile 630]) 
           
  Protein Group 65   
      Expression Quality:  
         Score      Num Spectra      Num Peptides      High-Qual Peptides      % Coverage       212    5    4    3    9   
   
      Peptides:   
        Query    Observed    Mr(expt)    Mr(calc)    Score    Peptide    Result File   
		    95    560.86    1119.70    1119.59    35    GNLVNYLAEK    41A_6   
		    156    615.95    1229.88    1229.60    40    HVFEGESNIAK    41_6   
		    100    579.33    1156.66    1156.51    44    NEMIESAYGK +Oxidation (M)    41A_6   
		    268    846.51    1691.00    1690.74    93    SNSNLQTYTYSEER    41A_6   
   
      Matching Genes:  
               gi|115251723|emb|CAJ69558.1|  (oligopeptide ABC transporter, substrate-binding protein [Clostridium difficile 630]) 
           
  Protein Group 66   
      Expression Quality:  
         Score      Num Spectra      Num Peptides      High-Qual Peptides      % Coverage       209    7    4    4    22   
   
      Peptides:   
        Query    Observed    Mr(expt)    Mr(calc)    Score    Peptide    Result File   
		    452    576.69    1727.06    1726.88    45    EGITSVGENKPQELAR    41_3   
		    309    934.27    1866.52    1865.85    58    GLMTMAPFIEDEDEIR    41A_3   
		    314    681.44    1360.87    1360.67    55    TVDVDLVNEAMR    41_3   
		    73    483.36    964.70    964.50    51    VGTSIFGER    41A_3   
   
      Matching Genes:  
               gi|115251674|emb|CAJ69509.1|  (putative alanine racemase [Clostridium difficile 630]) 
           
  Protein Group 67   
      Expression Quality:  
         Score      Num Spectra      Num Peptides      High-Qual Peptides      % Coverage       207    7    4    4    48   
   
      Peptides:   
        Query    Observed    Mr(expt)    Mr(calc)    Score    Peptide    Result File   
		    145    652.93    1303.84    1303.63    45    EGASKEEADQIK    41A_1   
		    515    721.17    2160.50    2160.05    74    FGVSASAPVMVAGAAAGGPAAEEK +Oxidation (M)    41_1   
		    225    749.27    1496.53    1495.74    42    TEFDVVLTDVGSSK    biorep41CdiffA_1   
		    218    529.10    1056.19    1055.62    46    VLELNELVK    biorep41Cdiff_1   
   
      Matching Genes:  
               gi|115249068|emb|CAJ66879.1|  (50S ribosomal protein L7/L12 [Clostridium difficile 630]) 
           
  Protein Group 68   
      Expression Quality:  
         Score      Num Spectra      Num Peptides      High-Qual Peptides      % Coverage       206    4    4    2    6   
   
      Peptides:   
        Query    Observed    Mr(expt)    Mr(calc)    Score    Peptide    Result File   
		    27    430.84    859.66    859.48    62    FAGDILPK    41A_7   
		    89    561.42    1120.83    1120.61    29    FVGSTLEQLK    41A_7   
		    264    794.59    1587.16    1586.84    90    LSADILDSLGLVDEK    41_7   
		    88    498.29    994.57    994.50    25    NLESYIEK    41_7   
   
      Matching Genes:  
               gi|115251485|emb|CAJ69318.1|  (glycyl-tRNA synthetase beta chain [Clostridium difficile 630]) 
           
  Protein Group 69   
      Expression Quality:  
         Score      Num Spectra      Num Peptides      High-Qual Peptides      % Coverage       205    5    5    4    51   
   
      Peptides:   
        Query    Observed    Mr(expt)    Mr(calc)    Score    Peptide    Result File   
		    414    481.94    1442.81    1442.66    26    AHNDANMLSLGER +Oxidation (M)    41_2   
		    272    592.06    1182.10    1181.57    44    CAVVSDVFSAK    biorep41Cdiff_2   
		    203    541.39    1080.77    1080.57    46    EIISYLESK    41_2   
		    665    1159.93    3476.76    3475.61    45    GIECVDYGTNNATDSVDYPVYGEIVANSVINK    41_2   
		    440    468.80    1403.38    1402.67    44    IGLGCDHGGYNLK    biorep41Cdiff_2   
   
      Matching Genes:  
               gi|115252540|emb|CAJ70383.1|  (ribose-5-phosphate isomerase 2 [Clostridium difficile 630]) 
           
  Protein Group 70   
      Expression Quality:  
         Score      Num Spectra      Num Peptides      High-Qual Peptides      % Coverage       192    6    5    2    6   
   
      Peptides:   
        Query    Observed    Mr(expt)    Mr(calc)    Score    Peptide    Result File   
		    80    548.32    1094.63    1094.50    41    EGYIYEGHK    41A_8   
		    380    1179.86    2357.71    2357.08    28    ENFDQLFPADYISEGIDQTR    41A_8   
		    64    487.00    971.98    971.46    33    ESVFSFEK    biorep41Cdiff_7   
		    265    704.47    1406.93    1406.70    57    LTEELINEGYAR    41_8   
		    348    873.10    1744.19    1743.81    33    SLEEQNLNDHMTGIK +Oxidation (M)    41_8   
   
      Matching Genes:  
               gi|115251669|emb|CAJ69504.1|  (isoleucyl-tRNA synthetase [Clostridium difficile 630]) 
           
  Protein Group 71   
      Expression Quality:  
         Score      Num Spectra      Num Peptides      High-Qual Peptides      % Coverage       191    6    4    1    7   
   
      Peptides:   
        Query    Observed    Mr(expt)    Mr(calc)    Score    Peptide    Result File   
		    263    887.22    1772.43    1771.92    38    EDLPVLLPTDVEFTGK    41A_7   
		    420    737.54    2209.61    2209.04    26    FGFNTAISALMELINDMYK +2 Oxidation (M)    41_7   
		    29    431.82    861.62    861.44    34    VTADLSEK    41A_7   
		    254    771.97    1541.93    1541.66    93    YVDSNNENEPFSK    41_7   
   
      Matching Genes:  
               gi|115251575|emb|CAJ69408.1|  (leucyl-tRNA synthetase [Clostridium difficile 630]) 
           
  Protein Group 72   
      Expression Quality:  
         Score      Num Spectra      Num Peptides      High-Qual Peptides      % Coverage       187    7    4    1    11   
   
      Peptides:   
        Query    Observed    Mr(expt)    Mr(calc)    Score    Peptide    Result File   
		    338    899.63    1797.25    1796.95    29    AMDLLVPGVGEIVGGSQR    41_6   
		    142    637.43    1272.85    1272.63    33    EEREDVLLDR    41A_6   
		    141    601.39    1200.76    1200.59    30    NVYTFGPTFR    41_6   
		    323    869.66    1737.30    1736.85    95    SIVVEGESDSSYPLQK    41_6   
   
      Matching Genes:  
               gi|115251299|emb|CAJ69130.1|  (asparaginyl-tRNA synthetase [Clostridium difficile 630]) 
           
  Protein Group 73   
      Expression Quality:  
         Score      Num Spectra      Num Peptides      High-Qual Peptides      % Coverage       184    7    4    3    30   
   
      Peptides:   
        Query    Observed    Mr(expt)    Mr(calc)    Score    Peptide    Result File   
		    165    486.86    971.70    971.53    48    ILGEGNLEK    41_2   
		    337    440.67    1319.00    1318.74    41    LHELKPAEGAVR    41_2   
		    281    631.40    1260.79    1260.63    70    VGFEGGQMPLAR    41_2   
		    408    717.53    1433.04    1433.75    25    VYTEVNVEVLNR    41_2   
   
      Matching Genes:  
               gi|115249096|emb|CAJ66907.1|  (50S ribosomal protein L15 [Clostridium difficile 630]) 
           
  Protein Group 74   
      Expression Quality:  
         Score      Num Spectra      Num Peptides      High-Qual Peptides      % Coverage       181    5    4    2    10   
   
      Peptides:   
        Query    Observed    Mr(expt)    Mr(calc)    Score    Peptide    Result File   
		    64    500.78    999.55    1000.56    33    GKPSVTLDGK    41A_6   
		    52    466.32    930.62    930.50    50    LEDAVAVSK    41A_6   
		    508    995.69    2984.06    2983.51    34    VVDLSALSEEVVLIAHDLTPSDTATMNK +Oxidation (M)    41A_7   
		    248    706.86    1411.70    1411.55    64    WAGMCGESAGDQK +Oxidation (M)    41_6   
   
      Matching Genes:  
               gi|115251808|emb|CAJ69643.1|  (phosphoenolpyruvate-protein phosphotransferase [Clostridium difficile 630]) 
           
  Protein Group 75   
      Expression Quality:  
         Score      Num Spectra      Num Peptides      High-Qual Peptides      % Coverage       180    4    3    3    11   
   
      Peptides:   
        Query    Observed    Mr(expt)    Mr(calc)    Score    Peptide    Result File   
		    233    831.56    1661.10    1660.76    51    AHCSTVGAGEFLQER    41_5   
		    205    839.12    1676.23    1675.81    67    EVVFAADDNVVGENAK    41A_5   
		    204    745.53    1489.05    1488.72    62    MENGDVVLLENTR    41_5   
   
      Matching Genes:  
               gi|115252230|emb|CAJ70070.1|  (phosphoglycerate kinase [Clostridium difficile 630]) 
           
  Protein Group 76   
      Expression Quality:  
         Score      Num Spectra      Num Peptides      High-Qual Peptides      % Coverage       173    5    4    3    34   
   
      Peptides:   
        Query    Observed    Mr(expt)    Mr(calc)    Score    Peptide    Result File   
		    690    703.24    2106.70    2105.99    43    MPDLNAASVEAAMSMIAGTAR    biorep41Cdiff_2   
		    685    697.68    2090.03    2089.09    49    TADQAGMIIPVVITVYQDR    biorep41Cdiff_2   
		    689    1053.94    2105.86    2105.08    36    TADQAGMIIPVVITVYQDR +Oxidation (M)    biorep41Cdiff_2   
		    131    455.52    909.03    908.57    45    TPPAAVLIK    biorep41Cdiff_2   
   
      Matching Genes:  
               gi|115249065|emb|CAJ66876.1|  (50S ribosomal protein L11 [Clostridium difficile 630]) 
           
  Protein Group 77   
      Expression Quality:  
         Score      Num Spectra      Num Peptides      High-Qual Peptides      % Coverage       171    3    3    2    41   
   
      Peptides:   
        Query    Observed    Mr(expt)    Mr(calc)    Score    Peptide    Result File   
		    329    675.36    1348.71    1348.59    79    GCGLCVEACPVK    41_1   
		    276    628.97    1255.93    1255.71    66    IIQLDSNVINK    41_1   
		    43    440.34    878.66    878.42    26    VSFNQER    41A_1   
   
      Matching Genes:  
               gi|115249124|emb|CAJ66935.1|  (ferredoxin [Clostridium difficile 630]) 
           
  Protein Group 78   
      Expression Quality:  
         Score      Num Spectra      Num Peptides      High-Qual Peptides      % Coverage       168    3    3    2    13   
   
      Peptides:   
        Query    Observed    Mr(expt)    Mr(calc)    Score    Peptide    Result File   
		    250    627.98    1253.95    1253.75    69    LPIILYNVPGR    41_4   
		    408    761.01    1520.01    1519.78    64    TAMNILGFNVGDLR    41_4   
		    304    456.81    1367.40    1366.82    35    VNIKPSVVAELAK    biorep41Cdiff_4   
   
      Matching Genes:  
               gi|115252280|emb|CAJ70121.1|  (dihydrodipicolinate synthase [Clostridium difficile 630]) 
           
  Protein Group 79   
      Expression Quality:  
         Score      Num Spectra      Num Peptides      High-Qual Peptides      % Coverage       168    4    4    3    15   
   
      Peptides:   
        Query    Observed    Mr(expt)    Mr(calc)    Score    Peptide    Result File   
		    527    936.87    1871.73    1872.01    46    EGLLNEDVILESITSIK    41_4   
		    607    730.88    2189.63    2189.16    41    VNVEDLIYPLFVVEGENIK    41_4   
		    671    897.42    2689.23    2688.43    39    VNVEDLIYPLFVVEGENIKEEIK    41_4   
		    239    619.37    1236.73    1236.56    42    YASNYYGPFR    41_4   
   
      Matching Genes:  
               gi|115252479|emb|CAJ70322.1|  (delta-aminolevulinic acid dehydratase [Clostridium difficile 630]) 
           
  Protein Group 80   
      Expression Quality:  
         Score      Num Spectra      Num Peptides      High-Qual Peptides      % Coverage       166    6    4    3    22   
   
      Peptides:   
        Query    Observed    Mr(expt)    Mr(calc)    Score    Peptide    Result File   
		    256    870.78    1739.54    1738.88    42    AAALDTFETEGLFLNK    41A_7   
		    128    533.59    1065.16    1064.53    25    DGAYIVNTGR    biorep41Cdiff_4   
		    456    1321.40    2640.79    2640.31    53    GLGANVIAFDQYPNSDLNDILTYK    41A_5   
		    522    1355.11    2708.20    2707.30    46    GYDGISIQQTNYIDNPYIYETLK    41_5   
   
      Matching Genes:  
               gi|115249400|emb|CAJ67215.1|  ((R)-2-hydroxyisocaproate dehydrogenase [Clostridium difficile 630]) 
           
  Protein Group 81   
      Expression Quality:  
         Score      Num Spectra      Num Peptides      High-Qual Peptides      % Coverage       164    6    3    3    9   
   
      Peptides:   
        Query    Observed    Mr(expt)    Mr(calc)    Score    Peptide    Result File   
		    119    618.48    1234.94    1234.64    62    GKETVISTADSK    41_5   
		    40    465.36    928.70    928.56    55    IGIEGSILK    41A_5   
		    231    827.12    1652.22    1651.75    47    YGFHGTSHNYVSQR    41_5   
   
      Matching Genes:  
               gi|115250207|emb|CAJ68028.1|  (acetate kinase [Clostridium difficile 630]) 
           
  Protein Group 82   
      Expression Quality:  
         Score      Num Spectra      Num Peptides      High-Qual Peptides      % Coverage       163    5    4    2    15   
   
      Peptides:   
        Query    Observed    Mr(expt)    Mr(calc)    Score    Peptide    Result File   
		    125    629.48    1256.94    1257.66    32    AGINTVEELANK    41A_4   
		    91    473.53    945.05    944.52    29    SLEEVIQK    biorep41Cdiff_4   
		    382    1085.97    2169.92    2169.16    56    TENVPIGVLPVDSIYTPVEK    41_5   
		    64    552.84    1103.66    1103.54    46    VSYHVENTR    41A_5   
   
      Matching Genes:  
               gi|115249106|emb|CAJ66917.1|  (DNA-directed RNA polymerase alpha chain [Clostridium difficile 630]) 
           
  Protein Group 83   
      Expression Quality:  
         Score      Num Spectra      Num Peptides      High-Qual Peptides      % Coverage       162    8    4    2    16   
   
      Peptides:   
        Query    Observed    Mr(expt)    Mr(calc)    Score    Peptide    Result File   
		    245    794.52    1587.03    1586.74    29    DNMIPYYPLGNFK +Oxidation (M)    41A_3   
		    101    574.87    1147.72    1147.56    54    NAETQGYPIR    41A_4   
		    122    473.30    944.59    944.48    37    NVEVEEVK    41_3   
		    163    532.84    1063.66    1063.58    42    VAVDTPAHVR    41_3   
   
      Matching Genes:  
               gi|115249126|emb|CAJ66937.1|  (putative subunit of oxidoreductase [Clostridium difficile 630]) 
           
  Protein Group 84   
      Expression Quality:  
         Score      Num Spectra      Num Peptides      High-Qual Peptides      % Coverage       162    5    3    3    15   
   
      Peptides:   
        Query    Observed    Mr(expt)    Mr(calc)    Score    Peptide    Result File   
		    158    654.89    1307.78    1307.60    59    AATPDVNSENYK    41A_4   
		    141    638.43    1274.85    1274.65    50    HYGLTTEDIVK    41A_4   
		    662    848.15    2541.44    2540.28    53    VVTAEEHSIIGGLGSAVCEALAETK    41_4   
   
      Matching Genes:  
               gi|115251376|emb|CAJ69208.1|  (transketolase [Clostridium difficile 630]) 
           
  Protein Group 85   
      Expression Quality:  
         Score      Num Spectra      Num Peptides      High-Qual Peptides      % Coverage       161    4    3    3    11   
   
      Peptides:   
        Query    Observed    Mr(expt)    Mr(calc)    Score    Peptide    Result File   
		    240    604.82    1811.44    1810.94    74    ALDNILEEGIENVLNR    41A_5   
		    153    438.74    1313.18    1312.62    40    IGHMGENANLNK +Oxidation (M)    41_5   
		    233    897.23    1792.44    1791.93    47    IPESIGALELTDYMLK    41A_5   
   
      Matching Genes:  
               gi|115251586|emb|CAJ69419.1|  (putative aminotransferase [Clostridium difficile 630]) 
           
  Protein Group 86   
      Expression Quality:  
         Score      Num Spectra      Num Peptides      High-Qual Peptides      % Coverage       160    3    3    2    23   
   
      Peptides:   
        Query    Observed    Mr(expt)    Mr(calc)    Score    Peptide    Result File   
		    518    1021.16    2040.31    2039.94    37    EGYNQIAAIFEETANNEK    41_3   
		    99    555.87    1109.74    1109.61    48    IAYLFEAVGK    41A_3   
		    180    676.90    1351.79    1351.62    75    NLMAGFAGESEAR    41A_3   
   
      Matching Genes:  
               gi|115249842|emb|CAJ67659.1|  (rubrerythrin [Clostridium difficile 630]) 
           
  Protein Group 87   
      Expression Quality:  
         Score      Num Spectra      Num Peptides      High-Qual Peptides      % Coverage       160    5    3    3    5   
   
      Peptides:   
        Query    Observed    Mr(expt)    Mr(calc)    Score    Peptide    Result File   
		    317    543.35    1627.03    1626.77    40    IKEHNHNVGTCYR    41_8   
		    109    490.81    979.61    979.46    58    LYGEDANAK    41_8   
		    356    1122.79    2243.57    2243.14    62    SLGNGIDPLEIIEQYGADALR    41A_8   
   
      Matching Genes:  
               gi|115252312|emb|CAJ70153.1|  (valyl-tRNA synthetase [Clostridium difficile 630]) 
           
  Protein Group 88   
      Expression Quality:  
         Score      Num Spectra      Num Peptides      High-Qual Peptides      % Coverage       159    9    4    2    40   
   
      Peptides:   
        Query    Observed    Mr(expt)    Mr(calc)    Score    Peptide    Result File   
		    93    457.35    912.68    912.56    50    AVLELAGLK    41A_2   
		    379    637.50    1909.47    1909.01    32    FAALVVVGDENGHVGIGAGK    biorep41CdiffA_3   
		    716    1232.01    2462.01    2461.29    29    GHFGAGNILIMPAVEGTGVIAGGPAR    biorep41Cdiff_3   
		    546    806.27    1610.53    1609.88    48    RKPIDAGQLDLQEK    biorep41Cdiff_3   
   
      Matching Genes:  
               gi|115249094|emb|CAJ66905.1|  (30S ribosomal protein S5 [Clostridium difficile 630]) 
           
  Protein Group 89   
      Expression Quality:  
         Score      Num Spectra      Num Peptides      High-Qual Peptides      % Coverage       159    3    3    2    8   
   
      Peptides:   
        Query    Observed    Mr(expt)    Mr(calc)    Score    Peptide    Result File   
		    240    788.52    1575.02    1574.66    59    EFECYTQEQVDK    41A_6   
		    178    422.68    1265.01    1264.65    27    GHSVSIHSNTVK    41_6   
		    212    747.52    1493.03    1492.72    73    NIEYAGENIEVSR    41A_6   
   
      Matching Genes:  
               gi|115251397|emb|CAJ69229.1|  (succinate-semialdehyde dehydrogenase [NAD(P)+] [Clostridium difficile 630]) 
           
  Protein Group 90   
      Expression Quality:  
         Score      Num Spectra      Num Peptides      High-Qual Peptides      % Coverage       159    7    3    3    32   
   
      Peptides:   
        Query    Observed    Mr(expt)    Mr(calc)    Score    Peptide    Result File   
		    229    580.40    1158.78    1158.61    55    EVVATDGEIVK    41_1   
		    298    685.13    2052.37    2052.02    53    NYELVYVVKPNSDEEVR    41A_1   
		    350    694.01    1386.00    1385.78    51    VKEVVATDGEIVK    41_1   
   
      Matching Genes:  
               gi|115252728|emb|CAJ70572.1|  (30S ribosomal protein S6 [Clostridium difficile 630]) 
           
  Protein Group 91   
      Expression Quality:  
         Score      Num Spectra      Num Peptides      High-Qual Peptides      % Coverage       156    4    3    2    29   
   
      Peptides:   
        Query    Observed    Mr(expt)    Mr(calc)    Score    Peptide    Result File   
		    386    738.04    1474.08    1473.79    71    IGVIGGGSITYPNAR    41_1   
		    228    572.38    1142.75    1142.52    60    LSETDEFFR    41_1   
		    161    478.27    954.52    954.47    25    TYSEGAISK    41_1   
   
      Matching Genes:  
               gi|115251040|emb|CAJ68871.1|  (putative decarboxylase [Clostridium difficile 630]) 
           
  Protein Group 92   
      Expression Quality:  
         Score      Num Spectra      Num Peptides      High-Qual Peptides      % Coverage       151    4    3    1    3   
   
      Peptides:   
        Query    Observed    Mr(expt)    Mr(calc)    Score    Peptide    Result File   
		    153    567.40    1132.78    1133.52    26    GETELTAEER    41_8   
		    192    817.26    1632.50    1631.85    32    STGPYSLVTQQPLGGK    biorep41CdiffA_8   
		    185    723.47    1444.93    1444.70    93    VLTDEDQEIEVR    41A_8   
   
      Matching Genes:  
               gi|115249070|emb|CAJ66881.1|  (DNA-directed RNA polymerase beta chain [Clostridium difficile 630]) 
           
  Protein Group 93   
      Expression Quality:  
         Score      Num Spectra      Num Peptides      High-Qual Peptides      % Coverage       150    3    2    2    15   
   
      Peptides:   
        Query    Observed    Mr(expt)    Mr(calc)    Score    Peptide    Result File   
		    339    706.98    1411.95    1411.68    69    QNFGQVSNSYIR    41_3   
		    461    889.04    1776.07    1775.81    81    TGEGDGDDEQIVVDLSK    41_3   
   
      Matching Genes:  
               gi|115250675|emb|CAJ68499.1|  (tellurium resistance protein [Clostridium difficile 630]) 
           
  Protein Group 94   
      Expression Quality:  
         Score      Num Spectra      Num Peptides      High-Qual Peptides      % Coverage       148    3    2    2    39   
   
      Peptides:   
        Query    Observed    Mr(expt)    Mr(calc)    Score    Peptide    Result File   
		    56    456.33    910.66    910.50    54    GPQAANVVR    41A_1   
		    466    919.13    1836.24    1835.88    94    TLEEGQSVEFEVVDGAK    41_1   
   
      Matching Genes:  
               gi|115250391|emb|CAJ68213.1|  (putative cold shock protein [Clostridium difficile 630]) 
           
  Protein Group 95   
      Expression Quality:  
         Score      Num Spectra      Num Peptides      High-Qual Peptides      % Coverage       146    6    3    1    15   
   
      Peptides:   
        Query    Observed    Mr(expt)    Mr(calc)    Score    Peptide    Result File   
		    24    416.90    831.79    831.47    29    IDVTGISK    biorep41Cdiff_3   
		    393    762.51    1523.00    1522.75    80    VDSVEGYTVGQEIK    41_3   
		    152    570.53    1139.04    1138.57    37    VGMTQIFTDK    biorep41Cdiff_4   
   
      Matching Genes:  
               gi|115249077|emb|CAJ66888.1|  (50S ribosomal protein L3 [Clostridium difficile 630]) 
           
  Protein Group 96   
      Expression Quality:  
         Score      Num Spectra      Num Peptides      High-Qual Peptides      % Coverage       145    4    3    2    12   
   
      Peptides:   
        Query    Observed    Mr(expt)    Mr(calc)    Score    Peptide    Result File   
		    222    726.01    1450.00    1449.75    57    GFFPEEELITLR    41A_4   
		    123    627.95    1253.89    1253.81    26    KVTGKPTVIIAK    41A_4   
		    177    539.77    1077.53    1077.46    62    MNVCAENPK +Oxidation (M)    41_4   
   
      Matching Genes:  
               gi|115251377|emb|CAJ69209.1|  (transketolase [Clostridium difficile 630]) 
           
  Protein Group 97   
      Expression Quality:  
         Score      Num Spectra      Num Peptides      High-Qual Peptides      % Coverage       139    4    3    3    42   
   
      Peptides:   
        Query    Observed    Mr(expt)    Mr(calc)    Score    Peptide    Result File   
		    590    1002.33    2002.65    2002.00    40    LEANAGDVVTLNEVLACSK    biorep41Cdiff_1   
		    356    671.44    1340.86    1340.73    41    LGSPVVEGASVQAK    biorep41Cdiff_1   
		    117    611.37    1220.73    1220.63    58    VSEGDVLFVEK    41A_1   
   
      Matching Genes:  
               gi|115250193|emb|CAJ68014.1|  (50S ribosomal protein L21 [Clostridium difficile 630]) 
           
  Protein Group 98   
      Expression Quality:  
         Score      Num Spectra      Num Peptides      High-Qual Peptides      % Coverage       137    5    3    2    26   
   
      Peptides:   
        Query    Observed    Mr(expt)    Mr(calc)    Score    Peptide    Result File   
		    70    478.48    954.95    954.55    51    KYTFVVAK    biorep41CdiffA_1   
		    13    425.71    849.40    849.46    33    VFGVSVDK    biorep41CdiffA_1   
		    76    512.35    1022.69    1022.50    53    VNTLNYDGK    41A_1   
   
      Matching Genes:  
               gi|115249079|emb|CAJ66890.1|  (50S ribosomal protein L23 [Clostridium difficile 630]) 
           
  Protein Group 99   
      Expression Quality:  
         Score      Num Spectra      Num Peptides      High-Qual Peptides      % Coverage       137    7    3    2    26   
   
      Peptides:   
        Query    Observed    Mr(expt)    Mr(calc)    Score    Peptide    Result File   
		    278    584.41    1750.21    1749.89    29    HQKPSAMNQQGGIINK    biorep41CdiffA_1   
		    447    589.71    1766.11    1765.89    47    HQKPSAMNQQGGIINK +Oxidation (M)    41_1   
		    231    585.91    1169.81    1169.70    61    VLVEGVNVITK    41_1   
   
      Matching Genes:  
               gi|115249088|emb|CAJ66899.1|  (50S ribosomal protein L24 [Clostridium difficile 630]) 
           
  Protein Group 100   
      Expression Quality:  
         Score      Num Spectra      Num Peptides      High-Qual Peptides      % Coverage       136    5    2    1    10   
   
      Peptides:   
        Query    Observed    Mr(expt)    Mr(calc)    Score    Peptide    Result File   
		    261    558.38    1672.11    1671.87    35    IADELTQLKDEIER    41A_3   
		    504    910.11    1818.20    1817.88    101    TLSLQSANEINNTEER    41_4   
   
      Matching Genes:  
               gi|115249247|emb|CAJ67060.1|  (flagellin subunit [Clostridium difficile 630]) 
           
  Protein Group 101   
      Expression Quality:  
         Score      Num Spectra      Num Peptides      High-Qual Peptides      % Coverage       136    4    3    1    5   
   
      Peptides:   
        Query    Observed    Mr(expt)    Mr(calc)    Score    Peptide    Result File   
		    186    721.50    1440.98    1440.76    32    GVVDVYPNKPEPK    41A_7   
		    104    530.38    1058.74    1058.56    39    LVTLDDVER    41_7   
		    352    962.15    1922.29    1921.94    65    SLGLAGVMGGANSEITSNTK +Oxidation (M)    41_7   
   
      Matching Genes:  
               gi|115249716|emb|CAJ67533.1|  (phenylalanyl-tRNA synthetase beta chain [Clostridium difficile 630]) 
           
  Protein Group 102   
      Expression Quality:  
         Score      Num Spectra      Num Peptides      High-Qual Peptides      % Coverage       135    5    3    2    20   
   
      Peptides:   
        Query    Observed    Mr(expt)    Mr(calc)    Score    Peptide    Result File   
		    538    797.30    1592.59    1591.86    53    APVSNFAYLIDAIAK    biorep41Cdiff_3   
		    262    565.58    1129.14    1128.60    36    IVEMANIPSR    biorep41Cdiff_3   
		    103    559.90    1117.78    1117.59    46    SEVVSEIVEK    41A_3   
   
      Matching Genes:  
               gi|115249067|emb|CAJ66878.1|  (50S ribosomal protein L10 [Clostridium difficile 630]) 
           
  Protein Group 103   
      Expression Quality:  
         Score      Num Spectra      Num Peptides      High-Qual Peptides      % Coverage       135    5    3    2    39   
   
      Peptides:   
        Query    Observed    Mr(expt)    Mr(calc)    Score    Peptide    Result File   
		    465    914.61    1827.21    1826.94    63    AEGDTGSPEVQIALLTAR    41_1   
		    82    519.36    1036.70    1036.57    42    INELNGHLK    41A_1   
		    36    418.07    834.12    833.50    30    NLLAYLK    biorep41Cdiff_1   
   
      Matching Genes:  
               gi|115250352|emb|CAJ68174.1|  (30S ribosomal protein S15 [Clostridium difficile 630]) 
           
  Protein Group 104   
      Expression Quality:  
         Score      Num Spectra      Num Peptides      High-Qual Peptides      % Coverage       133    3    2    2    13   
   
      Peptides:   
        Query    Observed    Mr(expt)    Mr(calc)    Score    Peptide    Result File   
		    339    706.98    1411.95    1411.68    69    QNFGQVSNSYIR    41_3   
		    284    887.59    1773.17    1772.86    64    VEKDEDFIFYNNLK    41A_3   
   
      Matching Genes:  
               gi|115250676|emb|CAJ68500.1|  (tellurium resistance protein [Clostridium difficile 630]) 
           
  Protein Group 105   
      Expression Quality:  
         Score      Num Spectra      Num Peptides      High-Qual Peptides      % Coverage       132    8    3    2    25   
   
      Peptides:   
        Query    Observed    Mr(expt)    Mr(calc)    Score    Peptide    Result File   
		    360    574.77    1721.28    1720.92    36    KPEEVISHAVSGMLPK    41A_2   
		    236    544.07    1086.13    1085.64    55    LATEIATVLR    biorep41Cdiff_3   
		    143    547.24    1092.47    1093.56    41    YHTGYVGGLK    41A_2   
   
      Matching Genes:  
               gi|115249112|emb|CAJ66923.1|  (50S ribosomal protein L13 [Clostridium difficile 630]) 
           
  Protein Group 106   
      Expression Quality:  
         Score      Num Spectra      Num Peptides      High-Qual Peptides      % Coverage       129    3    3    2    10   
   
      Peptides:   
        Query    Observed    Mr(expt)    Mr(calc)    Score    Peptide    Result File   
		    237    778.01    1554.00    1553.78    56    GIPVSIGTDGAPSNNR    41A_6   
		    304    817.64    1633.27    1632.75    31    SVMDTGDGLPEAWQK    41_6   
		    233    775.05    1548.08    1547.83    42    VVLGFASIPEMLEK +Oxidation (M)    41A_6   
   
      Matching Genes:  
               gi|115251756|emb|CAJ69591.1|  (putative amidohydrolas [Clostridium difficile 630]) 
           
  Protein Group 107   
      Expression Quality:  
         Score      Num Spectra      Num Peptides      High-Qual Peptides      % Coverage       127    4    3    1    27   
   
      Peptides:   
        Query    Observed    Mr(expt)    Mr(calc)    Score    Peptide    Result File   
		    224    518.50    1034.99    1034.54    35    GITEVVFDR    biorep41Cdiff_2   
		    69    493.84    985.67    985.52    62    IQELAEGAR    41A_1   
		    626    574.85    1721.52    1720.87    30    SANNIYAQIIDDTKR    biorep41Cdiff_2   
   
      Matching Genes:  
               gi|115249093|emb|CAJ66904.1|  (50S ribosomal protein L18 [Clostridium difficile 630]) 
           
  Protein Group 108   
      Expression Quality:  
         Score      Num Spectra      Num Peptides      High-Qual Peptides      % Coverage       127    5    3    1    4   
   
      Peptides:   
        Query    Observed    Mr(expt)    Mr(calc)    Score    Peptide    Result File   
		    396    699.48    2095.43    2095.02    37    IDYIVVSHTEPDHAGSVEK    41_7   
		    73    522.38    1042.74    1042.60    29    TIIVNDGAIK    41A_7   
		    46    459.38    916.75    916.52    61    VVASETAIK    41A_7   
   
      Matching Genes:  
               gi|115250664|emb|CAJ68488.1|  (putative nitric oxide reductase flavoprotein [Clostridium difficile 630]) 
           
  Protein Group 109   
      Expression Quality:  
         Score      Num Spectra      Num Peptides      High-Qual Peptides      % Coverage       125    3    3    1    19   
   
      Peptides:   
        Query    Observed    Mr(expt)    Mr(calc)    Score    Peptide    Result File   
		    142    636.20    1270.38    1269.73    29    GKNVDEALAILK    biorep41CdiffA_1   
		    115    580.37    1158.73    1158.62    57    KAGQICDLVR    biorep41CdiffA_1   
		    101    543.81    1085.61    1084.61    39    NVDEALAILK    biorep41CdiffA_1   
   
      Matching Genes:  
               gi|115249082|emb|CAJ66893.1|  (50S ribosomal protein L22 [Clostridium difficile 630]) 
           
  Protein Group 110   
      Expression Quality:  
         Score      Num Spectra      Num Peptides      High-Qual Peptides      % Coverage       125    6    2    2    16   
   
      Peptides:   
        Query    Observed    Mr(expt)    Mr(calc)    Score    Peptide    Result File   
		    309    730.54    1459.06    1458.78    80    ILGGGLPYESAVQR    41A_2   
		    134    530.83    1059.65    1058.60    45    VKVETGIDAK    41A_2   
   
      Matching Genes:  
               gi|115249807|emb|CAJ67624.1|  (putative NUDIX-family hydrolase [Clostridium difficile 630]) 
           
  Protein Group 111   
      Expression Quality:  
         Score      Num Spectra      Num Peptides      High-Qual Peptides      % Coverage       125    4    3    1    17   
   
      Peptides:   
        Query    Observed    Mr(expt)    Mr(calc)    Score    Peptide    Result File   
		    114    487.30    972.58    972.54    27    GLHDYIKK    41A_2   
		    132    530.32    1058.63    1058.61    64    LASGFPIGLGK    41A_2   
		    194    529.37    1056.73    1056.49    34    NISEEYFR    41_2   
   
      Matching Genes:  
               gi|115252075|emb|CAJ69912.1|  (conserved hypothetical protein [Clostridium difficile 630]) 
           
  Protein Group 112   
      Expression Quality:  
         Score      Num Spectra      Num Peptides      High-Qual Peptides      % Coverage       122    4    3    1    10   
   
      Peptides:   
        Query    Observed    Mr(expt)    Mr(calc)    Score    Peptide    Result File   
		    327    738.54    1475.07    1474.85    33    QVGVPYIVVFLNK    biorep41Cdiff_5   
		    66    460.46    918.91    918.54    26    TTLTAAITK    biorep41Cdiff_5   
		    381    1083.31    2164.61    2164.04    63    YQLGEAVDFANIDKAPEER    41_5   
   
      Matching Genes:  
               gi|115249061|emb|CAJ66872.1|  (elongation factor TU [Clostridium difficile 630]) 
              Other Genes Matching Peptide Subset:  
               gi|115249075|emb|CAJ66886.1|  (elongation factor TU [Clostridium difficile 630]) 
           
  Protein Group 113   
      Expression Quality:  
         Score      Num Spectra      Num Peptides      High-Qual Peptides      % Coverage       117    3    2    2    5   
   
      Peptides:   
        Query    Observed    Mr(expt)    Mr(calc)    Score    Peptide    Result File   
		    437    1242.23    2482.44    2482.19    54    EALEELGLPYTINEGDGAFYGPK    41A_7   
		    109    551.85    1101.68    1101.54    63    EVADNNVSVR    41_7   
   
      Matching Genes:  
               gi|115249589|emb|CAJ67406.1|  (threonyl-tRNA synthetase [Clostridium difficile 630]) 
           
  Protein Group 114   
      Expression Quality:  
         Score      Num Spectra      Num Peptides      High-Qual Peptides      % Coverage       117    3    2    1    6   
   
      Peptides:   
        Query    Observed    Mr(expt)    Mr(calc)    Score    Peptide    Result File   
		    72    520.41    1038.80    1038.62    34    AEAIVKPGVR    41A_7   
		    171    681.07    1360.12    1359.78    83    FLLNLIELGGGSK    41_5   
   
      Matching Genes:  
               gi|115251664|emb|CAJ69499.1|  (probable peptidase [Clostridium difficile 630]) 
           
  Protein Group 115   
      Expression Quality:  
         Score      Num Spectra      Num Peptides      High-Qual Peptides      % Coverage       115    2    2    2    8   
   
      Peptides:   
        Query    Observed    Mr(expt)    Mr(calc)    Score    Peptide    Result File   
		    240    504.37    1510.10    1509.83    53    ISSKPIIATHSNSR    41A_4   
		    159    654.96    1307.90    1307.63    62    LMENVETSELK +Oxidation (M)    41A_4   
   
      Matching Genes:  
               gi|115252633|emb|CAJ70476.1|  (probable dipeptidase [Clostridium difficile 630]) 
           
  Protein Group 116   
      Expression Quality:  
         Score      Num Spectra      Num Peptides      High-Qual Peptides      % Coverage       112    2    2    2    10   
   
      Peptides:   
        Query    Observed    Mr(expt)    Mr(calc)    Score    Peptide    Result File   
		    417    688.17    1374.32    1373.76    61    ALQATGLEVTMIK    biorep41Cdiff_2   
		    430    696.10    1390.19    1389.75    51    ALQATGLEVTMIK +Oxidation (M)    biorep41Cdiff_2   
   
      Matching Genes:  
               gi|115249104|emb|CAJ66915.1|  (30S ribosomal protein S11 [Clostridium difficile 630]) 
           
  Protein Group 117   
      Expression Quality:  
         Score      Num Spectra      Num Peptides      High-Qual Peptides      % Coverage       112    3    2    1    7   
   
      Peptides:   
        Query    Observed    Mr(expt)    Mr(calc)    Score    Peptide    Result File   
		    238    782.50    1562.98    1562.66    35    AVTSSDGMTSDWYK +Oxidation (M)    41A_6   
		    385    1084.27    2166.52    2166.08    77    GIIFTGGPNSAYLEDSPTISK    41A_6   
   
      Matching Genes:  
               gi|115249206|emb|CAJ67019.1|  (GMP synthase [glutamine-hydrolyzing] [Clostridium difficile 630]) 
           
  Protein Group 118   
      Expression Quality:  
         Score      Num Spectra      Num Peptides      High-Qual Peptides      % Coverage       112    3    2    1    30   
   
      Peptides:   
        Query    Observed    Mr(expt)    Mr(calc)    Score    Peptide    Result File   
		    169    686.45    1370.88    1371.59    80    CACGNTFVAGSTK    41A_1   
		    88    438.77    875.52    875.45    32    YNPVEVR    41_1   
   
      Matching Genes:  
               gi|115252547|emb|CAJ70390.1|  (50S ribosomal protein L31 [Clostridium difficile 630]) 
           
  Protein Group 119   
      Expression Quality:  
         Score      Num Spectra      Num Peptides      High-Qual Peptides      % Coverage       111    3    2    2    14   
   
      Peptides:   
        Query    Observed    Mr(expt)    Mr(calc)    Score    Peptide    Result File   
		    537    1068.71    2135.41    2135.02    54    EVAFSVVDEISPEEIEESK    41_3   
		    83    507.87    1013.72    1013.58    57    INELIDVAK    41A_3   
   
      Matching Genes:  
               gi|115249398|emb|CAJ67213.1|  (Radical SAM-superfamily protein [Clostridium difficile 630]) 
           
  Protein Group 120   
      Expression Quality:  
         Score      Num Spectra      Num Peptides      High-Qual Peptides      % Coverage       111    1    1    1    9   
   
      Peptides:   
        Query    Observed    Mr(expt)    Mr(calc)    Score    Peptide    Result File   
		    282    881.63    1761.25    1760.81    111    TGVGDGDDEQINVDLSK    41A_3   
   
      Matching Genes:  
               gi|115250845|emb|CAJ68669.1|  (tellurium resistance protein [Clostridium difficile 630]) 
           
  Protein Group 121   
      Expression Quality:  
         Score      Num Spectra      Num Peptides      High-Qual Peptides      % Coverage       106    5    2    1    29   
   
      Peptides:   
        Query    Observed    Mr(expt)    Mr(calc)    Score    Peptide    Result File   
		    188    724.95    1447.88    1447.74    74    FQLATGQLENTAR    41A_1   
		    15    426.55    851.09    850.45    32    SELFSLR    biorep41CdiffA_1   
   
      Matching Genes:  
               gi|115249085|emb|CAJ66896.1|  (50S ribosomal protein L29 [Clostridium difficile 630]) 
           
  Protein Group 122   
      Expression Quality:  
         Score      Num Spectra      Num Peptides      High-Qual Peptides      % Coverage       103    4    3    0    26   
   
      Peptides:   
        Query    Observed    Mr(expt)    Mr(calc)    Score    Peptide    Result File   
		    162    479.85    957.68    957.51    35    AIEQEQLK    41_2   
		    263    566.54    1131.07    1130.67    34    IQVFEGVVLK    biorep41Cdiff_2   
		    412    687.53    1373.04    1372.66    34    NEVPNFGPGDTVK    biorep41Cdiff_2   
   
      Matching Genes:  
               gi|115250291|emb|CAJ68113.1|  (50S ribosomal protein L19 [Clostridium difficile 630]) 
           
  Protein Group 123   
      Expression Quality:  
         Score      Num Spectra      Num Peptides      High-Qual Peptides      % Coverage       101    4    2    2    14   
   
      Peptides:   
        Query    Observed    Mr(expt)    Mr(calc)    Score    Peptide    Result File   
		    618    929.91    1857.80    1856.99    45    GVEELEMISGQKPVITK    biorep41Cdiff_3   
		    20    435.79    869.56    869.53    56    LVSVSLPR    biorep41CdiffA_3   
   
      Matching Genes:  
               gi|115249089|emb|CAJ66900.1|  (50S ribosomal protein L5 [Clostridium difficile 630]) 
           
  Protein Group 124   
      Expression Quality:  
         Score      Num Spectra      Num Peptides      High-Qual Peptides      % Coverage       101    3    3    1    4   
   
      Peptides:   
        Query    Observed    Mr(expt)    Mr(calc)    Score    Peptide    Result File   
		    68    529.33    1056.65    1056.55    25    GDVPEGLKDK    41A_8   
		    131    529.37    1056.72    1057.58    43    TAIVEGLAER    41_8   
		    289    749.13    1496.24    1495.69    33    VYGEGADSQGVSATR    biorep41Cdiff_7   
   
      Matching Genes:  
               gi|115251074|emb|CAJ68905.1|  (chaperone [Clostridium difficile 630]) 
           
  Protein Group 125   
      Expression Quality:  
         Score      Num Spectra      Num Peptides      High-Qual Peptides      % Coverage       99    3    2    1    11   
   
      Peptides:   
        Query    Observed    Mr(expt)    Mr(calc)    Score    Peptide    Result File   
		    475    869.07    1736.12    1735.76    72    ADIDYGFAEADTTYGK    41_4   
		    280    832.61    1663.21    1662.82    27    TEGYSEGNVPLQTLR    41A_4   
   
      Matching Genes:  
               gi|115249083|emb|CAJ66894.1|  (30S ribosomal protein S3 [Clostridium difficile 630]) 
           
  Protein Group 126   
      Expression Quality:  
         Score      Num Spectra      Num Peptides      High-Qual Peptides      % Coverage       97    3    3    0    9   
   
      Peptides:   
        Query    Observed    Mr(expt)    Mr(calc)    Score    Peptide    Result File   
		    166    609.58    1217.14    1216.64    30    EILQEVEVMK    biorep41Cdiff_5   
		    86    487.74    973.46    973.51    31    ENLDDIKK    41_6   
		    457    1242.25    2482.49    2482.21    36    IPNVPHPEVPQGETDEDNVQIR    41A_6   
   
      Matching Genes:  
               gi|115249017|emb|CAJ66828.1|  (seryl-tRNA synthetase [Clostridium difficile 630]) 
           
  Protein Group 127   
      Expression Quality:  
         Score      Num Spectra      Num Peptides      High-Qual Peptides      % Coverage       96    4    2    2    8   
   
      Peptides:   
        Query    Observed    Mr(expt)    Mr(calc)    Score    Peptide    Result File   
		    144    500.77    999.52    999.51    43    NGAQEVINR    41_3   
		    70    482.82    963.62    963.50    53    YLGDLLDR    41A_3   
   
      Matching Genes:  
               gi|115249180|emb|CAJ66992.1|  (putative DNA-binding protein [Clostridium difficile 630]) 
           
  Protein Group 128   
      Expression Quality:  
         Score      Num Spectra      Num Peptides      High-Qual Peptides      % Coverage       95    2    2    1    5   
   
      Peptides:   
        Query    Observed    Mr(expt)    Mr(calc)    Score    Peptide    Result File   
		    191    734.07    1466.13    1465.80    31    EVVESNPIPELLK    41A_7   
		    222    788.24    1574.47    1573.84    64    VVFLEDALNQAIDK    41A_7   
   
      Matching Genes:  
               gi|115249727|emb|CAJ67544.1|  (arginyl-tRNA synthetase [Clostridium difficile 630]) 
           
  Protein Group 129   
      Expression Quality:  
         Score      Num Spectra      Num Peptides      High-Qual Peptides      % Coverage       93    4    1    1    10   
   
      Peptides:   
        Query    Observed    Mr(expt)    Mr(calc)    Score    Peptide    Result File   
		    487    935.55    1869.09    1868.91    93    LAGEGGLFFVDQEFANR    41_3   
   
      Matching Genes:  
               gi|115251248|emb|CAJ69079.1|  (ferritin [Clostridium difficile 630]) 
           
  Protein Group 130   
      Expression Quality:  
         Score      Num Spectra      Num Peptides      High-Qual Peptides      % Coverage       92    2    2    1    2   
   
      Peptides:   
        Query    Observed    Mr(expt)    Mr(calc)    Score    Peptide    Result File   
		    135    645.00    1287.99    1287.67    64    TNDEVNVVSIAK    41A_7   
		    22    427.32    852.62    853.42    28    TYNISEK    41A_7   
   
      Matching Genes:  
               gi|115249009|emb|CAJ66820.1|  (DNA gyrase subunit A [Clostridium difficile 630]) 
           
  Protein Group 131   
      Expression Quality:  
         Score      Num Spectra      Num Peptides      High-Qual Peptides      % Coverage       92    3    2    1    9   
   
      Peptides:   
        Query    Observed    Mr(expt)    Mr(calc)    Score    Peptide    Result File   
		    58    432.30    862.58    861.44    26    NMITNAAK    41_3   
		    146    503.78    1005.54    1005.51    66    VPGATYAEAK    41_3   
   
      Matching Genes:  
               gi|115251487|emb|CAJ69320.1|  (conserved hypothetical protein [Clostridium difficile 630]) 
           
  Protein Group 132   
      Expression Quality:  
         Score      Num Spectra      Num Peptides      High-Qual Peptides      % Coverage       89    2    2    1    11   
   
      Peptides:   
        Query    Observed    Mr(expt)    Mr(calc)    Score    Peptide    Result File   
		    140    625.50    1248.99    1248.67    50    LAVENNVSYLK    41A_3   
		    149    474.85    947.69    947.46    39    MAVNPNFR    biorep41Cdiff_3   
   
      Matching Genes:  
               gi|115249853|emb|CAJ67670.1|  (putative acetyltransferase [Clostridium difficile 630]) 
           
  Protein Group 133   
      Expression Quality:  
         Score      Num Spectra      Num Peptides      High-Qual Peptides      % Coverage       88    3    2    2    7   
   
      Peptides:   
        Query    Observed    Mr(expt)    Mr(calc)    Score    Peptide    Result File   
		    187    556.84    1111.66    1111.51    44    GSGSSFIENSK    41_4   
		    418    780.00    1557.99    1557.75    44    VLDCTPGACEVLPK    41_4   
   
      Matching Genes:  
               gi|115250297|emb|CAJ68119.1|  (putative FMN-dependent dehydrogenase [Clostridium difficile 630]) 
           
  Protein Group 134   
      Expression Quality:  
         Score      Num Spectra      Num Peptides      High-Qual Peptides      % Coverage       88    4    2    1    28   
   
      Peptides:   
        Query    Observed    Mr(expt)    Mr(calc)    Score    Peptide    Result File   
		    262    905.20    1808.38    1807.91    57    SIMGIMSLGLAQGEELK +2 Oxidation (M)    41A_1   
		    16    416.82    831.63    831.47    31    STVEVVAK    41A_1   
   
      Matching Genes:  
               gi|115251809|emb|CAJ69644.1|  (PTS system, phosphocarrier protein [Clostridium difficile 630]) 
           
  Protein Group 135   
      Expression Quality:  
         Score      Num Spectra      Num Peptides      High-Qual Peptides      % Coverage       85    2    2    1    4   
   
      Peptides:   
        Query    Observed    Mr(expt)    Mr(calc)    Score    Peptide    Result File   
		    277    911.15    1820.28    1819.88    48    SLYSSSEEPVTPPSNVK    41A_7   
		    44    438.30    874.59    874.48    37    VITNDVSK    41_7   
   
      Matching Genes:  
               gi|115251820|emb|CAJ69655.1|  (cell surface protein [Clostridium difficile 630]) 
           
  Protein Group 136   
      Expression Quality:  
         Score      Num Spectra      Num Peptides      High-Qual Peptides      % Coverage       85    4    2    2    10   
   
      Peptides:   
        Query    Observed    Mr(expt)    Mr(calc)    Score    Peptide    Result File   
		    103    579.36    1156.71    1156.54    44    MIFSNESVSK +Oxidation (M)    41A_4   
		    436    795.82    2384.45    2384.26    41    NVILLDDMIDTAGTIVNAANALK    41A_4   
   
      Matching Genes:  
               gi|115252575|emb|CAJ70418.1|  (ribose-phosphate pyrophosphokinase [Clostridium difficile 630]) 
           
  Protein Group 137   
      Expression Quality:  
         Score      Num Spectra      Num Peptides      High-Qual Peptides      % Coverage       84    1    1    1    3   
   
      Peptides:   
        Query    Observed    Mr(expt)    Mr(calc)    Score    Peptide    Result File   
		    260    931.16    1860.30    1859.92    84    AVASGAACYLHVVENTAK    41A_8   
   
      Matching Genes:  
               gi|115249184|emb|CAJ66996.1|  (putative oxidoreductase, acetyl-CoA synthase subunit [Clostridium difficile 630]) 
           
  Protein Group 138   
      Expression Quality:  
         Score      Num Spectra      Num Peptides      High-Qual Peptides      % Coverage       84    1    1    1    5   
   
      Peptides:   
        Query    Observed    Mr(expt)    Mr(calc)    Score    Peptide    Result File   
		    223    727.50    1452.98    1452.71    84    ALLDAVNTGDYSSK    41A_4   
   
      Matching Genes:  
               gi|115252096|emb|CAJ69934.1|  (proline iminopeptidase [Clostridium difficile 630]) 
           
  Protein Group 139   
      Expression Quality:  
         Score      Num Spectra      Num Peptides      High-Qual Peptides      % Coverage       83    3    2    1    8   
   
      Peptides:   
        Query    Observed    Mr(expt)    Mr(calc)    Score    Peptide    Result File   
		    19    425.23    848.44    849.46    30    DFSILGAK    41A_4   
		    241    756.61    1511.20    1510.81    53    VVEPDILIEEVEK    41A_4   
   
      Matching Genes:  
               gi|115250078|emb|CAJ67898.1|  (3-hydroxybutyryl-CoA dehydratase [Clostridium difficile 630]) 
           
  Protein Group 140   
      Expression Quality:  
         Score      Num Spectra      Num Peptides      High-Qual Peptides      % Coverage       81    2    2    1    22   
   
      Peptides:   
        Query    Observed    Mr(expt)    Mr(calc)    Score    Peptide    Result File   
		    319    652.00    1301.98    1301.68    27    EVEASVGGGAVTVK    41_2   
		    166    682.90    1363.78    1364.62    54    NIDDIQASQMSK +Oxidation (M)    41A_1   
   
      Matching Genes:  
               gi|115249020|emb|CAJ66831.1|  (conserved hypothetical protein [Clostridium difficile 630]) 
           
  Protein Group 141   
      Expression Quality:  
         Score      Num Spectra      Num Peptides      High-Qual Peptides      % Coverage       81    1    1    1    4   
   
      Peptides:   
        Query    Observed    Mr(expt)    Mr(calc)    Score    Peptide    Result File   
		    270    909.23    1816.45    1815.97    81    ILVINNPSNPTGSVYTK    41_5   
   
      Matching Genes:  
               gi|115249115|emb|CAJ66926.1|  (aspartate aminotransferase [Clostridium difficile 630]) 
           
  Protein Group 142   
      Expression Quality:  
         Score      Num Spectra      Num Peptides      High-Qual Peptides      % Coverage       81    3    2    1    17   
   
      Peptides:   
        Query    Observed    Mr(expt)    Mr(calc)    Score    Peptide    Result File   
		    59    465.84    929.66    929.48    55    ISNESPVGK    41A_3   
		    529    696.13    2085.36    2084.99    26    VAISFGDLSENAEYDEAKK    41_3   
   
      Matching Genes:  
               gi|115252616|emb|CAJ70459.1|  (transcription elongation factor [Clostridium difficile 630]) 
           
  Protein Group 143   
      Expression Quality:  
         Score      Num Spectra      Num Peptides      High-Qual Peptides      % Coverage       80    3    2    1    16   
   
      Peptides:   
        Query    Observed    Mr(expt)    Mr(calc)    Score    Peptide    Result File   
		    248    534.00    1598.98    1598.72    26    NLSDNHESQEAEVK    41A_3   
		    210    739.03    1476.04    1475.76    54    VEAGIYNEILNNK    41A_3   
   
      Matching Genes:  
               gi|115252635|emb|CAJ70478.1|  (putative preprotein translocase [Clostridium difficile 630]) 
           
  Protein Group 144   
      Expression Quality:  
         Score      Num Spectra      Num Peptides      High-Qual Peptides      % Coverage       79    2    1    1    6   
   
      Peptides:   
        Query    Observed    Mr(expt)    Mr(calc)    Score    Peptide    Result File   
		    269    815.68    1629.35    1628.88    79    FGDGGVDILPIANLTK    41A_4   
   
      Matching Genes:  
               gi|115249811|emb|CAJ67628.1|  (NH3-dependent NAD(+) synthetase [Clostridium difficile 630]) 
           
  Protein Group 145   
      Expression Quality:  
         Score      Num Spectra      Num Peptides      High-Qual Peptides      % Coverage       77    2    1    1    4   
   
      Peptides:   
        Query    Observed    Mr(expt)    Mr(calc)    Score    Peptide    Result File   
		    218    779.08    1556.14    1555.76    77    SIQAIDSHTAGEATR    41_5   
   
      Matching Genes:  
               gi|115252294|emb|CAJ70135.1|  (putative proline racemase [Clostridium difficile 630]) 
           
  Protein Group 146   
      Expression Quality:  
         Score      Num Spectra      Num Peptides      High-Qual Peptides      % Coverage       76    2    2    1    6   
   
      Peptides:   
        Query    Observed    Mr(expt)    Mr(calc)    Score    Peptide    Result File   
		    115    603.39    1204.77    1204.54    26    DGHTNLYEEK    41A_1   
		    169    533.28    1064.54    1064.51    50    SEVFNNLDK    41_4   
   
      Matching Genes:  
               gi|115250155|emb|CAJ67976.1|  (nitroreductase-family protein [Clostridium difficile 630]) 
           
  Protein Group 147   
      Expression Quality:  
         Score      Num Spectra      Num Peptides      High-Qual Peptides      % Coverage       76    2    2    1    21   
   
      Peptides:   
        Query    Observed    Mr(expt)    Mr(calc)    Score    Peptide    Result File   
		    429    711.41    2131.21    2131.16    51    IAQMIVKPIYDINIEEVK +Oxidation (M)    41A_2   
		    424    489.99    1466.96    1466.75    25    LNDDAIIPNFAHK    41_2   
   
      Matching Genes:  
               gi|115251455|emb|CAJ69288.1|  (deoxyuridine 5'-triphosphate nucleotidohydrolase [Clostridium difficile 630]) 
           
  Protein Group 148   
      Expression Quality:  
         Score      Num Spectra      Num Peptides      High-Qual Peptides      % Coverage       76    1    1    1    8   
   
      Peptides:   
        Query    Observed    Mr(expt)    Mr(calc)    Score    Peptide    Result File   
		    301    716.36    1430.71    1430.76    76    VTSTGIVNGVIEDK    41A_2   
   
      Matching Genes:  
               gi|115252223|emb|CAJ70063.1|  (putative phosphatidylethanolamine-binding regulatory protein [Clostridium difficile 630]) 
           
  Protein Group 149   
      Expression Quality:  
         Score      Num Spectra      Num Peptides      High-Qual Peptides      % Coverage       75    2    2    1    13   
   
      Peptides:   
        Query    Observed    Mr(expt)    Mr(calc)    Score    Peptide    Result File   
		    428    491.34    1470.99    1470.80    31    IYSSLYLEDLKK    41_2   
		    10    409.76    817.51    817.42    44    LSDGLGEK    41_2   
   
      Matching Genes:  
               gi|115250371|emb|CAJ68193.1|  (MarR-family transcriptional regulator [Clostridium difficile 630]) 
           
  Protein Group 150   
      Expression Quality:  
         Score      Num Spectra      Num Peptides      High-Qual Peptides      % Coverage       75    3    2    1    8   
   
      Peptides:   
        Query    Observed    Mr(expt)    Mr(calc)    Score    Peptide    Result File   
		    107    459.77    917.53    917.48    47    GENIGTTVK    41_3   
		    239    632.37    1262.73    1262.61    28    NVDAVYDKDPK    41_3   
   
      Matching Genes:  
               gi|115251192|emb|CAJ69023.1|  (uridylate kinase [Clostridium difficile 630]) 
           
  Protein Group 151   
      Expression Quality:  
         Score      Num Spectra      Num Peptides      High-Qual Peptides      % Coverage       74    3    2    1    13   
   
      Peptides:   
        Query    Observed    Mr(expt)    Mr(calc)    Score    Peptide    Result File   
		    6    410.30    818.59    818.45    41    IETTVTR    41A_1   
		    78    494.91    987.80    987.55    33    NLVTCLLR    biorep41CdiffA_1   
   
      Matching Genes:  
               gi|115249107|emb|CAJ66918.1|  (50S ribosomal protein L17 [Clostridium difficile 630]) 
           
  Protein Group 152   
      Expression Quality:  
         Score      Num Spectra      Num Peptides      High-Qual Peptides      % Coverage       74    1    1    1    5   
   
      Peptides:   
        Query    Observed    Mr(expt)    Mr(calc)    Score    Peptide    Result File   
		    254    930.14    1858.26    1857.88    74    TIDEDESGALNPELVEK    41A_5   
   
      Matching Genes:  
               gi|115251648|emb|CAJ69481.1|  (low-specificity L-threonine aldolase [Clostridium difficile 630]) 
           
  Protein Group 153   
      Expression Quality:  
         Score      Num Spectra      Num Peptides      High-Qual Peptides      % Coverage       73    1    1    1    6   
   
      Peptides:   
        Query    Observed    Mr(expt)    Mr(calc)    Score    Peptide    Result File   
		    351    1041.34    2080.67    2080.03    73    TLEKPGTNVSGTSDFVSVDK    41_5   
   
      Matching Genes:  
               gi|115249887|emb|CAJ67706.1|  (ABC transporter, substrate-binding lipoprotein [Clostridium difficile 630]) 
           
  Protein Group 154   
      Expression Quality:  
         Score      Num Spectra      Num Peptides      High-Qual Peptides      % Coverage       72    2    2    1    7   
   
      Peptides:   
        Query    Observed    Mr(expt)    Mr(calc)    Score    Peptide    Result File   
		    221    611.38    1220.74    1220.64    47    AYGANLVLTDGK    41_4   
		    118    618.41    1234.81    1235.61    25    TTAVEIMNDVK +Oxidation (M)    41A_4   
   
      Matching Genes:  
               gi|115250635|emb|CAJ68459.1|  (putative O-acetylserine sulfhydrylase [Clostridium difficile 630]) 
           
  Protein Group 155   
      Expression Quality:  
         Score      Num Spectra      Num Peptides      High-Qual Peptides      % Coverage       70    2    2    1    18   
   
      Peptides:   
        Query    Observed    Mr(expt)    Mr(calc)    Score    Peptide    Result File   
		    373    479.64    1435.91    1435.63    40    SLDNGDLDHEHGK    41_1   
		    205    544.35    1086.68    1086.54    30    VCIPVEENK    41_1   
   
      Matching Genes:  
               gi|115250736|emb|CAJ68560.1|  (putative dinitrogenase iron-molybdenum cofactor [Clostridium difficile 630]) 
           
  Protein Group 156   
      Expression Quality:  
         Score      Num Spectra      Num Peptides      High-Qual Peptides      % Coverage       70    1    1    1    3   
   
      Peptides:   
        Query    Observed    Mr(expt)    Mr(calc)    Score    Peptide    Result File   
		    350    686.88    1371.76    1371.70    70    GEADTISAAPVSVR    41_4   
   
      Matching Genes:  
               gi|115251246|emb|CAJ69077.1|  (cell surface protein [Clostridium difficile 630]) 
           
  Protein Group 157   
      Expression Quality:  
         Score      Num Spectra      Num Peptides      High-Qual Peptides      % Coverage       70    1    1    1    15   
   
      Peptides:   
        Query    Observed    Mr(expt)    Mr(calc)    Score    Peptide    Result File   
		    228    839.12    1676.22    1675.83    70    VETQGATGIDNELTTK    41A_1   
   
      Matching Genes:  
               gi|115252071|emb|CAJ69908.1|  (PTS system, IIb component [Clostridium difficile 630]) 
           
  Protein Group 158   
      Expression Quality:  
         Score      Num Spectra      Num Peptides      High-Qual Peptides      % Coverage       70    3    1    1    22   
   
      Peptides:   
        Query    Observed    Mr(expt)    Mr(calc)    Score    Peptide    Result File   
		    391    745.48    1488.95    1488.71    70    EAEEGCPVSAITVK    41_1   
   
      Matching Genes:  
               gi|115252670|emb|CAJ70513.1|  (ferredoxin [Clostridium difficile 630]) 
           
  Protein Group 159   
      Expression Quality:  
         Score      Num Spectra      Num Peptides      High-Qual Peptides      % Coverage       69    1    1    1    16   
   
      Peptides:   
        Query    Observed    Mr(expt)    Mr(calc)    Score    Peptide    Result File   
		    459    900.16    1798.30    1797.88    69    FIEEIGYYNPISEPK    41_1   
   
      Matching Genes:  
               gi|115250287|emb|CAJ68109.1|  (30S ribosomal protein S16 [Clostridium difficile 630]) 
           
  Protein Group 160   
      Expression Quality:  
         Score      Num Spectra      Num Peptides      High-Qual Peptides      % Coverage       69    4    1    1    8   
   
      Peptides:   
        Query    Observed    Mr(expt)    Mr(calc)    Score    Peptide    Result File   
		    520    1022.34    2042.67    2042.21    69    AIEEAGIPTIIIAALPPVVR    41_3   
   
      Matching Genes:  
               gi|115252298|emb|CAJ70139.1|  (proline reductase [Clostridium difficile 630]) 
           
  Protein Group 161   
      Expression Quality:  
         Score      Num Spectra      Num Peptides      High-Qual Peptides      % Coverage       68    1    1    1    12   
   
      Peptides:   
        Query    Observed    Mr(expt)    Mr(calc)    Score    Peptide    Result File   
		    283    636.44    1270.87    1270.69    68    QNINIVDISQK    41_1   
   
      Matching Genes:  
               gi|115251678|emb|CAJ69513.1|  (conserved hypothetical protein [Clostridium difficile 630]) 
           
  Protein Group 162   
      Expression Quality:  
         Score      Num Spectra      Num Peptides      High-Qual Peptides      % Coverage       67    2    2    1    18   
   
      Peptides:   
        Query    Observed    Mr(expt)    Mr(calc)    Score    Peptide    Result File   
		    85    525.30    1048.59    1048.49    42    FEDAVAAGNR    41A_1   
		    262    555.19    1662.56    1661.80    25    FEDAVAAGNREDAVAK    biorep41CdiffA_1   
   
      Matching Genes:  
               gi|115251527|emb|CAJ69360.1|  (30S ribosomal protein S20 [Clostridium difficile 630]) 
           
  Protein Group 163   
      Expression Quality:  
         Score      Num Spectra      Num Peptides      High-Qual Peptides      % Coverage       66    3    2    0    2   
   
      Peptides:   
        Query    Observed    Mr(expt)    Mr(calc)    Score    Peptide    Result File   
		    240    450.32    1347.92    1347.77    33    GRPVTGPGNRPLK    41_8   
		    227    661.50    1321.00    1321.64    33    VIETWTETTDK    41_8   
   
      Matching Genes:  
               gi|115249071|emb|CAJ66882.1|  (DNA-directed RNA polymerase beta' chain [Clostridium difficile 630]) 
           
  Protein Group 164   
      Expression Quality:  
         Score      Num Spectra      Num Peptides      High-Qual Peptides      % Coverage       66    2    1    1    6   
   
      Peptides:   
        Query    Observed    Mr(expt)    Mr(calc)    Score    Peptide    Result File   
		    388    756.55    1511.09    1510.87    66    ALVVIADKNDNVIK    41_3   
   
      Matching Genes:  
               gi|115249078|emb|CAJ66889.1|  (50S ribosomal protein L4 [Clostridium difficile 630]) 
           
  Protein Group 165   
      Expression Quality:  
         Score      Num Spectra      Num Peptides      High-Qual Peptides      % Coverage       65    2    2    0    35   
   
      Peptides:   
        Query    Observed    Mr(expt)    Mr(calc)    Score    Peptide    Result File   
		    217    547.76    1640.25    1639.80    37    HVPVYITEDMVGHK +Oxidation (M)    41A_1   
		    353    1044.84    2087.66    2087.01    28    SSTVFPQMVENTIAVHDGR    biorep41CdiffA_1   
   
      Matching Genes:  
               gi|115249081|emb|CAJ66892.1|  (30S ribosomal protein S19 [Clostridium difficile 630]) 
           
  Protein Group 166   
      Expression Quality:  
         Score      Num Spectra      Num Peptides      High-Qual Peptides      % Coverage       65    1    1    1    12   
   
      Peptides:   
        Query    Observed    Mr(expt)    Mr(calc)    Score    Peptide    Result File   
		    246    863.62    1725.23    1724.79    65    IVDEYDYGYNAIYK    41A_1   
   
      Matching Genes:  
               gi|115249824|emb|CAJ67641.1|  (hypothetical protein [Clostridium difficile 630]) 
           
  Protein Group 167   
      Expression Quality:  
         Score      Num Spectra      Num Peptides      High-Qual Peptides      % Coverage       65    2    2    0    7   
   
      Peptides:   
        Query    Observed    Mr(expt)    Mr(calc)    Score    Peptide    Result File   
		    226    513.37    1537.08    1536.76    31    GQGGNNAGHTLVVEGK    41A_6   
		    217    760.05    1518.08    1517.80    34    IITDFPASLDDLAK    41A_6   
   
      Matching Genes:  
               gi|115252719|emb|CAJ70563.1|  (adenylosuccinate synthetase [Clostridium difficile 630]) 
           
  Protein Group 168   
      Expression Quality:  
         Score      Num Spectra      Num Peptides      High-Qual Peptides      % Coverage       63    2    1    1    3   
   
      Peptides:   
        Query    Observed    Mr(expt)    Mr(calc)    Score    Peptide    Result File   
		    196    486.26    1455.75    1455.65    63    TESMHGAGSPQAQR    41A_6   
   
      Matching Genes:  
               gi|115251396|emb|CAJ69228.1|  (gamma-aminobutyrate metabolism dehydratase/isomerase [includes: 4-hydroxybutyryl-coa dehydratase; vinylacetyl-coa-delta-isomerase] [Clostridium difficile 630]) 
           
  Protein Group 169   
      Expression Quality:  
         Score      Num Spectra      Num Peptides      High-Qual Peptides      % Coverage       62    2    2    0    12   
   
      Peptides:   
        Query    Observed    Mr(expt)    Mr(calc)    Score    Peptide    Result File   
		    605    602.13    1803.36    1802.88    30    EENTIMVERPTDNKK    biorep41Cdiff_3   
		    48    447.28    892.55    892.47    32    YVDEVIR    41A_3   
   
      Matching Genes:  
               gi|115249092|emb|CAJ66903.1|  (50S ribosomal protein L6 [Clostridium difficile 630]) 
           
  Protein Group 170   
      Expression Quality:  
         Score      Num Spectra      Num Peptides      High-Qual Peptides      % Coverage       62    1    1    1    10   
   
      Peptides:   
        Query    Observed    Mr(expt)    Mr(calc)    Score    Peptide    Result File   
		    326    693.90    1385.79    1385.70    62    ELVPNTTDAAVEK    41_3   
   
      Matching Genes:  
               gi|115249844|emb|CAJ67661.1|  (rubredoxin oxidoreductase (desulfoferrodoxin) [Clostridium difficile 630]) 
           
  Protein Group 171   
      Expression Quality:  
         Score      Num Spectra      Num Peptides      High-Qual Peptides      % Coverage       61    2    1    1    12   
   
      Peptides:   
        Query    Observed    Mr(expt)    Mr(calc)    Score    Peptide    Result File   
		    245    861.62    1721.23    1720.82    61    GYDVSSISTPDLENPK    41A_1   
   
      Matching Genes:  
               gi|115249013|emb|CAJ66824.1|  (anti-sigma-B factor (serine-protein kinase) [Clostridium difficile 630]) 
           
  Protein Group 172   
      Expression Quality:  
         Score      Num Spectra      Num Peptides      High-Qual Peptides      % Coverage       60    1    1    1    4   
   
      Peptides:   
        Query    Observed    Mr(expt)    Mr(calc)    Score    Peptide    Result File   
		    144    640.46    1278.90    1278.68    60    IYVIGGENSISK    41A_4   
   
      Matching Genes:  
               gi|115249861|emb|CAJ67678.1|  (cell surface protein [Clostridium difficile 630]) 
           
  Protein Group 173   
      Expression Quality:  
         Score      Num Spectra      Num Peptides      High-Qual Peptides      % Coverage       60    1    1    1    7   
   
      Peptides:   
        Query    Observed    Mr(expt)    Mr(calc)    Score    Peptide    Result File   
		    232    770.05    1538.09    1537.73    60    TSTGMGTGGATLEDIK    41A_3   
   
      Matching Genes:  
               gi|115250542|emb|CAJ68366.1|  (putative deoxyribose-phosphate aldolase [Clostridium difficile 630]) 
           
  Protein Group 174   
      Expression Quality:  
         Score      Num Spectra      Num Peptides      High-Qual Peptides      % Coverage       59    2    1    1    7   
   
      Peptides:   
        Query    Observed    Mr(expt)    Mr(calc)    Score    Peptide    Result File   
		    382    748.06    1494.11    1493.85    59    AGVVLNPATPVDTIK    41_3   
   
      Matching Genes:  
               gi|115251631|emb|CAJ69464.1|  (ribulose-phosphate 3-epimerase [Clostridium difficile 630]) 
           
  Protein Group 175   
      Expression Quality:  
         Score      Num Spectra      Num Peptides      High-Qual Peptides      % Coverage       58    4    1    1    5   
   
      Peptides:   
        Query    Observed    Mr(expt)    Mr(calc)    Score    Peptide    Result File   
		    294    436.98    1307.91    1307.69    58    KENIAIQEAHR    41_4   
   
      Matching Genes:  
               gi|115251194|emb|CAJ69025.1|  (30S ribosomal protein S2 [Clostridium difficile 630]) 
           
  Protein Group 176   
      Expression Quality:  
         Score      Num Spectra      Num Peptides      High-Qual Peptides      % Coverage       58    1    1    1    5   
   
      Peptides:   
        Query    Observed    Mr(expt)    Mr(calc)    Score    Peptide    Result File   
		    346    952.71    1903.40    1902.89    58    SLGPEPWQVCYVEPSR    41A_4   
   
      Matching Genes:  
               gi|115251486|emb|CAJ69319.1|  (glycyl-tRNA synthetase alpha chain [Clostridium difficile 630]) 
           
  Protein Group 177   
      Expression Quality:  
         Score      Num Spectra      Num Peptides      High-Qual Peptides      % Coverage       58    1    1    1    2   
   
      Peptides:   
        Query    Observed    Mr(expt)    Mr(calc)    Score    Peptide    Result File   
		    174    699.04    1396.06    1395.74    58    AGEDQQPIILANK    41A_7   
   
      Matching Genes:  
               gi|115251837|emb|CAJ69672.1|  (cell surface protein [Clostridium difficile 630]) 
           
  Protein Group 178   
      Expression Quality:  
         Score      Num Spectra      Num Peptides      High-Qual Peptides      % Coverage       58    1    1    1    5   
   
      Peptides:   
        Query    Observed    Mr(expt)    Mr(calc)    Score    Peptide    Result File   
		    211    702.47    1402.92    1402.68    58    EVLPNVENNYGR    41A_4   
   
      Matching Genes:  
               gi|115252283|emb|CAJ70124.1|  (dihydrodipicolinate reductase [Clostridium difficile 630]) 
           
  Protein Group 179   
      Expression Quality:  
         Score      Num Spectra      Num Peptides      High-Qual Peptides      % Coverage       57    1    1    1    11   
   
      Peptides:   
        Query    Observed    Mr(expt)    Mr(calc)    Score    Peptide    Result File   
		    448    785.61    1569.21    1568.88    57    VKLENIIQNEELK    41_2   
   
      Matching Genes:  
               gi|115251313|emb|CAJ69144.1|  (conserved hypothetical protein [Clostridium difficile 630]) 
           
  Protein Group 180   
      Expression Quality:  
         Score      Num Spectra      Num Peptides      High-Qual Peptides      % Coverage       56    1    1    1    5   
   
      Peptides:   
        Query    Observed    Mr(expt)    Mr(calc)    Score    Peptide    Result File   
		    369    704.96    1407.91    1407.68    56    MVNLNDAYEIAR    41_4   
   
      Matching Genes:  
               gi|115252284|emb|CAJ70125.1|  (2,3,4,5-tetrahydropyridine-2,6-dicarboxylate N-succinyltransferase [Clostridium difficile 630]) 
           
  Protein Group 181   
      Expression Quality:  
         Score      Num Spectra      Num Peptides      High-Qual Peptides      % Coverage       56    2    1    1    4   
   
      Peptides:   
        Query    Observed    Mr(expt)    Mr(calc)    Score    Peptide    Result File   
		    437    1092.33    2182.64    2182.05    56    ELDIDPIDNPDLDIEEISK    41_6   
   
      Matching Genes:  
               gi|115252362|emb|CAJ70203.1|  (trigger factor [Clostridium difficile 630]) 
           
  Protein Group 182   
      Expression Quality:  
         Score      Num Spectra      Num Peptides      High-Qual Peptides      % Coverage       55    1    1    1    4   
   
      Peptides:   
        Query    Observed    Mr(expt)    Mr(calc)    Score    Peptide    Result File   
		    485    868.62    1735.22    1734.89    55    VITGLATSDDDSSITIK    41_2   
   
      Matching Genes:  
               gi|115251108|emb|CAJ68939.1|  (aspartokinase [Clostridium difficile 630]) 
           
  Protein Group 183   
      Expression Quality:  
         Score      Num Spectra      Num Peptides      High-Qual Peptides      % Coverage       54    1    1    1    7   
   
      Peptides:   
        Query    Observed    Mr(expt)    Mr(calc)    Score    Peptide    Result File   
		    212    556.89    1111.76    1111.62    54    ILVPIDGTER    41_1   
   
      Matching Genes:  
               gi|115249829|emb|CAJ67646.1|  (putative universal stress protein [Clostridium difficile 630]) 
           
  Protein Group 184   
      Expression Quality:  
         Score      Num Spectra      Num Peptides      High-Qual Peptides      % Coverage       54    2    1    1    4   
   
      Peptides:   
        Query    Observed    Mr(expt)    Mr(calc)    Score    Peptide    Result File   
		    15    413.26    824.50    824.40    54    TGEYLSR    41_3   
   
      Matching Genes:  
               gi|115250279|emb|CAJ68101.1|  (elongation factor P [Clostridium difficile 630]) 
           
  Protein Group 185   
      Expression Quality:  
         Score      Num Spectra      Num Peptides      High-Qual Peptides      % Coverage       54    2    1    1    5   
   
      Peptides:   
        Query    Observed    Mr(expt)    Mr(calc)    Score    Peptide    Result File   
		    390    1103.87    2205.73    2205.04    54    YSFSTIMEDKPGNFAELTR    41_5   
   
      Matching Genes:  
               gi|115251567|emb|CAJ69400.1|  (threonine dehydratase catabolic [Clostridium difficile 630]) 
           
  Protein Group 186   
      Expression Quality:  
         Score      Num Spectra      Num Peptides      High-Qual Peptides      % Coverage       53    1    1    1    6   
   
      Peptides:   
        Query    Observed    Mr(expt)    Mr(calc)    Score    Peptide    Result File   
		    54    497.87    993.73    993.55    53    YPFLLVDK    41A_8   
   
      Matching Genes:  
               gi|115249137|emb|CAJ66948.1|  ((3R)-hydroxymyristoyl-[acyl carrier protein] dehydratase [Clostridium difficile 630]) 
           
  Protein Group 187   
      Expression Quality:  
         Score      Num Spectra      Num Peptides      High-Qual Peptides      % Coverage       53    2    2    0    2   
   
      Peptides:   
        Query    Observed    Mr(expt)    Mr(calc)    Score    Peptide    Result File   
		    46    428.33    854.65    854.50    27    LDINRPK    41_8   
		    176    614.43    1226.85    1226.69    26    QVDIVINTPTK    41_8   
   
      Matching Genes:  
               gi|115252652|emb|CAJ70495.1|  (carbamoyl-phosphate synthase,pyrimidine-specific, large chain [Clostridium difficile 630]) 
              Other Genes Matching Peptide Subset:  
               gi|115252654|emb|CAJ70497.1|  (carbamoyl-phosphate synthase,pyrimidine-specific, large chain [Clostridium difficile 630]) 
           
  Protein Group 188   
      Expression Quality:  
         Score      Num Spectra      Num Peptides      High-Qual Peptides      % Coverage       52    1    1    1    7   
   
      Peptides:   
        Query    Observed    Mr(expt)    Mr(calc)    Score    Peptide    Result File   
		    208    731.06    1460.11    1459.74    52    FNALGIGQNEELR    41A_3   
   
      Matching Genes:  
               gi|115250846|emb|CAJ68670.1|  (tellurium resistance protein [Clostridium difficile 630]) 
           
  Protein Group 189   
      Expression Quality:  
         Score      Num Spectra      Num Peptides      High-Qual Peptides      % Coverage       51    2    1    1    6   
   
      Peptides:   
        Query    Observed    Mr(expt)    Mr(calc)    Score    Peptide    Result File   
		    502    908.16    1814.30    1813.98    51    ADILLAPDIEGGNILYK    41_4   
   
      Matching Genes:  
               gi|115249121|emb|CAJ66932.1|  (phosphate butyryltransferase [Clostridium difficile 630]) 
           
  Protein Group 190   
      Expression Quality:  
         Score      Num Spectra      Num Peptides      High-Qual Peptides      % Coverage       50    1    1    1    4   
   
      Peptides:   
        Query    Observed    Mr(expt)    Mr(calc)    Score    Peptide    Result File   
		    165    589.63    1177.25    1176.58    50    VATIEYDPNR    biorep41Cdiff_4   
   
      Matching Genes:  
               gi|115249080|emb|CAJ66891.1|  (50S ribosomal protein L2 [Clostridium difficile 630]) 
           
  Protein Group 191   
      Expression Quality:  
         Score      Num Spectra      Num Peptides      High-Qual Peptides      % Coverage       50    1    1    1    5   
   
      Peptides:   
        Query    Observed    Mr(expt)    Mr(calc)    Score    Peptide    Result File   
		    325    667.93    1333.84    1333.69    50    NLGLESEFLQGK    41_4   
   
      Matching Genes:  
               gi|115249688|emb|CAJ67505.1|  (conserved hypothetical protein [Clostridium difficile 630]) 
           
  Protein Group 192   
      Expression Quality:  
         Score      Num Spectra      Num Peptides      High-Qual Peptides      % Coverage       50    1    1    1    11   
   
      Peptides:   
        Query    Observed    Mr(expt)    Mr(calc)    Score    Peptide    Result File   
		    150    657.97    1313.94    1313.74    50    ITLPETAIDTLK    41A_1   
   
      Matching Genes:  
               gi|115250989|emb|CAJ68818.1|  (conserved hypothetical protein [Clostridium difficile 630]) 
           
  Protein Group 193   
      Expression Quality:  
         Score      Num Spectra      Num Peptides      High-Qual Peptides      % Coverage       50    1    1    1    2   
   
      Peptides:   
        Query    Observed    Mr(expt)    Mr(calc)    Score    Peptide    Result File   
		    171    623.41    1244.80    1244.68    50    IVEIINEYPR    41_6   
   
      Matching Genes:  
               gi|115251548|emb|CAJ69381.1|  (L-seryl-tRNA(Sec) selenium transferase (selenocysteinyl-tRNA(Sec) synthase) [Clostridium difficile 630]) 
           
  Protein Group 194   
      Expression Quality:  
         Score      Num Spectra      Num Peptides      High-Qual Peptides      % Coverage       49    2    1    1    5   
   
      Peptides:   
        Query    Observed    Mr(expt)    Mr(calc)    Score    Peptide    Result File   
		    152    638.41    1274.81    1274.60    49    NQDVSDEDILK    41A_3   
   
      Matching Genes:  
               gi|115251628|emb|CAJ69461.1|  (putative nitroreductase [Clostridium difficile 630]) 
           
  Protein Group 195   
      Expression Quality:  
         Score      Num Spectra      Num Peptides      High-Qual Peptides      % Coverage       49    2    1    1    7   
   
      Peptides:   
        Query    Observed    Mr(expt)    Mr(calc)    Score    Peptide    Result File   
		    258    822.66    1643.31    1642.86    49    GFIYTLTESKPYPK    41A_6   
   
      Matching Genes:  
               gi|115252732|emb|CAJ70576.1|  (conserved hypothetical protein [Clostridium difficile 630]) 
           
  Protein Group 196   
      Expression Quality:  
         Score      Num Spectra      Num Peptides      High-Qual Peptides      % Coverage       48    2    1    1    2   
   
      Peptides:   
        Query    Observed    Mr(expt)    Mr(calc)    Score    Peptide    Result File   
		    159    637.43    1272.85    1272.63    48    LPEPQFEGQTK    41_7   
   
      Matching Genes:  
               gi|115249008|emb|CAJ66819.1|  (DNA gyrase subunit B [Clostridium difficile 630]) 
           
  Protein Group 197   
      Expression Quality:  
         Score      Num Spectra      Num Peptides      High-Qual Peptides      % Coverage       46    1    1    1    6   
   
      Peptides:   
        Query    Observed    Mr(expt)    Mr(calc)    Score    Peptide    Result File   
		    320    904.15    1806.29    1805.85    46    AGATIGGANSEQMEEITK    41A_4   
   
      Matching Genes:  
               gi|115250238|emb|CAJ68059.1|  (geranyltranstransferase [Clostridium difficile 630]) 
           
  Protein Group 198   
      Expression Quality:  
         Score      Num Spectra      Num Peptides      High-Qual Peptides      % Coverage       45    1    1    1    3   
   
      Peptides:   
        Query    Observed    Mr(expt)    Mr(calc)    Score    Peptide    Result File   
		    54    533.88    1065.75    1065.59    45    ASVVHALNQK    41A_5   
   
      Matching Genes:  
               gi|115249122|emb|CAJ66933.1|  (butyrate kinase [Clostridium difficile 630]) 
           
  Protein Group 199   
      Expression Quality:  
         Score      Num Spectra      Num Peptides      High-Qual Peptides      % Coverage       45    1    1    1    8   
   
      Peptides:   
        Query    Observed    Mr(expt)    Mr(calc)    Score    Peptide    Result File   
		    269    849.23    1696.45    1696.03    45    VALVGGGIGVAPLYLVAK    41A_3   
   
      Matching Genes:  
               gi|115249195|emb|CAJ67007.1|  (dihydroorotate dehydrogenase electron transfer subunit [Clostridium difficile 630]) 
           
  Protein Group 200   
      Expression Quality:  
         Score      Num Spectra      Num Peptides      High-Qual Peptides      % Coverage       45    1    1    1    13   
   
      Peptides:   
        Query    Observed    Mr(expt)    Mr(calc)    Score    Peptide    Result File   
		    497    881.46    2641.35    2641.24    45    LPGEVESYEYGLEYGTDTLEIHK    41A_2   
   
      Matching Genes:  
               gi|115251797|emb|CAJ69632.1|  (adenine phosphoribosyltransferase [Clostridium difficile 630]) 
           
  Protein Group 201   
      Expression Quality:  
         Score      Num Spectra      Num Peptides      High-Qual Peptides      % Coverage       45    1    1    1    10   
   
      Peptides:   
        Query    Observed    Mr(expt)    Mr(calc)    Score    Peptide    Result File   
		    341    548.69    1643.06    1642.79    45    GKVEEGENNQEAALR    41A_2   
   
      Matching Genes:  
               gi|115252458|emb|CAJ70301.1|  (putative DNA repair protein (nucleotide pyrophosphatase) [Clostridium difficile 630]) 
           
  Protein Group 202   
      Expression Quality:  
         Score      Num Spectra      Num Peptides      High-Qual Peptides      % Coverage       43    1    1    1    7   
   
      Peptides:   
        Query    Observed    Mr(expt)    Mr(calc)    Score    Peptide    Result File   
		    311    645.95    1289.88    1289.64    43    EALNICGTISGR    41_2   
   
      Matching Genes:  
               gi|115251448|emb|CAJ69281.1|  (conserved hypothetical protein [Clostridium difficile 630]) 
           
  Protein Group 203   
      Expression Quality:  
         Score      Num Spectra      Num Peptides      High-Qual Peptides      % Coverage       42    1    1    1    13   
   
      Peptides:   
        Query    Observed    Mr(expt)    Mr(calc)    Score    Peptide    Result File   
		    601    834.03    1666.04    1665.77    42    MLSEMAIQDPEGFAK    biorep41Cdiff_2   
   
      Matching Genes:  
               gi|115249703|emb|CAJ67520.1|  (50S ribosomal protein L20 [Clostridium difficile 630]) 
             
