# Supplementary material for: Correction: Semiquantitative Analysis of Clinical Heat Stress in Clostridium difficile Strain 630 Using a GeLC/MS Workflow with emPAI Quantitation
Source: PLoS One. 2014 May 7;9(5):e97413. doi: 10.1371/journal.pone.0097413 (PMC4013136; doi:10.1371/journal.pone.0097413)
Supplement: File S2 — Republished corrected article. (PDF) [file pone.0097413.s002.pdf]

# Semiquantitative Analysis of Clinical Heat Stress in *Clostridium difficile* Strain 630 Using a GeLC/MS Workflow with emPAI Quantitation

**Nigel G. Ternan[1]*, Shailesh Jain[1], Robert L. J. Graham[2], Geoff McMullan[1]**

1 Northern Ireland Centre for Food and Health (NICHE), School of Biomedical Sciences, University of Ulster, Coleraine, Co. Londonderry, Northern Ireland, United Kingdom, 2 School of Medicine, University of Manchester, Manchester, Greater Manchester, United Kingdom

## Abstract

*Clostridium difficile* is considered to be the most frequent cause of infectious bacterial diarrhoea in hospitals worldwide yet its adaptive ability remains relatively uncharacterised. Here, we used GeLC/MS and the exponentially modified protein abundance index (emPAI) calculation to determine proteomic changes in response to a clinically relevant heat stress. Reproducibility between both biological and technical replicates was good, and a 37°C proteome of 224 proteins was complemented by a 41°C proteome of 202 proteins at a 1% false discovery rate. Overall, 236 *C. difficile* proteins were identified and functionally categorised, of which 178 were available for comparative purposes. A total of 65 proteins (37%) were modulated by 1.5-fold or more at 41°C compared to 37°C and we noted changes in the majority of proteins associated with amino acid metabolism, including upregulation of the reductive branch of the leucine fermentation pathway. Motility was reduced at 41°C as evidenced by a 2.7 fold decrease in the flagellar filament protein, FliC, and a global increase in proteins associated with detoxification and adaptation to atypical conditions was observed, concomitant with decreases in proteins mediating transcriptional elongation and the initiation of protein synthesis. Trigger factor was down regulated by almost 5-fold. We propose that under heat stress, titration of the GroESL and dnaJK/grpE chaperones by misfolded proteins will, in the absence of trigger factor, prevent nascent chains from emerging efficiently from the ribosome causing translational stalling and also an increase in secretion. The current work has thus allowed development of a heat stress model for the key cellular processes of protein folding and export.

**Citation:** Ternan NG, Jain S, Graham RLJ, McMullan G (2014) Semiquantitative Analysis of Clinical Heat Stress in *Clostridium difficile* Strain 630 Using a GeLC/MS Workflow with emPAI Quantitation. PLoS ONE 9(2): e88960. doi:10.1371/journal.pone.0088960

**Editor:** Gayatri Vedantam, University of Arizona, United States of America

**Received** August 5, 2013; **Accepted** January 16, 2014; **Published** February 24, 2014

**Copyright:** © 2014 Ternan et al. This is an open-access article distributed under the terms of the Creative Commons Attribution License, which permits unrestricted use, distribution, and reproduction in any medium, provided the original author and source are credited.

**Funding:** SJ was supported by a Vice Chancellor's Research Scholarship award (2007–2010) from the University of Ulster. RLJG was supported by the Northern Ireland Centre of Excellence in Functional Genomics, with funding from the European Union (EU) Programme for Peace and Reconciliation, under the Technology Support for the Knowledge-Based Economy. GM was supported by an Innovation Leaders award (2009) from the HSC Research and Development office (http://www.publichealth.hscni.net/directorate-public-health/hsc-research-and-development. NGT was supported by a Society for General Microbiology vacation studentship award for Peter McKee (VS07/49, 2007). The funders had no role in study design, data collection and analysis, decision to publish, or preparation of the manuscript.

**Competing Interests:** The authors have declared that no competing interests exist.

* E-mail: ng.ternan@ulster.ac.uk

## Introduction

*Clostridium difficile*, a Gram positive spore forming anaerobic bacterium, infects the human colonic epithelia causing diarrhoeal infections with symptoms including mild, self limiting diarrhoea with associated abdominal pain, cramping, and low grade fever (up to 40.6°C). Untreated, however, *C. difficile* infection (CDI) can lead to potentially life-threatening fulminant pseudomembranous colitis [1]. The factors underlying CDI – including extended hospitalisation and the widespread administration of broad spectrum antibiotics – and the organism's pathogenesis are well understood [2,3] and *C. difficile* is said to be most frequent cause of infectious bacterial diarrhoea in hospitals worldwide [4]. In addition to gastrointestinal disease, complications including build up of fluid in the peritoneal cavity and between the pleural layers of the lungs (ascites & pleural effusion, respectively), hepatic abscesses and renal failures have been reported [5] and worldwide, the cost of CDI is increasing annually [6,7].

The pathophysiological effect on host tissues of the primary virulence factors, the large clostridial glucosylating toxins, A and B, is well established [8,9,10] and the epidemiology of the disease – including the increased morbidity, cost and mortality associated with hypervirulent ribotype 027 and ribotype 078 strains – has been the subject of careful study for over 20 years [11,12,13,14]. However, *C. difficile* virulence is a multifactorial phenomeon and is still poorly understood [15,16]. For example, the 'hypervirulence' of ribotype 027 strains has previously been attributed in part to increased sporulation, yet recent work has shown that ribotype 027 strains do not, in fact, sporulate more readily or at higher rates than other, non ribotype 027 strains [17].

Thus it is timely for researchers to adopt global systems biology-driven approaches to understanding this pathogen. Public availability of well over 30 *C. difficile* genome sequences [4,18,19,20] has afforded researchers the opportunity to better understand the evolution and lineages of these organisms, yet generation of post-genomic comparative datasets has lagged somewhat. While the ClosTron gene disruption system [21] has allowed precise analysis of the functions of a considerable number of individual genes/proteins involved in, for example, sporulation

[22], motility [23,24] secretion [25], regulation of virulence factor expression [26] and the release of toxins A and B [27], comparatively little is known about the adaptive response of *C. difficile*. Emerson et al. [28] began to address this by analysing the transcriptional response of *C. difficile* strain 630 to seven different antimicrobial and environmental stresses and the work of Scaria et al., [29] built on both cell culture and *in vivo* porcine CDI models has expanded upon this. The recent work of Janoir et al. furthermore described for the first time the adaptive transcriptomic changes throughout the colonisation phase of infection in a mouse model of CDI [30]. While we have a relative abundance of transcriptome data for *C. difficile*, it is well established, however, that the correlation between transcripts and actual functional protein levels is not always good, with factors including transcription efficiency [31], protein stability/stabilisation, or the presence of small regulatory RNAs [32], amongst others, contributing to discrepancies between measurements.

Shotgun Proteomics analyses, using nanoflow liquid chromatography coupled with tandem mass spectrometry (MS/MS) instrumentation offer life scientists a powerful direct assessment of, and insight into, the functional components of cellular machinery [33,34,35]. Due perhaps to the complexity and cost of the instrumentation and the operator skill required for robust proteomics analyses, however, only four shotgun proteomics datasets exist for *C. difficile* [36,37,38,39]. Whilst these provide useful global snapshots of metabolic function, measurement of the adaptive response of the *C. difficile* proteome is much less advanced. Two recent reports describing iTRAQ-driven analysis of the organism's response to clinically relevant stress [40,41] exist, both complemented by analysis of the transcriptional programme under the same conditions [29,42]. However, proteomes generated by isobaric labelling necessarily comprise only those proteins found in both experimental conditions, and yield few insights into those unique to each experimental condition [43]. Semiquantitative approaches such as calculation of the exponentially modified protein abundance index (emPAI) [44] and other means of protein quantitation, including spectral counting [45], are widely used in comparative proteomics [46,47], and our group has previously used emPAI to allow analysis of functional and adaptive proteomic profiles in two distinct phases of bacterial growth in the nosocomial pathogen *Ochrobactrum anthropi* [48]. We now present a semi-quantitative analysis of clinically relevant heat stress in *C. difficile* strain 630 in which we both quantitate proteomic changes and investigate proteins unique to the 37°C and 41°C proteomes.

## Materials and Methods

### Reagents

All chemicals and reagents, of the highest purity available, were purchased from Sigma-Aldrich (Poole, UK), unless otherwise stated. All 1D-PAGE reagents were purchased from Invitrogen (Renfrewshire, UK); Lysing Matrix E tubes were from MP Biomedicals (Cambridge, UK); MS-grade water and acetonitrile (ACN) were purchased from Romil (Cambridge, UK) and trypsin was from Promega (Madison, WI, USA).

### Cell culture and growth conditions

*Clostridium difficile* strain 630 was a kind gift from Dr Peter Mullany of the Eastman Dental Institute, London and was routinely maintained on BHI agar (Oxoid) at 37°C in a MACS MG500 Anaerobic workstation fitted with an airlock (Don Whitley Scientific, UK). The workstation was operated on a conventional anaerobic gas mixture containing 80% $N_2$, 10% $H_2$ and 10% $CO_2$ and resazurin (1 mg $L^{-1}$) was used in all growth media as a redox indicator. Routine growth of the organism involved the inoculation of autoclaved, pre-reduced BHI broth (100 ml) with a single actively growing colony from BHI agar. Cultures were grown overnight (~16 h), and used as inocula at 5% (v/v) for growth in 1 L cultures, which were monitored by the increase in culture attenuance at 650 nm ($D_{650}$) versus uninoculated BHI broth. Biological duplicate cultures were set up, comprising 2×1 L cultures grown at 37°C for the entirety of the experiment, and 2×1 L cultures grown at 37°C until the early exponential phase ($D_{650}$ = 0.3), following which heat stress was induced by transferring them to a pre-heated 41°C circulating water-bath: incubation continued for a further 3 h to $D_{650}$ = 1.1, at which point cells were harvested from all four cultures. Temperature equilibration from 37°C to 41°C occurred in approximately 4 min, and maintenance of anaerobiosis was confirmed by the observation that the resazurin remained colourless at all times. Plating and subculture experiments showed that the cells remained viable at 41°C. Attenuance was measured in the 41°C culture bottles by briefly transferring them back to the anaerobic cabinet for removal of an aliquot, followed by a return to the 41°C water bath (total time for sample retrieval, ~2 min).

### Cell harvest and lysis

Cultures were harvested at late-log phase ($D_{650}$ = 1.1) of anaerobic growth by centrifugation in a sealed tube at 10,000× g for 15 min at 4°C in the JA10 rotor of a Beckman J2-HS centrifuge (Beckman Instruments, Fullerton, CA, USA). Spent broth was discarded inside the anaerobic cabinet and the cells resuspended and washed in ice-cold 10 mM phosphate-buffered saline (pH 7.8) by centrifugation as before. PBS was decanted in the cabinet and pellets resuspended (1 g of cells/2 ml of buffer) in fresh PBS. For cell breakage, 1 mL aliquots of cell suspension were transferred to a Lysing Matrix E tube and homogenized using the FastPrep FP120 Instrument (BIO 101 Inc., CA, USA) for four 30 s disruptions at a speed setting of 5.5. The cell homogenate was chilled on ice for 2 min between disruptions. The homogenate was centrifuged at 25,000× *g* for 30 min at 4°C in the F2402H rotor of a Beckman Allegra 64R centrifuge to remove unbroken cells and debris. The resultant supernatant was then centrifuged at 150,000× *g* for 2 h at 4°C in the 70.1 Ti rotor of a Beckman L8-M centrifuge in order to sediment the insoluble, membrane-associated fraction [34,38]. Subsequently, the ultracentrifuged supernatant containing the soluble, cytosolic sub-proteome was decanted and stored frozen in multiple 1 ml aliquots at −70°C until required. Total protein in the cell extracts was determined using the method of Bradford [49].

### One-dimensional polyacrylamide gel electrophoresis (1D-PAGE)

The soluble protein fraction was made up in 1× Tris-Glycine SDS sample loading buffer at a concentration of 5 mg/mL and boiled in a water-bath for 5 min. Subsequently, 100 µg (20 µL) was loaded onto a 1.5 mm thick NuPage 4–12% Bis-Tris gel. SeeBlue Plus2 Pre-Stained Standard was used as a protein molecular mass marker. Electrophoresis was carried out using 1× MES-SDS running buffer in an XCell II Mini Gel System at 200 V, 120 mA, 25 W per gel for about 40 min. Proteins were visualized using SimplyBlue SafeStain as per the manufacturer's instructions. The entire lane was excised from the gel using a sterile scalpel and cut into eight fractions (1 $mm^3$ cubes) based on molecular mass as previously described [38].

## In-gel tryptic digestion

Excised gel pieces, in 0.5 mL siliconised tubes, were washed overnight in 50% (v/v) methanol/5% (v/v) glacial acetic acid. The gel pieces were then dehydrated by incubation for 10 min in 100% ACN at room temperature, followed by drying under vacuum in a SpeedVac. The fractions were then reduced using freshly prepared 10 mM dithiothreitol (DTT) solution for 30 min, followed by alkylation using freshly prepared 100 mM iodoacetamide solution for a further 30 min. The fractions were once again dehydrated using 100% ACN for 10 min and subsequently rehydrated for 10 min in 100 mM ammonium bicarbonate ($NH_4HCO_3$). The gel pieces were completely dried under vacuum and 0.2 µg of trypsin (Promega, Madison, USA) dissolved in 20 µL of 50 mM $NH_4HCO_3$ (pH 7.8) was added to each sample, which was then incubated overnight at 37°C. Subsequently, the supernatant was recovered into fresh siliconised tubes and a second peptide extraction from the gel pieces was carried out using 5% (v/v) formic acid and 50% (v/v) ACN for 10 min. Peptide-containing liquid fractions were pooled together, dried under vacuum and resuspended in 20 µL of 0.1% formic acid in 2% ACN prior to storage at −70°C until required.

## Liquid chromatography-mass spectrometry (LC-MS) analysis

LC-MS was carried out as previously described [38,48]. Briefly, MS was performed using a 3200 Q-TRAP Hybrid ESI Quadrupole linear IT mass spectrometer, ESI-Q-q-Qlinear IT-MS/MS (Applied Biosystems/MDS SCIEX, Toronto, Canada) with a nanospray interface, coupled with an online Ultimate 3000 nanoflow LC system (Dionex/LC Packings, Amsterdam, The Netherlands). A m-Precolumn Cartridge (300 µm×5 µm, 5-µm particle size) was placed prior to the $C_{18}$ capillary column (75 µm×150 mm, 3 µm particle size) to allow desalting and filtering. Both columns contained the reversed phase material PepMAP 100 ($C_{18}$ silica-based) with a 100-Å pore size (Dionex/LC Packings). The following elution buffers were used in the gradient: Buffer A (0.1% formic acid in 2% ACN) and Buffer B (0.1% formic acid in 80% ACN). The nanoLC gradient used was 60 min in length: 0–55% B in 45 min, 10 min at 90% B followed by 5 min at 100% A. The flow rate of the gradient was 300 nL/min and the detector mass range was set at 400–1400 *m/z*. MS data acquisition was performed in positive ion mode. During MS acquisition, peptides with 2+ and 3+ charge states were selected for fragmentation.

## Database searching, protein identification and PROVALT analysis

Protein identification was carried out using an internal MASCOT server (version 1.9; Matrix Science, London, UK) searching against a combined *C. difficile* genomic DNA and plasmid database [38] and containing 3573 sequences in total. Peptide tolerance was set at ±1.2 Da with MS/MS tolerance set at ±0.6 Da and the search set to allow for 1 missed cleavage. To expedite the curation of the identified protein list from MASCOT, PROVALT analysis was carried out as previously described by Graham *et al.* [48]. The MASCOT output files were re-analysed against the extracted *C. difficile* database using PROVALT [45], which takes multiple MASCOT results and identifies matching peptides. Redundant peptides are removed and related peptides grouped together, associated with their predicted matching protein. PROVALT also uses peptide matches from a random database (in this case the *C. difficile* database was randomised) to calculate false-discovery rates (FDR) for protein identifications as previously described by Weatherley *et al.* [45]. For identification purposes, the minimum peptide length was set at 6 amino acids, the minimum peptide MOWSE score was set at 25 and the minimum high quality peptide MOWSE score was set at 40. The FDR calculations employed by PROVALT provide a good balance between the number of correct and incorrect protein assignments. As in previous work [38,48], the FDR was set at 1%, thus 99% of proteins identified should be correct. The proteins identified by standalone PROVALT analysis were subsequently quantified by calculation of the exponentially modified protein abundance index (emPAI) and molar % values for identified proteins [44].

## Application of emPAI

Proteogest software (http://www.utoronto.ca/emililab/proteogestnosummary.htm) was used to generate lists of *in silico* digested peptides ($N_{obsbl}$) [50] to facilitate calculation of PAI and emPAI values.

The Protein Abundance Index (PAI) [44] is defined as: $PAI = N_{obsd}/N_{obsbl}$, where $N_{obsd}$ and $N_{obsbl}$ are the number of MS-observed peptides per protein and the number of theoretically observable peptides per protein respectively. Based upon PAI, emPAI is defined as: $emPAI = (10^{PAI})$-1. The protein content in molar fraction percentage (M%) can then be calculated using the following formula: Protein content $(M\%) = emPAI/\Sigma(emPAI)$, where $\Sigma$(emPAI) is the summation of emPAI values for all the identified proteins. Fold change ratios for identified proteins were calculated by dividing the calculated molar percentage value for an individual protein at 41°C with the cognate 37°C value.

## Bioinformatics

PSORTb version 2.0.4 [51], http://www.psort.org/psortb/index.html was used for the prediction of bacterial protein subcellular localisation. SignalP version 3.0 [52], http://www.cbs.dtu.dk/services/SignalP/ was used to predict the presence and location of signal peptide cleavage sites in amino acid sequences for classically secreted proteins. SecretomeP version 2.0 [53], http://www.cbs.dtu.dk/services/SecretomeP/ was used for the prediction of non-classical protein secretion (referring to protein secretion that is not triggered by signal peptides).

# Results and Discussion

## Comprehensive analysis of the *C. difficile*proteome using GeLC/MS: Calculation of protein abundances using emPAI

The main aim of the current work was to conduct a comparative proteomic analysis of *C. difficile* 630 grown under two different physiological conditions (37°C and 41°C). We set out to identify proteins whose abundance changed significantly under this clinically relevant heat stress and to determine which, if any, were apparently unique to each temperature. From a technical standpoint, we wished in addition to establish some parameters regarding overall reproducibility of our GeLC/MS method by applying the emPAI workflow, which we have previously successfully applied to the comprehensive analysis of the soluble subproteome of *Ochrobactrum anthropi* at two distinct phases of growth [48]. In the current work, we initially utilised the emPAI protocol to estimate the abundance of the proteins identified in technical replicates of our 37°C samples. PROVALT [45] output data was exported to Excel spreadsheets and the emPAI value was then calculated and used to estimate the protein content within the sample mixture in molar fraction percentages [44,54].

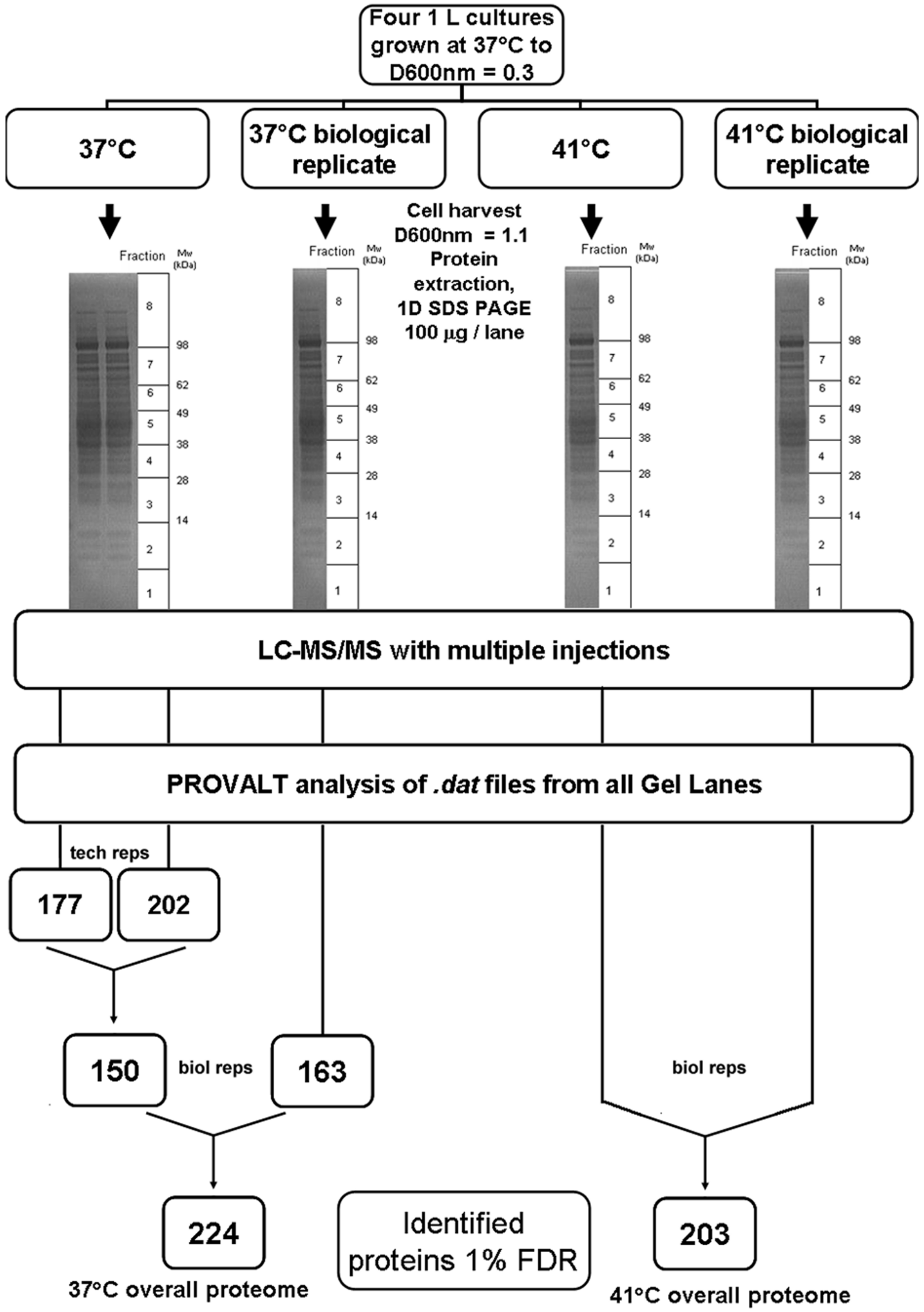


**Figure 1. GeLC/MS-PROVALT workflow used to generate the 37°C and 41°C proteomes in *Clostridium difficile* strain 630.**
doi:10.1371/journal.pone.0088960.g001

## Reproducibility of GeLC/MS: analysis of technical and biological variability

An overview of our experimental set up is depicted in Figure 1. Four *C. difficile* strain 630 cultures (1 L) were set up and at D650 nm = 0.3, two of these cultures were transferred to 41°C. At D650 nm = 1.1, cells were harvested from both 37°C and 41°C cultures and proteins extracted for GeLC/MS as per materials and methods. Due to the chance nature of automated selection of peptides for MS/MS analysis and the resultant requirement for multiple injections of a single sample to maximise peptide identification [33,34,35,38,48], we initially wished determine the level of reproducibility between technical replicates for a single sample. To do so, we firstly compared inter-lane variability, as proteins identified, for the same protein sample. Thus, for one of the 37°C cultures, the same sample of cell extract protein was electrophoresed on two separate lanes of a gel. Each gel lane was then subject to fractionation, tryptic digestion and LC/MS analysis as per materials and methods and the peptide samples derived from each individual digested gel fraction were injected once (Figure 1). The proteins identified by single injection LC/MS for lane1 were then compared with those identified in lane 2 (the 'pseudoereplicate'). Thus, each lane was analysed over 8 injections and generation of a final list for each complete lane using

PROVALT [45] identified 177 proteins in lane 1, versus 202 proteins in the pseudoreplicate lane 2 (Figure 1 and Files S1, S2). A total of 150 proteins were common to the datasets from both lanes and upon calculation of molar % for all proteins identified in each technical replicate, a Pearson correlation of 0.864 was obtained, indicating very good reproducibility between the technical replicates. To assess variation between biological duplicates, we analysed the biological duplicate 37°C culture via single gel lane GeLC-MS with multiple injections (n = 3) and identified a total of 163 proteins (File S3). Pearson analysis revealed a strong positive correlation between the biological replicates of 0.7 (n = 129) and overall our GeLC-MS analysis identified a proteome of 224 proteins for *C. difficile* grown at 37°C (File S4). Within this 37°C proteome, analysis revealed that the average MOWSE score was 284, with an average of 5 peptides per protein and 22% sequence coverage (Table S1). The largest protein identified was Toxin A (CD0663) at 308.25 kDa while the smallest was a "hypothetical protein" encoded by the CD630 plasmid, CDP09 at 5.8 kDa. This is the first proteomic identification of a protein from the *C. difficile* strain 630 plasmid, and BLASTP analysis revealed it to contain a Ribbon-helix-helix domain (pfam12651), which is likely to be DNA binding. The most acidic protein was ferredoxin (CD3605A) with a pI of 4.26, while the most basic was 50S ribosomal protein L20 (CD0687) at pI = 11.4.

Having established that reproducibility between both technical and biological replicates was good, we proceeded to identify the proteome of *C. difficile* subjected to heat stress at 41°C (Figure 1) via the same GeLC/MS workflow (duplicate cultures, single lane geLC/MS, n = 3 injections/gel slice), yielding an overall proteome of 203 proteins (File S5). Within the 41°C proteome, which exhibited an average MOWSE score of 245, 4.5 peptides per protein and 19% sequence coverage, the lowest mass protein identified was ferredoxin (CD3605A) at 6.43 kDa, which was also the most acidic protein identified. The largest protein was DNA directed RNA polymerase beta chain at 139.3 kDa while the most basic protein was again 50S ribosomal protein L20 (pI = 13.47) (Table S2). In both 37°C and 41°C proteomes, SignalP analysis [52] identified 10 proteins as having predicted signal peptides: the majority of these were cell surface proteins (e.g. CD2193, cwp24; CD2793, slpA) or substrate binding components of transporters (e.g. CD0873, CD2672) and we noted that 8/10 of these proteins were common to both 37 and 41°C proteomes. The total number of proteins identified from biological duplicate cultures of *C. difficile* strain 630 grown at either 37°C or 41°C is therefore commensurate with our previous work: GeLC-MS analysis of *Geobacillus thermoleovorans* T80 identified 157 proteins [34], and a similar analysis of *Oceanobacillus iheyensis* identified 153 proteins [55]. In both these investigations, as here, we also identified a large proportion of the total complement of ribosomal proteins, as well as molecular chaperones, elongation factors, central metabolic enzymes and other relatively abundant proteins. Reproducibility, between technical replicates and between biological duplicates, was good and thus we could proceed with confidence to analyse the proteins identified only at either 37°C or 41°C and to examine changes in protein abundance within the *C. difficile* strain 630 combined proteome.

## Proteins identified only at either 37°C or 41°C

Due to the complex nature of the peptide mixtures being analysed, the separation capabilities of LC/MS systems can be exceeded, with only the most abundant peptides in a scan being selected for MS/MS analysis – a limitation of data dependent acquisition [48]. Thus, as in our previous work, all samples were analysed three separate times [40] resulting in increased overall peptide identifications. With a multiple injection workflow, the resolution of the MS becomes the limiting factor in proteome penetration. Thus, a protein that was identified as unique to the 37°C *C. difficile* proteome was present in 37°C samples at abundance sufficiently high to allow its detection. However, we cannot say with certainty that the same protein is definitely not present in the 41°C samples: only that its abundance at 41°C may be below the limit of detection for the workflow used. It may be reasonable to hypothesise, however, that if a protein is detected at 37°C alone, its relative abundance is likely to be much lower at 41°C, and *vice versa*. With this in mind, we can begin to consider the biology implied by the proteins identified as 'unique' to each growth condition (Table S3) in the context of the entire identified proteome. These 'unique' proteomes from 37°C or 41°C are relatively small when considered by themselves: a total of 25 distinct proteins with an average MOWSE score of 63 and 9% sequence coverage were identified only within the 41°C samples.

The most abundant protein in the 41°C proteome by molar % was Ribosomal protein L31 (CD3486A), a component of the large 50S ribosomal subunit that contains four conserved cysteine residues that allow it to rapidly form intracellular disulfide bonds *in vitro*. Furthermore, the protein contains a CXXC motif, also commonly found in thiol-disulfide redox proteins such as thioredoxin. L31 is known to play a role in stress response in a variety of microorganisms, as do thioredoxins [48,56]. It has been proposed that ribosome stalling via oxidation of the CXXC motif of ribosomal protein L31 occurs as a result of disulfide stress in *S. coelicolor* [57] and this may also be possible in *C. difficile*. In the combined proteome, we noted that Coenzyme A disulfide reductase (CD1797, CoADR) was upregulated by 1.98-fold, perhaps in order to counteract this putative oxidation of L31. Ribosomal protein S6, which was upregulated 2.27 fold in the current work, is proposed to play a unique role in sensing temperature differences in order to control ribosome function [56,58] and we suggest evidence elsewhere in this work for ribosome stalling under heat stress.

Two cell surface proteins (cwp19, CD2767; cwp25, CD0844) were also found only in the 41°C proteome. The cell wall proteins of *C. difficile* all contain three copies of the pfam 04122 motif, a complex motif annotated as 'putative cell wall binding repeat 2' and have recently had their nomenclature standardised in the work of Fagan et al. [59]. The idea has been proposed that these pfam motifs mediate binding of the *cwp* proteins to the underlying cell wall [59,60] and also enhance adhesion to surfaces [61]. It is therefore possible that increases in GroEL abundance, combined with various cwp (for example, cwp19 was upregulated 1.56 fold in our microarray experiment) might enhance *C. difficile* adherence to surfaces under heat stress and adaptation. Interestingly however, the two cell wall proteins identified in the combined proteome were down regulated (cwp6, CD2784, and cwp24, CD2193). Certain cwp contain, in addition to three pfam04122 repeats, a second domain that specifies a known or putative function [59]. In the case of cwp6 and cwp24, the additional domains are a peptidoglycan amidohydrolase and a glucosaminidase, respectively: these domains could potentially allow remodelling of the peptidoglycan layer. This process could therefore be down regulated under heat stress, a hypothesis reinforced, to some extent, by the detection of cwp22 (CD2713) only at 37°C: the additional YkuD domain of cwp22 is a predicted transpeptidase that allows alternative peptidoglycan cross linking and impacts upon sensitivity to β-lactam antibiotics. Analysis of the cell wall proteins (2.4% of our total identified proteome), including slpA (CD2793) which was unchanged in all our analyses, again suggests

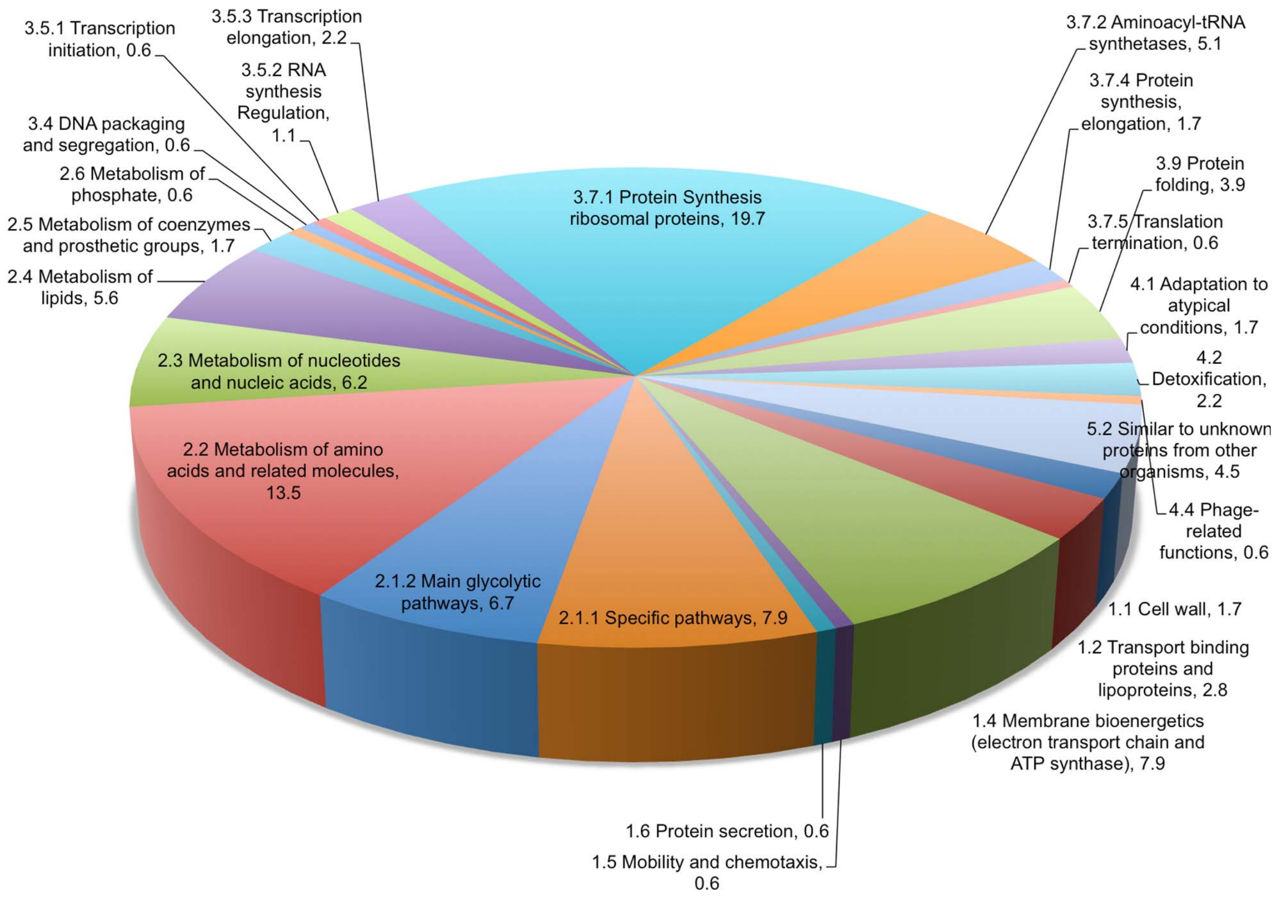


**Figure 2. Functional categorisation of identified proteins in the combined *Clostridium difficile* strain 630 GeLC/MS proteome.**
doi:10.1371/journal.pone.0088960.g002

increased cell adhesion, combined with decreased rearrangement of the cell wall constituents under heat stress. A number of proteins associated with response to intracellular stress were also found only in the 41°C proteome. CD3398, a putative DNA repair protein (nucleotide pyrophosphatase) belonging to the NUDIX superfamily, was also slightly upregulated by 1.34-fold in our microarray dataset. Since Nudix hydrolases hydrolyze X-linked nucleoside diphosphates and enable cellular housecleaning via this hydrolysis of aberrant deoxynucleoside triphosphates, they function to reduce incorporation of undesired bases into DNA. The substrates of these proteins are diverse and include 8-oxo-dGTP, a classical marker of DNA damage [62], although only a small number of protein/substrate combinations have been characterised to date [63,64].

CD0812 encodes a universal stress protein that promotes stress endurance under prolonged stress conditions such as those imposed by our experiment. Its precise function is unknown, however under stress conditions such as heat shock, nutrient starvation, the presence of oxidants, uncouplers, and DNA-damaging agents that may arrest cell growth, USPs are overproduced [65]. An additional stress responsive protein, CD1800 (annotated as a 'tellurium resistance protein') was found only in the 41°C proteome and is one of seven such proteins within the *C. difficile* genome containing a terD-like domain. Tellurite resistance proteins are found in many pathogenic bacteria, and although their precise role is as yet uncertain [66], they are known to respond to a variety of stresses. Within our microarray data, the raw data 41°C/37°C ratios for most of these tellurium resistance protein encoding genes suggested up-regulation, albeit with $p$ values $>0.05$. In addition, we identified phosphate butyryltransferase (CD0112) and butyrate kinase (CD0113) – proteins involved in short chain fatty acid metabolism, which may be biologically more important than carbohydrate metabolism at 41°C.

Within the 37°C proteome samples, a total of 46 proteins with an average MOWSE score of 76 and 10% coverage were unique to this temperature alone. Toxin A (CD0663) was detected only in the 37°C proteome and thus its abundance can be hypothesised to be lower in the 41°C proteome. This is corroborated by our other datasets that show down regulation of tcdA under heat stress, using both microarray (3.1 fold down) and q-RT-PCR techniques [42]. This is the first detection of peptides from any of the *C. difficile* toxins at 1% FDR using our workflow. It may be that the levels of toxin produced by *C. difficile* 630 under our growth conditions are relatively low, or that, at time of cell harvest, the majority of toxin protein molecules were present extracellularly. Nonetheless, it is known that the virulence of *C. diff*icile, and other pathogens, is set at a certain, optimum, temperature and other researchers have shown decreased abundance of tcdA at temperatures other than 37°C [67]. Our microarray data for *tcdB* indicated that the transcript was unchanged as the p value was $>0.05$, however the raw data 41°C/37°C expression ratio suggested down regulation at the higher temperature [42].

Evidence that the phosphotransferase system (PTS) for sugar uptake may be of less importance under heat stress was provided

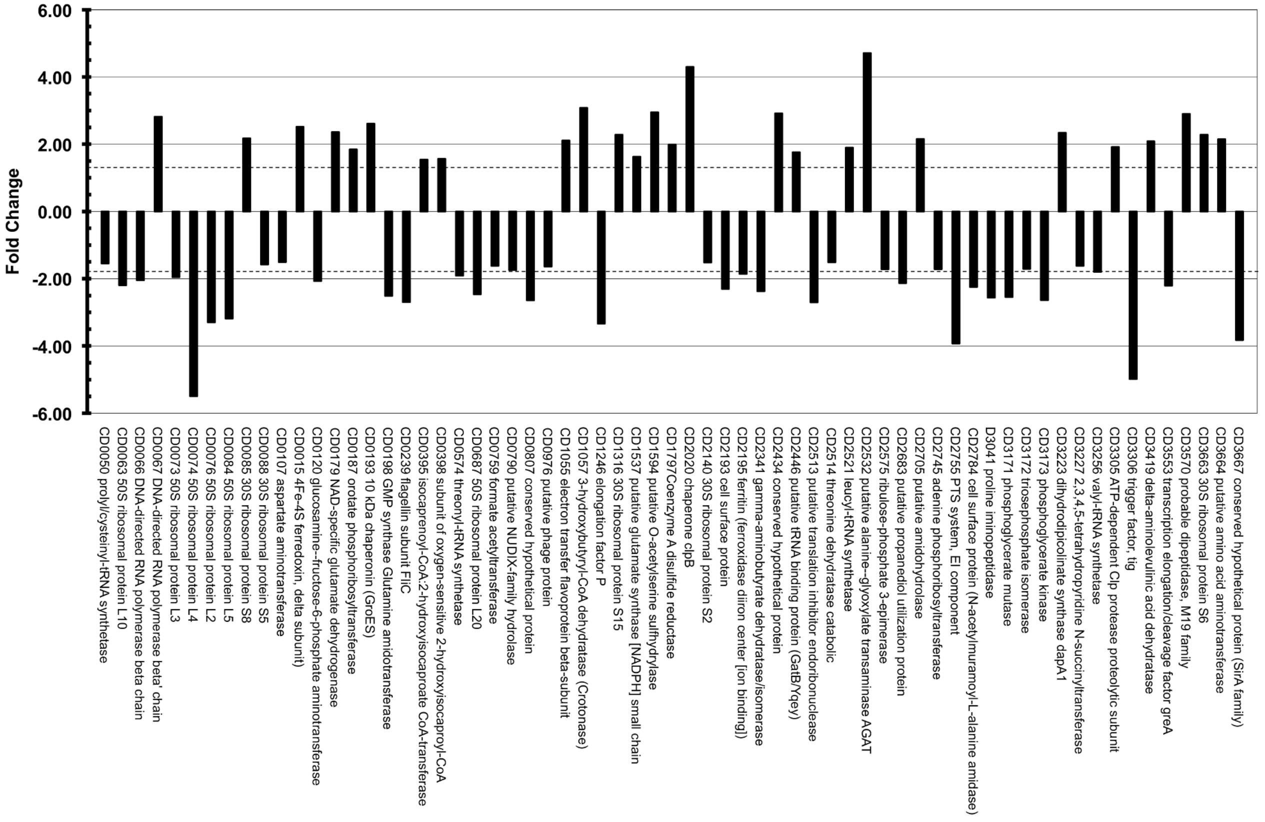


**Figure 3. Differential expression profile of 65 proteins common to both the 37°C and 41°C *Clostridium difficile* strain 630 GeLC/MS proteomes whose abundances changed by ≥1.5 fold (dotted lines).**
doi:10.1371/journal.pone.0088960.g003

by detection of CD3068 (Enz IIb component, manX) and CD3277 (enz IIc component, manY), both of which are involved in mannose transport, only at 37°C. In support of this, CD3068 was down regulated some 1.9-fold by microarray, as indeed was CD3069, the IId component at 2.2-fold down [42]. In the combined proteome, the EI component (CD2755), common to, and essential for all phosphotransferase systems in the cell, was down regulated by 3.9 fold, although transcripts were reduced by only 1.46 fold [42].

A putative thiol peroxidase (bacterioferritin comigratory protein, CD1822) was detected only at 37°C and thus was potentially less abundant in the 41°C proteome: its cognate transcript was down regulated by 2.8-fold at 41°C [42]. These thioredoxin-dependent thiol peroxidases (peroxiredoxins) are widely expressed in pathogenic bacteria, where they protect against oxidative stress that might be encountered during the infection process. The transcription antitermination factor, nusB (CD1201) regulates transcription of rRNA operons by modulating the efficiency of transcriptional antitermination (i.e. the progression of transcription) in complex with 30S S10 (CD0072, unchanged in this work). While S10, identified as a central hub in the antitermination process, cannot bind NusB and the 30S subunit at the same time it nonetheless represents an example of the functional diversity of ribosomal proteins, and indeed a number of ribosomal proteins also function as transcription factors [68]. NusB detection only at 37°C could imply greater quantities of free S10 available for 30S subunit binding at 41°C, perhaps concomitant with decreased rRNA synthesis.

## Quantitation of protein abundance changes in 37°C and 41°C proteomes using emPAI

Overall, a total of 178 proteins were available for comparison and quantitation in both 37°C and 41°C samples (Table S4), a slightly higher number than was available in our quantitative analysis of the emerging nosocomial pathogen *Ochrobactrum anthropi* (131 proteins) [35]. In *Ochrobactrum*, we noted significant changes in abundance for only 19 proteins between early and late log phases of growth, including a number of gene products under the control of the *oxyR* regulon which is induced in response to oxidative stress and whose protein products have been linked with pathogen survival in response to host immunity. In the *C. difficile* strain 630 combined proteome, the most abundant protein as calculated by molar fraction % at 37°C was phosphoglycerate mutase (CD3171) while at 41°C, ferredoxin (CD3605A) and ruberythrin (CD1524) were most abundant. The least abundant protein at 37°C was DNA directed RNA polymerase beta' subunit (CD0067), while at 41°C, cwp6 (CD2784) was least abundant. Within the combined proteome, the largest category of identified proteins were those involved in protein synthesis (ribosomal proteins) at ~20%, followed by those involved in metabolism of amino acids and related molecules (13.5%), those involved in specific pathways (7.9%) and those of the main glycolytic pathway (6.7%). The remaining proteins were distributed amongst the other functional categories (Figure 2).

In addition to emPAI and molar fraction percentages, the fold change in the expression level of proteins identified under both growth conditions was also calculated [48] by dividing the molar percentage value for an individual protein at 41°C with the

cognate value at 37°C. The log2 value of the 41/37 ratio was calculated and finally the absolute fold change calculated as $2^{\log 2\ \text{value}}$ [40]. We selected a cutoff for biological significance of 1.5 fold, in keeping with our previous *C. difficile* investigations [40,42]. Taking a ≥50% cut-off value for biological significance, 65 proteins were modulated, representing some 37% of the total proteome: 26 proteins were significantly up-regulated, whereas 39 proteins were significantly down-regulated in response to the 41°C heat-stress in the combined proteome (Figure 3). A number of the 50S ribosomal proteins were downregulated, as were a number of transcription and elongation factors including trigger factor (CD3306), elongation factor P (CD1246), and GreA (CD3553), as well as flagellin (CD0239) and several cell surface proteins (e.g. cwp24, CD2193; cwp6, CD2784). As expected, a number of proteins associated with chaperone and housekeeping functions were upregulated, including the chaperone clpB involved in reactivating aggregated proteins and protease clpP1 (CD2020 & CD3305, repectively), an electron transfer flavoprotein beta-subunit (CD0400), GroES (CD0193), ferredoxin (CD0115) and a number of aminoacid aminotransferases (Table S4). A number of components of the 30S ribosome, including the temperature sensor, S6 [56,58]] were upregulated, however whilst GroeS was upregulated, its co-chaperone GroEL (CD0194) was unchanged, as was the DnaK chaperone (CD2461).

## Central Metabolism

Protein abundances in the main glycolytic pathway and those involved in metabolism of lipids were largely unchanged, however we noted that abundance of over half of the proteins involved in amino acid metabolism and interconversion (14 of 24 proteins) changed under heat stress. The increased abundance of dihydrodipicolinate synthase 2 (CD3223), involved in both lysine biosynthesis and in production of dipicolinate in spores – recognised as one of the organism's main virulence factors – suggests that cells under heat stress are more reliant upon fermentation and metabolism of amino acids, possibly due to lifting of carbon catabolite repression [69], although there is limited evidence in our data for such an effect upon either carbohydrate utilisation or amino acid fermentation pathways. The catabolite control protein CcpA (CD1064) is a pleiotropic regulator that via binding to well defined $cre_{CD}$ sites upstream of some 18% of *C. difficile* genes, enables both positive and negative control of global transcription in response to carbohydrate availability [69]. Our microarray data suggest down regulation of CD1064 transcripts, implying that lifting of ccpA mediated transcriptional control could be important for survival and maintenance of metabolism under heat stress. CD3664, a putative aminoacid aminotransferase with a $cre_{CD}$ site upstream that is predicted to be unregulated by ccpA, was upregulated by 2.14 fold in the combined proteome, and by 1.45 fold by microarray [42], suggesting increased reliance on amino acid metabolism. An M19 family Zn-metallo-dipeptidase (CD3570) was upregulated by 2.9 fold, and while this protein could be involved in amino acid metabolism, its precise function is as yet unknown. Conserved domain analysis suggests it could play a role in detoxification – either of carbapenam or β-lactam antibiotics, or in metabolism of glutathione or its firmicutes surrogate, bacillithiol [70]. The HadA protein (CD0395), involved in leucine catabolism, with a $cre_{CD}$ site upstream and predicted to be negatively regulated by ccpA, was also upregulated in the current work. Leucine is an essential amino acid growth substrate of *C. difficile*: during fermentation, three moles of leucine are fermented to a mixture of fatty acids – two moles of leucine are reduced to isocaproate, whereas one mole is oxidised to isovalerate and $CO_2$ [71]. Previously, we identified seven of the eight proteins necessary for the reductive branch of the leucine fermentation pathway encoded by genes CD0394 – CD0401 [38] with the sole exception of the ATP-dependent activator protein, hadI. We subsequently identified hadI (CD0396) in our iTRAQ investigation, where, like hadA (CD0395), hadB (CD0397) and hadC (CD0298), its abundance did not change under heat stress [40]. In the current work, however, isocaprenoyl-CoA:2-hydroxyisocaproate CoA-transferase (CD0395, *hadA*), and a subunit of the oxygen-sensitive 2-hydroxyisocaproyl-CoA dehydratase (CD0398, *hadC*), were both upregulated under heat stress, with the remaining proteins in this operon being just under the 1.5 fold cutoff. The absence of hadI in the GeLC-MS proteome is unsurprising: Kim et al. [72] demonstrated that only sub stoichiometric amounts of this activator are required for full activity of the dehydratase, thus it's abundance is likely to be much lower than the other enzymes encoded by this operon. Indeed, in the last step of the reduction, the electron recycles on the dehydratase for up to 10,000 turnovers until another hadI catalysed activation is required [73], demonstrating the efficiency of this evolutionarily ancient metabolic process. Our systems biology data is therefore consistent with the *in vitro* biochemistry and stoichiometry elucidated for this pathway – which we have now identified in three independent proteomics investigations, thereby emphasising its importance during heat stress in *C. difficile*.

Transcript data shows that during two quite different heat treatments [28,42], no significant changes occurred in the expression of genes within this operon. However, exposure of *C. difficile* 630 to other stresses including pH, oxygen and antibiotics led to upregulation of transcripts [28] suggesting that the reductive leucine fermentation pathway may be regulated as part of a more general stress response, or possibly in response to changes in global ccpA-mediated transcriptional regulation. While a polycistronic mRNA is proposed to be produced, ribosome binding sites exist upstream of several of the genes, including *hadI* [72], which may allow regulation of protein abundances via translational control. This could in some part explain the similar levels of transcript detected for all genes in the operon during heat stress [42], despite variations in the abundance of the cognate proteins. The oxidative branch of the leucine fermentation pathway, leading to isovalerate, is mediated by a ferredoxin (CD0115) in tandem with a 2-keto-isovalerate ferredoxin reductase encoded by CD0116-CD0118 (α, β and γ subunits, respectively) – only ferredoxin was upregulated in the current investigation, although our iTRAQ data [40] showed upregulation of the α subunit of the 2-keto-isovalerate ferredoxin reductase (CD0116) by 1.6 fold and an upward perturbation of CD0117 and CD0118 by 1.45 and 1.4-fold, respectively.

A putative aminoacid aminotransferase encoded by CD2532 was the most up regulated protein (4.7 fold) in the current work, correlating well with the iTRAQ data in which it was the second most upregulated protein, at 3.4-fold. Interestingly, the CD2532 transcript does not change significantly under heat stress as revealed by both microarray and qRT-PCR analysis [42]. CD2531 and CD2532 are predicted to generate a bicistronic messenger and indeed the transcript for CD2531 was also unchanged [42]. The protein encoded by CD2532 belongs to the class VI pyridoxal phosphate-dependent alanine-glyoxylate aminotransferase (AGAT) family, homodimeric proteins that catalyse the transamination of glyoxylate to glycine [70]. The gene is well conserved across the genus *Clostridium* and other gut microbes including *Roseburia hominis*, *R. intestinalis* and *Eubacterium* and *Fusobacterium* spp. It has been suggested that PLP-dependent enzymes (representing ~1.5% of prokaryotic genes) might represent useful targets for therapeutic agents [74] and indeed AGAT has been shown to be required for

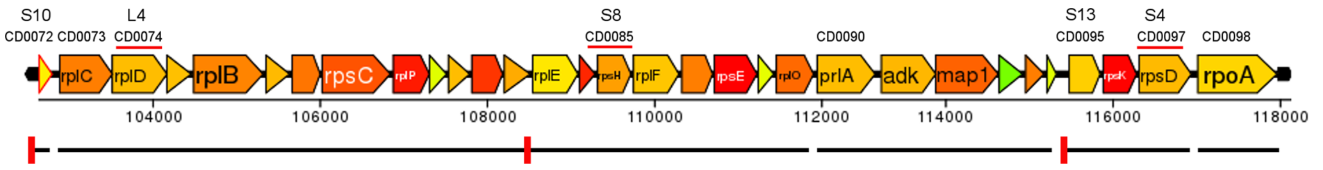


**Figure 4. Organisation of the S10-spx-α region in the genome of *Clostridium difficile* strain 630.** Black line: Biocyc predicted transcriptional units. Red boxes: Location of autogenous RNA regulatory structures in *Escherichia coli*. Orange underscore: protein with predicted regulatory function.
doi:10.1371/journal.pone.0088960.g004

pathogenesis of *Magnaporthe oryzae*, the causative agent of rice blast disease [75]. However, whilst the precise function of this protein is not yet elucidated within *C. difficile*, we have clear evidence for post-transcriptional regulation of its abundance by an as yet unknown mechanism.

## Cellular Information flow

Our previous data has indicated a robust system-wide response of *C. difficile* to clinical heat stress. iTRAQ labelling proteomics analysis [40] revealed no significant changes in the abundance of the 40 ribosomal proteins detected and our previous microarray analysis [42] furthermore suggested that all but three (30S S11, 30S S8, and 30S S1) of the transcripts were unchanged. However the current data indicates that the abundance of a number of the large subunit ribosomal proteins (L2, L3, L4, L5, L10, L20) are decreased under heat stress. The recent work of Hockett et al. [76] has shown that, in the plant pathogen *Pseudomonas syringae*, many genes associated with translation are downregulated at temperatures higher than those where the organism's pathogenicity is usually expressed. The virulence of *C. difficile* is accepted to be 'set' at 37°C and it is to be expected that many genes encoding traits important for host-microbe interactions, including protein synthesis, will be thermo-regulated. Indeed, we noted that the key virulence factor, TcdA (CD0663), was unique to the 37°C proteome as was DNA binding protein HU (CD3496), which is known to be expressed more highly during rapid growth [77] (Table S3). The ribosome, of which there may be up to $10^7$ within a single cell, is emerging as a key player in proteome quality control and homeostasis, a molecular machine monitoring all aspects of protein synthesis during translation [78]. In the current work we identified 35 ribosomal proteins, of which 11 were modulated under heat stress (Table S4). We previously detected a similarly large number of these highly abundant ribosomal proteins, some of which were modulated, within the proteome of *Ochrobactrum anthropi* [48]. The emPAI protocol appears to generate a more dynamic result than that observed using isobaric labelling – for example, the ribosomal protein 50S L4 (*rplD*, CD0074) was the most downregulated protein in this investigation at 5.49 fold down.

Ribosomal protein L4 forms part of the constriction site between the peptidyl transfer centre of the ribosome and the exit tunnel at the other end of the large subunit. This constriction site has been implicated in communication between the interior of the ribosome and the outside: certain protein sequences cause translation to stall in response to cellular signals including tryptophan and secA. In addition, the shape of the nascent polypeptide can alter overall ribosome structure, further influencing protein trafficking [78]. Indeed, one question for future research raised by Pechmann et al. [78] is the extent to which the ribosome tunnel communicates with factors at the ribosome exit site, including molecular chaperones such as trigger factor, N-acetyl transferases and translocation factors such as secA.

In addition to influencing communication pathways from the inside to the outside of the ribosome, L4 can also function as a transcriptional repressor and, independently, as a translational repressor. In *E. coli*, L4 is an essential gene and regulates the S10 ribosomal protein operon by binding to the highly structured mRNA within the S10 leader some 30 bp upstream of the S10 gene, causing premature termination of transcription [79,80]. In *E. coli*, the absence of L4 would consequently lead to an increase in S10 operon output. Our proteomic and transcript datasets do not support this model of regulation in *C. difficile* and it may be that in *C. difficile*, S10 regulation is more akin to that of the Gram positive model organism, *B. subtilus*. In *B. subtilus*, L4 is again an essential gene and a single 15 kbp transcriptional unit encompassing the S10, *spc* and α gene clusters is predicted [81,82]. In *C. difficile* strain 630, the S10 operon is encoded by CD0072 – CD0081. *In silico* analysis using the biocyc operon predictor [83] suggests that the S10 gene exists in a transcriptional unit by itself, with a further four transcriptional units (Figure 4) encompassing L3–L15 (CD0073, *rplC* – CD0089, *rplO*), *secY* – L36 (CD0090, *prlA* – CD0094A, *rpmJ*), S13 – S4 (CD0095, *rpsM* – CD0097, *rpsD*) and DNA directed RNA polymerase α subunit (CD0098, *rpoA*). Thus the transcriptional arrangement as predicted for *C. difficile* is quite different to that of *E. coli* and, possibly, *B. subtilus*. The current GeLC-MS data indicates that abundance of the regulatory protein L4 (CD0074, *rplD*) is 5.49 fold lower under heat stress and that L2 (CD0076, rplB) and L5 (CD0084, *rplE*) are each down regulated by some 3-fold. In addition, S8 (CD0085, *rpsH*), which is also predicted to play a regulatory role in *E. coli*, was upregulated by 2.1-fold, a change corroborated by our microarray data in which S8 was upregulated 1.8 fold [42]. Autogenous RNA regulatory structures are predicted to exist upstream of certain ribosomal protein genes including S10, L5 and S13 within a diversity of bacteria [84], however no such regulatory RNA structures have been predicted thus far within the *rps* leaders in *C. difficile*, possibly as a result of very different RNA secondary structures [80,84,85] in this organism. It is as yet unclear if L4, L8 or S4 proteins influence regulation of the ribosomal protein gene operons in *C. difficile* and this may be an area for future investigations.

## Molecular Chaperones and protein folding/export

Of the class I heat shock proteins, GroES (CD0193, 2.6 fold) was upregulated, commensurate with its upregulation at the transcript level of 2.04 fold [42], however its co chaperone GroEL was unchanged at 1.26 fold up (2.6 fold up in array), as was the DnaK chaperone at 1.29 fold up (2.18 fold up in array). We did not identify DnaJ or GrpE proteins in the combined proteome, however grpE – upregulated by 2.4 fold in the array – was one of the proteins unique to the 37°C proteome (Table S3) although it should be noted that we have a smaller overall proteome from the GeLC/MS approach than from iTRAQ. The Class III heat shock proteins clpP1 (CD3305, 1.9 fold up) and clpB (CD2020, 4.289 fold up) were up regulated, as per their iTRAQ data. The abundance of the class IV heat shock protein htpG (CD0273, HSP90) did not change. ClpP1 was unchanged in the array, and while raw expression data for both clpB and htpG suggested

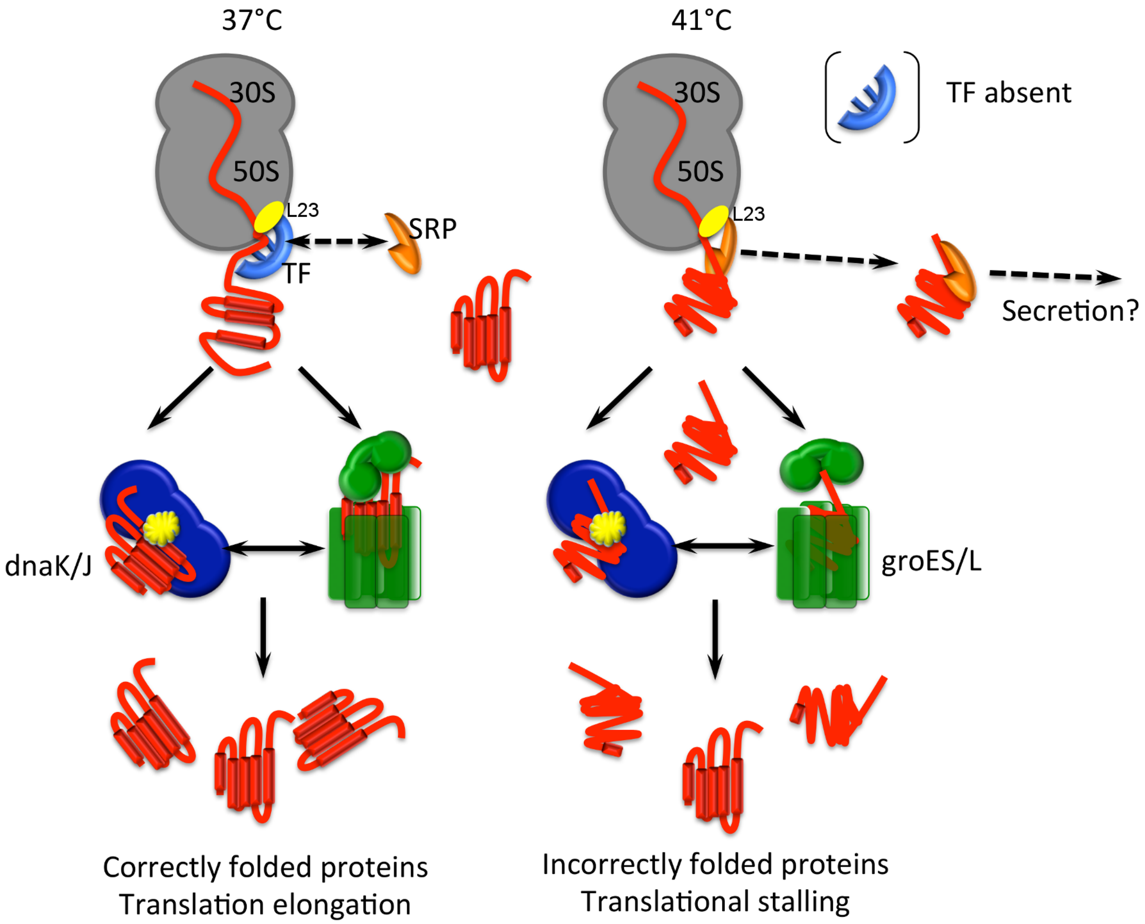


**Figure 5. Proposed model for translational stalling under heat stress in *Clostridium difficile* strain 630.** Trigger factor (TF) docks with L23 on the ribosome and is the first protein to interact with nascent polypeptides as they emerge from exit site on the ribosome, preventing or reversing premature protein folding. At 37°C there is competitive association of the signal recognition particle (SRP) and TF, both of which interact with L23, with nascent chains emerging from the ribosome: TF inhibits binding of the SRP to proteins destined to remain in the cytoplasm and these are passed to the dnaJ/K/GrpE and GroES/L chaperone systems, resulting in correctly folded mature cytosolic proteins. Upon temperature upshift to 41°C, however, decreased abundance of trigger factor enables non client proteins to be targeted for export by the SRP, and consequently fewer proteins are presented to the dnaJ/K/GrpE and GroES/L systems. Under heat stress, the dnaJ/K/grpE system is titrated by misfolded proteins, resulting in decreased stringency of protein quality control at the ribosome exit port: this prevent nascent chains from emerging cleanly from the ribosome thereby causing translational stalling and decreased growth rates.
doi:10.1371/journal.pone.0088960.g005

upregulation of their transcripts [42], their *p* values in the array dataset were $>0.05$ for both genes.

The greA protein was originally identified as a transcription elongation/cleavage factor but has recently been shown to possess a molecular chaperone function. GreA (CD3553) suppresses protein aggregation and, much like clpB, promotes the reactivation of denatured proteins. Thus it confers resistance to heat and oxidative stresses [86] and in in the current work was downregulated 2.2 fold by heat stress.. As one of the most abundant transcription factors in the cell, GreA provides a link between RNA polymerase/transcription apparatus and protein quality control by interacting with ribosome subunits and chaperones including DnaK, DnaJ, GroES and ClpX [86]. The reason for GreA protein downregulation is unclear at present – the transcript did not change in our array experiment [42] – however it is known to stimulate the endonucleolytic activity of RNA polymerase when bound to the $\beta'$ subunit [87,88], thus allowing transcription to continue past template-encoded arresting sites. Thus, in the absence of GreA, there will tend to be an increase in transcriptional pausing and therefore a global decrease in transcription and translation rates, an hypothesis strengthened by our observation that elongation factor P (CD1246, *efp*), an essential gene required for peptidyl transferase activity and the rescue of stalled ribosomes [89,90], was down regulated by 3.3 fold, although it's transcript was unchanged in the array [42].

During translation, the smooth transition from the inside of the polypeptide exit tunnel of the ribosome to the outside ribosome surface is most likely facilitated by the presence of ribosome-associated chaperone systems [91]. Trigger factor is a cytosolic ATP-independent, ribosome exit tunnel port-bound chaperone found in all eubacteria and, as the first protein to interact with nascent polypeptides as they emerge from exit site on the ribosome (Figure 5), is the best studied of the ribosome associated chaperones [78]. TF binds cyclically to L23 – unchanged in our three datasets – on the ribosome [92] and in accordance with our previous transcriptomic analysis [42], where it was down regulated by 1.57 fold , TF (CD3306, *tig*) was downregulated by some 4.9-fold under heat stress, which initially appears somewhat counter-intuitive. However in *E. coli*, TF is not a heat stress inducible protein and thus is not required for viability at high temperatures;

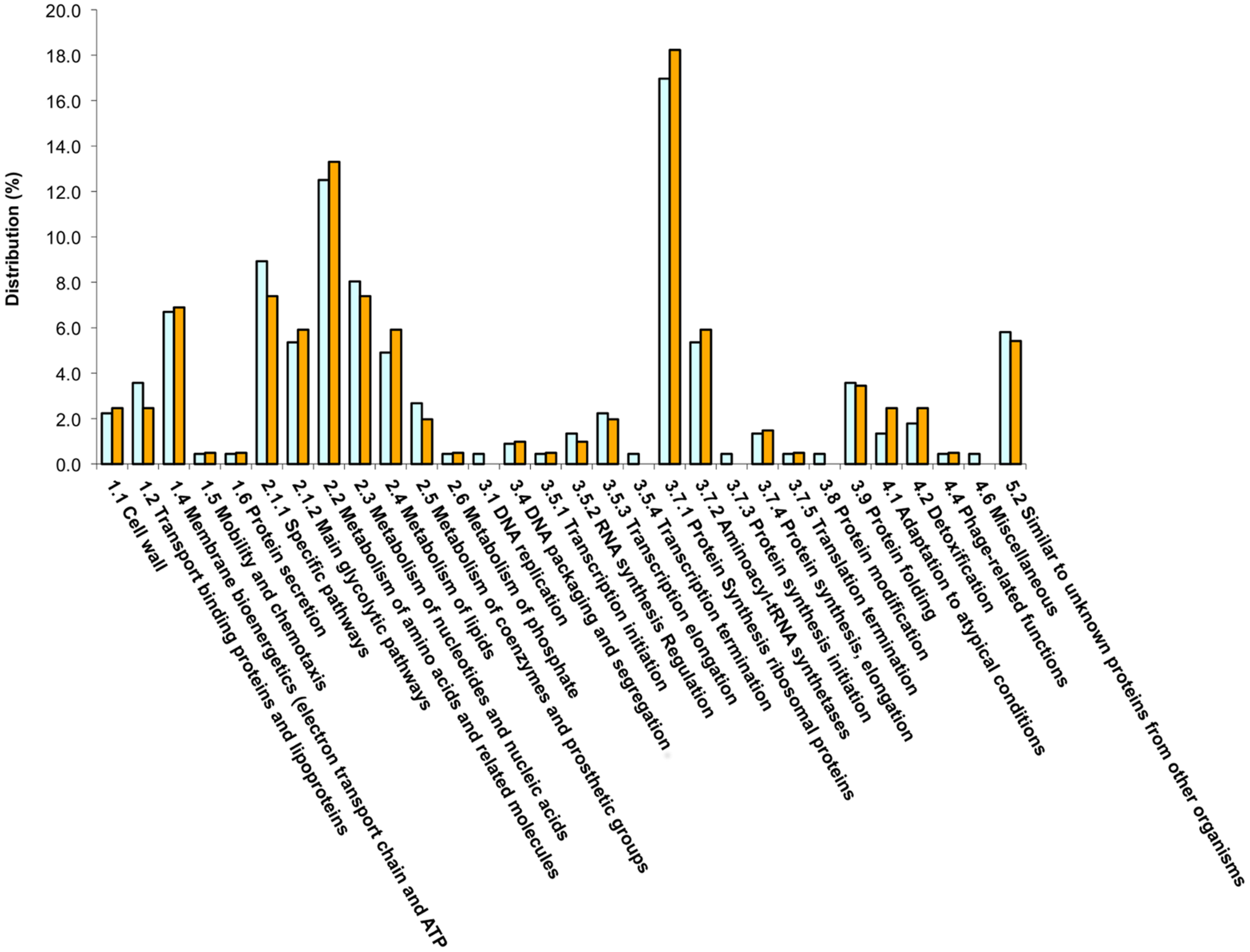


**Figure 6. Functional category distribution changes (% of proteins identified) in the 37°C and 41°C GeLC/MS proteomes from *Clostridium difficile* strain 630.** Blue: 37°C proteome; Orange: 41°C proteome.
doi:10.1371/journal.pone.0088960.g006

rather, it is proposed to play a role in protecting cells against low temperatures, under which conditions its abundance increases [93]. TF can actually prevent or reverse premature protein folding at a molecular level and thus plays a role in preventing premature or mis-folding of nascent polypeptide chains at lower temperatures [94]. At higher temperatures however, following interaction with TF, the DnaK chaperone system, including the DnaJ and GrpE co-chaperones, becomes more important, assisting *de novo* folding of cytosolic proteins both co- and post-translationally [94].

TF also influences protein export via the Sec system – the abundance of whose gene products were largely unchanged in our experiments [40,42]. Under normal circumstances, there is competitive association of the signal recognition particle (SRP, encoded in *C. difficile* by CD1251 – *ftsY*) and TF with nascent chains emerging from the ribosome [91]. The hydrophobic leaders of proteins destined for export via the two Sec systems of *C. difficile* [25] cause TF to dissociate thereby allowing an increase in the association of the SRP with the ribosome – which is subsequently targeted to the membrane via interaction between the SRP (ftsY, CD1251) and the SRP receptor (CD1252, *ffH*). TF, in turn, inhibits binding of the SRP to the less hydrophobic leaders of non-client proteins destined to remain in the cytoplasm (Figure 4). Thus it is clear that an absence of TF will tend to accelerate protein export [95,96], or certainly, the targeting of proteins to the secYEG translocon. Indeed, the wet weight of protein recovered from trichloroacetic acid precipitations of culture supernatant proteins from equal volumes of 37°C and 41°C grown-cultures of *C. difficile* strain 630 showed a ~40% increase in protein from the 41°C culture (Ternan, unpublished data). Whether this is due to increased traffic through the secYEG translocon under heat stress remains to be determined although it is likely, given that TF and dnaK share the same substrates [97]. The actions of TF and dnaK – which we have previously shown is upregulated under heat stress [40,42] – overlap to ensure continued accuracy of ribosomal output irrespective of the environmental conditions. However, under proteotoxic stress, which may be defined as the intracellular accumulation of misfolded or mistargetted proteins, translation elongation is often attenuated as cells reduce global protein synthesis. This happens in bacteria under most, if not all, types of adverse conditions [98]. Association of the *dnaK* chaperone with the ribosome decreases under proteotoxic stress as it is titrated by misfolded proteins in the cytosol: we propose that this, and the decreased abundance of TF, will prevent nascent chains from

emerging cleanly from the ribosome thereby causing translational stalling (Figure 5).

### Motility and Flagella

With regard to motility and as per our iTRAQ and microarray analyses [40,42] we again noted downregulation of the flagellar filiament protein, FliC (CD0239). The recent work of Kitagawa et al. [99] showed that in *E. coli*, FliC is post-translationally negatively regulated by the degradative action of the clpX/ClpP1 protease system on the master regulator of flagellar biosynthesis, flhDC. We noted upregulation of the clpP1 bipartite protease (CD3305, 1.9 fold up) in the current work, and of both clpP1 and the substrate binding clpX in our iTRAQ dataset [40], while their transcripts did not change [42]. The presence and identity, therefore, of homologues of flhDC in *C. difficile* remains to be established. Kitagawa et al. [99] suggest that in their model of flagellar regulation in *E. coli*, post-translational regulation contributes more to the control of flagellar biogenesis than transcriptional control: this is consistent with our observation that expression of the putative flagellar regulatory genes did not change under heat stress [42].

### Virulence factors

The main virulence factors of *C. difficile* are the host damaging toxins, A and B, and the transmissible agents, the spores. In addition, a number of other factors have been identified as important for *C. difficile* pathogenesis [100], including the binary toxin found in certain ribotypes (but not in CD630), proteins associated with motility such as FliC and those associated with adhesion (e.g. SLPs, cwps, fibronectin binding proteins). Yet other proteins such as haemolysins and collagenases allow host interaction and immune evasion. By and large, most of these virulence factor transcripts are downregulated at 41°C [42], although within the proteomes the picture is less clear since we do not have total global proteome coverage. However, integration of our proteome data with the array data does suggest down regulation of *C. difficile* virulence under heat stress. In terms of maintenance of cellular metabolic activities and stress survival however, we noted that changes in the % distribution of functional categories (Figure 6) indicated an increase in proteins associated with both detoxification and adaptation to atypical conditions as well as those associated with amino acid and lipid (fatty acid) metabolism. There was also a decrease in proteins associated with the initiation of protein synthesis and with transcriptional elongation, and these observations are supported by the proteins identified in the, 'unique' 37°C and 41°C proteomes.

## Conclusion

An important aspect of systems biology research is that in order to be able to construct an accurate model for a system, it is necessary to have multiple measurements of changes in the components of that system. Consequently, the integration of transcriptomic and proteomic data is required to obtain a comprehensive molecular characterisation of a biological system [101]. Many investigators look at proteomes, or other 'omes, individually in isolation. Fewer investigate and attempt to tease apart changes in both proteins and transcripts – and those that do so do not always achieve perfect protein/mRNA abundance correlations: the lack of correlation between transcripts and protein abundance is well known. Thus, there is a requirement for additional studies that will allow links to be defined, if they exist, between the various parts of the central dogma of biological information flow. We have analysed the same biological conditions using different experimental procedures and, crucially, at different times, to understand how selection of a proteomics workflow influences the quantity and quality of data generated. This has allowed us to validate the biological picture of heat stress response and adaptation in *C. difficile* and develop some perspectives on the two different proteomic workflows. Analysis of cost, time and data quality associated with iTRAQ and GeLC/MS indicates that reproducibility is very good under both workflows. However, the cost to researchers that do not have on demand LC/MS facilities could be considerable. For biological duplicate GeLC/MS experiment, as here, where 8 gel fractions are injected 3 times across four samples, LC/MS analysis could take perhaps 4 weeks' machine time at a cost of almost £10,000, assuming a conservative LC/MS cost of £100 per sample: this is before proceeding to carry out the emPAI and fold change calculations. On the other hand, the iTRAQ 4 plex kit costs in the region of £1200, so our five strong cation exchange fractions [40] would comprise some £1500 worth of LC/MS time, in addition to the entire workflow – including sample preparation and automated protein plot software analysis – taking less than a week. Thus, from a technical standpoint and in terms of proteome coverage, researcher time not withstanding, iTRAQ labelling will generate robust quantitative data more rapidly and economically.

The data presented here indicates a decrease in transcription and translation under heat stress that may well feed back into transcriptional control of ribosomal protein genes. We believe that the key players may be misfolded cytosolic proteins, coupled with the ability of the system to deal with these. It is clear that *C. difficile* emerges relatively unscathed by temperature upshift to 41°C, despite considerable protein synthesis/folding and energy conservation stress and it is clear that *C. difficile* can compensate for misfolding events by increasing the abundance of chaperone proteins such as GroES/L and the dnaK system [40,42]. Therefore a clearer picture of decreased virulence, combined with transcriptional and translational stalling mediated by a potentially complex network of regulatory mechanisms, emerges.

## Supporting Information

**File S1 PROVALT output html file from 37°C single lane GeLC/MS, single injection, 177 proteins.**
(HTML)

**File S2 PROVALT output html file from 37°C pseudo-replicate single lane GeLC/MS, single injection, 202 proteins.**
(HTML)

**File S3 PROVALT output html file from 37°C biological replicate, single lane GeLC/MS, 3 injections, 163 proteins.**
(HTML)

**File S4 PROVALT output html file – overall 37°C proteome, 224 proteins.**
(HTML)

**File S5 PROVALT output html file – overall 41°C proteome, 202 proteins.**
(HTML)

**Table S1 Overall 37°C proteome with emPAI and bioinformatics analysis xls file.**
(XLS)

**Table S2 Overall 41°C proteome with emPAI and bioinformatics analysis xls file.**
(XLS)

**Table S3 Proteins unique to either 37°C or 41°C proteomes xls file.**
(XLSX)

**Table S4 Combined final emPAI master list 41°C v 37°C xls file.**
(XLS)

## Author Contributions

Conceived and designed the experiments: NGT GM RLJG. Performed the experiments: SJ RLJG NGT. Analyzed the data: NGT SJ RLJG. Wrote the paper: NGT SJ RLJG GM.

## References

1. Bartlett JG (2008) Historical perspectives on studies of *Clostridium difficile* and *C. difficile* infection. Clin Infect Dis 46 (Suppl 1): s4–s11.
2. Pepin J, Saheb N, Coulombe MA, Alary ME, Corriveau MP, et al. (2005) Emergence of fluoroquinolones as the predominant risk factor for *Clostridium difficile*-associated diarrhea: A cohort study during an epidemic in Quebec. Clin Inf Dis 41: 1254–1260.
3. Rupnik M, Wilcox MH, Gerding DN (2009) *Clostridium difficile* infection: new developments in epidemiology and pathogenesis. Nature Rev Microbiology 7: 526–536.
4. Stabler RA, He M, Dawson L, Martin M, Valiente E, et al. (2009) Comparative genome and phenotypic analysis of *Clostridium difficile* 027 strains provides insight into the evolution of a hypervirulent bacterium. BMC Genome Biol 10: R102. doi:10.1186/gb-2009-10-9-r102
5. Sun X, Wang H, Zhang Y, Chen C, Davis B, et al. (2012) Mouse Relapse Model of *Clostridium difficile* Infection. Infect Immun 79: 2856–2864.
6. Dubberke ER, Olsen MA (2012) Burden of *Clostridium difficile* on the healthcare system. Clin Infect Dis 55 (suppl_2): s88–s92.
7. European Center for Disease Control and Prevention. Available at: http://ecdc.europa.eu/EN/HEALTHTOPICS/*CLOSTRIDIUM_DIFFICILE_*INFECTION/Pages/index.aspx Accessed Jul24, 2013.
8. Voth DE, Ballard JD (2005) *Clostridium difficile* toxins: Mechanism of action and role in disease. Clin Microbiol Rev 18: 247–263.
9. Kuehne SA, Cartman ST, Heap JT, Kelly ML, Cockayne A, et al. (2010) The role of toxin A and toxin B in *Clostridium difficile* infection. Nature 467: 711–713.
10. Carter GP, Rood JI, Lyras D (2012) The role of toxin A and toxin B in the virulence of *Clostridium difficile*. Trends Microbiol 20: 21–29.
11. Stubbs SLJ, Brazier JS, O'Neill GL, Duerden BI (1999) PCR Targeted to the 16S–23S rRNA gene intergenic spacer region of *Clostridium difficile* and construction of a library consisting of 116 different PCR ribotypes. J Clin Microbiol 37: 461–463.
12. Pépin J, Valiquette L, Alary ME, Villemure P, Pelletier A, et al. (2004) *Clostridium difficile*-associated diarrhea in a region of Quebec from 1991 to 2003: a changing pattern of disease severity. Can Med Assoc J 171: 466–472.
13. Freeman J, Bauer MP, Baines SD, Corver J, Fawley WN, et al. (2010) The changing epidemiology of *Clostridium difficile* infections. Clin Microbiol Rev 23: 529–549.
14. Wilcox MH, Shetty N, Fawley WN, Shemko M, Coen P, et al (2012) Changing epidemiology of *Clostridium difficile* infection following the introduction of a national ribotyping based surveillance scheme in England. Clin Infect Dis 55: 1056–1063.
15. Cartman ST, Heap JT, Kuehne SA, Cockayne A, Minton NP (2010) The emergence of 'hypervirulence' in *Clostridium difficile*. Intl J Med Microbiol 300: 387–395.
16. Sirard S, Valiquette L, Fortier LC (2011) Lack of association between clinical outcome of *Clostridium difficile* infections, strain type, and virulence-associated phenotypes. J Clin Microbiol 49: 4040–4046.
17. Burns DA, Heeg D, Cartman ST, Minton NP (2011) Reconsidering the sporulation characteristics of hypervirulent *Clostridium difficile* BI/NAP1/027. PLoS ONE 6(9): e24894.
18. Sebaihia M, Wren BW, Mullany P, Fairweather NF, Minton NP, et al. (2006) The multidrug-resistant human pathogen *Clostridium difficile* has a highly mobile, mosaic genome. Nat Genet 38: 779–786.
19. He M, Sebaihia M, Lawley TD, Stabler RA, Dawson LF, et al. (2010) Evolutionary dynamics of *Clostridium difficile* over short and long time scales. Proc Natl Acad Sci U S A 107: 7527–7532.
20. Forgetta V, Oughton MT, Marquis P, Brukner I, Blanchette R, et al. (2011) Fourteen-genome comparison identifies DNA markers for severe-disease-associated strains of *Clostridium difficile*. J Clin Microbiol 49: 2230–2238.
21. Heap JT, Kuehne SA, Ehsaan M, Cartman ST, Cooksley CM, et al. (2010) The ClosTron: Mutagenesis in *Clostridium* refined and streamlined. J Microbiol Meth 80: 49–55.
22. Heap JT, Pennington OJ, Cartman ST, Carter GP, Minton NP (2007) The ClosTron: A universal gene knock-out system for the genus *Clostridium*. J Microbiol Meth 70: 452–464.
23. Twine SM, Reid CW, Aubry A, McMullin DR, Fulton KM, et al. (2009) Motility and flagellar glycosylation in *Clostridium difficile*. J Bacteriol 191: 7050–7062.
24. Dingle TC, Mulvey GL, Armstrong GD (2011) Mutagenic analysis of the *Clostridium difficile* flagellar proteins, FliC and FliD, and their contribution to virulence in hamsters. Inf Immun 79: 4061–4067.
25. Fagan RP, Fairweather NF (2011) *Clostridium difficile* has two parallel and essential Sec secretion systems. J Biol Chem 286: 27483–27493.
26. Saujet L, Monot M, Dupuy B, Soutourina O, Martin-Verstraete I (2011). The key sigma factor of transition phase, SigH, controls sporulation, metabolism, and virulence factor expression in *Clostridium difficile*. J Bacteriol 193: 3186–3196
27. Olling A, Seehase S, Minton NP, Tatge H, Schröter S, et al. (2012) Release of TcdA and TcdB from *Clostridium difficile* cdi 630 is not affected by functional inactivation of the tcdE gene. Microb Pathog 52: 92–100.
28. Emerson JE, Stabler RA, Wren BW, Fairweather NF (2008) Microarray analysis of the transcriptional responses of *Clostridium difficile* to environmental and antibiotic stress. J Med Microbiol 57: 757–764.
29. Scaria J, Janvilisri T, Fubini S, Gleed RD, McDonough SP, et al. (2011) *Clostridium difficile* transcriptome analysis using pig ligated loop model reveals modulation of pathways not modulated *in vitro*. J Infect Dis 203: 1613–1620.
30. Janoir C, Denève C, Bouttier S, Barbut F, Hoys S, et al. (2013) Adaptive Strategies and Pathogenesis of *Clostridium difficile* from *In Vivo* Transcriptomics. Infect Immun 81(10): 3757–3769.
31. Olivares-Hernández R, Bordel S, Nielsen J (2011) Codon usage variability determines the correlation between proteome and transcriptome fold changes. BMC Syst Biology 5:33.
32. Soutourina OA, Monot M, Boudry P, Saujet L, Pichon C, et al. (2013) Genome-Wide Identification of Regulatory RNAs in the Human Pathogen *Clostridium difficile*. PLoS Genet 9(5): e1003493.
33. Graham RLJ, Pollock CE, Ternan NG, McMullan G (2006) Top-down proteomic analysis of the soluble sub-proteome of the obligate thermophile, *Geobacillus thermoleovorans* T80: Insights into its cellular processes. J Proteome Res 5: 822–828.
34. Graham RLJ, O'Loughlin SN, Pollock CE, Ternan NG, Weatherly DB, et al. (2006) A combined shotgun and multidimensional proteomic analysis of the insoluble subproteome of the obligate thermophile, *Geobacillus thermoleovorans* T80. J Proteome Res 5: 2465–2473.
35. Graham RLJ, Pollock CE, O'Loughlin SN, Ternan NG, Weatherly DB, et al. (2006) Multidimensional proteomic analysis of the soluble sub-proteome of the emerging nosocomial pathogen, *Ochrobactrum anthropi*. J Proteome Res 5: 3145–3153.
36. Wright A, Wait R, Begum S, Crossett B, Nagy J, et al. (2005) Proteomic analysis of cell surface proteins from *Clostridium difficile*. Proteomics 5: 2443–2452.
37. Lawley TD, Croucher NJ, Yu L, Clare S, Sebaihia M, et al. (2009) Proteomic and genomic characterization of highly infectious *Clostridium difficile* 630 spores. J Bacteriol 191: 5377–5386.
38. Jain S, Graham RLJ, McMullan G, Ternan NG (2010) Proteomic analysis of the insoluble subproteome of *Clostridium difficile* strain 630. FEMS Microbiol Lett 312: 151–159.
39. Boetzkes A, Felkel KW, Zeiser J, Jochim N, Just I, et al. (2012) Secretome analysis of *Clostridium difficile* strains. Arch Microbiol 194(8): 675–687.
40. Jain S, Graham C, Graham RLJ, McMullan G, Ternan NG (2011) A quantitative proteomic analysis of the heat stress response in *Clostridium difficile* strain 630. J Proteome Res 10: 3880–3890.
41. Janvilisri T, Scaria J, Teng C-H, McDonough SP, Gleed RD, et al. (2012) Temporal Differential Proteomes of *Clostridium difficile* in the Pig Ileal-Ligated Loop Model. PLoS ONE 7(9): e45608.
42. Ternan NG, Jain S, Sirivastava M, McMullan G (2012) Comparative transcriptional analysis of clinically relevant heat stress response in *Clostridium difficile* strain 630. PLoS ONE 7(7): e42410.
43. Ross PL, Huang YLN, Marchese JN, Williamson B, Parker K, et al. (2004) Multiplexed protein quantitation in *Saccharomyces cerevisiae* using amine-reactive isobaric tagging reagents. Mol Cell Proteomics 3: 1154–1169.
44. Ishihama Y, Oda Y, Tabata T, Sato T, Nagasu T, et al. (2005) Exponentially Modified Protein Abundance Index (emPAI) for estimation of absolute protein amount in proteomics by the number of sequenced peptides per protein. Mol Cell Proteomics 4: 1265–1272.
45. Weatherly DB, Atwood JA, III, Minning TA, Cavola C, Tarleton RL, et al. (2005) A heuristic method for assigning a false-discovery rate for protein identifications from Mascot database search results. Mol Cell Proteomics 4: 762–772.
46. Ulrich PN, Jimenez V, Park M, Martins VP, Atwood J, III, et al. (2011) Identification of Contractile vacuole proteins in *Trypanosoma cruzi*. PLoS ONE 6(3): e18013.
47. Wright PC, Noirel A, Ow S-Y, Fazeli A (2012) A review of current proteomics technologies with a survey on their widespread use in reproductive biology investigations. Theriogenology 77: 738–765.
48. Graham RLJ, Sharma MK, Ternan NG, Weatherly DB, Tarleton RL, et al. (2007) A semi-quantitative GeLC-MS analysis of temporal proteome expression

in the emerging nosocomial pathogen *Ochrobactrum anthropi*. BMC Genome Biol 8, R110.

49. Bradford M (1976) A rapid and sensitive method for the quantitation of microgram quantities of protein utilizing the principle of protein-dye binding. Anal Biochem 72:248–254.
50. Cagney G, Amiri S, Premawaradena T, Lindo M, Emili A (2003) *In silico* proteome analysis to facilitate proteomics experiments using mass spectrometry. Proteome Sci 1: 5.
51. Gardy JL, Laird MR Chen F, Rey S, Walsh CJ, et al. (2005) Psortb v.2.0: Expanded prediction of bacterial protein subcellular localization and insights gained from comparative proteome analysis. Bioinformatics 21: 617–623.
52. Bendtsen J, Nielsen H, von Heijne G, Brunak S (2004) Improved prediction of signal peptides: Signalp 3.0. J Mol Biol 340: 783–795.
53. Bendtsen J, Kiemer L, Fausboll A, Brunak S (2005) Non-classical protein secretion in bacteria. BMC Microbiol 5: 58.
54. Shinoda K, Tomita M, Ishihama Y (2010) emPAI Calc–for the estimation of protein abundance from large-scale identification data by liquid chromatography-tandem mass spectrometry. Bioinformatics 26(4): 576–577.
55. Graham RL, Pollock CE, O'Loughlin SN, Ternan N, Weatherly DB, et al. (2007) Multidimensional analysis of the insoluble sub-proteome of *Oceanobacillus iheyen*sis HTE831, an alkaliphilic and halotolerant deep-sea bacterium isolated from the Iheya ridge. Proteomics 7: 82–91.
56. De Angelis M, Di Cagno R, Huet C, Crecchio C, Fox PF, et al (2004) Heat shock response in *Lactobacillus plantarum*. Appl Environ Microbiol 70:1336–1346.
57. Paget,MS, Molle V, Cohen G, Aharonowitz Y, Buttner MJ (2001) Defining the disulphide stress response in *Streptomyces coelicolor* A3(2): identification of the sigmaR regulon. Mol Microbiol 42:1007–1020.
58. Graumann P, Schroder K, Schmid R, Marahiel MA (1996) Cold shock stress-induced proteins in *Bacillus subtilis*. J Bacteriol 178:4611–4619.
59. Fagan RP, Janoir C, Collignon A, Mastrantonio P, Poxton IR, et al. (2011) A proposed nomenclature for cell wall proteins of *Clostridium difficile*. J Med Microbiol 60(8): 1225–1228.
60. Karjalainen T, Waligora-Dupriet A-J, Cerquetti M, Spigaglia P, Maggioni A, et al. (2001) Molecular and genomic analysis of genes encoding surface-anchored proteins from *Clostridium difficile*. Infect Immun 69: 3442–3446.
61. Waligora AJ, Hennequin C, Mullany P, Bourlioux P, Collignon A, et al. (2001) Characterization of a cell surface protein of *Clostridium difficile* with adhesive properties. Infect Immun 69(4): 2144–2153.
62. Brown EM, Latimer C, Allsopp P, Ternan NG, McDougall GJ, et al. (2013) *In Vitro* and *In Vivo* Models of Colorectal Cancer: Anti-Genotoxic Activity of Berries. J Agric Food Chem, *in press*.
63. McLennan AG (2006) The Nudix hydrolase superfamily. Cellular and Molecular Life Sciences CMLS, 63(2): 123–143
64. Xu A, Desai AM, Brenner SE, Kirsch JF (2013) A continuous fluorescence assay for the characterization of Nudix hydrolases. Anal Biochem 437(2): 178–184.
65. Tkaczuk KL, A Shumilin I, Chruszcz M, Evdokimova E, Savchenko A, et al. (2013) Structural and functional insight into the universal stress protein family. Evol Appl 6(3): 434–449.
66. Chasteen TG, Fuentes DE, Tantaleán JC, Vásquez CC (2009) Tellurite: history, oxidative stress, and molecular mechanisms of resistance. FEMS Microbiol Rev 33(4): 820–832.
67. Karlsson S, Dupuy B, Mukherjee K, Norin E, Burman LG, et al. (2003) Expression of *Clostridium difficile* Toxins A and B and their sigma factor TcdD is controlled by temperature. Infect Immun 71: 1784–1793.
68. Luo X, Hsiao HH, Bubunenko M, Weber G, Court DL, et al. (2008) Structural and functional analysis of the *E. coli* NusB-S10 transcription antitermination complex. Mol Cell 32(6): 791–802.
69. Antunes A, Camiade E, Monot M, Courtois E, Barbut F, et al. (2012) Global transcriptional control by glucose and carbon regulator CcpA in *Clostridium difficile*. Nucleic Acids Res 40(21): 10701–10718.
70. Marchler-Bauer A, Zheng C, Chitsaz F, Derbyshire MK, Geer LY, et al. (2013) CDD: conserved domains and protein three-dimensional structure. Nucleic Acids Res 41(Database Issue): D384–52.
71. Kim J, Darley DJ, Buckel W, Pierik AJ (2008) An allylic ketyl radical intermediate in clostridial amino-acid fermentation. Nature 452: 239–242.
72. Kim J, Darley D, Buckel W (2005) 2-Hydroxyisocaproyl-CoA dehydratase and its activator from *Clostridium difficile*. FEBS J 272: 550–561.
73. Knauer SH, Buckel W, Dobbek H (2011) Structural basis for reductive radical formation and electron recycling in (R)-2-hydroxyisocaproyl-CoA dehydratase. J Am Chem Soc 133(12): 4342–4347.
74. Amadasi A, Bertoldi M, Contestabile R, Bettati S, Cellini B, et al. (2007) Pyridoxal 5′-phosphate enzymes as targets for therapeutic agents. Curr Med Chem 14(12): 1291–1324.
75. Bhadauria V, Banniza S, Vandenberg A, Selvaraj G, Wei Y (2012) Peroxisomal Alanine: Glyoxylate aminotransferase AGT1 is indispensable for appressorium function of the Rice Blast Pathogen, *Magnaporthe oryzae*. PLoS ONE 7(4): e36266.
76. Hockett KL, Burch AY, Lindow SE (2013) Thermo-regulation of genes mediating motility and plant interactions in *Pseudomonas syringae*. PLoS ONE 8(3): e59850.
77. Ali Azam T, Iwata A, Nishimura A, Ueda S, Ishihama A. (1999) Growth phase-dependent variation in protein composition of the *Escherichia coli* nucleoid. J Bacteriol 181(20): 6361–6370.
78. Pechmann S, Willmund F, Frydman J. (2013) The ribosome as a hub for protein quality control. Mol Cell 49(3): 411–421.
79. Zengel JM, Lindahl L (1985) Transcriptional control of the S10 ribosomal protein operon of *Escherichia coli* after a shift to higher temperature. J Bacteriol 163(1): 140–147.
80. Zengel JM, Lindahl (1996) A hairpin structure upstream of the terminator hairpin required for ribosomal protein L4-mediated attenuation control of the S10 operon of *Escherichia coli*. J Bacteriol 178(8): 2383–2387.
81. Li X, Lindahl L, Sha Y, Zengel JM (1997) Analysis of the *Bacillus subtilis* S10 ribosomal protein gene cluster identifies two promoters that may be responsible for transcription of the entire 15-kilobase S10-spc-β cluster. J Bacteriol 179(22): 7046–7054.
82. Akanuma G, Nanamiya H, Natori Y, Yano K, Suzuki S, et al. (2012) Inactivation of ribosomal protein genes in *Bacillus subtilis* reveals importance of each ribosomal protein for cell proliferation and cell differentiation. J Bacteriol 194(22): 6282–6291.
83. Karp PD, Paley SM, Krummenacker M, Latendresse M, Dale JM, et al. (2010) Pathway Tools version 13.0: Integrated Software for Pathway/Genome Informatics and Systems Biology. Brief Bioinform 11: 40–79.
84. Fu Y, Deiorio-Haggar K, Anthony J, Meyer MM (2013) Most RNAs regulating ribosomal protein biosynthesis in *Escherichia coli* are narrowly distributed to Gammaproteobacteria. Nucleic Acids Res 41(6): 3491–3503.
85. Weinberg Z, Wang JX, Bogue J, Yang J, Corbino K, et al. (2010) Comparative genomics reveals 104 candidate structured RNAs from bacteria, archaea, and their metagenomes. Genome Biol 11(3): R31.
86. Li K, Jiang T, Yu B, Wang L, Gao C, et al. (2012) Transcription elongation factor GreA has functional chaperone activity. PLoS ONE 7(12): e47521.
87. Iyer LM, Aravind L (2012). Insights from the architecture of the bacterial transcription apparatus. J Struct Biology 179(3): 299–319
88. Chlenov M, Masuda S, Murakami KS, Nikiforov V, Darst SA, et al. (2005) Structure and function of lineage-specific sequence insertions in the bacterial RNA polymerase beta' subunit. J Mol Biol 353: 138–154.
89. Aoki H, Dekany K, Adams SL, Ganoza MC (1997) The gene encoding the elongation factor P protein is essential for viability and is required for protein synthesis. J Biol Chem 272: 32254–32259.
90. Hersch SJ, Wang M, Zou SB, Moon K-M, Foster LJ, et al. (2013) Divergent protein dotifs direct elongation factor P-mediated translational regulation in *Salmonella enteric*a and *Escherichia coli*. mBio 4(2): e00180-13.
91. Kramer G, Boehringer D, Ban N, Bukau B (2009) The ribosome as a platform for co-translational processing, folding and targeting of newly synthesized proteins. Nat Struct Mol Biol 16(6): 58–597.
92. Schlunzen F, Wilson DN, Tian P, Harms JM, McInnes SJ, et al. (2005). The binding mode of the trigger factor on the ribosome: implications for protein folding and SRP interaction. Structure 13: 1685–1694.
93. Kandror O, Goldberg AL (1997) Trigger factor is induced upon cold shock and enhances viability of *Escherichia coli* at low temperatures. Proc Natl Acad Sci U S A 94(10): 4978–4981.
94. Hoffmann A, Becker AH, Zachmann-Brand B, Deuerling E, Bukau B, et al. (2012) Concerted action of the ribosome and the associated chaperone trigger factor confines nascent polypeptide folding. Mol Cell 48(1): 63–74.
95. Lee HC, Bernstein HD (2002) Trigger factor retards protein export in *Escherichia coli*. J Biol Chem 277: 43527–43535.
96. Ullers RS, Ang D, Schwager F, Georgopoulos C, Genevaux P (2007) Trigger factor can antagonize both SecB and DnaK/DnaJ chaperone functions in *Escherichia coli*. Proc Natl Acad Sci U S A 104: 3101–3106.
97. Deuerling E, Schulze-Specking A, Tomoyasu T, Mogk A, Bukau B (1999). Trigger factor and DnaK cooperate in folding of newly synthesized proteins. Nature 400: 693–696.
98. Liu B, Han Y, Qian SB. (2012) Cotranslational response to proteotoxic stress by elongation pausing of ribosomes. Mol Cell 49(3): 453–463.
99. Kitagawa R, Takaya A, Yamamoto T (2011) Dual regulatory pathways of flagellar gene expression by ClpXP protease in enterohaemorrhagic *Escherichia coli*. Microbiology 157(11): 3094–3103.
100. Vedantam G, Clark A, Chu M, McQuade R, Mallozzi M, Viswanathan VK (2012) C*lostridium difficile* infection: toxins and non-toxin virulence factors, and their contributions to disease establishment and host response. Gut Microbes 3(2): 121–134.
101. Resch A, Leicht S, Saric M, Pa'sztor L, Jakob A, et al. (2006) Comparative proteome analysis of *Staphylococcus aureus* biofilm and planktonic cells and correlation with transcriptome profiling. Proteomics 6: 1867–1877.
